# Pseudorabies virus gM and its homologous proteins in herpesviruses induce mitochondria-related apoptosis involved in viral pathogenicity

**DOI:** 10.1371/journal.ppat.1012146

**Published:** 2024-04-26

**Authors:** Qiongqiong Zhou, Deshi Shi, Yan-Dong Tang, Longfeng Zhang, Boli Hu, Chunfu Zheng, Li Huang, Changjiang Weng

**Affiliations:** 1 Division of Fundamental Immunology, State Key Laboratory of Animal Disease Prevention and Control, Harbin Veterinary Research Institute, Chinese Academy of Agricultural Sciences, Harbin, Heilongjiang, China; 2 Heilongjiang Provincial Key Laboratory of Veterinary Immunology, Harbin, Heilongjiang, China; 3 State Key Laboratory of Agricultural Microbiology, College of Veterinary Medicine, Huazhong Agricultural University, Wuhan, Hubei, China; 4 MOA Key Laboratory of Animal Virology, Zhejiang University Center for Veterinary Sciences, Hangzhou, Zhejiang, China; 5 Department of Microbiology, Immunology & Infection Diseases, University of Calgary, Calgary, Alberta, Canada; State University of New York Upstate Medical University, UNITED STATES

## Abstract

Apoptosis is a critical host antiviral defense mechanism. But many viruses have evolved multiple strategies to manipulate apoptosis and escape host antiviral immune responses. Herpesvirus infection regulated apoptosis; however, the underlying molecular mechanisms have not yet been fully elucidated. Hence, the present study aimed to study the relationship between herpesvirus infection and apoptosis *in vitro* and *in vivo* using the pseudorabies virus (PRV) as the model virus. We found that mitochondria-dependent apoptosis was induced by PRV gM, a late protein encoded by PRV *UL10*, a virulence-related gene involved in enhancing PRV pathogenicity. Mechanistically, gM competitively combines with BCL-XL to disrupt the BCL-XL-BAK complex, resulting in BCL-2-antagonistic killer (BAK) oligomerization and BCL-2-associated X (BAX) activation, which destroys the mitochondrial membrane potential and activates caspase-3/7 to trigger apoptosis. Interestingly, similar apoptotic mechanisms were observed in other herpesviruses (Herpes Simplex Virus-1 [HSV-1], human cytomegalovirus [HCMV], Equine herpesvirus-1 [EHV-1], and varicella-zoster virus [VZV]) driven by PRV gM homologs. Compared with their parental viruses, the pathogenicity of PRV-ΔUL10 or HSV-1-ΔUL10 in mice was reduced with lower apoptosis and viral replication, illustrating that *UL10* is a key virulence-related gene in PRV and HSV-1. Consistently, caspase-3 deletion also diminished the replication and pathogenicity of PRV and HSV-1 *in vitro* and in mice, suggesting that caspase-3-mediated apoptosis is closely related to the replication and pathogenicity of PRV and HSV-1. Overall, our findings firstly reveal the mechanism by which PRV gM and its homologs in several herpesviruses regulate apoptosis to enhance the viral replication and pathogenicity, and the relationship between gM-mediated apoptosis and herpesvirus pathogenicity suggests a promising approach for developing attenuated live vaccines and therapy for herpesvirus-related diseases.

## Author summary

Apoptosis plays a crucial role in eliminating virus-infected cells and maintaining tissue homeostasis. Herpesviruses have evolved numerous strategies for manipulating apoptosis. However, the molecular mechanisms by which herpesvirus infection induces apoptosis are not fully unclear. In this study, PRV was used as a model virus to elucidate the relationship between herpesvirus infection and apoptosis. We reported that PRV gM induced mitochondria-dependent apoptosis by competitively binding to the anti-apoptosis protein BCL-XL and activating the apoptosis effectors caspase-3/7/9 to enhance the viral replication and pathogenicity *in vitro* and in mice. Importantly, we found that the PRV gM homologs of herpesviruses (HSV-1, HCMV, EHV-1, and VZV) shared the same mechanism to promote apoptosis. Additionally, the reduced pathogenicity of PRV and HSV-1 in caspase-3^+/-^ mice suggested that caspase-3-mediated apoptosis driven by gM protein was a key regulator to the replication and pathogenicity of PRV and HSV-1. Our study firstly reveals the molecular mechanism by which PRV gM and its homologs in several herpesviruses regulate apoptosis to enhance the viral replication and pathogenicity. These findings provide novel insights for developing new vaccine candidates and antiviral drugs. Based on these findings, inhibiting apoptosis is a promising therapeutic strategy for treating herpesvirus-related diseases.

## Introduction

Herpesviruses are a large family of enveloped DNA viruses including the following three major subfamilies: α, β, and γ herpesvirus [1]. Herpes simplex virus 1 (HSV-1), pseudorabies virus (PRV), equine herpesvirus 1 (EHV-1), and varicella-zoster virus (VZV) belong to α herpesvirus, while human cytomegalovirus (HCMV) belongs to β herpesvirus. HSV-1 and PRV are closely related functionally with other herpesviruses; therefore, they are widely used as model viruses to study viral protein functions [2,3]. In addition to its natural host, pigs, PRV also infects other livestock, rodents, and wild animals, resulting in huge economic losses. Recently, the emergence of virulent PRV isolates and an increasing number of PRV-infected patients have indicated that PRV poses a potential threat to public health [4]. The PRV genome consists of approximately 150 kb linear double-stranded DNA, which encodes approximately 70 specific proteins, including 11 glycoproteins: glycoprotein B (gB), gC, gD, gE, gG, gH, gI, gK, gL, gM, and gN [5]. Similar to HSV-1, PRV *UL10* gene-encoded gM protein contains eight transmembrane domains and an N-glycosylated component [5]. In contrast, the gM homologs in HCMV, EHV-1, and VZV are encoded by *UL100*, *ORF52*, and *ORF50*, respectively. Interestingly, the PRV gM protein and its homologs in other herpesviruses are conserved throughout Herpesviridae, suggesting their significant role in the viral life cycle [1]. For example, PRV gM plays an essential role in virion assembly during viral infection, and *UL10* deletion reduces viral titers by 10- to 100-fold in HSV-1 and PRV [6]. gM homologs have also been identified in HCMV and EHV-1, which are major structural proteins in the virions [7,8]. Furthermore, gM proteins of PRV and HSV-1 prevent syncytium formation mediated by gB, gD, and gH/gL and are involved in viral invasion [9,10]. Thus, gM is a multifunctional protein involved in the herpesvirus life cycle.

Apoptosis, also known as programmed cell death, plays a crucial role in eliminating virus-infected cells and maintaining tissue homeostasis. The two main apoptotic signaling pathways are the extrinsic (death receptor) and intrinsic (mitochondria) pathways. Mitochondria play a central role in regulating intrinsic apoptosis [11]. The intrinsic pathway is triggered by intracellular stress signals, such as virus infection and DNA damage, resulting in conformational changes of pro-apoptotic effectors BAX and BAK. Then, BAX and BAK translocate, oligomerize, and form large pores on the mitochondrial membrane [12,13], altering mitochondrial outer membrane permeabilization and releasing cytochrome C (Cyto C) and apoptosis inducing factor (AIF). The apoptosome, a complex containing Cyto C, apoptotic protease activating factor-1, and pro-caspase-9, is assembled in the cytosol, and the released AIF is transferred into the nucleus to induce DNA fragmentation [14]. The activation of initiator caspases, including caspase-9, results in the activation of effector caspases, such as caspase-3/7, which selectively process their substrates, such as poly (ADP-ribose)-polymerase 1 (PARP1), leading to apoptosis [11].

Herpesviruses, including HSV-1, PRV, and VZV, have evolved numerous strategies to manipulate apoptosis [15]. For example, HSV-1 renders infected cells resistant to apoptosis induced by cytotoxic T lymphocytes *in vitro* to enhance viral replication [16]. Additionally, HSV-1-infected neurons are resistant to apoptosis and form latent infections in rabbit [17]. However, HSV-1-infected human immature dendritic cells undergo apoptosis [18]. These studies revealed the various mechanisms of apoptosis involved in HSV-1 infection. With ubiquitous tissue tropism, PRV can infect almost all organs and tissues, including the liver, spleen, lung, kidney, tonsil, and nervous system, resulting in extensive tissue necrosis [19]. PRV infection induces host cell apoptosis during the late stage of viral infection [20,21], and cell death is the main way that PRV passes through the basement membrane and releases viral particles [22]. The inhibition of apoptosis by phosphonoformate sodium decreases DNA copy numbers and gE, gG, and gD protein levels of PRV [23]. These studies suggest that PRV-induced apoptosis is correlated with viral replication. Moreover, PRV-infected pigs demonstrate altered expression levels and ratios of apoptosis-related proteins, such as BCL-2 and BAX, and PRV-infected immune cells, including lymphocytes and macrophages, exhibit morphological characteristics of apoptosis, such as cell shrinkage, nuclear fragmentation, and apoptotic body formation [24,25], suggesting that PRV-induced apoptosis may be closely related to viral pathogenicity. Thus, exploring the mechanism of PRV infection-induced apoptosis can provide a foundation for elucidating the pathogenic mechanisms and pathogenicity of PRV. However, the molecular mechanisms by which PRV induces apoptosis, and the role of apoptosis in PRV infection are still unclear.

The present study aimed to study the relationship between herpesvirus infection and apoptosis *in vitro* and *in vivo* using PRV as the model virus. Our results revealed that the gM proteins of PRV and HSV-1 are key virulence-related proteins involved in inducing mitochondria-mediated apoptosis, and that caspase-3-mediated apoptosis is closely linked to the replication and pathogenicity of PRV and HSV-1; these findings provide novel insights for the development of new attenuated live vaccine candidates and antiviral drugs.

## Results

### PRV gM protein induces apoptosis

To characterize the pathogenicity and cell death induced by PRV infection *in vivo*, three specific pathogen-free (SPF) piglets (6-week-old) were intranasally infected with 10^5^ TCID_50_ (50% tissue infective dose)/mL of PRV (1 mL/piglet), and two piglets treated with phosphate buffered saline (PBS) were used as controls. At 8 days post-infection (dpi), all piglets incubated with PRV died, and more copies of PRV genomic DNA were detected in the brain, lung, spleen, and tonsil than those in the PBS-treated group (S1A and S1B Fig). Furthermore, obvious cell death was observed after hematoxylin and eosin (H&E) staining and terminal deoxynucleotidyl transferase-mediated dUTP nick end labeling (TUNEL) staining in the tissues of PRV-treated group. PRV infection induced piamater necrosis and neuroglial cell death in the brain. Extensive inflammatory cell infiltration and congestion as well as bronchiolar epithelial cell degeneration and necrosis were observed in the lung. The tonsil presented lymphopenia, nuclear pyknosis, and focal necrosis (black arrowhead) (S1C Fig). Moreover, the tonsil and brain of PRV-infected piglets presented more TUNEL-labeled apoptotic cells (red) than those in PBS-treated piglets (S1D and S1E Fig). These results indicate that PRV infection induces severe apoptosis in piglets.

To further visualize PRV-induced apoptosis, the morphological changes in PRV-GFP-infected HeLa cells were observed using a living cell imaging system. PRV-GFP-infected cells gradually became rounder and smaller until they collapsed (black arrowhead) (S1 Video and S2A Fig). Infection of primary porcine alveolar macrophages (PAMs) and HeLa cells with PRV followed by TUNEL labelling revealed typical morphological characteristics of apoptotic cells, such as cell shrinkage, rounding, and DNA fragmentation (TUNEL-labeled, yellow) until cell death (red arrowhead) (S2B and S2C Fig). Additionally, PRV infection-induced extensive apoptosis in PAMs, MPK, IPEC-J2, and 3D4/21 cells was confirmed using flow cytometry after Annexin V-FITC/propidium iodide (PI) staining (S2D-S2G Fig). In summary, these observations clearly demonstrate that PRV infection induces significant apoptosis *in vitro* and *in vivo*.

**Fig 1:**
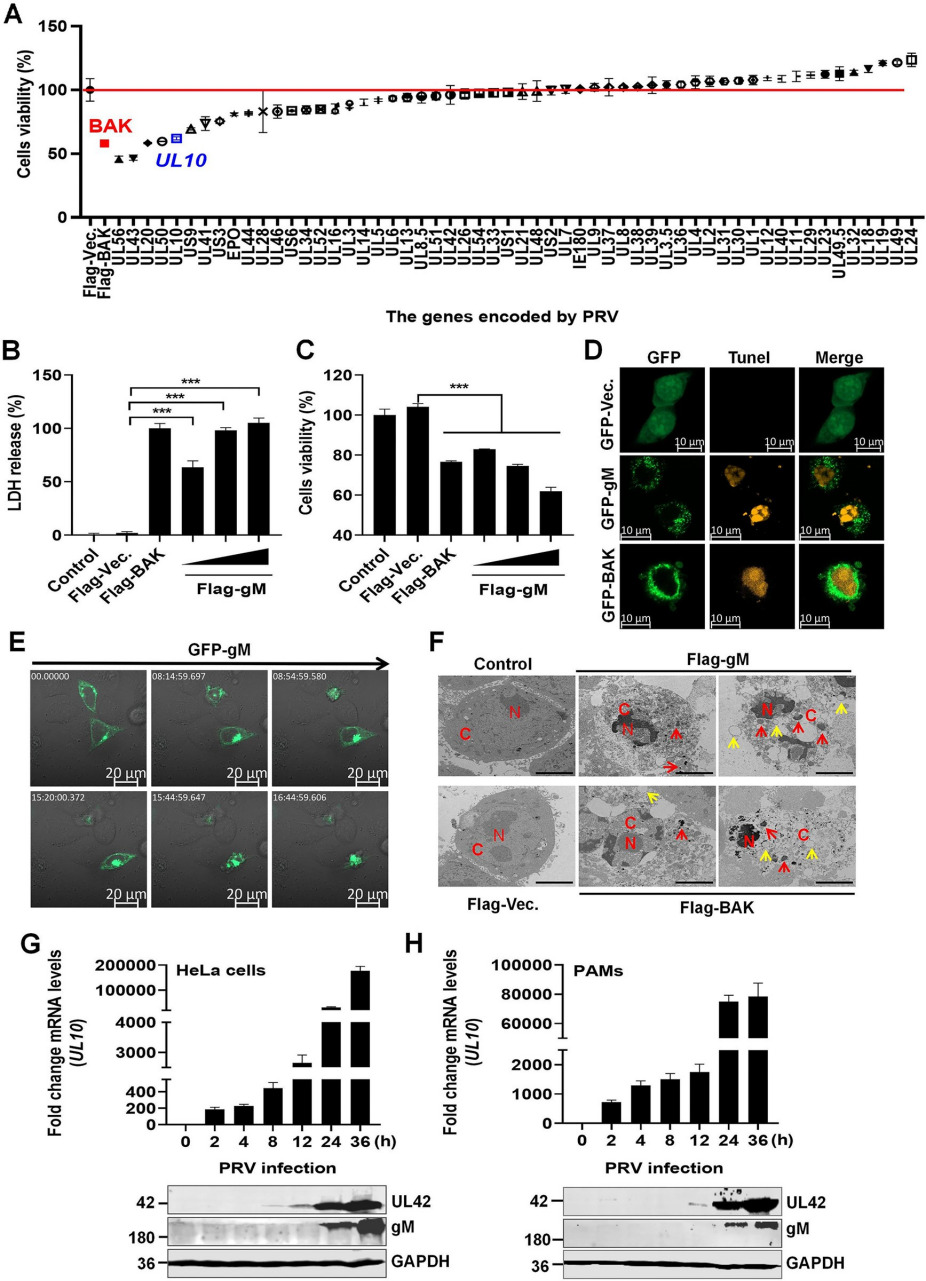
Pseudorabies virus (PRV) gM protein causes apoptosis. (**A**) Screening for PRV genes involved in cell death *in vitro*. Plasmids expressing Flag-Vec. and Flag-BAK were used as negative and positive controls, respectively. (**B-C**) PRV gM protein caused cell death in a dose-dependent manner. HEK293T cells transfected with plasmids expressing Flag-gM were analyzed for lactate dehydrogenase (LDH) levels in the supernatants (**B**) and cellular ATP enzymatic activity (**C**). The Flag-Vec. and Flag-BAK plasmids were used as negative and positive controls, respectively. (**D**) PRV gM protein induced DNA damage in the HeLa cells. Apoptotic cells were detected using TUNEL staining and analyzed using laser confocal microscopy. GFP-Vec. and GFP-BAK plasmids were used as the negative and positive controls, respectively. (**E**) Morphological images of HeLa cells overexpressing GFP-gM. HeLa cells transfected with a plasmid expressing GFP-gM were monitored for morphological changes under a 63× oil objective using a real-time confocal microscope. White number in top-left corner indicates relative time. **(F)** Observation of apoptosis in HeLa cells transfected with plasmids expressing Flag-gM using transmission electron microscopy. The Flag-Vec. and Flag-BAK plasmids were used as negative and positive controls, respectively. Red N, nucleus; red C, cytoplasm; red arrowhead, apoptotic body; yellow arrowhead, vacuolated endoplasmic reticulum. Scale bars, 4 μm. (**G-H**) Detection of gM transcription and expression levels during PRV infection. HeLa cells (**G**) or porcine alveolar macrophages (PAMs) (**H**) were infected with PRV at a multiplicity of infection (MOI) of 1 for 36 h. *UL10* mRNA and gM protein levels were detected using quantitative polymerase chain reaction (qPCR) and western blotting, respectively. Results shown are representative of three independent experiments (mean ± standard deviation [SD]) or of three independent experiments with similar results (one-way analysis of variance [ANOVA] in panels B-C). ***, *P* < 0.001.

Further, to identify the viral proteins involved in PRV infection-mediated apoptosis, 56 recombinant plasmids expressing PRV-encoded proteins were individually transfected into HEK293T cells, and cell viability was determined by evaluating cellular ATPase activity at 36 h post-transfection. Interestingly, similar to the pro-apoptotic protein BAK, *UL56*, *UL43*, *UL20*, *UL50*, and *UL10* gene-encoded proteins markedly decreased cell viability (Fig 1A). Under a microscopy, we observed that HEK293T cells overexpressing *UL10* gene-encoded PRV gM protein exhibited apoptotic cell morphology. Moreover, overexpression of the PRV gM protein decreased HEK293T cell viability dose-dependently, as determined by quantifying lactate dehydrogenase (LDH) levels in the supernatants and cellular ATPase activity (Fig 1B and 1C). Similar to the pro-apoptotic protein GFP-BAK, PRV gM-induced DNA fragmentation (TUNEL-labeled, yellow) was observed in HeLa cells (Fig 1D). To further validate our findings, we tracked the dynamic changes in HeLa cells after GFP-gM overexpression using a living cell imaging microscopy. The gM protein formed aggresomes, which were single, large, juxtanuclear structures, and HeLa cells gradually shrunk and became rounder and smaller until they collapsed (S2 Video and Fig 1E). Using transmission electron microscopy, condensed chromatin appeared as fragmented electron-dense masses (red C) and was marginalized to the periphery of the nucleus (red N) in the Flag-gM-overexpressing HeLa cells, similar to Flag-BAK. In addition, vacuolated endoplasmic reticulum (yellow arrowhead), apoptotic bodies (red arrowhead), and swollen mitochondria, commonly associated with apoptosis, were also observed in Flag-gM-overexpressing HeLa cells (Fig 1F). Thus, these results indicate that the PRV gM protein triggers apoptosis.

It has been reported that PRV gM is a late envelope glycoprotein [5]. We observed that PRV gM protein formed an aggresome, a large juxtanuclear structure that appears to impinge upon and distort the contour of the nuclear envelope. To better characterize the expression cycle of gM protein during PRV infection, we tracked *UL10* transcription and expression levels in HeLa cells and PAMs during PRV infection and detected gM protein at 24 h post infection (Fig 1G and 1H). Notably, the molecular weight of PRV gM protein was >180 kDa when the protein samples were boiled at 100°C for 10 min. A possible reason for this discrepancy is that PRV gM is a hydrophobic membrane protein, and heat treatment induces membrane proteins to aggregate and form polymers or undergo conformational changes. Interestingly, we overexpressed a plasmid expressing GFP-gM in human glioma cells (Ln229 cells) for 24 h and monitored cellular morphological changes for the next 72 h. We observed the cytoplasmic enrichment of gM protein in Ln229 cells, similar to that observed in HeLa and HEK293T cells. However, gM aggresomes did not affect Ln229 cell division, wherein the gM aggresomes gradually decomposed into small particles and disappeared, rather than leading to cell shrinkage and death (S3 Video), suggesting that PRV gM protein fair to induced apoptosis in the Ln229 cells.

### PRV gM protein facilitates mitochondria-mediated apoptosis

**Fig 2:**
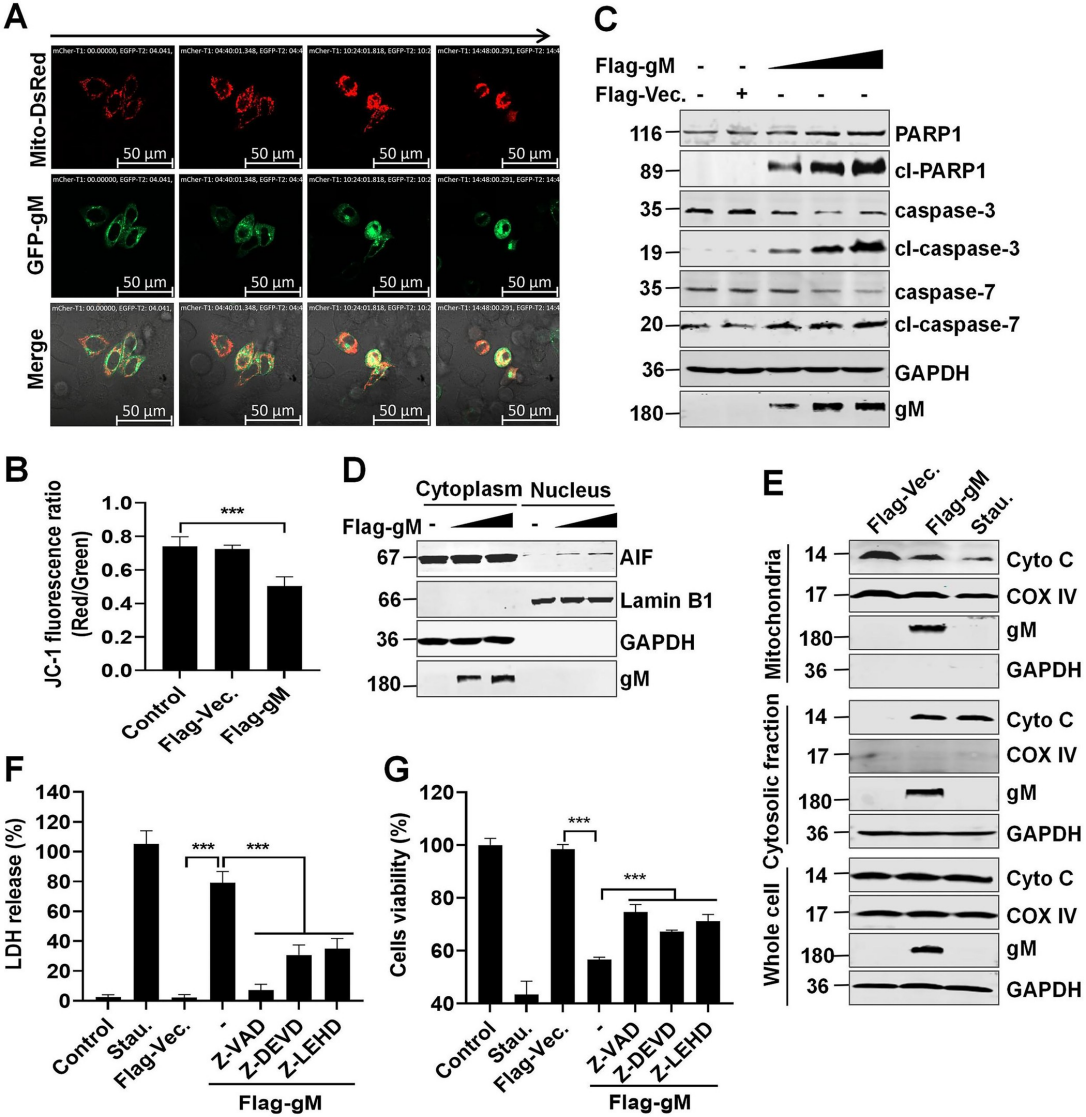
PRV gM protein triggers mitochondria damage. (**A**) Representative morphological images of HeLa cells co-expressing GFP-gM (green) and Mito-DsRed (red). White number in top-left corner indicates relative time. (**B**) Detection of the mitochondrial membrane potential. HeLa cells transfected with a plasmid expressing Flag-gM were harvested, stained with JC-1, and 10,000 cells were analyzed using flow cytometry. (**C**) PRV gM activated caspase-3 and caspase-7 to promote poly (ADP-ribose)-polymerase 1 (PARP1) cleavage. HeLa cells were transfected with the plasmid expressing Flag-gM for 36 h. Activation of caspase-3 and caspase-7 in the cell lysates was determined using western blotting. (**D**) Detection of the nuclear transport of apoptosis inducing factor (AIF). Cell lysates from HeLa cells transfected with the plasmid expressing Flag-gM were used for cytoplasmic and nuclear separation and were analyzed using western blotting with the indicated antibodies. (**E**) PRV gM induced Cyto C translocation. Cell lysates from HeLa cells transfected with the plasmid expressing Flag-gM were used for cytoplasmic and mitochondrial separation and analyzed using western blotting. Cells treated with Staurosporine (Stau.) were used as a positive control. COX IV and GAPDH were used as internal controls for mitochondrial and cytosolic fractions, respectively. (**F-G**) Detection of LDH levels and ATP enzyme activity. HeLa cells pre-treated with Z-VAD, Z-DEVD, or Z-LEHD were transfected with a plasmid expressing Flag-gM. LDH levels in cell supernatants (**F**) and cellular ATP enzymatic activity (**G**) were measured. The cells treated with Stau. were used as a positive control. Results shown are representative of three independent experiments (mean ± SD) or of three independent experiments with similar results (one-way ANOVA in panels B, F-G). ***, *P* < 0.001.

Mitochondria play an important role in activating apoptosis in mammalian cells. Considering the transmission electron microscopy findings of many swollen mitochondria in gM-overexpressing HeLa cells, we investigated whether mitochondria were involved in PRV gM-induced apoptosis. HeLa cells were transfected with plasmids expressing GFP-gM and Mito-DsRed, which was used to label the mitochondria. A real-time confocal assay was conducted to monitor the dynamic changes in the mitochondria using a living cell imaging microscopy. Initially, GFP-gM protein (green) was uniformly expressed in the cytoplasm, and mitochondria presented a normal rod shape and were divided into a network along the cytoplasm (S4 Video and Fig 2A). Notably, the co-localization of PRV gM and mitochondria was absent. Subsequently, with PRV gM overexpression, the mitochondria shrank, aggregated, and fragmented until cell death, while gM and mitochondria colocalized (S4 Video and Fig 2A). Meanwhile, the mitochondrial potential was significantly reduced in gM-overexpressing HeLa cells (Fig 2B). The central component of apoptosis is the proteolytic system, which involves a family of proteases called apoptotic caspases. These enzymes participate in a cascade by cleaving a set of proteins, such as PARP1, to trigger apoptosis, resulting in cell disassembly [26]. Western blotting of the lysates from HEK293T cells transfected with a Flag-gM-expressing plasmid revealed that PRV gM overexpression activated the apoptotic effectors caspase-3/7 and cleaved the apoptotic substrate PARP1 (Fig 2C), resulting in the nuclear transport of AIF (Fig 2D) and the release of Cyto C from the mitochondria (Fig 2E), thus inducing mitochondrial damage. To further validate our findings, HEK293T cells were pretreated with a general caspase inhibitor (Z-VAD), caspase-3/7 inhibitor (Z-DEVD), or caspase-9 inhibitor (Z-LEHD), and cells viability (cellular ATPase activity) was evaluated. Consequently, LDH levels in the supernatants were reduced and cells viability was partially rescued (Fig 2F and 2G). Collectively, these results illustrate that PRV gM induces mitochondria-mediated apoptosis.

**Fig 3:**
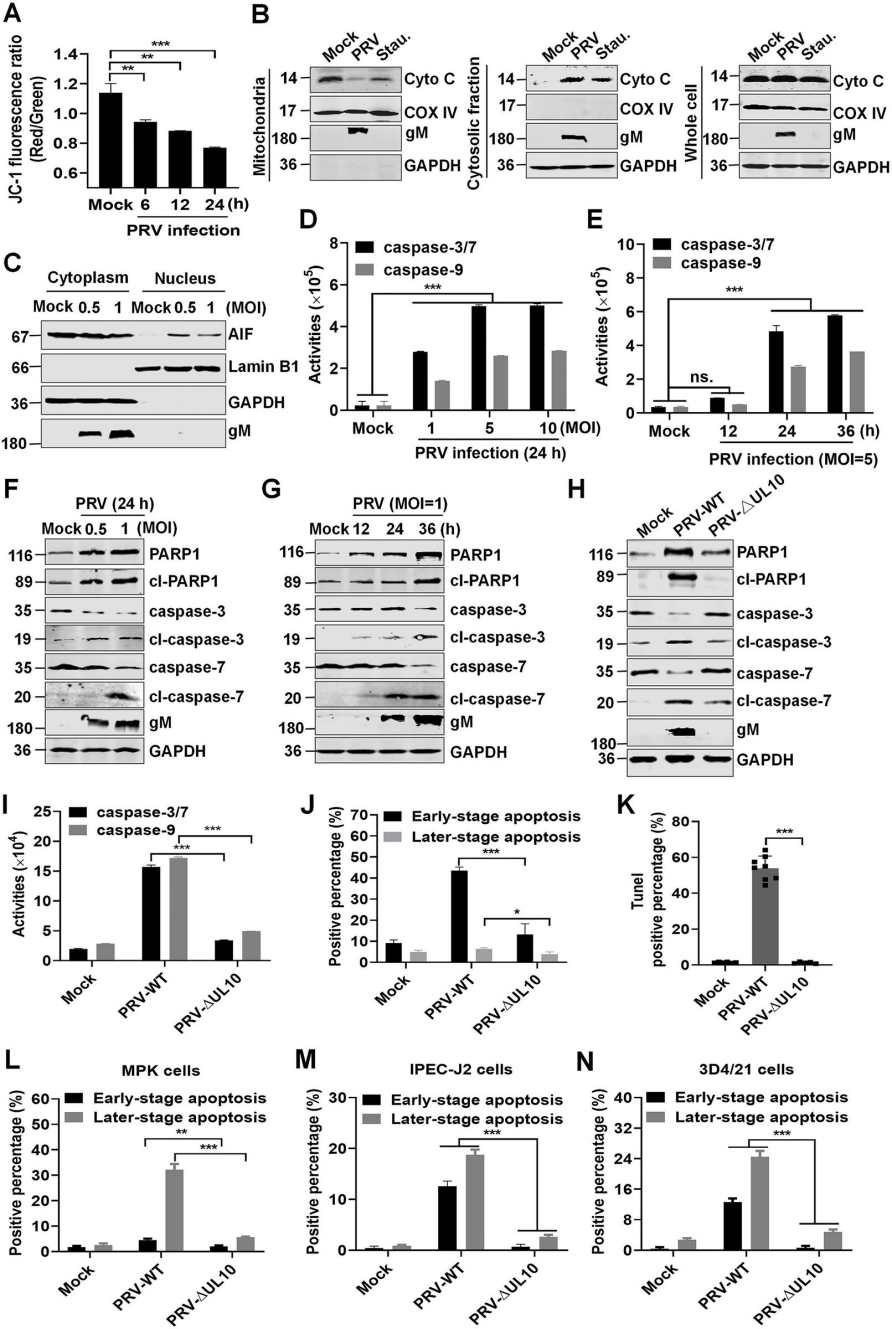
PRV infection-mediated mitochondria-related apoptosis is dependent on the gM protein. (**A**) Detection of the mitochondrial membrane potential. HeLa cells infected with PRV at an MOI of 1 were harvested and stained with JC-1, and 10,000 cells were analyzed using flow cytometry. (**B**) PRV infection induced Cyto C translocation. Lysates of HeLa cells infected with PRV at an MOI of 1 were used for cytoplasmic and mitochondrial separation and analyzed using western blotting with the indicated antibodies. The cells treated with Stau. were used as the positive control. COX IV and GAPDH were used as internal controls for the mitochondrial and cytosolic fractions, respectively. (**C**) Detection of the nuclear transportation of AIF. Following the method described in (**B**), HeLa cell lysates were subjected to cytoplasmic and nuclear separation. The resulting cytoplasmic and nuclear fractions were analyzed using western blotting. (**D-E**) Detection of activated caspase-3/7 and caspase-9 in PRV-infected PAMs. **(F-G)** Activation of caspase-3/7 in PRV-infected HeLa cells. HeLa cells were mock-infected or infected with PRV at an MOI of 0.5 or 1 for 24 h or at an MOI of 1 for 12, 24, or 36 h. Cell lysates were harvested to detect the indicated proteins using western blotting. (**H**) PRV-ΔUL10 failed to activate caspase-3/7. HeLa cells were infected with PRV-WT or PRV-ΔUL10 at an MOI of 1 for 24 h. Cell lysates were harvested for western blotting analysis. (**I-K**) PRV-ΔUL10 could not induce apoptosis in PAMs. PAMs were infected with PRV-WT or PRV-ΔUL10 at an MOI of 5 for 36 h, and then cellular activities of caspase-3/7/9 were detected (**I**). Or the cells were stained with propidium iodide (PI)/Annexin V and the percentage of labeled cells was quantified using flow cytometry (**J**). Or cells were stained with TUNEL and visualized under a laser confocal microscope (**K**). (**L-N**) PRV-ΔUL10 failed to induce apoptosis in various cell lines. MPK (**L**), IPEC-J2 (**M**), and 3D4/21 (**N**) cells were infected with PRV-WT or PRV-ΔUL10 at an MOI of 1 for 24 h. The cells were stained with PI/Annexin V and the percentage of labeled cells were quantified using flow cytometry. Both PI and Annexin V double-stained cells showed later-stage apoptosis, and only Annexin V-labeled cells showed early-stage apoptosis. Results shown are representative of three independent experiments (mean ± SD) or of three independent experiments with similar results (one-way ANOVA in panel A, K; two-way ANOVA in panels D-E, I-J, L-N). *, *P* < 0.05; **, 0.001 < *P* < 0.01; ***, *P* < 0.001.

Consistently, we observed reduced mitochondrial potential, released Cyto C, and nuclear transport of AIF in PRV-infected HeLa cells (Fig 3A-3C). Furthermore, PRV infection also induced the activation of caspase-3/7/9 in PAMs (Fig 3D and 3E). In agreement with these results, western blotting showed that PRV infection also activated caspase-3/7, resulting in PARP1 cleavage in HeLa cells (Fig 3F and 3G). To further investigate whether gM is required for PRV-induced apoptosis, a recombinant mutant PRV strain lacking 146 bp of the *UL10* gene (PRV-ΔUL10) was generated from the highly pathogenic PRV-TJ strain. As shown in S3A-S3D Fig and S1 Table, the 146 bp deletion completely disrupted the PRV *UL10* gene (S1 Appendix), leading to complete silencing of the PRV gM protein in PRV-ΔUL10-infected Vero cells. And PRV-ΔUL10 was further verified through whole genome sequencing (S2 Appendix). After comparison, we found that there was only 146 bp deletion inside of *UL10* gene and no second site mutation in PRV genomic DNA (81357 bp-81502 bp, S3 Appendix). In addition, the replication efficiency of PRV-ΔUL10 in Vero cells was significantly lower than that of its parental virus (S3E Fig). Moreover, PRV-ΔUL10 infection could not activate caspase-3/7 and cleave PARP1 in HeLa cells (Fig 3H). Consistently, compared with wild-type PRV (PRV-WT), PRV-ΔUL10 infection failed to activate caspase-3/7/9 (Fig 3I), remarkably decreasing apoptosis (Fig 3J) and DNA fragmentation (TUNEL-labeled) (Fig 3K) in PAMs. To further verify our results, other porcine cell lines, including MPK, IPEC-J2, and 3D4/21 were infected with PRV-WT or PRV-ΔUL10, and the apoptotic cells were analyzed using flow cytometry. As shown in Fig 3L-3N, PRV-ΔUL10 infection failed to induce apoptosis in these cells, compared with PRV-WT. Thus, PRV infection induces mitochondria-dependent apoptosis by activating the apoptotic effector caspase-3/7, which is mainly dependent on the gM protein.

### PRV gM targets BCL-XL

**Fig 4:**
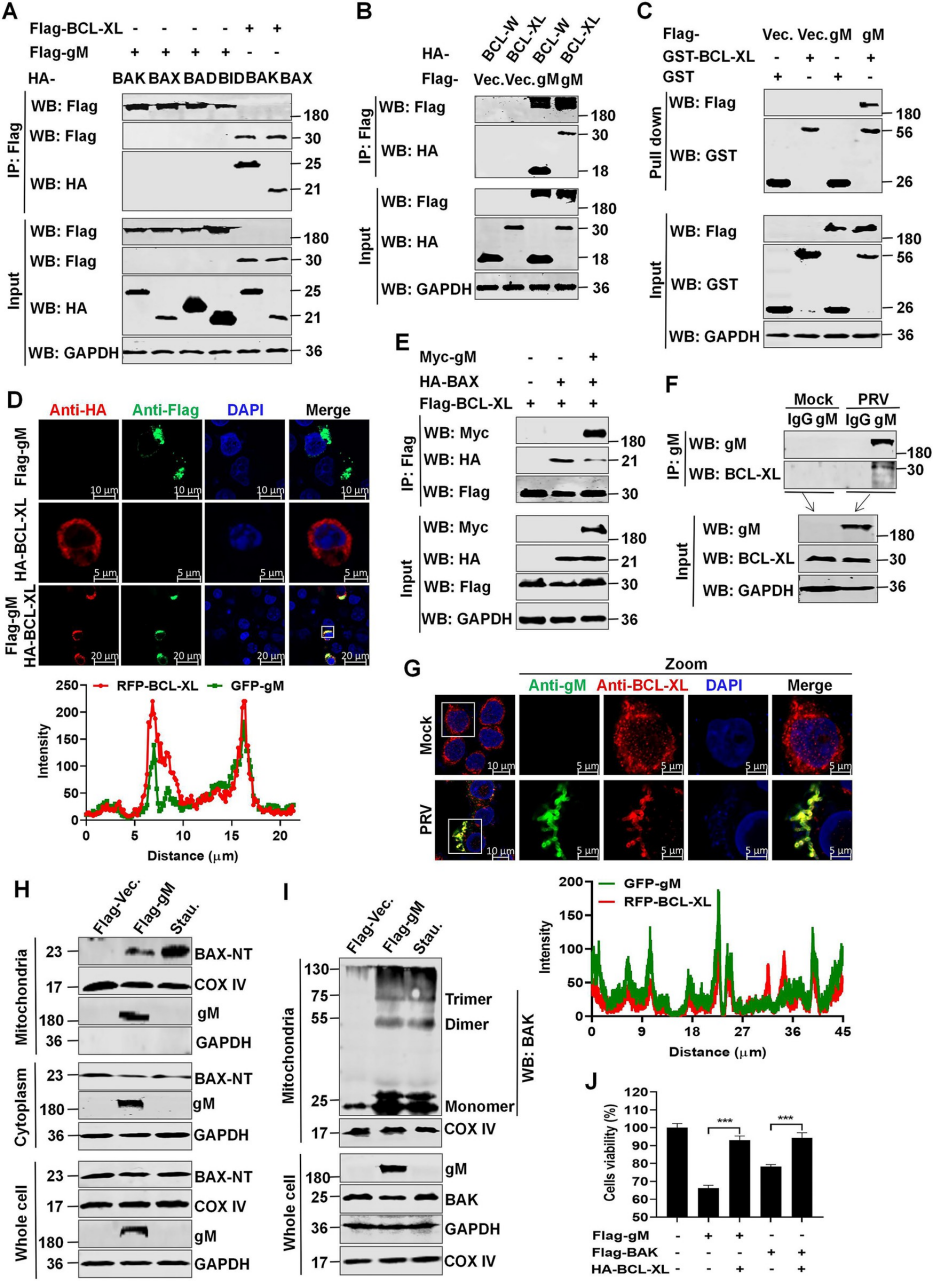
PRV gM interacts with BCL-XL to activate BAX and BAK. (**A-B**) Interactions between pro-apoptotic or anti-apoptotic proteins and Flag-gM. HEK293T cells were transfected and the lysates were subjected to co-immunoprecipitation (Co-IP) analysis. (**C**) The direct interaction between Flag-gM and BCL-XL was detected using a GST-pulldown. (**D**) Colocalization of Flag-gM (green) and HA-BCL-XL (red) was observed using laser confocal microscopy. The colocalization between gM and BCL-XL was analyzed using the ZEISS software. (**E**) PRV gM competed with BAX to bind to BCL-XL in HEK293T cells. (**F-G**) PRV gM interacted and colocalized with endogenous BCL-XL in PRV-infected HeLa cells. HeLa cells were mock-infected or infected with PRV at an MOI of 1 for 24 h and cell lysates were subjected to Co-IP analysis using an anti-gM antibody (**F**). Or the cells were incubated with anti-gM (green) and anti-BCL-XL (red) antibodies and observed using laser confocal microscopy. The colocalization between gM and BCL-XL was analyzed using the ZEISS software (**G**). (**H-I**) PRV gM activated both BAX and BAK. The mitochondrial and cytosolic fractions of Flag-gM-transfected HEK293T cells were isolated to detect BAX activation using western blotting (**H**). In addition, the isolated mitochondrial fraction was cross-linked with Bismaleimidohexane (BMH) to analyze BAK homo-oligomerization (**I**). The cells treated with Stau. were used as the positive control. COX IV and GAPDH were used as internal controls for the mitochondrial and cytosolic fractions, respectively. (**J**) BCL-XL relieved gM-induced apoptosis. Transfected HEK293T cells were used to analyze ATP enzymatic activities. Results shown are representative of three independent experiments (mean ± SD) or of three independent experiments with similar results (one-way ANOVA in panel J). ***, *P* < 0.001.

Mitochondrial outer membrane permeability and mitochondria-dependent apoptosis are regulated by members of the BCL-2 family [27]; they are subdivided into the following three subgroups based on their structure and function: anti-apoptotic members (such as BCL-2, BCL-XL, BCL-W, and MCL-1), pro-apoptotic effectors (BAK and BAX), and pro-apoptotic BH3-only members (such as NOXA, BIM, PUMA, and BID) [28,29]. Co-immunoprecipitation (Co-IP) was performed to determine whether members of the BCL-2 family are involved in gM-induced apoptosis. HEK293T cells were transfected with a plasmid expressing Flag-gM and a plasmid expressing different members of the three subgroups. The results revealed that PRV gM interacted with the anti-apoptotic members BCL-XL and BCL-W, but not with the pro-apoptotic members BAK, BAX, BAD, and BID (Fig 4A and 4B), which was further confirmed using the GST-pulldown assay and laser confocal microscopy (Fig 4C and 4D). In addition, Myc-PRV gM overexpression blocked the interaction between BCL-XL and BAX (Fig 4E). Furthermore, Co-IP and laser confocal microscopy revealed an association between endogenous BCL-XL and the physiological levels of PRV gM in PRV-infected HeLa cells (Fig 4F and 4G). BAX activation and BAK homo-oligomerization essentially initiate mitochondria-mediated apoptosis [29]. Hence, HEK293T cells were treated with the apoptosis inducer, staurosporine (Stau.), or were transfected with a plasmid expressing Flag-gM. BAX-NT levels and BAK homo-oligomerization were observed in the mitochondria of Flag-gM-overexpressed HEK293T cells (Fig 4H and 4I), suggesting the induction of apoptosis, similar to Stau.-treated cells. Notably, transfection with a plasmid expressing HA-BCL-XL significantly reversed PRV gM-induced apoptosis in HEK293T cells (Fig 4J). Together, PRV gM targets the anti-apoptotic protein BCL-XL to activate BAK and BAX, thereby enhancing mitochondria-dependent apoptosis.

To identify the gM domain responsible for PRV gM-induced apoptosis, the transmembrane domains of gM were analyzed using the TMHMM website and a series of plasmids expressing progressively truncated gM mutants were constructed (S4A Fig). HEK293T cells were individually transfected with these plasmids and visualized using laser confocal microscopy. Like intact PRV gM (gM_1-393_), gM_1-204_, gM_1-268_, gM_106-393_, and gM_35-393_ also formed aggresomes, whereas gM_1-118_, gM_1-155_, gM_292-393_, gM_228-393_, and gM_178-393_ did not, suggesting that the fourth transmembrane domain (aa156–177) was primarily responsible for gM aggresomes formation. Consistently, deletion of the fourth transmembrane domain (Flag-gM_Δ156–177_) caused diffuse cytoplasmic distribution of gM (S4B Fig) and partially reversed the reduced cells viability (ATPase activity) induced by PRV gM in the HEK293T cells (S4C Fig). Flow cytometry analysis also showed that transfection of Flag-gM_Δ156–177_ in the HEK293T cells induced a weaker apoptosis than that of Flag-gM (S4D Fig). Together, these results illustrate that the transmembrane regions, particularly the fourth transmembrane region (aa156-177) of gM, are required for its subcellular localization and apoptosis.

### PRV gM homologs in other herpesviruses induce apoptosis

**Fig 5:**
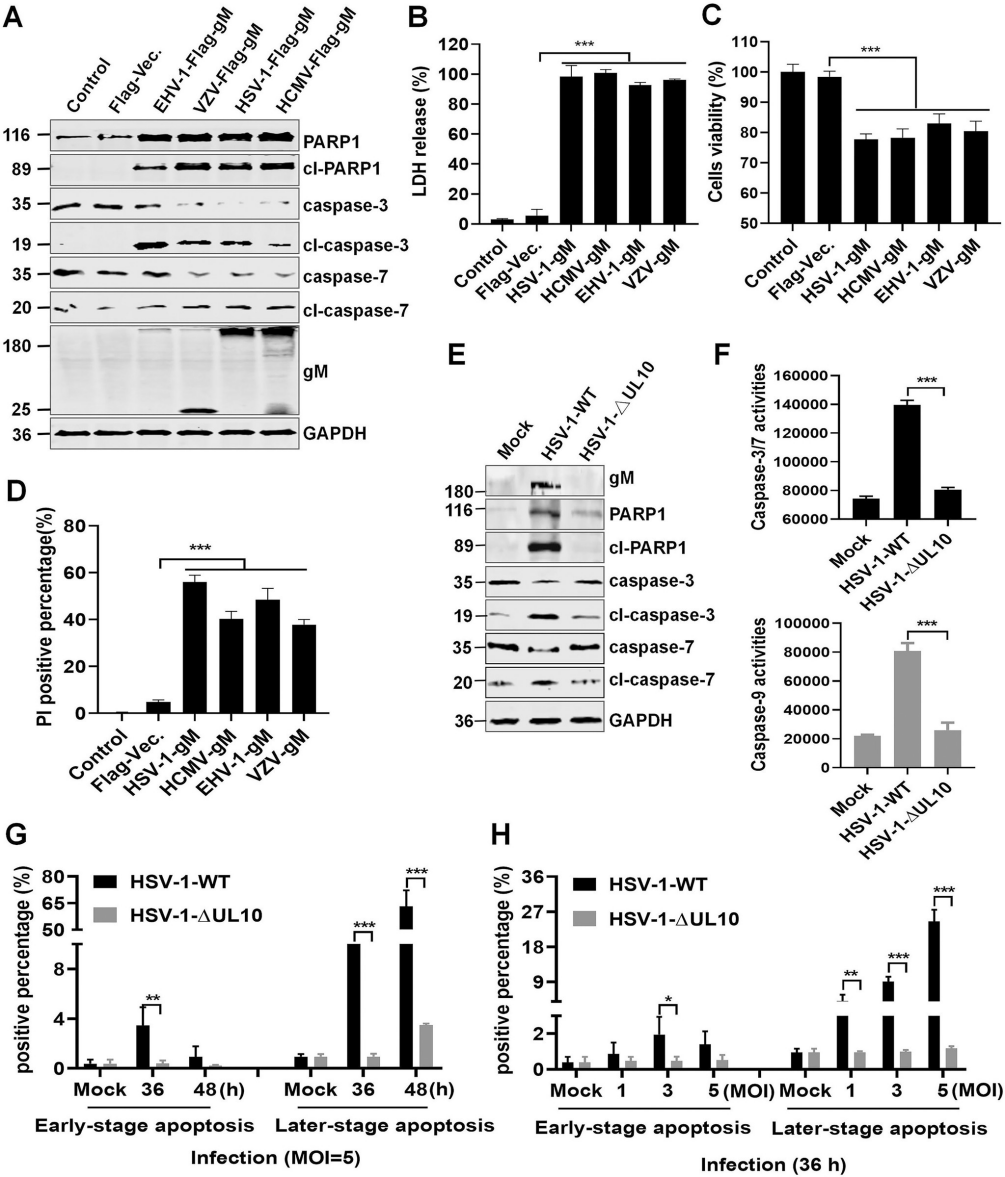
PRV gM and its homologous proteins in herpesvirus also activate caspase-3/7 and induce apoptosis. (**A**-**D**) PRV gM homologs in herpesvirus induced apoptosis. HEK293T cells transfected with plasmids expressing PRV gM homologs in HSV-1, HCMV, EHV-1, or VZV were collected, and protein levels were detected using western blotting (**A**). LDH released in the supernatants (**B**) and cellular ATP enzymatic activity (**C**) were also detected. Or the cells were stained with PI and subsequently analyzed using fluorescence microscopy (**D**). (**E-F**) HSV-1-mediated activation of caspase-3/7 was dependent on gM. HeLa cells lysates infected with HSV-1-WT or HSV-1-ΔUL10 at an MOI of 1 were harvested for western blotting analysis (**E**). THP-1 cells infected with HSV-1-WT or HSV-1-ΔUL10 at an MOI of 5 were collected to analyze caspase-3/7 and caspase-9 activity (**F**). (**G-H**) Detection of apoptotic cells. THP-1 cells were infected with HSV-1-WT or HSV-1-ΔUL10 as indicated, and stained with PI/Annexin V for flow cytometry analysis. The percentage of PI- and Annexin V-labeled cells was quantified. Results shown are representative of three independent experiments (mean ± SD) or of three independent experiments with similar results (one-way ANOVA in panels B-D, F; two-way ANOVA in panels G-H). *, *P* < 0.05; **, 0.001 < *P* < 0.01; ***, *P* < 0.001.

PRV gM and its homologs in herpesviruses share amino acid sequence similarities between 17.5–37.6% (S5A Fig). Next, we determined if these homologs were functionally conserved to induce apoptosis. A series of plasmids, including Flag-HSV-1-UL10, Flag-EHV-1-ORF52, Flag-VZV-ORF50, and Flag-HCMV-UL100, expressing PRV gM homologs in other herpesviruses, were constructed and individually transfected into HEK293T cells. Similar to the PRV gM protein, PRV gM homologs in other herpesviruses also formed aggresomes with large juxtanuclear structures (S5B Fig). Furthermore, the overexpression of these PRV gM homologs activated caspase-3/7 and induced PARP1 cleavage (Fig 5A), leading to increased LDH in the supernatants (Fig 5B), decreased cells viability (cellular ATPase activity) (Fig 5C), and increased apoptotic cells (PI staining) (Fig 5D) in HEK293T cells.

Then, HSV-1 was selected as a model virus to further verify whether these PRV gM homologs were required for herpesvirus-induced apoptosis at the physiological level. Thus, a recombinant HSV-1-ΔUL10 mutant was generated from the HSV-1-WT strain using CRISPR-Cas9; it contained an extra nucleotide insertion after 127 bp within the *UL10* gene, which caused frame shift of the *UL10* coding sequence and total disruption of the *UL10* gene (S6A and S6B Fig and S1 Table and S4 Appendix). And HSV-1-ΔUL10 was further verified through whole genome sequencing (S5 Appendix). After comparison, we found that there was only an extra nucleotide insertion inside of *UL10* gene and no second site mutation in HSV-1 genomic DNA (between 23255 bp and 23256 bp, S6 Appendix). Compared with HSV-1-WT strain, the replication kinetics of the HSV-1-ΔUL10 mutant was obviously reduced in Vero cells (S6C Fig). Additionally, HSV-1 infection also activated caspase-3/7 and cleaved PARP1 in HeLa cells (Fig 5E), as well as induced caspase-3/7/9 activation in THP-1 cells (Fig 5F), while HSV-1-ΔUL10 infection could not. Consistently, flow cytometry analysis showed that HSV-1 infection led to extensive apoptosis (Annexin V-FITC/PI-labeled) in THP-1 cells in a time- and dose-dependent manner, while HSV-1-ΔUL10 infection could not induce a similar effect (Fig 5G and 5H), suggesting that gM protein is required for HSV-1 infection-induced apoptosis. In general, PRV gM homologs in other herpesviruses are functionally conserved and induce apoptosis by activating the effector caspase-3/7.

### *UL10* is a key virulence gene of PRV and HSV-1

**Fig 6:**
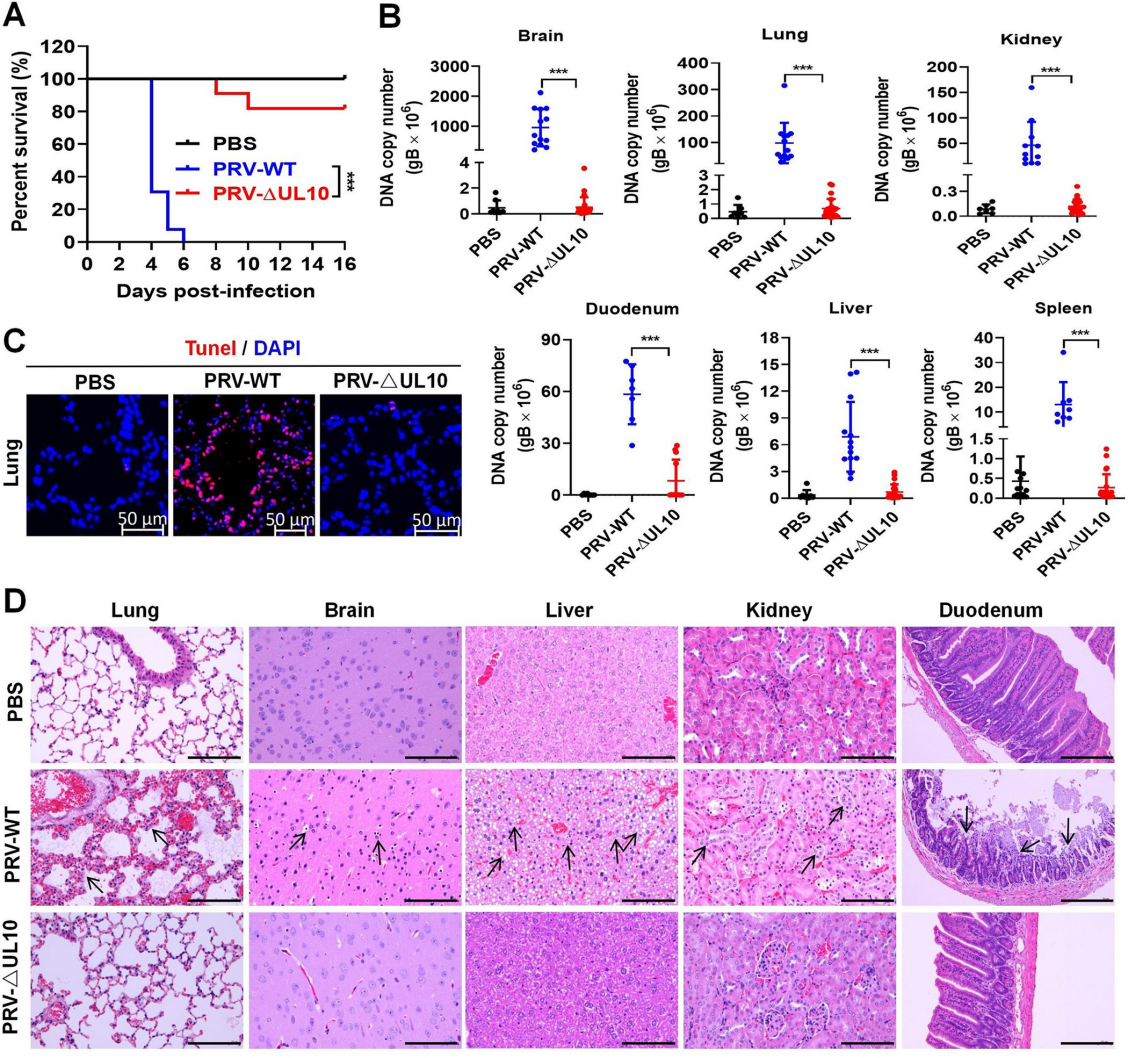
*UL10* gene deletion reduces PRV infection-induced apoptosis and viral pathogenicity *in vivo*. (**A**) Survival rate. Mice were challenged intraperitoneally with PRV-WT or PRV-ΔUL10 at a dosage of 2,000 PFU per mouse (n = 4 in PBS group, n = 12 in PRV-WT challenge group, n = 12 in PRV-ΔUL10 challenge group) and their survival was recorded for 15 days. Statistical significance was determined using the log-rank test. (**B**) qPCR analysis of PRV genomic DNA copies in tissues from mice challenged with PRV-WT or PRV-ΔUL10. (**C**) Pathological lesions in the mouse lungs (TUNEL staining). (**D**) Pathological lesions in the tissues from mice challenged with PRV-WT or PRV-ΔUL10 (hematoxylin and eosin (H&E) staining). Scale bars, 100 μm for the lung, brain, liver, and kidney; 200 μm for the duodenum. Results shown are representative of three independent experiments (mean ± SD) or of three independent experiments with similar results (one-way ANOVA in panel B). ***, *P* < 0.001.

The role of PRV gM in the pathogenicity of PRV was further confirmed *in vivo* by challenging SPF C57BL6J mice with PRV-WT or PRV-ΔUL10. As shown in Fig 6A, all mice (n = 12) challenged with PRV-WT died within 6 dpi, while only 2 mice died (n = 12) in the group challenged with PRV-ΔUL10 during the 15 dpi observation period. Consistently, the PRV genomic DNA copy numbers in the brain, lung, kidney, duodenum, liver, and spleen in mice challenged with PRV-WT were considerably higher than those in the PRV-ΔUL10-challenged group (Fig 6B). We observed robust cell apoptosis (TUNEL-labeled) in the PRV-WT-infected mouse lung, but not in the PRV-ΔUL10-infected mouse lung (Fig 6C). Compared with the control group, the PRV-WT infection group demonstrated severe tissue damage, including edema and blood vessel congestion, widening of the lung alveolar diaphragm, significant proliferation of glial cells and extensive degeneration of neuronal cells as well as the neurophilic phenomena in the brain, extensive vacuolar degeneration of liver cells, broad nuclear pyknosis and degenerating renal tubular epithelial cells, and considerable necrosis and shedding of duodenal villi. In contrast, these pathological lesions were negligible in the PRV-ΔUL10 infection group (Fig 6D). Overall, we conclude that the PRV *UL10* gene is a virulence-related gene and that *UL10*-encoded gM is a determining factor in PRV pathogenicity.

**Fig 7:**
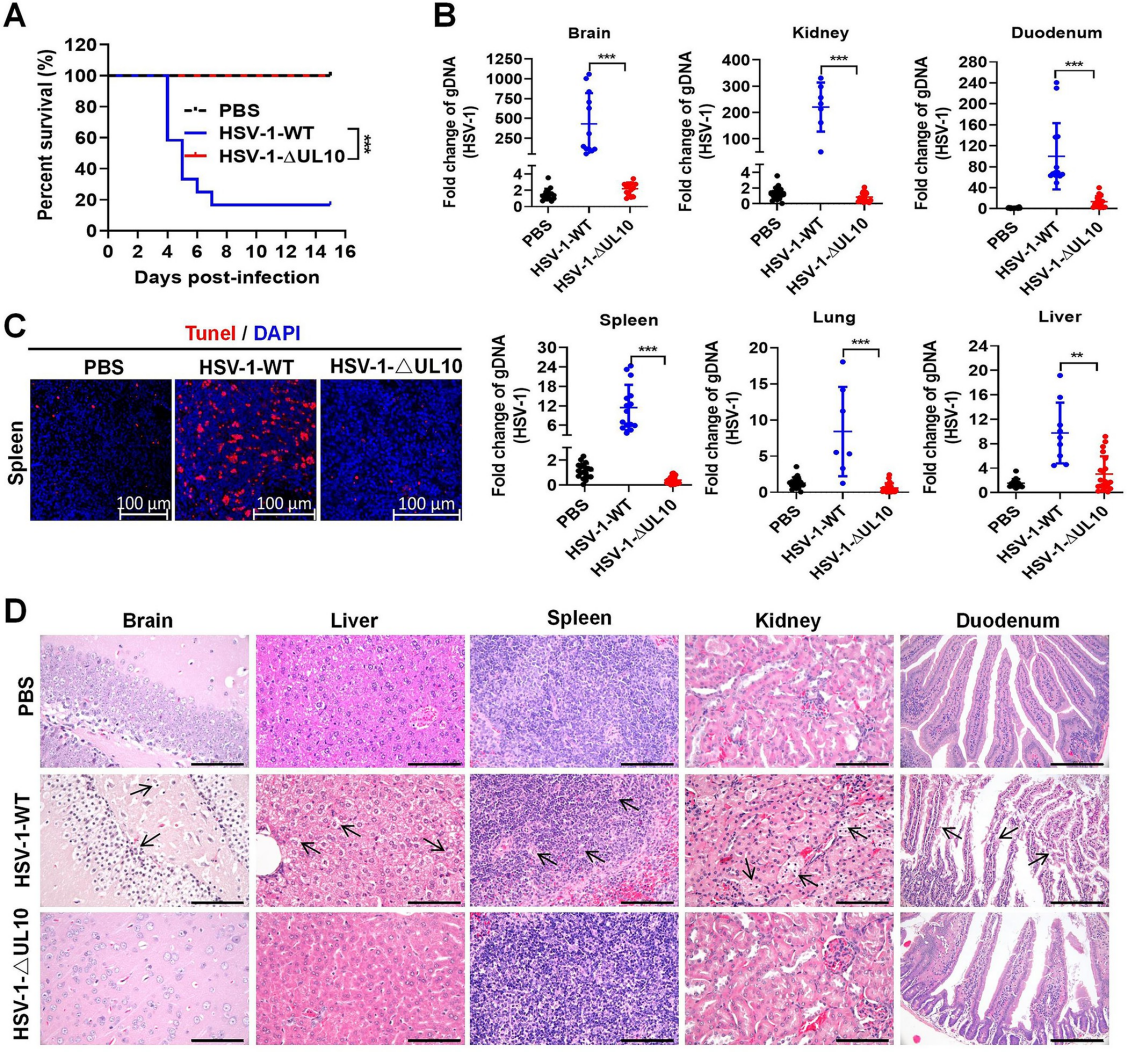
*UL10* gene determines the replication and pathogenicity of HSV-1 *in vivo*. (**A**) Survival rate. Mice were challenged intraperitoneally with HSV-1-WT or HSV-1-ΔUL10 at a dosage of 1×10^7^ PFU per mouse (n = 4 in PBS group, n = 12 in HSV-1-WT challenge group, n = 12 in HSV-1-ΔUL10 challenge group) and their survival was recorded for 15 days. Statistical significance was determined using the log-rank test. (**B**) qPCR analysis of the HSV-1 genomic DNA in tissues from mice challenged with HSV-1-WT or HSV-1-ΔUL10. (**C**) Pathological lesions in mouse spleens (TUNEL staining). (**D**) Pathological lesions in mouse tissues (H&E staining). Scale bars, 100 μm for the brain, liver, spleen, and kidney; 200 μm for the duodenum. Results shown are representative of three independent experiments (mean ± SD) or of three independent experiments with similar results (one-way ANOVA in panel B). **, 0.001 < *P* < 0.01; ***, *P* < 0.001.

In addition, 83% (10/12) of the HSV-1-WT-infected mice died within 15 days of observation, while no mice (0/12) died in the HSV-1-ΔUL10 infection group (Fig 7A). The relative levels of HSV-1 genomic DNA (gDNA) in the tissues from brain, lung, kidney, duodenum, liver, and spleen of mice challenged with the HSV-1-WT were also higher than those in mice challenged with HSV-1-ΔUL10 (Fig 7B). Consistently, robust cell apoptosis (TUNEL-labeled) was observed in the HSV-1-WT-infected mouse spleen tissue, which was significantly reduced in the HSV-1-ΔUL10-infected mouse spleen (Fig 7C). Compared with the control group, the HSV-1-WT-infected mice showed noticeable tissue damages, including extensive neurodegeneration and vacuolization in the hippocampus area of the brain, hepatocellular degeneration, lymphocyte enrichment in the white pulp of the spleen undergo necrosis and apoptosis, and extensive degeneration and necrosis in renal tubular epithelial cells and in small intestinal mucosal epithelial cells. In contrast, these pathological findings were negligible in HSV-1-ΔUL10-infected mice (Fig 7D). In summary, these results suggest that *UL10* is a key virulence gene of HSV-1.

### Deletion of caspase-3 attenuates the replication and pathogenicity of PRV and HSV-1

**Fig 8:**
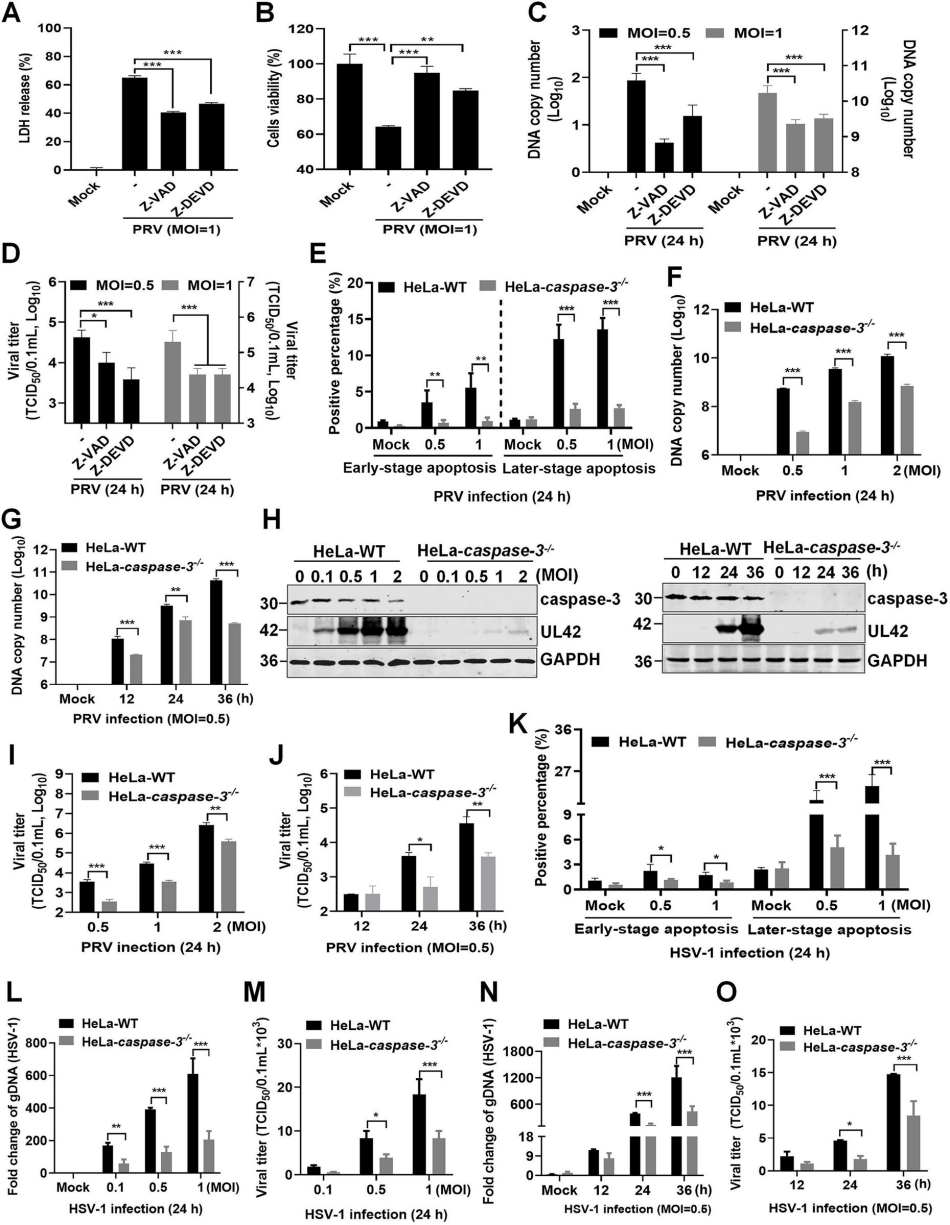
Deletion of caspase-3 impairs PRV and HSV-1 replication *in vitro*. (**A-B**) Detection of cells viability. HeLa cells pretreated with Z-VAD or Z-DEVD, followed by PRV infection at an MOI of 1 for 24 h, were analyzed for the LDH released in the supernatants (**A**) and cellular ATP enzymatic activity (**B**). (**C-D**) Caspase-3 inhibitors reduced PRV replication. HeLa cells were pretreated with Z-VAD or Z-DEVD, followed by PRV infection at MOI of 0.5, 1 for 24 h. The cell pellets were used for the PRV DNA copy number assay, and the cell supernatants were collected for the TCID_50_ assay. (**E**) Deletion of caspase-3 eliminated PRV infection-mediated apoptosis. HeLa-WT and HeLa-*caspase-3*^*-/-*^ cells infected with PRV were stained with PI/Annexin V for flow cytometry. The percentage of PI- and Annexin V-labeled cells was quantified. (**F-J**) Deletion of caspase-3 gene impaired PRV replication. The pellets of PRV-infected HeLa-WT and HeLa-*caspase-3*^*-/-*^ cells were used for detecting PRV DNA copy numbers using qPCR (**F-G**) or PRV-encoded UL42 protein levels using western blotting (**H**). The cell supernatants were collected for the TCID_50_ assay in Vero cells (**I-J**). (**K**) Deletion of caspase-3 eliminated HSV-1 infection-mediated apoptosis. HeLa-WT and HeLa-*caspase-3*^*-/-*^ cells infected with HSV-1 were stained with PI/Annexin V for flow cytometry. The percentage of PI- and Annexin V-labeled cells was quantified. (**L-O**) Deletion of caspase-3 diminished HSV-1 replication. The pellets of HSV-1-infected HeLa-WT and HeLa-*caspase-3*^*-/-*^ cells were used for HSV-1 genomic DNA detection using qPCR, and the cell supernatants were collected for TCID_50_ assay in Vero cells. Results shown are representative of three independent experiments (mean ± SD) or of three independent experiments with similar results (one-way ANOVA in panels A-D; two-way ANOVA in panels E-G, I-O). *, *P* < 0.05; **, 0.001 < *P* < 0.01; ***, *P* < 0.001.

To investigate whether gM protein-induced apoptosis was linked to PRV replication, HeLa cells were pretreated with Z-VAD or Z-DEVD to block mitochondria-mediated apoptosis, and then infected with PRV at an MOI of 1 for 24 h. We found that treatment with Z-VAD or Z-DEVD reduced LDH levels in the supernatants and partially rescued cells viability (ATPase activity) in HeLa cells (Fig 8A and 8B). Furthermore, treatment with these apoptosis inhibitors reduced the DNA copy numbers in the cells and viral titers in the supernatants of PRV (Fig 8C and 8D). Flow cytometry analysis of PRV-infected HeLa-WT and HeLa-*caspase-3*^*-/-*^ cells revealed that PRV infection caused low levels of apoptosis in HeLa-*caspase-3*^*-/-*^ cells than those in HeLa-WT cells (Fig 8E). Consistently, deletion of caspase-3 in HeLa cells significantly impaired the DNA copy numbers of PRV (Fig 8F and 8G) and reduced the expression of the PRV-encoded protein UL42 (Fig 8H), as well as viral titers in the supernatants (Fig 8I and 8J). Similar results were obtained for HSV-1-infected HeLa-*caspase-3*^*-/-*^ cells, including low levels of apoptotic cells (Fig 8K), decreased genomic DNA levels and viral titers of HSV-1 (Fig 8L-8O). Thus, PRV and HSV-1 infections activate caspase-3 to induce apoptosis and promote their replication *in vitro*.

**Fig 9:**
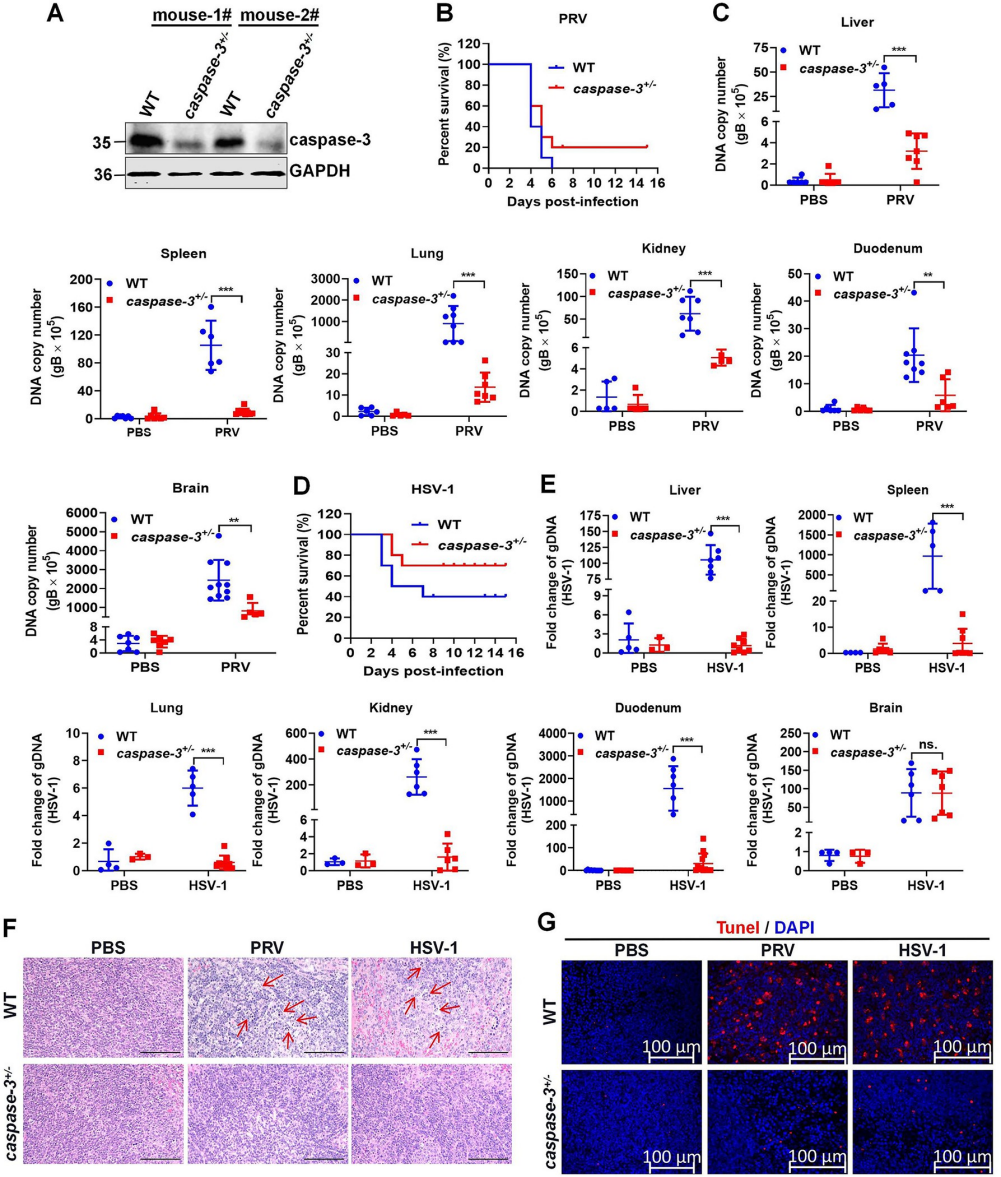
Deletion of caspase-3 reduced the replication and pathogenicity of PRV and HSV-1 in mice. (**A**) Detection of caspase-3 protein levels. Mouse peritoneal macrophages were isolated from WT and *caspase-3*^*+/-*^ mice, and the expression levels of caspase-3 were detected using western blotting. (**B**) Survival rate. WT and *caspase-3*^*+/-*^ mice were challenged intraperitoneally with PBS or PRV at a dosage of 2,000 PFU per mouse (n = 4 in the PBS group, n = 10 in the PRV-injected WT mice group, and n = 10 in the PRV-injected *caspase-3*^*+/-*^ mice group) and their survival was recorded for 15 days. (**C**) qPCR analysis of PRV genomic DNA copy number in tissues from WT and *caspase-3*^*+/-*^ mice challenged with PRV. (**D**) Survival rate. WT and *caspase-3*^*+/-*^ mice were challenged intraperitoneally with PBS or HSV-1 at a dosage of 0.5×10^7^ PFU per mouse (n = 4 in the PBS group, n = 10 in the HSV-1-injected WT mice group, n = 10 in the HSV-1-injected *caspase-3*^*+/-*^ mice group) and their survival was recorded for 15 days. (**E**) qPCR analysis of HSV-1 genomic DNA in tissues from WT and *caspase-3*^*+/-*^ mice challenged with HSV-1. (**F-G**) Pathological lesions in the spleen of mice using H&E staining (**F**) and TUNEL staining (**G**). Scale bars, 100 μm. Results shown are representative of three independent experiments (mean ± SD) or of three independent experiments with similar results (two-way ANOVA in panels C, E). **, 0.001 < *P* < 0.01; ***, *P* < 0.001; ns., not significant.

*Eskandari E et al*. reported altered cell differentiation and viability in *caspase-3*^*-/-*^ mice, thus hindering the growth and development of mice [30]. Moreover, we observed that *caspase-3*^*-/-*^ mice displayed a small body size, poor growth state, and even death, whereas *caspase-3*^*+/-*^ mice presented a normal growth state. To investigate whether gM-induced caspase-3-dependent apoptosis was related with the pathogenicity of herpesvirus *in vivo*, WT and *caspase-3*^*+/-*^ mice were challenged intraperitoneally with PRV or HSV-1. Western blotting analysis of the isolated mouse peritoneal macrophages revealed lower caspase-3 expression in *caspase-3*^*+/-*^ mice than that in WT mice (Fig 9A). Moreover, 100% (10/10) of the PRV-infected WT mice died within 6 dpi, while 20% (2/10) of the *caspase-3*^*+/-*^ mice survived during the 15-dpi observation period (Fig 9B). Consistently, the PRV DNA copy numbers were considerably lower in the liver, spleen, lung, kidney, duodenum, and brain of *caspase-3*^*+/-*^ mice than those in WT mice (Fig 9C). Similar to PRV, the knockdown of caspase-3 *in vivo* increased the survival rate of mice by approximately 30% (Fig 9D) and reduced HSV-1 replication in tissues (Fig 9E). Additionally, reduction of lymphocytes in the white pulp of the spleen, nuclear pyknosis, cell fragmentation, and apoptosis (TUNEL-labeled) were observed in the PRV- or HSV-1-infected WT mouse. In contrast, these pathological findings were negligible in spleen of PRV- or HSV-1-infected *caspase-3*^*+/-*^ mice (Fig 9F and 9G). These results suggest that caspase-3-mediated apoptosis is closely associated with the pathogenicity of PRV and HSV-1.

To verify that the activation of caspase-3 to enhance the replication and pathogenicity of PRV and HSV-1 is closely related to gM, HeLa-WT and HeLa-*caspase-3*^*-/-*^ were infected with PRV-ΔUL10 or HSV-1-ΔUL10 for 24 h. We found that the deletion of caspase-3 did not affect the DNA copy numbers in the cells and viral titers in the supernatants of PRV-ΔUL10 (S7A and S7B Fig) and HSV-1-ΔUL10 (S7C and S7D Fig). Furthermore, the same survival rates were observed in WT and *caspase-3*^*+/-*^ mice after PRV-ΔUL10 (S7E Fig). Similar to PRV, the knockdown of caspase-3 *in vivo* did not affect the pathogenicity of HSV-1-ΔUL10 to mice (S7F Fig). These results suggest that, at the late stage of PRV or HSV-1 infection, the activation of caspase-3 to enhance the replication and pathogenicity of PRV and HSV-1 is specifically dependent on gM protein.

## Discussion

**Fig 10:**
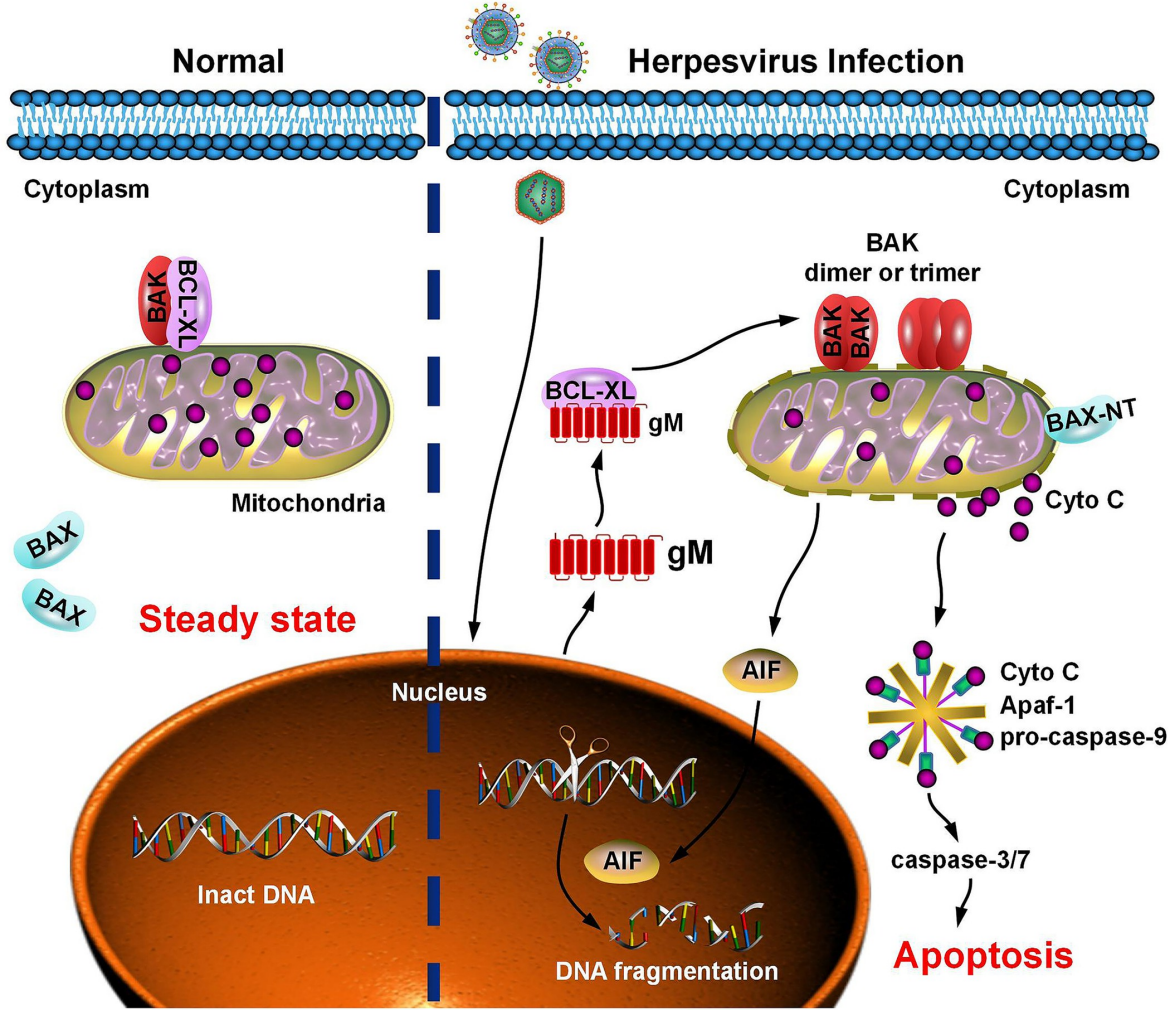
Schematic representation of herpesvirus-induced apoptosis at the late stage of viral infection. In the steady state, the pro-apoptotic protein, BAK, and the anti-apoptotic protein, BCL-XL, form dimers residing in the mitochondria, while BAX is dispersed in the cytoplasm. At the late stage of herpesvirus infection, herpesvirus-encoded gM competitively binds to BCL-XL and disrupts the BCL-XL-BAK complex, resulting in the oligomerization of BAK and activation of BAX. Subsequently, the mitochondrial membrane is damaged, leading to Cyto C release and AIF nuclear transportation, which promotes the formation of apoptotic bodies and DNA fragmentation. Ultimately, the apoptotic effectors caspase-3/7 are activated, and the infected cells undergo apoptosis, which is beneficial for herpesviruses to establish persistent infection and enhance their pathogenicity.

Apoptosis is a critical cellular defense mechanism against invading pathogens. As a countermeasure, viruses have evolved multiple strategies to manipulate apoptosis for immune evasion and viral replication. PRV infection induces apoptosis by activating p38 MAPK and JNK/SAPK signaling [31]. Additionally, caspase-3 activation is a consequence of HSV-1 infection-induced apoptosis [32]. However, the viral proteins involved in PRV-induced apoptosis and their underlying molecular mechanisms remain unknown. In this study, we report that PRV gM, a late envelope glycoprotein, competitively combines with BCL-XL to disrupt the BCL-XL-BAK complex, resulting in the oligomerization of BAK and activation of BAX. Subsequently, the mitochondrial membrane potential is destroyed to promote the release of Cyto C and AIF nuclear transportation, leading to caspase-3/7 activation and DNA fragmentation, which triggers mitochondrial-dependent apoptosis. Notably, PRV gM homologs in other herpesviruses, including HSV-1, HCMV, EHV-1, and VZV, had similar functions in inducing apoptosis (Fig 10). Furthermore, *UL10* gene of PRV and HSV-1 was identified as a key virulence-determining gene, which significantly reduced PRV and HSV-1 pathogenicity in mice. Consistently, caspase-3 deficiency impaired the replication and pathogenicity of PRV and HSV-1 *in vitro* and *in vivo*, suggesting that caspase-3-mediated apoptosis is a key regulator of replication and pathogenicity in these viruses. Unfortunately, we could not obtain HCMV, EHV-1, or VZV to verify the function of PRV gM homologs at the physiological level.

Apoptosis plays a critical role in the herpesvirus life cycle. In the early stage of viral infection, apoptosis disrupts virion assembly and limits viral replication, thus protecting the surrounding cells from viral infection. However, herpesviruses have evolved anti-apoptotic mechanisms to block the premature death of host cells; therefore, the propagation of progeny viruses or establishment of persistent infection can be maintained. For example, mouse cytomegalovirus-infected macrophages secrete soluble factors that induce apoptosis to inhibit viral replication, whereas mouse cytomegalovirus M36 blocks host apoptosis to sustain replication [33]. Interestingly, apoptosis appears to be a double-edged phenomenon. Herpesvirus infection or virus-encoded proteins induce apoptosis at the late stage of viral infection, which promotes the efficient dissemination of progeny viruses to neighboring cells [34]. It was reported that the immediate-early gene-encoded proteins, including ICP0, ICP4, ICP22, and ICP27, regulated late gene expression, which is required for HSV-1-induced apoptosis in human epithelial cells [35]. For example, HSV-1-encoded ICP27 induces apoptosis by promoting BAX translocation to the mitochondria by interacting with 14-3-3θ [36]. Moreover, HSV-1 infection leads to apoptosis, which destroys the blood-brain barrier, resulting in a high mortality rate and severe neurological sequelae. This is an important pathological basis for the development of HSV-1-mediated encephalitis [37]. Additionally, PRV infection also induced apoptosis, and low-dose Houttuynia cordata Thunb or emodin can effectively inhibit PRV-induced apoptosis to reduce the replication of PRV and increase the survival rate of mice [38,39]. Thus, PRV and HSV-1 infection-induced apoptosis is required for viral pathogenicity. The inhibition of apoptotic signaling at the late stage of viral infection is a promising therapeutic strategy for herpesvirus-related diseases. Consistent with previous reports showing that *UL10* deletion significantly reduces PRV plaque size, penetration kinetics [5, 40] and pathogenicity [41], this study elucidates the detailed molecular mechanism by which PRV gM induces apoptosis at the late stage of infection to enhance the replication and pathogenicity of PRV *in vitro* and *in vivo*, providing an important target for the development of vaccines and treatment of herpesvirus infection-related diseases.

Previous studies indicated that HSV-1 infection induced caspase-3-mediated apoptosis in a caspase-8-dependent manner [42]. In this study, we found that caspases 3/7/9 were activated in HSV-1- and PRV-infected HeLa cells; caspase inhibitor treatment partially reversed PRV infection-mediated apoptosis. Moreover, we also found that PRV and HSV-1 infections modestly activated caspase-8 in PAMs and THP-1 cells. Therefore, we speculate that caspase-8 may also be involved in herpesvirus-induced apoptosis. Additionally, PRV infection induces RIPK3-dependent necroptosis in PK-15 cells [43]. The activation of p38 MAPK and JNK/SAPK signaling, oxidative stress, and free radical formation have also been observed in PRV-induced apoptosis [44,45], suggesting that multiple signaling molecules participate in regulating PRV-induced apoptosis. Notably, in addition to PRV gM, several other PRV-encoded viral proteins, including UL56 and UL43, are involved in cell death. The involvement of these viral proteins in regulating PRV-induced cells damage and their molecular mechanisms for triggering cell damage require further investigation.

During PRV infection, apoptosis is prominent among infiltrating immune cells surrounding PRV-infected neurons, whereas majority of the PRV-infected neurons do not show morphological or histochemical evidence of apoptosis, including neurons surrounded by inflammatory cells exhibiting profound pathological changes [46]. Moreover, the PRV-infected trigeminal ganglionic neurons did not undergo apoptosis and maintained latent infection [46,47], suggesting that apoptosis was blocked in PRV-infected neurons. In addition, herpesviruses can establish a latent infection in the neurons of the peripheral nervous system, which establishes a high-density viral reservoir and evades host antiviral defense [48,49]. In our study, PRV gM formed aggresomes in the cytoplasmic, which induced apoptosis in HeLa and HEK293T cells. However, despite cytoplasmic aggresomes formation in Ln229 cells, the gM aggresomes gradually decomposed into small particles and disappeared in the cells after 72 h without inducing cell apoptosis. Thus, either the gM protein may be degraded by an unknown signal or gM-induced apoptosis was inhibited in Ln229 cells. Blocking apoptosis in PRV-infected neurons plays a pivotal role in the establishment, maintenance, and reactivation of herpesvirus latent infections. In latently infected neurons, the latency-associated transcript (LAT) is the only abundantly expressed gene that can silence lytic gene caspase-3 expression and DNA laddering to block apoptosis [17,50]. LAT has anti-apoptotic activity and is involved in HSV-1 reactivation [17,50]. However, whether LAT inhibits PRV gM expression and antagonizes gM-induced apoptosis in the latent herpesvirus infection process remains unclear. Moreover, it is reported that the initiation of host cell apoptosis and activation of caspase-3 are necessary and sufficient for the reactivation of latent herpesvirus [32,51]. Whether gM is involved in the reactivation of herpesvirus with latent infection remains unclear. Interestingly, a potential cellular candidate, Brn-3a, is highly expressed in trigeminal ganglionic neurons and dorsal root ganglion neurons, and protects them from α-herpesvirus infection-induced apoptosis [47]. Whether Brn-3a antagonizes gM-induced apoptosis in herpesvirus-infected neurons requires further investigation.

In conclusion, this study highlighted a novel role and key molecular mechanism for PRV gM and its homologous herpesvirus proteins in triggering mitochondria-dependent apoptosis by competitively interacting with BCL-XL to promote BAK oligomerization and activate the apoptosis effector caspase-3/7. Thus, gM is an important virulence-related factor of PRV and HSV-1, and caspase-3-mediated apoptosis driven by gM is closely related to their replication and pathogenicity, which suggests a potential approach for developing attenuated live vaccines and curing herpesvirus-related diseases.

## Materials and methods

### Ethics statement

All animal experiments were performed according to the animal protocols approved by the Subcommittee on Research Animal Care at the HVRI and were carried out in strict accordance with the recommendations of the Guide for the Care and Use of Laboratory Animals of the Ministry of Science and Technology of the People’s Republic of China.

### Cell lines and viruses

PAMs isolated from 30-day-old male SPF pigs. THP-1 and 3D4/21 cells were purchased from the American Type Culture Collection (Virginia, USA) and cultured in Roswell Park Memorial Institute 1640 medium supplemented with 10% fetal bovine serum, 100 U/mL penicillin, and 100 mg/mL streptomycin at 37°C with 5% CO_2_. HeLa, HEK293T, Vero, MPK, and IPEC-J2 cells were purchased from the American Type Culture Collection (Virginia, USA) and cultured in Dulbecco’s Modified Eagle’s medium supplemented with 10% fetal bovine serum, 100 U/mL penicillin, and 100 mg/mL streptomycin at 37°C with 5% CO_2_. HeLa-*caspase-3*^*-/-*^ cells were kindly provided by Professor Zhengfan Jiang (Peking University, China). The PRV-TJ strain was provided by Prof. Tongqing An (HVRI, China). The PRV-GFP strain was obtained from Prof. Ming Liu (HVRI, China). The HSV-1 strain was provided by Prof. Chunfu Zheng (Fujian Medical University, China).

### Antibodies and chemical reagents

Anti-caspase-3 (9662S), anti-cleaved-caspase-3 (D175) (9661S), anti-caspase-7 (9492S), anti-cl-caspase-7 (D198) (9491S), anti-cytochrome C (11940S), anti-COX IV (3E11) (4850S), anti-PARP1 (46D11) (9532S), and anti-HA-tagged rabbit monoclonal antibody (3724S) were purchased from Cell Signaling Technology (Massachusetts, USA). Anti-Flag-tagged rabbit monoclonal antibody (SAB4301135-100UL), anti-BAK (ab3237), and anti-AIF (ab32516), and DAPI (D9542) were purchased from Sigma-Aldrich. Anti-PRV-UL42 mouse monoclonal antibody was kindly provided by Prof. Changming Liu (HVRI, China). The anti-PRV-gM rabbit polyclonal antibody was prepared in the laboratory. Alexa Fluor 488-labeled goat anti-rabbit IgG and Alexa Fluor 594-labeled goat anti-mouse IgG were purchased from Thermo Fisher Scientific (Massachusetts, USA). Anti-GAPDH (60004-1-Ig), anti-BAX-NT (50599-2-Ig), anti-lamin B1 (12987-1-AP), and anti-BCL-XL (10783-1-AP) antibodies were purchased from Proteintech (Wuhan, China). Z-VAD (S7073), Z-LEHD (S7313), and Z-DETD (S7312) were purchased from Selleck Chemicals (Texas, USA).

### Plasmids

To establish eukaryotic expression plasmids, cDNA of the PRV-TJ *UL10* gene was amplified and cloned into pEGFP-N1, pCAGGS-Flag, and pCAGGS-HA vectors, and named pEGFP-UL10, pCAGGS-Flag-UL10 (Flag-UL10), and pCAGGS-HA-UL10 (HA-UL10), respectively. All the constructs were validated using DNA sequencing. Primers used in this study are presented in Table 1. Other eukaryotic plasmids expressing the gM homologs of HSV-1, HCMV, EHV-1, and VZV were synthesized by Jilin Kumei Biotechnology Co., Ltd. (Jilin, China). Plasmids containing pro-apoptotic proteins (BAX, BAK, BID, and BAD) and anti-apoptotic proteins (BCL-XL and BCL-W) were constructed and stored in our research laboratory.

**Table 1:** The primer sequences used in the study.

Plasmids	Primers (5′-3′)
Flag-UL10-F	GATGACGACGATAAGGAATTCATGTCCGGGCCGCGCAAC
Flag-UL10-R	ATTAAGATCTGCTAGCTCGAGTTATTCAAAGCCGAGGTT
HA-UL10-F	GTTCCAGATTACGCTGAATTCATGTCCGGGCCGCGCAAC
HA-UL10-R	ATTAAGATCTGCTAGCTCGAGTTATTCAAAGCCGAGGTT
pEGFP-C1-UL10-F	AAGTCCGGACTCAGATCTCGAGCAATGTCCGGGCCGCGCAAC
pEGFP-C1-UL10-R	TTATCTAGATCCGGTGGATCCTTATTCAAAGCCGAGGTT
Flag-UL10-1-118-F	GATGACGACGATAAGGAATTCATGTCCGGGCCGCGCAAC
Flag-UL10-1-118-R	ATTAAGATCTGCTAGCTCGAGTCAGCTGGGCTGGAACGCCAG
Flag-UL10-1-155-F	GATGACGACGATAAGGAATTCATGTCCGGGCCGCGCAAC
Flag-UL10-1-155-R	ATTAAGATCTGCTAGCTCGAGCGCCTGCTTGTGGGCCAT
Flag-UL10-1-204-F	GATGACGACGATAAGGAATTCATGTCCGGGCCGCGCAAC
Flag-UL10-1-204-R	ATTAAGATCTGCTAGCTCGAGTCAGACGGCGCGCGCGTG
Flag-UL10-1-268-F	GATGACGACGATAAGGAATTCATGTCCGGGCCGCGCAAC
Flag-UL10-1-268-R	ATTAAGATCTGCTAGCTCGAGTCACACCTGCACGTAGCC
Flag-UL10-292-393-F	GATGACGACGATAAGGAATTCATGGCCAAGTTCAGCGAGGCC
Flag-UL10-292-393-R	ATTAAGATCTGCTAGCTCGAGTTATTCAAAGCCGAGGTT
Flag-UL10-228-393-F	GATGACGACGATAAGGAATTCATGCTGGCCAACAGCTTCCAC
Flag-UL10-228-393-R	ATTAAGATCTGCTAGCTCGAGTTATTCAAAGCCGAGGTT
Flag-UL10-178-393-F	GATGACGACGATAAGGAATTCAAGGGCGCGGGTGAC
Flag-UL10-178-393-R	ATTAAGATCTGCTAGCTCGAGTTATTCAAAGCCGAGGTT
Flag-UL10-106-393-F	GATGACGACGATAAGGAATTCCGGCGCGAGGCCGGC
Flag-UL10-106-393-R	ATTAAGATCTGCTAGCTCGAGTTATTCAAAGCCGAGGTT
Flag-UL10-35-393-F	GATGACGACGATAAGGAATTCATGTTCGCCTCGCTCCCG
Flag-UL10-35-393-R	ATTAAGATCTGCTAGCTCGAGTTATTCAAAGCCGAGGTT

### Cells viability assay

LDH and cellular ATPase activity were measured to determine cells viability. HEK293T or HeLa cells were seeded in 48-well plates at a density of 1 × 10^4^ cells per well for 12 h and transfected with the indicated plasmids for 36 h. Cell supernatants were harvested and cleared by centrifugation at 2,000 ×g for 5 min, and 50 μL was used to perform the LDH assay using the CytoTox 96® Non-Radioactive Cytotoxicity Assay kit (Promega, Madison, WI, USA), according to the manufacturer’s instructions. Cell lysates were harvested and cellular ATPase activities were detected using the Cell Titer-Glo Luminescent Cells Viability Assay (Promega, Madison, WI, USA), according to the manufacturer’s instructions.

### Apoptosis assay and flow cytometry

Cells were infected with viruses at an indicated MOI, collected, and stained with PI (1 μg/mL) alone or together with Annexin V-FITC (0.1 g/mL) using the Annexin V-FITC/PI assay kit (BD Pharmingen, USA), according to the manufacturer’s instructions. Subsequently, 10,000 cells were analyzed and apoptotic cells were identified using a Cytomics FC 500 flow cytometer (Beckman Coulter Inc., USA) on the FL-1 and FL-3 channels.

### TUNEL assay

PRV-infected tissues from mice or PAMs, HeLa and HEK293T cells transfected with the indicated plasmids were fixed with 4% paraformaldehyde for 30 min, permeabilized with 0.3% Triton X-100 in 1 × PBS, and then stained with TUNEL reagent according to the manufacturer’s instructions using the One Step TUNEL Apoptosis Assay kit (Beyotime, Shanghai, China).

### Caspase activity and inhibitor assays

Caspase-3/7 and caspase-9 activities in PAMs and HeLa cells were measured using Caspase-Glo® 3/7 Assay Kits (Promega, Madison, WI, USA) and Caspase-Glo® 9 Assay Kits (Promega, Madison, WI, USA) according to the manufacturer’s protocols. PAMs were infected with PRV-WT or PRV-ΔUL10 at an MOI of 1, 5 or 10 for 24 h, or at an MOI of 5 for 12, 24, or 36 h. Alternatively, HeLa cells were transfected with plasmids expressing Flag-gM for 36 h. Cells were collected and detected according to the manufacturer’s instructions. For the inhibitor assay, cells were pre-treated with 50 μM caspase-3/7 inhibitor (Z-DEVD) or caspase-9 inhibitor (Z-LEHD) for 1 h. The cells were infected with PRV at an MOI of 1 for 24 h or transfected with plasmids expressing Flag-gM for 36 h; caspase-3/7 and caspase-9 activation were then detected.

### GST-pulldown assay

GST or GST-BCL-XL was expressed in *Escherichia coli* (BL21), and Flag-Vec. or Flag-gM was expressed in HEK293T cells for 36 h. Then, HEK293T cell lysates were harvested and incubated with purified GST or GST-BCL-XL proteins along with the Glutathione Resin (GenScript, USA) at 4°C for 12 h on a roller. The pellets were washed five times with cell lysis buffer and analyzed using western blotting.

### Co-IP and western blotting analysis

For Co-IP, cells were collected and lysed in lysis buffer (50 mM Tris-HCl, pH 7.4, 150 mM NaCl, 5 mM MgCl_2_, 1 mM EDTA, 1% Triton X-100, and 10% glycerol) containing 1 mM phenylmethylsulfonyl fluoride and a 1× protease inhibitor cocktail (Roche, Basel, Switzerland). Subsequently, the cell lysates were incubated with anti-Flag agarose or protein G plus agarose immunoprecipitation reagent (Santa Cruz Biotechnology, Santa Cruz, CA, USA) along with 1 μg of the corresponding antibodies at 4°C for 12 h on a roller. The pellets were washed five times with cell lysis buffer. For western blotting analysis, cell lysates and immunoprecipitants were boiled at 100°C for 10 min, resolved using 10–12% sodium dodecyl sulfate-polyacrylamide gel electrophoresis and then transferred to a polyvinylidene difluoride membrane (Millipore, Darmstadt, Germany). After incubation with the indicated primary and secondary antibodies, the membranes were visualized using an Odyssey 2-color infrared fluorescence imaging system (LI-COR, USA).

### Confocal microscopy analysis

The cells were fixed for 30 min in 4% paraformaldehyde diluted in 1× PBS (pH 7.4). The fixed cells were permeabilized for 20 min with 0.3% Triton X-100 in 1 × PBS and then blocked with 10% fetal bovine serum in 1 × PBS for 60 min. The cells were incubated with the indicated antibodies and stained with an Alexa Fluor 488-labeled goat anti-rabbit IgG or Alexa Fluor 594-labeled goat anti-mouse IgG antibody. The expression and subcellular localization of the target proteins were visualized using a Zeiss LSM-980 laser scanning fluorescence microscope (Carl Zeiss AG, Oberkochen, Germany) equipped with a 63× oil objective. Zeiss processing system software was used to determine the positive rate (%).

### JC-1 staining to determine mitochondrial membrane permeabilization

HeLa cells were infected with PRV as indicated, or transfected with Flag-gM for 36 h. Cells were then harvested and washed with 1× PBS. According to the manufacturer’s instructions of the MitoProbe Assay Kit (Invitrogen, Massachusetts, USA), the cells were stained with 2 nM JC-1 (5,5′,6,6′-tetrachloro-1,1′,3,3′-tetraethylbenzimi-dazolylcarbocyanine iodide, Cayman Chemical, USA) and incubated in a 5% CO_2_ incubator at 37°C for 30 min in the dark; following this, 10,000 cells were analyzed using Cytomics FC 500 flow cytometer (Beckman Coulter incorporated, USA) with appropriate excitation using emission at 529 nm (green) and 590 nm (red). The mitochondrial membrane potential energy was calculated as the ratio of red fluorescence intensity to green fluorescence intensity.

### DNA extraction and quantitative polymerase chain reaction

To detect the PRV genome copies, *gB*-specific absolutely quantitative polymerase chain reaction (qPCR) was performed as previously described [52]. Briefly, PRV genomic DNA was extracted from cells, or tissues using the Universal Genomic DNA Kit (AXYGEN, Silicon Valley, USA) and used for *gB*-specific absolute qPCR to precisely detect PRV genome copies. The primer and TaqMan probe sequences are listed in Table 2. The final PRV genome copy numbers were calculated based on a standard curve established using the recombinant pCAGGS-Flag-*gB* plasmid as a template. HSV-1 copies were detected using the primers listed in Table 2, and the DNA levels of HSV-1 *gD* were detected and normalized to those of *Gapdh* or *Actin*.

**Table 2:** Primers and TaqMan probe sequences.

Primers	Sequence (5′-3′)
PRV-*gB*-probe-F:	ACGGCACGGGCGTGATC
PRV*-gB*-probe-R:	ACTCGCGGTCCTCGAGCA
PRV*-gB*-TaqMan-probe:	FAM-CTCGCGCGACCTCATCGAGCCCTGCAC-MGB
HSV-1-*gD*-F:	TACAACCTGACCATCGCTTG
HSV-1-*gD*-R:	GCCCCCAGAGACTTGTTGTA
Human-*Actin*-F:	CCTTCCTGGGCATGGAGTCCTG
Human-*Actin*-R:	GGAGCAATGATCTTGATCTTC
Mouse-*Gapdh*-F:	TGGCCTTCCGTGTTCCTAC
Mouse-*Gapdh*-R:	GAGTTGCTGTTGAAGTCGCA

### Generation of *UL10* gene-deleted PRV and HSV-1

Recombinant PRV with *UL10* gene deletion (PRV-ΔUL10) was generated by homologous recombination in HEK293T cells transfected with a recombination transfer vector and then infected with PRV as described previously [41]. The following specific gene-targeted small guide RNA (sgRNAs) were designed (https://wwws.blueheronbio.com/external/tools/gRNASrc.jsp) and used to knock out the *UL10* gene in PRV and HSV-1, respectively: PRV-sgRNA-UL10, 5′-CACCGGCAACGCCGAGGCCGTGAGC-3′ and HSV-1-sgRNA-UL10, 5′-CACCGCGCGGACGGCGTGGTG GGTC-3′.

HEK293T cells were seeded in 6-well plates and transiently transfected with the sgRNA-UL10-CRISPR/Cas9 plasmid for 24 h. Cells were then infected with PRV or HSV-1 at 200 TCID_50_ for another 24 h. Supernatants were collected for plaque purification. Purified PRV-ΔUL10 and HSV-1-ΔUL10 were identified using PCR and sequencing (S1 Table), illumina whole-genome sequencing, and western blotting. The PCR primers for sequencing were as follows: PRV-UL10-F: 5′-AAGAAGCTGGTCACGGTGGG-3′; PRV-UL10-R: 5′-AGCTGCGCGTTGATCGTGGC -3′. HSV-1-UL10-F: CGTTGGGGAAGGTGACGGGTCCGTG CTGCTG; HSV-1-UL10- R: AAACCAAAACAATGTTCTGTATACGGTCGCA.

### Experiments in mice

Caspase-3 knockout mice were kindly provided by Zhengfan Jiang (Peking University, China). The genotypes of these mice were confirmed through sequencing using the primers listed in Table 3 and western blotting. Knockout mice and their WT littermates (6–8-week-old) were used in all experiments. All mice were housed in SPF barrier facilities at the HVRI of the Chinese Academy of Agricultural Sciences (CAAS) (Harbin, China). All animal experiments were performed according to animal protocols approved by the Subcommittee on Research Animal Care at HVRI.

**Table 3:** PCR primers used to identify the genotypes of mice.

Genotype	Primers (5′-3′)
*caspase-3*	F: GGTGATTGGGATCAAACCCA R: CAGGGCTTCTCTACTTTGCC

For infecting mice, 6–8-week-old and sex-matched SPF C57BL6J WT mice were intraperitoneally injected with sterile 1 × PBS (n = 4, 200 μL/mouse), PRV-WT (n = 12), or PRV-ΔUL10 (n = 12) (2,000 plaque forming units (PFU)/mouse, 200 μL/mouse). Similar to PRV, SPF C57BL6J WT mice were intraperitoneally injected with sterile 1 × PBS (n = 4, 200 μL/mouse), HSV-1-WT (n = 12) or HSV-1-ΔUL10 (n = 12) (1×10^7^ PFU/mouse, 200 μL/mouse). In addition, 6–8-week-old and sex-matched SPF C57BL6J WT mice and *caspase-3*^*+/-*^ mice were intraperitoneally injected with sterile 1 × PBS (n = 4, 200 μL/mouse), PRV-WT (n = 10) or PRV-ΔUL10 (n = 10) (2,000 PFU/mouse, 200 μL/mouse). Similar to PRV, WT and *caspase-3*^*+/-*^ mice were intraperitoneally injected with sterile 1 × PBS (n = 4, 200 μL/mouse), HSV-1-WT (n = 10), or HSV-1-ΔUL10 (n = 10) (0.5×10^7^ PFU/mouse, 200 μL/mouse). Survival rate of all the mice was monitored daily until 15 dpi. The mice were euthanized and blood and tissues (liver, spleen, lung, kidney, duodenum, and brain) from the infected mice were collected for qPCR, viral genome copies, or histological analysis.

### Histological analysis

The tissues of PRV-infected mice were fixed overnight in 10% formalin neutral buffer solution. Histological analysis of tissue damage was performed using standard H&E staining or TUNEL staining, and the results were analyzed using a light microscope.

### Statistical analyses

All data were analyzed using the Prism software (GraphPad, version 8.0). Statistical analysis was performed using unpaired, two-tailed Student’s t-test for two-group comparisons, log-rank test for survival experiments, one-way analysis of variance (ANOVA) of Dunnett’s multiple comparisons test for comparisons of more than two groups, and two-way ANOVA for comparisons of more than two groups with two or more time points. *P* values < 0.05 were considered to be statistically significant. *, *P* < 0.05; **, 0.001< *P* < 0.01; ***, *P* < 0.001; ns. was considered statistically non-significant.

## Supporting information

S1 Fig PRV infection induces tissue damage and cell death in piglets. Five specific pathogen free (SPF) piglets were mock-infected intranasally with PBS (n = 2, 1 mL/each) or infected intranasally with PRV (n = 3, 10^5^ TCID_50_/1 mL/each). (**A**) Survival rate. (**B**) PRV DNA copy number in tissues were analyzed using qPCR. (**C**) Pathological lesions (H&E staining) in the brain, lung, and tonsil. Scale bar, 50 μm. (**D**) Detection of dead cells in the tonsil and brain of piglets using TUNEL staining. (**E**) The percentages of TUNEL-labeled cells in (**D**) were quantified. Results shown are representative of three independent experiments (mean ± SD) or of three independent experiments with similar results (one-way ANOVA in panel E). ***, *P* < 0.001.
(TIF)

S2 Fig PRV infection induces apoptosis *in vitro*. (**A**) Representative morphological images of HeLa cells infected with PRV-GFP. HeLa cells infected with PRV-GFP at an MOI of 1 were monitored under a 63× oil objective using real-time confocal microscopy for 36 h. The black arrowhead indicates apoptotic cells. White number in top-left corner indicates relative time. (**B-C**) Representative images of cytopathic effects and TUNEL staining of PAMs (**B**) and HeLa cells (**C**) after PRV infection. PAMs and HeLa cells were mock-infected or infected with PRV at MOI of 5 or 1, respectively, for 36 h. Cells were fixed, stained with TUNEL (yellow), and visualized under a microscope. Apoptotic cells were detected and quantified using TUNEL staining. (**D-G**) Detection of apoptosis induced by PRV infection in various cell lines. PAMs (**D**), MPK (**E**), IPEC-J2 (**F**), and 3D4/21 (**G**) cells were either mock-infected or infected with PRV. Cells were harvested, stained with propidium iodide (PI) and Annexin V, and analyzed using flow cytometry. The percentages of PI- and Annexin V-labeled cells were quantified. Both PI- and Annexin V-stained cells showed later-stage apoptosis, and only Annexin V-labeled cells showed early-stage apoptosis. Results shown are representative of three independent experiments (mean ± SD) or of three independent experiments with similar results (one-way ANOVA in panels B-C, two-way ANOVA in panels D-G). *, *P* < 0.05; **, 0.001 < *P* < 0.01; ***, *P* < 0.001.
(TIF)

S3 Fig Generation of PRV-ΔUL10 recombinant virus. (**A**) The construction strategy of PRV-ΔUL10 recombinant virus. (**B-D**) Identification of PRV-ΔUL10 recombinant virus using gene sequencing (**B**), PCR amplification (**C**), and western blotting (**D**). (**E**) Growth curve analysis of PRV-WT and PRV-ΔUL10. Vero cells were infected with PRV-WT or PRV-ΔUL10 at MOI of 0.1 and PRV genomic DNA copy numbers were detected using qPCR. Results shown are representative of three independent experiments (mean ± SD) or of three independent experiments with similar results. **, 0.001 < *P* < 0.01; ***, *P* < 0.001.
(TIF)

S4 Fig The fourth transmembrane region (aa156-177) of PRV gM is mainly required for its subcellular localization and apoptosis. (**A-B**) PRV gM and its truncation mutants (**A**) as well as subcellular localization in HEK293T cells under laser confocal microscopy (**B**). (**C**) Detection of ATP enzymatic activities in HEK293T cells after the transient expression of Flag-gM or its truncation mutant with deletion of the fourth transmembrane region (Flag-gM_△156–177_). Transfection of plasmids expressing Flag-Vec. and Flag-BAK were used as negative and positive controls, respectively. (**D**) Apoptotic cell detection. Transfected HEK293T cells were harvested, stained with PI and Annexin V, and analyzed using flow cytometry. The percentages of PI- and Annexin V-labeled cells were quantified. Results shown are representative of three independent experiments (mean ± SD) or of three independent experiments with similar results (one-way ANOVA in panel C; two-way ANOVA in panel D). ***, *P* < 0.001.
(TIF)

S5 Fig Homology comparison and subcellular localization analysis between PRV gM and its homologs in the indicated herpesviruses. (**A**) Homologous comparison of the amino acid sequences from different herpesviruses. (**B**) Subcellular localization of PRV gM and its homologs from different herpesviruses in HEK293T cells.
(TIF)

S6 Fig Generation of HSV-1-ΔUL10 recombinant virus. (**A**) The construction strategy of HSV-1-ΔUL10 recombinant virus. (**B**) Identification of HSV-1-ΔUL10 recombinant virus using gene sequencing. (**C**) Growth curve analysis of HSV-1-WT and HSV-1-ΔUL10. Vero cells were infected with HSV-1-WT or HSV-1-ΔUL10 at an MOI of 0.1 and HSV-1 genomic DNA were detected using qPCR. Results shown are representative of three independent experiments (mean ± SD) or of three independent experiments with similar results. ***, *P* < 0.001.
(TIF)

S7 Fig Deletion of caspase-3 does not affect the replication and pathogenicity of PRV-ΔUL10 and HSV-1-ΔUL10. (**A-D**) Deletion of caspase-3 did not affect PRV-ΔUL10 (**A-B**) and HSV-1-ΔUL10 (**C-D**) replication. The pellets of PRV-ΔUL10- or HSV-1-ΔUL10-infected HeLa-WT and HeLa-*caspase-3*^*-/-*^ cells were used for PRV DNA copy numbers or HSV-1 genomic DNA detection using qPCR, and the cell supernatants were collected for TCID_50_ assay in Vero cells. (**E**) Survival rate. WT and *caspase-3*^*+/-*^ mice were challenged intraperitoneally with PBS or PRV-ΔUL10 at a dosage of 2,000 PFU per mouse (n = 4 in the PBS group, n = 10 in the PRV-ΔUL10-injected WT mice group, and n = 10 in the PRV-ΔUL10-injected *caspase-3*^*+/-*^ mice group) and their survival was recorded for 15 days. (**F**) Survival rate. WT and *caspase-3*^*+/-*^ mice were challenged intraperitoneally with PBS or HSV-1-ΔUL10 at a dosage of 1×10^7^ PFU per mouse (n = 4 in the PBS group, n = 10 in the HSV-1-ΔUL10-injected WT mice group, and n = 10 in the HSV-1-ΔUL10-injected *caspase-3*^*+/-*^ mice group) and their survival was recorded for 15 days. Results shown are representative of three independent experiments (mean ± SD) or of three independent experiments with similar results (two-way ANOVA in panels A-D). ns., not significant.
(TIF)

S1 Video PRV infection induces apoptosis. Morphological changes in HeLa cells infected with PRV-GFP at an MOI of 1 were monitored under a 63× oil objective using real-time confocal microscopy for 36 h. Scale bar: 20 μm. White number in top-left corner indicates relative time. Black arrowhead indicates apoptotic cells. White number in top-left corner indicates relative time.
(AVI)

S2 Video PRV gM induces apoptosis in HeLa cells. Morphological changes in HeLa cells transfected with a plasmid expressing the PRV GFP-gM were monitored under a 63× oil objective using real-time confocal microscopy for 36 h. Scale bar: 20 μm. White number in top-left corner indicates relative time.
(AVI)

S3 Video PRV gM fails to induce apoptosis in Ln229 cells. Morphological changes in Ln229 cells transfected with a plasmid expressing the PRV GFP-gM were monitored under a 63× oil objective using real-time confocal microscopy for 36 h. Scale bar: 50 μm. White number in top-left corner indicates relative time.
(AVI)

S4 Video PRV gM induces mitochondrial damage. Morphological changes in HeLa cells transiently transfected with plasmids expressing PRV-GFP-gM (green) and Mito-DsRed (red). were monitored under a 63× oil objective using real-time confocal microscopy for 36 h. Scale bar: 50 μm. White number in top-left corner indicates relative time.
(AVI)

S1 Table The DNA sequences covering the recombinant region of PRV and HSV-1. (DOCX)

S1 Appendix Comparison of *UL10* gene sequences between PRV-WT and PRV-ΔUL10. (JPG)

S2 Appendix The whole genomic DNA sequence of PRV-ΔUL10. (DOC)

S3 Appendix Comparison of the whole genomic DNA sequences between PRV-WT and PRV-ΔUL10. (HTM)

S4 Appendix Comparison of *UL10* gene sequences between HSV-1-WT and HSV-1-ΔUL10. (JPG)

S5 Appendix The whole genomic DNA sequence of HSV-1-ΔUL10. (DOC)

S6 Appendix Comparison of the whole genomic DNA sequences between HSV-1-WT and HSV-1-ΔUL10. (HTM)

## References

[1] Montague MG, Hutchison CA. Gene content phylogeny of herpesviruses. Proceedings of the National Academy of Sciences. 2000;97(10):5334–9. doi: 10.1073/pnas.97.10.5334 10805793 PMC25829

[2] Pomeranz LE, Reynolds AE, Hengartner CJ. Molecular Biology of Pseudorabies Virus: Impact on Neurovirology and Veterinary Medicine. American Society for Microbiology. 2005;69(3):462–500. doi: 10.1128/MMBR.69.3.462-500.2005 16148307 PMC1197806

[3] Brittle EE, Reynolds AE, Enquist LW. Two Modes of Pseudorabies Virus Neuroinvasion and Lethality in Mice. Journal of Virology. 2004;78(23):12951–63. doi: 10.1128/JVI.78.23.12951-12963.2004 15542647 PMC525033

[4] Liu Q, Wang X, Xie C, Ding S, Yang H, Guo S, Li J, Qin L, Ban F, Wang D, Wang C, Feng L, Ma H, Wu B, Zhang L, Dong C, Xing L, Zhang J, Chen H, Yan R, Wang X, Li W. A Novel Human Acute Encephalitis Caused by Pseudorabies Virus Variant Strain. Clin Infect Dis. 2021;73(11):e3690–e700. doi: 10.1093/cid/ciaa987 32667972

[5] Dijkstra JM, Visser N, Mettenleiter TC, Klupp BG. Identification and characterization of pseudorabies virus glycoprotein gM as a nonessential virion component. Journal of Virology. 1996;70(8):5684–8. doi: 10.1128/JVI.70.8.5684-5688.1996 8764089 PMC190535

[6] Baines JD, Roizman B. The open reading frames UL3, UL4, UL10, and UL16 are dispensable for the replication of herpes simplex virus 1 in cell culture. Journal of Virology. 1991;65(2):938–44. doi: 10.1128/JVI.65.2.938-944.1991 1846207 PMC239835

[7] Lehner R, Meyer H, Mach M. Identification and characterization of a human cytomegalovirus gene coding for a membrane protein that is conserved among human herpesviruses. J Virol. 1989;63(9):3792–800. doi: 10.1128/JVI.63.9.3792-3800.1989 2547996 PMC250972

[8] Pilling A, Davison AJ, Telford EA, Meredith DM. The equine herpesvirus type 1 glycoprotein homologous to herpes simplex virus type 1 glycoprotein M is a major constituent of the virus particle. J Gen Virol. 1994;75:439–42. 8113768 10.1099/0022-1317-75-2-439

[9] Klupp BG, Nixdorf R, Mettenleiter TC. Pseudorabies Virus Glycoprotein M Inhibits Membrane Fusion. Journal of Virology. 2000;74(15):6760–8. doi: 10.1128/jvi.74.15.6760-6768.2000 10888614 PMC112192

[10] Turner A, Bruun B, Minson T, Browne H. Glycoproteins gB, gD, and gHgL of Herpes Simplex Virus Type 1 Are Necessary and Sufficient To Mediate Membrane Fusion in a Cos Cell Transfection System. Journal of Virology. 1998;72(1):873. doi: 10.1128/JVI.72.1.873-875.1998 9420303 PMC109452

[11] Bock FJ, Tait SWG. Mitochondria as multifaceted regulators of cell death. Nature reviews: molecular cell biology. 2020;21(2):85–100. doi: 10.1038/s41580-019-0173-8 31636403

[12] Dorstyn L, Akey CW, Kumar S. New insights into apoptosome structure and function. Cell Death & Differentiation. 2018;25:1194–208. doi: 10.1038/s41418-017-0025-z 29765111 PMC6030056

[13] Boatright KM, Renatus M, Scott FL, Sperandio S, Shin H, Pedersen IM, et al. A unified model for apical caspase activation. Molecular Cell. 2003;11(2):529–41. doi: 10.1016/s1097-2765(03)00051-0 12620239

[14] Cecconi F, Alvarez-Bolado G, Meyer BI, Roth KA, Gruss P. Apaf1 (CED-4 homolog) regulates programmed cell death in mammalian development. Cell. 1998;94(6):727. doi: 10.1016/s0092-8674(00)81732-8 9753320

[15] Steain M, Slobedman B, Abendroth A. Modulation of Apoptosis and Cell Death Pathways by Varicella-Zoster Virus. Curr Top Microbiol Immunol. 2023;438:59–73. doi: 10.1007/82_2021_249 35624346

[16] Jerome KR, Tait JF, Koelle DM, Corey L. Herpes Simplex Virus Type 1 Renders Infected Cells Resistant to Cytotoxic T-Lymphocyte-Induced Apoptosis. J Virol. 1998;72(1):436–41. doi: 10.1128/JVI.72.1.436-441.1998 9420243 PMC109392

[17] Perng GC, Jones C, Ciacci-Zanella J, Stone M, Henderson G, Yukht A, Slanina SM, Hofman FM, Ghiasi H, Nesburn AB, Wechsler SL. Virus-induced neuronal apoptosis blocked by the herpes simplex virus latency-associated transcript. Science. 2000;287(5457):1500–3. doi: 10.1126/science.287.5457.1500 10688801

[18] Gabriele P, Katharina S, Laila S, Mansi R, Yvonne MG, Jonathan L, et al. Herpes Simplex Virus Infection of Dendritic Cells: Balance among Activation, Inhibition, and Immunity. Journal of Infectious Diseases. 2003;187(2):165–78. doi: 10.1086/367675 12552441

[19] Verpoest S, Cay AB, Van Campe W, Mostin L, Welby S, Favoreel H, De Regge N. Age- and strain-dependent differences in the outcome of experimental infections of domestic pigs with wild boar pseudorabies virus isolates. The Journal of general virology. 2016;97(2):487–95. doi: 10.1099/jgv.0.000347 26589961

[20] Xu C, Wang M, Song Z, Wang Z, Liu Q, Jiang P, Bai J, Li Y, Wang X. Pseudorabies virus induces autophagy to enhance viral replication in mouse neuro-2a cells in vitro. Virus Research: An International Journal of Molecular and Cellular Virology. 2018;248:44–52. doi: 10.1016/j.virusres.2018.02.004 29452162

[21] Sun M, Hou L, Song H, Lyu C, Tang YD, Qin L, Liu Y, Wang S, Meng F, Cai X. The relationship between autophagy and apoptosis during pseudorabies virus infection. Front Vet Sci. 2022;20(9):1064433. doi: 10.3389/fvets.2022.1064433 36605762 PMC9810027

[22] Nauwynck H, Glorieux S, Favoreel H, Pensaert M. Cell biological and molecular characteristics of pseudorabies virus infections in cell cultures and in pigs with emphasis on the respiratory tract. Veterinary Research: A Journal on Animal Infection. 2007;38(2):229–41. doi: 10.1051/vetres:200661 17257571

[23] Ren X, Li G, Sui X. Antiviral activities of phosphonoformate sodium to pseudorabies herpesvirus infection in vitro. Pharmaceutical biology. 2011;49(6):608–13. doi: 10.3109/13880209.2010.538416 21554003

[24] Alemañ N, Quiroga MI, López-Peña M, Vázquez S, Guerrero FH, Nieto JM. Induction and inhibition of apoptosis by pseudorabies virus in the trigeminal ganglion during acute infection of swine. J Virol. 2001;75(1):469–79. doi: 10.1128/JVI.75.1.469-479.2001 11119615 PMC113939

[25] Yuan JF, Zhang SJ, Jafer O, Furlong RA, Chausiaux OE, Sargent CA, et al. Global transcriptional response of pig brain and lung to natural infection by Pseudorabies virus. Bmc Microbiology. 2009;9(1):1–11. doi: 10.1186/1471-2180-9-246 19948073 PMC2793263

[26] Thornberry NA, Lazebnik Y. Caspases: Enemies Within. Science. 1998;281:1312–6. doi: 10.1126/science.281.5381.1312 9721091

[27] Green DR, Reed JC. Mitochondria and Apoptosis. Science. 1998;281(5381):1309–12. doi: 10.1126/science.281.5381.1309 9721092

[28] Birkinshaw RW, Czabotar PE. The BCL-2 family of proteins and mitochondrial outer membrane permeabilisation. Seminars in Cell and Developmental Biology. 2017;72:152–62. doi: 10.1016/j.semcdb.2017.04.001 28396106

[29] Wei MC, Zong WX, Cheng EH, Lindsten T, Panoutsakopoulou V, Ross AJ, et al. Proapoptotic BAX and BAK: a requisite gateway to mitochondrial dysfunction and death. Science. 2001;292(5517):727–30. doi: 10.1126/science.1059108 11326099 PMC3049805

[30] Eskandari E, Eaves CJ. Paradoxical roles of caspase-3 in regulating cell survival, proliferation, and tumorigenesis. The Journal of cell biology. 2022;221(6):e202201159. doi: 10.1083/jcb.202201159 35551578 PMC9106709

[31] Cheung AK, Chen Z, Sun Z, Mccullough D. Pseudorabies virus induces apoptosis in tissue culture cells. Archives of Virology. 2000;145(10):2193–200. doi: 10.1007/s007050070049 11087101 PMC7086653

[32] Hunsperger EA, Wilcox CL. Caspase-3-dependent reactivation of latent herpes simplex virus type 1 in sensory neuronal cultures. Journal of Neurovirology. 2003;9(3):390–8. doi: 10.1080/13550280390201678 12775421

[33] Ebermann L, Ruzsics Z, Guzmán CA, van Rooijen N, Casalegno-Garduño R, Koszinowski Ul, Čičin-Šain Luka. Block of death-receptor apoptosis protects mouse cytomegalovirus from macrophages and is a determinant of virulence in immunodeficient hosts. PLoS Pathog. 2012;8(12):e1003062. 23271968 10.1371/journal.ppat.1003062 PMC3521658

[34] Imre G. Cell death signalling in virus infection. Cell Signal. 2020;76:109772. doi: 10.1016/j.cellsig.2020.109772 32931899 PMC7486881

[35] Sanfilippo CM, Chirimuuta FN, Blaho JA. Herpes Simplex Virus Type 1 Immediate-Early Gene Expression Is Required for the Induction of Apoptosis in Human Epithelial HEp-2 Cells. Journal of Virology. 2004;78(1):224–39. doi: 10.1128/jvi.78.1.224-239.2004 14671104 PMC303390

[36] Kim JA, Kim JC, Min JS, Kang I, Oh J, Ahn JK. HSV-1 ICP27 induces apoptosis by promoting Bax translocation to mitochondria through interacting with 14-3-3θ. BMB Rep. 2017;50(5):257–62. 28256197 10.5483/BMBRep.2017.50.5.023 PMC5458675

[37] Qiang H, Hui L, Huang C, Wang R, Lu W. Herpes Simplex Virus 1-Induced Blood-Brain Barrier Damage Involves Apoptosis Associated With GM130-Mediated Golgi Stress. Frontiers in Molecular Neuroscience. 2020;13(2). 10.3389/fnmol.2020.00002 PMC6992570 32038167

[38] Ren X, Sui X, Yin J. The effect of injection on pseudorabies herpesvirus (PrV) infection. Pharmaceutical Biology. 2011;49(2):161–6. 20942608 10.3109/13880209.2010.505242

[39] Cai X, Wang Z, Li X, Zhang J, Ren Z, Shao Y, Xu Y, Zhu Y. Emodin as an Inhibitor of PRV Infection In Vitro and In Vivo. Molecules. 2023;28(18):6567. doi: 10.3390/molecules28186567 37764342 PMC10537396

[40] Brack AR, Dijkstra JM, Granzow H, Klupp BG, Mettenleiter TC. Inhibition of Virion Maturation by Simultaneous Deletion of Glycoproteins E, I, and M of Pseudorabies Virus. Journal of Virology. 1999;73(7):5364–72. doi: 10.1128/JVI.73.7.5364-5372.1999 10364283 PMC112592

[41] Tang YD, Liu JT, Wang TY, Sun MX, Tian ZJ, Cai XH. Comparison of Pathogenicity-Related Genes in the Current Pseudorabies Virus Outbreak in China. Scientific Reports. 2017;7(1):7783. doi: 10.1038/s41598-017-08269-3 28798304 PMC5552686

[42] Marino-Merlo F Klett A, Papaianni E, Drago SFA, Macchi B, Rincón MG, Andreola F, Serafino A, Grelli S, Mastino A, Borner C. Caspase-8 is required for HSV-1-induced apoptosis and promotes effective viral particle release via autophagy inhibition. Cell Death Differ 2023;30:885–96. doi: 10.1038/s41418-022-01084-y 36418547 PMC10070401

[43] Gou H, Bian Z, Cai R, Chu P, Li C. RIPK3-Dependent Necroptosis Limits PRV Replication in PK-15 Cells. Frontiers in Microbiology. 2021;12:664353. doi: 10.3389/fmicb.2021.664353 34149651 PMC8211757

[44] Yeh CJ, Lin PY, Liao MH, Liu HJ, Lee JW, Chiu SJ, et al. TNF-alpha mediates pseudorabies virus-induced apoptosis via the activation of p38 MAPK and JNK/SAPK signaling. Virology. 2008;381(1):55–66. doi: 10.1016/j.virol.2008.08.023 18799179

[45] Chang CD, Lin PY, Liao MH, Chang CI, Hsu JL, Yu FL, Wu HY, Shih WL. Suppression of apoptosis by pseudorabies virus Us3 protein kinase through the activation of P13-K/Akt and NF-kappa B pathways. Research in Veterinary Science. 2013;95(2):764–74. 23835241 10.1016/j.rvsc.2013.06.003

[46] Aleman N, Quiroga MI, Lopez-Pena M, Vazquez S, Guerrero FH, Nieto JM. Induction and Inhibition of Apoptosis by Pseudorabies Virus in the Trigeminal Ganglion during Acute Infection of Swine. Journal of Virology. 2001;75(1):469–79. doi: 10.1128/JVI.75.1.469-479.2001 11119615 PMC113939

[47] Geenen K, Favoreel HW, Nauwynck HJ. Cell type-specific resistance of trigeminal ganglion neurons towards apoptotic stimuli. Vet Microbiol. 2005;113(3–4):223–9. doi: 10.1016/j.vetmic.2005.11.007 16326038

[48] Knipe DM, Cliffe A. Chromatin control of herpes simplex virus lytic and latent infection. Nat Rev Microbiol. 2008;6(3):211–21. doi: 10.1038/nrmicro1794 18264117

[49] Steiner I, Benninger F. Update on Herpes Virus Infections of the Nervous System. Current Neurology and Neuroscience Reports. 2013;13(12):1–7. 10.1007/s11910-013-0414-8 24142852

[50] Carpenter D, Hsiang C, Brown DJ, Jin L, Osorio N, Mohamed B, et al. Stable cell lines expressing high levels of the herpes simplex virus type 1 LAT are refractory to caspase 3 activation and DNA laddering following cold shock induced apoptosis—ScienceDirect. Virology. 2007;369(1):12–8. 17727910 10.1016/j.virol.2007.07.023 PMC2276668

[51] Prasad A, Remick J, Zeichner SL. Activation of Human Herpesvirus Replication by Apoptosis. Journal of Virology. 2013;87(19):10641–50. doi: 10.1128/JVI.01178-13 23885073 PMC3807386

[52] Zhou Q, Zhang L, Lin Q, Liu H, Ye G, Liu X, Jiao S, Li J, Tang Y, Shi D, Huang L, Weng C. Pseudorabies Virus Infection Activates the TLR-NF-κB Axis and AIM2 Inflammasome To Enhance Inflammatory Responses in Mice. J Virol. 2023;97(3):e0000323. 36877049 10.1128/jvi.00003-23 PMC10062126

