# Supplementary figures and images for: Pseudorabies virus gM and its homologous proteins in herpesviruses induce mitochondria-related apoptosis involved in viral pathogenicity
Source: PLoS Pathog. 2024 Apr 26;20(4):e1012146. doi: 10.1371/journal.ppat.1012146 (PMC11051632; doi:10.1371/journal.ppat.1012146)

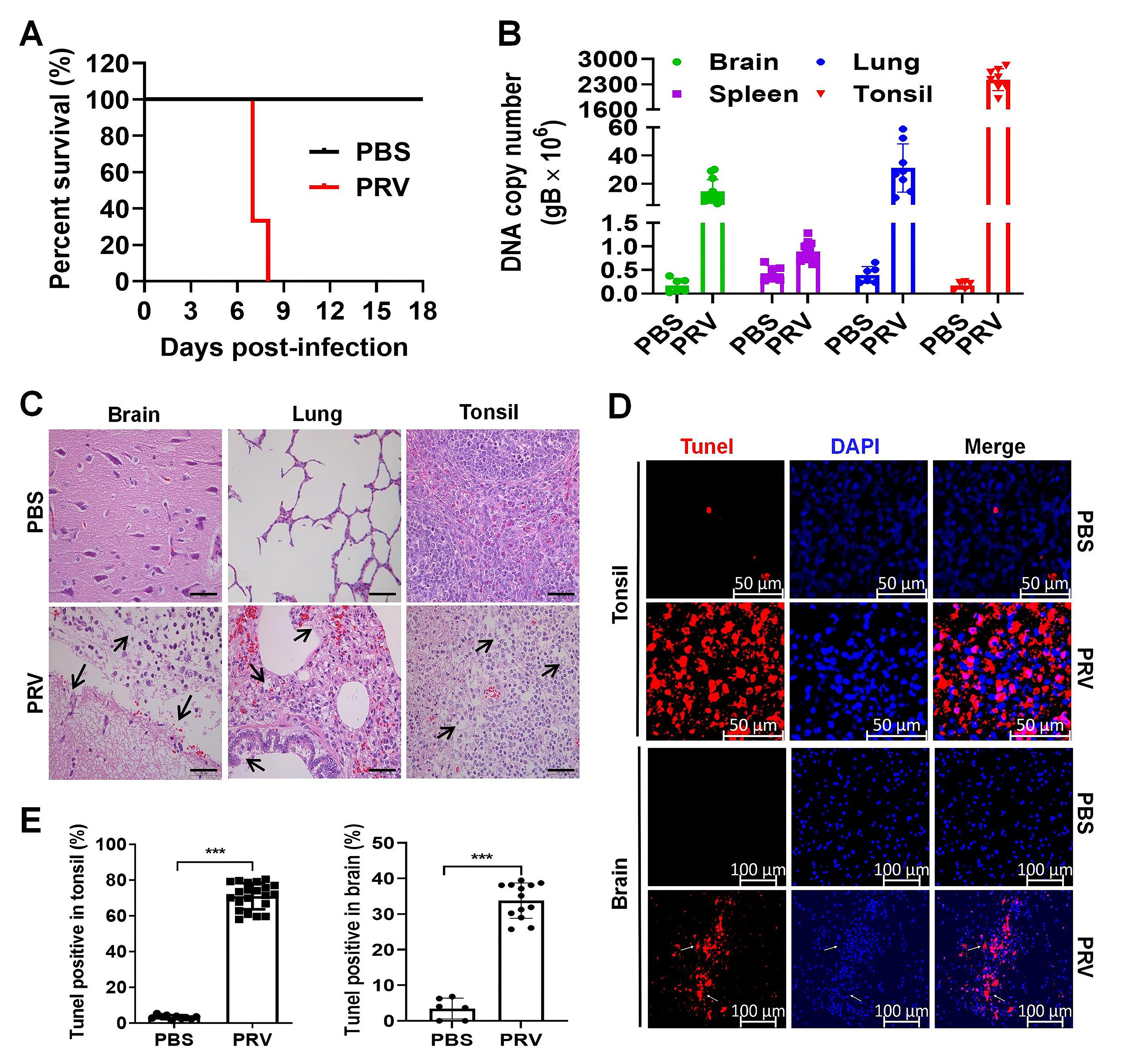

Supplement: S1 Fig — Five specific pathogen free (SPF) piglets were mock-infected intranasally with PBS (n = 2, 1 mL/each) or infected intranasally with PRV (n = 3, 105 TCID50/1 mL/each). (A) Survival rate. (B) PRV DNA copy number in tissues were analyzed using qPCR. (C) Pathological lesions (H&E staining) in the brain, lung, and tonsil. Scale bar, 50 μm. (D) Detection of dead cells in the tonsil and brain of piglets using TUNEL staining. (E) The percentages of TUNEL-labeled cells in (D) were quantified. Results shown are representative of three independent experiments (mean ± SD) or of three independent experiments with similar results (one-way ANOVA in panel E). ***, P < 0.001. (TIF) [file ppat.1012146.s001.tif]

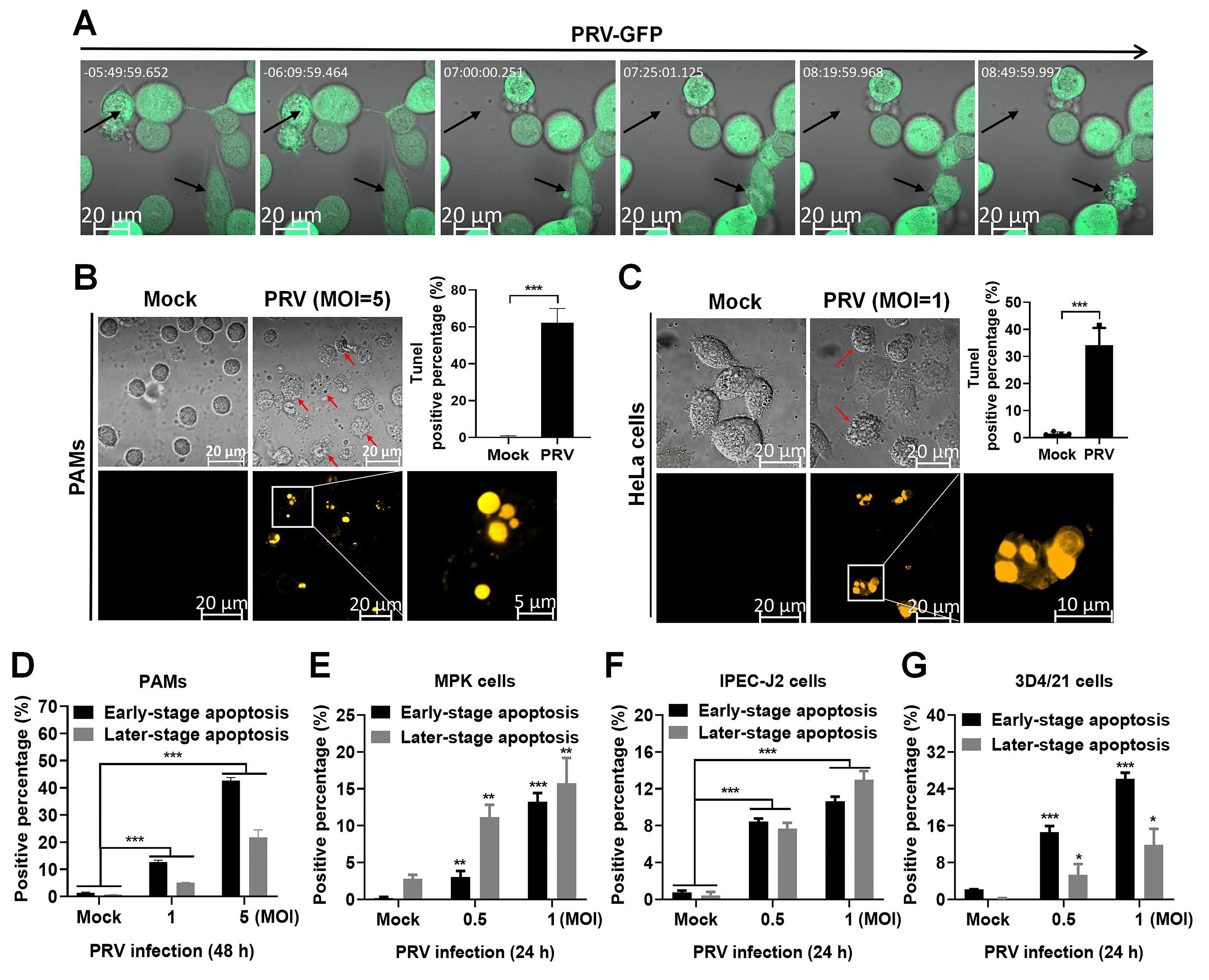

Supplement: S2 Fig — (A) Representative morphological images of HeLa cells infected with PRV-GFP. HeLa cells infected with PRV-GFP at an MOI of 1 were monitored under a 63× oil objective using real-time confocal microscopy for 36 h. The black arrowhead indicates apoptotic cells. White number in top-left corner indicates relative time. (B-C) Representative images of cytopathic effects and TUNEL staining of PAMs (B) and HeLa cells (C) after PRV infection. PAMs and HeLa cells were mock-infected or infected with PRV at MOI of 5 or 1, respectively, for 36 h. Cells were fixed, stained with TUNEL (yellow), and visualized under a microscope. Apoptotic cells were detected and quantified using TUNEL staining. (D-G) Detection of apoptosis induced by PRV infection in various cell lines. PAMs (D), MPK (E), IPEC-J2 (F), and 3D4/21 (G) cells were either mock-infected or infected with PRV. Cells were harvested, stained with propidium iodide (PI) and Annexin V, and analyzed using flow cytometry. The percentages of PI- and Annexin V-labeled cells were quantified. Both PI- and Annexin V-stained cells showed later-stage apoptosis, and only Annexin V-labeled cells showed early-stage apoptosis. Results shown are representative of three independent experiments (mean ± SD) or of three independent experiments with similar results (one-way ANOVA in panels B-C, two-way ANOVA in panels D-G). *, P < 0.05; **, 0.001 < P < 0.01; ***, P < 0.001. (TIF) [file ppat.1012146.s002.tif]

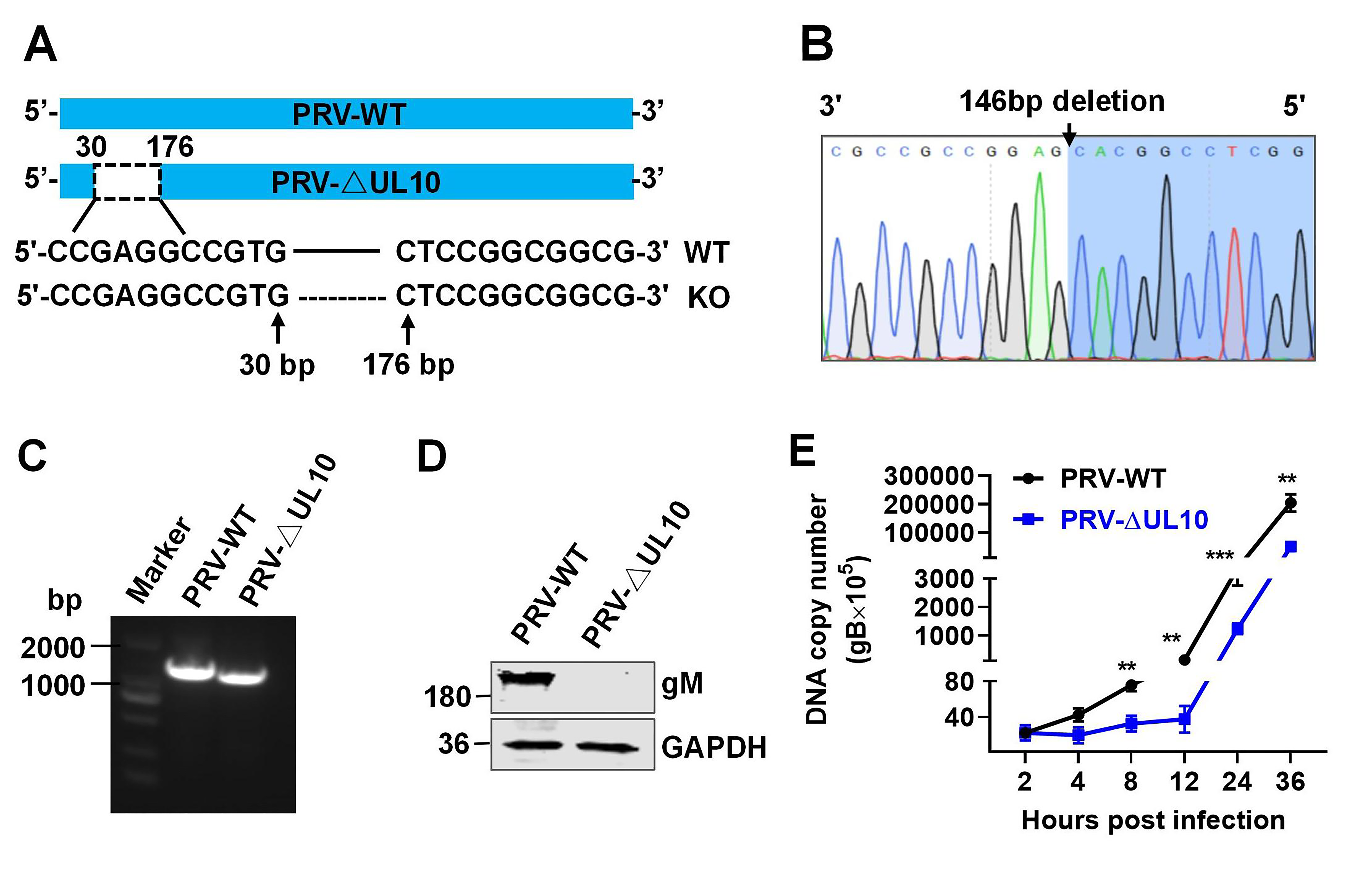

Supplement: S3 Fig — (A) The construction strategy of PRV-ΔUL10 recombinant virus. (B-D) Identification of PRV-ΔUL10 recombinant virus using gene sequencing (B), PCR amplification (C), and western blotting (D). (E) Growth curve analysis of PRV-WT and PRV-ΔUL10. Vero cells were infected with PRV-WT or PRV-ΔUL10 at MOI of 0.1 and PRV genomic DNA copy numbers were detected using qPCR. Results shown are representative of three independent experiments (mean ± SD) or of three independent experiments with similar results. **, 0.001 < P < 0.01; ***, P < 0.001. (TIF) [file ppat.1012146.s003.tif]

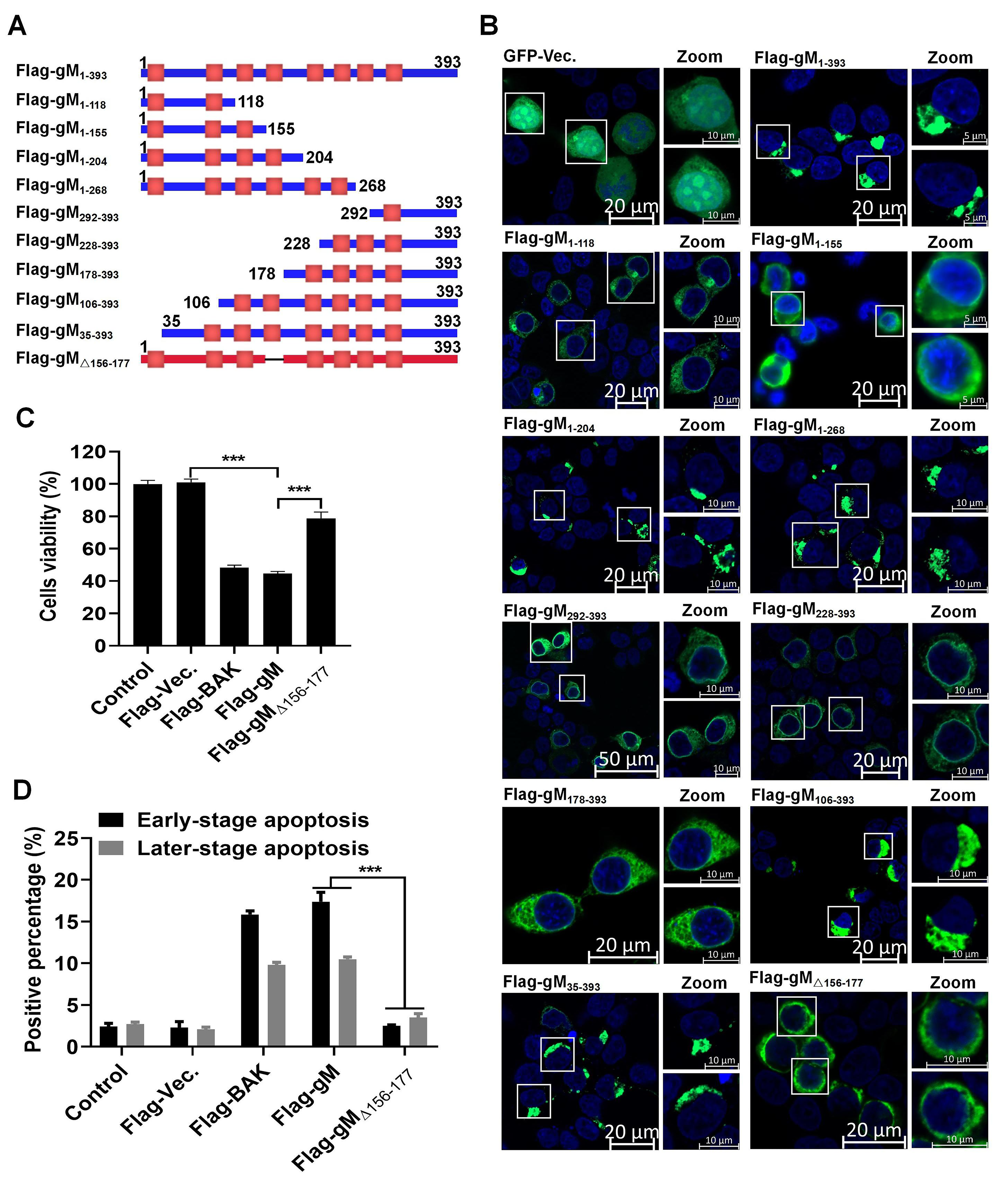

Supplement: S4 Fig — (A-B) PRV gM and its truncation mutants (A) as well as subcellular localization in HEK293T cells under laser confocal microscopy (B). (C) Detection of ATP enzymatic activities in HEK293T cells after the transient expression of Flag-gM or its truncation mutant with deletion of the fourth transmembrane region (Flag-gM△156–177). Transfection of plasmids expressing Flag-Vec. and Flag-BAK were used as negative and positive controls, respectively. (D) Apoptotic cell detection. Transfected HEK293T cells were harvested, stained with PI and Annexin V, and analyzed using flow cytometry. The percentages of PI- and Annexin V-labeled cells were quantified. Results shown are representative of three independent experiments (mean ± SD) or of three independent experiments with similar results (one-way ANOVA in panel C; two-way ANOVA in panel D). ***, P < 0.001. (TIF) [file ppat.1012146.s004.tif]

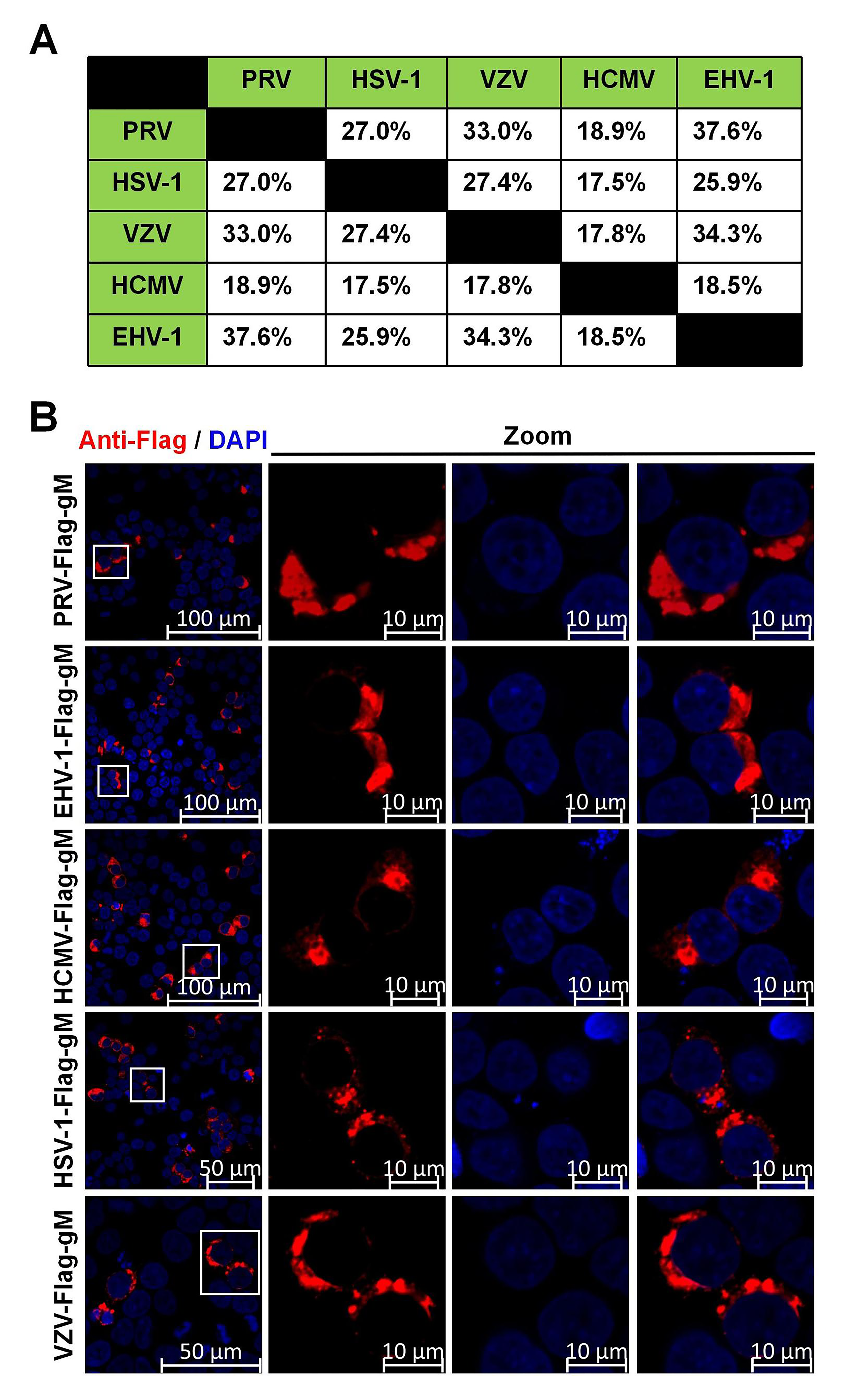

Supplement: S5 Fig — (A) Homologous comparison of the amino acid sequences from different herpesviruses. (B) Subcellular localization of PRV gM and its homologs from different herpesviruses in HEK293T cells. (TIF) [file ppat.1012146.s005.tif]

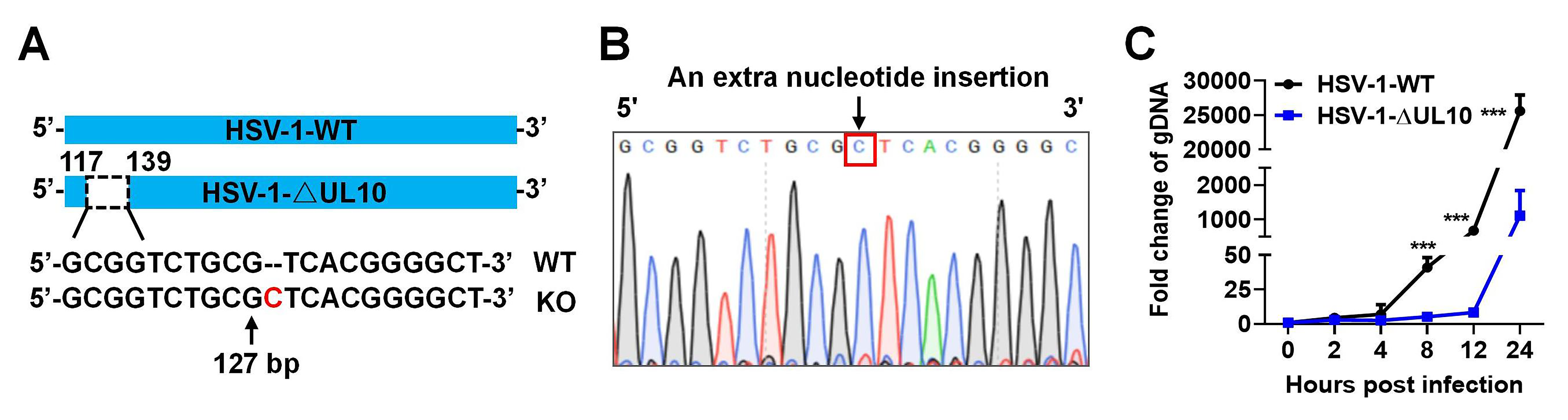

Supplement: S6 Fig — (A) The construction strategy of HSV-1-ΔUL10 recombinant virus. (B) Identification of HSV-1-ΔUL10 recombinant virus using gene sequencing. (C) Growth curve analysis of HSV-1-WT and HSV-1-ΔUL10. Vero cells were infected with HSV-1-WT or HSV-1-ΔUL10 at an MOI of 0.1 and HSV-1 genomic DNA were detected using qPCR. Results shown are representative of three independent experiments (mean ± SD) or of three independent experiments with similar results. ***, P < 0.001. (TIF) [file ppat.1012146.s006.tif]

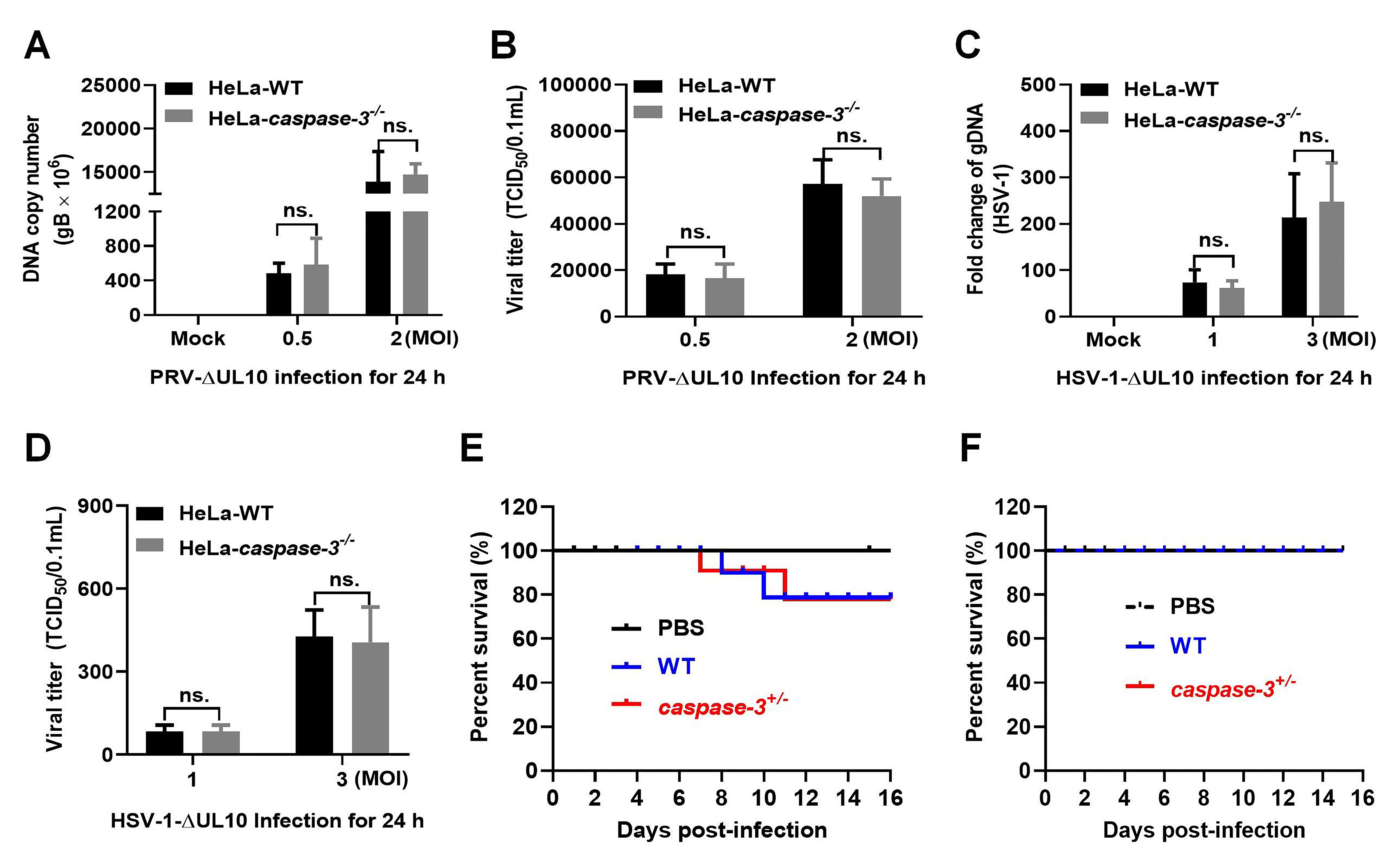

Supplement: S7 Fig — (A-D) Deletion of caspase-3 did not affect PRV-ΔUL10 (A-B) and HSV-1-ΔUL10 (C-D) replication. The pellets of PRV-ΔUL10- or HSV-1-ΔUL10-infected HeLa-WT and HeLa-caspase-3-/- cells were used for PRV DNA copy numbers or HSV-1 genomic DNA detection using qPCR, and the cell supernatants were collected for TCID50 assay in Vero cells. (E) Survival rate. WT and caspase-3+/- mice were challenged intraperitoneally with PBS or PRV-ΔUL10 at a dosage of 2,000 PFU per mouse (n = 4 in the PBS group, n = 10 in the PRV-ΔUL10-injected WT mice group, and n = 10 in the PRV-ΔUL10-injected caspase-3+/- mice group) and their survival was recorded for 15 days. (F) Survival rate. WT and caspase-3+/- mice were challenged intraperitoneally with PBS or HSV-1-ΔUL10 at a dosage of 1×10^7^ PFU per mouse (n = 4 in the PBS group, n = 10 in the HSV-1-ΔUL10-injected WT mice group, and n = 10 in the HSV-1-ΔUL10-injected caspase-3+/- mice group) and their survival was recorded for 15 days. Results shown are representative of three independent experiments (mean ± SD) or of three independent experiments with similar results (two-way ANOVA in panels A-D). ns., not significant. (TIF) [file ppat.1012146.s007.tif]

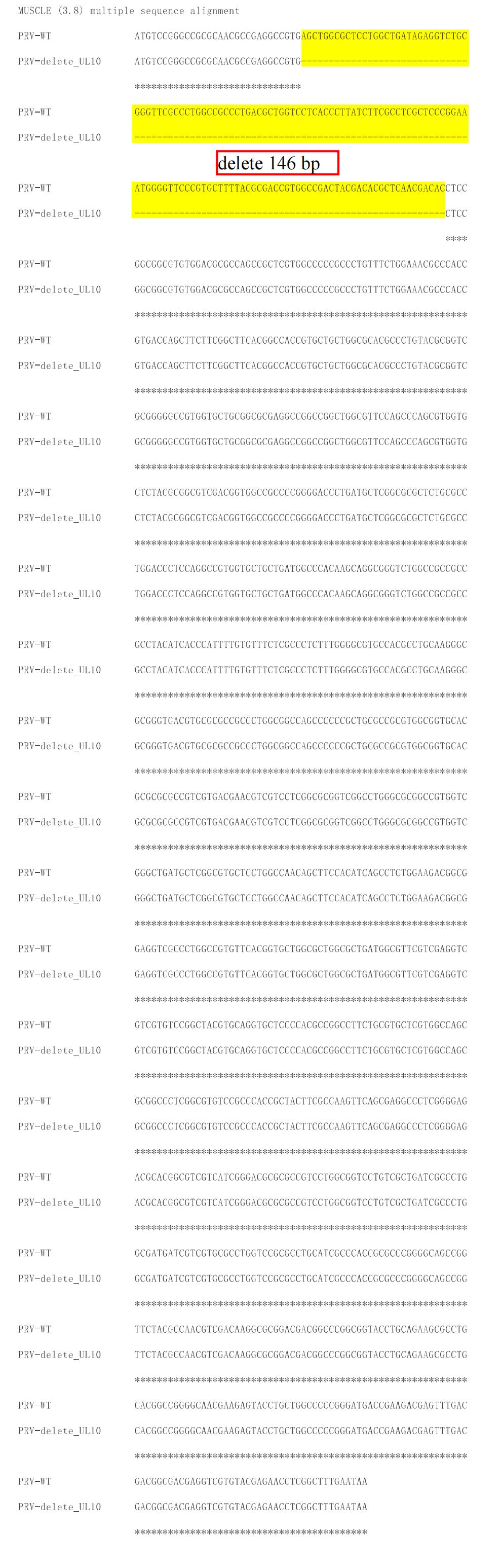

Supplement: S1 Appendix — (JPG) [file ppat.1012146.s013.jpg]

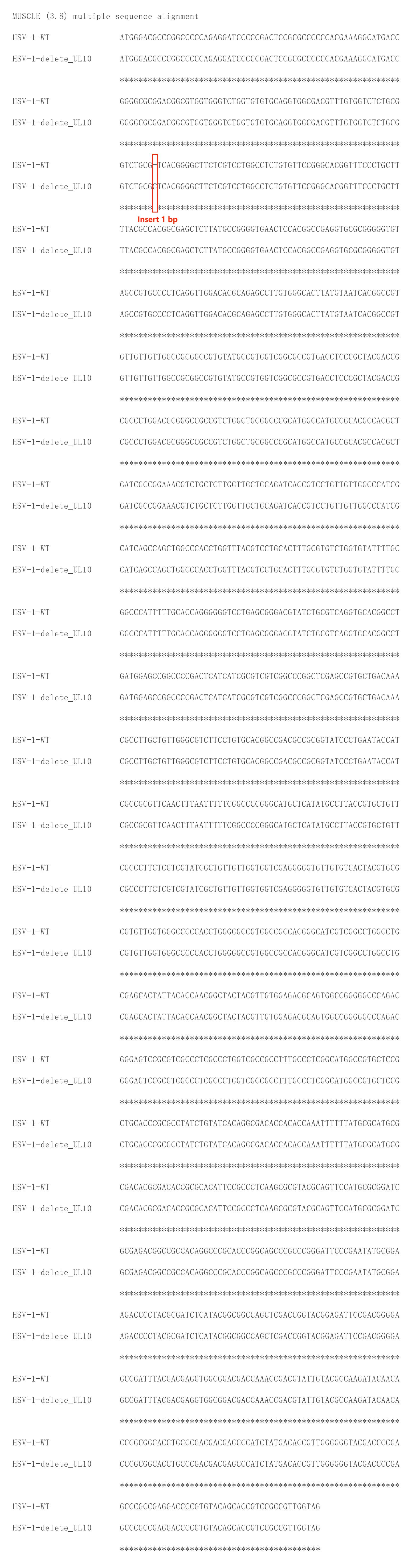

Supplement: S4 Appendix — (JPG) [file ppat.1012146.s016.jpg]
