# Supplementary material for: Pseudorabies virus gM and its homologous proteins in herpesviruses induce mitochondria-related apoptosis involved in viral pathogenicity
Source: PLoS Pathog. 2024 Apr 26;20(4):e1012146. doi: 10.1371/journal.ppat.1012146 (PMC11051632; doi:10.1371/journal.ppat.1012146)
Supplement: S1 Table — (DOCX) [file ppat.1012146.s012.docx]

**Table S1. The DNA sequences covering the recombinant region of PRV and HSV-1**

| Recombinant viruses | Sequence (5’-3’) |
| --- | --- |
| PRV-WT | ATGTCCGGGCCGCGCAACGCCGAGGCCGTGAGCTGGCGCTCCTGGCTGATAGAGGTCTGCGGGTTCGCCCTGGCCGCCCTGACGCTGGTCCTCACCCTTATCTTCGCCTCGCTCCCGGAAATGGGGTTCCCGTGCTTTTACGCGACCGTGGCCGACTACGACACGCTCAACGACACCTCCGGCGGCGTGTGGACGCGCCAGCCGCTCGTGGCCCCCGCCCTGTTTCTGGAAACGCCCACCGTGACCAGCTTCTTCGGCTTCACGGCCACCGTGCTGCTGGCGCACGCCCTGTACGCGGTCGCGGGGGCCGTGGTGCTGCGGCGCGAGGCCGGCCGGCTGGCGTTCCAGCCCAGCGTGGTGCTCTACGCGGCGTCGACGGTGGCCGCCCCGGGGACCCTGATGCTCGGCGCGCTCTGCGCCTGGACCCTCCAGGCCGTGGTGCTGCTGATGGCCCACAAGCAGGCGGGTCTGGCCGCCGCCGCCTACATCACCCATTTTGTGTTTCTCGCCCTCTTTGGGGCGTGCCACGCCTGCAAGGGCGCGGGTGACGTGCGCGCCGCCCTGGCGGCCAGCCCCCCGCTGCGCCGCGTGGCGGTGCACGCGCGCGCCGTCGTGACGAACGTCGTCCTCGGCGCGGTCGGCCTGGGCGCGGCCGTGGTCGGGCTGATGCTCGGCGTGCTCCTGGCCAACAGCTTCCACATCAGCCTCTGGAAGACGGCGGAGGTCGCCCTGGCCGTGTTCACGGTGCTGGCGCTGGCGCTGATGGCGTTCGTCGAGGTCGTCGTGTCCGGCTACGTGCAGGTGCTCCCCACGCCGGCCTTCTGCGTGCTCGTGGCCAGCGCGGCCCTCGGCGTGTCCGCCCACCGCTACTTCGCCAAGTTCAGCGAGGCCCTCGGGGAGACGCACGGCGTCGTCATCGGGACGCGCGCCGTCCTGGCGGTCCTGTCGCTGATCGCCCTGGCGATGATCGTCGTGCGCCTGGTCCGCGCCTGCATCGCCCACCGCGCCCGGGGCAGCCGGTTCTACGCCAACGTCGACAAGGCGCGGACGACGGCCCGGCGGTACCTGCAGAAGCGCCTGCACGGCCGGGGCAACGAAGAGTACCTGCTGGCCCCCGGGATGACCGAAGACGAGTTTGACGACGGCGACGAGGTCGTGTACGAGAACCTCGGCTTTGAATAA (Blue: deleted region) |
| PRV-△UL10 | ATGTCCGGGCCGCGCAACGCCGAGGCCGTG**************************************************************************************************************************************************CTCCGGCGGCGTGTGGACGCGCCAGCCGCTCGTGGCCCCCGCCCTGTTTCTGGAAACGCCCACCGTGACCAGCTTCTTCGGCTTCACGGCCACCGTGCTGCTGGCGCACGCCCTGTACGCGGTCGCGGGGGCCGTGGTGCTGCGGCGCGAGGCCGGCCGGCTGGCGTTCCAGCCCAGCGTGGTGCTCTACGCGGCGTCGACGGTGGCCGCCCCGGGGACCCTGATGCTCGGCGCGCTCTGCGCCTGGACCCTCCAGGCCGTGGTGCTGCTGATGGCCCACAAGCAGGCGGGTCTGGCCGCCGCCGCCTACATCACCCATTTTGTGTTTCTCGCCCTCTTTGGGGCGTGCCACGCCTGCAAGGGCGCGGGTGACGTGCGCGCCGCCCTGGCGGCCAGCCCCCCGCTGCGCCGCGTGGCGGTGCACGCGCGCGCCGTCGTGACGAACGTCGTCCTCGGCGCGGTCGGCCTGGGCGCGGCCGTGGTCGGGCTGATGCTCGGCGTGCTCCTGGCCAACAGCTTCCACATCAGCCTCTGGAAGACGGCGGAGGTCGCCCTGGCCGTGTTCACGGTGCTGGCGCTGGCGCTGATGGCGTTCGTCGAGGTCGTCGTGTCCGGCTACGTGCAGGTGCTCCCCACGCCGGCCTTCTGCGTGCTCGTGGCCAGCGCGGCCCTCGGCGTGTCCGCCCACCGCTACTTCGCCAAGTTCAGCGAGGCCCTCGGGGAGACGCACGGCGTCGTCATCGGGACGCGCGCCGTCCTGGCGGTCCTGTCGCTGATCGCCCTGGCGATGATCGTCGTGCGCCTGGTCCGCGCCTGCATCGCCCACCGCGCCCGGGGCAGCCGGTTCTACGCCAACGTCGACAAGGCGCGGACGACGGCCCGGCGGTACCTGCAGAAGCGCCTGCACGGCCGGGGCAACGAAGAGTACCTGCTGGCCCCCGGGATGACCGAAGACGAGTTTGACGACGGCGACGAGGTCGTGTACGAGAACCTCGGCTTTGAATAA (Blue *: deleted region) |
| HSV-1-WT | ATGGGACGCCCGGCCCCCAGAGGATCCCCCGACTCCGCGCCCCCCACGAAAGGCATGACCGGGGCGCGGACGGCGTGGTGGGTCTGGTGTGTGCAGGTGGCGACGTTTGTGGTCTCTGCGGTCTGCG*TCACGGGGCTTCTCGTCCTGGCCTCTGTGTTCCGGGCACGGTTTCCCTGCTTTTACGCCACGGCGAGCTCTTATGCCGGGGTGAACTCCACGGCCGAGGTGCGCGGGGGTGTAGCCGTGCCCCTCAGGTTGGACACGCAGAGCCTTGTGGGCACTTATGTAATCACGGCCGTGTTGTTGTTGGCCGCGGCCGTGTATGCCGTGGTCGGCGCCGTGACCTCCCGCTACGACCGCGCCCTGGACGCGGGCCGCCGTCTGGCTGCGGCCCGCATGGCCATGCCGCACGCCACGCTGATCGCCGGAAACGTCTGCTCTTGGTTGCTGCAGATCACCGTCCTGTTGTTGGCCCATCGCATCAGCCAGCTGGCCCACCTGGTTTACGTCCTGCACTTTGCGTGTCTGGTGTATTTTGCGGCCCATTTTTGCACCAGGGGGGTCCTGAGCGGGACGTATCTGCGTCAGGTGCACGGCCTGATGGAGCCGGCCCCGACTCATCATCGCGTCGTCGGCCCGGCTCGAGCCGTGCTGACAAACGCCTTGCTGTTGGGCGTCTTCCTGTGCACGGCCGACGCCGCGGTATCCCTGAATACCATCGCCGCGTTCAACTTTAATTTTTCGGCCCCGGGCATGCTCATATGCCTTACCGTGCTGTTCGCCCTTCTCGTCGTATCGCTGTTGTTGGTGGTCGAGGGGGTGTTGTGTCACTACGTGCGCGTGTTGGTGGGCCCCCACCTGGGGGCCGTGGCCGCCACGGGCATCGTCGGCCTGGCCTGCGAGCACTATTACACCAACGGCTACTACGTTGTGGAGACGCAGTGGCCGGGGGCCCAGACGGGAGTCCGCGTCGCCCTCGCCCTGGTCGCCGCCTTTGCCCTCGGCATGGCCGTGCTCCGCTGCACCCGCGCCTATCTGTATCACAGGCGACACCACACCAAATTTTTTATGCGCATGCGCGACACGCGACACCGCGCACATTCCGCCCTCAAGCGCGTACGCAGTTCCATGCGCGGATCGCGAGACGGCCGCCACAGGCCCGCACCCGGCAGCCCGCCCGGGATTCCCGAATATGCGGAAGACCCCTACGCGATCTCATACGGCGGCCAGCTCGACCGGTACGGAGATTCCGACGGGGAGCCGATTTACGACGAGGTGGCGGACGACCAAACCGACGTATTGTACGCCAAGATACAACACCCGCGGCACCTGCCCGACGACGAGCCCATCTATGACACCGTTGGGGGGTACGACCCCGAGCCCGCCGAGGACCCCGTGTACAGCACCGTCCGCCGTTGGTAG  (Blue *: an extra nucleotide insertion) |
| HSV-1-△UL10 | ATGGGACGCCCGGCCCCCAGAGGATCCCCCGACTCCGCGCCCCCCACGAAAGGCATGACCGGGGCGCGGACGGCGTGGTGGGTCTGGTGTGTGCAGGTGGCGACGTTTGTGGTCTCTGCGGTCTGCGCTCACGGGGCTTCTCGTCCTGGCCTCTGTGTTCCGGGCACGGTTTCCCTGCTTTTACGCCACGGCGAGCTCTTATGCCGGGGTGAACTCCACGGCCGAGGTGCGCGGGGGTGTAGCCGTGCCCCTCAGGTTGGACACGCAGAGCCTTGTGGGCACTTATGTAATCACGGCCGTGTTGTTGTTGGCCGCGGCCGTGTATGCCGTGGTCGGCGCCGTGACCTCCCGCTACGACCGCGCCCTGGACGCGGGCCGCCGTCTGGCTGCGGCCCGCATGGCCATGCCGCACGCCACGCTGATCGCCGGAAACGTCTGCTCTTGGTTGCTGCAGATCACCGTCCTGTTGTTGGCCCATCGCATCAGCCAGCTGGCCCACCTGGTTTACGTCCTGCACTTTGCGTGTCTGGTGTATTTTGCGGCCCATTTTTGCACCAGGGGGGTCCTGAGCGGGACGTATCTGCGTCAGGTGCACGGCCTGATGGAGCCGGCCCCGACTCATCATCGCGTCGTCGGCCCGGCTCGAGCCGTGCTGACAAACGCCTTGCTGTTGGGCGTCTTCCTGTGCACGGCCGACGCCGCGGTATCCCTGAATACCATCGCCGCGTTCAACTTTAATTTTTCGGCCCCGGGCATGCTCATATGCCTTACCGTGCTGTTCGCCCTTCTCGTCGTATCGCTGTTGTTGGTGGTCGAGGGGGTGTTGTGTCACTACGTGCGCGTGTTGGTGGGCCCCCACCTGGGGGCCGTGGCCGCCACGGGCATCGTCGGCCTGGCCTGCGAGCACTATTACACCAACGGCTACTACGTTGTGGAGACGCAGTGGCCGGGGGCCCAGACGGGAGTCCGCGTCGCCCTCGCCCTGGTCGCCGCCTTTGCCCTCGGCATGGCCGTGCTCCGCTGCACCCGCGCCTATCTGTATCACAGGCGACACCACACCAAATTTTTTATGCGCATGCGCGACACGCGACACCGCGCACATTCCGCCCTCAAGCGCGTACGCAGTTCCATGCGCGGATCGCGAGACGGCCGCCACAGGCCCGCACCCGGCAGCCCGCCCGGGATTCCCGAATATGCGGAAGACCCCTACGCGATCTCATACGGCGGCCAGCTCGACCGGTACGGAGATTCCGACGGGGAGCCGATTTACGACGAGGTGGCGGACGACCAAACCGACGTATTGTACGCCAAGATACAACACCCGCGGCACCTGCCCGACGACGAGCCCATCTATGACACCGTTGGGGGGTACGACCCCGAGCCCGCCGAGGACCCCGTGTACAGCACCGTCCGCCGTTGGTAG  (Blue C: an extra nucleotide insertion) |
