# Supplementary material for: Pseudorabies virus gM and its homologous proteins in herpesviruses induce mitochondria-related apoptosis involved in viral pathogenicity
Source: PLoS Pathog. 2024 Apr 26;20(4):e1012146. doi: 10.1371/journal.ppat.1012146 (PMC11051632; doi:10.1371/journal.ppat.1012146)
Supplement: S2 Appendix — (DOC) [file ppat.1012146.s014.doc]

>PRV-delete UL10

CCCCCCAGCCCCTCCGCTCCCCCTTTCCCCCAGCCCCTCCGCTCCCCCTTTCCCCCCAGCCCCCTCCGCTCCCCCCTTTCCCCCCAGCCCCCTCCGCTCCCCCCTTTCCCCCCCAGCCCCCTCCGCTCCCCCCTTTCCCCCCCTTTCTAAGTGGACGCCGCGGGCGCGCGGGCCCCCACACGGCGTTCGCTCTCGTCCCGGCCGGTCGGGCCGCCGGGACGGGGCGCTCGGCGCCGCGATGACCGACGCCTCGTCGCGGCCCGGCGGGGAAGAGCCGATGGAGGTGGACGAGCACGTCGGGCCGGAGCCCGAGCCCATGGAGGTGGACGAGCCCCCCGCACCGCCGTCGGCACCGGCACCGACGGCGGCGGGCACCATCCTCGCGGTGGGCTTCACCCAGCCCGCGCAGGTCCTGCGGGCGTACCCGATCGGTTGAGGTGCGAACGATGGGCGGGGGATGCCCGGGGTCGGACGCGCTCACGTGGTGCCCCACCCACCCCACGCCGCGCTCTTTTTTCCCGGGCCGCCCGACCGGATCCTCCCGCAGAGGCGGAGGTGAGGCCGGAGAGAGACAGGGAGAGATGATGGACGCGGAGAGGGGAGCGGCCGCCGCGGGGCGCGTGGAGGTGGAGCTCGATGCCCGGCCCTCCGGCGCCGGAGGGCGCGGCGCGAGCCCCTCGCGCCGGGCGCGGGACCCCCGAACCAGCCCGTGCTCCCGGGGCCGAGCCGACCGGCGTTCCCGGGACAGAACCGGCGACGTCACCGGGCCGCGGGCAGCGCGGCCCGCCACCCTGGGTATTAACGTCCCGGGCACCGCCGTCCGCCGCCAGCCGCCGTGGGAGGCAGACATGCCTCCACAACGAGCCCGCGGGGCTCCGCCGCGCCGTCGCGGCAGCGACCCGCCCGATCCAGGCAGCCTCGCCGGGCGGCCCTCGCCCGGGGGGAGAGGAGGCGGAGGGCAGCGCCGCCCCCTCTCGCGCAGCAGCTCGCTCACGTCCGTCGCCTCGGCGCCCGTGGAGACGCCCGTCGTCGCGGAGGCTCCGGGGCTCGGCGCCCCCGGCTCCAGGCCGCCCTCCTACGGGGACGTCGTCCGCGTCGGGCCGCGCCCGCACCGATCGCCGGACACGCCGCTGTTTGCCCGGGGCCCGCCCCCGTCCTACTCGGAGACGCTCCTGTTCGACCCGCCCGCGTACGCGGTGACCATCCCGGACCCGCCGGCGTACGAGCCCACCGTCATCGGGCCGCACCCGCCGCGCCCCCGCGACTGGATCTCCTCGCCCTCGGTGGTGCAGCCGTCGCTGCTGGGCCCCTTCAGCCAGTGCCTCCCGCGGGTGACCTGCCCCGACTGCCGCTACCCCGAAGACCGCCCGATGGTGCTCGTGGGCTTCCTCTGGGGGGGACTGCTCCTGCTGGTGGGCCTCGTGTTTCTGATCCTGCTCCCGGTGCTCCGGGAGTCCGTCGTGTTTCCCTGATCAATAAACTCGGCTCGCCCGCCCCATCGATATTCGCGTCTCGTGTGGTGCTGCGACGAGGGGGGGAAGAGGTGGGACGCGAAGAGATGAAGGGATCGGGGGGAAAGACGGCGAGAGGAGGAGCGGGAGTGTGCGGAAGGGACGGTGAAGCGGCGGTGGTGGAGGGAGATGGGGAGATGGAGGGAGATGGAGGGAGATGGGGAGATGGAGGGAGATGGAGGGAGATGGAGGGAGATGGGGAGATGGAGGGAGATGGGGAGATGGAGGGAGATGGAGGGAGATGGAGGGAGATGGGGAGATGGAGGGAGATGGAGGGAGATGGAGGGAGATGGGGAGATGGGGAGATGGGGAGATGGGGAGATGGGGAGATGGGGAGATGGAGGGAGATGGGGAGCGACGACGACAGACTCGCGCACGCCAGAGAGGTACGGTTCAACGGTTTTATTCAAAACAGGTGGTTGCAGTAAAAGTACTTCCCGTGCATGTACACGGGGACGAGGGTGTAGCTGCACGTCAGGCGGCAGGTGGGGGCGTCGCAGCCGGTCTTGTGGGCGTCCAGCGCGTCCATGATCCCGCCGAGGCAGGCGCCGGGCACGTACTCCTCCAGCGGGGCGGGCGAGTCCCCCAGGTCCTCGGGCAGCACGCAGCTCGAGCCGCGCTCGACGACGCGGGCGATGCGGGCCAGCATCACCAGGGTGTAGGTGACGATGCAGCGCACGTCCCGCAGGCGGCGCCGGCGCACGAGCTCGTCGACGGGCGTGTCGCGGAGGTAGCAGCGCAGGAACGGGCCGAGCTTCAGGCGCAGGTTCTGGAGCACCGCCCCCGCGGTGGCCACGATGGGGTCCAGCGTGCGCAGCGTGAGCCCCTTGGTGAGGATGAGCTTGACCCACGTGAGCGTCTCGTCCGCCGAGGCGAGGGACTCCGTCAGGCCCTCGTTCAGGAGCACCATCTCGCGGAGCACGCGCGCCGCCTCGGTGGCGTGCGGCCGCACCTCAAACAGGCGGTGCAGGTCGGCCCCGTGGAACATGAGCGTCTCCCACGTGACGCGCCGCCCGTCCGGGGTGAACTGCTCGGCGCCGAACTCGAGGACGGCGGCCCACGGCGACGAGCCCGCGGCGCGGAAGCCGTCGTTGAACACGGGGCGGGAGAGGTCGCGCCGCGCCGAGGCGAAGAGGTCGTTGACGCGCGTCGCGCCGACCCGCGCGTGCGTCGCGGAGACGGTGGCGCGCGCGCTCTCCGACAGGCGGTCGGGGGGCGCGTCGGGCCCGCTGTCGGGACGATCCGCCTCCGGCCGCCGCCGCTGGTGCTGCTGCTGGCGCTGCTGCTGCTGTTGCTGTCGCTGCCGCTGTCGGAGGGCCTGTCGGGCGGCGCGCCCGCCGCGACCCCCGCGCCGGCGCCGGACGGCCCCGAGCCGCGCCCGGACGGGCGGCCGCTCGGAGGGGCGCGCGCCCAGGCGCTCGCGGACCGGGCGCCGCTCCTCGCTCCCCGACCGGGACGCCTCGCTGCCGCTGCTGTTGCCGCTGTCCTCCATGACCGTGGTGCGAGCGGGGCCGCGGTGTGCCGCCGGCGGGCGGCGGGGCGCCTTTAACCCCGCGCCCCCGCGCGACCTTCATCCAAATATGGTAATGTGGCGCTCGTAGTGCAGAAACAGCAACACGCCCCCTACCAGCAGGAGGAGGTACAGCGCCTTGGCAAAGATCCCGGCGAGCACGGTCGAGCAGCAGGCGCCGCAGACGCCGAGGGCGGCGCGCCCGCCGTCGGCGCGCGCGGGCGGGGCCGTCGCGCCGGAGACGCGCGCGATGTGCGGCAGCGTCGCCGCCAGGAGCTCCAGCGCCCCGACCGCCAGCACAAAGAGCCACACGAGGGCGCGGGCGTACAGCGGGAAGACGAGGGCGCACGGGGCGCGCGTGACGCCGAGGGTCGTGAACAGCGCCATGCGCGCCCCCAGGCGGAGCCCGATCTCGAGGAGGATGAGCGCGATCAGCGTGGGGTGGCGGTGCGCCAGCGTGATGGGGTCCTCGCGGAGGTCGCGGCTGAGCGCGGCGCGGCGCGTCGCGAGCTCGGCCATGTACCAGGCGAACTTGGTGTAGCTGCAGCCGATGACCGTGGCGGCGAGCACGCTCGCCGCGTAGTTGAGCGTGTACTTCTCCGGGGTCAGGAACTCGGCGGGGTCGCGGAAGGGGCCGAACATGCGCCGCTCCTGGCGCGCGTACACGAAGGCCGCGTAGAGGAGCCACGCGAGGGCCGCCAGGCGAAAGTGCACGTCCCACAGGTAGGCGCGGCAGTCGCGGCGCGCGTACACGACCCGCGCGCGGTCGCGCAGCTCCGGGCTCAGCGCGCCCGTCTCGTTGGGGCCCACGTAGATGGCCGTGGCGTTGAAGGCCTCCCAGTCCGGCGCGCCGCCGCCGGCGTGCTCGCCGAGCGGCAGCGCCGCGTACACGACGGGGTGGGGCAGGCGGGCGACGGTCGCGTAGTACGCGCCCAGGCCGGCGTACGCGCCCATCACCCCGAGGACGAGGAGGTGCAGCGGGCGTCCGCCGAGGAGCATGATTGCCCCACCTCCGCGGTGAAAATGGTGCGCGTCGCGTTGCTGCCGCACTTTGTAGCGAAGCACTGCTGACTCAGCGAGATGCACAGCCGGTCGTGGGCGTCGACGCTCAGGGCCACGAAGGTGCGGGCCGGCGGGCTGCGCGCGTGCCGGGCCCGCAGGCAGCTGAAGCGCGCGGGCCCGCAGAGCTGGAAGAGCACCCAGTCGGGCTTGGCCACCACCCGCTGCGCGGTCGCGCCCGCGTACTCGTGCGCGCGCCCGTTGAAGTGCGCGTCGAGGTGCCGCAGCACGGGCGCCATGGCCACGTCCGCCACGAAGGCCTCGAGCGCGCGCCGGTCCTCCGCGTCCACGGCCAGGCCGCGCAGCATGTCCGCCAGCGGCGTGCGCCGGGCGAAGAAGGCCTCGCGGTTCCGCGCCGCCTTGCGCTCGAAGAAGCTCTCGTACTCCCCGCCGAGGCTGTGCAGCACGCGCGTGACGGCGTGCGCCGGGCGGGCGTGGAAGTGAAAGTTGCGCGGGTCGTCGAAGCCCGGGGGCGCGTCCCCGAAGGGGAGCAGGCGCCCGTGGCAGCCGCCGCCGTCGACCTTGGCGAAGAAGGGCAGCCGCAGGCTGCGCCCGTGCGCGTACACGCCCGTGTCCACGAAGGTGAAGTCCTGGAGGTACGGGCCCATGGGCTCGATAAAGTCCCGCTCCAGCAGCACGGCCTGCTGGAGCACACGCGCCAGGCCGCGGACCGTGTCGGCGCCGGCCACCGCGTACGGCGACGGCACGGGCGTGCACACGCGGAAGCCCATCTTGGCGCGGCAGCCGCACGCCGGGCGCTCGTCCGCCGCGGGGGCGCGCGCGGGGCCGGGTTCGGGGTCGGGGGCGGCGGCGGAGGAGGAGCCGGGGATCCCGGCCCCGACCTCGTCGTCGTCGCACGTGTACACCTCGGCGGGGGCGCCCGCGTCCCAGTCCGCCCAGTCCTCGCCGTCCGGCGCGGCGCCAAAGTCGGCGGGGGCGGCGACGTCGTCGTCCCAGGGCGCCCCCTCGAGGCCGTCGTCGGAGGAGGCCGCGGCGGCGGACCCCGCGGCGCCGGCGCCCCCGGGCGGACAGGCGCTCTTGTACACGTAGCACGGGTGCGCGGCCCAGTCGCCGTCGGCCTCGGGGAAGAGCAGCGCCAGCGCGTCGAGCACCGCGCGGCGGAACCCGCGCATGGCCGCCTCGAGCGTGCCCGCGGGCACCGGGCGCGCGAGGCGAAAGTCGACGTCGAGGACGATGTTGGTCACGGCCAGCCGGGCGTTGAACACCTCGTGGCGGCTCACGTACATCTGCGCCGCCGGCTCGTGCGCCGCCATCTCCGGCGCCGCGTCGCGCCGCGCCGTCGCGCGCCCCCAGGCGTCCCCGGCCAGCACCGCGAAGGCCTGGCGCCGGTGCGCCAGCTCCACGCGGTACACCGGCGCCGGGGGGCGCGCGTCCGGCAGGCCGAGCAGGGCGCCCAGCGCCGTGCGCGCGCGCCCGCCGCCCGGCGCGGCGGCCAGCTCCAGCAGGCGGCGCAGGACGGTGCCGTCGGCGCCCGGCTGCTGCTGCTGCGAGTGCGCCTGCTCCTCGTCGGGGGCGCGCACCCAGCCGTGGGGCGCGAGGCGCTCCTCGACGGCGGTCACGTGGCGCGCGAGGCCGGCGGCCGCGTCGCAGAGCCCGCGCGGCGCGTCGGCCGCGAGCGCGGCGTACGTGCGGTCCTCCACGTAGCCGGCGCCCACGGCCGGGGCGAGGCGCGCGACGCTCGGCGTCACGTTCTGGGCGACGTAGTCGCGGATGTTGAGCTGCGCCCGCACGTGGCGGAAGAAGGTGGCGGCCGCGCGCTCCCCGAGGGGCGAGCGGCCCACGGGCGCCGCGGGGTCCGTGACGTTGACGGCGTCCAGGTGCTCGCGGAGCCGCGCGCGGTTGAAGCTCTCAAAGTGGGCCAGGTAGACGTACGTCACGAACTCGCGGTCGGGGAGCTTGAGGCTGCCGCGGTCGTGGGCGATGTAGTCCCGGAGGACGCCCATCTCGGCGCGGTCGGCGCGCACGCGCTCGTCGACGTAGCGCGCGGCGCGCGCCGCCGCGGGCCCCCGCGCGTAGCCGCTCAGGCAGCAGAAGCGCGAGAGCGCCGCGAACGAGAGCAGCCCCGCGGGGTCCAGGCCGCTGGGGTTGGGCCCCACGCGCGGGTTGTACGTGAGCAGCAGGTCCTTGAGGGCCCGCAGGTCGTAGGCCGGCCGCGCGTGGAAGAGGTGGAGGCGCGTGGCCAGCACCAGCGTCTTCTCCTCGGGCCCGAAGCGCGCCACGTACCAGAAGGGCGTGTTGTGGTTCCCGTAGAGGCGCCGGAAGGCGGCCAGCGTCTGGGTCTCGTGGTGCACAAAGAGCGAGGTCAGCCCGCGGCGCCCGACCGCGATGGAGCGCAGCGGGCGCTCCGGCGCGTACTCGGCGTTGCCGGTGCGCGGGCGCATCTGCTCGAGGACCATGGTCAGGGCCGCGATGAGGCCGTCGTGGAGCGCGAAGGTGGCCGCCTCGTCCATGGCCTCGAGGACGTCCCGCGCCGCGAGGGGCTCCCCGCGGCAGAGCGCGCGGACGACCGCCGGGGTCGCGGCGGCGACGAACACGGGCCGCACGCGGACGCTGGCCACGTGCCCGGTGAGGCAGAACGTGACGCCGGGGCGGCGCGCTAAAAGCTCGGCCTCGGCGCGCTCGCCCGGCAGTGAGAAGGCCTCGTCCAGCCCGGCGCCGTCCCACGCGTACGAGACGACGTAGACCGCCCCCGAGGGCTCCTGGCCCGTGAGCAGCATCAGCGAGTACGTGATCGCGCAGCCGTCCGTGGCGTACAGGACGCGGATCATGCTGGGCGGCATCTTCTCCGGGTGGTGCGGGCGCCGCGGCCGTGGGAGGGCTTATGAGCCGCCGGACGAACTCCGCCTCCGGGAGTCGCTGGCCGTGCTCAACGTGATCCTCCCCGCCCCGCTCGCCGCCGAGGACGTGATGGCCTCGGCCGAGGCCGCGCGCCGCCTGGCCCGCGCGGACACGCTGGCGCGCACCTACCAGGCCTGCCAGCGCAACCTCGAGTGCCTGGCGCGCCACGAGGCCTCCGGCGACGACGGCCTGGACGCCGTGGTCGCGGCGCACGCGGCCAACGCGCGGCGGCTCGCGGACACGTGCCTGGCGGCGCTCATGCACCTGTACCTCTCGGTCGGCGCCGTCGACGCGGACACGGACGACCTCGTCGAGCAGGCCCTGCGCATGACCGCCGAGAGCAACGTGGTGATGTCCGACGTGGCCGTCCTGGAGCGGACGCTGGGACTGGCCCGGCGGCGGCAGCCGGCGCCCGTCCGGGCCGCGCCCGCCGGGGTGGGCCTGCCCGCCCCCGTCCCGCCCGCCCCGCACGTCGCCGCCCCGCCGGCCCCGCAGACCCCGCCCGAGGAGGAGGCGGATGCGGAGGCGGAGGAAGACGAGGCTGGCGGGGACCGCCCCGAGGAGGACGCGGTCCCGCTGCTGCCCCCGCGCGCCCCCGTCAAGACGCGCCCGCCCGAGAAGAAGAAGGTCCGGAGGCTGGCCGAGGCCTGCAAGATGTAGACGGAGAGCGGGGGGAGAGAGAGGGCACGCGCGAAGAGAAGAGACGAGGGGCGACCGAGAAGAAGGGGACGGAGACCACCGGGTGTTTTTTTTTCTTTTCAATAAACATCTTTATTGAAAAAAAAGAGAAGCGTCCTGCGTGTGTTGTGTGCGCGCGCCCGGGTGCCATCGCCATCGTCCCGGCGCGGGCCACCGCGGCCTCACAGGCCCGTGGAGCCGAAGCCGCGGGTCCCGCGCGCGCTGCTCGGGGCGACAAAGTCCCGCGTGAAGAGCCACGCGGGGCGGTGCTGCATCGGGGTGCGCGGGGTGGCGGGGAACGGGGACCGCCCCGTGATCCAGCTCAGCGGCTCGCGCGTCAGCACCAGCTGCGCCACGCGCTGGCCCGCCTCCAGGGTCACCGGGTGCGCGCCGCGGTTCTGGATCCGGAAGCGGCACGGGCCGCTCTCCCAGGGCGTGGGGAAGACGACGATGCCGCGGAGGTTGAGGGACGAGCGCCCGAAGACGTAGGCCCAGTGGCGGCCGTCCGTGCGGTGCACCGGCAGCGCCACGGTCTCCGCGCCGCCGGGGGGCAGCACCAGCTCGCGCGGGCAGGGGATGTCGTATCCGGCGTCCTCGTCGCGCTTCGGCGCAAAGACCTCGAAGAAGGGCACGTCCGTGGCGATGCCCGGCGCGAGGCGCAGCGGCGTGCAATAAAACTGCGCCCGTGGGCGGCCCGGGGCTACAATCGCCTGCACCTCGCCGCGAAAGCCCGCGTCCACGATCCCGTTGGTGGTGCGACCGCCCCCGCGCTGCGCGACGAGCATCGCGTATCCGGTGGGCAGCGCCACCTTGAGGCCCAGGGGAACCTTATAAAATCCCTCCGGCCCCGGACTCCGGCACACCAGCAGCCGCCCTCCGTCCACTGTCACGGGCTCGCTCGCAGACACCAGGATGGTCTCTTCCTCCGCCCGGCTCTCGCTGACGCTCGTCGCCGCGCTCTGCGCGCTGGTGACGCCCGCGCTCTCTTCCATCGTCTTTTCCGAGGGGTCGCTGCCGCTGCTGCGCGAGGAGTCGCGGACCAGCTTCTGGAGCGCCACCTGCGCCGCCAGGGGAGTCCCGGTGGATCAGCCGACGGCCGCCGCCGTGACATTTTATATCTGCCTCCTGGCGGTGCTCGTCGTCGCCCTGGGTTACGCCACCCGGACGTGCGCCCGGATGCTGCACACCTCCCCCGCGGGGAGGCGCGTATAAAGATCGATCCGCACGCGCCCGATCCCACTCGCTCGCCATGTCCAGCTCGAGAAAGACCCGGGTCGCCGCCGACGAGACCGCCTCGGGGGCGCGCCGCCGCGCCGGCAGCGCCTCTCGCGCCCGGGCCGCCGCCCCGGCCGTCCCGACCGCCGCGACCCCCAGACGCCCCTCGGCCTACGACGACGGCTTCTCCTACCGGTCTGCCCCGTCCTACGACGACGATGACTACTACGGCTACGATGGCCACGGCTCCTCCCACGCCCCCCGCGCCGCCAAGGTGACGCCCGCGGCCGCCTCGCGGGCCTCGACCGGGGCCAAGAGCGCCTCGGCCGCCAAGACCCCCGCGGCCAAGACCGCCCGCTCGGCCTCGGCCGCCGCCCCGGCCGCCGCCGCCACCACCGCCGCCGCCGCGGAACCGGCCGCCCGGCGCGCCTCGACGCGGGCCGCCCCCGGGGAGAACCTCGACGTGGGCCGCCGCCGGCTCGCCTTCAGCGACAGGCCGTGCGAGGCCAACGTGCCCTGGCGCGGCGCGACGCACGCCTTCAACAAGCGCATCTTCTGCGCGGCCGTGGGGCGCGTGGCCGAGGCCCACGCCCGCGCCGCCGCCGAGTCCCTCTGGGACATGAACCCCCCGACGACGGACGCGGCGCTCGACCGCTTCCTGCAGGCCGCGGTGGTGCGCATCACCGTGTGCGAGGGGCTGGACCTGATCGAGGCGGCCAACGCCGTCCTGGACGAGAGCACCCCGGGGCGGAAGGGAAAAGTGTATAAATAAAGGCGTTGCAAATGGCACCCCGCTCAGTCGTCCGCTGTCTTGCTTGTGGGGGGGTCGGTGAGGATGCGCGACGAGGAGTGCGTGGTCGCGTTCGACGAGGCCCTGCTGGGGGGCGCGGCCCCGGGCCGGCCGCCCGTGGTGGCCGTCCCGCGGGCGGCCAGCCCGGACGCGCTGTACCAGCGCCTCATCAGCGACCTGGCCTTCGACGAGGGCCCGGCGCTGCTGAGCGCCATGGAGCGCTGGAACGAGGACCTGTTCTCGTGCCTGCCCGGCAACGAGGACCTGTACGCGCGCATGGTCATGCTCTCGGCCTCCGCGGACGAGGTGGCGGCCCAGGTCCAGGCGCCGACGGCGGACGCGAGCGTCGACCTCGGGGTGCCCGGGGCGGAGCCCATGCCGCGGGTCCCGGCCGTGGAGGAGGAGCTGCCCGCGTACGTGGCGGCCGTGGAGCGCTTCTTCGTGTCCACGCTGCGGGCGCGCGAGGAGCGGTACGCGCGCCTGCTGGACGCCTACTGCCGCGCGGTGCTGCGCTACCTGCACGCGCAGGCGCGCCGCGACGAGGGCCGGCTCCGGCAGCTGGTGCGCGGGCGCTACTACCGCGACGTCGCGCGCCTGGCGCGCCTGCTCTTTCTGCACCTGTACGTGGCCACGGCGCGCGAGCTCTCGTGGCGCCTGCACGCGGACCAGGTGGTGGCCCAGGACGTGTTCATGTCCCTGCGCTACGAGTGGGACCAGACGCGGCAGCTGACGTGCCTCTTCCACCCGGTGCTGTTCAACCACGGCGTGGTGCTGCTGGCGGGCGCGCCGGTGCCGGCCGCGCAGCTGCGCGCCGTCAACCGCCGCCGGCGCGAGCTGGGCCTGCCGCTCGTGCGCGCCGGCCTCATCGAGGAGGACGGCGCCGACCTGGTCGAGGAGCCGCCGTTCTCCGCCGCGCTGCCGCGCGCCGCCGGCTACCTCTCGCACCAGGTGCGGATCAAGATGGAGGCCTACTCGCGCGAGTACCGCGACCACACGTACTGCCGCCCGCCCTCGCCCGTGGCCAGCTACGGCAGCACCGCGGAGGCCCTGCTGCCGCCCCCGTCGCCCTCGGCCGTGCTGCCCTGCGACCCGACGCCGCCGCCGCGCGTCTCGGCCGCCCCGCTCATCACCACGGTGACGCTGGCCGAGGCCGAGGAGGACGGCGCGCTGACGACGGCGGCGCCCGCGGCCGCGGTCGCGACCGCGACGGTCGCCTCGCCCGGGCCCGCCACCCCCGCCTACCACCTCATCCCCCGCGACGCGCTCAACCGCATGTTTGAGATGTGACGCCGCGCCCCGCGCGCGCGGTCGGATCTGCGGCGCCCGACCACCTGTGGGCGGGGCGTATAAAGCCGCGGGCCGGCCCGGAGGGGGGGCATTTGCCGCAGCGACCACCATGAGCGACGCGGGGCCCGTGCGGCGCCCGAGGCGCTCGCAAGAGCAGCGCTTCCAGCCGTTCCCCCGCGCGGGGCTCCTGGTGCACCTGCAGGACGTGCTCGTGGGCGAGGTGCGGCGCCCGGACTTCCGCCCTCCGCCCGACGAGGAGAGCAGCGAGGAGGAGGAGCCCGTCTGGACCGACGACGAGGAGGAGGACGAGGGGGAGGAGGGGGGAGCCCGGCGCGGGGACGGGGAGGGGGACGACGACGTGGACGTGGACGACGAGGAGGAGGAGAGCGAGGGGGGCGCGTGGTCCGACGGGGAGCTGTACGTGGCGGCCGACGGCGACGCGTGGGACGCGGAAGAGGACGAGGACGGGGACGAGGATGAGGATGATGACTATGACGATGACGACGACGGCTATGACGGCCGCGGCGCCGCGGCCTCCCGCGCCGCGGTCGGGCCCCCCGCCGACGTGGACTACCTGTCGCGCGCCCTCCGCGGCATGGAGACGGCCTCCGGCCCCGACGAGCGGGAGGCCTTCACCCGCTACGCCGGCACGCTCTACCGCCGCCAGTTCCAGCCCGGGCGGCCGGGGTACCGGCCCCCGCGGCCGCGCGACCCGCCGCCTCCCCCGCCCCCGCGGGCGCGGCCGCCGCTGACGACGGCGACGACGGCCGCGCCCGCCGTCGGCACCGCCGCCCCCTGCGGCGGCGACGACTCCGCGCCGCGCCTCGAGCGGGACCCGGACGCCGCGTACGCCAGCTGGACGCGCGACATGCCCGACGGGACCTTTCTGGCCATGCCGTCGTGGGTGGCGCTGCGCCGCGAGCCCCGCGGGCCCCCGGGCCGCCCCGTCGGGCCCCCGGACATCCTGCGCCGCGCCCCGCGGGCCCTGACCTTCACGCCGCAGGCGGCCTCGGCGGCGCTCGTGAGCCGCGACCAGGACGCCTGGGAGGCGCTGGTGCCGCTGCACCACCTGCAGATGCACTCGTGGGGCGGCAGCGCGCCGCCCTCGCGCGGGGGCGTGTACGCGACGCCCGCGCCCGAGACGCGCGGCGTGTGGCGCCGCGCGCTGCGGCAGGCCATCGCCCTGCACCACGCCATGTTCCTGGCGCCGCTGCAGTCCTCGACGGTCGGCGCGCCCGCGCTCGCGGGCGAGGCGCTGGCCTTCGCGCTGGACGCCGCGGCGCGCGCGGCCGTCAACCACGACGCCGCGGCGCGCGAGCTCCCCGAGCGCGCGGCGCGCGCGCTGCGCGCCGTCGGCCGGCGCGCGCCCGCGGCCTCGGGGCCGCGGCCCGGCCTCTTCCGCGCCTTCTGCGGGTCCCTGGCCTACTGGCCGGAGCTGCGCGTCCTCAGCGGGCACCCCGAGGACGTCCGCTTCGCGGCCTTCCACCTGGCGGTGGCCGAGGTGTACCTGCTGGCGCGCGCCCACGGCCAGAACCCGGGCTTCACGGCCGAGGAGCGCGAGCTGCTCGCGCCCATGTTCACGCTGACCGTGCTCGCGGTGCACCACGCGCTGCGCTGGCTCACGACGGCCGTGGCGCGCGCCGTCTCGGACATCCCCGACGACGAGGCCTTCCGCGCCGTGCGCTCGCGCGTGCCGGCGTCGCTGGTGCCGCTGGGCAGCATCGCGCTCTCGGACGCCGAGTACGCCGTGCTGAACGCGACCGAGGTGGCGGCGGCGCGCGACGCCACGGGCCTGGGCCAGGCCGTGTCGCTGGGCTACGCCGCCGCGCGCTCCGCGCTCACGGGCCTCATGCGGGCCCACGCCGGCGGGAGCGACGCCCTCGTCGCCTTCGCGCTGGTGCTGCAGCGCCTGGCGGGCCACGCGAACCTGCTGCTCAACTGCCTGCTCGGCGCCGCCGTCCACGGCGGCCGGCCGGTGCGCGTGTACGAGGCGGCGCTGGACGACTACGCGGAGCTCATGGACGCGCTCGACCCGCTGGTGCGCGCGTGCCCGCTCGCGGAGTTCTGGGAGCAGCGCGACGCCGTCATGCGCCAGCTGCGGCTGACGCCGGCCCCGGGCCCCCCCACGGGCGGCAAGCGGCTCGTCATCGCGCCCGCGCTGCCCTCGGAGGACCTGGACGGGCTCGTCCCGCCCACGGCCGTGCGCCCCGCCCACTTGGGGCCGGACGTCGACCTCGCCGAATACGCCGCGCGCCACCCGGAGCTGGTGGGCGTGCCGGAACCCCGAGCCCATTTATCGGCGCGCCGGCCGCCCCGGGGCCGCTAACCCGTCCCTGCCTCCGCCATGATCCGCCGCGCCCGAGGAACGCGCCGCGCTTCGTGGAAGGATGCCTCGCGCCGCGTCACCGAGGGGCGCACCCGCGCCAGCTGCCTGCTGGCGGCCCCCGGGGAGGTGCTGACGGCGGCCGTGGCGGCCCTGCGCGAGGCGGCCGAGCGCCAGTGCGCGCCCGCGCTCTTCGGCGCCCAGCGCGCCTCGGCCCTGGCGCTCGTGCGCAGCAACTACGCCCCCGAGTCCGTGATCACGGCGTGCTCGGCGGAGCCGCACCGCGAGGCGTACGCGCGCGCGGCCGAGGCCGCGCTGAAGGGCATCTCGGCGGACGTGGCCTGGCGCGCGGTGCTGGCCACCTACTGGCGCTACCTGAAGGCCAGCTCGGGCGTGGAGGTGCGCTCGGACGACGAGCGCGACGCCCCCACCGTCATGCTGCTGTGGTCGACGTTCGGCAAGACGCTGTCGAAGCACCCGTTCAAGCACAAGGCCCAGAGCGCCGCGTACGGCGCCACGCGCGCCGCGCTCGCCGAGGCGACCGAGGCCGTGGAGCGGTACGCCTACTTCATGCGCCCGCTCGACCCCATGGTCTCCAGCCCCCAGACGAGCGTGCGCCTGCACGAGATGCTGGCGTACGCGGCCACGCTGTACCGCTGGCTGCTGTGGATGATGGACACGGTCGACGCGCGCGTGCTCCGCCACCTGGGGCGCACGCCGCGGGTGGTGCGCGGCCCGCGCGAGACGATGTCGCCCGAGGCGCTCTTTGGGCGGCACCGCGCCGGCGGGCCCGCCGTGGCCTCGGGCTCGGGCGAGGTGCTCGTGCTGACGAGCCACACGGCGACGGTGTTTGACGCCCTGGAGATCCTCGGCGCCACGTGGGCCGAGACGCCCTGGAAGAGCGGCGTGTGCGGCCTCACGGCCGCCGTCGTGGCCGCCGTGGACCTCGTGACCTTCGTGCACCACCACGCGCAGACCCTCATCAACCTGACGCTCGCCGGCTACGTGTGCTGGCTCGACGACGGGATGGAGGACCCGTACCTGCGCGCGGCGCTGCGCGCGCAGTGCCGCTTCCACCACCTGATCGGCGACCTGGCCCCCACGAGCTCGTCGCACGCCTGGGGCGCCATGGAGCGCGGCACGCTCGCCTGGTTCAACTACGCCATCGCGCGCAGCCTGGCCTCGTACGGCGGGCCCACGGAGCGCTTCGCGCGCGTGCTGGCGACGACGGGCCAGCGGGACATGCGCTTCCTGGCCGTCGCCCCCGCGCAGCCGCCGCTGCCGCCCCTGCCGCGGGCGTCGCGGACGCCCTCGCCGCCGCCCCGCGACCGCGACGCGGAGCCGCCCCACGACTACGTGGACCCCCTCGCCGAGTACCTGAACTGGCCCGTGCCCGCCTCGCCGCCCCCGCTCCCCGAGGGGCCCCCGAGCTCGGACGACGAGCTGGAGGTGGACGGCGGCGGGCGGCGCCCGCTCCGGCGCAGCCGCGACGCCGCCACGTACGTGAACCGCAAGGACATTGCCCGCGCGATGGCGGGGGCGGAGGACGAGGACGAGGATGAGGAGGAGGACGGCCGGTACCCGCGCCGCGACGGGGACGCCACGGCCTCGACGAGCTCGTCGCAGGAGAGCGCCGCCGACGAGGAGTTCCCGCCCGGGATCGGCGTGCGCGACGGCGAGGAGGACGACGAGGACGAGGGGGTGGACGAGGAGGAGGAGGAGGAGGACGTGTACGTCGGCCCCGACGGTACCGGCGAACGGGGCGCCGCCGGCGGCGGCAACGCCGCGGACGACGACGACGGCGCCCCCTGGGCCCCGCTGACGCGCCACGGGAGCATGCGCACCAGCTTCCGGCGCGGGGTCCGCGCGGCCCAGCGCTTCGTGCGCCGCCGGCTCTCGCGCACGAGCGCCGAGGCGGCCCCGCGGGCCTCCGGCGACTCAGCCTCCGCGGCGGCCCCCGCCGCCGCCTCCGCCCGCGGCGAGACCGACCACGTGTACCAGCACCCCCGCCCGCGGACCCGCGCGGACGACGGCCTGTACCAGCAACCCCGACCCGTCATCGACCTCACCGGCCACCGCGCGTCGCGCCGCAAGAGCTGGCGCGTGTGACGGCTCCCACGGACTCACCGTTGTATTGTTTTCCCCCGTTGCCCCCCTGTGTGGAAATAAAGTTTTTTTCTAATTCTGTACACAAGGCGTGTGTGTCGTGGCGGTCGTGGTCGCGGGACGGGAGGGCGGCGGGGAGAGGGGAGACGAGAGGGGGAGAGGGGAGACGAGAGGGGAGAGGGGAGACGAGAGGGGAGAGGGGAGACGAGAGGGGAGAGGGGAGACGAGAGGGGAGAGGGGAGACGAGAGGGGAGAGGGGAGACGAGAGGGGAGAGGGGAGACGAGAGGGGAGAGGGACGAGGCGGACGCGAGACGTGCGATCAACGGCATCTTTATTTTTTTCCATCTGCGGGGAGGGGGCTAGGGGGCGTCGGGGTCCTCGTTCTCGAGGCGCTGGTAGTGCCGGCGGCGCGTGGCCATCGCCCCGACGCGGCTGGCCAGCAGCGCGGGCCCGCTGTTCTTCTTGCGCGCCTTGTGCTCCTGCTGCTCGAGGGCCGACACGATGGACATGTACCGGATCATGTCCCGGGCCTGGTCCAGCTTGGCCTCGTCCACGTCGTCCTCTTCGACGCCGTCCTCCTTGAGCGCCTTCGTCGTGACGGGGTACAGGGCCTTCATGGGGTTGCGGCGCAGGCGCGAGATGTGCCGGTAGGCCAGGAAGGCCGCGACCAGGCCGGCCAGCACCAGCAGCCCGATGGCGAGCGCCCCGAAGGGGTTGGACAGGAAGGACACCATGCCGCCGACGGCCGAGATCACGGCCCCCGTGGCGCCCAGGACCACCTTGCCGACGGCGGCGCCCACGTCGCCGAGGCCCTGGAAGAAGTTGGCGATGCCGCGCAGCAGCACCACGTTGTGGTCCACCTTGACCACGCGGTCAATGTCGTAGAACTTGAGCGCGTGCAGCTGGTTGCGGCGCTGGATCTCGCTGTAGTCCAGGAGGCCCGTGTCGGCGAGCTCCTCGCGCGTGTACACCTCGAGGGGCAGGAACTCGCGGTCCTCGAGCAGCGTCAGGTTCAGGGTCACCCGCGTGCTGATCGTCTCGGGCACCTCCACCATGCGCACGTAGCTGTAGTCCTCGTAGTACACGTACCCGCCGCCCAGCTTAAAGTAGCGCCGGTGGTTGCCGGTGCAGGGCTCGATGAGGTCGCGCGAGATGAGGAGCTCGTTGTCGTCGCCGAGCTGGCCCTCGATCACGCCCGTGCCGTTGTGCTCGAAGGTCACCAGCGGGCGGCTGTAGCACGTGCCGCGCTCGCCGGGCACGCGCATGGAGTTCTGCACGTACACGCCGCCGCGCACCTCCACGCACCGCGAGATGGCCATCACGTCGCCGAGCATGCGCGCCGAGACGCGCTGGCCCAGCGCGGCCGTGGCCACGGCGCTGGGGTTCAGGCGCGACATCTCGCCCCACAGGGTGCGGTCCTTGTTCTGCAGCTCGCACCAGGCGGCCGCGATGCGGCTCAGCATGTCGTTCACGTGCGCCTGGATGTGGTCGTAGGTGAACTGCAGGCGCGCAAACTCGGCCGAGCCCGTGGTGATGCGCAGGTGCCCCGTGCCGTTGACGGCCGGCGGCTCGGGCGTCCCCGCCGGGCCGGGGGAGCGCCGGGCCCGACGGGCGGCCGCGGGGGACGCGGGGCCCACGACGCCGGCGAGGCCGAGGCGCTCGAGCTCGCGCGCGTACAGCTGCGCCAGCTCGTTCGAGATCAGCGGGCGGAAGGCCACCACGAAGCCCCCGCGGGCGAGGTACACCTCGGGCTTGTCGCCGGCCAGCACGTGCGTGTTGTTGTAGCGCCGCCGGTAGATGGCGTCGATGGCCTCCGAGGCCTCGCGGAGGACGCAGTCGCCCAGGTGCACGCGCTGCAGGTCGAGCTGCGTGACGTCGCTGACGAAGGAGGCGCCCAGGGCCCGCGACGTGAAGCGGAAGGACCCGTCGCGCGTCTCGTCGCGGATCATCTCCTCGGCCTCGCGCCACTTGGCCAGGCTGCACACGCGCCGCGTCTTGGGGGCCCAGTCCCAGGCCACCGTGAAGTGCGGCGTGCGCAGAAAGTTGCGCGTCACGCTCTCGGAGGCGCGGAGGCGCGAGTCCAGGTCGATGGGGTAGTAGTGCTCCACCTGCTGGAAGCGCCCGGGCGCGTAGCCGATGTGCTCCCCGTGGGCCCCCTCGCGCAGGCCGTAGAAGGGGGACATGTACACGATGTCCCCCGTGGACAGGGCGAAGGAGTCGTAGGGGTACACGGAGCGCGCCTCCACCTCCTCGACGATGCAGTTGACGGAGGTGCCCGTGTGGTAGAAGCCCGCGGCGCCGATCTTGGTGTAGGTGTCGTTGGTGGTGTGCCAGCCGCGGGTGCCGAGCGCGTTCAGGCGCGAGGGGCGCAGGTCCACCTCGACGGGGTTCTCGTCGCGGTCGAAGGCGGTCACCTTGTGGTTGTTGCGCACGTACTCGGCCTTGGAGACGCACTTGCCGCGGCGGTCGATCACGTCCGTGATCTCCTGCACGGGGACGGGCACGCGGTCCGTGAAGCGGTTCGTGATGGCCGCGTACGTGCTCCCGGACCACACGGTCGTGACGATGACGTTCTTGTAGTAGATGTGGGCCTTGAACTTGTGCGGGGCGATGTTCTCCTTGAAGAGCACGGCGATCCCCTCCGTGAAGTTGCGCCCCTGCGAGTACTCGGGGCAGGCCTGCTCGGGCTCCAGGCGCACCACCGTGGAGCCGGACGGCGGCGGGCAGACGTAGAAGCGGTCCCGCTCGGTCGCGGCCGCGCGCACGGCCGTGCGCGCGTCCAGGTCGCCGTACTCGCCGTCGGGGGCGTCCGAGGGGCCGGGGGAGACGGCCCCGTCGATCTCCTCGAGGGACTCCTCCGCGGAGAAGCCGTCTGGGGTGGCGCCCGTCCCGGGCGCGGGCGAGGCCGAGGCGGCCCGCGTCACGGCCGCCGCGCCGCACGTCGGGGTCGCGGCGAGCGCCAGCAGCAGCAGCGCTAGCGCGACGGCGCCCCGCGCAGCTGCAGCGTGGTGTGGAGCAGGCCAAAGACGTCCGAGGCCAGCACCGCCGTGGTGCCCGGGCCGATGCCCGCGGGGCCCGCGCCAAAGACCGCCACCAGCGGGCATGACGCCTCGTAGGTCAGGTACAGCCCGTCGCGTCCGGCGAAGCGATCCGCGCGGAGGACCTCGACGTCCCCGCAGTGGAAGACGGACCTGAAGACTGCAACCGCCAGCACCAGCTCGCGGATATATCGCCAGGCCTGCCGCTGGGTGGCGGTTACGCCGGACGTCTGGAAGCGGTAGAAGCCGCGGAACTCGCTGACGCACCAATCGCGGGCCACCATGAAGCGCGCCAGCTCCTCCTTCAGGTGCGGCAGGAGGCCCACGTTCTCCACCGCAAAGTACAGCGCCGTGTTGGGGGGCTGGGCGAAGGCGTGCGTGTCGTGCGCGAAGAGCGGCCCGTTGACGAGCTCGAAGAACTTGTGCGTGAGCGCCGGGAGGAGCGCGGCGTCCACGGGGTGGCGCTGCACCGTGGCCTTCATGAAGCTGTGCGCGTCGAAGCAGCCCGTCTCGCGCTCGTCCACGACGGTGCCCGCGCGCGCCACGGCCTCGCAGAACGCGCGCCGCGCGCGGAACCCGGCGGCCACCGCCACGTACGTGTGCAGCAGCGCGTCGCCGTACACGTTCACGCGCAGGGTCTTCTCCAGCTCGCGCCGCTGCTCGCGCACGCACTGCGCCAGGCTCGCCGCCGAGCGCCGCGAGAGGCGGTCCGCGTACAGGCGCCGCCGGCGCTCGGCGTCGCGCAGGGCCGCGGCCGGCAGGTCCGCCCACGCCACGGCGTCCGCCGGCGGGGGCGCGCCGGGCCCGTCCTCGCCGCCACCGCCCCCCGCGCCGCCGCCGGCGTCCGCGCCCCCCGCGGCGCCGCCCTCGGGGTCCGCGTCGTCGCCGCCCTCGAGGAGGCGCTCGAGGGCGTCGCGGTTGGCCCGGTCCGGGTCCAGCATGCGCAGCATGGGCGTGGACATGTGGTGGTCGTAGCACGCGCGGATGAGCGCCTCGAGGCGCTCGTCGGGCGAGGCCGCACAGCCGCCCACCAGCAGCCCGTCGATGATGTCGAGCTCGCGGGCCGCGCGGGCGCGGTCAAAGTGCTCGGGGCGCCGCCCGAAGAGCGCGAGCTCCACGGCCGCCGCGCGCACCGCGGCGCGGCGCTCGCGCCGCTCCAGGTCGTCCAGGTTGTGCGTGAACAGGTCGAGGGCCCGCACCGCCTGGTTAGTGGACGCCAGCCAGAACTGCAGCTCGCTCACGGCGTACAGGCGCCGCGAGGCGGGCCGGAACACGTCGTGCGCGTCCAGGAGCGCGTCCGCGCGCCGGCGCGCCGTCTCGCCCGCGTCCCCGGCCTCGGCGCCCGCCGCCGCCGCCTCCGCCTCCCGGGCCTCGGCCGCGTCCACGGCGGCGAGCGCCGCCAGCGCGCGCCCGCGGCGCGCCTCGTCGAGCCCGCGCACGTGCGGCAGGTTCTTGGCCACGTCCTCGGGGTCCACGCGCACCGCCAGCTGCCGCGTGAGGTGGTCGCACACGCAGCCGAGCAGGCGCCGGTGCGTGGCCTCGCCCTGGTTGGCCGTCACGCACAGCTCCTCGAAGCACACCGCGCACGGCTGCGCCGGGTCGTACAGCTCCGGCGGCACCACGAGCCCGCCGCCGATCGTGCGCGCGAGGAACTCGTCCACGGCCGACAGGTCCACCGCGTGGGGCGTGATCAGGTGGCAGTAGTTGAGCTGCTTGAGCAGGTTCTCGGCGTCGTGGAGGAACTGCAGCTCCGTCTCCACGCGCCCGCCGTACGTGTCGAGCGCGACGCGCGCGTGGAAGCGGCACGCGCCGCCCATGGTGCGCTCAAAGTAGGCCTCGGCGTCGTCGCCGGCGGCGGCCAGCGCCCCCAGCACGCGGTCCCCCTCGGCCCGCGCGTACGCGAGCGCCAGGCGCAGCGCGCACGTGAGCGGCGTCATCTGCGCCTCCAGCGGCAGCCGCCGCGCGAGGTAGCGGCACATCAGCGCGTTCAGCTTGACGCGCGGGGCCAGCTCGCGCACCACCGCGGGGTCGCAGCGCTTCAGCATCTCCAGCTGAAACACGTACGTCTGCACCTGCCCCAGGACCGCCACGAGCCGCCGCTCCGCCATGGCCGAGTCCGCCCGCTCGCTCCCTCCCTCCGCCCGCCCGTCCGCCGCGAGAGAGGCCACGGGACGCGCGTGTGTGTCTGTCAAACGCGGTCATCTTTATTGATACAGCCGGGCACCCGCGCCCGTCCCCTCCTCCTCCTCCCCCCATCACAGCATGTCGAAGGTCAGCCGCTTCTCGGGCGGGGCGCCCATGTCAAACAGGTCGTCCTCCGGGGGCGGGCGCTTGCCGCCCAGCTCCGCGGGCGCCAGCGAGGCCGGCGCGAGCCCCGCGCCCGCGTCCCCGCCCGCGCCGCAGGCCCCAAAGTCGAAGGCGGTCTCCTCGCCGCCGCCGTCCGCGCCCGTCTGGCCCTCGAGGTCGCGCACCAGCTCGGCGGCCGCCTCCACGCTCCAGCGCCCGTCGCGGTCGGCCACGCGCCCGTTGATCTCCGCCAGCGCGGCCGCCAGGAACTCGTCGTCCACGATCGCCCGCCAGTCGTCGGGCTCCAGGTGCTGCGTGCGCGCCCCGAGCGCGTGCAGCACGGCCGCGTACACGGCCGTCTGCACCAGCGGCCCGCCGTCGGCCAGGATGGTCTTGACGTGCTCCCCGAGCGAGTGGTCGTGCAGCCCGGCGCCGCCGCCCGTCTGCGAGCACGTGAAGCCCACGCGCGGGCAGGCCAGCACAAAGTGCCGCGTGCGGTCAAACACCATCAGGGGGCACACGTGCTTGCCGCCGTTGAGCCCGCTCCAGTTCCCCGCCTGGAAGACGCGGTTGTTGCCGGCCATGCCGTGGTACTTGCTGACGCCCAGGCCCAGCACCACCAGCGGGCGCGAGGCCATGACGGAGCGGAGCAGGTGGCTGGACGTGTACGTGGCCACCCACGTGTCGTCGCGCGGCGCGTCCAGCACCTCGCGCGCGGCGCGCTCCGCGTCGGCCGCGTCCGCCAGCGGGCGCCGCACCCAGGCCATGACGGCGGCCGGGTCCCGCGGGCGCTTCGACTGCATCGTGATCCCCGTGAGGCTGTTGATGAAGAACTGCTTGTGGTCGCAGTAGCGCAGGATCAGGTTGGCCAGGTAGAACTGGGCCAGCTCCGCCACGGTGCCCGGGGTCAGGTTCACGTAGTTGATGGCGCCGTAGTCCACCGAGAAGCGCTTCACGGCCGCGATGGTCTCGATGTCGTCCTTGGTCAGCAGCCGCGCCGGCAGCTGGTTGCGCTGCAGCAGCGTCCAGAACCACTGCGGGTTGGGGCTCTGCCCGCCGGGCGGCTTGCCGCGCGGGAACAGCGTCGCGTGGTGCTGCTTCAGCAGGAAGCCCAGCGGGCCGTTGAGCACGTCCACGGCCGTGTCCGGGCGCTGGTAGGCGCCCGCGAGCCCGGCCACGCGGGCGCGCGCGGCGTCCGAGAGGCTGCCCGAGCCCGTCGAGAACATGACCCGGCTCTTGACGCGCAGCTCCCGCAGCACCTCGAAGCTCACGCGCGCCAGGTCCCCGTCGTGGTCGCGCGCCGGGGGCTCCGCGCTGCCCTTGGTGGGGTCCGGGGCCACGATGCCGTCGGCGAGCGTCACCGTCACCGTCTTGGCCGTGACGAAGCCCCCGTTGAGCATGTCCACCACGCGGCGGCGCAGCACCGGCTGGAACTGGTTGCGGAAGTTGCGCCCCTCCACGGGCTGCCCGTGGAGCACGCCGTGGCACTGGCTCAGGGCCAGGTCCTGCAGCACCGCCAGCAGCGTGCGCCGCGCCAGGAAGCTCGTCGCCGGGCACAGCGCGCCCGAGTACGGGTCCAGCGAGAGGGACATGGTGTGGTTGGCGTCGTAGAGCGCGTCGCGGAGCTTGAAGTCGCGCGTCTCCACGAGCGTGCGCACGAAGCGGTCCGCCGCCTGCTCGACCGTGTCGCGCAGGGCGCGCAGCGCGTGCCGGAAGCCGGCGTGGTCCGTCACCGTCTCGCCGATGCGCGCGCCGGCCACGTCCGCCAGCAGCCGGTCCACGGCGGCGCGGTACGTGTCCTGCATGATCGCCTTCGGGGGCTCGCTGTCGTTGGGGCGCTTGAGGGCCCCGTACGAGGCGTAGTTGCCGAGCACGTCGCAGTCGCTGTAGGCGCTGTTCATGGTCCCGAAGACGCCCATGGGGCTGCGCGTCGGCGCGCCGAAGCGCGGGAGGCGGTGGCGCAGGCGGTGCAGCGTCGTGTGCGCGCAGGCGGGGCGCGAGGCGCGGTCGCAGAGCGAGCAGGGGACCTCGGCGTCGAGGCTGCCGGCCACGTAGCGCACGGCGTCCACGTCGCCGCGGCCCACGAACGTGCCCGCGTCGCAGCGCTCCAGGTAGAACAGCACGCGCGCCAGCAGCTGCGGGCAGAAGCCGCACGCCATCACCAGGTGGTCCATGGCGAAGTCGTGGTTGCCCGGGGCCGTCGCGGGCTGCGAGGCCGTGTGCGGCAGCACGCGCCCGTCCTTGTCCGTCTGCGGGTTCCCGGCCAGGTAGGGCGCCGCCACCTGGTAGAAGCGGTTAAACGAGGGGCCGGCGCCCTCCTTGCCGCCGCCGTCGGCGGCGCCCGCGTCGTCCACCTCCGTCAGGTACAGCACCGAGTTGGAGCTGAACACCAGCGCCCCCACGAGGCCGGCCGCGCGGCTCACGTACGCGCCGAGCGCCGCGGCCTGCGAGACGGGCGCGCCCGCGGAGGCGGCCGCGCCGCCGCCGCCGTCCTTGGCGCCCGCCTCGGAGAGCACGGGCCAGTCGTCCAGCGCGGGGGGCTCCTCGTCGTACACGCCGGCGGCGACGAGCGCCTCCAGCGACAGCGCCGCGTCGGCGCTCATGACGGACGCCATGCGGCGCTCGAGGCCGCCGGCGGCCGCGTCGCCGCCGCGCCGCCGCTGCTCAAAGAGCGTGAAGGTCACGTCGGCGGGCAGCACGGCGCTCTCGTGGTTCTCGTCGAAGGCCAGGTGCGCCGCGCCGCGCGCCACCGCGTCCAGGTTCCGCACGCGCGTGGCCACGGCCGCCGGGCCCAGCACGTAGCCGTGCAGCAGGCGGCAGAGCGGGCCGTTGAAGAAGGGGCGCGGGTACACAAACTCCTCGCTGATGGAGCGGTTCTTGGCGTTGAAGGGGTCGGCCACGAGCCGGTTGACGTCCGGCATGAAGAGGTGCACGGGGTAGAGCGGCACGCGCCACGCCTCGTGCCCGTTGATGGACACCGTGCCCGCGCCCCCGTAGTGCAGGAAGGCGTTGCACAGGTACACCGCCTCCTTGAAGCCGTCGGCCACCACGAGGTAGGCCGCGTGCGCGTCCGCGGCCAGGCCGAGCCGCGCGCACACCTCCGCGCCCGTCGTCTCCTCGGCGTTGTCCACCGGCGTCCGGTAGGCCGAGAACCCGAAGCGGGCGCGCGCCGCCTCGCAGGCGCGCGAGAGGGCGGGCGCGGCGCTGCTCGGGGGCACGCAGTCCCCGTTGTGGAAGAAGAACACGTTGGGGTGGAAGTGGTTGGGCGTCAGCTTCAGCGTGAGCCCGCCGCCGAGCCCGGTGGTCCGGGCGCCCGCCACCACGGCCACGTGCCCGGCGAAGCCGGCCTCGACGGTCAGGCCGCGCACCAGCGGCGCCACCGCCGCGGCGTCGTCGGCGCTGCGCGCCACGAGCAGCGCCAGCAGGTCGCGGCGCAGCGCCTCGATCGGCGTCACGTACACGTAGCCCAGCGGCGCGGCGCGCACGGTCACGGTCTTGGCCGCGGCCTCCATCGTCGCGGGTGAGACGAGACGAGAGCGGGAGCGGGAGCGGGAGCGGGAGAGAGAGAAGGCGCGGCGGACGGGGTCGAGTCTCGGTCCCCTCTCCGGGCGGACGGGTCCGGCGGCGGGGTGAAGAGAGAGACGGAGGTGTCGGCGCCGAGGGGCGAACGCGACGTCGGCGGCGGTGGTAGAGCCCGGCCCGCCCCGGCCGGGTCTTAAAGCGTGGGGCCCCGCCCCGCCGGGGCGTGGCGCCCCGCCCCGGAGCCTATACCTCGGCCGGCGGCGTTCGCACCTCCGCGTGGGACGAGCGATGGCGGCGCGGCAGGGCAGCTACGTGACGCGCCTGAGCGAGTTCAAGTTCATCGCGCCGCGGTGCCTGGACGCCCCGGACCAGCGCGGCGTGCACGTGGGCACGCTCGCGCGCGAGCCCACGGTCTACTGCGGCGGCGCCACGCGGCCCATCCTCCGCGGGGAGCCCTTCTGGCCGCGGCGCGTCGCCGCGTGGGACGGGGCCCCCGAGCGGCCCGTGAGCCCGCGCTTCGAGCGCTTCCACGTGTACGACATCGTCGAGAGCACCGAGTACGCCAGCGCGGACCCGCGCTTCCCCAACGGCACGGTGGTCACGCTGCTGGGGCTCAGCGCCTGCGGCAAGCGCGTGGCCGTGCACGTGTACGGCGTGCGCCACTACTTCTTCCTGGGCAAGGCCGAGGCCGACGCCGCCCTCGGCGTGACGAGCGCCGAGCAGCTCGCGCGCGCGCTCTCGGCGGCGGCGGCGCGCGGCCCGCGCCTCGGGCCCGCCGACGTGGACGCGCGCGTCGTCGACGCCGCGCCCGTCTACTACTACGACGCCCCCCGGCGGCCCTTCTACCGCGTCTCGAGCAACAGCGGGCGCCTGGTCGCGCACCTCCGCGAGACGGTCTGCGCCGGCCTGGTGACGCACGAGGCCGGCGTGGACGCGACCACGCGCCTGCTGCTCGACCACGACCTGCCCAGCTTCGGCTGGTACCGCCTGCGGCCCGGCCCCGCGGGGGAGCGCGTGGTGCTGCGGCACCAGCGCCTCACGTCCAGCGACGTGGAGGTCAACTGCACGCCCCTCAACCTGGCCCGCGACGAGGACGGCCCGTGGCCCGACTACAAGCTCCTGTGCTTCGACATCGAGTGCAAGGCCGGCGACGACGCGGCCTTCCCCGCCGCCGAGAACCCCGAGGACCTGGTGATCCAGATCTCGTGCCTGCTGTACTCGCTCGCCACCCAGCGGCTGGAGCACACGCTGCTCTTCTCGCTGGGCTCGTGCGACTCGGACGACCCCGCGGTGACGGTCCTCGAGTTCGACAGCGAGTTCGAGCTGCTGCTCGCCTTCGTGACCTTCCTGAAGCAGTACGCGCCCGAGTTCGCCACGGGGTACAACATCATCAACTTTGACTGGGCCTTCGTGCACACCAAGCTCACGGCCGTCTACGGGCTGGCCCTCGACGGCTACGGGCGCTTCAACCGCGGCGGCCAGTTCCGCGTCTACGACGCGGGCCAGAACAGCTTCCAGAAGCGCAGCAAGGTCAAGATCAACGGCCTCGTCTCCCTGGACATGTACGCCGTCGCCGCCGACAAGCTGAAGCTGCCGAGCTACAAGCTCAACGCCGTGGCCGAGGAGGCGCTCGGCGAGCGCAAGCTGGACCTGGACTACAAGGACATCCCGCGGTACTACGCCGCGGGCCCGCGCGAGCGCGGCGTCATCGGGCGCTACTGCGTGCAGGACTCGGCGCTCGTGGGCAAGCTCTTCTTCAAGTTCCTGCCGCACCTCGAGCTCTCGGCCGTGGCGCGGCTCGCCAACATCACGCTCGCGCGCGCCATCTACGACGGCCAGCAGATCCGCGTGTTCACGTGCCTGCTGAAGCTCGCGGGCTCGCGCGGCTTCGTGCTGCCCGACAAGCGCCGCGACATCGCGGACGAGGACGACGGCGGCGGCTACCAGGGCGCCAAGGTGCTCGAGCCCGACTCGGGCTTCCACGTGGACCCGGTGCTCGTGCTGGACTTTGCCAGCCTGTACCCGAGCATCATCCAGGCGCACAACCTGTGCTTCACGACGCTCGCGCTCGCGCGCCCGGCGGGCCTGCGCGAGGACGAGTTCAGCGCCTTCGAGGTCAACGGGGAGCGGCTCTACTTTGTGCACGCGGGGGTGCGCGAGAGCCTGCTCTCGATCCTGCTGCGCGACTGGTTGGCCATGCGCAAGGCCATCCGCGCGCGCATCCCGACGAGCGCGCCCGAGGAGGCCGTGCTGCTGGACAAGCAGCAGGCGGCCATCAAGGTGGTGTGCAACTCGGTGTACGGGTTCACGGGCGTGGCCAACGGGCTGCTCCCCTGCCTCCCCGTGGCGGCCACCGTGACGACCATCGGCCGCGACATGCTGGTGGCCACGCGCGACTACGTGCAGACGCGCTGGGCCACGCGCGAGCTGCTCGAGCGCGACCTGCCCGCGCGCCCGCCCGCGGGCGAGTACGCCGTGCGCGTGGTCTACGGGGACACGGACTCGGTCTTCATCCGCTTCTCGGGCATCGCGTACGACGACGTGTGCGAGCTCGGGGAGCTCATGGCCGCGCGCATCACGCGCGACCTCTTCCGCCCCCCCATCAAGCTCGAGTGCGAGAAGACCTTCCGGCGCCTGCTGCTCATCACGAAGAAGAAGTACATCGGCGTCATCAACGGCGGCAAGATGCTCATGAAGGGCGTCGACCTGGTGCGCAAGAACAACTGCGCCTTCATCAACGCGTACGCGCGCCGCCTGGTGGACCTGCTCTTTGGCGACGAGGCCGTCTCGGCGGCCGCGGCCGCGATCGCGCGCGCGCCCGCGGCGCGCTGGCTGGAGCGCCCCCTGCCGCGCGGCTTCGCCGCCTTCGGGCGCGTGCTGGCCGAGGCGCACGCGCGCGTCGCCGGCGGCGCCGGCCTGGACGTGGCCGACTTCGTCATGACGGCCGAGCTCAGCCGCCCCCCGGACGCGTACAGCAACACGCGCCTGCCGCACCTCACCGTCTACCACAAGCTGGCCATGCGCCACGGGGAGGTGCCGAGCGTCAAGGAGCGCGTGTCCTACGTCATCATCGCGCCCACCCCCGAGGCCGAGCGCGACGCGCGGGCCGTGCGCCCGGGCCTCGCCCCGGGGAAGCTGCTCGTCAGCGACCTGGCCGAGGACCCCGCCTACGTGGTCGCGCACGGCGTGCCCCTCAACACCGAGTACTACTTCTCCCACCTGCTGAGCACGCTCAGCGTCACCTTCAAGGCCCTCTTTGGAAATGACACCAAGATCACGGAGAGGTTGCTCAAGCGCTTTATTCCGGAAACCGCCCCGGGGGACGCGCCCTTCGAGCACGAGGCCTTCGCGCCGCTCACGGGCGAGGGGGGCGAAAGTCTTCAAACGCTGCGTACAATCTTTTGTACTCCAACAGCAGCTCCCCGTCGAAGCTGATGTCCCGCATCTTGCAATAAATGTCGCTCGCGCTGACGGCGGGCACGTCGTCCGCGGGTCTCCCGTCTCCCTTCTTTCGCACCACCAACACGAACATGGTCTGCCACACGTGCGCGACGATGCGGTGGCCGGCGCAGGCCCCGAGCAGGCGGTCCAGCACGCGGTGGTGCACGTGCACCGACTTCTCGGGGAACACCACGTACATCATGAGCGCGCCCTGCGCGTCCGGGTCCTCGAAGAAGAGCACGCGCACGTCGCGCAGGCCCCCGTCGCGCAGCACGTAGTAGGCGAAAAAGAGCTCCGGGTGCGCGAGCACCTCCTCGAGCGCGCGCTCCGAGGGGTCCCGCGCGGCCACCGAGGCCAGGAACACGCGGTACTCGTAGATGCTGTTGAGCTGCTGCACGTAGGCCAGCACGAGCGCCGCGCGGTCGCTGCGCCCGAGCCGCGGCTCGGCGGCGGCGCACGTCGGGCAGCAGCCGCCGAGGCCCAGGTAGTAGCCCATGCCCGAGAGCGACAGGCAGTTGTCGGCCACCGCCTGGCCGAGGTCGAAGGGCAGCGTCACGGGGGCCGTCTTGATGAGCGGGTTCGAGAGGCCGCGCACGGTGGCCACCTCGTCCGAGGGCTGCGCGGCCGCGTAGGCAAAGTAGGGCGAGTAGCGATCGCGGACCGCGCGCGCCAGCGTCTTGCGCCGCGCGGCCGCCGACTTGCGCCGCAGGAGTCGCCGCCGCTCAAACATAGCTGCCGAGCGTGTGCTCGAGGGAGACGAGCGCGAGGCCGTGGCGCCGCAGCAGGGCGTGTGCGTCGCGCAGGAAGGCGGCGCGGGCAGTGGGCTTCGGCGGGAACAGCTGGTACGCTTTAAATACGTACGCCAGGGTCCACAGCTCCCCGCACACGCGCCCCTCGAAGCGGTTCTCGGCGGCGATCTGCGCCTTGCGCAACGCGTCGCCGCGCTCAAAGAGCACGCGCGGGTCCGCCTGGCGCTCGGCCACGGCGCACAGGGGGTCGCAGAAGAGGTGCTTGTAGACGGCGCGCGGGCCCGCGGCGCGCATCAGCGCCAGGAAGCGCGCGTCCGCCGCGAAGCCCTCGAGCACGGGCTCGATGCAGTCAAAGAGGCCCGCGTTGTTCTCCGAGTAGCTGAGCACGCGGCGCCGGAAGCGGCGCAGCGCCAGCCAGTGCGCGCGCGTGAGCAGCATGTTGCAGAGCAGGCACTCGCCGTGCGTGCGGTTGCGGCCGCGCACGTGCTTCAGCGCCAGCGCCAGCACGGGCCCCAGCGTGGCGTCCACCGCGCGGTAGCGCGCGTACGCCGCGGCGGTGTTGCGGGCCAGGAAGCCCTCGTGCTCCGCCCAGTACTCGTCCACGGCGCGGTGCCGGCGCTCCTCGGCGGCGTCCGTCTGCGCGTTGGGCTCCCCCGTGGCCACGTTGCCCAGCAGCAGCATGGCGAGGTCGGCGTAGCCGGGCCCGCCGGCGTCCGCGTCCTCGGCCCCGCCCCCGCCCCCGGCGGCGGCGACGGCGGCGCGCTCGTACGCGTCGTCCTCGCGCTCCGCCGCCGCGCAGCACCCGGCGCACGCCTCGTCGCCCGTGTGGTCCTTCCAGCCCCCCAGGCTCGGCGGCGCGCGCGCGTCCGCCACGGTGAAAAACATCGCGCGCGTCGCGGCCTGGACCAGAAAGGACTGGTTCGAGAAGCGGATCTTGTCGCCGACGGTGCGGAAGCAGCCGTGCAGGAAAAAGTGCCGCTGCACGTCCACGAGCGTGAGCACGCGCGTGGCGAAGGCGCGCTGCAGCGACGCGCGCCCGCGAAAGTGCTTGTCCATCACGTACACGAACTCGAAGGCGGCCACGAAGACGGCCGTGGCGCACGGGGGGCAGGCCAGGGCCTTGGCGCACAGCGCCGCGTAGTCCGCCAGCCAGGGCGGGGCCAGCGAGCGCTCGCGGCGGTACACGTCGATCGTGCGACACACGCGGCAGGGCTCGTCCAGCGCCAGCGGCGCCGCGGCGGCCTCCACGAAGCCCGCGGGCGAGTCCTCCTCGTCCAGCATCACGTGGGCGGCGAAGATCAGCTCGCGGAAGAGGGCGTCGTTCTTGGTCAGCAGCGCGGGGTCGAAGGCCGTGTACGGCGCCTCGAAGGCGTCCTCGCTCCAGCCCATGGCGTGCGACGGCGGCGGCAGCGGCGGCGGCGCGGTCGATCTGCGCGCGGCGATCCCGGAGGCCGCGCTGCGCGACTTCGACGTGGACTTTCTCGAGGCCAACTACCTGCCCGCGCGGGTGCGCGTCTGGTTCGAGGACGTGATGCCGCGCGAGCTGGAAGTGATCCTCCCCACGACGGACGCCAAGCTAAATTACCTGGCCCACACGCAGCGCCTGGCGGCGGCGACGAGCGAGCGCGACTGTGCCCACGGGGAGGCGCTGCGGGCGCGGCGCGATCGCTTCGCCGCGGCCGTCAACAAGTTTCTGGACCTGCACCAGGTCCTGCGCGACGGGGTGGAGCTGGGCGCCCTATAAAGTCCGCCGCCCGGCCCCGGGAGCTCCACCAGCCCCACCACCACCGCCACTGCCACCGCAGCCCGCCCGCCCGCCGGGAAGACCGCCGCCGTCGCCGCGTCGACGTCGACGTCTTAGCCGCGCCGCTCTCCCTCGCCCTCCCGGCCCACGGCGACGACAACGACGACGACCATGAGCGGCACCCTGATCCAGCGCCTGAAGCTCATCCTCTCCGGGGGGAACCTGCGCTGCAGCGACGGCGAGACGGCCTGCGACCCCGAGCGGCCCCCGACGCGGTGCGTCTTCCAGGTCCACGGGCAGGACGGCTCCAACGACACCTTCCCGCTGGAGTACGTGCTGCGCCTCATGCGCAGCTGGGCCCACGTCCCCTGCGACCCCTACGTGCGCGTGCAGAACACGGGCGTGTCGGTGCTCTTCCAGGGCTTCTTCTTCCGGCCCGCCGACGCCCCCCTGGCCGCGATCACGGCCGAGCACAACAACGTGATCTTGGCCTCGACGCACAGCACCGGCATGAGCCTCTCGGCGCTCGACGACATCAAGCGCGCCGGGGGCGTGGACATGCGGCCGCTGCGCGCCATGATGTCGGTGAGCTGCTTCGTGCGCATGCCGCGCGTGCAGCTCTCGTTCCGCTTCATGGGCCCCGACGACGCCTCGCAGATGCAGCGCCTCCTGGACCGCGCCGAGCTGCGGCAGCGCTCGGTCTCCCGCCCCGGCGGTGGCGACGACGGCTGCTCGGGCGAGGGCCCCTCGCCCCGCGCGCCCATCCGCCCACCCGTCCTCTCGCCCGTGCCCGGACACGCGGCCGCCGCCCTCGTGGGCCAGGCCGCGTACCCTCCGCCCACGCGGTTCCCGGCCTCGCTGCTGCACGCCCTCCTGGGGCTGCGGCGCCTCGCCGGCTACGCCGTGGCGTGCGTCACCGGCGCTCTCGCGATAGTCATCATCCTGAACATGCGTTAAAGGCGGCCGCGCGCGCGGGGGCGCGCACAGACGCGCGCTCCCCGCCGAGCCATCATGTCCTTCGACCCGAACAATCCCCGGACGATCACCGCGCAGACGCTCGAGGGCGCCCTGCCCGTGGACATTCTGCTCCGGCTCAACCGCGCGACGGGGCTGCATATGGATGCGGCCGAGGCGCACGCGATCGTGGAGGACGCCCGCCGGACCCTGTTTATCGGCACCTCCCTGGCCCTCGTGAACCTGCGGCACGCGCACGACAAGCACCTCGTGGAGCGCCAGCCCATGTTCGCCACCAGCGACTACAGCAGCTGGGCCAGGCCCACGGTCGGCCTGAAGCGGACCTTCTGCCCTCGCCCCTCGCCCTAGCCCCGCGCGATCAATAAAGCGAGCCGCGTTGCACCGTCCTCTCGTCTCTCGTGTGGTGTCTGGGTGGGGTTTCCCCCGAGATCCCCCGATCTCCCCGCCGGGTCTTTGGGTCCCCCACCGAGTTCCCCACCGAGTTCCCCACCGAGTTCCCCACCGAGTTCCCCAAATGCCCACCACCACCAAGTCTTTGGGTCCCACAACCCCCAAGTTTTGGGGTCTCGGGTCCGAGTCCCCCGAGTCCCCCGAGTCCCCCGAGTCCCCCGAGTCCCCCGAGTCCCCCGAGTCCCCCGAGTCCCCCGAGTCCCCCGAGTCCCCGAGTCCCCCGAGTCCCCCGAGTCCCCCGAGTCCCCGAGTCCCCGAGTCCCCCGAGTCCCCCGAGTCCCCCGAGTCCCCCGAGTCCCCCGAGTCCCCGAGTCCCCCGAGTCCCCCGAGTCCCCCGAGTCCCCGAGTCCCCCGAGTCCCCCGAGTCCCCCGAGTCCCCCGAGTCCCCCGAGTCCCCCGAGTCCCCCGAGTCCCCCGAGTCCCCGAGTCCCCCGAACACACCACCGCAGAGACAAACAGGTTGGGTAATAAACAATTTATTAACCAAGAATCAGGCGGACCTGAAATAGGTCCACGTTGAACCGGCGGGCGCTGCCCTTGAGGTTCGCCCGCGTCTCCCGCAGGCGGCGCGCGATCAGGCGGCACGCGGCGATGAGCAGGGCCAGGGCCCCAAGTCCGGTGGCGCGCATGTACCGCCTGTTGATGAGAGTGTCCGACTCCACCACGCGCGGGGGGAGCAGCTCCTGGGCCAGAGAGACGTCCTCGTCCTCGTCGTCCTCATCGTCCTCGAACTCCGCGCCCCAGGGGCCCGGCTCGGGCGCCTCCTCGGCGGGCTCGCGGGTCAGGTCCGCCCGGGTGGGCCAGACGCGGCCGAGGCCGCCCAGGGGAGAGTCCGGCGGTGGGGGTCTCTGCTGCTGCCGCGGCGGCGGAGGAGGGTGGGCGGGAGGGACGAGCGCGGCCAGGTCGTCGAACCACGGCGGGGGCTGGTGGCGGTCCGCCAGCGGGGAGCGCGGGGCCGGCGGCGCAACCTCCGGCGGGAGCTCGGGGACGGTGGTGGGCGCCTCAGACAGCGGCGGCCAGGGCAGTCGCTCCAGCGGCGAGCGCGGCGGGGTCGCCGGGGCCTCGGCCTCTTCTCCCTCGCTCGCCCATAGCGGCTGAGGGGGCGGCGGCAGCGGTGATGGCGGCAGCGGCGGCGCCTCTCCTCCGGGGAACGGGAACGGGAACGGCTGGTCCGAGGGCGGCGGGGTGAAGAGCGCGGGTGGGCCGGCGGGGTCCCTCGGCTCGGGCCACGACCACGGCGCCTCCGGGGGGCGCTGCTGAGACGGCTGCGGCGGCAGCGGCGGAGGTGTGAACAGGAGCCTAGGGGGCTCCGCGGGCTTCTCGGGCTCCGCCGGCGCCGGTCTCGTCTGCGCCGGGGCCCGGGCGGGCTCGGCCCTCGGCGGCGCCGGGGCCGGCCGCGAAGATGGTGGTGGTGGTGGCGGCGGCGGCGGCTGGGGTGGTGGTGGAGCGGCGGCGGAGGAGGCCGAGGAGGAGATGGTGGTGGCGGCGGTGGAGGAGGAGGTGGTGGTGGCGGTGGCGGTGGTGGTAGAGGCCGGTGCTGTTGTTGCTGTTGTTGTTGGAGCTGAGACGGCGGCGGGGGGCCGCGCGCTCTCGGGGGCGGCGGCCAGCGACGTCGGGGCCGCCAGCGGAGACCGCGGCGGCTGGGTCTGCGCCGGGGCCGGAGTCTCCGGCGAGGCCGGCAGCGGCACCGCGACGCCCTCCAGCGGCACAGACTGCGGCGGCGGGATGTGGAGACGGAGGTGCCCGGCCGGCGGGCGCGCGACGGCGGCGGCTCGCCTCACTGGTCCTCCGGGGGCTGGGACCACATCTCCGAGCCCGACTCCGAGGGCGGGGACGGCTGCTGCTGCTGTTGCTGCTGCCGCCGCCGGTGGGCCCGCTGGAGCAGTCGCCGGCGCTTGGGCTTGGGCTTGAGGCGGGCGCGGTTCAGCTCCCCCGGGAGGACGGCCCTCGGGTTGAGCCGCGGGCGGAAGACTCCCGGTCTGGCGCGAGGCTGATACGGGCCTTCGCGGAACAAGAGCCATGGATTTTCGGCGACTGTGGTCTCTAATACCCACCGACGTTCGCGCCTTGGTGCGTAGAACAGCGGAGGGAAGGGGCTGCGCCACTGGGGCCGCGGGGAGATGTCCCGCAGGTTGATCTCCACGGCTCGCATCCGAAGCCGGGCCACCGTCTCCTCGGCGCTGGCCACCACTCCCGGGGGCTCCAGGAGGTGCTCGGGCCGCTGCTCCGGCTGCTGGGGGGGAACCTCCGCGGGAGCCATCGCTCGGTGTTCCGGCCGGGGAAGAAGAGGAGGACGAGGCGGCTTCTCGGGACGGAGTCGAGGCGGTGGAGGAGGACGCGGACGAGAGCTCCGAGTCCGATGTCCCGGGAGGAGAGCGGGTCCGACGCTGGTCTGGTCCGACCGTGTGTTCGCACTTGTCTGCGCACCCCGCTTTATAGCGTTCGTCATCGAGGGGGCGGCCCGGGGAGGCGGTCCCCGCCACCCTCTCACCAAATTTGGTGTCTCGGACGTCCGGCGGGGCATCGGCCCTCGGCGCTACGATGCGGTCACCGTCGGGTTTCTGGGCCGATGGGGCGGGCCCGGGCGCGGGGGGCCGGAAGGTCGCCGTCGGGGCAGATGGGGGTGGCGGGGCGGGCGGGGCGGCGGGCCCGGGGGTCGTCGTCGGGGCAGATGGGGGCGGCGGCTGTGGGGGAGCGGGCGGGGCGGGGGGTGCCGGGGGCGCGGGCGGGGCGCCCCGGGGCTTGGCGGCCGGCTGGGACGATGGGGGCTGAGCGGCGGCGGCGGCGACGGTGGTGGTCGCGGCGGGCTGTGGCGGGGGACCCTGTGACGGAGGTTGGGGCCGGGGCTGGGGCTGGGTCTGGGGGATGGGGGCCTTGGCCGCGGCGGGCTGCGACGGTGGGGCCTGGGTCGGGGGAGCCGGGGCCGACGGGGGAGCCTGAGTCTGGGGCTGGGGTTGGGGGGTCGGCTTCGCCGTCGCGGCGGGCTGCGGCGGGGGCGCCTGGGCCGGGGGAGCCGGGGCCTGGGGGGTCTGCTCCGCCGGCGCGGCGGGCTGGGTCTGGGGGGCCTTCTCGGGCTTCGGGGGCGCCGCGGGGTAAGGCGGGTTGATGATCGGCGGCGGGGGCTCGACGCGCGCCTTCAGCGAGGGCTGGATCTTTCGCGGCGACGAGTGCGGCTTGGGGGTGGGCTCCTGGGGCCGCTGCTTGGGGGCCGCGGCGGCCTTGGCGGCCTTCTTGGAGGCGGTGGCGGCGGGGGGCTCCTGCCGCTGCTGCTGCTGCTGTTGTTGCTGCTGCGGCGGCGGCGGTTGCGATGGCGCCTTGGGGGCGGCGGGGGGCTCCTGCTGCCGCTGCGGCGGCGGCTGCTGCGGCTGCGGCGGCGGCGGCGCCCTGGTGAGCGGGGTCGCCTCCACGGGCGCGGCGGGCGGCGGCGCCGGCGGGGCCAGCTTGACGAGCGGGGTCGTCTCCGCGGGCGCGGCGGGCGGCGGCGCCGGCTTGGCGGCGGGGGCCGCCTCCGCGGGGGGAGCCGGTGGCGGCGGCGCCGGGGGGGCCAGCGGCGGGAGCGCGGGCGGGTCGAACGGCGGCGACGGGGGCAGCGGCAGCGCGCTGGCCACCAGGTCGTCGCCGAGCAGGTCCAGCCACGGGTTGGCGTCAAAGTCGTCGTCCGGGACGAACCCGTCGTCCTCCTCGGCCAGCTCGGGCGGCAGCCCGGGGATCGGGGTCGCGGGCCGGGTGGGCGTCGCCGACACGGCGTACGGGAACGGGTCGCCCAGGTTGCGGAAGCTCGGCCGGCCGTACAGCTCCTCCGCGGTGGCCGGGTCCACGCGGGCGTCGCCCATGATGATGGAGGTGAACAGCGGGTCCCGGGGGTCCATCCCCGGCAGCTGGTCCTCCGTCGTCTCCAGGAAGGGGATCTCCCGCAGCAGCGGGTCGTCCTCGAGCGTGAAGCCCACGCGCCGCGGCGGCTCGGGCGGCGCCCAGAGCACGCGCGACACCGCCAGCTGCGAGTCCACCAGCACGAGGCAGGCCGGCGTGCCGGCCAGGGGCCGCGCCGCGATCAGCGCCGAGAGCCGCTCCAGCTGCGGCGTCAGGCACGCGTTCTCGATGGGGTTGTCGTCGAGCGCCAGCAGGCGCTCGCCCCAGGAGCTGAGGTCCGTGGACACGCCGCGGTGCAGCGCGGGGTCGCGCAGGTCGCACAGCGTCACGGCCAGGGTCAGGCCCGAGCCGGGCGAGTACACGTCCAGGTTCTCCATGGCGATCACCAGCGGGGCCCCGACGAGCACCGCCGCCGCGGCCAGGTCCATGGCGCTCACGCGCGTCGCGCTGCTCGGCGGCGAGCCCGTGACGGTGAACGTCATGCCCGTGCCCACGGGCGCGTACAGGTCGCCGGGCGCCGGCGCGCCGAGCGCCGCGGCCTCGCGCACGTGCGCCGCGGCGTCCAGGCGCTCCGTGACCGCGGCGTCGTACGCGGCGTTCGCCTCCGGCTGCAGGGCGGACCACAGCGCCGAGAGCAGCCGCGGGGGGATGCACATGCGCGCCAGCACGGTGAAGGTGGCCAGCGCGCCCGGCACGGCGCGCGCCAGCCGCTGGCCCGTCTCCGTGTGCGCCCAGGGCGCCCACAGGTCCGTCTCCGAGAAGCGGCCCTCGGTCCAGTCGGCGACGCGCAGCGTGAACAGCGCCTGCCCGTCGCGCAGCGGGCGCCGCGGCGGGCGCGCGCCCGCGTCCAGCGTGTCCACCGCGTCCAGCGCGCGGTCCAGCACCGCGTCCAGCGTCTTGGCCATGTACTCGTGCTGCCCGGCCAGGTCCACGCGCGTAAAGTTGAACAGGTGCGCCGGCTCGCCGGCCACCGCGGCCACCATCAGGTCGGGGAGGGCGAGCCGCACCGCCGGCTCCAGCGGCGGCTGCACGACGCGGTCCAGCGCCCCGAAGGTGCGCGAGAGCGTCGTGGCCAGCACGGCCGCGTACGTCTCCGCGGCGGCCTTGGCCGCCGAGTCCCAGTCGGCGCGGCGGTGGCGCACGAAGCGCGCGAAGGTGGCGAAGCCCGTGTCGCGGGCCTTCTCGTAGTTGAGCCGCAGGTGGATGATCTCGTTCATGAGGCGCATGCCCGCCACGGTCGCCGTCTCCATGAGCCCCTCGGTGAGCGGCGGGCGCAGCTCGGCGCCCTCGTCGGTGCACAGCATGGCCGCCAGCTTGTCGCCGACCGCGCGGTAGCACACCTGGTACTCCACGGGCACGTTGTTGTCGTCCAGGAAGACCGTGGGGAACACCGCGTCCGCGGCGCTGCGCGCCTCGCGCACCGTGCAGATGACGTCGCGGTCGCCCAGGCGCGCCGCCACGCGCTGCTTCAGGCGCGCGCACGGCGCCGTCGAGGCGCGCGGCCGCGCCGGCGTCGGCCCCGGGCCGCCGTAGGCCGCGTAGAACTGCAGCCGCAGCGTGAGCAGCTCGCGGTGCGCGGCGTACTTGGGCGGGACCCAGCGCGGCAGCCGCTCCGCGCGCGCGTCGAAGGCGGCCGCCAGCGCGCGCAGGGCGTCGTGCTGCAGCTCGGGCCCGATGCGCGCCGCCATGCGGTCGAGCTCGCGCATGTCCTCCTCGACGGCGCCGGCGTCGCGGAAGAAGTCGCCCAGGGCCCCGGCGCGCGCCGAGTACTTGCTCTCGATGCGCCCCACGAACTTGGCGGGCAGGCGCGGGTAGACGGCGTCGCTGCGCAGCGCCAGCACCTCCATGGTCGCCGTCTGCAGCGCGCGCGCGCGGGCCTCGGCCAGGCGCGCCTCGTCCTCGGAGGCGCCGGCGCGGCCGCGCGCGCGCCCAAAGTCCTCCCAGGCCTCGTCCCAGGCGACCTCGGCGGCCGTCAGCCGCTGCCGCAGCGTGTCCGCCTCCTGGCGCAGCGTCTGCAGCGCGTCGATGCGCTCGGCGAACTCGTCCATGGGCCCGCGGCCGTCGACGCGCGTCGTCAGCGGGTGCGTGTCGATGATGAGCCGCGCCTGGTGCAGCCACTCGACGGCCTGCACGTCCAGCTCGGGCGCCTCCTCGACGGCGCCGAGCAGCAGCGCCGCCTGCGTCACCAGCTCCTCCGCGGAGGCGGCCCGGTCGATGTTGAGCGAGGCCGGCGGCGGCGTCACCCGCAGCAGGTTCTTGAGGTTGGCCAGCTCGAGGGCGCCGTCGCGGTCGCGCGCCGCCGCCGCGTCCACCACCTCGCGCAGCAGGCGCGTGGCGCGCTCCGTGGCCGCGGCCTGGTCCGCCTCCGCCGCGGCGAGGCCGTCGCGCGCCGCCGCCAGGTCGCGGCGCACGAGCGCGCGCATGGCCTCCGCGTACGCCGTGCGCTCGAAGCGCGCAAAGTCGAAGCGCTCCAGGTAGGTCACGGCGCCGCGCAGCAGCTCGGTGCTCGGCACGTGCAGGTGCACGCCCTCGTCGAGCGCGCGCGCCGCGTCGGCGGTCGCGCGCACGTACGTCACCTCCTCGAGCGCGCGGCAGATCTCGGCGCCGCGGCTGCCGCCGGCCTGCAGCACGTCCGCGCGGCGCGCGCCCAGGGCGGCCAGCTCGGCCTCGTACTCGGGGAAGCCGCGGCGGTAAAAGTCCACGTACTTGGCCAGGCGCGGCACGTGCGCCATGTCCGCCCCGACGAAGCCCACGGCGTCGTAGTAGCTGAAGCGCCCCATGCCGGCGGCGGCGCGGGCCAGCACGCCGCCGGCGATGCGCGCCAGCCGCGAGAGCGGCTCCGTGTCGGCGCCGAAGAGCGAGCCGATGACGGGCGCCGCGACGGCGTAGTCGTCGAGCCAGGTCAGGCGCTGCAGCGCCTGCAGCGGCGGGCCCGCCGCCGCGCCGGCGTTCTCGGGCAGGTGCGGGTTGAAGGCGAACACGGCGTCCAGCGCCGTGGTCACGGTCTCGGCGTTCGCGGCGAGCGCGCGCTCGGCGGCGGGGCGCAGCTCCGCGGCGTCGTAGCCGCGGTCCGCCGCCACGTCGCGCAGGCGCACGAACTCGGCCGCGTCGAAGGCGTGCTGCTCGGCGCGGCGCAGCGCCGCGTCGGCGGCGCGCGCCCAGAGCTGGCGCTGCGCGCGCTCCACCTCCGCCGCGCGGCGCGCCTCCACGGCCGCCACGGCCGCCTCCAGCTCGCCGACGCGCTGGCGCGCCGCGAGCGCCATCTTCTGCAGCTGCGGCTCCTCCACGGCGGCCGCGCGCGCCACCTCCAGCGCCAGGTGCCGCGCCTCGACCAGCGTCCCCTCGTCGGGCTGCGCGCGCAGCCGCCCGAGCATCTCCACGGCCGCCGCCACGGCCGCCGACAGCGCGGCCTGCAGCGCCGCCACGCGCTCAGACTCGCGCAGCGCCGCGGCGCGCTCCTCGCACTGCCGCGCCAGCGGCTCCAGGAAGCCCAGCTCCGGGTGCTCCACGGCGGCGCGGAGCACCCCCGCGGCGCGCGCCGCCTTCTCCGCCGTGTCCGCGTCCGCCAGCGCCGCCGCCACCGCGTCGGCGTGCGCGAGGAAGAAGCGCGTGATCGGCGGGTTGTCCGCGAGCGCGTTGACCAGCGCGCCGAGCGGCTGCCCGGCGCCCACCAGCGCGCCGGGGTCGGAGGTCAGCAGCTCGCGGTAGCGCGCCACCAGCCGCCACAGCGCCGCGACCACCGTGCCGGGCACGGGCGTGGCCGTGATGTGCGCGAGCAGGTCCACCGCCGGGCCCAGCGTGGCAAAGTCGCCGAGCAGCGCGCGCAGCCGCATGTAGAGGTCGTTCTGCTCGGCGGCGCGCTGCTGCGCCGCCTGCACCAGCGTGCGCAGCTCGGCGTCGCGCCGCGCGAGCCGCGCGCGCGTCTCCTGGCTCAGCACGTGGCCGCTCATGCCGCGGATGTGCCGCAGCAGGTCCTCCATCGCGCGCACGCGCGTCTCCTCGTCCGGGGCGGCCGCCAGGGCGGCGTCCATGGCGTCGAAGCGCGCCAGGTCGGCGTCGGCCGAGGCGCGCTTGGCCGCGCGCTCGTTGATCTGGTTCACGTCGCGCGTGAGCGTGTCCAGCTCGCGCCGCTCCAGATGCCCGTGCGCCTGCGCCTCGGTCAGCAGCGCGAACCAGGCCCCGAGGCGCTCGTCCGTGCTCACGTCCTCGCCCGCCTCCAGCAGCTGCGCCAGCAGCGCGCCGTCGTGGGCGGCGGGCGCCGCGTCCAGCGCGCGCAGCCGCTCCTCGGTGGCCGCCAGCGAGTCGACGAGGCGGCGCAGGCCCGCCACCGCGGCGTTGGCCACGCGGAAGCGCTGCGCGCCGTTGCGGTCCATCAGCATGGCCTTGGCGCTGTACTGCGCGCCGCGCAGAAAGTACTCGCGGATGGCGTCGCCGACCATCAGCGCCAGCTTGCGCGGCAGCTTGGCCGCGCGCTCCTTGACGTCGCGCGTGTTCGGCTCGGCCGCGCCGGGCGGCGCCCCCGCGTCCACGGCGTCAAAGTTGGCGTGCGCGGCCTCGAGCGCCGCCTCGAGCGCGCTCACGTCGCGCGCGCGCTCGAGCGCGCGCTGCGACTCGCGGGACTCGCGCGCCCGCGCGCGCGCAAACAGGCCCCGCGCGCGCGACACCACCGTCTGGTCGTAGACGCCGCCGGGGTCCCGCGCCGCGGCCTCCATGAAGCACGCCGAGAGCTGCGCGTAGGAGCCCCCGGACTCGAGCGCGTCCAGCGCCCGGTGCATGGCCTCGTTCGCGGGCCGCGCGACGCGCGCCGCCACCAGCGCCAGCTTGGCCACGGGCGCGGACGCCTCGCCGCCGGCCGCGTCCAGCAGCGCGCGCGCCTCCGTCAGCACCATGTGCGTGGCGGCCACAAAGTCCCCGGCCGGGCCGCGCTCCGGCAGCGCGGCCAGGACGGGCTCGAAGAGCGCCGCGACGGCGCACGGCGCCTCCGCGTGGGTGCGGGCGCCGTTCTCCACCAGAAAGTCCTGCAGGTGCCCGAAGAGGAGCTCGGCGAAGCGCTCCAGCAGCCCGGCGCGCCCGGGGAACAGGCTCCGGCCCGCGCACGCGACCACGTGCTCCTCGAGCTCGTCGGCGTGGGCCTGCAGCGGGCCCAGGTCGAGCGGGACCAGCTCCAGCTCCCGCACGGGGAAGCCCATGGGCTCGCGGGTGCGCGGGCGGTACTTGCGCGACGGCCGCCCGCCGACCGTGTGCGTGCTGCTCAGGTCCTCCTGGCTCGCCGGCCACGGGGGCCGGCGCCGCTTGGGCATGCTCGGCCGCTTCTGGACCCGCGGCGGCGCGGCGAGGGACGAGGGCTGGGGCTGGGACTGGGGCACCGCCGTCGAGAGGGGGGCGGCGGGGACGGGGGCGACGGCGAGGACGGGGATGGTCCCGACGGCGGCGACGGCGGGGGCGCCCCCGGGGCCGCGCGGCGGCGGCGCGGCGGCCGCCGGGTCCGGGGGCAGGGCCATCGGGTGCACGGAGGGCACCGCCCGCTCCACGTACGGCTCGTCCACGAGGTACGTCTCGCCCACGCCGTAGAGCGCGGGCACGGCGGCGAACTGGTCGTGGGGCAGGAAGAACACGAAGGCCCCGGCCCAGCGGAGCTCGCTGGCGGCGTAGCCCGCGACGTGCGCGTACACGTCCGCCGGGCGCAGGCGCGCCACGAAGGCCTGCGTGATCTGCCCGTGGCCGTGCGGGTCGAAGACGTAGACGACGTCGCCGCGGCGGTAGAGGCCCAGGCCCACGGCGCCGACCACGACCAGCGTGAAGGACTCGGCGCGCTGCGCCCAGAGGCCGTCGAAGAAGGCGCGCGCCGTGATCTGCGTGCTCTGGAAGCCCTCGCCGGGCGGCGTGAAGAAGTTGCACTCGCCGTGCACCCGCGAGAAGACGCAGTGCAGCGCGGCGCCGCCGGCGCCCTCGTAGACGATCTTGTTCGGGAGCTCGCTGATGGCGCACATGCCCCCGTCGGGCCCCGTCCACGCCTGGCCCTCCCGCAGGCACGCGTCCACGGCGTCCGCCGTCAGGGCGGCCTCCACGCCGTTCGTGAAGACCAGGCGCAGGAAGGAGAGGGAGGAGCGCAGGCACGAGACGCCCGACCCGGGCCCCAGGTCGGGGTCATACTGATTACGATAGCCGACGACCACCGCGTCGGCCGTCATGGCTGAAATAACACACGCGCGTGGGGGAAAAATCTTTTTATTTGGACACGGCGTCGACCTCGGCCAGGAGCCGATCCACGGGCACCCACGTTTGCGCGAGCAGGGGCACGCCCGCGCCCGCGTCGTCGGGCGCGCGCGCGCCGCGGCGCAGCACCTCCAGGTTCACGGCGGCCCAGTCGCGCACGTTGACGCTCGCCGAGAGCGGCACGGCGTCGGGGCCCGCGTCGCCGTCGGTCGCCACCTCGTGGTAGTCGCCCAGGACGCGGTCCGCGGCCTCGCGCAGCTCCCCGGGCGTGAAGGCGGCCGCGGGCGGCGCCTGCTCCTCGCGCGCCTCGGTCCAGGTCTCGCGGAGCGTCGCGGCGAGCGCCAGCACGCCGCGCTCGTCGACGGCGCGGCGCAGCGCGGCGCTGACCAGGTCCCAGCGCGCCAGGAAGCGCTCCACGGGCCGCAGCACGGTGGCGGCCTGCAGGCGGCCGGCCGTGAGGGCGGCGCTCGTCTGCAGCTTGCTCAGGCGCGCGTGGTCGGCGCACAGCGTCCGGAACACGGGCGCCTGCAGGCCCAGCGCCTCGCACTCGCGCAGCGCCTCCGTCGTCTGCGCCATCACGCCGCGCAGCGCCCCCGCGTCGGCGCGCATGTCCGAGCGGTGCGTGTTAACCTCGGCGTGGAAGGCGCCCACGGCGTCCACGACGTCCGCCAGGCGCCACGTGCCCAGGCAGGGCCCGCCCTCGCCGGGCGCGAAGACGGAGGCCGTGCGCCCGCGGAAGACGAAGCGGCAGGTGAAGAGGCGCTCGAGCGCCACCACGCGCTCGGCCACGGCGCCCGCGGCCGTGGCCATCGAGTCCAGCAGGTCGCCCGTGTAGGTGGCCAGGTCCTCCAGGCGGCGCCGGAGGGCGCGCGCCATCAGGTCCCCGAGGAAGGCGCTGGTGGCCAGCGCGGGCGGCGCGGGCACCGGCGCCTCGAGCACCTCGGCGGCCGCGCGCACGACCCCGTGCAGCTCGTGCGCGAGCGCCTCGACGTTGCGCGCGTGCGCCACCGTCAGGTCCTCGCGCGCCAGCGCCGCCACGAGCGCGGCGAGCTTGGTGCGGTAGCCGCTGACGTTGGGGATGAGGCCGGCGCCGTAGTCGCGCGCCCACGTCAGCACCTGCGCCAGCTCCGCGCGGCGGTCGTAGGAGGCCGTCAGGAGGCAGTTGTAGGCGTCGGCCACGGCCGTCACGACGAAGAAGGGCGCGAAGGACGGGTCGGCCATGGCCTCGAGGTGCACCATGGACGGGTAGTTCACGAGGTCCGCGGCGGCGGCGAGGTCGACGAGCGCCGCGCTCGTCGGCGCCTCCATGGGCGTCTCGCCGGCGCCGTCGGCGCGCAGGCCCCAGCGGCTCTGCGCGCGGAAGCCGAGCCACGTGCCGAGGGCCTCGCGCGCCGCGCTGTGCGCCAGCCCGGCGCGGCGCTCGGCCACCGCCACCTCGCGCATCAGCGCGGCGCGCGCGGCGCCCACGTGCTCCGTGGGCGGGCGCGGCGCCATGCAGTGGCGCACCACGTGCTCCACGCCCTTCTCGGGGAGCCCCAGCGCCGCCGTCACGTCCCCGTAGAGGGGCTCCAGCGTCTCCGCGACGTCCGTGTAGGCGCGCACGTAGTCCAGCCCGGGCCCGTAGTCGTCGAGGAGGCGGTGCACGACGCCGCCGACGACCGCCGTGCAGCCGACGGCGCGCTGCGCGTCCGTCAGGCGGGGCGCGCGGTCCAGCACGGCGCGCTCGAGGAGGTCGCAGTGGCGGCGCGTGAAGCCCGCGGTCACGAGCGCCTCCACGACCTGCAGCGGCGAGGCGCTGGCCTGCACCGCCTCCCACACGCCCGTGGCGTCCACCTGCGCGTGGTCGAGGTCCGCGGCGGCGGCCACGTGCACGGCCTGCGCCCCGGGGTCCAGCAGCGCCAGCACGCCCTGGTCCGTGCGCAGGCGCCGCGCCACGGTGCCCGGGCGCAGCTCGCGCACGTACGCGTCCCCGTGCGCGAGGGCCGCGGACACGCGCACCTCGACGGCCGTGCGCGGCGTGGCGTTGACCTCGTCCACGGGCACCAGCGCCGCGTCGTCCACGGCCCGCGCCGCCTCGGCGAACTGCTCGCGCAGCGTCGGGTTCTGGCGGTCGTGGAAGAACTCGTCGGCGATCGTGAGGGCAAAGAGCGCGCGCGTCAGCGGCTTCAGGCCCTCGTTCTGCATCTGGCGCACCGCCAGCGCCAGCGGCAGCGCCCAGTTGGTGCGCCGCGCCACGTGGCGCAGGCCGTCCTGCACAAAGTCCATGTCGTAGAGCGTGGCGAAGCGGGGCTGCACGAGCGCGCGCGCCGTCTGCTCCGGGGGCACGACGCGGGGCGCGGCCAGGACGTCGTCGGCGGAGCGCGCGCCCGCCAGGGCCTCGTCGAAGGCGGCGCACCACTCGCTCACGAGGCGGAACATGGCGTCGGGGCCGAACTCGGCCGAGGTGCGCAGCCCGGCCTCGGTGAGCGCGGCCGTGGTGGCGTCGGCGTTGACGACCCCCAGGAGCTCCAGCAGCGGGGCCGTGCCCTCGTCCCAGTCGTGGCGCAGGCGCCACAGCGCCAGGCCGGCCAGGTTCTCGGCGAGCACGGCCGCCTCGCAGGTGCCGGTCGCGGCGTAGGCGCGGCAGGCCAGGCGCAGCGCGCGCGCCCAGAGCCCGCGCACCCCCGCGGGCGTCTCGCGGCCCGGGGCCATGAAGAACTCCAGGATGTGCGACTGCACCACCGTGGCGACGGCATGGTCGGCCTCCTCGAGCGCGCGCACGAGCGCCTCCATTATAAGAGGGTCCGCGCGCGGGCGCCGCCGCGCACTCGACCCCGCGCGGCGCGACGATGAGCGTGCAGATCGGCAACGGGCTGCTGATGGTGGTCGCGCCGGGCACACTAACCGTGGGCTCGGCGCGCGCGCGCCTTATACGCCAGGTGACGCTGGCGGACTTTTGCGAGCCCCAGGCCGAGCGCCCCGGGCTGGTGGTGCTGGCGCTGCGGCACCCCGCGGACCTGGCCGGCGCCGCCTACGCGGCCACGCCGCCCGGCAAGAACCACCGCGACCTGGAGGAGGCGTGGCTGGCCCTCGACGAGGGCGGGCGCGGCCTCGGCGGCGACGGCATCCGCGCCTCCGTCGTCTCGCTCAACTTTTTGGTGGCGGCCGCCGAGAACGGGGACGACGCGCTGCGCGCCCACGTGACGACCAACTACCGCGACCGGCGCACGGCCGCGCGCCTCGAGCGCTTCGCGGCCGTGCTGCGCGCCATGATCCGGAGCCACGTGTTCCCGCACCGCGCGCTGCACGTCCTCGGAGGGCTCCTGGGCCACGTGACGCAGGACCGGCTGGCCAGCGTCACGTGCGTGGCGCGCGGCGACCAGGAGGCGGCGCGCACCAACGACATGGCCGCGCGCCGCTCGCAGGTGCACGTGCCCGCGTGCGCGCTGATGGACGTGGACCGCGAGCTGCGCCTCGGCGGCGACGACGGGCTCCGCTTCGTGTACCTGGTCTTCGTCTACACGCAGCGCCACCGCCGCGAGGCGCTGCGCGTCCACGTGGCGGTGAGCCGCCTGCCCGAGCTCGGCGACGCCCTCAGCTTCCTCCTGGCCGGCACGCGCGTCGACAACGCGATCCACGGCACGGACGAGGCCGACGCCCCCGCCGCGCCCGCCGCCGCCGCGGCCTTCCCCGCGTACCTGTTCAACGACCCGCGCAGCGCGCGCTGCCCGACGGGCCGGCTCAACACGCCCGCCGCCGAGGCGCTGCCCGTGTGGGCCCCCGACATGCGCGGCCGCGCCACCCGGAACTCGTGCATGTACGCCGCCTACGTGCGCCTCGGCACCGTCGAGCGCGTCGTGCGCCGGGCCGAGCGCTGCGGCTCGGTGGACCTGCCGCTGGCCCACATGGAGCGCTTCACCTGGGACGTGGGGGCGTGGGAGGAGTGTTTCTTCTGAAAAAAAACGGGGGGAGGTGTGCGTGAGACGGGTCTGATGTTGCTGACGAGACTAATAAAAAGCGGGGGATACGCGCGCGCGTGTGTCCCCCCGTTGACCCTTCATGCCGCGTCCGCGTCGTGTGTGACTCACCCCCATCGTCTCTCCCGCCCGCGATCCCGGCCCGTCCCGACTCGTCCCGCCCGCCCAGACGCGTCCCGTCACGCCCCCGCGCACGCCCCGCCCGCCGGGCGCGGTTTTCCCCCCTTTCCCCCCATTGGCGGCCTCCGCCGCTAAAAGCCCGCGCGCCCCGAGCTCGGGACTCTCCGCGCACTCCCCGTTTCGAGCACACACCGTCATGGCCTCCCCCGTCGTGCCCGCCGCCGCCGCCGCCGCTCCCGGCGCGGACTCCTTCCTCGACGCCGTCTGCCCCGAGGACGTCGCCCGGGCCCTGGCCGCCGAGCTCGAGGCGCTCCGCGCCCTCGGGCACGACGTCGGCGCGCCCGCGCCCGGGGCCTCGCGTCGCGAGGTGGCGCTGTTTGTCACCCGCGCCGTGGACGGGCTCAAGGCCTTCTCCCGGGTGGACGAGCGCGTGTACGTGGCCTGCGGCAAGCTGGTGCACCTGCGCGTGCGCAGCCGCGAGGCGGACCTGGACGCCTGGCTCGCCTCGCCGGAGCTCGCGCTGATCCCCGCGGTGGCGGGCGCCGTGCGCCGGCACCGCGCGCGCGTGGAGGCGGCCCTCCGCTGGTTCTGGCGGGAGGCCTACCCGGCGCTCTACGCGCGCGGCCTGCAGTCGGCGCTCAAGTACGAGGAGATGTACCTGGCCCGCCTCGAGCACGGGCGCTGCGAGGCCATGGACCAGTTCTTTGTGCGCCTCGCCGCCGCCGCCGCCACGGCGACGCGGCGCGCCATGGCGCTCGTGCTCTGCGGCTCGGACGCGTGGCCGGAGGTCTTTGACGCGTACTTTCGCGCGCTGGCCACGCAGGCGATCGTGCCCGCCACCCCGCTCATGCTCTTCGCCGGCCGCGCGCGCGGCTCGCTCGCCAGCTGCTACCTGCTGAACCCCCTGCCGCGCACCACCGAGGAGGCGGTGCGCGCCATCACGGACGAGGTGGCCCCCATCCTGCTGCGCCGCGGCGGCGTCGGGCTCTCGCTGCAGAGCTTCAACCGCACGCCCTCGGGCGACTGCACGCGCGGCATCATGGCCGTGCTCAAGGCGCTGGACTCGATGACGGCGGCGATCAACAGCGACAGCGAGCGCCCCACGGGCGTGTGCGTGTACGTCGAGCCGTGGCACGCGGACGTGCGCGCCGTGCTGAACATGCGCGGCATGCTCGCCGCCGACGAGAGCCTCCGCTGCGACAACATCTTCAGCTGCCTCTGGACGCCGGACCTGTTCTTCCAGCGCTACCAGCGGCACCTGGACGGCGAGCGCGCCGTCAAGTGGACGCTCTTTGACGACCGCGCCTCGCACCTGACCTCGCTGCACGGGCCCGACTTTGCGCGCGAGTACGAGCGCCTGGAGCGCGCCGGCCTCGGCGTCGAGTCCCTGCCCATCCAGGACATGGCCTTCCTCATCGTCCGCAGCGCCGTCATGACGGGCAGCCCCTTCCTCATGATGAAGGACGCGTGCAACCGGCACTTCCACACGGACACGCGCGGCGCCGCCCTCGCCACGTCCAACCTCTGCACGGAGATCGTGCAGCGGGCCACGCCCGGCGAGAACGGCGTCTGCAACCTGGCCAGCGTGAACCTGCCCGCGTGCCTGGCGGGCGGCGCCTTCGACTTTGCCGCGCTGCGCCGGGCCGCGCGCGTCGCCGCCGTCTTCGTCAACGCCATGATGCGCATCGGGAACTACCCGACGGAGGCCTCGGTCGAGGGCGTGCGGCGCAGCCGCTCGCTGGGCATCGGCCTGCAGGGCCTGCACACGACCGTGCTGGCGCTGGACATGGACATGGCCGACCCGGCGGCGCGGCGCCTCAACGCCGCCATCGCCGAGGAGCTGCTCTACGGCGTCATGGACGCCAGCGTGGAGCTCTGCGAGCGCGGCCTGCGCCCCTTCGACGGCTTCGAGCACAGCCGCTACGCGCGCGGGGTCATGCCCTTCGACGCCTACGAGCGCGTCTCGCTCCGCGAGCCGATGCGCTGGGACGCGCTGCGCGTCCGCATCGCGGAGCACGGCGTGTACAACGCGCAGTTTGTGGCGCTGATGCCCACCGTGTCCTCGTCGCAGGTCACCGAGAGCAGCGAGGGCTTCTCGCCCACCTTCACCAACATGTTCAGCAAGGTCACCATCTCGGGGGAGCTCCTGCGGCCCAACCTGCCGCTCATGGAGACGCTGCGGCGCCTGTTTCCGCGCGAGTGCGCGCGCCGGGACGCCGTGGCGCGGCTGGAGCGCGCGCAGTGGTCCGTGGCCGCGGCCTTCGGGGAGCTGCCCGCCGGGCACCCGCTGGCCAAGTTCAAGACGGCCTTCGAGTACGACCAGGAGCTGCTCATCGACATGTGCGCGGACCGCGCCCCCTTTGTGGACCACAGCCAGTCCATGTCCCTCTTCCTGACCGAGCCCGCCGACGGGAAGTTACACGCCTCCCGCGTCATGGGCCTCCTCATGCGGGCATATAACCTGGGCCTCAAGACGGGCATGTACTACTGCAAGATCAGGAAGGCCACCAACAACGGGGTGTTCACCGGCGGGGACCTGGTCTGCACGAGCTGCCACCTGTGAGCGCGCGCCATGGAGTACTTTTACACGTCCCAGTGCCCCGACATGGACCACCTCCGCTCGCTGAGCGTGGCCAACCGCTGGCTCGAGACGGACCTGCCGCTGGGCGACGACGCCAAGGACGTGGCCGCGCTCAGCGAGGCCGAGCTCGAGTTCTACCGCTTCCTCTTCGCCTTCCTCTCCGCCGCGGACGACCTCGTCAACGTCAACCTGGGCAGCCTCTCGGAGCTCTTCACGCAGAAGGACATCCTGCACTACTACATCGAGCAGGAGTGCATCGAGGTGGTGCACTCGCGCGTGTACAGCGCCATCCAGCTGATGCTGTTCCGCGGCGACGCCGCGGCGCGCGAGCGCTACGTGCGCGCGGCGCTGCGGGACGAGGCCATCCGCCGCAAGGTGGAGTGGCTCGACTCGCGCGTCGCCGAGTGCGCCTCCGTCGCGGAAAAGTACCTGCTCATGATCCTCATCGAGGGCATCTTCTTCGCCTCCTCGTTCGCCTCCATCTCGTACCTCCGCACGCACAACCTGTTTGTGGTGACGTGCCAGTCCAACGACTTCATCAGCCGCGACGAGGCCATCCACACCTCGGCCTCGTGCTGCATCTACAACAACTACCTGGGGGACGCCCCGCGCCCCGACGAGGCCCGCATCCACCAGCTCTTCGCCGAGGCGGTGGAGATCGAGTGCGAGTTTCTGCGGGCGCGCGCCCCGCGCGACAGCCTCCTGCTGGACCTGCCGGCCATCATCTCGTACGTGCGCTACAGCGCGGACCGGCTGCTGCAGGCCATCGGCGCGAGCCCGCTCTTTGGCGCGCCCGCCCCCGCGGCGGACTTTCCCATGGCGCTGATGGTCGCCGAGAAGCACACCAACTTTTTCGAGCGGCGCAGCACCAACTACACGGGGACCGTCGTGAACGACCTGTAGCGCCCAGCGGCGGCGACCCCCGCTCTCCCCCCTCCTCCTCTCTCTGCGACAAAACACTAATAAAGCGTTGAGACACTAGCGCGCGCCTCCGGCCGTCATCCTTGGGGCGAGGGTGGGATGGGGAAGTGGAGGCGGGATGGGAGAGTGTGGGGAGAGTGTGGGGAGAGTGGGGTGGGGAGAGTGCGGGGATGATGACGGGGGAAGGGAGATGGGGCTGACGGGGGTGGACTGGGACTGGGGAAGGATGACGGCGGCCGGCGCAGAGTGGGAGGCGGTGAGGTGGACGGGGGACGGAGGGGAGGACCGATGTGGGACGGCAGAGGGGCGGCAGAGGGGCGGCAGAGGGGCGGCAGAGGGGCGGCAGAGGGGCGGCAGAGGGGCGGCAGAGGGGCGGCAGAGGGGCGGCAGAGGGGCGGCAGAGGGGCGGCAGAGGGACGGCAGAGGGGCGGCAGAGGGACGGCAGAGGGGCGGCAGAGGGGCGGCAGAGGGGCGGCAGAGGGGCGGCGGCGAGGTGAGACGGAGCCCGCGGACGGAGTGGCCGGCGGGGGGAGGCGACGACGGAGAGAGAAGGTGGGGGAGAGGGCGAGCATCACACGGGCCGCACAACCGAGGATTTTTTTCAAGTTGTTTTTTATTTTCTCCTATGGGCGTTACAGTCGTCCCAGTACGTCATGAGGACGGCCTGGTAGTTCGGAGGCGGAGGTATGTGCTTCCAAAACAGGCCCGCCAGCTCCTTCGCCCGCGTCTCGTTCTTCACGTGCCGCAGCAGCGAGCCAAAGACCTTGTTCACGTCCGAGGGCTCCTGCACGATGGGGACGCGCCGCAGCACGCTCAGGTGCCCGTGCCGCGCCGGCGTGAGCATGGCCACCACGTGCTGGATGAACTCGCGCTCGGCGCGGCGCGAGCGCGCGTCCCCGGCCGCCGGCAGCAGCGCGAGCGCCTTGCCGGCGTCGCGCATGATGTCGGGGCAGCGGGACGCGTACTTGAGCTTGACGTCCTCGGGCGCCACCTCGCGGCCCTCCAGCGACTCGGCCACCTGCTGCACCGAGTCCACGTCCGGCGCCCGGTGCAGGTCCGTGTGGCAGCGCACGAACGCGGCCAGGAACTCCGAGTAGTCCACCCCCAGGCTCGCGAGGACGTCGCGGCAGCGCAGCACCAGCGGGAACATGGGCGCGATGTCGAGGATCATGTCGCACCCGGTGAGGATCATGTCCGTGTCGGTCGTGTGCACCTGCGCGACCGTGTTGGTGTGGTAGAGGTTGGCGCAGACGTCGTCCGCCTCCATGTCCGACACGTCCACGTAGGCGTAGCCCATGTGGCGGATGAGGTTGACGCAGAGGCGGTGGACGATGCGCGGCGCGTGCAGCATCGTGCTCCAGCGCGGCCGCGGCGGCGCGTCGGCGGCCCCGTCGCCGTCGGCGCGCGCCGCCGCGGCCATGATGGCCTTGGCGCCGTGCGCGACGCGGCCGTTCCCGAAGATGCCGCGGTCCGAGACGAAGATGGGGAAGTAGGTGCGCTTGTGCAGCATGCGCAGCAGGCGCAGCAGGCAGCGGGCGGTCGTGGTCGCGTTGTCCTCGGTCGTCTCCTGGTAGTGCTTCTCCATGAGGGTGTACATGACGTTCCACAGGTCGATGGCGATGGGCGTGAGGACCCCGGGCGGCGTGGAGATGGCCTCCGAGCGGACCAGACGATGGCGATGTGCGTACTTTAAAAGGCCAAAGAGCCCCATGTCTCCGCTCGCACACCGAGGCGCCGGTCTAAAATACGCAGGTGGCGGGGCGGGGCCACCCGCGCATATAAGCCGGGCGGTGATTGGTCTGGCGCACACCGCCCGCCCGAGCCCGCTCGCCCGCCGCGATGTCGCTGTTCGACGACGGCCTCGAGGACCTGGACCGCCACCCCACCCACGCGCACCACCCGGCGCAGGTGATCCACGACGGGCCCTTCGTGCTGGAGGACGGCGAGCCCCTGCAGCGCACCGGCATGCTGGTGCTCAGCGACGAGCACCTGGAGCACGCGCGCGCCGCCATCGCCCCGCTCGCCGCGCACCTCGCGCACGCCTTCCTCGTCTTCAGCGAGGCCGGGCTGCTGGTGCACGCCAGCGTGCGCGGCGAGCAGGTCTACGTGACCCTGGCCCCGGACCAGTTCAGCACGTTCGTGTGGAGCGGGCCCCAGGCCGTGTTCCTGGGCAACGTCGACGGCAGCGGCGGCGTGCTCGACGCGCTCAAGGTCGACCGGCGGCGGACCGTCTTCAACGTCACCTTCGAGGTGTACGGCGCCTTCCCGGCGCGGCTGCTGACGCGGCGCGCGTACTTTGCGGACGCGGGCCTCCTCGCGGCGGGGCCCGGCTCCCCGAGCGTCGCCTGCGTCTACAAGCACGAGTTCAACGACTACTGCATCATGCTCCCCTCGCGGGCGCCCGACGTGAGCCTGACGCTCTCGCGCCCCCAGGTGGCCAAGCTCGCCGCCGTGGCGAAGGGCGCCGCGGCCGGGACGACCTTCGCGCTCGCCCGCGGCCTCGACTTCTCCGTCTCCTCCAGCGCCGGGGTCGTGACCTTCCCGGCGCGCGACCACGACGGGACCGCCGTGCTGGAGCGCGCCAGCCGGCGGCGCCAGGGCGTCGACGCGGTCGGCGCGACGGAGCCCTTCGCCATGACGCTCGAGGCGGCGCACGGGCTGCTGACGCTGCTGCAGCGGCTGCGGGCCGGGAACGCCGAGCTCACGTTCAACTTTTTCACGACGCCGCGGCAGGCGCCCCTGTTCAGCGTGACCACCTGCGGCCCGGTGCGGGCGACCACCTTCTTCTTCTGCGCGCCCGCCGACCCCGCCACCGTGCCCGCCGCCCCCGAGGGCGCCGCCGCCACCGTCGCCGCCGCCTGCGGGGCGGGCGCGTCCGCCGCCTCCCCCGCCGCGGGGGACAAGCGGCCCGCCGCCCCGCGCATGTACACGCCCATCGCCAAGCGCCCGCGGACCGCCTCGGGGGAAGGGGGCCACGCCTACGGAGATTTATTCTAATAAAGTGAGCAGGTGTATAAAAGAGAGGTCGCACCTCCGCGTCTTTACTCGCCGTCGCGATGATCTCCCGGGGGAACGGAGGGGGTTGCCGCCCCGGCGAGCCATGCTGGCGCTGCGCGCTCGAGTCCACCCGCTGCATCACGCTGATGGGCGTGCTCGTCGCGCTCCTCGCCGCCTGCATGCTGTCCGTCCCGCCCGCGGCGTCGACGATGCTGCTCGGCGTCGCCAGCCTCATGGCCATGCTGCGCCTGCCCATGCCCCTCGTGGACCGGTTCATCCCCGCGTGCATGGGCCTCCAGCTCGTCGGCGCCGCCGTCTTCGCCGCGGGCTGGGCGCTGGCCAGCCGCGACGCGATCTCGGCCGGCGTCCTGCTGTGGGCCGTCTGCGCGCTCATCTCGCACATGTACAACGTCGTCTGCGTCGCCAGCGGGCCCGACGCGCACTACCGCCCCGCCTGCCTCGTCATGGGCGTCGCCGCGGCCTGCGGCGCGGCGGGGGCCCTCGTGAACGTGCGCACCGAGGCGCGCCTCGGCATCGCCCTGGGGCTGGCCGTCACGTGCGCCACGAACAACGTCGCCCGCAGCCTGCGCGGCACGTGCACCTACGTCGCCAGCCGCGCCCGGTTCCTGGCCGCGCCCGCGGACCTGGGGCGCGGCTACAGCGTGGAGAACGCGGACGCCGACCCCACCGCCGAGCCCGAGCGGCGCGTCTACGAGGCGACCGTCCCGCCCACCCACGTCTACGCCGGCAGCATCGCGCTCTTCGCCCTCGTCTTCTCGGCCGCCTCCTCCCTCCAGTGGATGGTCTCCCAGATGGTGGGCCGCGGCAACCAGCTCGTCTCGCCCACCACCGCGGCCGCGGCCGGCGCCGCCGGGTTCCTGGACGCGGCGGCGCTCTCGCTCTTTGTGCGCCCGAGCACGCGCCACCTCTCGGTGGCCGTCAAGGGCGCGCACGCGCTCCTCATCCTCGCGGCCATCGTCCTCACGGCCGCCGGCGAGCCCATGGGCGTGCCCATCAGCCTCGCGGCCTCCACCGGCCTCGCGCTGCTCGAGGCGGACCACGTCCGCCTGCGCCACACCCGCGCGTACCGGCTCGCCGCCGCGCACGTGACGCGGGCCCTGCTCGTGCAGGCGTACGTGACCGTCGCCATGTGTGCCACTAGCATTAAATCCGTTTCCTGATTCACGCCCACGCCCGTGTCGTTTTTAAAACCGCGATGGGGGGACGGGGGGCCATTCGCACGCGCCATGGCCTCGCTCGCGCGTGCGATGCTCGCGCTGCTGGCGCTCTACACGGCGGCCATCGCCGCGGCGCCGTCGTCCACGACGGCGCTCGGCACGACGCCCAACGGGGGCGGGGGCGGCAACAGCAGCGCGGGCGAGCTCTCGCCCTCGCCGCCCTCGACGCCCGAGCCCGTCTCGGGGACGACGGGGGCCGCGGCCTCCACGCCCGCCGCCGTCTCGACGCCCCGGGTCCCGCCGCCCTCGGTCTCGCGCCGGAAGCCCCAGCGGAACGGCAACAGGACGCGCGTCCACGGCGACAAGGCCACCTCGCACGGGCGCAAGCGCATCGTGTGCCGCGAGCGGCTGTTCTCGGCGAGGGTGGGGGACGCGGTCAGCTTCGGGTGCGCCGTCGTCCCGCGCGCCGGGGAGACCTTCGAGGTCCGCTTCTGCCGCCGCGGGCGCTTCCGCTCGCCCGACGCCGACCCCGAGTACTTTGACGAGCCCCCGCGCCCGGAGCTCCCGCGGGAGCGGCTCCTCTTCAGCTCCGCCAACGCCTCCCTCGCCCACGCGGACGCGCTCGCCTCCGCCGTCGTCGTCGAGGGCGAGCGCGCGACCGTCGCCAACGTCTCGGGCGAGGTGTCCGTGCGCGTGGCCGCGGCGGACGCCGAGACCGAGGGCGTCTACACGTGGCGCGTGCTGTCCGCCAACGGCACCGAGGTCCGCAGCGCCAACGTCTCGCTCGTCCTGTACCACCAGCCCGAGTTCGGCCTGAGCGCGCCGCCCGTCCTCTTCGGCGAGCCCTTCCGGGCGGTGTGCGTCGTCCGCGACTACTACCCGCGGCGCAGCGTGCGCCTGCGCTGGTTCGCGGACGAGCACCCGGTGGACGCCGCCTTCGTGACCAACAGCACCGTGGCCGACGAGCTCGGGCGCCGCACGCGCGTCTCCGTGGTGAACGTGACGCGCGCGGACGTCCCGGGCCTCGCGGCCGCGGACGACGCGGACGCGCTCGCGCCGAGCCTGCGCTGCGAGGCCGTGTGGTACCGCGACAGCGTGGCCTCGCAGCGCTTCTCCGAGGCCCTGCGCCCCCACGTCTACCACCCGGCGGCGGTCTCGGTGCGCTTCGTCGAGGGCTTCGCCGTCTGCGACGGCCTCTGCGTGCCCCCGGAGGCGCGCCTCGCCTGGTCCGACCACGCCGCCGACACCGTCTACCACCTCGGCGCCTGCGCCGAGCACCCCGGCCTGCTCAACGTGCGGAGCGCCCGCCCGCTGTCGGACCTCGACGGGCCCGTCGACTACACCTGCCGCCTCGAGGGCATGCCCTCGCAGCTGCCCATCTTCGAGGACACGCAGCGCTACGACGCCTCCCCCACGTCCGTGAGCTGGCCCGTCGTGACCAGCATGATCACCGTCATCGCCGGCATCGCCATCCTAGCCATCGTGCTGGTCATCATGGCGACGTGCGTCTACTACCGCCGGTCCGCGCTGTGACGCCCCCGCCCGCCCCGAATCAATAAACGACCGCGCGTTGAACCGAGCCATCGCGTCCGGTTGTTTGTTTGTGCGTGTGTGTGTGAGCTCTCGCGCGTCCCCGGGCCCCGCTCTCTCCCGTCCCACTCCACTCTCCTCTCCCGCACCCTCCCCACACACACGCCCGGCCCGCAATAAACCACACGAGACGACGGCGTGTTGTACCGGCGCGACCGGGCCATCTCTTTATTGGTCGTCGGGGAACAGGGACCTGGCCGGGGTGGGGGGTGTGGGCGGCGGGCGAGCGACGCCGTCCTCATCGGTGGGACATCATCTGGGAGACGAAGATGTCGGCGCCGCGCTGGCAGGCCACGGCGGCCGAGCTGGCGTCGATGGTGCCGGCGGAGACGTCTGCCGGGGCCGAGACCGAGGCCTGGACGGGCGGCTGGGGGGCCGAGGCGGGCGCGGCCGGGGCGGCGGCCTGAACGGGGGCGGCCGCGGCGGCCGCCGCTGCTGGAGCCGGAGCGGGGGCGGCCGCGGCGGCGGCGGCGGCGGGCACGGCCACCGGCTGGGCCTGCACCACCACCGGCTGCTGCGGGACCACCTGGGCGGGGACGTGCGCGACGGGGGCCGCCAGACCCTGGAGGCCCGGCAGGCCCGGGAGCGCCGGCACGGCGTGCGCCACGGGCGAGGGGGGCGGCGCGTGCAGCCCCGGCTGGTGGGCGTACATCGGCGGGTACAGCGCCCCGGCGGCGTGGTGCGGGTGCTGGTGCGCGTGTCCCACGACGGCCCCGGGGGGCAGGCCGGGCGGCAGCAGCTGCGGCGGCGTCGCGGGCGCGTAGTACGGCGTCGGGTAGCGGAGCTGCTGGGCGCGCAGGTGGCTGATCTCGCGCTGGAGCGACGAGACCACGGCGGCGAGGTCGCTCGCGGGGGGCGGCGCCTCCCCGGGGTAGTACGCGTTGTCCTGCGCGTAGTCGTCGTAGCGGCGCTTCCGGGCGGGGGGCCGCGGCTCGGGCGAGGGCGAGCGGCGGTGGCGGCGGTCCATGGCGGCCGAGACGATGGCGCCGAGCTGGGACTCCAGGCTGGGCGCCGGGCGCTGGTTCACCACCAGCTGGTTGTACTGCGCCGCGGGCACGAAGATGTAGTCGCCGGGCGCTGCGCTCGGCGGGGCTGCCATGGCCGCACTGGGCCCGGGCGCCGGGGAGGCCCCGCTTTTAGGCAGAAGCCCCGCCCACATCGTCGCCTGCAGGTAGGTGTGTCCGCGCACGCCGGCCTCGCGGCGGCGCGCCGCGACCAGCTCCCAGCGGTCGCGCAGCAGCATGTTGTTCACGGCCGTCGACAGCAGCGCGCGCGTGAGCGCCTCCTCGCTCATGTGCCACACGCGCTCGCGGTCCGGCGACTCGGCGGCGCGCGCCAGCAGCTCGGAGCGCGCCCGCGCCGAGAGCTGCCGGAACGGCGCCACGGCGGCCTCGGGCGAGGCGTCGTACACCACGATGGTGCCCACGCGCCGCCCGATCACGCAGAGCGCCACGTGCGCAAAGAGCGTCTCGTCGGGGGCCTCGCCGGGCGCCAGGCGCCGCGAGGACAGGGACGCCGAGGGCAGGTAGTTGCTCAGCAGGTACAGCAGCCGCTCCTCGTCCGAGAGCCGCATGTCCCCGAAGAAGTCCGGGCCCACGGCGCGGGCGAGCACGGCCCCCAGCTGCGGGCAGTTGACGACGCCGAGGAAGAAGGGGCCGCGGTCGTCGTCGACCACGGCCAGCACGGCGCCGATGTCGCAGCGCGGGCGGTGGTCGATGTTGATGGGCAGCGGGCCCGCCGGGGGCAGCGCGGCGGCCACGACCTCGCGCGTCAGCGCGAGCTCCCCGCCGTCCCTATCGTACAGGGCCAGGTAGCCGGACACGTACACGGGCCCCATGGCGGCGGCGGTGGCGACGGCGGCGGCGCGCGCGCGGAGGAGACTGCGGGCTCAGGCGGCGGCGAACTGCGGGATATAGCCCAGGCAGAGAAAGTACAGGAGGTCGTAGTCGCTGGAGACGCCAAACTGCCCGAGGCGCTTGTCCGCCGCGTTGCGCAGGTGCGGCTGCTGCAGCAGCAGGCCCAGCCCCTCCTCGTACTGCACGGCCACGGCGTCGTGCGCCGCGATGACCTCGTTGACGGGGGCCGTCACGCGCTGGCGGTTCTGCAGCTCCAGCGCCACCAGGCGCACCAGGCTGAGCTGGTGGCGCTGCACGCGCGCGCGGATCGAGTGCGCGTCCAGCGCCAGCGCGGCCAGCCCGGGGAAGAGCTGCGTGAGCTCCGTGCGCTCGTCCGCGCGGTAGGCCACGACGACGTAGTGGGCCAGCAGGAAGCGCAGGTTGCCGTCGCCGCTGCGCGAGGCGGGCGCGTCCCCGCTGGCGCCGCGCGGCGGCGGCGCGTTGGGCACCAGCGCGCCGAGCTGGAAGTTGTTGCGCAGGCGGTCCCCGTACACGTTGCCGTTGTGCAGCAGGCGCCGCAGCAGCAGCAGGCCCGTGATGGTGTTCACCGTGGCGCGCAGCAGCGGCGCGTCCTCGCCGAGGAAGAGGTTCTGGCCGCGGCCCAGGAAGGCGGCCGCGGCGCGGTTGACCTCGTCCACGAAGCCCGCGCCGTCGTCGACCTCGTTGGCGCCGACGTTCGCGCCGGGGACCACGCGCAGGCGCGCCAGCGCGGCGAGCACGCTGTGGCCCTCCAGCGCGTAGCGCCCGTGGAGCTGCTCGCGCGGCACGCGCGCGTAGTTGTAGCGGTACGAGATGCGCCCGCCCTCGGCCAGCGCCTGGCTCAGCGCGTCCAGGTACTCGGGGATCCGCTCCAGCAGGTCCACGATGCCCAGCGGCGCGGGCGCCGCCGCCGCGGCGGCGGGGCCCTGCCGCGCGCGGTGCACCAGGTGCAGGCACAGCGCGGCGCTCTCGAGCGCCGAGTAGTGCCGGTTGCCCACGTACAGGCGCCCGCAGGCCTGCAGCGCCGTGGTCGTCACGCCCATGAAGGTGCGCGACATGCGGCCGTCGCGCGAGTCGATGGCGCGCGAGACCTGCGGGCGCTCCGCGACGAGCGTGGCCTGGAGCGTGGCGTACCACGTGCCGAAGAGCACGCCGGAGGCGGCGCCGCGCGCGGCGTACACCATGGCCAGGAAGTCCAGCGAGAGGTTGGCGTCGTAGGCGAGCGCCACGTCGTTCTTGGCGATCTGCACCTCGCGGCCCTCGTCCGCGGCCGCGACCGCCTCGGGCGCCTCCTCGGCGGCGCGCGCCGCGTCCGCCTCCTCGGCGGCGCGCGCCGTCTCCTCGAGCACCACGAGCGCCTCGGCCACGCGCTCCACCTGCCGCTCCAGCGGCCGCAGCTGCTCGTCCACCTCGGCCTCGAGGCGCGCGCCCGCGGCCATGGCGTTGTCCAGCGCCGCGGCGGCCGCGCGGCGGCGCGCGTTCGCGTGCGCCAGCGCGAGGCGCGCGTCGAGGCCCTCGCCGAAGCCCGGGCGGGCCCAGAAGCCCACGGGGAACGGGGGCGCGATGAAGTGGCGCGCGCTGCCCGGGATCGCAGCGGCCTCGAAGGCGAACCACGCGCGGTCCATGGCGCGGGGGGACATGGGCCGCGCGGCGGCGCGCGCCGCCTTATCATCCCCGCTCCCCGCCGCCGCCCGGCCCGGCCCCGCGCGCGCCGCGATCGCGATCACCGCCGCGGCCCGGCGACGTACTCGGCGAGGCCGCGCACGGTCGCGGCCATCGCGCTCGCGTTGCCGCGCGTCTGGGTGCAGGGCAGGCGCGTCACGTCGAGCACGCGCATGCTCCGCTGGGCCACAAACACCAGCAGGGGCACGAGCGTGATCTCCTCGCCGCCCGGGGGCACGGCGGCGGCGAGGAGGCGCGCCGAGTCGCGCAGCTGGCACAGCCCCTCGTGCCGCTGCCCGCGCTTGCTGGGCGTGTTGAGGTTCCGGGGGAAGCGGCACGTCTTGAGCTCGATGAGGAAGCACAGGTGCGGGCCCGCCCCCAGCCGCACCACGCACACGCAGTCGGGGCGGCGCACCCCGAGGTTGACTTCAAAGGCCAGGGTCAAGGACGCCTTCTTAAGCGTCTCTCGGGGAAGCCCGAAGAGACTCTCGCCGTACGCGGACGGGTCGCGTCGCAGGCGTTCGTAGAAGCGGTTGTGGCAGCGGATCCCCGCCCGGAAGCGCGCCGGGATGCGCATCCTCCGGATCTACCTCGACGGCGCCTACGGCACCGGCAAGAGCACCACGGCCCGGGTGATGGCGCTCGGCGGGGCGCTGTACGTGCCCGAGCCGATGGCGTACTGGCGCACTCTGTTCGACACGGACACGGTGGCCGGTATTTACGATGCGCAGACCCGGAAGCAGAACGGCAGCCTGAGCGAGGAGGACGCGGCCCTCGTCACGGCGCAGCACCAGGCCGCCTTCGCGACGCCGTACCTGCTGCTGCACACGCGCCTGGTCCCGCTCTTCGGGCCCGCGGTCGAGGGCCCGCCCGAGATGACGGTCGTCTTTGACCGCCACCCGGTGGCCGCGACGGTGTGCTTCCCGCTGGCGCGCTTCATCGTCGGGGACATCAGCGCGGCGGCCTTCGTGGGCCTGGCGGCCACGCTGCCCGGGGAGCCCCCCGGCGGCAACCTGGTGGTGGCCTCGCTGGACCCGGACGAGCACCTGCGGCGCCTGCGCGCCCGCGCGCGCGCCGGGGAGCACGTGGACGCGCGCCTGCTCACGGCCCTGCGCAACGTCTACGCCATGCTGGTCAACACGTCGCGCTACCTGAGCTCGGGGCGCCGCTGGCGCGACGACTGGGGGCGCGCGCCGCGCTTCGACCAGACCGTGCGCGACTGCCTCGCGCTCAACGAGCTCTGCCGCCCGCGCGACGACCCCGAGCTCCAGGACACCCTCTTCGGCGCGTACAAGGCGCCCGAGCTCTGCGACCGGCGCGGGCGCCCGCTCGAGGTGCACGCGTGGGCGATGGACGCGCTCGTGGCCAAGCTGCTGCCGCTGCGCGTCTCCACCGTCGACCTGGGGCCCTCGCCGCGCGTCTGCGCCGCGGCCGTGGCGGCGCAGGCGCGCGGCATGGAGGTGACGGAGTCCGCGTACGGCGACCACATCCGGCAGTGCGTGTGCGCCTTCACGTCGGAGATGGGGGTGTGACCCTCGCCCCTCCCACCCGCGCCGCGGCCGGATGGAGACCGCGACGGAGGCAACGACGACGGCGTGGGAGGGGGCTCGGGGCGCGTATAAAGCCATGTGTATGTCATCCCAATAAAGTTTGCCGTGCCCGTCACCATGCCCGCGTCGTCCGTGCGCCTCCCGCTGCGCCTCCTGACCCTCGCGGGCCTCCTGGCCCTCGCGGGGGCCGCCGCCCTCGCCCGCGGCGCGCCGCAGGGTGGGCCGCCCTCGCCGCAGGGGGGTCCCGCGCCCACCGCGGCGCCCGCGCGCGGGCCCACCCTGTTCGTCCTGGACGGCGACGGCTCCGCGTGGTTCGTCTTCCAGCTCGGCGGGCTGGGGGCGCTCAACGACACGCGCATCCGCGGGCACCTGCTCGGCCGGTACCTCGTCTCGTACCAGGTGGTGCCCCCGCCCGTCTCCGCGTGGTACTTTGTGCAGCGCCCGCGCGAGCGCCCGCGCCTCTCGGGGCCGCCCTCGGGCGCGGAGCTCGTGGCCTTCGACGCGCCCGGCGTCTGGCGCACGTACACCACGGCGGCGGTGTGGCCCGCGGAGGTGGCCGTCCTCGCGGACGCGGAGGCGCGCTGCCCCGCGGCCGTCTTCAACGTGACGCTGGGCGAGGCCTTCCTCGGCCTGCGCGTCGCGCTGCGCTCCTTCCTGCCGCTGGAGGTCATCATCTCCGCCGAGCGGATGCGCATGATCGCGCCCCCGGCGCTCGGCTCGGACCTGGAGCCGCCGGGCCCGCCCGCGGGCCGCTTCCACGTGTACACGCTCGGCTTCCTCTCCGACGGGGCCATGCACCAGACGATGCGCGACGTGGCCGCCTACGTGCACGAGAGCGACGACTACCTCGCCCAGCTGTCGGCGGCGCACGCGGCCGCCCTGGCCGCCGTGGTGCAGCCCGGGCCGTACTACTTTTACCGCGCGGCGGTGCGCCTCGGCGTGGCCGCCTTCGTCTTCTCCGAGGCGGCGCGCCGCGACCGGCGCGCCTCGGCGCCGGCGCTCCTGCGCGTCGAGAGCGACGCGCGCCTGCTCTCGCGCCTGCTCATGCGCGCGGCCGGCTGCCCCGCGGGCTTCGCCGGGCTCTTCGACGGGCGCGCCGAGCGCGTCCCGGTGGCGCCCGCGGACCAGCTCCGCGCCGCCTGGACCTTCGGCGAGGACCCGGCGCCCCGGCTGGACCTCGCGCGGGCGACCGTCGCCGAGGCGTACCGCCGCTCCGTGCGGGGGAAGCCCTTCGACCAGCAGGCGCTCTTCTTCGCCGTCGCCCTGCTGCTGCGCGCCGGCGGCCCCGGCGACGCGCGCGAGACCCTGCTCCGCACCACGGCCATGTGCACCGCGGAGCGCGCCGCCGCGGCCGCCGAGCTCACGCGGGCCGCGCTCTCGCCGACGGCCGCGTGGAACGAGCCCTTCAGCCTGCTCGACGTCCTCTCGCCGTGCGCCGTCTCGCTGCGCCGCGACCTCGGCGGGGACGCCACCCTGGCCAACCTGGGCGCCGCGGCGCGGCTCGCGCTGGCGCCCGCCGGGGCCCCGGGCGCGGCGGCGGCGACGGACGAGGGGGCGGAGGAGGAGGAGGACCCCGTCGCGCGCGCCGCGCCCGAGATCCCCGCCGAGGCGCTGCTCGCCCTGCCCCTGCGCGGGGGCGCCAGCTTCGTGTTCACGCGCCGGCGCCCGGACTGCGGCCCGGCGTACACGCTCGGCGGCGTGGACATCGCCAACCCGCTCGTGCTCGCCCTCGTCAGCAACGACAGCGCCGCGTGCGACTACACGGACCGCATGCCCGAGTCCCAGCACCTGCCGGCGACGGACAACCCGTCCGTGTGCGTGTACTGCGACTGCGTGTTCGTGCGCTACTCCTCCGCGGGCACGATCCTGGAGACCGTCCTCATCGAGTCCAAGGACATGGAGGAGCAGCTCATGGCCGGCGCCAACTCCACCATCCCCAGCTTCAACCCGACGCTGCACGGCGGCGACGTCAAGGCCCTGATGCTCTTCCCCAACGGCACCGTGGTCGACCTGCTGTCGTTCACGTCGACGCGGCTCGCGCCCGTGTCCCCGGCCTACGTGGTGGCCTCCGTCGTGGGGGCGGCCATCACCGTGGGGATCCTGTACGCCCTGTTCAAGATGCTCTGCAGCTTCTCCTCCGAGGGCTATTCTCGGTTAATAAACGCCAGGTCGTGAGGCCCGCGCCGGGCCGCGAACCCAGACTCTCTGCGTGCGCGTGTTTTTTCCTTGTCGGGCGCAGGGGAGCCGGGAGGGGGGAGAGAGACGGCACGGGCGCCGTCCGTCGGGGAGACGGCGGGATGACATTACGAGAGTCGGGTGGAGGGGATGCGGGGAGCGCCGTCCACGGGGGAAGGCTGCTGGATGACATAATTAGAGCCAGGGGTGAGGATCCCCTTTCCCCCGTTCCCCGAGTATACACCACTCTCTCCCCCGCACACATTTCCCACCGGGCGCCGAACGTGGATTGCACCCCGGTCCGCGCTACCCGGTTGCGTGTGAGACGGGCGGAGAGCCGCCCTCGCCCCGGTCCCCCAAAAACTCCTGCGGATGCGTGGTCCGACGAAGCCTCCGCGGATATGTCCGCCGTACCCTCATCCCCCTCTCTCCCACAGCCCTCTCTCCCACAGCCCTCTCTCCCACAGCCCTCTCTCCCACAGCCCTCTCTCCCACAGCCCTCTCTCCCACAGCCCTCTCTCCCACAGCCCTCTCTCCCACAGCCCTCTCTCCCACAGCCCTCTCTCCCACAGCCCTCTCTCCCACAGCCCTCTCTCCCACAGCCCTCTCTCCCACAGCCCTCTCTCCCACAGCCCTCTCTCCCACAGCCAGACTCCCGCCTCCCCCGTCCCCGATCGCCGAACGCGTTCAAGAATTTTTTACCCGCTCCGAGGAAGGGGTCTCTAAGGGGTCTCTAAGGGGTCTCTAAGGGGTCTCTAAGGGGTCTCTAAGGGGTCTCTAAGGGGTCTCTAAGGGGTCTCTAAGGGGTCTCTAAGGGGTCTCTAAGGGGTCTCTAAGGGGTCTCTAAGGGGGAACCAGAGAGTAAAGACCGGAGAGTAAAGACCAGAGACACATTTTACAATTTCGTTGTACCCGGGTTTATTGAAAACTTTGTTCAAGAGTTAAAGTGTCTGCCGCTTGCCGGTGCGCCGCGCCGAGGCCGCGATCCGTCCGCCGTGGACCGGGGGATCCGTGGGGCCCGCGGGCCTGGATTCGACGGGATGGCGGGTCCGAGCGACGGCGGTCCGGCGGCGTTTCGGCTCGCGGTCCTCGCGGCGGCGACGATCCTGGTCCGGGGGATACAGCGGCGACGGCGGCGCTCCGGGTTGTGGCGCCGGGCTCCGGCTCCCCGGTCTTTTATACCCACACCTCATGAATATTCATGAATGGTTGGTATGTGCGAATTTTTCGGCGTTCGCACTTGTTGTATATAAAATATTATATCCTATATATATTAGCAATTGGTGCGAACGTGACGTGGCCCAATCATTTTCCTCGGATTTGCCCATGTGACCGAGTTTCCAATAGCTCCGCCCGGATCGATCACCGGGCCGATATTTTGCCGATAATCCATCAATATTTTCTTCGGCCGACGCGGGTTCCACCCGGGCCGTCCCGCCGGGGAGCCCGGCCCGGGCGCGGGACTCTGCTCTCCGGGCGCCGCGCGGCGTCGAGACCGCGCCGGGACGATGGCGTCTGCGCATGCGCGGGTGGAGGCGAGTGGCCGGGAATCCCCTCTCGTGCCGAGCCCCGCCCATCGTCGCGTGTCCTCTCCCTCACCCCCTTCTCCCCCGACCGGGCGACGATGGGCCGCGTCCTCGCCACGATGGAGGAGAGGCGCTCACGCACGCGCGGGTACGCGGGAGTTGCGGGGATCGGGGACCGGGACGATCGGGGTGTGCGGACCGTGGCGCGTCGGGGGGCTGGGTGCGAGCGGGGGTTGGGCGCGCGCGTGTGTGGGGGGGGAACGGGAAACGGGGGACGGGGGAACCCGCCGAGGCGCGCGTGCGCGCGCACGCGTGTGTGTGCCGCGGACAGAGACGGACGGGAGAACGGACGTGTGATTTGTTTAACGGTTTTTATTGAGGACGATGGAGATGTTGGCGCGGGCCAGGCGCGCCCGAAACAGCTGAAACAGGTACTGGGCCCCACGGAGGGCCGCGGGCGAGTCGCCGAAGGCGGTCAGGAGCGCCGCCTCGGCGCAGACGGCGGACGTGAGCCGCAGCGCGCGGCCCACGGGCGCGGTCGCGGCGACGAGGCCCCGGCCGGCGTAGAGCCCGTCCAGCACCCGCGCCCCGGCCGCCTCCACCAGCTCGCACACGCCGGGCGCCGGCGCGTTCGCCTCCGCGTCGCTCATCATGACCGCCAGCTCCAGGCCCGTCGCCGCGTCCTCCCGGGCGTGCTTCGTCGTCGGCGGTGAGGCCCCCGCCGGGGCCGCGGAGACGATGGGCGCGAGCGCGGGGGCGTGCACCACGGCGGCGTTCACGAGCATCCGCATCACCTCGCCGAAGACGGTCGCCTCGCGCAGCACGCGGTTGACGGCGTGGGCCACGGCGCTCGCGGCCCGAGAGGCGCCGCCGTCGCCGTTGCCGTTGCCTCCGCCGCCTCCCCCCTCGCCGCCGTCCTCGTCGTCGCCCGCGCCCGCCCCGGGGCGCTCGTCCAGCGTGTCGAGCATGCCCTCCAGCGCGGCGTAGCAGCTCGGCAGGTGCCAGCGGGCCAGGATGAACTGGTACAGCGCGAACCGCTGCGCGCCCGTGAGCGACGTGGAGCCCGTCCCGAAGAAGGGGTCCGCCGCGACGGCGGACACGGCCTCGACCACGGCGGCCGAGGCGTCGTCCGAGGCGCACGCGGCCCGCCGCAGGATGGCGTCCGCCCCGGCCACGAGGTCGCAGAGCATCTGCAGGGACAGGCTCCCGCCGCGCGTCGCCCAGATGCGCCGCGTCGCCGGGATGTAGCGCACCTGCACAAAGTCGCTGACCACCGTCGCGCGCAGCGCGCGCTCGGCGTACCGGCGCGGGCGCACGCTCAGCGTGGGGGCCGCGCGCCCGCCCTTCACCACCACGGCCGTGGGCCGCGTCTCGCCGCTGAGCGGGGGGCGCACGCCGGGGATGCCGTCAAAGAGCCCCTCCGTCAGCACCTGCAGCGAGCTGGGCAGCTCGGAGAGGTACGCCTGCACCAGGGCGAAGCAGGCCTCGTTCACGGCGTACACGAAGCCCGAGGAGGGGTGCACGATGGCGGGCTCGGCCAGGAGCTCCAGGACACGGTCCTGCTGGCGGTCGTGCACGAGCGTCAGCGAGAGCGCCGACGCCGGCAGCGAGACCATGCACTCGACGAGGTAGGGGTCGTACACGTCCAGCTCCGCGCGGCCGCAGAGGTCCAGCTCCGTCAGCGGCAGCAGCAGCCCCAGGGCGTCCACGAAGACGTCGTCGCTCCCCTGCGGGGCGGCCCACGCGCGGCCGAGGCGCGCCAGCTCCGTGCGCACGTAGTTGGCGACGACGCGGTTCCCCGGCGCGTTCCCGCGCAGCGTGAGCCCGAACTTGCCGATCTCCCGCGAGCGCCGGTGCACGGACACGATGCAGCCCCCGTGCACAAAGTACGCGCGGTCCCCGCCGTCGGCGATGTAGAACAGCACCCCCTGGTGCACGATCGTGCTCTGGTACTCAAACTCCATGGCGGCGGACACACGCGGCGCGGCAAATGGGTGAGCGAGGCGGCGCGACCACTCCTTTAAACCCGCCGGGGGGCGCCTCCCGCCGCACACACGCGGCGGCGCGGGGATGGAGGACGCGGCGGCGGTGGACGTGGACGCGGCGTCGGACGCGACGACGACGGCGGCGGTGGGGGAGAAGGACGCGCTGCTGAGCTCGGCCTTTGTGGGCGCGCGCCCGCCGCGCCCGCGCTTCAGCTCGCACGTGGTGAGCCTCCTGGTCCTGGCGCTCGCGCTCCGGCCCGCGTGCTGCCTCGTCCTGGCGCTCCACGGATCGCGGGCCACCCTCGCGGCGCTCCTCACCGCGCTGGCCTTCTACGCGCGCGCGGCCGTCTGCGCCGCCCTCGTGGCGCGGAACGTGGCCCGGGACCGGATGCCGCTCTCGCCGGCGCAGCAGGTGGCGCTGGGGCTGCTGGCCGCGGCGCGGCTGGCCTTCTTGTACGTCGCCCTGGACGCGGGCCGCCATTACGCGCCCGCCCTCGCCGGGGCGCTGTACGGCGCCGACTGCGTCTGTGACGCGCTCGCCTTCCTGCTGCCGCGCGCGTACGCGCGCTCCATCATGCATTAAAAGGCGGCCGCGACGCGCCCGCGCCATTCTTCCCCCGCCGCGCCCCAGCGACCGCGCACACGCGCCCGTCCGCGGTCGTCGCTACACGATGGAGCGCCCGGCCATCCTGCCGTCCGGGCAGATCCTGTCCAACATCGAGGTGCACTCGCACCGCGCGCTCTTCGACCTCTTCAAGCGCTTCCGCTCGGACGACAACAACCTCTACGGCGCCGAGTTCGACGCGCTGCTCGGCACCTACTGCAGCACGCTCTCGCTCGTGCGCTTCCTGGAGCTCGGGCTCTCGGTGGCGTGCGTGTGCACCAAGTTCCCGGAGCTCAGCTACGTGGCCGAGGGCACCATCCAGTTTGAGGTGCAGCAGCCGATGATCGCGCGCGACGGGCCGCACCCGGCCGACCAGCCCGTGCACAACTACATGATCAAGCGCCTCGACCGCCGCTCCCTCAACGCCGCCTTCTCGATCGCCGTCGAGGCCCTCGGGCTCATCTCGGGCGAGAACCTCGACGGCACGCACATCTCGTCGGCCATGCGCCTGCGCGCCATCCAGCAGCTCGCGCGCAACGTGCAGGCGGTGCTGGACTCGTTCGAGCGCGGCACGGCCGACCAGATGCTGCGCGTCCTCATGGAGAAGGCGCCGCCCCTGTCGCTGCTGGCGCCCTTCACGCTCTACGAGGGCCGGCTCGCGGACCGCGTGGCCTGCGCCGCGCTCGTGTCGGAGCTGAAGCGCCGCGTGCGCGACGACACCTTCTTCCTCACGAAGCACGAGCGCAACAAGGACGCGGTCCTGGACCGGCTCTCGGACCTGGTGAACTGCACCGCGCCCTCGGTGGCCGTCGCGCGCATGACCCACGCGGACACGCAGGGGCGCCCCGTCGACGGGGTGCTGGTGACCACGGCCGGCGTGCGCCAGCGGCTCCTGCACCACGTGCTGACCCTGGCGGACACGCACGCGGACGTGCCCGTGACCTACGGCGAGATGGTGATCGCCAACACCAACCTCGTGACGGCGCTGGTGATGGGCAAGGCCGTCAGCAACATGGACGACGTGGCGCGCTACCTGCTCGGCGGCGAGCCGGCCCCCGACGACGGCAAGCCCGTGGGCTCGGCGCGCGTGCGCGCGGACCTCGTCGTCGTCGGCGACCGCCTGGTGTTCCTCGAGGCCCTCGAGAAGCGCGTGTACCAGGCCACGCAGGTGCCGTACCCGCTGGTGGGCAACCTGGACGTGACCTTCGTCATGCCCCTGGGGGTCTTCAAGCCGGCCGCCGACCGCTACGCGCGCCACGCCGGCAGCTTCGCGCCCACGCCGGGCCTCCCGGACCCGCGCACGCACCCGCCCCGCGCCGTGCACTTCTTCAACAAGGACGGCGTGCCCTGCCACGTGACCTTCGAGCACGCCATGGGCACCCTCTGCCACCCCTCCTTCCTGGACGTGGACGCCACGCTGGCGGCGCTGCGCCAGGAGCCCGCGGAGGTGCAGTGCGCCTTCGGCGCCTACGTGGCCGACGCGCGCCCCGACGCCCTCGTCGGGCTCATGCAGCGCTTCCTCGAGGAGTGGCCCGGCATGATGCCCGTGCGCCCGCGCTGGGCCGCGCCGCCGGCCGCCGACCAGCTGCTGGCGCCCGGCAACGCCGACCTGCGCCTGGAGCTGCACCCGGCCTTTGACTTCTTCGTCGCGCCCGAGGTCGACGTCCCCGGGCCCTTCGCCGTGCCCCAGGTGATGGGGCAGGTGCGCGCGATGCCGCGCATCATCAACGGCAACATCCCGCTGGCCCTGTGCCCCGTGGACTTTAGGGACGCGCGCGGCTTCGAGCTCAGCGTGGACCGGCACCGGCTGGCCCCGGCCACGGTGGCCGCGGTGCGCGGCGCCTTCCGCGACGCCAACTACCCCATGGTGTTCTACATCATCGAGGCCGTGATCCACGGGAGCGAGCGCACCTTCTGCGCGCTCGCGCGGCTCGTGGCGCAGTGCATCCAGAGCTACTGGCGCAACACACACAACGCCGCCTTCGTGAACAACTTCTACATGGTCATGTACATCAACACCTACCTGGGCAACGGGGAGCTGCCCGAGGACTGCGCCGCCGTGTACAAGGACCTGCTGGAGCACGTGCACGCCCTGCGGCGCCTCATCGGCGAGTTCACGCTGCCCGGGGACCCGCTGGGCAACCAGCCCCAGGAGGAGCTCAACCACGCGCTGGCGGACGCCGCGCTGCTGCCGCCGCTCATCTGGGACTGCGACCCCATCCTGTACCGCGACGGGCTCGCCGAGCGCCTGCCGGAGCTGCGCGTCAACGGCGCGCACTTTCAGCACATCCTGTGGGTCGAGATGGCCCAGGTGAACTTTCGCAACGTCGGCGGCGGCCTGGTCCACAACCGGCCCGTGCGGAACGAGAACCAGCCCCTGCACCCGCACCACGACGCCGAGTGGTCGGTGCTGTCCAAGATCTACTACTACGCCGTGGTGCCCGCCTTCTCGCGCGGCAACTGCTGCACCATGGGCGTGCGCTACGACCGCGTGTACCAGCTGGTGCAGACGATGGTGGTGCCCGAGGCGGACGAGGAGGTGGGCGCGGACGACCCCCGGCACCCGCTGCACCCGCGCAACCTCGTGCCCAACTCGCTGAACGTGCTCTTCCACAACGCCTGCGTCGCCGTGGACGCGGACGCCATGCTGATCCTGCAGGAGACGGTGACGAACATGGCCGAGCGCACGACGCCGCTCCTGGCCTCGGCGGCGCCCGACGCGGGCATGGCCACGGTGGCCACGCGGGACATGCGCACGCACGACGGCTCGCTGCACCACGGCCTGCTCATGATGGCCTACCAGCCCAACGACGCCACGCTGCTCGAGGGGGCCTTCTTCTACCCGGCGCCCGTGAACGCGCTCTTTGCCTGCGCCGACCACCTCGGCGCCATGCGCGACGTGGGCGCCGAGGTGCGCGCCGCCGCCCAGCACGTGCCCTGCGTGCCCCACTTTCTCGGGGCCAACTACTACGCCACGGTGCGCCAGCCCGTGGCCCAGCACGCGGCGCAGAGCCGCGCCGACGAGAACACGCTCTCGTACGCGCTCATGGCGGGCTACTTCAAGATGAGCCCCGTGGCCTTCACCCACCAGCTGCGGCGCCAGCTGCACCCCGGCTTCGCCCTGACGGTGGTGCGCCAGGACCGCTTCGCCACGGAGAACGTGCTCTTTGCGGAGAAGGCCAGCGAGTCGTACTTTATGGGCCAGATGCAGGTGGCGCGCACCGAGAGCGGCGGCGGGCTGCACATGCAGCTCACGCAGCCCCGCGCCAACGTGGACCTGGGCGTGGGCTTCACGGCCGCGTACGCGGCGGCCGCGCTGCGTGCCCCCGTGACGGACATGGGCAACCTGCCGCAGAACCTCTTCGCCACGCGCGGCGCCCCGCCCATGCTGGACGCGGACGCGGACGACTACCTGCGCCGCACGGTCAACGCCGGCAACCGCCTGGCGCCCGTGCCCGTCTTCGGCCAGATGCTGCCCCAGGTGCCCGTGGGCCTGGCCCGCGGGCAGCAGTCGGTGTGCGAGTTCATCGCCACGCCCGTCTCGGTCGACCTGGCCTACTTTCGGCGCGCGTGCAACCCGCGCGGGCGCGCCGCCGGCGAGGTGCACGGCGAGGAGGGGCTGATGTTCGACCACAGCCACGCGGACCCCGCGCACCCCCACCGCGCCACCGCCAACCCCTGGGCCTCGCAGCGGCACTCGTACGCGGACCGGCTCTACAACGGCCAGTACAACATGAGCGGCCCGGCCTACAGCCCCTGCTTCAAGTTCTTCACGCCCGCCGAGGCGGTCGCCAAGAGCCGGGGGCTGGCGCGGCTCATCGCCGACACGGGCGCCGCCGCCTCGCCGACGAGCAACGGCGAGTACCAGTTCAAGCGGCCCGTGGGCGCCGGCGAGCTCGTCGAGGACCCGTGCGCGCTCTTCCAGGAGGCGTACCCGCCGCTGTGCGCCAGCGACTCGGCGCTGCTGCGGACGCCCCTCGGCGCCGAGGAGCACTTTGCGCAGTACCTCATCCGCGACGAGTCCCCGCTGAAGGGCTGCTTCCAGCACGCGAGCGCCTGAGCCGCCGGACCGATACCGGACCGCCGCCGAACCGTCGTCTCCGCCCTGCCCCCTCCCCCCCCGACTGCTCTGTCACCCGCCCCCACCCTCCCGCGCCGGCCGCGCGCCGCGCGTGTATATAAACTGTGTACACGGCACCCGCCCGCGTCAGTGTCTTCTTTCGCTCGCGTCGCCATGGAGGTGGACATTGCGCTGCCGACGCTGTCCCCGGGGGATCTGTCGGCCCTGCAGAGGTGCGAGGGCCGCGTGGTGTTTCTGGAGACGCTGCGGCGTCACGCGACGCTGCGCGAGGTCGCGCTGCCCTGCGGCGGCGATGTGCTCGCGGCCATGGCCGCGTACCGGCGGCGCTTCGCGGCCGTCATCACGCGCGTGACGCCGCACCGCATGCTGGCCACGCCGCTCGGCGTCGGCGGCCGCGGGCAGTCGCTGGTGCTGCAGAACACGGGGCCCTTCGACCTGACCAACGGGGACCACGTGTGCCTCGTCCCGCCGCTGCTCGGCGACGAGTGCCTGCGGCTCACGTCCGCCAACCTGGAGCTGCGCTTCCCCATGACGCTGCCGCTCGCCCAGGCCCGCGAGCTGACGGCGCGGGTGGTGGCGCGCGCCGTCGAGACGCTGCGCGGCGGCGCGCCCGCGCGGGGCGCCGACGTGGTCTTCAACAACGGCCGGCGCTACCAGCTGCCCCCGCCGCACCGCGACAACGCCGAGGCGGCCACGCGCTCGCTGGTGCTGAACATGATCTTCCTGCTCAACGAGGGCGCCGTGATCCTGCTGTCGCTCATCCCGAACCTGCTGACGCTGGGGGCGCAGGACGGCTACGCCAACGCCGTGATCCAGCTGGGCAGCGCCACGCGCGAGCTCGGCCAGCTGGTGCGGCAGCCCCCGCCGCCGCTGCCGCAGGACCACGCGCGGCGCTTCTGCGTGTTCGAGGCGCTGGAGGCCTGGATCGCCTCGGCCTCGCGGCTGGGCGACACGCTCGGCACGCGGCCCGTGGCGCGCGTGTGCATCTTCGACGGCCCCCCGACCGTCCCCCCCGGCGAGAAGGCCGCGGTGGTGGAGGTGTGACGGCGCGGCGCCGAGGGGGCCCGTGTTTCCCCCGCCCCACCCCACCCCCCTGCAATAAAAAAATCCGCGAAGGAAAACAAAGAAACGAGTCGTGCGCGTCTGGCGTTTATTTTCCGGGTCGGGGAAAATGGGGGCGTCGGGGGGTGGAGGGCGGACGGTGGACGGTGGATGGGGGAAGCCCCCTCAGGCAAACAGCCCGTCGGGCAGCGTGCTCAGGTGCACGGCCATGACGAGGGCCACGGCGAGGTCGTCGGCGCAGCCGCCGCGCTTGCCGGTGAAGACGCGGTGCTCGCCGGCCCCCGAGGCCGTCTCCACGAGGTTCCCGAGCTGGGCGTTCAGGTACTCGACGGGGTCGGCGCGCGCGCCCACGGTCACGGACACGAGCTCCTGCGAGGCCATGACGGCGCCCGAGTTGAACTGCTTCACGAAAAAGTCGAAGGCGGGGGTCTTTTGCTTCTGCAGCAGGAAGAAGGGGTAGCGCACGGCGCCCGCGCCCGCGCCCGGGGCGCCGTGGTGGTAGAAGACCACGGCCGGGTCGGCGACGACGCGCGCGGCGCGCAGCTCCGCGAGCTCGCGGCGCATGGCGCCCACGATGGCCACGGCCGAGTCCTGGCTGCTGTTGCCCTCGACGGCCACGCGCACCGCCGAGAAGGCGCCGTCGTGCAGCGCCAGCACGCGCGCCAGGCAGCGCACGACGCAGCGCGCGATCTCGTCGGCGGAGGCGCCGGTGAGCGCGCGCAGGAAAAAGTGCTCCAGGCCGAGCACGATCCAGCTGCGCCGGTAGCTGCCCACGACGGCCACGCCGGTGCCGGAGGCGCGCGCGTTGGCCGTGAAGGCCGGGTCCACGTACACGTGCAGCGTGCCGGCCAGCAGCCGGCGGTTGGCCACCGAGGAGGGGCGGTAGAGCAGGAAGCGCTCGCCGGCGGCGCGCGTGAGGACGGGCGCCTCGCGCGCGGCCACGGCGCCGCCGAGGATCTCCTGCATGAAGGAGTCCGGGAGCAGCCGCTCGGCCGTGGTGCGCGTGGCGGCGTCCATGGTGATGAAGACGGGCTTGTTGAGCACGTAGCACGAGCAGGCGGTGGCGTCGGTGTGCGCCACCACGCGGCGCATGTGCTCGTCGCAGACGTACGTGACCACGTTGAGCATGTCGGTGCCGCGGAGGTTGGTGAGGAAGCTGGTGCTGGCCTTGCCGGTGTTGGTGGAGGAGACGAAGATGATCTTGCAGCTGGCCTGGTTCATGAAGCCGAGGATGGTCTGCACGGCATCGGGGCGTATAAAGTTGGCCTCGTCGACAAAGAGCAGGTTAAAGTCCTGTCCGCGGATCCCCTGTAACGACACGCGGGGAGAGAGAGGGGGGGACGGTGAGCGGCGCCGCGATGGACGCCCACATCGCCAACGAGACCAAGCAGCAGATGACGCGCTTCGCGCCCGCGCTCGTGCACGTCATCGTGCCCGACCCCCTCCTGCGGCGCGCGGGCGTGGACCCGCTCGCGCCCTTCGCCGCCCACGCGCAGACGCGCTACCACGGCTCGGGCGTCTGCGAGCCGTGGGTCTCCGTGTTCGCGGGCCACGTGCAGACGGGCGCCGTCGAGAGCGTGCTGACGCTGCCGCCGCTGCAGCGCGCGCGCGGCCCCGGGGGGCTGTTCGTCTCGCTGCCCCTGGCGCTCGGCGCCCACTTTGACGGCTTCACGGCGGTGGCGCTGCGCGTGGGCGCGCGCGAGCTGATCTTCACCTACGACGAGCTCCTGCCGGCCCGCACGCGCTACAACGTGGACGGCGAGCGCCTGGAGCGCCTGTGCCGCCAGTTCGCCAACTACGCGCGCGCGCGGCGCGTGGCCCCGGAGGTCGCGGCCGCGGGGGGCCACATCGACGCGCTGCTGCCCCCGGCGGCGGCCACCATCGACGGCGAGGCGCAGCTGACGCGCGGGGGCTTCGACGACCCGGCGGCGCCGCACGCGCGCGACGCCGACCGCGAGATCCTGTCGCTGGTGCGCCGCGCGGCCGAGCTCGTCGCGGCGCGGCACCCGGTGCGCAGCCACGTGGCGAGCGGGCTGATGCAGGGCGCCCTCGCGCGGCGCGGCGGGGACGGCGGCGGCTCCGGGGCGCTCGAGGCGGCCGCGGCGGTGCCCGCCGCGCCGCGCGAGGCCGCGGGCGGCGGCGACGGCGGCGGCGCCGCCTGGCGCGACGAGCTCCTGCTCACGCCCCAGGACCCCCGGCCGCTGACGGCGCTCGACTGGCTGGACGCGGGCTACGCCGCGCTCGCGGGCGGCGACGCGCCGGCGCACGTGTGGCGCCGGCGGCCCGTGTCCCTGGTGGCGCGCCGCCACTACCAGACCGGCGAGACGTTCGTCGTCGTCGCCTACGAGCACTCCACCGCCTGGGGCGGCCGGCGCGGGCCCGCGAGCGAGCCCCTGGCGCGCGTGCTCGCCGAGGAGTGCGAGCGCCACGGCGTCGAGCACCCGCGCGCCCTGCCCGCGGAGGCGCGGCTGGAGCTGGTGCGGCGGCACGCCGAGCTGGCCGTGCCGCTGGGGGACGAGGAGCCGCCGCTGCCGGTCTTTGACGCCACCGCGGAGCTGGTGCTGCTGGAGCGCTTCCGCAACGCCTGCGTGCGCGCGCTGCTGGCCGGCGTCCGCGAGTCCGTGCGCCGCGAGCCGCGCATGCGCCAGATCATCGAGTTCGCGATCCGCCCGCGCGACCGCGAGGCCGTCCTCGACGTGGCCGGGCGCGCCCCGGCGCTGCTGGACGCGTTCGCGCGGCGCCTCGAGCAGACGCCGGCGCGGGAGATGGTCGACTCGGGCCTGATGACGGCCGCCGCGGCGCACCTCGCCGCGCGCGCCACCGCCGGCTACGTGACCTTCGAGAGCGGCCCGCTGCTGGGCGGCGTTTTCCTCTTCGACTACTACAGCGCCGGCGGGGAGGTTATAAGGGTGACGCGGGCCCCGCCGGCCGTGGCAGTGGAGCCGGCGACGCGCGGGCAGTTCGCGTGCCGCTTCCGCGGCGCGTCGCATCGCTGTCTCCCGGGCGAGAGCTACGCGTACCTCTGCGTCGGCGTGTCGCGGGACCTGCGCGCGCTGGTGGTGCTGCCCGGCGGCTTCGGCTTCTTCGCGGCCCTGCGGCTGGAGTGGCCCGCCGCGCTGGTGGACCCCGTGCTCGAGCGCCTGTGCCGCCGCGTGTAGCGAGGGTGGGCGCGACGAGCGCCGCCATGGAGGTGGCCGCGGCCCTGACGGAGGACTTTGCCGCCTGGCGGCTGCTGCGCTCCGACTCGCGCGTCAAGGTCTACGCCGCCCTCGGCGTGGTGGGCGCGCGCCTGGCCGCCGTGGCGCCGGGCGCCGCGACCGTCGACGCGCGCGTCTACCTGACGCGCCCGCGGGCCCTGCGCCTGGCCCAGGGCCGCTTCCACGTCGTGGTCCTGCTCAACGACGCCGCCTACGCGCTGGTGGCGGCCGTGACCACGACCACGCTCCGCGGGAGCGGCGGCGAGCTCGTGCGCCTGACGCTGGGCGACGCGAGCCTCGAGGCCCTGCCCGCCGACCTGCCCGTCGCCGAGCCCGTGCCCGCCGCGCCGGTGAGCCGCCTGGACCTGGACGCCGCCGAGCCCGTGTCCGCGGCGCCCGCGCGCCGCCGCGAGTGCGTGGTCCTGGCCCCCGGCGCGTGGTGGTCGCGCGACCGCATCTACTTTCTGCAGATGGACCCGGCGCTGCTGGCGCTGTGCCCCGCCGGCTGGCGCGCGCGCCACCTCGGCGCCGTGCTCGCCGGGCTGCTGAGCCCCCGCGACGACGACGGCGGCAGCTGCTGCCGCGAGTGCCGCGTGGACCACGTGGACGCGCTCAACGCGACGCCGCACCCCGACGGGGCGGGCGCGGCCTGCCTGTGCGCCGCGCCGTGCCTGTGGCGCCAGGCGGACAAGCGGGAGCTGCGCGCGAGCGACTCGGGCCTCTTCCGCGTGCTGTTCCTGGACGCCGTGCGCTTCGTGCGCATGCTGCCCCGGCGCAAGATCGTCGACGTCGCCTCCGAGCTCATCGGCGGGCTGGACGCCCGCGGGCGCCACGTGGTGGTGAACGACGCCGGCTGGCGCCTGGTGGCCCTGGACCCGGACGCGAGCCACGCGCTCGTGTGCGGCTGCCCGCTGCTGCGCGCCCTCTGCGACCCCCCCTCCCGCGCCATCCCCGAGCTAATAAACGACTATTGAAATAAAAAAATGGGTACTTGCGTTTGTGTTGTGGCTGGAGGCGAACACGATGGTGCTGCGCGCGCCGTCGGGGAAGGTGACGGTGATGTTCTCGCCCTTGACGTGGTCCACGCGCGCGTCGCGGCACCAGCGGCGCAGGCGCGCGTGGATCTCCTCGAAGACGGGCTCCGTGGCCTTGCGGATGTGGGCCGTGTAGCCGACGCGGATGCCGCGGAAGGTGGCCAGCGCGAGGGCGATGAGCGGGACGAGGAACCAGGTCTTGCCGTGGCGCCGCGGCACGAGGAAGACCGTGACGCGCTGGCGAAAGTGGCGCACCGCGTCCTCGGAGAAGAGCGGCGTGTCGAAGGCCGCCAGCAGGTAGGCGGTGGCGCGCTCGGCGTGGTCCCCGAGCAGCACCGTGGCCAGAAAGTACACCGCGTGCATGAGGATCATCTTCTGGAAGAGCTCCAGCGTGCCGCGCCCTCCGCCGCCGCCGCCGCCTCTGCCCTTTGCCCCGCTTCCAGTTCCACCTCCACCGCCGCCGCCGTCTCCGCTCTCGCCGCGGCCCTCCTCGGCGGCGTCCTCCGGCTCGGGGCGGCCGGGCGCGACCCCCGCGCCCCTCTTCTCCGCGGCCCCGTCCCCATCGTCCTCCGCCTCCTCGTCGCCGTCGTCGTTGGCCCCGGTCCCGGCCCCGGCCGTCCCGAGGGCGGCCGTCGCGGCGTCGCGGAAGGAGCGCCGCGACAGGAGCGCGAAGCGGTTGACGAAGCGCCGGAGCTGGCGGAAGGCCCCGGACGACCGCATGGCGTCCACCGCCGCCATGGCGCTCGTGTACGCGTTGCGGTGGAAGGCCAGGCGCTCGCCGTCGTAGGCGGCGAACTCGAGCGCCTCGGCCGCGCGCGCGAGCGCCGGGTCCACGGCGGCGCGGCCGCGCGCGCCCCCGAAGAGCGCGGCGCGCGCGAGCCCGGCGAAGATCCGCGCGCTCTCGCAGCAGTTGTGCAGCGTGCCGACCGCCGGGACCACCGTCTGGTGGCGCTGTGGGGCGGCGATGGCAAAGTTGAAAAAGCGGGCCTCCTCGGCGCAGTCGCGCTCACTGGCCTCGCGCTTGCGCTTGAGGTCCGCGTAGTAGCGCCGCGTCGCGTCGCCCAGGCCGAACATGTTCGCCTCGGACCGCCGGGAGCGCCGCGTGCGCCTCGCGGAGGCCTTTCAGCGCGAGAGCGTCTTCAAGGCGCGCACCGTCGAGCTTCTGCGCGGGCGCGCCGACAAGAAAAACCCCGAGTTCGTCCGGGCCTTTATGGCGGCCAAACAGGCCCGGCGGGATGTAGAGCGCCACCTGCGGCTGGCCGCCCGGGTGGAGTCTGTGGAACAAAAAGCGCGCGCGCTGCAGGCCCGCGTGGAAGCCCAGGCGGCCGTCCGCGGGGTCCTGGACAGGCACCGGCGGTTCACGCGGGCGGACTTTGCCGAGGCGCTCGACGCCGCGGAGGACGCGCTCGTGGCCGGCGAGGACCGGCTCGACGACGCGGCCGCGCTCGACGAGGACTGGGCCGGCGGTGGGGACCCTGACGAGGACGAGGGGGAGGAGGCGGACGAGGCCCTGCTGACCCAATGGCTGCTGGAGGAGGCGGCGGAGGCGTGAGCCGGACCGCGCTGGCGCGGCCGCCCATCCACCGCGGCACGTCCGCGCCCTGCGGCGACGGCGACGGCGACGAGGCGTCCCGGCTCCTGGGCCGCGCGCAGCCGCGCGAGGCCCCCTACCTGATCCCGCGCCCGGACGGGGACCTCGCCGTGCCGGACGACCTGCAGTACGCGACCTTCGACCTCACGGGCGACCCCGTGGCCGTCGGGGCCGGCTCGTACGGCAGCGTGCTCGTGTACGGCTCGGTGGCCGTGAAGACGCTCCGCGCCGGCTTCGGCCACGAGGCCGTCATGACGCTGCTGGCCGCGGAGGCGACGCGCTCCGCCGGCGTCCGCGGCGTGGTGCGCCTGGTGGGGCTCTCGGCGCCGCTGCGCCAGCTCGTGTTCCCGGCCTACGAGATGGACATGGACGCGTACCGCCGCTCGCTCACGGCGCGCCCCGGGCACGTGGTGCACGCCCTGGGGCGCGTCTTCGCCGAGCTCGGGCGCGCGCTCGTGTTCCTCAACAGCCGCGGGCTCAGCCACCTGGACGTCAAGGGCGGCAACATCTTTGTGCGCACGTGCGGCAACATGGTCGTGACGGCCGTCATCGGGGACTTTAGCCTCATGGCCCTCAACTCGCGCAGCACGCTCGCGGACCCCCGCTTCCGCCTCGCGCGCCGCAAGGCGCTGAAGCTCACGTCGCTGGCGCGGAGCCCGCCGACGGGCGTCCTCCTGGGGCATGCGCGCGACCGGCCCACGGGGGTGCTGATGGACTTTATTAACGGGCGCCCGCCGCCGCCGGGCCCCCTGCCGTACGAGGTGGGCCTGGCGATCGACCTGTGCGCGCTGGGCCACGTGCTGCTGGACGTGGCGCTCGGCCTGCGCCCGCAGCGCGGCCAGACGCTGACGCGCGAGTACGCCGTGGAGGTGCTCGCGCGCCGCTGCGTCCTCTTCGCGGCCCTGCTCCCGCCGGGCAGCGGCCCCGCCGCCGAGGCCCTCGCCGAGGACATCCTCGAGGAGGAGCTGGCCGCCAGCTTCCGCGAGTGCGTGGCCAGCTCGCGCCCCGGCAACCAGCCCCCGCGCACGGTGGCCCCGCTGCTCGAGCTCGTGGCCCGGTTCTGCGGCGAGGATGGCGGCGCTCGTTTTGCCGAACTCGCTGCCTGAGGAGCTGGCCGCGCGCACCTTCCTGCGGTTCCTGCGCGCGGCGCCGCGCCCCGCGGCCGGCGGCGCGCCGCTGGCGTACCGCCTGGCGTACGTGCACGACCTGCTCGTCGAGCTGGCGCGCCACGGCCTCGCCGCCCCCGACGCGGCCGCGGCGGCCTTTGGCGGCGCGCGCCCGCCGCCCGCGCCCGCGGACGTCCCCGCCGCGGCGGCGCGCGCCGCCATCCTGACGGTGGAGGCGGCCACGCGCGCGCAGAGCGAGAGCGACCTGTGGACGCTGCTGCGCCGCGGGCTGGCCACGGCCTCGACCGTGCGCTGGGGGGCGGACGGCCCGCGCTTCCCGCCGACGTGGTGCGAGGCCAGCACGGCGCGCTGCGGCACGCCCGACAACGCGGCGCTCATCTTTGGCCGCGTCAACGAGCGCGTGGCCCGCGCCGCCGTCGCGGCCCTGTACGCCGAGGCGCCCACGCCGGACCTGCCCGCGGCGGCGACGGGCGGCGACGAGGGCGACGGGGACGGGGCGAAGGAGGAGATGTTCACCTTCGACGAGACGGGCGCGCCCCCGCCGGGTCACGACCTCTTCTCGTGCGGGCTGCTGCTGGACCCGCGCACCGGCATGGTCGGGGCCTCGCTGGACCTGCTGGTCTGCGACCGCGACGCGCGCGGGCGCCTGGCGCCGCACCGCACGCAGACGGAGATGCTCTTCTTCGAGATCAAGTGCCGCGCCAAGTACCTGTTCTCGGCGGACGACGCGAGCCCCACGGCGCGCGCGTACGCGCGCCTGCTCGGGCGCCCCGACGCGGACACGCTGCGCGGCTTCCTGAACTCCATCGCCCGGCCGGGCGTGGAGTTCTTCGAGGGCGCCCCGGGCCCCGGCGAGGCGCTGGCGACGGCCGACCCCGCGTGGCGGCGCGCGGGCGCCGAGGACGCCCCCGCGACGCGTCGGAGGTGTGGCGCCTTCGACGCGCGCCACGTCGCCGCGAACGCGCACGCCCAGTCGGAGGTCTGGCTATTTAGCGACCCGGTGGACGGCCGTCAGGACATTGTGCCCTGGGCCTCGGGCGAGCGCGCGCTGAGGGTGCCCGTGTTCGCGAACCCGCGCCACGCCAACTTTCGGCAGATCCTGGTGCAGAGCTACGTGGTCGCCGGCGTCTTCCCCGACCGCCCCGCGCGCCCCCACCTGGCCACCTTCTTCGGGCGCCGCCGCCGCCCCTGCGAGCAGAACCGGACGCTCGACCTCGCCTCGCTGTGCGACGTGCCGCCCGCCTGCGCCGTGCCCGTGCTGCTGATCGTCACGCCCGTCTCCGTGTGCGAGGAGGCGTTCGAGGACCTGCGGACGCGCGCCGAGGAGGCCTTTCGCGCCACCGCGTCGCGGACATGGGACAGTGCTGCTGCCGATTCTCCAGCAACCGCGTCGTGACGAGCTCCGGCGACGTGCTGACCTTCGACGCGGACGCGTTCGAGGACTTTGAGCTCGAGCCCATGGTCGGGGAGCCCGGCCCCGTGCGCCCCAAGGCCCCGTACCGCGTGTCGCGCGGGAACCTCCGTGAAGCCTCGCGGGCGTACTGACTGCAATAAACCCGTTTGTCATACTATCGCATCGTTCCCGTCTGGTGTCTCGGGGCGGGAGGGACGATGGGACGGGGGAGACGGAGGACGGGGGAGACGGAGGACGGGGGAGACGGAGGACGGGGGAGACGGAGGACGGGGGAGACGGAGGACGGGGGATGTGGCGGGTTGGGTGTGAGACGATGGCCGAGGACGGGGGAAGAGGGCCGTGGGGCGGGGCGAGATGGAATGGCGCACCGGCCGCAAAGTGGGACGATGGGGACGGGCCTCAACGCGGGACGAGGGCGGCGGGGCGGGACCGGGGACAAGGACAAGGGCGGCGAGAGACGGACACGAGAGGACCATGTGCGATCGAAACGAACGTTTATTCAAAGCCGAGGTTCTCGTACACGACCTCGTCGCCGTCGTCAAACTCGTCTTCGGTCATCCCGGGGGCCAGCAGGTACTCTTCGTTGCCCCGGCCGTGCAGGCGCTTCTGCAGGTACCGCCGGGCCGTCGTCCGCGCCTTGTCGACGTTGGCGTAGAACCGGCTGCCCCGGGCGCGGTGGGCGATGCAGGCGCGGACCAGGCGCACGACGATCATCGCCAGGGCGATCAGCGACAGGACCGCCAGGACGGCGCGCGTCCCGATGACGACGCCGTGCGTCTCCCCGAGGGCCTCGCTGAACTTGGCGAAGTAGCGGTGGGCGGACACGCCGAGGGCCGCGCTGGCCACGAGCACGCAGAAGGCCGGCGTGGGGAGCACCTGCACGTAGCCGGACACGACGACCTCGACGAACGCCATCAGCGCCAGCGCCAGCACCGTGAACACGGCCAGGGCGACCTCCGCCGTCTTCCAGAGGCTGATGTGGAAGCTGTTGGCCAGGAGCACGCCGAGCATCAGCCCGACCACGGCCGCGCCCAGGCCGACCGCGCCGAGGACGACGTTCGTCACGACGGCGCGCGCGTGCACCGCCACGCGGCGCAGCGGGGGGCTGGCCGCCAGGGCGGCGCGCACGTCACCCGCGCCCTTGCAGGCGTGGCACGCCCCAAAGAGGGCGAGAAACACAAAATGGGTGATGTAGGCGGCGGCGGCCAGACCCGCCTGCTTGTGGGCCATCAGCAGCACCACGGCCTGGAGGGTCCAGGCGCAGAGCGCGCCGAGCATCAGGGTCCCCGGGGCGGCCACCGTCGACGCCGCGTAGAGCACCACGCTGGGCTGGAACGCCAGCCGGCCGGCCTCGCGCCGCAGCACCACGGCCCCCGCGACCGCGTACAGGGCGTGCGCCAGCAGCACGGTGGCCGTGAAGCCGAAGAAGCTGGTCACGGTGGGCGTTTCCAGAAACAGGGCGGGGGCCACGAGCGGCTGGCGCGTCCACACGCCGCCGGAGCACGGCCTCGGCGTTGCGCGGCCCGGACATGGCGGCGGACGCGGGCGAGCGCCGCGGCGCGACCGAGGAGGACGGCGGGGCGTTCGCCTCGAGCGTGTCGCTCGCGCGGATGCTATACGGTTGCGACCTCCCGGCCGTGGTGCGCAGCCGCTGGCCGGGCGTCTCCCTGGACCTGCAGCGCGACGCGCCCGTCGAGCTTCCGTCCCCGCACGATACCGCCTGCCGCCGCGTCCTCGTGGCGCGCGCTCCGATGGGCTCGGGCAAGACGACGGCGCTGCTGAAATGGCTCTCCGCGGCGCTGGCGGCCACTGATATGAGCGCGCTGGTCCTGTCCTGCCGACGCAGCTTCACCCGCACCCTGGCGAGACGAATGGACGACGCGGGCCTGGGATTTGTGACGTACTTCGACTCGGACGCATACGTCATGACGGGCCGGCCCTACCGCCGGCTGCTGGTGCAGATCGAGAGCCTGCACCGCGTGGACGAGCACCTGATCAACAACTACGACGTGCTCGTCGTCGACGAGGTCATGTCCACGCTGGGGCAGCTCTACTCGCCCACCATGGCGCGGCTGGCCCGCGTGGACGCCCTGCTCGCGCGGCTGCTGCGCGGCTGCCCGCGGGTCCTCGTCATGGACGCCACGATCAACGCGCAGCTCGTGGAGCTGCTCGTGGAGCTGCGCGGCGAGCCCAGCGTGCACGTGGTGGTGAGCGACTACGCCACGGCCGCCTTCGCGAGCCGCCGCTGCCTCGTGCTCCGGCACCTCGGGGCCGAGGTCGCGGCGGGCGCGGCGGGCGCGCGGGAGGACGGCGGCGGCGACGGGAGCGAGGACGCGGCGCGGGCCGGGAGCCCCGCCCCGGTGACGGCGGCGGCGACGACGACGGCGGTGGAGGCGGCGGGGGCGGCGGGGGACAGCTTCTTCGGCCTCCTGGGCGCGCGCCTCGCCGCGGGGGACAACGTCTGCGTCTTCTCGTCGACGCTGGCCTTCTCCGAGCTCGTGGCGCGGTTCTGCGCGCGCTTCACGCCCTCCGTGCTGGTGCTCAACTCGCAGCGGCCCCCCGAGGACGTGGGCCGCTGGGCCGTGCGCGCCCTCGTCTACACCACCGTGGTCACCGTCGGGCTCAGCTTCGACGCGCCGCACTTTCACAGCATGTTCGCCTACGTGAAGCCGATGGCGCACGGCCCGGACATGGCCTCCGTCTACCAGTCCACGGGGCGCGTGCGGCGGCTGCTGCGCGACGAGCTCTTCGTGTACGTGGACGGCTCCGGCGCGCGCGGCGAGCCCATCTTCACGCCCGTGCTGCTGAACCACGTCGTGGGCGCGGGCTGGCCGGCGCGCCTGTCGCAGGTGACGAACCTCGTGTGCGCGCAGTTCCAGCGCCGCTGCCGCCCGGCGTTCGCGGCGGCGCGCGGGATGCGCCTCTTCTCCCGCTTCAAGTTCAAGCACCTGTTTGAGCGCTGCACGCTGACGAGCGTCAACGACAGCCTCAACATCCTGCACGCGCTGCTGGAGAACAACCGCCTGCGGGTGGCGCTCGAGGGCTGCGAGCCGCCGCTGACGGCGCGCGCCTTCTGCGACTTCCTGCGCGACGCGCGCCTGGACGCCTTCGCCAGCCAGCAGGTGCTGCGGCAGCTGCGCCCCCCGGACCGGCCGGTGGCGGCCGACATCGCGGACAGCGGCGAGGTGGCCACCTTCGTGGAGAAGTACCTCGTGGCCGACGTGCCCGAGGACGAGCTGCAGGAGCTGCTGCGCGCGCTCGCCAACCCCGTGACGCGGGAGCAGTTTGTGGGCCTGGCCGTGCTCGGCGCGTGCGCGCGCGTCCCCGAGGCGCTGCGCAGCGAGCGCGTCTTCGGGGCCGTGTACGGGCACTACGCCTCGGGGGCCGTGCCCGTGGTGGCCGACGGGCGCCTGGAGCTGGCGGCGCTGGCCCCCGACTTCAACGTGCCCGCGCGCTGGGCCCTCACGCGGCGCTGCGCGCGCGTCGCGGAGGCCGCCGGGCTCTTCGAGGGCGCCTCGCCGGAGGTGGACTCGGCCGCGGTCGCGGCGGCGGCGGCCGACGCCGAGCTGGCGCCGCTGCTGCTCGAGGTGCTGCGGTGCCACGTGCTGGACGCGACGACGGCGGCGCGGCGCCCCGTGCGCGCGGCCCTGAGCGCGCTGGGGGCGGGCGGGGGCGCGGGCCCGCTGAGCCGCGGGCGGCACGCCGCGCTCGTGTTCAAGGTGATGTGGGAGGAGGCCTTCGGCGTGCGCGTCGGCCGCAGCCGGCAGACCTTCCCGGGGCCCACGCGCGTGAAGAACCTGCGCAAGGCCGAGATCGCCGCGCTGCTGCGCGACGCCGGCCTGGACCCGCCCGCGGGCGCCACCCACCGGCAGCTGTACGCCCTGCTGATGGAACGCCGGGAGGACTTTGCGGGGGAGAGGTATAAACTGCGCCTGCCCGCCTGGAGCCGGCTCATGTACCTGACCCAGGGCGGCTTCGACGCGCCGCTGGACGCCGCGCTCTCGCTCGTGCCCGCGGAGGCGTGGCCGCGCATCGAGGGGGCCGTGGACTTTGCGGCGCTATGACGGCCGCGGCGGGGGAGCGCGTGTGCGCGGCCGGGATCTACACGGCCTGGACGGAGCCCGGCGCGCCGGGCGAGCTCCACGCGCTGGCGCACCTGCTGTGCCGCGACGGGGCCGGCGCGTACGCGGCGCGCTTCGTCCACGTGACGGCGGAGCCGTGGACGGGCTCGGCGGCCGCGGTGGCCGCGGCGCTGCGCGACGCGAGCGGCACGGGGGCGCTGGCGGACCCGGCGCTGTGGCGCGGCGCGCACGGCGCGGTCATGGCGGCCCTGCGCCGCGCCCTCCCGGCGCTGGCCTTCTACGAGCCGCTGCGCTTCGAGACGGACCCGGCCACGAACCTGGTCACGGCCGCGACGCCGGTGGCCGGGGGTGGCGGCGAGGAGAGGGGCGAGGGGGAGAAGAGCGCGGAGGAGGACGGGGACGACGAGGACGAGGCCGGCCCGCGCGCGGGCATCATCCACGTGGACGCCGACGTCGTCGTGGACCGCGACGCCGCGCGCGCGCACGCCACCGAGGCGTGGCTGCCGCACGCCCAGCTGCGCGCCATGGCCGGCTGCGCCGCGCTCTCCGTCGAGATCACGACGCGGCGGACCAGCTTCGCGCGCGCCTACGCCGAGGCCGGCGCGCCGCCGCTGAAGCGCCGGGGCGAGATGGGCGACCTCTTTGACGTGCGCGAGACGGTGCTGCGGCCCCGGGGCCGGCGCGTGGCGGCGCGCGTGCTCGTCCCGCGCGGCTTCGACTGCCTGGTGGCCCACGGCGTGAGCAACGCGGCGCTGGTGGCGCTCTACCGCCGGTGGCACGCGGCCGCGTACGGCGCGGGCGACGCGACCCCGCCCGTCTTCGCCTTCCTGGGGCCCGAGCTCGCCCGCGAGGGCGCGGACGAGGACTACTACTGCGCGCTGGGCTTCCCCCTGTTTGCGACGATCAAGGTGGCCGTGGCCGCGCCGGAGGCGGTCCGCGGCGCGCTCGCCGCCCACCGGCTCACGGACGGGCTGTGGCCCGCGCTCGGCTTCCGCGCCTTCCACGCCCTCGGGCCCGTGACCTGCGGCCTGCGCCTGCGCTGCGACGTCTGGCCGGCGGGGCGCGTGACCAGCGCCGTGGAGCACCCGTGCGCCCTCCGCGGGGCCTGGCTGGCCAAGTTTGACTTCGCGGCCTTCTTCCCCAACCTGTTTCTGGCCCTGTGCCCGGGCCACGCGCGGCTGCGGCGCGCCGTGCGGGCGCGCGCGGTCAAGCCGGCGCTCGTGGCCTTCTTCGGCGGGCTCAAGCACACGTGCCCGGAGGCGTACCACAGCGTCATCGCGCTGGCCAACGGCGTCTCGCGCGCCGTCGAGGCGGCCGCGGCCGAGGCCGGCCTGGTCACGTGCGCCTACGTCAAGGACGGCTTCTGGGGCGTGCTCGGCGGCGTGCCCGCGGACGCGCCGGAGGCGGCCGCCGCCGCCGCGGCCGAGCGCGTGCGCGCCGCCTGCGAGGCCGCCGCGGCGCGGCACCTGGCCGACGCGGGGACCGCCGCGACGGACGTGACGCTGCGCTGCGAGGGCGTCTTCGACGCCGCCCTGGCCTGGTCCTGCCACGCCTACTGGCTGCGGCGGCGCGAGGGCCCCGCGGACTTTGTCGGCTTCCCGGCGCGCAGCGGCTTCGGCCGCGCCGCCAAGGCCGCGCTGGACGCCCTGCTGCGCGAGGCCGTCGAGGGCCGCGCGGACGCCGGGGCCGCGCGCGCCGCCTGCGACGGGCTCGTCGAGCGGGCCTTCGCCTCGCGCCACGACCCCGACTTCTGGGCCGCCCCGGGGGACGCCCTCGCCCCCGCCGCGTGCGCCGGGGCGACCGCCGGGCGCGGCGGCGCGCGGTGCTCGCGGCGCTGCGTGCGCCTGCGCGGCGCCGGCCCGGTGCCCTACGAGCAGCTGCGCCCCCCGCTGATCCTGCCCTGGATAGACTGCCTGGCCCACATGGAGCCCGTCTTCGCGGCCCTCGTCGGCATGTACAACCGCGCGCTCGAGGCGCTGAGCGACGGCGACGCCCCACCGCCCTTTGTGTTTACATACACACCGGACGCGTTTCTGTTTGGTTAAACAATAAAGTCTGTTTATTGTCCGAACGAGCCACCCGGCGTCCTGTTGTCGTCCGCGGTCGGGGATGGTGGGGGAGAGGGAAGGGGAGAGGGAAGGGGGGGAGGGCGAGGGGGGGAGAGGAGGGGGGCAGATGGAGAGGGGAAAGGGGAAAGGGGGGGTCGGGGCGGGGAGGTGGAGGAGGCGGAGGAGGGGGGAGGCGCGCTTCACTCGTAGCGCACAAACATGGTCTTCTCGGTGAGCGCCCGCGGCATCGTCGTCCACCAGCGGTACACGGCCTCGGCCATCTCGGGCGTGCGCAGGGGCGCGGTGAGCGCGCCGCCGGGGCGCGTCCGCACCAGCCCGTCGGGCGGCGGCGCGTCGCCCGGCAGGCCGAGCAGGGGCCCGAACCCGGCGGCGAAGAGGGCGTCCACGACGGGCGGGGTCCGGGGGGCCGACAGCAGGCGCTGGGCGATGGGGTAGTGGGGCAGCACGCGCGTCTCGTCGAGCGGGCTGAGGTGGGCCGCGAACATGAGCGTGTTGAGCGCGCGCGTGGCGCCGACGTGCAGCAGGCGGCGCAGCTTCTGCGCCGGCGGGGCGCGCGCGCGCTCCAGCCACCGGCCCAGGCGCAGGCACCGCGCGGCGTCCGCCTCGGCCGCGGGGCAGTTCTCCAGGAACATCTGCAGCACGCAGAGCGCGAACTCCGTCGGGTCCTCGGGGCGCACCAGGGCCAGGCGGTGCGGCAGCGCCACCGGCGGCACGGCCAGCGAGAGCACGCGGTCCTCGGCGGCCGTGACCACCGCGAAGGCGAACCCGCGGAAGGCCTCGCCGGCGAGGCAGCGGCGGCGGTACTCGTCGCAGGACACCGTCTCGCCGCTCTCGAGGCGCAGCGTCAGCGCGGCGCCCTCGACGCGCGCGTCAGTCACCGAGGCGCTCGTGAAGACCGGGATCAGCCCGGGCACCTCGCGCACCTCGCACACCAGCCGCGGGAGGGCGTGCCAGGCCAGCTCGTCCATCACGGCCTCCATCGCGGACCCGCTCGGTCGCCCGGCGCACGTCCGCCGCGAACAGCGCCGCGATCTCGGTCAGGTAGGCCTCGAGGCGCGTCTTCTTAAACACCACGCCGCACAGGTCCTCCTCCGAGCGGTACACCTCCTGGCTGGTGATGAGGAAGCCGAGGTGGCGCACGCGCAGGATGGAGAAGAAGTACGGCGCGAGGATGAGCGAGATGGAGCTGTTGGAGTACGAGACGGACACCTCCTGGCCCTGGTTGTTGGCCACGCGCGTCAGCTTGAAGCAGCGGATCAGCTCCTGCTCCCAGAGCCGCGAGAGGCGCTCCACGTCCGACTCGTACGGCGGCACGTAGCGCGACTGGAAGCTGTTGGCCACGTAGGCGTCGTCGCCCATGGCGCGCGTGATGTCGATGACCTCGTGGCCGAGGTCCCGCGGCGGCTGGCCGTCCGCGGCGCCGGCGCCCGGGCCGGGCGCGCGCCCCCCGGGGCCGGCGCCGCGGTCCGCGCCCGCCTGCGCGGCGCGCAGGGCCTGCTCGCGCAGGCGCCGCACCTCCTGCTCGCGCTCGCGCACCTGCTCGCGGAGGCCCCCGTTGTCCGCCTTCAGGCTCTCGATGGTCTTGAAGAGGTTGTTGACGTAGCCCTCGAGCATGCCGTTGATGCTGTTGACCACGGAGGTGCGGAAGGCGTCCTGCACCGGCTGCGCGCCGGCGCCGTGGTGCCCGAAGCCCGGCTGCGACGTGTCCACGCTGTCCAGGATGCGCGCCCCGGTCTCGTCGAGGTACGAGCGCACCGTCTCCGTGATGTCGCCGATGTGGCGCATGCCCTTCATGTTCACGATGAGGCGCACGAGGCGCGCCGCGGCCGAGCCCGCCGGGTCCTCGCCGAGCATCTTCTCCACGGCGCCGGCCGGGTGCCCCTCGGCGGGCTTGCGCCCGACGAGCACCTTCACCGGCGCCGTGTTGAGCAGCTGGCAGAGGCGCGCGTGCTCGCGCAGCGCGTGGCACGCCAGCACCTCGCCGTGCACGCGCTGCGCCGGCGAGTCAAAGAGCAGCTCGCCGTCGCGCCACAGCGGCTGCCAGAGCACCAGGCACTCCCCGCGCTCGCCCGTGAGCGCGTCGCGCGCCAGCACGCGCCGGTGCCGGGGCTCGTAGTAGAGCTGCACGCGGTCGTAGTCCATGATGGTGACGCCGCGCATGGCCTCGGCGAGCTCGGCCACCTCGTCCAGGCCCTGGCCCAGCACGCTGCCGGCGACGCGCAGGTGCCCGGCCAGGTCGTGCTCGTCCACGCAGCGGTCGCGCCGCCGCTTCCCCCCGGGGCGCGCCCGGCGCACCGGGACCACGCCGAGGCACACGATCCAGTCGATGTACTTGGCGAAGCTGGCCGTGCCGTTGGACTCGCTGGCCGAGAAGCAGGCCGCGATGCCGCGCACAAAGTCCAGGAGGGCCATCTGCAGCGTGCGGTACCAGGTGTCGAAGAGGCGCTCGGCCACGGCGGGCGCCTCGTCGCCGAAGCGCCGCGCCAGCGCCTCGGCGCCGAGGCCGCGCGCGCGCAGGTGCGCCATCCAGTCCCGGGCCACGTCCTCGTAGCGCGCCGCGTTGAGCGTGCGCGTGAGCAGCGTGGACTGGATCTGCCGCACGGCGGCCTCGGTGGAGCGCACCGAGTTGTAGATGCCCTGGCCCTCGGTGTAGCCCAGGTTGCCCATGAGGATCTCCTTGAAGACCATGGTGCGCGGCGTGGGGTGGATGAGGATCCGCGAGGGGGCGCCCAACAGGTTATTAGCCTCCTCCGCGGCGCCGCCGGGGCAGAGCCCGTCCGCAGCCGCCGCCGCCGTCGCAGCCGACATGGCCGCCGCCGCAGAGGGTCCGCGCGCCACCAACGCCACCTATCTGAACTTTACGTCCATGCACGGCGTCGAGCCGATCGTGGAGCGCGTCCGGGAGCTCGCCGCGACGCCCGCGGAGGCGCCGCCGCCGCTGGCGTGGTTCAAGTCCCTCGCCGCGGCGGACAACCCGGTGGATATAGAGGCGCTGGAGCTGCCCTTCGCCGCGTACCTGATCAGCGGCAATGCCGGCTCCGGCAAGAGCACGTGCATCCAGACCCTCAACGAGACCATGGACTGCGTCATCACGGGCTCGACGCGCGTCGCCGCCCAGAACGTCTACGCCAAGCTCTCGGCGGCGTACTCGAGCCGCTACGTGAACACCATCTTCCAGGAGTTCGGCTTCCGCGGGAACCACGTGCAGGCGCAGCTCGGGCGCTACAGCTACGCGTGCCCCACGAGCCCGCCGACGGTGCGCGAGCTGCAGAAGCGCGACCTGGTGTACTACTGGGAGGTGCTGCAGGACATCTCGCGGCGCGTGCTCGCGGGGAGCCACGAGGAGTTTGCGCGCCTGCGCGCCCTGGAGCGGCTCACGGGGCGCGCGGCGGAGCACCTGGCCTTCGCGTGCCACGGCTCGCTGCCGGCGTTCGCGCGCAGCAACGTCATCGTGATCGACGAGGCCGGGCTGCTCGGGCGGCACCTGCTCACGGCCGTCGTCTACTGCTGGTGGCTGATCAACGCCGCGTACGACACGACGCAGTACGCGGCGCGCGCGCGCCCCGTGCTCGTCTGCGTGGGCTCGCCCACGCAGACGGACTCGCTCGAGTCGCGCTTCGAGCACGCGCGGCAGCTGTGCCGCGTGCGCGCCAGCGAGAACCTGCTCACGTACCTGATCACCAACCGCGCGCTGCGCGAGTACACGGACCTCTCGCGCAACTGGGCCATCTTCATCAACAACAAGCGCTGCCAGGAGTACGAGTTCGGCGAGCTCATGAAGGCGCTCGAGTACGGCCTCCCGCTGACGGACGAGCACCTGCGCCTCGTCGACAGCTTCGTGGTGCCCGAGGCCTACATCAACAACCCGGCGAACCTGCAGGGCTGGACGCGCCTGTACTCATCGCACCGCGAGGTCAGCGCGTACATGAGCCGCCTGCACGCGCACCTCAAGGTGGCGGGCGACGCGCAGTTTGTGGTCTTCACGCTCCCGGCCTACACCATCGTGCGCACGGCGGCCTTCGACCGGTACCGCGAGGCCACGCAGCAGCCGCACCTGACGCTGGACCGCTGGCTCGCGGCCAACGCCGGCCGCATCACCAACTACTCGCAGAGCCGCGACCAGGACGCCGCGGCGCTGCGCTGCGAGGCGCGCGCGCAGCAGGGCGTCGTGCTCGCCCGCTGCGAGGTCACGTACGTGCTCAACAGCCAGGTGGCCGTCACCACGCGCCTCAAGAAGCTGGTCATCGGCTTCAGCGGCACCTTCGAGGCCTTCGCGGCCGTCCTGCGCGACGACGCCTTCGTCCACGCGCAGGGCGGCAGCGCCGAGTACACGTACCGCTTCCTGTCGAGCCTGCTGTTTAGCGGCATGATCGCCTTCTACAACTTCCTGCAGCGCCCCGGGCTGGCGCCCGAGGCCGTCACGGCGGCCTACCGGCGCCTGGCCGCCGTGACGGCGGCGGCGCTGCGCGTGCCCGAGGAGCACTTTGACTTCTCGGGCGCGGCGGCGCCCGCCGCCCCCGCGGGCCCGCGGGCCCGGGGCCCGGGGGCCCGGGGGCGCGCCGCGTCCGCGCCGGCGGACGACGACGACGACCTCTTTGCGGCGCTCAGCGAGAACATGCTGGACATGCTCTACTGCCACTACGACTTTGCGCGCCCCGAGACCACGAGCGAGGTGTACGCGCAGTTCCTGATGCTCAAGACGCTCTTCGCCGACCGCTACGCGGCGCTCTCCGAGCTCTTCGGGCCCGCGTTCGCGCGCGCGCCCTTCGAGACGCACGTGGACAGCGTCTCGGTGCGCGGGTGCGAGGTGTTTGTGGGGGGGCTGCGCGGCGCGCTGCTCTCGACGGCGCTGCAGACGGACAGCTACACGCTCGTGGGCTACACGCACGCGACGGCGCCGGCCTTCGCCGAGGAGCTGGCGCGGCGCAAGCTGCACGCCGGCACCGCGGAGCTGCTCGCCTCGCTGGACACGCCGCGCGTGGTGCTGCGCGACCAGAGCGGCTTCCTGTCCATCCTCAACGTCAACCTCAGCGACTTTGTCGAGTCGCTGGACGACCTGGAGCTGGACATGGCCACCTACGTCGACTACGGCATCAGCTCCAAGCTGGCCATGACCATCGCGCGGTCGCAGGGCCTGAGCCTGGAGCGGGTGGCCGTGTGCTTCACGCGCGCCAACCTGCGCATGAACAGCGTCTACGTGGCCATGTCCCGCGTGATCTCGTCGCGCTTCCTGCGCATGAACATGAACCCGCTGCGCGAGGAGCCCGAGCGGGACAACGGCATCAGCGAGCATATATTGGCCGCGCTGCGCGACGGCGCGGTGCACATCGTGTACTGACCCGCCGTCCACCCGCCCGAGGGGGCCGAGGGACCGAGGGACCAAGCGAGACCGACCATGACGGCGCTGGTGGCCTACTCGTTTTACGAAATAAAGCTACCCGGCGGCTGGGCGCAGCCGGGCTGTGGGCAGACCGTGTGCGAGTACGAGCGCGGCGTCCGCGTCATGGCCACGGACGGGTGCACGCGCTGCGACGCGCTCGCCCCGGGCCGCGTCACGATCCAGCACGGGCCGGTGCTCACGGTCCTGGCCGTGGACGGGGAGCCGGAGCGGTGCTCGTACGTGTTCGCGCGGACCTGGCCGGCGGCCCCCGAGGGCGCGCTCGTGATGCCCTTCTCGACCTGGAGCTGCGCCGAGCGCTCGCGCCGCCTGCGCGGGCCCGCGGGCGGCCTGCTGGCCACCCTCGTCGCGGAGCGGGCCCTGCACGTCACCATCACCGCGTACCGGCCCGACGTGCTCCGGGACGCCCTGCGCGAGGCCCGCATCCTGGAGTGAGAGGCGGCGAGGGCCGGGCGAGCGGAGGAGGCCGCCGCCATCACCATCGCCGCCACCGTCACCATCCCCATCCCACTCCCATCCCACCCCGACAATAAACGACGACGGCCGCGCGTGCGCGGAAAGAGAGACCGAACCGGTTGTGTCTTTTGTCTGGTCCGTGGGCATCCGCCCTTTATTGATCGCGGTAGCAACAGGAGGCGGGGGGCGCGCGCCGGCCCCGGGACGGGGTCCGGGGGCGCGGGAGGGCGGCCGCCGAGGGGACGGCGGCGTGGGCCGAGGCGTGGACGCTGCGCTGGCGGGGTCTCGGCAGCTGGGGCGTCGGGGCGGCCGTCGCCGAGGCGTTGATGCTCCGTTGCCGGGGCCGCGGCAGCTGGACCGCCGGGGCGGCCGTCGCCGAGGCGTGGACGCTGCCGCGGCGGCGCGGGGGTGCGGCCGCGCACGACTCGGGTCCGTCCGCGGCCGTCGCCGAGGCGTGGACGCTGCCGCGGCGGCGCGGGGCCCGCTGCTGTTGTTGCTGCTGCTGCTGCGGATGCTGAGAGCGGTGGTGGTGCTGCTGCTGGTGGTGGTGGTGGGTAGGCTGGACGACGGTGGCGTAGACGGGGTCCGGCTCGGTCGCGCCGCGCTGGCGCGAGCGCCCGCGGGCGGTGCCCAGGCCGCCGGCGCGCAGGGGCGCCTCCTCGTCGGGGGGGGCGCGCGAGGACCGCGGGGCCCGCGTCTCGCCCCCGCCGGCGGAGACGCGGCGGGAGGCCGCGGCCAGGCGCCGGGCGGCCGTCTCGTTGGCCTGCAGGAACCGGTGGTAGTCATCGACCGTGGCCACGGACGCGCGTCCGAAGGTGCTCATGGCGCCGCAAACACGTTGTCCCGGTGCAGCCGCACCGGCTCGGGGGTGATGAACTCGTGGATGAACACCGGCGCGGCCGGCCGCGTTCGTCCGCTCGTCGGGCGCGCGCGGGGCTCGCGTGGGCGTCGGGCGCGCACCGCGGACCGGAGCTGATCGCGTACGCGCGCCGCGTGGGCTCTGCGGCACAGGATAAACATCTGCAGGCTTTTATGGCCGCGCCCGGGGGCGCCGCGCGGGCCGCGCGCGCCGCGCGCAAACGCGGACGTCTTGGTGCAGGCGACGGGGAGGCCGGCCAGGGCCGCGGCCAGGTCCCGGCGGATCGTGTCCGTCAGCCGCCGGCGCCCGAGCTCGTCCACGGACGCGACCATAAACAGCGTGTCAAACTCCACGTGCTGCGCCTGGCGCGGGCCCGCCGCCGCCGTCGTCGTCGTCGTCGTCCCTTCGTCCCCCCCATCTTCCTCGTCTTCCGTCTCCTCCTTCTCCGTCGCCTTCCCCTCCTCGCCGTCTTCCACGTCCCCCTCTCCGTCTTCGCTCCCGCGCACCCCCTCCCGGGGAGGGGGTGCGCTCTCCGGGGGCGCGTCCGCGTCCTCCGGGCACGGCCCGGGGGACTCGACCTCCTCCACGGCCCATCCCCACTCGCGCAGCACCGCCAGGACCGGCAGCGCGCTGGCCGGGGCGCCGGCGAGCGCGGGCTCCATCATCCGCTCTCCTCCGTCCATCCCACCCCCCTCCCCGCCTCCTCGTCCTCCGCCGGCGGTGTGTGCGCACGCCCGACTCAGTCCACGCTCCAGTCGACGGGGGCCCGGCCCGTCTGGACGACGTACGCGTTCGCCTCTCCAAAGTGCGGGCAGGTCCTGAAGGGCGTGCGGGCCAGCGGCGACGGGTGGCTGAAGGTGAGCACCTTGTGGCGGCGCGGGTCCGGCGCGCAGGCCTTTTGAGCGTGGGCGCCCCAGAGCATGAACACCAGCTTGGGGCGGGTCTCGCAGAGCCGCTGGACGACGGCGCGCACGAGCCGCGCCCAGCCGAGCGGGGCGTGGGAGCCGGGGACCCCGCGCCGCACGGTCAGCGTCGTGTTCAGCAGCAGCACCCCGCGGCGCGCCCAGGCCTCCAGGCAGCCGTGGGCGGGCGCGGGCAGCGTCGGGTACGTCGCCCGGACCGCCGCGAAGATGTTGGCCAGGCTCGGGGGGATCGGCACCCCGCGCCGGACGCTGAAGGCCAGCCCGTGGGCCTGGCCCGGCCCGTGGTACGGGTCCTGGCCGATGATGACCACCTTGACGTCCTCGGGCGCCGTGAGGCGCGTCCAGGCGAACACGTCCTCGCGCGCGGGCAGCACCTCCTCGGTCAGGCAGCGGCCGCGGTACTCGGCCAGCAGCAGGCGCGCGTACGGCTTGGCGAGCTCGGGCTCCAGCAGCGCGCGCCACGAGGGGGCCACGTCGAACTCGGCGGCGAAGGCGTCCCACGTGGGCGCCCCGGGGTCGGGGGCCGGGGCGGAGGCGTCGGCCCCCGTGGCTGTTGTCGCCGCCGACGCCGCGGGCGTGCCCTTCGCGGGCCGGGCGGCGGCGGAGGGTCGGGCCGGCTTCGAGGCCCCGGCCCCTGCCCCTGCCCCTGCCCCTGCCCCTGCCCCGGCGGGGGCTGCTGCTGCAGCGGCGGTGGTGGCGTTAGAGGCAGAAGCGGCGGCGGCAGGCACGACGAGGCGCACCCCGGGCGGCAGGCCGCAGGGTCTCTTACTCGGCGGGGGGCCCTCCATTATAGCGCAGCGGCGGCAGGGAGTCCTTGTGCACGGCCACCTCTCCGAGCTCCTTGAGGGCCGCCTCATTTAAGGGCTCTCCGACGGCCAGGGCCTGGATGGCGACAAAGGGGTTGACCAGGTAGGCGGTGCCAGACGCCCACAGGATGACCTCCGGCGGCGAGCAGTAGGGCTTCACGACCACGCTCGTGACGGCGTTCATGTCCGGCGGCAGCGAGTACGCGATGTAGGGATGGCAGCGCGATCCCACGGGCGGCTCGAGGTCCAGCAGCGGGTGATCCTCATCGTCCCACTCCAGCTCCTCGCGCCGAGGATGGCGAGTGGGGGGCGCGGGCGTGACCGGGGCCTCGAAGATGGCATCGAGCCCACGGGGGTTTCCCGCGACGCCGGGGCCAGGAATCCCCAGCGCCGCCGAAAAAAACACCAGCACCGCCACGAGCGGCGACATCGCGGCTGGTGTATCCTTTTTCGGCGCGGGGTCCCGGCTTTTATCTCCGGAAAGAGGAAATTGAGAAATAAACTAGACATCCGCATAATCCCCTTTGATTTACGGAGGGGAAATACGTGCCACGTGGCGGGGGGCGGGGCAACTGGAGCGTGGCGAAGCGGAACGGGGTGCGATGGGGCGCATCGAGGCGCGGACGCGTCTGGGGGGAGTCTGGGGGGAGTCTGGGGGGAGTCTGGGGGGAGTCTGGGGGGAGTCTGGGGGGAGTCTGGGGGGAGTCTGGGGGGAGTCTGGGGGGAGTCTGGGGGGAGTCTGGGGGGAGTCTGGGGGGAGTCTGGGGGGAGTCTGGGGGGAGTCTGGGGGGAGTCTGGGGGGAGTCTGGGGGGAGTCTGGGGGGAGTCTGGGGGAATTCCAGCTCGGCGCGATCGCCGCATCGCGCAAAGTCCGGCTGCAGTAAATTTACTGCGGATGCAGTTCCCGGGGCGGCCGCCCCGGCCAGATGGCGCTGCAGTATAGCTACTGCGGCTGCAGTTTACTACAGTTGCAGTACCGCGCGCCACCGCTAAATACTACAGTAGATTTACCGCGGCCGCGGTAAACTGCAGTTTCCTGCGGCCGCGGTAAACTGCAGTATCGCGCGGTAAATTGTAGTCTGGCAGCCGCGCGTTACTGCAATTAGCGGTGGCTCCCGACACTCTGGCCAATTGGTGCTAATGGGCCGTGATGGTCCACGTGGGGGTGATGTAACCGCCGGGCCCCGGTTGGGCACTCAGACAGTGGCCGGGCGCCAGGCCAAAGTGCTGTCTGAGTGCCACTTTATGACTTTGTTTTTCTCAAACAACATCAATTATGGATGCACATCGTGTATATAATCCCCGGTCCGCGCTCCGCCCACCCATCACAGCAGCCGCGGACGCTGCGCGCCGGAGCGGTCCATCTCGCCAGCCAGCCAACCAGCCGAGCCGCCCGGCCGACCCGAGAGCCCCGAGAGCCAGACTCCCTCAGCCATAGAAGACACCGGGCGGGAGAGACGGACTGAAAAAATATATCTTTTTTTATTTTGTCCGGGCCTGGAGACCCGCAGCAGGAGCGGAGGTGGGTGTGGGGCCGGGAGCCGGAGCAGGACCGGGAACAGGAACAGGAACAGGAACAGGAACAGGAGTGGGGCCGGGAGCAGGAGCAGGAGCGGGAGCCGAAGTGGGGGCAGGAGCGGCGGCGGCCGCAGCAGCAACAGGGTCGCCCCAGTCCGCGGCGAGGAAGAGGGAGCTCAGTCGTCGTCCTGGGTGAGGTCGATGAAGATGGTAGCGGAGCGGGGGGATCCCGACGAGCTAGACGCCGGAGGCCCGCCCCGGGGGGCGGCGGTCTCGGGGGCAGAGGCAGAGGGCGACGGGCGCCGCATCGAGGAGGAGGGTGAAGACGAGGGGGAGGAGCGAGCCGAAGCGGCGGTGTTCGCCGACCCCGGGCCGGCCCCGGCCCCCGAGGCACCATGCTGCGCAGAGGACCCCTCGCCGGACGATGGCGCCTCCGGAGTCTCGCCCTGGGCCTGTCCGCCCGTCCGGCCGCGCCGCAGGCACCGAGTCCGTCTCTGCGCGCGCCTCAGCACGGCCGCCCGTCGGGCCCTGCGCGGGGAGCGCCTGGGCGCCGGCCTCTGGTCGTCCGCGGACTCGGAGGCCTCCGTCAGATCCTCCGTGTGCACCCCGCTGCTCGAGGCGCCCGAGTCTTCCTCGTCGGGGGAAGACACCTCAGAGTCAGAGTGCGCCTCGGACTCGGACGTGTCGATATAGTTCACACCCTGGTGGCTCATCGGGGCTCGCTGCATCCGCCGCATCCACTGCGCCGATATGTCAAACAGCGTATCGACGAGGGCGTGGGTGTTTGCCCCAAACATGGGGAGCATGGCCTCGGTCACGCGCTGGCGGTTCATCCCGTGCTCCTGGATAATCTCGACGATGTTGTCCACGACGGCCTCGCGGATGGGGTCGCTCTCGATGACCGTCGAGACCTGCCCATAAAGCCAGTTGAAGATGGGGACTCTGGGGCGGGCGCGAGACCCAGACCCGGAGCCCTGCCCTTCGGCCTCCTCGTGGCGCACCTCCTCGGTATAGTCTTCACCCCAGATGACCGCGAAGCCCCCCCCTACCGGCTCATCCTCTTCCCCGTCGACATCCGTCGCCCCCTCCACGGGCGTCTCCACAAACGAAGCGTCGCTGTCCACGTGGTGGAGGATGGAGGTGACGCGGGTCTTGCACAGCGGGCAGGCGGTGCTCGTCAGGGTCCAGCGCTGGATGCAGTCCAGACAGAACTTGTGCATGCACGGCAGCGTCTGCGCCTCGGTGGCCGCGACGTCCAGGCAGATGGGGCAGTCCATGACGGTGAGTGGCGGTAGCAGGCGCTGCGGCGGTGGCGAGGGGCTTCTGGGGCGGTAGAAGCCAAAGATCCCCCAGGCGCTGGAAGCCTCGGCGGTGGTCGTCCGTCTCCGAGAGACCGTGCAGCCCATGGTGTCGAGGGCCCCGTTTTTTAACCGGGTGCCCGGCCCCTTTGTTTGTCTTCGCCCGGGGCGTCCACGCCCCCTTCCGCCCGCCCCGTCGGGGCTCCCCACGCCCCCTCGGGCCGTCCCGGGACGATGGCCCCGGCGCGCGCCGTCGTGGGAAGCGGGCCCTCCCGCGGTGGGTCGACGGCTCCTGGGTCTGAAAGCGGCGCTACGGATCCCCCGCTCTCACCCCTGGGTCCGTCGCCGCCCCGCGGTGCGTGCGACGCCCGCGAACCCCCGGCCCACAACACACCGGCACCACCGCCACCATCATCATCCCCCCTCTCCACAGACACGCTCGCACTCGGGCCACACGCTCCCGTCCTCCGCCGCCACCCTCCCAGCCTCCCAGCCTCGCGGGTCCTCGGGACGACCGCCGCTCACCCCGACGGGGGGGGGGGACGGGGACAGGGAGCGAGGGGGGGAGGAGGACGGGGGGGGGGAAGAGGGGAACGCGAGCGCGGCGCACCCCGTCGCCGTCCGTGGTGGCGTGCCCGCCACCCCGTCCGTCCATCCATCCGTCCATCCAACCCCCTTCTCCTTTTTCCGCCTCCCGTCCCACGGCACGGCCCCATCGACGCCACACCGCGCGGTGCGCGCTCTCCAGCCGTCCCATCCCCCCACACCCCCCACGCTTCCCCCATTCGCTTCTCATCCCGTCAGACCTGCGCCATGGAGTTTCGAGGACGGGCGCGGGGCAACGGTGGTGAGAGGCGAGCCGGGGGGGTCGAGTGGGCCTCGCACTTTACGCGCACACGCCCCTCTCGCGCACCACACACCACCGCACATCGCCGAGGTCGGGCGAGAGAGGGCGCCCTCCCCGCGACCCCGCTCGCCCCGGTCCCGACGCCCTCGCACCCCCACAGCACCCCCTTTCACCCCCCCCCGCTTCTCCCCGCTCTCTCCGCGCGATCGCACACACAACACCCGCGCGCATGCTCGGCTTCTTCACACGGAGGACACCATGGGCAGCAGATGGTACGGTCAGCCGCCGGCCGCCGTGCGCATGTGTCGGGCTGTCGCGGCCAAGAGTATCAGCGTGGCTTTTTTGTTGGTCAGGGTGTGAAAAAGGCACGCTGATGCGTCCGCCGCTGCAGCGGAGCTCCTCGGCAGCGAGATCATCGACCACCACCACCACATCGCTCTTCTCGCCCGCGCTCCCCCACCGCCCCCCCCCCGCCATCGCCCGCGCCCCTTCCCCACTCCCCCACCCCCGCCCCCTCTCACCCCACCGCCACCCCACACCATCCCCCACCCCCCCGTCCACCCTCACCCCCCATCGCGTCGCGAGCGCTCTCACACATGCTTACATGCACACGCGCCCCTCCCTCCTCGGCGCCCCCGCGGCGGGACCGTCGCAACCCTCTCCCCCGCCGTCCCACCCGTCCGCGGCCCTCCTCTCCTTTTCCCCCTCCCCTTCCCCTTCCCATCCCCGTTCCCCCCTGTTCCCCATTTCCCCGCTCCCTCCACCATCGACTCTCATCGACGGGCTTCTCCGCCATCATCACCACCAACCACCAATCACCATCTCCCGCCGCCGCCCGGACGGCGACCCGCGGTGCTCCCGGGGGGCGCGTTCCCACTCACAACCCGGTGGTTTCGATGGCCGCCCCCGGGGGGTTGATGGGGATGGGCGCTCGGGGGTGATCGTCTGCTACCAAACCGCACCACCGTCCCCCTGTCCCTCATCCACTTCTCGACGGCCGGCTCCCCGCGGGGCGGGGGCTGCGGGGGGGTGGGTGGGTGATAGAGCAGGCGGGCGGGTCTGGACGGGCGGAGGGGGGGGGGTCCCGGCCCCTCTCCCTCCCCTCTCTTCCCCCCTCCCCTACCCTCTCTCCGTGCCGACTCGACGAGCGGGGGCGAGGCAGGGTGCGTACCGACCCGCCTACCAGGCAGGCTAGAAGCCTCACGCTGCTGCTTGGCAGCGGGTGGGTACCCAACCCCTCCCCTCAGGACGCGACCGACCGGTGTGGAGAGAGAAAGATGCCCGTCCCCCCTGCCCCCACCATCCCCGATTTTTCGGTTCCTCTCCCCGTCGCGCCGTCGGGCCCCTCGTCGCCGGGGGTTTTTTTGGTGGAAGGCCCACCGGGGTGAGGGGGGGGGGGGGGGTTGGTGGTGTGTGCGCGACGGAACGGGCCGCAGGAGGATCGGGGTGTATGCACGGTCGTCCCCGTCTCAAACTTCCTCGTGTCCCCTGTTCCCCATCCTTCCACCCATACATTCATCCGTGCGGTGGTGGGGATGAGTGGATGGATGGAGGCGAGGGACGCGGTGGACCATGCCAGGCAAGACCTGCGTCACTCGAAGAAAGGGTGCCTGGATTGGCCTTCGCTTCTTTGCCGGCCACGAGCGACGGCCACCACCGCCCCCCCCGACGGGCTCTCTTTTCCCCCCTCCCGCTTCTCCTCTTTCCTCTCCGGCGCGTACCCCACCGTTTTCCCGACGAGGTTCGAGACCCCCCCCCAAAGCAAAGCGAGAGTACCCCACACCCGCACGCACTCCCCGGTGTCGCTCCTTTTCCCCCCCGGCAGCGCCGGAACCGGGTGCAGGCGGTGGGGGCGAAGATTGGGTTGGGTGAGAGACTAGAACCGGTGTTCTCAACCCTTCTGGAGCCCTACCCTCTGTGCCTGGACTTTCCAGCGCGCGACACGGGGGGGAGCGGGCACGCGGCGGTGGCGATGGTGGCGACGGCGGTGGCTGGGCGCGATCGAGGAGATGTGGAGGGGTGCCAAGCGCCCGCCGCCCTCCCCCGCATCTCTTCTCTCTCGCACACGCACACGACCCGCGGATGGCGAGGATGTGGGGGGAGAGGTCTCCCGGCGTTAGTGGGTGCTATTTGATTCTCCTCTAACCCCCTCTCCCTCGCGGCCCCCTCGACCATCGTCGTGCAGCGGCGACGGCGGCTCCCACCCCGTCTCTCTCCCCCACACGACCTCGACGCGTCCCGCCACCCCGCCCAGTCACGTCCCACCCCCCGGGGAGAGGGGAGAGGGATGCACGGGGGTGGGGGTCCCCGCCCCCTCTCTCTGATCCCAAGACGGTGTTTGTCCCCGTCTCCGGCTCGCACGCACACATCCCCGCCGGGCGGGCCCCGGCGGGATGCCCGGCCAGACAAACCGAGGGGGAGGAGAGGGGGAGGAGGAGGAAGTATAGGCCTCTCGGGGACGGGCGGCGCGTGGCGCCGGAGAGGGGGGTGGCGGGGAAGAGGGAAACCTAGAAGGCGGCCCATCCCCCGAGATGACGAGAGCGGAGAGAGGCGCTAAACGGCGCCTGGGGAGTTTGGGGGAGGAGGAGACGGCGCGCGAGGAGCTGGGGGAGGAGGAGGAGGAGGAGGAGACGGCGCGCGAGGAGGAAAATGGCCGGTCCAACCCTTGGGACGAGAGGTCTCGAATCTTGACACCATTTCCCCTCCCCTCCACCCCACTCCCGTCGCGGCTTCGCGCGGGGAGGAAAATAATCCGATCCGACCCCCGGGGCGAGAGGCTCGCGATCCCACCCCCCCGCTCCCCGGGGGCCGCGAAAAAAGGGGGCGGGGCTTAAAGGGGGGGGGCTAATTTGCATAAATTTGCATGCATTTGCATAGCCCCGCCCCCTCATTTGCATAAATTAGCATGGCCCCTCCCCCCCATTTGCATAAATTAGCATGGCCCCTCCCCCAATTTGCATGGCCCCACCCCCTCATTTACATAACCCCTCCCACTCATTTGCATGCATTTACATAACCCCTCCCCCTAATCTGCATAACCCCTCCCCCTAATCTGCATAACCCCTCCCCCTAATCTGCATAACCCCTCCCCCTAATCTGCATAACCCCTCCCCCTAATCTGCATAACCCCTCCCCCTAATCTGCATAACCCCTCCCCCTAATCTGCATATGACAACTTCCTCCGGAAGTGACGCTCTGGCCCCTCCCCCCTCATTTACATATGACCGCTTCCCCCGGACGTGACGCCGGGCTCCTCCCACCCCCCCCCATTTGCATATGACCGCTTCCCGGACGTGACGTTTTTGCCGATGACGACTTCCGCCTCATTTGCGTATGACCGCTTCCCGCGCGCAACTTCCGCCGGACGTGACGCCGGTCCCCACGGATCTCATCTGCATATCGCGCCGGCCCCTTCCGCTTCCCCCGGACGTGACGCCGGCTTCCGGGGCGCGGACGGGGCGGGCTCCGCGGATCGCATCGGCGCGCCGAGCCTGCCCCTTCCGTCGCACCGGGGGTCCGCGGGCGGGGGCTTCCGCTCCGCGGCACCCGCCCCATTGGCTCCCCTCGCGCCACGCGGTCGCCGTCGTCATCGTCCCGCCGGCCAATCGGGTGGCAGCGGGGGAGGCTGGGAGTGGGGACGGAAGACGGAAGCCAGATGAACCTGTTCCGCCCGCTCTCCCACCGCCTTTCCCGCCCCTCCACGACGACCACCCGGGACCACCACCACCAACAACACCACCACCACCACCCCCCCCCCCCCCCCCCCACGCACACACTTTACTACTATCACCACCAGGGGGCGATGGTTGCAATGGCAGTTCCCTGTACTGACCACCACCGTGTTTTTTTCTCTCTCTCTCTTTCCTTCCCCCCCCCTCGACCACCGCAGGACCACCATCGTCTAACTCCCACCCGGGACCACCGGGACCCTCGGGACCATCTACCTCCCACCAGGACCCGCCGGGACCACCAACACCGTCCACCTCCCACCACCACCACCATCAAGGACCCCCAACATCCCCAAGACCCTCTACTTCTTCCCACCCAGACCCACCAGGAGGAGGACCCCCATCTGCTGAGACCCACCAGGACCCACCGGGACCCTCATCTGCTGAGACCCACCACCACCACCACCAAGACCCTCCAGGAGGAGGACCCCCATCTGCTGAGACCCACCAGGACCCACCAGGACCCCCATCTGCTGAGACCCACCACCACCAAGACCCACCAGGAGGAGGACCCCCATCCACTTCTTCCCATCACCACCACCAAGACCCTCCAGGAGGAGGACCCCCATCACCCCCACCAAGACCCTCCACCTCTTCTTCTTCCTCCCACCAGGGACCCCCATCCACAAGACCACCTCCGCCCCAGAGACCACCGCCAAGATGGCCGCCTCCATCTCCCCAAAAAATCTCAGAGACTCGAGCTGGTTCAGAAAATACAGCACAAACTTTATTTTCTCACTCTGAAAATAAACTCTTTTCTCACCCGATGGGAGAAGGAGGAGAAGGGGACCGGGGGACCGCGGGAGGAGGAGGAGGAGGAGAAGGGGACCGGGACGATCCTCCGCCGCCGAGCCCTCCGCCGCGGCCGCCGCCGCCGCTTCCACCACCGCCGCCACCACCGCCGCCACCTCCGCCGCCGCCGCAGCCGCCTCCGGCCGGGGGATCCGCGCGGAGGAGAAGGAGAGGAGGAGGAGGAGGAGGGCCGCCGGGCCGGGGAGGCAGGCGCCGGGGAGGCAAGCGCCGCCGGGCCGAGGGGACCGAGGCCGCCGCCGCGGACGCGGAGGAGGAGGAGGAGGAGGAGGAGGAGGACGGGGACGAGGACGAGGACCGGGCCGGGGGCGAGGGGAGAGAAGACGGAGGAGAAGGGCCTCGAGGAGCCGGTGGAGGGGCCGGAGAGTCAGAGTCAGAGTCCAGCCGGGCCGAGGGGGCGCCCCGCTCAGCGGAGCAGCAGGTAGGGGTTGCCGGCGTCCTCGGCCTCCTCGTCGTCCGAGATGGCCTCCACCTTGATGGGCCCGAGCGGGCCGCGGGGCCGGCCGTCGCCGCCGCGGAAGCCGACGATCTCCACCGCGGCAGAGTCCTCCCCGTCCTCGCCGGGCCCCCGGGCGCCCGAGGGCCGGTGGGTCTCCACGGCGCCGCCGGCGGCGCGGACGCTGGTCTCGAAGGGCGCAAAGTCCCAGCGCACGGCCGGCGGGGCGCCCGCGGCCGCGAGGGCGCCCGGGGCCAGCACCAGCGGGGCGGCCTCGGCGTCGGGCTCCAGCAGCGCCGCGGCGCAGAAGGCGCGCAGCTCGGCCGGCAGGCCCTCGGGGCCGCGGAGCTCGGCGAGGCCCCGGCGACCGCAGGAGACGAAGACGGGCCGCAGCGGGGCGCCGAGCCCCCAGCGGTTGGCCGCGCGGTGCCCGAAGGCGGCGCCCGCGTCAAAGTCCGGGTCCCCGAGCCCGAGCGCGGAGCGCTGGCGGGCCATGTCCTTGCAGCCGTCCACGGTGGGGAGCACGCGCTGGCGGTAGGCGCGCGGCGGCAGCGGGACCGGGGTCCGGGGCCCGGCGCGGGTGCTCACCGTGTAGCGCACGTTGTCCTGGCGGCAGAGGCGCAGCGGCTCGGCCCCGGGGTGCAGGCGGGCGAAGGAGGCCTCCACGCGGGCGAAGCAGGCCGGGCCCACGATGGAGCTCGAGTCCAGGACGGCCGCGCGCAGCTCGCGGCACTCGGGCCAGCGCACGGCGCACTGGGCGGCCGGGTCCAGGCGGGCGCGGACGTAGACGTGGTAGTCCCCCACGGCCGGGCCGTCCGCGGGCCAGTCCTCGATGGTGTCCAGCACGATGAGCCGGCGCCGCGCCGCGCCGAGCCGCGAGCAGAGGTACTCGACGGCGCCGGCGAAGCCGAGGTCCCGCGCCGAGAGCAGCAGCACCCCCTGGGCGTTGAGGCGGCCGATGTCGGGGCGCCCGGTCCAGTTCCCGGCCCAGGCGTGCGAGTCCGGCGTGCAGAGGCGGTGGGCGAAGGCGGCGAGCAGCGCCGAGAGGCCGCCGCGGCGCGGGTCCCAGGCCGGGCGCGGGGCGCCCTCGGCGGGCTCGGCGCAGAGCTCCTCGTGGGGCAGCGGGTCGTAGAGCACCACCACGCGCACGTCCTCGGGGTCGGCTATCTGCCGCATCCAGGCGGCGCGGCGGCGGAGCGGGGCGCCCGCGGCCCCGCGGCGCGCGGCGATGTGCGCCAGGGCGGCCGGGTCGAAGGTGAGCGCCGGGCGCCAGAGTTCGGGGAAGACCTCCTGGTCCACGAGGACGCGGGCCACCTCGGGCGGGCAGTAGGCGGCGAGGGCCGCCTCGGAGGGCCGCGGCGTGTGGGTCTCGCCGGCCGGGACGCGGCGGAAGCCGCCGTCGGGCGCGGGGTGCTCGGGCATGGGCCCGAGCGGGCGCCGGAGCCGGTCGTCCTCGGAGGAGGAGGAGGAGGAGGACAGCAGCGCGGGAGCGGGGTCCGGAGCGGGCCCGAGTCCGAGGGAGCGGCGCTTGCGCCGGGGCCCCCGGTCCTCTTCGTCGTCGCGGTGGCCGTGGCCGTCCCCGCGGAGGGCCGAGCCGGAGAGCCCCTCGTCCTCCTCGCCGTCCCCGGGGCGGCGGGCCCCGGGCGCGCGGCGCTTCTTCTTGCGCCGCTCGGGCGCTGGGTCCGGGCCGGCGGCGGGGGAGCTGGCGTAGCCGGAGGAGCCGGAGAGGCCGGACTTGGTGCCGGGGCTGCCGGACTTGGTGCCGGAGCTGGACTTGGTGCTGGAGCTGGACTTGGTGCTGGCGGGGCTGGAGGGCCCGGAGCCGGGGAGGCCGGAGGGGGCGCCCGCCGCCGCCGGCGCCGGCGCTGGGACGACGAGGCCGGGCTGCTCGGGCCAGAGCGGGGGCAGGCCGGGCGCGGGCTCCGCGGGCCCGGGCCGCGCGGCGGCCTCGGCGAGCCGGGCCGCGGCCACGTTGGCCGGGGCGAAGAGGGCCGCGGCGTAGGTCCAGGCGGCCTCGCGGGCGCGGGCCCCGTCCACGCTGTAGCGCACCAGCGGCGCCACGGTGCGGGCGACGAGGGCGACAGAGTCCGCGGCCTGCTGCCGCTCGGCCGGGCCGGCCCCGGGGATCGCGTCGCGGAGCGCGAGCAGCGCGGCGGTCACCTCCTCGAGGCAGGCGGGCCCGAGGGCGGCCGGGGCGCGGGCGGGCGCGGGCAGCCGGAGCGGGACGGGCAGCAGGCGCTCGAGGACGCCGCGGCAGGCCAGGACGCAGGCGTCCGCCAGCTCGCGGGGCACGCGGCCGGGCTGCGCGGCGGCGAAGGCGGCGCGGACGCGGGCGCAGAGGGCCTCGACGGTCGCCTCCCCGGCGCGGGGGTCCGCGGCGCGGCCCGGGTAGGCCATGTCGGCGTAGGCCCGGCGGAGGCTCTGCAGGATGAAGGTCTTCTGGGTGCGATCGTAGCGGCGGCTCATGGCCACGGCGCTCACCGCGTGCGGCAGGGCCCAGAGCGGGTCCTGGGCGGCCATGGCGTCCCCGATGTGCGGCAGCGGCGGGGTCACGCTGCCGGTGATGAAGGAGCCGTGGCCGTGGGGCGCGTGGACCCGGCGCTGGCAGAACTGGTTGAAGCGCTGGTCGGGGGCCTGCATCCGCGGGTTCTGCAGCCAGGACATGGCCTCGCCGGCGGCCCCGCTGTAGATGAGGCGCACGAGGGCCTCGTGCTGCTTCCTCGAGTCCCCCATCTCCGGGATGAAGACGGGCACGGGCCCGGCCGCGGCGCGGTAGCGGGCCGCGGCCTGGCGGACGTCGTCCTCGTCCCAGAGCCCCTCGCGGGAGTCCCCGGCGCCGCCGTAGCGGACGCGGCCGTCGGCCGGAGGGTCGGAGCCGGGCCAGGGCTCCCCGAGCGGGGTGAGCAGCGGCCCGTCGGTCGGCGGGGGCCCGTCGGCCATGAGCGAGAGGTGGTTGTTGGTGGAGCGGCGCTTCCTGCGCGGGGGCCGGGCGGGCTCCGGGGCCGGGGCCGGGGAGGCCGCGGCGGAGGAGGAGGAGGAGGAGGCGGCGGCGGAGGAGGCCGAGGGCCGCGGGGCCGCGGCGGGCGCCGGCGGAGACGGTGGCGGCCCGGCGCGGGCGAGTGGGGCGCCGGCCCGGACTCCTTCGTCTTCTTCTCCCTCGGAGGAGGACGAGGACGAGGAGGACGAGGAGGACGAGGACGAGGAGGAGGCCGAGCGCCGCGCGGCGGCGGCGGCGGAGGCCCGGGGGGCGGAGGGCGAGCGGGCCGGGGAGAGGTCCGAGTCGCTGCCGCTGCTGCTGGAGCTGCTGAAGCCGCGGCCGCGGCGGAGGGCGCCCTCTCCGGCGCGGCGCCGGCGGGGCTGTCTCTGCGGGGGCGCCCCGCCGTCCCCGGCGAGGCCGAGTCCGTCCTCGTCCTTCTCGGGGCCGCGGGCGACGGGCTCGACGGCGACGGTGGTGGAGGAGCTGGAGTTGGAGTTGGAGTTGGAGGAGACGGGGCTCCGGGCGCCAAGCGGCCGAGGATCGAGCCGCCTCGCGGCGGCGGGCTCGTCGAGCAGGGGCTCGCGGTGCTGGTGATGGTGACGACCGCGGTCCCCTCCGGCGGAGGGGGCGCCGCCGCCGCCGAGCGCCGAGACCGGCCCGGCGGCGGGGGAGGCTGGGGAAGCGGGCCCCCGCCGTGCCGGCGCTGCGGCCACCGCTGCTGGCTGTGCTGGTGGCGCCGGGGTCCGAGGCCGCGCCGCCGGCCCGGGCTCACCGACCGGGTCCCCCCTCGCGGGGGACCATCTCCGCGGGGCTGCCGAGGGGCCGGGGGAGCCGGAGGAGGAGGCCGGGGAGGCTGCGGAGGGGGACGGGCGCCCGGGGCCGCCGGGGGCCCCGGCCTCTGCCGCTGCGAGTGCTGCCGGGGTCGGCGGCCGGGGCCCGGAGCCGGCCCGGGACCGGGGCCCGAGGACGAGGTGACCGTGCTCGGAGCCCTGATGGAGAGCCCGACCGGGGGACCCGGCGGCCGGGGACCCGGGCTCGTCCTCCTCCTCGTCTTCGTCGTCTAGCACCACGATCTCGCCCGAGCCCCGGCGGGGCTGCTGCTGCTGCTGCTGCTGGGCCGAAGGAGGACGGGGCGGCCTCGTGGCTCCGGCCGCGGCCGCGAGGACGGCGGCCTCGGCCTCGGCCTCGCCGGCATCGTCGGAGAAGAGGCCGCCGGGGCCAAAGAGGAGATCCTCGCCGGAGGGGCCGCGGCGCCGGGAGCCCTGGCTGCCGCCGTCGGAGCCGGCCGCGGTCCCCTCTTCCTCGGCCGCCGCGGCCGCGGCCGCCGCCGCCAGGAGCTGGCTGAAGTTGCCCTCGGTCTCGATGAAGTCAAAGAGATCGTCGGCCATGGTCTCGATCGGTTCTCGGGGTGTCTCTCTCTCTCGCGCGCCGGGAGCCGAGCTCGGAGAGCGGGTGCTGGAGGGTGAGAGGGAGCCGGAGGAGCGGGCGCGGCAGAAGAAGAAGAAGGAGAGGGCGAAGAAGGAGAAGAAGAAGAGGGCTCTCCTCTCTCTTCCCCCCCGGTGGGTCGGGCCGTCCGTCGAGGGCTGATAGCCGCCGGAGAGCCGGAGTCTTCAGAGTCCGCGCCGGAGCCGAGACGATCGGATCCCCTCGGGTTGGCAGAGAACGATGCTGTCCGTACCTGCACCGCAGTGAAGTGCTACGATGGAGACCGTGCCTATAAACTCCCCGAGAAGAGCCCGCCCCCAGGTAAGCGGACCAATGGCCGATTTTCGCCGCGGACTTCCCCGACGGCCGGCCAATGGGATTTTTCTCGCCCGCTTCCTCTCGCGTCTGCTTTGCATGCCCGGCCCAAGATGGCGGCCGCCGGCCAATGGGATTTCGCGAGGAACTTCCTCGCGAGGACCATTTGCATGCCCGGCCCCCGCGGCGGCCATCTTGCCCACTCGACGGCCAATGGGATTTCTCTCGCCCGCTTCCTCTCGCGTCTACTTTGCATGTCCGTCCCCCGCGGCGGCCATCTTGGTCGACGGCGGCCAATGGGATTTCTCTCGCCCGCTTCCTCTCGCGTCTACTTTGCATGCCCGGCCCCGAGGGCGCCATCTTGGCCCCTCGACGGCCAATGGGATTTCTCTCCCTACTTCCTCTCGCGTCTACTTTGCATGCCCGGCCCCCGCGGCGGCCATCTCGGCTCGCCCGGGCCAATGGGCGCGCGGAGGCGTCTCCCGCGCGCCTCTGATTTGCATGCCCGGCCCGCTCTGCGGCCATCTTGGCCGCGGGCGGCCAATGAGATTGTCCGAAAATCCCTCGCGCGGGCGCGAGGCGCATGCTCGGCACGCGACCCACCCCCGTGGTGCTAGCGAGCCAATCAGATGATTTTCGGGGAAGCTTCCGTGTGCACGTCATTTGCATGCTCGCCCCACGTGGCCGCCCTCGGCCAATGGGGCCTCACGGTGCAAGCTTCCGTGTGTCTGCACGTGGTCCGCATGTGTTGTGGTGGTCTCTCAGTGTTTTGTGGTGGTCTCTGTGTTGTGTGGTGGTCTCTCAGTGTTTTGTGGTGGTCTCTGTGTTGTGTGGTGGTCTCACCGCCTCCCCCTGCCACTCGCGAGACCCCCGAGACCCCCGTTTCCCCCTCCCCGAGACCCTTGAGGCCCCCCGCGACCCCCGCGGTCGCCCCACCCGCGCCTGCGCGCTCGGCGCGCGCTCCGAGGGCGCCCCCAGCCGGTCGGAGAGACGAGCGGAACCGCCGTCGGACCGGGGACCGGCGACCGGACCCGAACCGGGAAGCGACGCCGGGGCGGGAGAACCGGACCCGAACCTCGAGCCCGGACCCGCCCGGACCCGGAAGGGAGGAGCCGGACAGCCACGCCTTGGATACTTTTGTCGCCCACCCACCCCCCCTCTACCTCTCTCTCCCGGTCCCCCCTCCCACCCCACGAGACACGCCCCAGAGTGAAAAAAAATAAAAGTTGTTCTCGTTGCACCGTCTTCCGGCTCGTGTCGTCCTTCCGCGGTACCTCGGGCGGGCGGGAGGGGGCGCGAGACCGGCTCGGCCTCGTCGGGGGAGAGGGAGTTGGGGGGGAGGGGAGCCAAGATGGCGGCGGGGCGTGGCGGGGCGTGGCAGAGGGGAGGGGGGGGTCGGAGCGCGGACCCCGCCCGGTGGGGGGGCGGGTGCAAAGGGGCGGGGCCCAAAAATGGACCTCGGGCCGGGACCCCGGGGGCGCTCCGGGAGACGAAGAGGGCCGGGCCCCCTTCCCGGCGGGGAGGGCCGGGCCGGCGCGCCGGGACGCCCCTCCGGGGGAAAGAGTGTCCCCGCGCGGGCGCCGCGTCCCGCCCCGAGCCCCCGGGGGCGCGCGGGCCTCGGTCGCGCCCGCCGGACGCGGAGGCGCGAGGCCCCCGCCCCCCCGGGGGGTCCGGGACGGGGGGGCCCAAAATCGGGCTGGGGGACGGCGGGGGGGTTTGCTCCGGAGGTCTGCGGGTGTGAGTGTGTGTGTGTGTGCGGGGAGAGAAGGCTCGCTCTCTCTCTCGGAGGGCCGGGCGGGTGGAGGGTTGGGCGGACGGATGGTCGGTCGGACGGGGCGGGCGGGGAGGGTGTCGTCCGTGTGTCTGTGCGCGGGGAGAGTGTGATGTGGGGGTGTGCGAGAGAGAGAGCGTGCGTGTGGGTGGGTGTGAGGGCCGGTGCGGGTATGGGGGCCCGTGCGGGTGGGAGGGTCCGTGTGGGTGTCGGTGGTGCGGGTGTTGCTGGTGATACCGGTACCGACGGTGGTGGCCGGGCGGGCGGTCTCTCCGTCTCCGTCTCCGCCGTCGCCTCGGTCCGAGGAGGGGGGGCGCCCGGGCGTGTCTCGCCTTTCCCCCAGTTCGCCTCCTCCTTCTCCTCCTTCTCCTCCTTCTCCTCCTCTCCCCGCCCCCGCCCCCTGTCCCGGTCCCGGTCCCACGCGCGCGCCCCGGGGAAGGGTCGGGCGATGGCCGCCGCCACCGCCCCACCCTTCCCCCGGACCCCCTCCCGCCCGCCGGGGCGCCCCGCGTGCTCCGGGGGCGCCGGCCGGCCATCCCCCCACACCTCCCCTCCTCCCCGTCCCCAACCCGCCCCGCACTCGCCCGACACTCGGCCCGCGGCACGCGCGCCCAGACCTTCCTCTCCCCCCTCCACCGCCCGCCCTCGCCCCCTCCGTCTCATGCCACCCGCGGCAGCATCCCCCCTCCCCGTCGTCCTCAGCCAACAGCCAACAGCCAACAGCCAACAACCCACAGCCAACAGCCTCACTCTCGCGCTCTCCCCGTCGCGGGAAAAAAAACACGGCACCACAGGCGGGAGCGGGGTCGCTCGGGGGAAGGTTTCATTCAAACGACAGGCGACAGGAGGGGGGCCCCCGCGCGGGTCGGGCCTTCTTCCCCATCCCCGCCGGCCCCCCTCTTTCTCTCTCTCTCTTCTTCCGCGTCTTCTTCCGCGTCCGCTTCGGCCCCCCGCGGCCGCGGCGTGGGAGAGCGGGGCGGGTGGGGAAGGAAGAGGGGACGGGGGGGAGAAGAAGAAACGGGGAAGCGGGGGGGGAAGGAACAGACGGGGAAGGGGGGGGGGAAAGGGAAACGGGGAAACGGGGAAGGGGCCGGCGGAGGGGAAGCGGGAGGGAGAGGCGCGGCGCCCCGCGCTTCCCCTTCCCGCTTCCCCCTCCGCCCGGCCGCGGGCGCCCGGGAGACGGGAGCGAAAGGAGGAGAGGGGAGGAAAAAAAGTCTAGAGCGGGGAGGAGAAGTCACCGTAGTAGTCATAGTAGTAGTCATAGTAATATAGTAGGAGTAGGAGTAGTAGGAGTAGTAGTAGTAGGAGAGTCTGTGCTTACCGAGAGAGGAGAAGAAGAAGCAGAAGCCGGGGCGCTTCCTCCCCGGCGGCCCGGGAAGAAAGCCCCGGCGCCTCGGGCCCCGGAGATGGGGACGCGGAGAAGGAAACACCCAAGGGCGGAGGAGGAGGAAGAAGGAGAGGAAGGGAAAAAAAAACCTCGAGAGCGGCGGCGGCGGTCGCCGCGGCCAGGAGGAAAAACGGGGGGAGCGGGAGCGGCTCCCGAGTCCGGGAAGGAAAGGCGGGCGGAGGAAGGCGGGTGTGGAGGAGGAAGCGCTCGCGCCCCTCGCTCTCTCTCCCCCCGTCCCCCTCTCCGCGTCTCTTCCCCGCGTCCTCTCTCTGCGTTCGCCGGTCGCGGCGCGGGCGGCGGCTGCAGAGGCGGCTGCGGACGCGGAGGGGGGCGAGGGGACACAGAACTCTTTTTGTCCTCCTCGCCGGCTCCCAGGCGGCGGGCGCGTCCCGCGTCCCCGGCGTCATCACGCGGGACCCCCCGGTCCCCGTGACGCAGGACGCGGGGAGGGGAGGGGAGGGAGGGGAGGGGGTCGGGGGGAAGGGAGGGGGGGGAGGAGGGGGAGAGCGGGTGACGCCGCGGGTGGGCCGAGGCCGGCGGGGAGGGAGGAGGAGGACGCGGCGGCGGCGGTGAAGGAGGAGAGCCGCCCGGCGCGCGCCCGCCGCCGCAGGCGCGTCCCCGGCGCGGGCGCCTCTCCACGCCCCGCTCCGCCCGGCGCCCAATGGCGCGGCCGGCTCGGCGGCCCGGCCCTTCCTCCGCCCTCCTCCTTCCCCGCCGCCACCGCCGCCTCCTCCCTTCTCCCTCCTCCCTCCCCTCCCGCCGCCCGCGCGCCCGCGTGCGCTCGTGCCGGCGCGGCATCCCCGCCCAGGCGGCGGGGGAGCGCGGAGCGCGCCGCCTCCCCGTGATCACGTTCGCGCACCGCGGCGCCAGCAGGAAAAAAAAACAACAACACAGAGACACACCTCCCTCTCCGTCTCCCTCGCCTTCTCCGTTTTGGGGGGAGACCGATGCCGGTGCCACATACACATACACACGCGCGCGCACCCCGTCCCCCCCCCTTCCCCTTCCCCTCGCCCCGGCCCCCCGGCTCGGTGCCGGAGAAAAGGGGGTGCCGGCGCGGACCGGGGTCGGGCCCGCGGCGCCGGGGGGTGGGCGCGCGTGTCCGTGTGAGAGTTTACGGTGTGGGTGGGTGTCACGGTGGAGCGAGCTGGGGGGCCCCGGCTCGCTCGCTCGCTCGCTCGCCAGGCCGGCAGGCCGGGGGCCCGGCCTGGACGCGGGCCACACTGTGCGGCGGACCGTGCCAAGTTTAGGGCGCTGCGACCCAGAGAGAGCGCCGAGCTAAAAGCTATTTTATCGCCGCTGCGGTTTCCCCTGGCGCGGCGCCTAGACACCGTTTCTGCAGACAGAGCCCTCTCTAATGGCGCGTCCGGGAAGGCTTCTCCCGGGCCGCGAGGGCCGCGGTGGCGACCCCCTTCCTCCCCCCTCCTCCCCGTCCCCGTCCCCGTCCCCCGCCCGCTCTCCCCGGGCCGCCGCGATACCGCGCGGGCGATACCGCGCGGGCCACCCGCGGGCGCTACCGCGCCGCTCCGCTCGCCGCCTCTTCCCCCTCCTCCACCGCCGCCAGTCCTCCTCGCTCCTCTCCGCCCACGCAACTCCTCCCGCCCGCGTGCCCCCGGGGCCATCGGCTGGAACACCCTGAGCCTTCGCTGAGTGCTGCCCCCGCCCCGCCCGCGGCACTCCGGGCCCGCGTGAGAGGGTACAGAGCGAACCCGGCCCCGACCCCCTCCGCCCGCCGGGGGAGGCTTCCCGGAACCAGCGCGGTCATCAACCCCCTCCCCCACCCACCCGCACAGCGCGCACGCCGACCGCCCGCGTTTGCTACGATCCCTGCGCCCGACGGGGCCGCGGGACGGCGCGCGCGGCACGGAACGAGAGGGGGGCGGGGGTGCGGGCGAGTGAGCGTGCGCGAAGGGGGGAGAGGGCGCGTGGGCGAGAGGGACGGGGCGGGGCGGGGTGGGGGGAGCGGCGGCTCGCCCTCCGTCCTCCCCTCCTTCCTCTCCCCGTCGACTCGCGTCGCGGCGATTCGGGCCGCATCGAGGCGCGTCGGGGTTTTCGCGCCCTCGCGCCGCCGCCGCCTCCTCATCTTTCGTGGTTAACACACACCACACATACACACACCCTCCCCCGCGCCGCTGTCTGTGGCGCCCGTCGTCTCTCCCGCCGGCGCAACCCCGGGCCCTGAGAGCTTTCTCCGCTTTCTATTTCGGCCCCGGCCTCCCCGACCTCTCTCACCCGCCCTCTCGCGCTCTCTTTCCCACACACACACACATAACAAGAGGCTCGCCCTCGTTCCATCATCTCGGCGTACTCGCCGCCTCGTACACTACATCCGTCTACCGCCGCCAGCAACCCTTTTTTTCCCCCGCTCACTCCTCTTCCCCCCTCCTCCTTCCCCCCTCCCGCCTCTCCATTCATCAATCTCGTCCAGGCCGCGGGCCCATTCACCATCGCCGCGGTCCGAGCTCGGCCAGGAGAGATGGATGGATGGATTGGGGGAAGGAGGGGGCGGGGAGCGCGAGCGGGAGAAGGTTGGCGAAGGAGAGAGCGAGAGCGGGAACTGGAGAGGGGGCTGGGTGGGGTGAGGCGAGTGACCGCCACCGCCCTCTACCACCACCACCCCTCCCCTCCCTCTCCTCTCCTCTCTCTCCCCGGTCCCGTGTGCACACACAGAGAGTGTTTTTTTTTCCTTCTTCTTCTTCCCGGTTCCTCTTCGTCCTCCGGACCCTCGGTGCCGCGATCCCCCACCCCGCCCCCCTTCCTCCTCCCCCCATTTCCCCCGCCCCCAATTCCCCCACTCCTCCCACCAGCCCCCGCCGCCGCGGCCGCAGCCGCCGCGATCCGTCCTCCCCATTCATCCTTTTTTCCAGAGGCCATTCATCGCCCCGCTCATCCGTCTTCATCGCCGCGGAGGGCTGGCTCGAGGCCAGCGCGCCCTTTCCTCTCCCGGCGCGCCCGCTCCCTCCGCGTCCCCTCTCCCCGCCGCCCCCCTCGGCCGCACCGAGCCTTCTCCGCGCGGTCCCGTCTCTCCCCTCCCCCCATCCCGCCCCCACTCCCCGCCCCCCTCTCCCCCTCCCTCTCCCGCCGGTGTTTTCCCACGCCCACACTCACTTTCTCCGCTCCCACCCCACCCTCCTGACTCACCCGTCCCGGGGGATTCCCTGTCCCCCGGGGCGCGGCCGACCCGGCGGTGACCCGTCCCCGCCCCGCCGCGCCCCTTTTTCCTCGAGAGCGGGCGTCCGGGAAGGGAGAGGTCGCGGCGGGTCACGCGCGCTCCCGCGACTTCCCGGCCCGCGATGCGGTCCCGGAAATTTCCGGGACGGTCGCGGCGACCGTCTCGGGGCCCGCGGCGCCGCCAACTCGAGTCTTGGCACGCGTCCAGCGTTCGCACTTGTTGTATATAAGATATAATACTAGTATATATTAGCAATTGGTGCGAACGTGACGTGGCCCAATCAAATCTCTCCACGGGCCCCACGTGCCCCTATTGGCAATTTATCGACCGCTTTCCGAAGGGATCGAACCCCGGGGGTGGGTGCCCCGGGGACGCGCCGGCCAATGGGGGAGCCGGACCCGCGTCCCCGGAGCCCGCATTGGCCGGTCCCGGACGCCCGTCCCGCGGGCCGGACCGCCCCCTCCTCGAGTCTTGGCACGCGTCCAGCGTTCGCACTTGCGGTATATAAGATATAATACTAGTATATATTAGCAATTGGTGCGAACGTGACGTGGCCCAATCAAATCCGGCCACGGGCACCACGTGGCCGTATTGGCGATTTATCGACCGCTTTCCGAAGGGATCGAACGCCCGAGCCCGCGTCGCCGCCGTCTCGAGTCTTGGCACGCGTCCAGCGTTCGCACTTGTTGTATATAAGATATAATACTAGTATATATTAGCAATTGGTGCGAACGTGACGTGGCCCAATCAAAACCTGCCGTTTGCCCCACGTGGCCGTATTGGCAATTTATCGACCGCTTTCCGAAGGGATCGAACCCCGGGGGCGGGTGCCCCGGGGACGCGCCGGCCAATGGGGGAGCCGGACCCGCGTCCCCGGAGCCCGCATTGGCCGGCCCGGGACGCCCGTCTCGCGGGCCGGACCGCCCCCTCCTCGAGTCTTGGCACGCGTCCAGCGTTCGCACTTGCGGTATATAAGATATAATACTAGTATATATTAGCAATTGGTGCGAACGTGACGTGGCCCAATCAAATCTCTCCACGGGCCCCACGTGCCCCTATTGGCAATTTATCGACCGCTTTCCGAAGGGATCGAACCCCGCGGCACCCGCCTCCGGGGACGCGCCGGCCAATGGGGGAGCCGGACCCGCGTCCCCTGAGCCCACGTGTGAGGCCCCGGCCAATGGGCCCGTCGGCCCGGCCCCCTCGACGGGGCGATAAAGGGGTGCGCGGCGGCCCGGCCGACGGCACTCGCCGCCCCAACGACGCCGACGGGGATCGACCGGGAGAGGCGCGGAGCGCGCCCCGAGCCGACGAGGAGCCGCGCCCCGCTCGAGGACGCCCGGACGACGACGGTGACGTCGGCATCGGCCTCGACGACGACGATCGCGGGACCCGACGGTACCGGATGCGGGGGATCCCCCGGTCGGGGTGGGCCGGGAGTGGGCGTGGCACCTCCTCCCCCGTCCCCCCTCCCTACCCACCCCGCGCGATCGCGCTCATCGTATCCGTTCTCGTTTCAGGTGCCGCATCGTTCCGGAAGAACCGCCTCGGGGGTCGCGCGTCCGTCGCCTAGGTGAGTCGGGGGGCTTCCCGGGTGCGGCTCCCCCCTCCCCCTCCCCCTCCCCGGGACGGGTCGGGTGGGTAGGTGGGCGGGGGTCGGGAGGGATCGGGGGAAGGGGAGGGGGTCGTGGCTCACGCTGACGTCATGCGTTTTCTCCCCCCCCCCCACAGGGACGCGAGCGGGACCGGCTGGTCGACCACCCATCCAATCCATCCAATCCATCCAGCCACCCACCCGTCGGGGCGGCCGGGCTCGGGTCCGGTTCCGGGTCGGCGTCGGGGAGCCTCGCCCACCCACCCGGCCCCCGTCCCAGCCCAGCCCCCCGCACACCCCCATCGCACCCCCTCCCACCCCACCCCGTTCCCACCACCCCGCCCGACCCACCGCGTCCGCCGCGCCCATGGACCGGGTCTGGGCCGACTGGTACGAGCCCGTGCCCTCCCCGCCGTTCTCGCCCGTCGACCCGCCCGGGCCCCGGCCCGCGACCCCGGTCCCGGGGAGCAGCCCCCCGTCCCCCGCCTCGACCCCCACGCCCCCCAAGCGCGGGCGCTACGTCGTCGAGCACCCCGAGTACGGGCCCCCGCCCGACCCCGAGGAGGTGCGCGTCCACGGCGCGCGGGGCCCCGGCGCCTTCTGCGCGGCCCCCTGGCACCCCGACACGCGGCGCCTCGGGGCCGACGTGAACCGCCTCTTTCGCGGCATCGCCGTCTCGGCCGCCGACGTGACGGGCGACACGCGCGCCCTGCGCCGCGCCCTCTTTGACTTTTACGCCATGGGCTACACGCGCCAGCGCCCCTCGGCCCCCTGCTGGCAGGCCCTGCTCCAGCTCTCGCCCGAGCAGAGCGCCCCGCTGCGCAGCGCGCTGCGCGAGCTCAACGAGCGCGACGTCTACGACCCGCGCGTCCTCTCCCCGCCGGTCATCGAGGGCCCGCTCTTTGGGGAGGAGTGCGACGTCGACGAGGACGACGCCGGCTCGGACACCACCGTCGCGTCCGAGTTTAGCTTCCGCGGCTCGGTGTGCGAGGACGACGGGGAGGACGAAGAGGAAGACGGGGAAGAGGAAGACGAGGACGAGGAGGGGGAAGAGGAAGAAGACGAGGAGGAAGAGGAAGGGGACGAGGACGGGGAGACGGACGTGTACGAGGAGGACGAGGACGAGGACGACGAGGACGGGGACGGGCTCTGTGAGGACGAGGACGAGGAGGACGGGGACGACTTTGACGGGGCCAGCGTGGGCGACGACGACGTGTTTGAGCCCCCCGAGGACGGCTCGGACGGAGAGGGCTCGGGCTCGGACGACGGCGGGGACGGGGAAGAGGAAGAGGAAGAGGAAGACGGGGACGAGGACGAGGAAGAGGAGGACGAGGAAGAGGAGGACGAGGAAGAGGAGGACGAGGAAGAGGAGGACGAGGAAGAGGAGGACGAGGAAGAGGAGGACGAGGAAGAGGAGGACGAGGAAGAGGAGGACGGGGGGGAAGACGGTGAAGACGGCGAAGAAGACGAAGACGGAGAGGGCGAGGAGGGCGGGAAGGACGCCGCCCGCCGGGGGACGCGCGCCCCGACGCGGCCCGCCGCCGCCCCGTGAGGCGGGCCGCGCCCCGCTCTGTTCTTCCTCCACCCCCACCGTCCGCCCCGTCGTTTGCCCCTTTCCCCTTCCCCCTCCCCTCTGTTGTGCCCTCAATAAACACGGCGGCCCGCCGCTCGAACCTCAACTCTCTCGTCTCTCGGGCGTTTTTTCCCCTCCCGGCCCTCGTGGGGAAGGGAGATGGGGGGGATGAGACGGGGTGGAGGGAGGGAGATGGGAGTGAGACGGGGGGATGAGACGGGGGGATGAGACGGGGAGCGAGCCGCGGAACCCTCCCGCGGGGGGCGAGAGTCGGACGAAGCGGGGGCAGGGGTCTGGGTGGGAAGAAGTAGAGGGTCTTGGGGATGTTGGGGGTCCTTGATGGTGGTGGTGGTGGGAGGTGGACGGTGTTGGTGGTCCCGGCGGGTCCTGGTGGGAGGTAGATGGTCCCGAGGGTCCCGGTGGTCCCGGGTGGGAGTTAGACGATGGTGGTCCTGCGGGGGGTGGGGGGGACGGGGTGGGGGGAGAACCGCCGGTCCGGCCGACGCCGGGCGCCGGACGCCGGACGACGCGGTCGCGCACCCAGCGCACCACACGCGCCGCGCACCATCGTCGGCGGCGCGCGGGGGGGGGATGGGGGGATGGGGGATGGGGGGAAGTGGCGTCAGTGGTGACGTCGGTGAAGCGGGAGGGGGAAAGGGGTGAGAGTGTGTGGGTGGTGGCGGCGCGAGTCTGCGGGATGGTGCCGAGGGGAGCCGGACGGTCACGGGGAGGGAGGATGTGGCGCGCGTGGGGCGTGGGCCGCCTCTGCCGCGGTCGCGAGCCGCCAACGCCGCCGCCACCGCCTCCCGCCCAAAGTGATATCGCGGCGTGCCGGGACGGCCGGCGCTCATTCACACCGCACCCGTTCACCCGCGTCCCCGCGGGCAAGCACGCACACACCCGGTCGCGCATCATGCTGGCGATGTGGAGATGGGTCACCAAGAGGTCGCGGCTCCGCCGAGGCCACGCCCATCTTGGGGGAAATAAAGGAGTCCGGGGAATGTGTTCCTTATACCTTGCCGGGCTCAGCAGGGGGTTGTCGCGCGTCCACGCCCAGCGCTCGCACGCAGCAACAATGGCCGACGCCGGAATCCCCGACGAGATCCTGTACTCGGACATCAGCGACGACGAGATCATCATCGACGGCGACGGCGACAGCAGCGGGGACGAGGACGACGATGACGGGGGGCTGACGCGGCAGGCCGCGTCGCGCATCGCCACGGACCTGGGCTTCGAGGTGCTGCAGCCCCTGCAGTCGGGCTCGGAGGGCCGCGTCTTCGTGGCCCGCCGGCCCGGCGAGGCGGACACGGTGGTGCTGAAGGTGGGCCAGAAGCCCTCGACGCTGATGGAGGGCATGCTGCTGAAGCGCCTGGCCCACGATAACGTCATGAGCCTGAAGCAGATGCTCGCCCGGGGCCCGGTGACGTGCCTGGTCCTGCCGCACTTTCGGTGCGATCTGTACAGCTACCTGACCATGCGGGACGGGCCGCTGGACATGCGCGACGCCGGGCGCGTGATCCGGTCCGTGCTCCGCGGGCTCGCCTACCTGCACGGGATGCGCATCATGCACCGCGACGTCAAGGCGGAGAACATCTTCCTCGAGGACGTGGACACGGTGTGCCTGGGGGACCTCGGGGCCGCGCGCTGCAACGTGGCGGCGCCCAACTTTTACGGGCTCGCCGGGACCATCGAGACCAACGCCCCCGAGGTGCTCGCGCGCGACCGCTACGACACCAAGGTCGACGTCTGGGGTGCGGGGGTGGTGCTCTTCGAGACGCTGGCCTACCCCAAGACGATCACCGGCGGGGACGAGCCCGCGATCAACGGGGAGATGCACCTGATCGACCTCATCCGCGCCCTCGGGGTGCACCCCGAGGAGTTCCCGCCCGACACGCGCCTCCGGAGCGAGTTCGTCCGGTACGCCGGGACCCATCGCCAGCCGTACACGCAGTACGCGCGCGTGGCTCGCCTCGGGCTGCCCGAGACGGGGGCTTTCCTGATTTACAAGATGTTGACGTTTGATCCCGTCCGCCGCCCTTCCGCTGATGAGATACTCAACTTTGGAATGTGGACCGTATAAAACGGCCCGGCTCCGAGCGGTAGGACACACACACCTTTGCGCATCTCCACAGCTCAACAATGAAGTGGGCAACGTGGATCCTCGCCCTCGGGCTCCTCGTGGTCCGCACCGTCGTGGCCAGAGAGGCCCCTCGGGAGCTCTGCTACGGCCACCCCGTCCACGACGACCGGCGGCCCGTCGGGCCCGCGACCGACGCCCAGCCCGTGAACCCGCTCGCCCCCGCCAACGCCACCGGGACGGACTACTCTCGCGGCTGCGAGATGCGCCTCCTGGACCCGCCTCTCGATGTCTCGTCCCGCTCCCCGGACCCCGTCAACGTGACCGTCGCCTGGTTCTTTGACGGCGGCCACTGCAAGGTGCCCCTCGTCCACCGCGAGTACTACGGCTGCCCCGGGGACGCCATGCCCTCCGTCGAGACGTGCACCGGCGGGTACTCGTACACCCGCACGCGCATCGACACCCTGATGGAGTACGCCCTCGTGAACGCCAGCCTCGTGCTGCAGCCCGGGCTGTACGACGCCGGCCTGTACATCGTCGTGCTCGTCTTTGGCGACGACGCCTACCTCGGCACCGTCTCCCTGTCGGTGGAGGCCAACCTGGACTACCCCTGCGGCATGAAGCACGGGCTCACGATCACCCGCCCCGGGGCCACCCTCCCGCCCATCGCCCCCACGGCCGGCGACCACCAGCGCTGGCGCGGGTGCTTCCCCTCGACCGACGAGGGCGCCTGGGAGAACGTGACCGCCGCCGAGAAGGGCCTGTCCGACGACTACGCCGACTACTACGACGTGCACATCTTCCGCCTGGAGTCTGACGACGAGGTCGTCCACGGCGATGCCCCCGAGGCCCCCGAGGGCGAGGAGGTGACCGAGGAGGAGGCCGAGCTGACCTCCAGCGACCTCGACAACATCGAGATCGAGGTCGTGGGCTCGCCCGCCGCTCCCGTCGAGGGCGCCGGCGACGGCGAGGAGGGGCACGGGGACGAGGAGGACGAGGAGCTGACCTCCAGCGACCTCGACAACATCGAGATCGAGGTCGTGGGCTCGCCCGCGGCCGCCCGCTTCTTCGCCGCCTCCACCACCCCCCGCGCCCCCACCCGCGCGGCCGAGATCACGACCATGACCACGGTCACCACCGTGCGGACGACCGAGGACCCCAGCGGCATCACCGACTGCCGCCGGAGCGACTTCGTCTCGCCCTCTGACATCTTCGTGACCCCCACCGGCAGCCCCGCCCTGCTCCTGGGCTTCCTGGGCAGCGCGCTCGCCTCGCGCCCCCTGCACCTGACGGCCGGGGAGACGGCCCAGCACGTGCGCGAGGCCCAGCAGAAGAGCCGCCACGTCCGCTCCCTCGGCGGCCTCCAGCTCTCGGTCGAGACCGAGACCACCAACACCACCACCACCCAGACGGGCCTGTCGGGCGACATCCGCACCTCGATCTACATCTGCGTCGCCCTCGCCGGCCTGGTCGTCGTGGGCATCGTCATCATGTGCCTCCACATGGCGATCATCAGGGCCCGGGCCCGGAACGACGGCTACCGCCACGTGGCCTCCGCCTGACCCGGCCCCGCCCGACTCCCCCGCGATTCCCCCCTCTCTCTCACCGGGTGTCCATCTTCAATAAAGTATGTCTCAAACACCTAATTTGCGTACGGCCTTGCTTACGGGGGGTGCGATCCACGCCCAGCGGTCCATAAAATTGGGTCGGCGCCCCAGGTTCCCATACACTCACCTGCCAGCGCCATGCTGCTCGCAGCGCTATTGGCGGCGCTGGTCGCCCGGACGACGCTCGGCGCGGACGTGGACGCCGTGCCCGCGCCGACCTTCCCCCCGCCCGCGTACCCGTACACCGAGTCGTGGCAGCTGACGCTGACGACGGTCCCCTCGCCCTTCGTCGGCCCCGCGGACGTCTACCACACGCGCCCGCTGGAGGACCCGTGCGGGGTGGTGGCGCTGATCTCCGACCCGCAGGTGGACCGGCTGCTGAACGAGGCGGTGGCCCACCGGCGGCCCACGTACCGCGCCCACGTGGCCTGGTACCGCATCGCGGACGGGTGCGCGCACCTGCTGTACTTTATCGAGTACGCCGACTGCGACCCCAGGCAGATCTTTGGGCGCTGCCGGCGCCGCACCACGCCGATGTGGTGGACCCCGTCCGCGGACTACATGTTCCCCACGGAGGACGAGCTGGGGCTGCTCATGGTGGCCCCGGGGCGGTTCAACGAGGGCCAGTACCGGCGCCTGGTGTCCGTCGACGGCGTGAACATCCTCACCGACTTCATGGTGGCGCTCCCCGAGGGGCAAGAGTGCCCGTTCGCCCGCGTGGACCAGCACCGCACGTACAAGTTCGGCGCGTGCTGGAGCGACGACAGCTTCAAGCGGGGCGTGGACGTGATGCGATTCCTGACGCCGTTCTACCAGCAGCCCCCGCACCGGGAGGTGGTGAACTACTGGTACCGCAAGAACGGCCGGACGCTCCCGCGGGCCTACGCCGCCGCCACGCCGTACGCCATCGACCCCGCGCGGCCCTCGGCGGGCTCGCCGAGGCCCAGGCCCCGGCCCCGGCCCAGGCCCCGGCCGAAGCCCGAGCCCGCCCCGGCGACGCCCGCGCCCCCCGGCCGCCTGCCCGAGCCGGCGACGCGGGACCACGCCGCCGGGGGGCGCCCCACGCCGCGACCCCCGAGGCCCGAGACGCCGCACCGCCCCTTCGCCCCGCCGGCCGTCGTGCCCAGCGGGTGGCCGCAGCCCGCGGAGCCGTTCCCGCCCCGGACCACCGCCGCGCCGGGCGTCTCGCGCCACCGCTCGGTGATCGTCGGCACGGGCACCGCGATGGGCGCGCTCCTGGTGGGCGTGTGCGTCTACATCTTCTTCCGCCTGAGGGGGGCGAAGGGGTATCGCCTCCTGGGCGGTCCCGCGGACGCCGACGAGCTAAAAGCGCAGCCCGGTCCGTAGCCTCCGCAGTACCGGCGTCGATGATGATGGTGGCGCGCGACGTGACCCGGCTCCCCGCGGGGCTCCTCCTCGCCGCCCTGACCCTGGCCGCCCTGACCCCGCGCGTCGGGGGCGTCCTCTTCAGGGGCGCCGGCGTCAGCGTGCACGTCGCCGGCAGCGCCGTCCTCGTGCCCGGCGACGCGCCCAACCTGACGATAGACGGGACGCTGCTGTTTCTGGAGGGGCCCTCGCCGAGCAACTACAGCGGGCGCGTGGAGCTGCTGCGCCTCGACCCCAAGCGCGCCTGCTACACGCGCGAGTACGCCGCCGAGTACGACCTCTGCCCCCGCGTGCACCACGAAGCCTTCCGCGGCTGCCTGCGCAAGCGCGAGCCGCTCGCCCGGCGCGCGTCCGCCGCGGTGGAGGCGCGCCGGCTGCTGTTCGTCTCGCGCCCGGCCTCGGGGGACGCGGGGTCGTACGTGCTGCGGGTCCGCGTGAACGGGACCACGGACCTCTTTGTGCTGACGGCCCTGGTGCCGCCGAGGGGGCGCCCCGTCCCCACGTCGCCGCCCGCGGACGAGTGCCGGCCCGTCGTCGGATCGTGGCACGACAGCCTGCGCGTCGTGGACCCCGCCGAGGACGCCGTGTTCACCACCCAGCCCCCGCCCGAGCCCGAGCCGCCGACGACCCCCGCGCCCCCCCGGGGGACCGGCGCCACCCCCGAGCCCCGATCGGACGAGGAGGAGGAGGGTGACGCGGAGACGACGACGCCGACGCTGACCCCGGCGCCCGGGACCCTGGACGCGAACGGCACGATGGTGCTGAACGCCAGCGTCGTGTCGCGCGTCCTGCTCGCCGCCGCCAACGCCACGGCGGGCGCCCGGGGCCCCGGGAAGATAGCCATGGTGCTGGGGCCCACGATCGTCGTCCTCCTGATCTTCCTGGGCGGGATCGCCTGCGTGGCCCGGCGCTGCGCGCGGAATCGCATCTACCGGCCGCGACCCGGGCGCGGATCGGCGGTCCATGCGGCGCCCCCGCGGCGCCCGCCCCCCAACCCCGTCGCCGGGGCGCCCGTCCCCCAGCCCAAGATGACGTTGGCCGAGCTGCGCCAGAAGCTCGCCACCATCGCAGAAGAACAATAAAAAGGTGGTGTTTGCATAATTTTGTGGGTGGCGTTTTATCTCCGTCCGCGCCGTTTTAAACCTGGGCACCCCCGCGAGTCTCGCACACACCGGGGTTGAGACCATGCGGCCCTTTCTGCTGCGCGCCGCGCAGCTCCTGGCGCTGCTGGCCCTGGCGCTCTCCACCGAGGCCCCGAGCCTCTCCGCCGAGACGACCCCGGGCCCCGTCACCGAGGTCCCGAGTCCCTCGGCCGAGGTCTGGGACGACCTCTCCACCGAGGCCGACGACGATGACCTCAACGGCGACCTCGACGGCGACGACCGCCGCGCGGGCTTCGGCTCGGCCCTCGCATCCCTGAGGGAGGCGCCCCCGGCCCATCTGGTGAACGTGTCCGAGGGCGCCAACTTCACCCTCGACGCGCGCGGCGACGGCGCCGTGCTGGCCGGGATCTGGACGTTCCTGCCCGTCCGCGGCTGCGACGCCGTGGCGGTGACCACGGTGTGCTTCGAGACCGCGTGCCACCCGGACCTGGTGCTGGGCCGCGCCTGCGTCCCCGAGGCCCCGGAGATGGGCATCGGCGACTACCTGCCGCCCGAGGTGCCGCGGCTCCGGCGCGAGCCGCCCATCGTCACCCCGGAGCGGTGGTCGCCGCACCTGAGCGTCCTGCGGGCCACGCCCAACGACACGGGCCTCTACACGCTGCACGACGCCTCGGGGCCGCGGGCCGTGTTCTTTGTGGCGGTGGGCGACCGGCCGCCCGCGCCGGCGGACCCGGTGGGCCCCGCGCGCCACGAGCCCCGCTTCCACGCGCTCGGCTTCCACTCGCAGCTCTTCTCGCCCGGGGACACGTTCGACCTGATGCCGCGCGTGGTCTCGGACATGGGCGACTCGCGCGAGAACTTTACCGCCACGCTGGACTGGTACTACGCGCGCGCGCCCCCGCGGTGCCTGCTGTACTACGTGTACGAGCCCTGCATCTACCACCCGCGCGCGCCCGAGTGCCTGCGCCCGGTGGACCCGGCGTGCAGCTTCACCTCGCCGGCGCGCGCGCGGCTGGTGGCGCGCCGCGCGTACGCCTCGTGCAGCCCGCTGCTCGGGGACCGGTGGCTGACCGCCTGCCCCTTCGACGCCTTCGGCGAGGAGGTGCACACGAACGCCACCGCGGACGAGTCGGGGCTGTACGTGCTCGTGATGACCCACAACGGCCACGTCGCCACCTGGGACTACACGCTCGTCGCCACCGCGGCCGAGTACGTCACGGTCATCAAGGAGCTGACGGCCCCGGCCCGGGCCCCGGGCACCCCGTGGGGCCCCGGCGGCGGCGACGACGCGATCTACGTGGACGGCGTCACGACGCCGGCGCCGCCCGCGCGCCCGTGGAACCCGTACGGCCGGACGACGCCCGGGCGGCTGTTTGTGCTGGCGCTGGGCTCCTTCGTGATGACGTGCGTCGTCGGGGGGGCCGTCTGGCTCTGCGTGCTGTGCTCCCGGCGCCGGGCGGCCTCGCGGCCGTTCCGGGTGCCGACGCGGGCGCGGACGCACATGCTCTCTCCGGTGTACACCAGCCTGCCCACGCACGAGGACTACTACGACGGCGACGACGACGACGACGAGGAGGCGGGCGTCATCCGCCGGCGGCCCGCCTCCCCCGGCGGAGACAGCGGCTACGAGGGGCCGTACGCGAGCCTGGACCCCGAGGACGAGTTCAGCAGCGACGAGGACGACGGGCTGTACGTGCGCCCCGAGGAGGCGCCCCGCTCCGGCTTCGACGTCTGGTTCCGCGATCCGGAGAAACCGGAAGTGACGAATGGACCCAACTATGGCGTGACCGCCAACCGCCTGTTGATGTCCCGCCCCGCTTAAATACCGGGAGAACCGGTCCGCCCGCATTCCGACATGCCCGGCGCCGCCTCCGTCGACATGGACACGTTTGACCCCAGCGCCCCCGTCCCGACGAGCGTCTCGAACCCGGCCGCCGACGTCCTGCTGGCCCCCAAGGGACCCCGCTCCCCGCTGCGCCCCCAGGACGACTCGGACTGCTACTACAGCGAGAGCGACAACGAGACGCCCAGCGAGTTCCTGCGCCGCGTGGGACGCCGGCAGGCGGCGCGTCGGAGACGCCGCCGCTGCCTGATGGGCGTCGCGATCAGCGCCGCCGCGCTGGTCATCTGCTCGCTGTCCGCGCTACTCGGGGGCATCGTCGCCAGGCACGTGTAGCGAGCGAGCGAGCGAACGGGAGCGGGGGCCCGCCCCCATCCGCCGCGCCCAGGAGAGGGGGAGAGAGCGGGGGGTTGGGCGCGCCACGTGGTGTGGGCACGGACTCGGACTTGTCACAATAAATGGGCCCCGGCGTGTCCGGGCGCACACAGCAGCCTTCCTCTCCTCCGCGTCTCTGTTCCGCCCGTCTCTCGCCGGACTCTTCTTCTCCACCGCCTCCACCGTCGCAGTTGTCGCGAGCGCGTTCGCACCATGGGGGTGACGGCCATCACCGTGGTCACGCTGATGGACGGGGCCGGGCGCATCCCCGCCTTCGTGGGCGAGGCGCACCCGGACCTGTGGAAGGTGCTCACCGAGTGGTGCTACGCGTCGATGGTGCAGCAGCGGCGCGCCGCCGACGAGAACTCGCCGCGGCAGCACGTGGTGCTGCGCTCCTCGGAGATCTCCCCCGGCTCGCTGGCCCTGCTGCCGCGCGCCGTGCGCCCCGTCGTGCGGACGCGGTCCGACCCCACGGCGCCGTTCTACATCACCACCGAGACGCACGAGCTGACGCGGCGCCCCCCGGCGGACGGCTCGAAGCCCGGGGAGCCCCTCAGGATCAGCCCACCCCCGCGGCTGGACACGGAGTGGTCGTCCGTCCTGAACGGGATCCAGTACCTGAACTCGGGGGCCCGGGGCACGGCCCCCGTCCACCTGTGGATCCTGGGCGCCGCCGACCTCTGCGACCAGGTGCTCCTGGCCGCCTCCCGCAGCACCGCCGCCGGAGCCTCCCACGCCCAGACGGGCGCGCGCCTGACCCGGCGCCGGCCCGGGCTGACGGACGCCGACGCCCTGGACGTGATCGTCGCCGGGATCCAGGCGACCCGCGCCATGTTCGCGCGGGTCCACAACCGCTCCTGGCGCCACGCCGGCGAGTGGACGGAGGCCCTGCACTCCCAGATCGTGACCCGGGGCGACGTGCGCCGGCGCCGAGGCGGGCGCGGCAACGGACGCGAGCGCGCCCCGCGATGTACCATCTCCTAGACGGCAGGATCTCTCCGCGTCCCCCACCCCCCCAAAAAACAAACAATAAACGCTCTCGCTCTGGCACCCGATGACACGCCTCCGTCCTCTCTCTCCCTCCCACTGACGCCACCCCTCCCCTCGCCGACAACGCCATCGTCGCCCGGCGTCGGCCGGACCGGCGGTTCTCCCCCACCCCGCCCCCCCCCCACCCCGTCCCCCCACCGCAGGACCACCATCGTCTAACTCCCACCCGGGACCACCGGGACCCTCGGGACCATCTACCTCCCACCAGGACCCGCCGGGACCACCAACACCGTCCACCTCCCACCACCACCACCATCAAGGACCCCCAACATCCCCAAGACCCTCTACTTCTTCCCACCCAGACCCCTGCCCCCGCTTCGTCCGACTCTCGCCCCCCGCGGGAGGGTTCCGCGGCTCGCTCCCCGTCTCATCCCCCCGTCTCATCCCCCCGTCTCACTCCCATCTCCCTCCCTCCACCCCGTCTCATCCCCCCATCTCCCTTCCCCACGAGGGCCGGGAGGGGAAAAAACGCCCGAGAGACGAGAGAGTTGAGGTTCGAGCGGCGGGCCGCCGTGTTTATTGAGGGCACAACAGAGGGGAGGGGGAAGGGGAAAGGGGCAAACGACGGGGCGGACGGTGGGGGTGGAGGAAGAACAGAGCGGGGCGCGGCCCGCCTCACGGGGCGGCGGCGGGCCGCGTCGGGGCGCGCGTCCCCCGGCGGGCGGCGTCCTTCCCGCCCTCCTCGCCCTCTCCGTCTTCGTCTTCTTCGCCGTCTTCACCGTCTTCCCCCCCGTCCTCCTCTTCCTCGTCCTCCTCTTCCTCGTCCTCCTCTTCCTCGTCCTCCTCTTCCTCGTCCTCCTCTTCCTCGTCCTCCTCTTCCTCGTCCTCCTCTTCCTCGTCCTCCTCTTCCTCGTCCTCGTCCCCGTCTTCCTCTTCCTCTTCCTCTTCCCCGTCCCCGCCGTCGTCCGAGCCCGAGCCCTCTCCGTCCGAGCCGTCCTCGGGGGGCTCAAACACGTCGTCGTCGCCCACGCTGGCCCCGTCAAAGTCGTCCCCGTCCTCCTCGTCCTCGTCCTCACAGAGCCCGTCCCCGTCCTCGTCGTCCTCGTCCTCGTCCTCCTCGTACACGTCCGTCTCCCCGTCCTCGTCCCCTTCCTCTTCCTCCTCGTCTTCTTCCTCTTCCCCCTCCTCGTCCTCGTCTTCCTCTTCCCCGTCTTCCTCTTCGTCCTCCCCGTCGTCCTCGCACACCGAGCCGCGGAAGCTAAACTCGGACGCGACGGTGGTGTCCGAGCCGGCGTCGTCCTCGTCGACGTCGCACTCCTCCCCAAAGAGCGGGCCCTCGATGACCGGCGGGGAGAGGACGCGCGGGTCGTAGACGTCGCGCTCGTTGAGCTCGCGCAGCGCGCTGCGCAGCGGGGCGCTCTGCTCGGGCGAGAGCTGGAGCAGGGCCTGCCAGCAGGGGGCCGAGGGGCGCTGGCGCGTGTAGCCCATGGCGTAAAAGTCAAAGAGGGCGCGGCGCAGGGCGCGCGTGTCGCCCGTCACGTCGGCGGCCGAGACGGCGATGCCGCGAAAGAGGCGGTTCACGTCGGCCCCGAGGCGCCGCGTGTCGGGGTGCCAGGGGGCCGCGCAGAAGGCGCCGGGGCCCCGCGCGCCGTGGACGCGCACCTCCTCGGGGTCGGGCGGGGGCCCGTACTCGGGGTGCTCGACGACGTAGCGCCCGCGCTTGGGGGGCGTGGGGGTCGAGGCGGGGGACGGGGGGCTGCTCCCCGGGACCGGGGTCGCGGGCCGGGGCCCGGGCGGGTCGACGGGCGAGAACGGCGGGGAGGGCACGGGCTCGTACCAGTCGGCCCAGACCCGGTCCATGGGCGCGGCGGACGCGGTGGGTCGGGCGGGGTGGTGGGAACGGGGTGGGGTGGGAGGGGGTGCGATGGGGGTGTGCGGGGGGCTGGGCTGGGACGGGGGGCCGGGTGGGTGGGCGAGGCTCCCCGACGCCGACCCGGAACCGGACCCGAGCCCGGCCGCCCCGACGGGTGGGTGGCTGGATGGATTGGATGGATTGGATGGGTGGTCGACCAGCCGGTCCCGCTCGCGTCCCTGTGGGGGGGGGGGAGAAAACGCATGACGTCAGCGTGAGCCACGACCCCCTCCCCTTCCCCCGATCCCTCCCGACCCCCGCCCACCTACCCACCCGACCCGTCCCGGGGAGGGGGAGGGGGAGGGGGGAGCCGCACCCGGGAAGCCCCCCGACTCACCTAGGCGACGGACGCGCGACCCCCGAGGCGGTTCTTCCGGAACGATGCGGCACCTGAAACGAGAACGGATACGATGAGCGCGATCGCGCGGGGTGGGTAGGGAGGGGGGACGGGGGAGGAGGTGCCACGCCCACTCCCGGCCCACCCCGACCGGGGGATCCCCCGCATCCGGTACCGTCGGGTCCCGCGATCGTCGTCGTCGAGGCCGATGCCGACGTCACCGTCGTCGTCCGGGCGTCCTCGAGCGGGGCGCGGCTCCTCGTCGGCTCGGGGCGCGCTCCGCGCCTCTCCCGGTCGATCCCCGTCGGCGTCGTTGGGGCGGCGAGTGCCGTCGGCCGGGCCGCCGCGCACCCCTTTATCGCCCCGTCGAGGGGGCCGGGCCGACGGGCCCATTGGCCGGGGCCTCACACGTGGGCTCAGGGGACGCGGGTCCGGCTCCCCCATTGGCCGGCGCGTCCCCGGAGGCGGGTGCCGCGGGGTTCGATCCCTTCGGAAAGCGGTCGATAAATTGCCAATAGGGGCACGTGGGGCCCGTGGAGAGATTTGATTGGGCCACGTCACGTTCGCACCAATTGCTAATATATACTAGTATTATATCTTATATACCGCAAGTGCGAACGCTGGACGCGTGCCAAGACTCGAGGAGGGGGCGGTCCGGCCCGCGAGACGGGCGTCCCGGGCCGGCCAATGCGGGCTCCGGGGACGCGGGTCCGGCTCCCCCATTGGCCGGCGCGTCCCCGGGGCACCCGCCCCCGGGGTTCGATCCCTTCGGAAAGCGGTCGATAAATTGCCAATACGGCCACGTGGGGCAAACGGCAGGTTTTGATTGGGCCACGTCACGTTCGCACCAATTGCTAATATATACTAGTATTATATCTTATATACAACAAGTGCGAACGCTGGACGCGTGCCAAGACTCGAGACGGCGGCGACGCGGGCTCGGGCGTTCGATCCCTTCGGAAAGCGGTCGATAAATCGCCAATACGGCCACGTGGTGCCCGTGGCCGGATTTGATTGGGCCACGTCACGTTCGCACCAATTGCTAATATATACTAGTATTATATCTTATATACCGCAAGTGCGAACGCTGGACGCGTGCCAAGACTCGAGGAGGGGGCGGTCCGGCCCGCGGGACGGGCGTCCGGGACCGGCCAATGCGGGCTCCGGGGACGCGGGTCCGGCTCCCCCATTGGCCGGCGCGTCCCCGGGGCACCCACCCCCGGGGTTCGATCCCTTCGGAAAGCGGTCGATAAATTGCCAATAGGGGCACGTGGGGCCCGTGGAGAGATTTGATTGGGCCACGTCACGTTCGCACCAATTGCTAATATATACTAGTATTATATCTTATATACAACAAGTGCGAACGCTGGACGCGTGCCAAGACTCGAGTTGGCGGCGCCGCGGGCCCCGAGACGGTCGCCGCGACCGTCCCGGAAATTTCCGGGACCGCATCGCGGGCCGGGAAGTCGCGGGAGCGCGCGTGACCCGCCGCGACCTCTCCCTTCCCGGACGCCCGCTCTCGAGGAAAAAGGGGCGCGGCGGGGCGGGGACGGGTCACCGCCGGGTCGGCCGCGCCCCGGGGGACAGGGAATCCCCCGGGACGGGTGAGTCAGGAGGGTGGGGTGGGAGCGGAGAAAGTGAGTGTGGGCGTGGGAAAACACCGGCGGGAGAGGGAGGGGGAGAGGGGGGCGGGGAGTGGGGGCGGGATGGGGGGAGGGGAGAGACGGGACCGCGCGGAGAAGGCTCGGTGCGGCCGAGGGGGGCGGCGGGGAGAGGGGACGCGGAGGGAGCGGGCGCGCCGGGAGAGGAAAGGGCGCGCTGGCCTCGAGCCAGCCCTCCGCGGCGATGAAGACGGATGAGCGGGGCGATGAATGGCCTCTGGAAAAAAGGATGAATGGGGAGGACGGATCGCGGCGGCTGCGGCCGCGGCGGCGGGGGCTGGTGGGAGGAGTGGGGGAATTGGGGGCGGGGGAAATGGGGGGAGGAGGAAGGGGGGCGGGGTGGGGGATCGCGGCACCGAGGGTCCGGAGGACGAAGAGGAACCGGGAAGAAGAAGAAGGAAAAAAAAAACACTCTCTGTGTGTGCACACGGGACCGGGGAGAGAGAGGAGAGGAGAGGGAGGGGAGGGGTGGTGGTGGTAGAGGGCGGTGGCGGTCACTCGCCTCACCCCACCCAGCCCCCTCTCCAGTTCCCGCTCTCGCTCTCTCCTTCGCCAACCTTCTCCCGCTCGCGCTCCCCGCCCCCCTCCTTCCCCCAATCCATCCATCCATCTCTCCTGGCCGAGCTCGGACCGCGGCGATGGTGAATGGGCCCGCGGCCTGGACGAGATTGATGAATGGAGAGGCGGGAGGGGGGAAGGAGGAGGGGGGGAAGAGGAGTGAGCGGGGGAAAAAAAGGGTTGCTGGCGGCGGTAGACGGATGTAGTGTACGAGGCGGCGAGTACGCCGAGATGATGGAACGAGGGCGAGCCTCTTGTTATGTGTGTGTGTGTGGGAAAGAGAGCGCGAGAGGGCGGGTGAGAGAGGTCGGGGAGGCCGGGGCCGAAATAGAAAGCGGAGAAAGCTCTCAGGGCCCGGGGTTGCGCCGGCGGGAGAGACGACGGGCGCCACAGACAGCGGCGCGGGGGAGGGTGTGTGTATGTGTGGTGTGTGTTAACCACGAAAGATGAGGAGGCGGCGGCGGCGCGAGGGCGCGAAAACCCCGACGCGCCTCGATGCGGCCCGAATCGCCGCGACGCGAGTCGACGGGGAGAGGAAGGAGGGGAGGACGGAGGGCGAGCCGCCGCTCCCCCCACCCCGCCCCGCCCCGTCCCTCTCGCCCACGCGCCCTCTCCCCCCTTCGCGCACGCTCACTCGCCCGCACCCCCGCCCCCCTCTCGTTCCGTGCCGCGCGCGCCGTCCCGCGGCCCCGTCGGGCGCAGGGATCGTAGCAAACGCGGGCGGTCGGCGTGCGCGCTGTGCGGGTGGGTGGGGGAGGGGGTTGATGACCGCGCTGGTTCCGGGAAGCCTCCCCCGGCGGGCGGAGGGGGTCGGGGCCGGGTTCGCTCTGTACCCTCTCACGCGGGCCCGGAGTGCCGCGGGCGGGGCGGGGGCAGCACTCAGCGAAGGCTCAGGGTGTTCCAGCCGATGGCCCCGGGGGCACGCGGGCGGGAGGAGTTGCGTGGGCGGAGAGGAGCGAGGAGGACTGGCGGCGGTGGAGGAGGGGGAAGAGGCGGCGAGCGGAGCGGCGCGGTAGCGCCCGCGGGTGGCCCGCGCGGTATCGCCCGCGCGGTATCGCGGCGGCCCGGGGAGAGCGGGCGGGGGACGGGGACGGGGACGGGGAGGAGGGGGGAGGAAGGGGGTCGCCACCGCGGCCCTCGCGGCCCGGGAGAAGCCTTCCCGGACGCGCCATTAGAGAGGGCTCTGTCTGCAGAAACGGTGTCTAGGCGCCGCGCCAGGGGAAACCGCAGCGGCGATAAAATAGCTTTTAGCTCGGCGCTCTCTCTGGGTCGCAGCGCCCTAAACTTGGCACGGTCCGCCGCACAGTGTGGCCCGCGTCCAGGCCGGGCCCCCGGCCTGCCGGCCTGGCGAGCGAGCGAGCGAGCGAGCCGGGGCCCCCCAGCTCGCTCCACCGTGACACCCACCCACACCGTAAACTCTCACACGGACACGCGCGCCCACCCCCCGGCGCCGCGGGCCCGACCCCGGTCCGCGCCGGCACCCCCTTTTCTCCGGCACCGAGCCGGGGGGCCGGGGCGAGGGGAAGGGGAAGGGGGGGGGACGGGGTGCGCGCGCGTGTGTATGTGTATGTGGCACCGGCATCGGTCTCCCCCCAAAACGGAGAAGGCGAGGGAGACGGAGAGGGAGGTGTGTCTCTGTGTTGTTGTTTTTTTTTCCTGCTGGCGCCGCGGTGCGCGAACGTGATCACGGGGAGGCGGCGCGCTCCGCGCTCCCCCGCCGCCTGGGCGGGGATGCCGCGCCGGCACGAGCGCACGCGGGCGCGCGGGCGGCGGGAGGGGAGGGAGGAGGGAGAAGGGAGGAGGCGGCGGTGGCGGCGGGGAAGGAGGAGGGCGGAGGAAGGGCCGGGCCGCCGAGCCGGCCGCGCCATTGGGCGCCGGGCGGAGCGGGGCGTGGAGAGGCGCCCGCGCCGGGGACGCGCCTGCGGCGGCGGGCGCGCGCCGGGCGGCTCTCCTCCTTCACCGCCGCCGCCGCGTCCTCCTCCTCCCTCCCCGCCGGCCTCGGCCCACCCGCGGCGTCACCCGCTCTCCCCCTCCTCCCCCCCTCCCTTCCCCCCGACCCCCTCCCCTCCCTCCCCTCCCCTCCCCGCGTCCTGCGTCACGGGGACCGGGGGGTCCCGCGTGATGACGCCGGGGACGCGGGACGCGCCCGCCGCCTGGGAGCCGGCGAGGAGGACAAAAAGAGTTCTGTGTCCCCTCGCCCCCCTCCGCGTCCGCAGCCGCCTCTGCAGCCGCCGCCCGCGCCGCGACCGGCGAACGCAGAGAGAGGACGCGGGGAAGAGACGCGGAGAGGGGGACGGGGGGAGAGAGAGCGAGGGGCGCGAGCGCTTCCTCCTCCACACCCGCCTTCCTCCGCCCGCCTTTCCTTCCCGGACTCGGGAGCCGCTCCCGCTCCCCCCGTTTTTCCTCCTGGCCGCGGCGACCGCCGCCGCCGCTCTCGAGGTTTTTTTTTCCCTTCCTCTCCTTCTTCCTCCTCCTCCGCCCTTGGGTGTTTCCTTCTCCGCGTCCCCATCTCCGGGGCCCGAGGCGCCGGGGCTTTCTTCCCGGGCCGCCGGGGAGGAAGCGCCCCGGCTTCTGCTTCTTCTTCTCCTCTCTCGGTAAGCACAGACTCTCCTACTACTACTACTCCTACTACTCCTACTCCTACTATATTACTATGACTACTACTATGACTACTACGGTGACTTCTCCTCCCCGCTCTAGACTTTTTTTCCTCCCCTCTCCTCCTTTCGCTCCCGTCTCCCGGGCGCCCGCGGCCGGGCGGAGGGGGAAGCGGGAAGGGGAAGCGCGGGGCGCCGCGCCTCTCCCTCCCGCTTCCCCTCCGCCGGCCCCTTCCCCGTTTCCCCGTTTCCCTTTCCCCCCCCCCTTCCCCGTCTGTTCCTTCCCCCCCCGCTTCCCCGTTTCTTCTTCTCCCCCCCGTCCCCTCTTCCTTCCCCACCCGCCCCGCTCTCCCACGCCGCGGCCGCGGGGGGCCGAAGCGGACGCGGAAGAAGACGCGGAAGAAGAGAGAGAGAGAAAGAGGGGGGCCGGCGGGGATGGGGAAGAAGGCCCGACCCGCGCGGGGGCCCCCCTCCTGTCGCCTGTCGTTTGAATGAAACCTTCCCCCGAGCGACCCCGCTCCCGCCTGTGGTGCCGTGTTTTTTTCCCGCGACGGGGAGAGCGCGAGAGTGAGGCTGTTGGCTGTGGGTTGTTGGCTGTTGGCTGTTGGCTGTTGGCTGAGGACGACGGGGAGGGGGGATGCTGCCGCGGGTGGCATGAGACGGAGGGGGCGAGGGCGGGCGGTGGAGGGGGGAGAGGAAGGTCTGGGCGCGCGTGCCGCGGGCCGAGTGTCGGGCGAGTGCGGGGCGGGTTGGGGACGGGGAGGAGGGGAGGTGTGGGGGGATGGCCGGCCGGCGCCCCCGGAGCACGCGGGGCGCCCCGGCGGGCGGGAGGGGGTCCGGGGGAAGGGTGGGGCGGTGGCGGCGGCCATCGCCCGACCCTTCCCCGGGGCGCGCGCGTGGGACCGGGACCGGGACAGGGGGCGGGGGCGGGGAGAGGAGGAGAAGGAGGAGAAGGAGGAGAAGGAGGAGGCGAACTGGGGGAAAGGCGAGACACGCCCGGGCGCCCCCCCTCCTCGGACCGAGGCGACGGCGGAGACGGAGACGGAGAGACCGCCCGCCCGGCCACCACCGTCGGTACCGGTATCACCAGCAACACCCGCACCACCGACACCCACACGGACCCTCCCACCCGCACGGGCCCCCATACCCGCACCGGCCCTCACACCCACCCACACGCACGCTCTCTCTCTCGCACACCCCCACATCACACTCTCCCCGCGCACAGACACACGGACGACACCCTCCCCGCCCGCCCCGTCCGACCGACCATCCGTCCGCCCAACCCTCCACCCGCCCGGCCCTCCGAGAGAGAGAGCGAGCCTTCTCTCCCCGCACACACACACACACTCACACCCGCAGACCTCCGGAGCAAACCCCCCCGCCGTCCCCCAGCCCGATTTTGGGCCCCCCCGTCCCGGACCCCCCGGGGGGGCGGGGGCCTCGCGCCTCCGCGTCCGGCGGGCGCGACCGAGGCCCGCGCGCCCCCGGGGGCTCGGGGCGGGACGCGGCGCCCGCGCGGGGACACTCTTTCCCCCGGAGGGGCGTCCCGGCGCGCCGGCCCGGCCCTCCCCGCCGGGAAGGGGGCCCGGCCCTCTTCGTCTCCCGGAGCGCCCCCGGGGTCCCGGCCCGAGGTCCATTTTTGGGCCCCGCCCCTTTGCACCCGCCCCCCCACCGGGCGGGGTCCGCGCTCCGACCCCCCCCTCCCCTCTGCCACGCCCCGCCACGCCCCGCCGCCATCTTGGCTCCCCTCCCCCCCAACTCCCTCTCCCCCGACGAGGCCGAGCCGGTCTCGCGCCCCCTCCCGCCCGCCCGAGGTACCGCGGAAGGACGACACGAGCCGGAAGACGGTGCAACGAGAACAACTTTTATTTTTTTTCACTCTGGGGCGTGTCTCGTGGGGTGGGAGGGGGGACCGGGAGAGAGAGGTAGAGGGGGGGTGGGTGGGCGACAAAAGTATCCAAGGCGTGGCTGTCCGGCTCCTCCCTTCCGGGTCCGGGCGGGTCCGGGCTCGAGGTTCGGGTCCGGTTCTCCCGCCCCGGCGTCGCTTCCCGGTTCGGGTCCGGTCGCCGGTCCCCGGTCCGACGGCGGTTCCGCTCGTCTCTCCGACCGGCTGGGGGCGCCCTCGGAGCGCGCGCCGAGCGCGCAGGCGCGGGTGGGGCGACCGCGGGGGTCGCGGGGGGCCTCAAGGGTCTCGGGGAGGGGGAAACGGGGGTCTCGGGGGTCTCGCGAGTGGCAGGGGGAGGCGGTGAGACCACCACACAACACAGAGACCACCACAAAACACTGAGAGACCACCACACAACACAGAGACCACCACAAAACACTGAGAGACCACCACAACACATGCGGACCACGTGCAGACACACGGAAGCTTGCACCGTGAGGCCCCATTGGCCGAGGGCGGCCACGTGGGGCGAGCATGCAAATGACGTGCACACGGAAGCTTCCCCGAAAATCATCTGATTGGCTCGCTAGCACCACGGGGGTGGGTCGCGTGCCGAGCATGCGCCTCGCGCCCGCGCGAGGGATTTTCGGACAATCTCATTGGCCGCCCGCGGCCAAGATGGCCGCAGAGCGGGCCGGGCATGCAAATCAGAGGCGCGCGGGAGACGCCTCCGCGCGCCCATTGGCCCGGGCGAGCCGAGATGGCCGCCGCGGGGGCCGGGCATGCAAAGTAGACGCGAGAGGAAGTAGGGAGAGAAATCCCATTGGCCGTCGAGGGGCCAAGATGGCGCCCTCGGGGCCGGGCATGCAAAGTAGACGCGAGAGGAAGCGGGCGAGAGAAATCCCATTGGCCGCCGTCGACCAAGATGGCCGCCGCGGGGGACGGACATGCAAAGTAGACGCGAGAGGAAGCGGGCGAGAGAAATCCCATTGGCCGTCGAGTGGGCAAGATGGCCGCCGCGGGGGCCGGGCATGCAAATGGTCCTCGCGAGGAAGTTCCTCGCGAAATCCCATTGGCCGGCGGCCGCCATCTTGGGCCGGGCATGCAAAGCAGACGCGAGAGGAAGCGGGCGAGAAAAATCCCATTGGCCGGCCGTCGGGGAAGTCCGCGGCGAAAATCGGCCATTGGTCCGCTTACCTGGGGGCGGGCTCTTCTCGGGGAGTTTATAGGCACGGTCTCCATCGTAGCACTTCACTGCGGTGCAGGTACGGACAGCATCGTTCTCTGCCAACCCGAGGGGATCCGATCGTCTCGGCTCCGGCGCGGACTCTGAAGACTCCGGCTCTCCGGCGGCTATCAGCCCTCGACGGACGGCCCGACCCACCGGGGGGGAAGAGAGAGGAGAGCCCTCTTCTTCTTCTCCTTCTTCGCCCTCTCCTTCTTCTTCTTCTGCCGCGCCCGCTCCTCCGGCTCCCTCTCACCCTCCAGCACCCGCTCTCCGAGCTCGGCTCCCGGCGCGCGAGAGAGAGAGACACCCCGAGAACCGATCGAGACCATGGCCGACGATCTCTTTGACTTCATCGAGACCGAGGGCAACTTCAGCCAGCTCCTGGCGGCGGCGGCCGCGGCCGCGGCGGCCGAGGAAGAGGGGACCGCGGCCGGCTCCGACGGCGGCAGCCAGGGCTCCCGGCGCCGCGGCCCCTCCGGCGAGGATCTCCTCTTTGGCCCCGGCGGCCTCTTCTCCGACGATGCCGGCGAGGCCGAGGCCGAGGCCGCCGTCCTCGCGGCCGCGGCCGGAGCCACGAGGCCGCCCCGTCCTCCTTCGGCCCAGCAGCAGCAGCAGCAGCAGCCCCGCCGGGGCTCGGGCGAGATCGTGGTGCTAGACGACGAAGACGAGGAGGAGGACGAGCCCGGGTCCCCGGCCGCCGGGTCCCCCGGTCGGGCTCTCCATCAGGGCTCCGAGCACGGTCACCTCGTCCTCGGGCCCCGGTCCCGGGCCGGCTCCGGGCCCCGGCCGCCGACCCCGGCAGCACTCGCAGCGGCAGAGGCCGGGGCCCCCGGCGGCCCCGGGCGCCCGTCCCCCTCCGCAGCCTCCCCGGCCTCCTCCTCCGGCTCCCCCGGCCCCTCGGCAGCCCCGCGGAGATGGTCCCCCGCGAGGGGGGACCCGGTCGGTGAGCCCGGGCCGGCGGCGCGGCCTCGGACCCCGGCGCCACCAGCACAGCCAGCAGCGGTGGCCGCAGCGCCGGCACGGCGGGGGCCCGCTTCCCCAGCCTCCCCCGCCGCCGGGCCGGTCTCGGCGCTCGGCGGCGGCGGCGCCCCCTCCGCCGGAGGGGACCGCGGTCGTCACCATCACCAGCACCGCGAGCCCCTGCTCGACGAGCCCGCCGCCGCGAGGCGGCTCGATCCTCGGCCGCTTGGCGCCCGGAGCCCCGTCTCCTCCAACTCCAACTCCAACTCCAGCTCCTCCACCACCGTCGCCGTCGAGCCCGTCGCCCGCGGCCCCGAGAAGGACGAGGACGGACTCGGCCTCGCCGGGGACGGCGGGGCGCCCCCGCAGAGACAGCCCCGCCGGCGCCGCGCCGGAGAGGGCGCCCTCCGCCGCGGCCGCGGCTTCAGCAGCTCCAGCAGCAGCGGCAGCGACTCGGACCTCTCCCCGGCCCGCTCGCCCTCCGCCCCCCGGGCCTCCGCCGCCGCCGCCGCGCGGCGCTCGGCCTCCTCCTCGTCCTCGTCCTCCTCGTCCTCCTCGTCCTCGTCCTCCTCCGAGGGAGAAGAAGACGAAGGAGTCCGGGCCGGCGCCCCACTCGCCCGCGCCGGGCCGCCACCGTCTCCGCCGGCGCCCGCCGCGGCCCCGCGGCCCTCGGCCTCCTCCGCCGCCGCCTCCTCCTCCTCCTCCTCCGCCGCGGCCTCCCCGGCCCCGGCCCCGGAGCCCGCCCGGCCCCCGCGCAGGAAGCGCCGCTCCACCAACAACCACCTCTCGCTCATGGCCGACGGGCCCCCGCCGACCGACGGGCCGCTGCTCACCCCGCTCGGGGAGCCCTGGCCCGGCTCCGACCCTCCGGCCGACGGCCGCGTCCGCTACGGCGGCGCCGGGGACTCCCGCGAGGGGCTCTGGGACGAGGACGACGTCCGCCAGGCCGCGGCCCGCTACCGCGCCGCGGCCGGGCCCGTGCCCGTCTTCATCCCGGAGATGGGGGACTCGAGGAAGCAGCACGAGGCCCTCGTGCGCCTCATCTACAGCGGGGCCGCCGGCGAGGCCATGTCCTGGCTGCAGAACCCGCGGATGCAGGCCCCCGACCAGCGCTTCAACCAGTTCTGCCAGCGCCGGGTCCACGCGCCCCACGGCCACGGCTCCTTCATCACCGGCAGCGTGACCCCGCCGCTGCCGCACATCGGGGACGCCATGGCCGCCCAGGACCCGCTCTGGGCCCTGCCGCACGCGGTGAGCGCCGTGGCCATGAGCCGCCGCTACGATCGCACCCAGAAGACCTTCATCCTGCAGAGCCTCCGCCGGGCCTACGCCGACATGGCCTACCCGGGCCGCGCCGCGGACCCCCGCGCCGGGGAGGCGACCGTCGAGGCCCTCTGCGCCCGCGTCCGCGCCGCCTTCGCCGCCGCGCAGCCCGGCCGCGTGCCCCGCGAGCTGGCGGACGCCTGCGTCCTGGCCTGCCGCGGCGTCCTCGAGCGCCTGCTGCCCGTCCCGCTCCGGCTGCCCGCGCCCGCCCGCGCCCCGGCCGCCCTCGGGCCCGCCTGCCTCGAGGAGGTGACCGCCGCGCTGCTCGCGCTCCGCGACGCGATCCCCGGGGCCGGCCCGGCCGAGCGGCAGCAGGCCGCGGACTCTGTCGCCCTCGTCGCCCGCACCGTGGCGCCGCTGGTGCGCTACAGCGTGGACGGGGCCCGCGCCCGCGAGGCCGCCTGGACCTACGCCGCGGCCCTCTTCGCCCCGGCCAACGTGGCCGCGGCCCGGCTCGCCGAGGCCGCCGCGCGGCCCGGGCCCGCGGAGCCCGCGCCCGGCCTGCCCCCGCTCTGGCCCGAGCAGCCCGGCCTCGTCGTCCCAGCGCCGGCGCCGGCGGCGGCGGGCGCCCCCTCCGGCCTCCCCGGCTCCGGGCCCTCCAGCCCCGCCAGCACCAAGTCCAGCTCCAGCACCAAGTCCAGCTCCGGCACCAAGTCCGGCAGCCCCGGCACCAAGTCCGGCCTCTCCGGCTCCTCCGGCTACGCCAGCTCCCCCGCCGCCGGCCCGGACCCAGCGCCCGAGCGGCGCAAGAAGAAGCGCCGCGCGCCCGGGGCCCGCCGCCCCGGGGACGGCGAGGAGGACGAGGGGCTCTCCGGCTCGGCCCTCCGCGGGGACGGCCACGGCCACCGCGACGACGAAGAGGACCGGGGGCCCCGGCGCAAGCGCCGCTCCCTCGGACTCGGGCCCGCTCCGGACCCCGCTCCCGCGCTGCTGTCCTCCTCCTCCTCCTCCTCCGAGGACGACCGGCTCCGGCGCCCGCTCGGGCCCATGCCCGAGCACCCCGCGCCCGACGGCGGCTTCCGCCGCGTCCCGGCCGGCGAGACCCACACGCCGCGGCCCTCCGAGGCGGCCCTCGCCGCCTACTGCCCGCCCGAGGTGGCCCGCGTCCTCGTGGACCAGGAGGTCTTCCCCGAACTCTGGCGCCCGGCGCTCACCTTCGACCCGGCCGCCCTGGCGCACATCGCCGCGCGCCGCGGGGCCGCGGGCGCCCCGCTCCGCCGCCGCGCCGCCTGGATGCGGCAGATAGCCGACCCCGAGGACGTGCGCGTGGTGGTGCTCTACGACCCGCTGCCCCACGAGGAGCTCTGCGCCGAGCCCGCCGAGGGCGCCCCGCGCCCGGCCTGGGACCCGCGCCGCGGCGGCCTCTCGGCGCTGCTCGCCGCCTTCGCCCACCGCCTCTGCACGCCGGACTCGCACGCCTGGGCCGGGAACTGGACCGGGCGCCCCGACATCGGCCGCCTCAACGCCCAGGGGGTGCTGCTGCTCTCGGCGCGGGACCTCGGCTTCGCCGGCGCCGTCGAGTACCTCTGCTCGCGGCTCGGCGCGGCGCGGCGCCGGCTCATCGTGCTGGACACCATCGAGGACTGGCCCGCGGACGGCCCGGCCGTGGGGGACTACCACGTCTACGTCCGCGCCCGCCTGGACCCGGCCGCCCAGTGCGCCGTGCGCTGGCCCGAGTGCCGCGAGCTGCGCGCGGCCGTCCTGGACTCGAGCTCCATCGTGGGCCCGGCCTGCTTCGCCCGCGTGGAGGCCTCCTTCGCCCGCCTGCACCCCGGGGCCGAGCCGCTGCGCCTCTGCCGCCAGGACAACGTGCGCTACACGGTGAGCACCCGCGCCGGGCCCCGGACCCCGGTCCCGCTGCCGCCGCGCGCCTACCGCCAGCGCGTGCTCCCCACCGTGGACGGCTGCAAGGACATGGCCCGCCAGCGCTCCGCGCTCGGGCTCGGGGACCCGGACTTTGACGCGGGCGCCGCCTTCGGGCACCGCGCGGCCAACCGCTGGGGGCTCGGCGCCCCGCTGCGGCCCGTCTTCGTCTCCTGCGGTCGCCGGGGCCTCGCCGAGCTCCGCGGCCCCGAGGGCCTGCCGGCCGAGCTGCGCGCCTTCTGCGCCGCGGCGCTGCTGGAGCCCGACGCCGAGGCCGCCCCGCTGGTGCTGGCCCCGGGCGCCCTCGCGGCCGCGGGCGCCCCGCCGGCCGTGCGCTGGGACTTTGCGCCCTTCGAGACCAGCGTCCGCGCCGCCGGCGGCGCCGTGGAGACCCACCGGCCCTCGGGCGCCCGGGGGCCCGGCGAGGACGGGGAGGACTCTGCCGCGGTGGAGATCGTCGGCTTCCGCGGCGGCGACGGCCGGCCCCGCGGCCCGCTCGGGCCCATCAAGGTGGAGGCCATCTCGGACGACGAGGAGGCCGAGGACGCCGGCAACCCCTACCTGCTGCTCCGCTGAGCGGGGCGCCCCCTCGGCCCGGCTGGACTCTGACTCTGACTCTCCGGCCCCTCCACCGGCTCCTCGAGGCCCTTCTCCTCCGTCTTCTCTCCCCTCGCCCCCGGCCCGGTCCTCGTCCTCGTCCCCGTCCTCCTCCTCCTCCTCCTCCTCCTCCTCCGCGTCCGCGGCGGCGGCCTCGGTCCCCTCGGCCCGGCGGCGCTTGCCTCCCCGGCGCCTGCCTCCCCGGCCCGGCGGCCCTCCTCCTCCTCCTCCTCTCCTTCTCCTCCGCGCGGATCCCCCGGCCGGAGGCGGCTGCGGCGGCGGCGGAGGTGGCGGCGGTGGTGGCGGCGGTGGTGGAAGCGGCGGCGGCGGCCGCGGCGGAGGGCTCGGCGGCGGAGGATCGTCCCGGTCCCCTTCTCCTCCTCCTCCTCCTCCCGCGGTCCCCCGGTCCCCTTCTCCTCCTTCTCCCATCGGGTGAGAAAAGAGTTTATTTTCAGAGTGAGAAAATAAAGTTTGTGCTGTATTTTCTGAACCAGCTCGAGTCTCTGAGATTTTTTGGGGAGATGGAGGCGGCCATCTTGGCGGTGGTCTCTGGGGCGGAGGTGGTCTTGTGGATGGGGGTCCCTGGTGGGAGGAAGAAGAAGAGGTGGAGGGTCTTGGTGGGGGTGATGGGGGTCCTCCTCCTGGAGGGTCTTGGTGGTGGTGATGGGAAGAAGTGGATGGGGGTCCTCCTCCTGGTGGGTCTTGGTGGTGGTGGGTCTCAGCAGATGGGGGTCCTGGTGGGTCCTGGTGGGTCTCAGCAGATGGGGGTCCTCCTCCTGGAGGGTCTTGGTGGTGGTGGTGGTGGGTCTCAGCAGATGAGGGTCCCGGTGGGTCCTGGTGGGTCTCAGCAGATGGGGGTCCTCCTCCTGGTGGGTCTGGGTGGGAAGAAGTAGAGGGTCTTGGGGATGTTGGGGGTCCTTGATGGTGGTGGTGGTGGGAGGTGGACGGTGTTGGTGGTCCCGGCGGGTCCTGGTGGGAGGTAGATGGTCCCGAGGGTCCCGGTGGTCCCGGGTGGGAGTTAGACGATGGTGGTCCTGCGGTGGTCGAGGGGGGGGGAAGGAAAGAGAGAGAGAGAAAAAAACACGGTGGTGGTCAGTACAGGGAACTGCCATTGCAACCATCGCCCCCTGGTGGTGATAGTAGTAAAGTGTGTGCGTGGGGGGGGGGGGGGGGGGGTGGTGGTGGTGGTGTTGTTGGTGGTGGTGGTCCCGGGTGGTCGTCGTGGAGGGGCGGGAAAGGCGGTGGGAGAGCGGGCGGAACAGGTTCATCTGGCTTCCGTCTTCCGTCCCCACTCCCAGCCTCCCCCGCTGCCACCCGATTGGCCGGCGGGACGATGACGACGGCGACCGCGTGGCGCGAGGGGAGCCAATGGGGCGGGTGCCGCGGAGCGGAAGCCCCCGCCCGCGGACCCCCGGTGCGACGGAAGGGGCAGGCTCGGCGCGCCGATGCGATCCGCGGAGCCCGCCCCGTCCGCGCCCCGGAAGCCGGCGTCACGTCCGGGGGAAGCGGAAGGGGCCGGCGCGATATGCAGATGAGATCCGTGGGGACCGGCGTCACGTCCGGCGGAAGTTGCGCGCGGGAAGCGGTCATACGCAAATGAGGCGGAAGTCGTCATCGGCAAAAACGTCACGTCCGGGAAGCGGTCATATGCAAATGGGGGGGGGGTGGGAGGAGCCCGGCGTCACGTCCGGGGGAAGCGGTCATATGTAAATGAGGGGGGAGGGGCCAGAGCGTCACTTCCGGAGGAAGTTGTCATATGCAGATTAGGGGGAGGGGTTATGCAGATTAGGGGGAGGGGTTATGCAGATTAGGGGGAGGGGTTATGCAGATTAGGGGGAGGGGTTATGCAGATTAGGGGGAGGGGTTAT
