# Supplementary material for: Pseudorabies virus gM and its homologous proteins in herpesviruses induce mitochondria-related apoptosis involved in viral pathogenicity
Source: PLoS Pathog. 2024 Apr 26;20(4):e1012146. doi: 10.1371/journal.ppat.1012146 (PMC11051632; doi:10.1371/journal.ppat.1012146)
Supplement: S3 Appendix — (HTM) [file ppat.1012146.s015.htm]

PRV.genome.mafft.fa


## Gblocks 0.91b Results

Processed file: **PRV.genome.mafft2.fa**  
Number of sequences: **2**  
Alignment assumed to be: **DNA**  
New number of positions: **143466** (selected positions are underlined in blue)

```
                         10        20        30        40        50        60        70        80        90       100       110       120       130       140       150       160       170       180       190       200       210       220       230       240       250       260       270       280       290       300
                 =========+=========+=========+=========+=========+=========+=========+=========+=========+=========+=========+=========+=========+=========+=========+=========+=========+=========+=========+=========+=========+=========+=========+=========+=========+=========+=========+=========+=========+=========+
PRV-WT complete  ccccccagcccctccgctccccctttcccccagcccctccgctccccctttccccccagccccctccgctcccccctttccccccagccccctccgctcccccctttcccccccagccccctccgctcccccctttccccccctttctaagtggacgccgcgggcgcgcgggcccccacacggcgttcgctctcgtcccggccggtcgggccgccgggacggggcgctcggcgccgcgatgaccgacgcctcgtcgcggcccggcggggaagagccgatggaggtggacgagcacgtcgg
PRV-delete UL10  ccccccagcccctccgctccccctttcccccagcccctccgctccccctttccccccagccccctccgctcccccctttccccccagccccctccgctcccccctttcccccccagccccctccgctcccccctttccccccctttctaagtggacgccgcgggcgcgcgggcccccacacggcgttcgctctcgtcccggccggtcgggccgccgggacggggcgctcggcgccgcgatgaccgacgcctcgtcgcggcccggcggggaagagccgatggaggtggacgagcacgtcgg
                 ############################################################################################################################################################################################################################################################################################################


                        310       320       330       340       350       360       370       380       390       400       410       420       430       440       450       460       470       480       490       500       510       520       530       540       550       560       570       580       590       600
                 =========+=========+=========+=========+=========+=========+=========+=========+=========+=========+=========+=========+=========+=========+=========+=========+=========+=========+=========+=========+=========+=========+=========+=========+=========+=========+=========+=========+=========+=========+
PRV-WT complete  gccggagcccgagcccatggaggtggacgagccccccgcaccgccgtcggcaccggcaccgacggcggcgggcaccatcctcgcggtgggcttcacccagcccgcgcaggtcctgcgggcgtacccgatcggttgaggtgcgaacgatgggcgggggatgcccggggtcggacgcgctcacgtggtgccccacccaccccacgccgcgctcttttttcccgggccgcccgaccggatcctcccgcagaggcggaggtgaggccggagagagacagggagagatgatggacgcggagaggg
PRV-delete UL10  gccggagcccgagcccatggaggtggacgagccccccgcaccgccgtcggcaccggcaccgacggcggcgggcaccatcctcgcggtgggcttcacccagcccgcgcaggtcctgcgggcgtacccgatcggttgaggtgcgaacgatgggcgggggatgcccggggtcggacgcgctcacgtggtgccccacccaccccacgccgcgctcttttttcccgggccgcccgaccggatcctcccgcagaggcggaggtgaggccggagagagacagggagagatgatggacgcggagaggg
                 ############################################################################################################################################################################################################################################################################################################


                        610       620       630       640       650       660       670       680       690       700       710       720       730       740       750       760       770       780       790       800       810       820       830       840       850       860       870       880       890       900
                 =========+=========+=========+=========+=========+=========+=========+=========+=========+=========+=========+=========+=========+=========+=========+=========+=========+=========+=========+=========+=========+=========+=========+=========+=========+=========+=========+=========+=========+=========+
PRV-WT complete  gagcggccgccgcggggcgcgtggaggtggagctcgatgcccggccctccggcgccggagggcgcggcgcgagcccctcgcgccgggcgcgggacccccgaaccagcccgtgctcccggggccgagccgaccggcgttcccgggacagaaccggcgacgtcaccgggccgcgggcagcgcggcccgccaccctgggtattaacgtcccgggcaccgccgtccgccgccagccgccgtgggaggcagacatgcctccacaacgagcccgcggggctccgccgcgccgtcgcggcagcgacc
PRV-delete UL10  gagcggccgccgcggggcgcgtggaggtggagctcgatgcccggccctccggcgccggagggcgcggcgcgagcccctcgcgccgggcgcgggacccccgaaccagcccgtgctcccggggccgagccgaccggcgttcccgggacagaaccggcgacgtcaccgggccgcgggcagcgcggcccgccaccctgggtattaacgtcccgggcaccgccgtccgccgccagccgccgtgggaggcagacatgcctccacaacgagcccgcggggctccgccgcgccgtcgcggcagcgacc
                 ############################################################################################################################################################################################################################################################################################################


                        910       920       930       940       950       960       970       980       990      1000      1010      1020      1030      1040      1050      1060      1070      1080      1090      1100      1110      1120      1130      1140      1150      1160      1170      1180      1190      1200
                 =========+=========+=========+=========+=========+=========+=========+=========+=========+=========+=========+=========+=========+=========+=========+=========+=========+=========+=========+=========+=========+=========+=========+=========+=========+=========+=========+=========+=========+=========+
PRV-WT complete  cgcccgatccaggcagcctcgccgggcggccctcgcccggggggagaggaggcggagggcagcgccgccccctctcgcgcagcagctcgctcacgtccgtcgcctcggcgcccgtggagacgcccgtcgtcgcggaggctccggggctcggcgcccccggctccaggccgccctcctacggggacgtcgtccgcgtcgggccgcgcccgcaccgatcgccggacacgccgctgtttgcccggggcccgcccccgtcctactcggagacgctcctgttcgacccgcccgcgtacgcggtga
PRV-delete UL10  cgcccgatccaggcagcctcgccgggcggccctcgcccggggggagaggaggcggagggcagcgccgccccctctcgcgcagcagctcgctcacgtccgtcgcctcggcgcccgtggagacgcccgtcgtcgcggaggctccggggctcggcgcccccggctccaggccgccctcctacggggacgtcgtccgcgtcgggccgcgcccgcaccgatcgccggacacgccgctgtttgcccggggcccgcccccgtcctactcggagacgctcctgttcgacccgcccgcgtacgcggtga
                 ############################################################################################################################################################################################################################################################################################################


                       1210      1220      1230      1240      1250      1260      1270      1280      1290      1300      1310      1320      1330      1340      1350      1360      1370      1380      1390      1400      1410      1420      1430      1440      1450      1460      1470      1480      1490      1500
                 =========+=========+=========+=========+=========+=========+=========+=========+=========+=========+=========+=========+=========+=========+=========+=========+=========+=========+=========+=========+=========+=========+=========+=========+=========+=========+=========+=========+=========+=========+
PRV-WT complete  ccatcccggacccgccggcgtacgagcccaccgtcatcgggccgcacccgccgcgcccccgcgactggatctcctcgccctcggtggtgcagccgtcgctgctgggccccttcagccagtgcctcccgcgggtgacctgccccgactgccgctaccccgaagaccgcccgatggtgctcgtgggcttcctctgggggggactgctcctgctggtgggcctcgtgtttctgatcctgctcccggtgctccgggagtccgtcgtgtttccctgatcaataaactcggctcgcccgccccatc
PRV-delete UL10  ccatcccggacccgccggcgtacgagcccaccgtcatcgggccgcacccgccgcgcccccgcgactggatctcctcgccctcggtggtgcagccgtcgctgctgggccccttcagccagtgcctcccgcgggtgacctgccccgactgccgctaccccgaagaccgcccgatggtgctcgtgggcttcctctgggggggactgctcctgctggtgggcctcgtgtttctgatcctgctcccggtgctccgggagtccgtcgtgtttccctgatcaataaactcggctcgcccgccccatc
                 ############################################################################################################################################################################################################################################################################################################


                       1510      1520      1530      1540      1550      1560      1570      1580      1590      1600      1610      1620      1630      1640      1650      1660      1670      1680      1690      1700      1710      1720      1730      1740      1750      1760      1770      1780      1790      1800
                 =========+=========+=========+=========+=========+=========+=========+=========+=========+=========+=========+=========+=========+=========+=========+=========+=========+=========+=========+=========+=========+=========+=========+=========+=========+=========+=========+=========+=========+=========+
PRV-WT complete  gatattcgcgtctcgtgtggtgctgcgacgagggggggaagaggtgggacgcgaagagatgaagggatcggggggaaagacggcgagaggaggagcgggagtgtgcggaagggacggtgaagcggcggtggtggagggagatggggagatggagggagatggagggagatggggagatggagggagatggagggagatggagggagatggggagatggagggagatggggagatggagggagatggagggagatggagggagatggggagatggagggagatggagggagatggagggag
PRV-delete UL10  gatattcgcgtctcgtgtggtgctgcgacgagggggggaagaggtgggacgcgaagagatgaagggatcggggggaaagacggcgagaggaggagcgggagtgtgcggaagggacggtgaagcggcggtggtggagggagatggggagatggagggagatggagggagatggggagatggagggagatggagggagatggagggagatggggagatggagggagatggggagatggagggagatggagggagatggagggagatggggagatggagggagatggagggagatggagggag
                 ############################################################################################################################################################################################################################################################################################################


                       1810      1820      1830      1840      1850      1860      1870      1880      1890      1900      1910      1920      1930      1940      1950      1960      1970      1980      1990      2000      2010      2020      2030      2040      2050      2060      2070      2080      2090      2100
                 =========+=========+=========+=========+=========+=========+=========+=========+=========+=========+=========+=========+=========+=========+=========+=========+=========+=========+=========+=========+=========+=========+=========+=========+=========+=========+=========+=========+=========+=========+
PRV-WT complete  atggggagatggggagatggggagatggggagatggggagatggggagatggagggagatggggagcgacgacgacagactcgcgcacgccagagaggtacggttcaacggttttattcaaaacaggtggttgcagtaaaagtacttcccgtgcatgtacacggggacgagggtgtagctgcacgtcaggcggcaggtgggggcgtcgcagccggtcttgtgggcgtccagcgcgtccatgatcccgccgaggcaggcgccgggcacgtactcctccagcggggcgggcgagtcccccag
PRV-delete UL10  atggggagatggggagatggggagatggggagatggggagatggggagatggagggagatggggagcgacgacgacagactcgcgcacgccagagaggtacggttcaacggttttattcaaaacaggtggttgcagtaaaagtacttcccgtgcatgtacacggggacgagggtgtagctgcacgtcaggcggcaggtgggggcgtcgcagccggtcttgtgggcgtccagcgcgtccatgatcccgccgaggcaggcgccgggcacgtactcctccagcggggcgggcgagtcccccag
                 ############################################################################################################################################################################################################################################################################################################


                       2110      2120      2130      2140      2150      2160      2170      2180      2190      2200      2210      2220      2230      2240      2250      2260      2270      2280      2290      2300      2310      2320      2330      2340      2350      2360      2370      2380      2390      2400
                 =========+=========+=========+=========+=========+=========+=========+=========+=========+=========+=========+=========+=========+=========+=========+=========+=========+=========+=========+=========+=========+=========+=========+=========+=========+=========+=========+=========+=========+=========+
PRV-WT complete  gtcctcgggcagcacgcagctcgagccgcgctcgacgacgcgggcgatgcgggccagcatcaccagggtgtaggtgacgatgcagcgcacgtcccgcaggcggcgccggcgcacgagctcgtcgacgggcgtgtcgcggaggtagcagcgcaggaacgggccgagcttcaggcgcaggttctggagcaccgcccccgcggtggccacgatggggtccagcgtgcgcagcgtgagccccttggtgaggatgagcttgacccacgtgagcgtctcgtccgccgaggcgagggactccgtcag
PRV-delete UL10  gtcctcgggcagcacgcagctcgagccgcgctcgacgacgcgggcgatgcgggccagcatcaccagggtgtaggtgacgatgcagcgcacgtcccgcaggcggcgccggcgcacgagctcgtcgacgggcgtgtcgcggaggtagcagcgcaggaacgggccgagcttcaggcgcaggttctggagcaccgcccccgcggtggccacgatggggtccagcgtgcgcagcgtgagccccttggtgaggatgagcttgacccacgtgagcgtctcgtccgccgaggcgagggactccgtcag
                 ############################################################################################################################################################################################################################################################################################################


                       2410      2420      2430      2440      2450      2460      2470      2480      2490      2500      2510      2520      2530      2540      2550      2560      2570      2580      2590      2600      2610      2620      2630      2640      2650      2660      2670      2680      2690      2700
                 =========+=========+=========+=========+=========+=========+=========+=========+=========+=========+=========+=========+=========+=========+=========+=========+=========+=========+=========+=========+=========+=========+=========+=========+=========+=========+=========+=========+=========+=========+
PRV-WT complete  gccctcgttcaggagcaccatctcgcggagcacgcgcgccgcctcggtggcgtgcggccgcacctcaaacaggcggtgcaggtcggccccgtggaacatgagcgtctcccacgtgacgcgccgcccgtccggggtgaactgctcggcgccgaactcgaggacggcggcccacggcgacgagcccgcggcgcggaagccgtcgttgaacacggggcgggagaggtcgcgccgcgccgaggcgaagaggtcgttgacgcgcgtcgcgccgacccgcgcgtgcgtcgcggagacggtggcgcg
PRV-delete UL10  gccctcgttcaggagcaccatctcgcggagcacgcgcgccgcctcggtggcgtgcggccgcacctcaaacaggcggtgcaggtcggccccgtggaacatgagcgtctcccacgtgacgcgccgcccgtccggggtgaactgctcggcgccgaactcgaggacggcggcccacggcgacgagcccgcggcgcggaagccgtcgttgaacacggggcgggagaggtcgcgccgcgccgaggcgaagaggtcgttgacgcgcgtcgcgccgacccgcgcgtgcgtcgcggagacggtggcgcg
                 ############################################################################################################################################################################################################################################################################################################


                       2710      2720      2730      2740      2750      2760      2770      2780      2790      2800      2810      2820      2830      2840      2850      2860      2870      2880      2890      2900      2910      2920      2930      2940      2950      2960      2970      2980      2990      3000
                 =========+=========+=========+=========+=========+=========+=========+=========+=========+=========+=========+=========+=========+=========+=========+=========+=========+=========+=========+=========+=========+=========+=========+=========+=========+=========+=========+=========+=========+=========+
PRV-WT complete  cgcgctctccgacaggcggtcggggggcgcgtcgggcccgctgtcgggacgatccgcctccggccgccgccgctggtgctgctgctggcgctgctgctgctgttgctgtcgctgccgctgtcggagggcctgtcgggcggcgcgcccgccgcgacccccgcgccggcgccggacggccccgagccgcgcccggacgggcggccgctcggaggggcgcgcgcccaggcgctcgcggaccgggcgccgctcctcgctccccgaccgggacgcctcgctgccgctgctgttgccgctgtcctc
PRV-delete UL10  cgcgctctccgacaggcggtcggggggcgcgtcgggcccgctgtcgggacgatccgcctccggccgccgccgctggtgctgctgctggcgctgctgctgctgttgctgtcgctgccgctgtcggagggcctgtcgggcggcgcgcccgccgcgacccccgcgccggcgccggacggccccgagccgcgcccggacgggcggccgctcggaggggcgcgcgcccaggcgctcgcggaccgggcgccgctcctcgctccccgaccgggacgcctcgctgccgctgctgttgccgctgtcctc
                 ############################################################################################################################################################################################################################################################################################################


                       3010      3020      3030      3040      3050      3060      3070      3080      3090      3100      3110      3120      3130      3140      3150      3160      3170      3180      3190      3200      3210      3220      3230      3240      3250      3260      3270      3280      3290      3300
                 =========+=========+=========+=========+=========+=========+=========+=========+=========+=========+=========+=========+=========+=========+=========+=========+=========+=========+=========+=========+=========+=========+=========+=========+=========+=========+=========+=========+=========+=========+
PRV-WT complete  catgaccgtggtgcgagcggggccgcggtgtgccgccggcgggcggcggggcgcctttaaccccgcgcccccgcgcgaccttcatccaaatatggtaatgtggcgctcgtagtgcagaaacagcaacacgccccctaccagcaggaggaggtacagcgccttggcaaagatcccggcgagcacggtcgagcagcaggcgccgcagacgccgagggcggcgcgcccgccgtcggcgcgcgcgggcggggccgtcgcgccggagacgcgcgcgatgtgcggcagcgtcgccgccaggagctc
PRV-delete UL10  catgaccgtggtgcgagcggggccgcggtgtgccgccggcgggcggcggggcgcctttaaccccgcgcccccgcgcgaccttcatccaaatatggtaatgtggcgctcgtagtgcagaaacagcaacacgccccctaccagcaggaggaggtacagcgccttggcaaagatcccggcgagcacggtcgagcagcaggcgccgcagacgccgagggcggcgcgcccgccgtcggcgcgcgcgggcggggccgtcgcgccggagacgcgcgcgatgtgcggcagcgtcgccgccaggagctc
                 ############################################################################################################################################################################################################################################################################################################


                       3310      3320      3330      3340      3350      3360      3370      3380      3390      3400      3410      3420      3430      3440      3450      3460      3470      3480      3490      3500      3510      3520      3530      3540      3550      3560      3570      3580      3590      3600
                 =========+=========+=========+=========+=========+=========+=========+=========+=========+=========+=========+=========+=========+=========+=========+=========+=========+=========+=========+=========+=========+=========+=========+=========+=========+=========+=========+=========+=========+=========+
PRV-WT complete  cagcgccccgaccgccagcacaaagagccacacgagggcgcgggcgtacagcgggaagacgagggcgcacggggcgcgcgtgacgccgagggtcgtgaacagcgccatgcgcgcccccaggcggagcccgatctcgaggaggatgagcgcgatcagcgtggggtggcggtgcgccagcgtgatggggtcctcgcggaggtcgcggctgagcgcggcgcggcgcgtcgcgagctcggccatgtaccaggcgaacttggtgtagctgcagccgatgaccgtggcggcgagcacgctcgccgc
PRV-delete UL10  cagcgccccgaccgccagcacaaagagccacacgagggcgcgggcgtacagcgggaagacgagggcgcacggggcgcgcgtgacgccgagggtcgtgaacagcgccatgcgcgcccccaggcggagcccgatctcgaggaggatgagcgcgatcagcgtggggtggcggtgcgccagcgtgatggggtcctcgcggaggtcgcggctgagcgcggcgcggcgcgtcgcgagctcggccatgtaccaggcgaacttggtgtagctgcagccgatgaccgtggcggcgagcacgctcgccgc
                 ############################################################################################################################################################################################################################################################################################################


                       3610      3620      3630      3640      3650      3660      3670      3680      3690      3700      3710      3720      3730      3740      3750      3760      3770      3780      3790      3800      3810      3820      3830      3840      3850      3860      3870      3880      3890      3900
                 =========+=========+=========+=========+=========+=========+=========+=========+=========+=========+=========+=========+=========+=========+=========+=========+=========+=========+=========+=========+=========+=========+=========+=========+=========+=========+=========+=========+=========+=========+
PRV-WT complete  gtagttgagcgtgtacttctccggggtcaggaactcggcggggtcgcggaaggggccgaacatgcgccgctcctggcgcgcgtacacgaaggccgcgtagaggagccacgcgagggccgccaggcgaaagtgcacgtcccacaggtaggcgcggcagtcgcggcgcgcgtacacgacccgcgcgcggtcgcgcagctccgggctcagcgcgcccgtctcgttggggcccacgtagatggccgtggcgttgaaggcctcccagtccggcgcgccgccgccggcgtgctcgccgagcggcag
PRV-delete UL10  gtagttgagcgtgtacttctccggggtcaggaactcggcggggtcgcggaaggggccgaacatgcgccgctcctggcgcgcgtacacgaaggccgcgtagaggagccacgcgagggccgccaggcgaaagtgcacgtcccacaggtaggcgcggcagtcgcggcgcgcgtacacgacccgcgcgcggtcgcgcagctccgggctcagcgcgcccgtctcgttggggcccacgtagatggccgtggcgttgaaggcctcccagtccggcgcgccgccgccggcgtgctcgccgagcggcag
                 ############################################################################################################################################################################################################################################################################################################


                       3910      3920      3930      3940      3950      3960      3970      3980      3990      4000      4010      4020      4030      4040      4050      4060      4070      4080      4090      4100      4110      4120      4130      4140      4150      4160      4170      4180      4190      4200
                 =========+=========+=========+=========+=========+=========+=========+=========+=========+=========+=========+=========+=========+=========+=========+=========+=========+=========+=========+=========+=========+=========+=========+=========+=========+=========+=========+=========+=========+=========+
PRV-WT complete  cgccgcgtacacgacggggtggggcaggcgggcgacggtcgcgtagtacgcgcccaggccggcgtacgcgcccatcaccccgaggacgaggaggtgcagcgggcgtccgccgaggagcatgattgccccacctccgcggtgaaaatggtgcgcgtcgcgttgctgccgcactttgtagcgaagcactgctgactcagcgagatgcacagccggtcgtgggcgtcgacgctcagggccacgaaggtgcgggccggcgggctgcgcgcgtgccgggcccgcaggcagctgaagcgcgcgggc
PRV-delete UL10  cgccgcgtacacgacggggtggggcaggcgggcgacggtcgcgtagtacgcgcccaggccggcgtacgcgcccatcaccccgaggacgaggaggtgcagcgggcgtccgccgaggagcatgattgccccacctccgcggtgaaaatggtgcgcgtcgcgttgctgccgcactttgtagcgaagcactgctgactcagcgagatgcacagccggtcgtgggcgtcgacgctcagggccacgaaggtgcgggccggcgggctgcgcgcgtgccgggcccgcaggcagctgaagcgcgcgggc
                 ############################################################################################################################################################################################################################################################################################################


                       4210      4220      4230      4240      4250      4260      4270      4280      4290      4300      4310      4320      4330      4340      4350      4360      4370      4380      4390      4400      4410      4420      4430      4440      4450      4460      4470      4480      4490      4500
                 =========+=========+=========+=========+=========+=========+=========+=========+=========+=========+=========+=========+=========+=========+=========+=========+=========+=========+=========+=========+=========+=========+=========+=========+=========+=========+=========+=========+=========+=========+
PRV-WT complete  ccgcagagctggaagagcacccagtcgggcttggccaccacccgctgcgcggtcgcgcccgcgtactcgtgcgcgcgcccgttgaagtgcgcgtcgaggtgccgcagcacgggcgccatggccacgtccgccacgaaggcctcgagcgcgcgccggtcctccgcgtccacggccaggccgcgcagcatgtccgccagcggcgtgcgccgggcgaagaaggcctcgcggttccgcgccgccttgcgctcgaagaagctctcgtactccccgccgaggctgtgcagcacgcgcgtgacggcg
PRV-delete UL10  ccgcagagctggaagagcacccagtcgggcttggccaccacccgctgcgcggtcgcgcccgcgtactcgtgcgcgcgcccgttgaagtgcgcgtcgaggtgccgcagcacgggcgccatggccacgtccgccacgaaggcctcgagcgcgcgccggtcctccgcgtccacggccaggccgcgcagcatgtccgccagcggcgtgcgccgggcgaagaaggcctcgcggttccgcgccgccttgcgctcgaagaagctctcgtactccccgccgaggctgtgcagcacgcgcgtgacggcg
                 ############################################################################################################################################################################################################################################################################################################


                       4510      4520      4530      4540      4550      4560      4570      4580      4590      4600      4610      4620      4630      4640      4650      4660      4670      4680      4690      4700      4710      4720      4730      4740      4750      4760      4770      4780      4790      4800
                 =========+=========+=========+=========+=========+=========+=========+=========+=========+=========+=========+=========+=========+=========+=========+=========+=========+=========+=========+=========+=========+=========+=========+=========+=========+=========+=========+=========+=========+=========+
PRV-WT complete  tgcgccgggcgggcgtggaagtgaaagttgcgcgggtcgtcgaagcccgggggcgcgtccccgaaggggagcaggcgcccgtggcagccgccgccgtcgaccttggcgaagaagggcagccgcaggctgcgcccgtgcgcgtacacgcccgtgtccacgaaggtgaagtcctggaggtacgggcccatgggctcgataaagtcccgctccagcagcacggcctgctggagcacacgcgccaggccgcggaccgtgtcggcgccggccaccgcgtacggcgacggcacgggcgtgcacacg
PRV-delete UL10  tgcgccgggcgggcgtggaagtgaaagttgcgcgggtcgtcgaagcccgggggcgcgtccccgaaggggagcaggcgcccgtggcagccgccgccgtcgaccttggcgaagaagggcagccgcaggctgcgcccgtgcgcgtacacgcccgtgtccacgaaggtgaagtcctggaggtacgggcccatgggctcgataaagtcccgctccagcagcacggcctgctggagcacacgcgccaggccgcggaccgtgtcggcgccggccaccgcgtacggcgacggcacgggcgtgcacacg
                 ############################################################################################################################################################################################################################################################################################################


                       4810      4820      4830      4840      4850      4860      4870      4880      4890      4900      4910      4920      4930      4940      4950      4960      4970      4980      4990      5000      5010      5020      5030      5040      5050      5060      5070      5080      5090      5100
                 =========+=========+=========+=========+=========+=========+=========+=========+=========+=========+=========+=========+=========+=========+=========+=========+=========+=========+=========+=========+=========+=========+=========+=========+=========+=========+=========+=========+=========+=========+
PRV-WT complete  cggaagcccatcttggcgcggcagccgcacgccgggcgctcgtccgccgcgggggcgcgcgcggggccgggttcggggtcgggggcggcggcggaggaggagccggggatcccggccccgacctcgtcgtcgtcgcacgtgtacacctcggcgggggcgcccgcgtcccagtccgcccagtcctcgccgtccggcgcggcgccaaagtcggcgggggcggcgacgtcgtcgtcccagggcgccccctcgaggccgtcgtcggaggaggccgcggcggcggaccccgcggcgccggcgccc
PRV-delete UL10  cggaagcccatcttggcgcggcagccgcacgccgggcgctcgtccgccgcgggggcgcgcgcggggccgggttcggggtcgggggcggcggcggaggaggagccggggatcccggccccgacctcgtcgtcgtcgcacgtgtacacctcggcgggggcgcccgcgtcccagtccgcccagtcctcgccgtccggcgcggcgccaaagtcggcgggggcggcgacgtcgtcgtcccagggcgccccctcgaggccgtcgtcggaggaggccgcggcggcggaccccgcggcgccggcgccc
                 ############################################################################################################################################################################################################################################################################################################


                       5110      5120      5130      5140      5150      5160      5170      5180      5190      5200      5210      5220      5230      5240      5250      5260      5270      5280      5290      5300      5310      5320      5330      5340      5350      5360      5370      5380      5390      5400
                 =========+=========+=========+=========+=========+=========+=========+=========+=========+=========+=========+=========+=========+=========+=========+=========+=========+=========+=========+=========+=========+=========+=========+=========+=========+=========+=========+=========+=========+=========+
PRV-WT complete  ccgggcggacaggcgctcttgtacacgtagcacgggtgcgcggcccagtcgccgtcggcctcggggaagagcagcgccagcgcgtcgagcaccgcgcggcggaacccgcgcatggccgcctcgagcgtgcccgcgggcaccgggcgcgcgaggcgaaagtcgacgtcgaggacgatgttggtcacggccagccgggcgttgaacacctcgtggcggctcacgtacatctgcgccgccggctcgtgcgccgccatctccggcgccgcgtcgcgccgcgccgtcgcgcgcccccaggcgtcc
PRV-delete UL10  ccgggcggacaggcgctcttgtacacgtagcacgggtgcgcggcccagtcgccgtcggcctcggggaagagcagcgccagcgcgtcgagcaccgcgcggcggaacccgcgcatggccgcctcgagcgtgcccgcgggcaccgggcgcgcgaggcgaaagtcgacgtcgaggacgatgttggtcacggccagccgggcgttgaacacctcgtggcggctcacgtacatctgcgccgccggctcgtgcgccgccatctccggcgccgcgtcgcgccgcgccgtcgcgcgcccccaggcgtcc
                 ############################################################################################################################################################################################################################################################################################################


                       5410      5420      5430      5440      5450      5460      5470      5480      5490      5500      5510      5520      5530      5540      5550      5560      5570      5580      5590      5600      5610      5620      5630      5640      5650      5660      5670      5680      5690      5700
                 =========+=========+=========+=========+=========+=========+=========+=========+=========+=========+=========+=========+=========+=========+=========+=========+=========+=========+=========+=========+=========+=========+=========+=========+=========+=========+=========+=========+=========+=========+
PRV-WT complete  ccggccagcaccgcgaaggcctggcgccggtgcgccagctccacgcggtacaccggcgccggggggcgcgcgtccggcaggccgagcagggcgcccagcgccgtgcgcgcgcgcccgccgcccggcgcggcggccagctccagcaggcggcgcaggacggtgccgtcggcgcccggctgctgctgctgcgagtgcgcctgctcctcgtcgggggcgcgcacccagccgtggggcgcgaggcgctcctcgacggcggtcacgtggcgcgcgaggccggcggccgcgtcgcagagcccgcgc
PRV-delete UL10  ccggccagcaccgcgaaggcctggcgccggtgcgccagctccacgcggtacaccggcgccggggggcgcgcgtccggcaggccgagcagggcgcccagcgccgtgcgcgcgcgcccgccgcccggcgcggcggccagctccagcaggcggcgcaggacggtgccgtcggcgcccggctgctgctgctgcgagtgcgcctgctcctcgtcgggggcgcgcacccagccgtggggcgcgaggcgctcctcgacggcggtcacgtggcgcgcgaggccggcggccgcgtcgcagagcccgcgc
                 ############################################################################################################################################################################################################################################################################################################


                       5710      5720      5730      5740      5750      5760      5770      5780      5790      5800      5810      5820      5830      5840      5850      5860      5870      5880      5890      5900      5910      5920      5930      5940      5950      5960      5970      5980      5990      6000
                 =========+=========+=========+=========+=========+=========+=========+=========+=========+=========+=========+=========+=========+=========+=========+=========+=========+=========+=========+=========+=========+=========+=========+=========+=========+=========+=========+=========+=========+=========+
PRV-WT complete  ggcgcgtcggccgcgagcgcggcgtacgtgcggtcctccacgtagccggcgcccacggccggggcgaggcgcgcgacgctcggcgtcacgttctgggcgacgtagtcgcggatgttgagctgcgcccgcacgtggcggaagaaggtggcggccgcgcgctccccgaggggcgagcggcccacgggcgccgcggggtccgtgacgttgacggcgtccaggtgctcgcggagccgcgcgcggttgaagctctcaaagtgggccaggtagacgtacgtcacgaactcgcggtcggggagcttg
PRV-delete UL10  ggcgcgtcggccgcgagcgcggcgtacgtgcggtcctccacgtagccggcgcccacggccggggcgaggcgcgcgacgctcggcgtcacgttctgggcgacgtagtcgcggatgttgagctgcgcccgcacgtggcggaagaaggtggcggccgcgcgctccccgaggggcgagcggcccacgggcgccgcggggtccgtgacgttgacggcgtccaggtgctcgcggagccgcgcgcggttgaagctctcaaagtgggccaggtagacgtacgtcacgaactcgcggtcggggagcttg
                 ############################################################################################################################################################################################################################################################################################################


                       6010      6020      6030      6040      6050      6060      6070      6080      6090      6100      6110      6120      6130      6140      6150      6160      6170      6180      6190      6200      6210      6220      6230      6240      6250      6260      6270      6280      6290      6300
                 =========+=========+=========+=========+=========+=========+=========+=========+=========+=========+=========+=========+=========+=========+=========+=========+=========+=========+=========+=========+=========+=========+=========+=========+=========+=========+=========+=========+=========+=========+
PRV-WT complete  aggctgccgcggtcgtgggcgatgtagtcccggaggacgcccatctcggcgcggtcggcgcgcacgcgctcgtcgacgtagcgcgcggcgcgcgccgccgcgggcccccgcgcgtagccgctcaggcagcagaagcgcgagagcgccgcgaacgagagcagccccgcggggtccaggccgctggggttgggccccacgcgcgggttgtacgtgagcagcaggtccttgagggcccgcaggtcgtaggccggccgcgcgtggaagaggtggaggcgcgtggccagcaccagcgtcttctcc
PRV-delete UL10  aggctgccgcggtcgtgggcgatgtagtcccggaggacgcccatctcggcgcggtcggcgcgcacgcgctcgtcgacgtagcgcgcggcgcgcgccgccgcgggcccccgcgcgtagccgctcaggcagcagaagcgcgagagcgccgcgaacgagagcagccccgcggggtccaggccgctggggttgggccccacgcgcgggttgtacgtgagcagcaggtccttgagggcccgcaggtcgtaggccggccgcgcgtggaagaggtggaggcgcgtggccagcaccagcgtcttctcc
                 ############################################################################################################################################################################################################################################################################################################


                       6310      6320      6330      6340      6350      6360      6370      6380      6390      6400      6410      6420      6430      6440      6450      6460      6470      6480      6490      6500      6510      6520      6530      6540      6550      6560      6570      6580      6590      6600
                 =========+=========+=========+=========+=========+=========+=========+=========+=========+=========+=========+=========+=========+=========+=========+=========+=========+=========+=========+=========+=========+=========+=========+=========+=========+=========+=========+=========+=========+=========+
PRV-WT complete  tcgggcccgaagcgcgccacgtaccagaagggcgtgttgtggttcccgtagaggcgccggaaggcggccagcgtctgggtctcgtggtgcacaaagagcgaggtcagcccgcggcgcccgaccgcgatggagcgcagcgggcgctccggcgcgtactcggcgttgccggtgcgcgggcgcatctgctcgaggaccatggtcagggccgcgatgaggccgtcgtggagcgcgaaggtggccgcctcgtccatggcctcgaggacgtcccgcgccgcgaggggctccccgcggcagagcgcg
PRV-delete UL10  tcgggcccgaagcgcgccacgtaccagaagggcgtgttgtggttcccgtagaggcgccggaaggcggccagcgtctgggtctcgtggtgcacaaagagcgaggtcagcccgcggcgcccgaccgcgatggagcgcagcgggcgctccggcgcgtactcggcgttgccggtgcgcgggcgcatctgctcgaggaccatggtcagggccgcgatgaggccgtcgtggagcgcgaaggtggccgcctcgtccatggcctcgaggacgtcccgcgccgcgaggggctccccgcggcagagcgcg
                 ############################################################################################################################################################################################################################################################################################################


                       6610      6620      6630      6640      6650      6660      6670      6680      6690      6700      6710      6720      6730      6740      6750      6760      6770      6780      6790      6800      6810      6820      6830      6840      6850      6860      6870      6880      6890      6900
                 =========+=========+=========+=========+=========+=========+=========+=========+=========+=========+=========+=========+=========+=========+=========+=========+=========+=========+=========+=========+=========+=========+=========+=========+=========+=========+=========+=========+=========+=========+
PRV-WT complete  cggacgaccgccggggtcgcggcggcgacgaacacgggccgcacgcggacgctggccacgtgcccggtgaggcagaacgtgacgccggggcggcgcgctaaaagctcggcctcggcgcgctcgcccggcagtgagaaggcctcgtccagcccggcgccgtcccacgcgtacgagacgacgtagaccgcccccgagggctcctggcccgtgagcagcatcagcgagtacgtgatcgcgcagccgtccgtggcgtacaggacgcggatcatgctgggcggcatcttctccgggtggtgcggg
PRV-delete UL10  cggacgaccgccggggtcgcggcggcgacgaacacgggccgcacgcggacgctggccacgtgcccggtgaggcagaacgtgacgccggggcggcgcgctaaaagctcggcctcggcgcgctcgcccggcagtgagaaggcctcgtccagcccggcgccgtcccacgcgtacgagacgacgtagaccgcccccgagggctcctggcccgtgagcagcatcagcgagtacgtgatcgcgcagccgtccgtggcgtacaggacgcggatcatgctgggcggcatcttctccgggtggtgcggg
                 ############################################################################################################################################################################################################################################################################################################


                       6910      6920      6930      6940      6950      6960      6970      6980      6990      7000      7010      7020      7030      7040      7050      7060      7070      7080      7090      7100      7110      7120      7130      7140      7150      7160      7170      7180      7190      7200
                 =========+=========+=========+=========+=========+=========+=========+=========+=========+=========+=========+=========+=========+=========+=========+=========+=========+=========+=========+=========+=========+=========+=========+=========+=========+=========+=========+=========+=========+=========+
PRV-WT complete  cgccgcggccgtgggagggcttatgagccgccggacgaactccgcctccgggagtcgctggccgtgctcaacgtgatcctccccgccccgctcgccgccgaggacgtgatggcctcggccgaggccgcgcgccgcctggcccgcgcggacacgctggcgcgcacctaccaggcctgccagcgcaacctcgagtgcctggcgcgccacgaggcctccggcgacgacggcctggacgccgtggtcgcggcgcacgcggccaacgcgcggcggctcgcggacacgtgcctggcggcgctcatg
PRV-delete UL10  cgccgcggccgtgggagggcttatgagccgccggacgaactccgcctccgggagtcgctggccgtgctcaacgtgatcctccccgccccgctcgccgccgaggacgtgatggcctcggccgaggccgcgcgccgcctggcccgcgcggacacgctggcgcgcacctaccaggcctgccagcgcaacctcgagtgcctggcgcgccacgaggcctccggcgacgacggcctggacgccgtggtcgcggcgcacgcggccaacgcgcggcggctcgcggacacgtgcctggcggcgctcatg
                 ############################################################################################################################################################################################################################################################################################################


                       7210      7220      7230      7240      7250      7260      7270      7280      7290      7300      7310      7320      7330      7340      7350      7360      7370      7380      7390      7400      7410      7420      7430      7440      7450      7460      7470      7480      7490      7500
                 =========+=========+=========+=========+=========+=========+=========+=========+=========+=========+=========+=========+=========+=========+=========+=========+=========+=========+=========+=========+=========+=========+=========+=========+=========+=========+=========+=========+=========+=========+
PRV-WT complete  cacctgtacctctcggtcggcgccgtcgacgcggacacggacgacctcgtcgagcaggccctgcgcatgaccgccgagagcaacgtggtgatgtccgacgtggccgtcctggagcggacgctgggactggcccggcggcggcagccggcgcccgtccgggccgcgcccgccggggtgggcctgcccgcccccgtcccgcccgccccgcacgtcgccgccccgccggccccgcagaccccgcccgaggaggaggcggatgcggaggcggaggaagacgaggctggcggggaccgccccgag
PRV-delete UL10  cacctgtacctctcggtcggcgccgtcgacgcggacacggacgacctcgtcgagcaggccctgcgcatgaccgccgagagcaacgtggtgatgtccgacgtggccgtcctggagcggacgctgggactggcccggcggcggcagccggcgcccgtccgggccgcgcccgccggggtgggcctgcccgcccccgtcccgcccgccccgcacgtcgccgccccgccggccccgcagaccccgcccgaggaggaggcggatgcggaggcggaggaagacgaggctggcggggaccgccccgag
                 ############################################################################################################################################################################################################################################################################################################


                       7510      7520      7530      7540      7550      7560      7570      7580      7590      7600      7610      7620      7630      7640      7650      7660      7670      7680      7690      7700      7710      7720      7730      7740      7750      7760      7770      7780      7790      7800
                 =========+=========+=========+=========+=========+=========+=========+=========+=========+=========+=========+=========+=========+=========+=========+=========+=========+=========+=========+=========+=========+=========+=========+=========+=========+=========+=========+=========+=========+=========+
PRV-WT complete  gaggacgcggtcccgctgctgcccccgcgcgcccccgtcaagacgcgcccgcccgagaagaagaaggtccggaggctggccgaggcctgcaagatgtagacggagagcggggggagagagagggcacgcgcgaagagaagagacgaggggcgaccgagaagaaggggacggagaccaccgggtgtttttttttcttttcaataaacatctttattgaaaaaaaagagaagcgtcctgcgtgtgttgtgtgcgcgcgcccgggtgccatcgccatcgtcccggcgcgggccaccgcggcct
PRV-delete UL10  gaggacgcggtcccgctgctgcccccgcgcgcccccgtcaagacgcgcccgcccgagaagaagaaggtccggaggctggccgaggcctgcaagatgtagacggagagcggggggagagagagggcacgcgcgaagagaagagacgaggggcgaccgagaagaaggggacggagaccaccgggtgtttttttttcttttcaataaacatctttattgaaaaaaaagagaagcgtcctgcgtgtgttgtgtgcgcgcgcccgggtgccatcgccatcgtcccggcgcgggccaccgcggcct
                 ############################################################################################################################################################################################################################################################################################################


                       7810      7820      7830      7840      7850      7860      7870      7880      7890      7900      7910      7920      7930      7940      7950      7960      7970      7980      7990      8000      8010      8020      8030      8040      8050      8060      8070      8080      8090      8100
                 =========+=========+=========+=========+=========+=========+=========+=========+=========+=========+=========+=========+=========+=========+=========+=========+=========+=========+=========+=========+=========+=========+=========+=========+=========+=========+=========+=========+=========+=========+
PRV-WT complete  cacaggcccgtggagccgaagccgcgggtcccgcgcgcgctgctcggggcgacaaagtcccgcgtgaagagccacgcggggcggtgctgcatcggggtgcgcggggtggcggggaacggggaccgccccgtgatccagctcagcggctcgcgcgtcagcaccagctgcgccacgcgctggcccgcctccagggtcaccgggtgcgcgccgcggttctggatccggaagcggcacgggccgctctcccagggcgtggggaagacgacgatgccgcggaggttgagggacgagcgcccgaag
PRV-delete UL10  cacaggcccgtggagccgaagccgcgggtcccgcgcgcgctgctcggggcgacaaagtcccgcgtgaagagccacgcggggcggtgctgcatcggggtgcgcggggtggcggggaacggggaccgccccgtgatccagctcagcggctcgcgcgtcagcaccagctgcgccacgcgctggcccgcctccagggtcaccgggtgcgcgccgcggttctggatccggaagcggcacgggccgctctcccagggcgtggggaagacgacgatgccgcggaggttgagggacgagcgcccgaag
                 ############################################################################################################################################################################################################################################################################################################


                       8110      8120      8130      8140      8150      8160      8170      8180      8190      8200      8210      8220      8230      8240      8250      8260      8270      8280      8290      8300      8310      8320      8330      8340      8350      8360      8370      8380      8390      8400
                 =========+=========+=========+=========+=========+=========+=========+=========+=========+=========+=========+=========+=========+=========+=========+=========+=========+=========+=========+=========+=========+=========+=========+=========+=========+=========+=========+=========+=========+=========+
PRV-WT complete  acgtaggcccagtggcggccgtccgtgcggtgcaccggcagcgccacggtctccgcgccgccggggggcagcaccagctcgcgcgggcaggggatgtcgtatccggcgtcctcgtcgcgcttcggcgcaaagacctcgaagaagggcacgtccgtggcgatgcccggcgcgaggcgcagcggcgtgcaataaaactgcgcccgtgggcggcccggggctacaatcgcctgcacctcgccgcgaaagcccgcgtccacgatcccgttggtggtgcgaccgcccccgcgctgcgcgacgagc
PRV-delete UL10  acgtaggcccagtggcggccgtccgtgcggtgcaccggcagcgccacggtctccgcgccgccggggggcagcaccagctcgcgcgggcaggggatgtcgtatccggcgtcctcgtcgcgcttcggcgcaaagacctcgaagaagggcacgtccgtggcgatgcccggcgcgaggcgcagcggcgtgcaataaaactgcgcccgtgggcggcccggggctacaatcgcctgcacctcgccgcgaaagcccgcgtccacgatcccgttggtggtgcgaccgcccccgcgctgcgcgacgagc
                 ############################################################################################################################################################################################################################################################################################################


                       8410      8420      8430      8440      8450      8460      8470      8480      8490      8500      8510      8520      8530      8540      8550      8560      8570      8580      8590      8600      8610      8620      8630      8640      8650      8660      8670      8680      8690      8700
                 =========+=========+=========+=========+=========+=========+=========+=========+=========+=========+=========+=========+=========+=========+=========+=========+=========+=========+=========+=========+=========+=========+=========+=========+=========+=========+=========+=========+=========+=========+
PRV-WT complete  atcgcgtatccggtgggcagcgccaccttgaggcccaggggaaccttataaaatccctccggccccggactccggcacaccagcagccgccctccgtccactgtcacgggctcgctcgcagacaccaggatggtctcttcctccgcccggctctcgctgacgctcgtcgccgcgctctgcgcgctggtgacgcccgcgctctcttccatcgtcttttccgaggggtcgctgccgctgctgcgcgaggagtcgcggaccagcttctggagcgccacctgcgccgccaggggagtcccggtg
PRV-delete UL10  atcgcgtatccggtgggcagcgccaccttgaggcccaggggaaccttataaaatccctccggccccggactccggcacaccagcagccgccctccgtccactgtcacgggctcgctcgcagacaccaggatggtctcttcctccgcccggctctcgctgacgctcgtcgccgcgctctgcgcgctggtgacgcccgcgctctcttccatcgtcttttccgaggggtcgctgccgctgctgcgcgaggagtcgcggaccagcttctggagcgccacctgcgccgccaggggagtcccggtg
                 ############################################################################################################################################################################################################################################################################################################


                       8710      8720      8730      8740      8750      8760      8770      8780      8790      8800      8810      8820      8830      8840      8850      8860      8870      8880      8890      8900      8910      8920      8930      8940      8950      8960      8970      8980      8990      9000
                 =========+=========+=========+=========+=========+=========+=========+=========+=========+=========+=========+=========+=========+=========+=========+=========+=========+=========+=========+=========+=========+=========+=========+=========+=========+=========+=========+=========+=========+=========+
PRV-WT complete  gatcagccgacggccgccgccgtgacattttatatctgcctcctggcggtgctcgtcgtcgccctgggttacgccacccggacgtgcgcccggatgctgcacacctcccccgcggggaggcgcgtataaagatcgatccgcacgcgcccgatcccactcgctcgccatgtccagctcgagaaagacccgggtcgccgccgacgagaccgcctcgggggcgcgccgccgcgccggcagcgcctctcgcgcccgggccgccgccccggccgtcccgaccgccgcgacccccagacgcccctc
PRV-delete UL10  gatcagccgacggccgccgccgtgacattttatatctgcctcctggcggtgctcgtcgtcgccctgggttacgccacccggacgtgcgcccggatgctgcacacctcccccgcggggaggcgcgtataaagatcgatccgcacgcgcccgatcccactcgctcgccatgtccagctcgagaaagacccgggtcgccgccgacgagaccgcctcgggggcgcgccgccgcgccggcagcgcctctcgcgcccgggccgccgccccggccgtcccgaccgccgcgacccccagacgcccctc
                 ############################################################################################################################################################################################################################################################################################################


                       9010      9020      9030      9040      9050      9060      9070      9080      9090      9100      9110      9120      9130      9140      9150      9160      9170      9180      9190      9200      9210      9220      9230      9240      9250      9260      9270      9280      9290      9300
                 =========+=========+=========+=========+=========+=========+=========+=========+=========+=========+=========+=========+=========+=========+=========+=========+=========+=========+=========+=========+=========+=========+=========+=========+=========+=========+=========+=========+=========+=========+
PRV-WT complete  ggcctacgacgacggcttctcctaccggtctgccccgtcctacgacgacgatgactactacggctacgatggccacggctcctcccacgccccccgcgccgccaaggtgacgcccgcggccgcctcgcgggcctcgaccggggccaagagcgcctcggccgccaagacccccgcggccaagaccgcccgctcggcctcggccgccgccccggccgccgccgccaccaccgccgccgccgcggaaccggccgcccggcgcgcctcgacgcgggccgcccccggggagaacctcgacgtggg
PRV-delete UL10  ggcctacgacgacggcttctcctaccggtctgccccgtcctacgacgacgatgactactacggctacgatggccacggctcctcccacgccccccgcgccgccaaggtgacgcccgcggccgcctcgcgggcctcgaccggggccaagagcgcctcggccgccaagacccccgcggccaagaccgcccgctcggcctcggccgccgccccggccgccgccgccaccaccgccgccgccgcggaaccggccgcccggcgcgcctcgacgcgggccgcccccggggagaacctcgacgtggg
                 ############################################################################################################################################################################################################################################################################################################


                       9310      9320      9330      9340      9350      9360      9370      9380      9390      9400      9410      9420      9430      9440      9450      9460      9470      9480      9490      9500      9510      9520      9530      9540      9550      9560      9570      9580      9590      9600
                 =========+=========+=========+=========+=========+=========+=========+=========+=========+=========+=========+=========+=========+=========+=========+=========+=========+=========+=========+=========+=========+=========+=========+=========+=========+=========+=========+=========+=========+=========+
PRV-WT complete  ccgccgccggctcgccttcagcgacaggccgtgcgaggccaacgtgccctggcgcggcgcgacgcacgccttcaacaagcgcatcttctgcgcggccgtggggcgcgtggccgaggcccacgcccgcgccgccgccgagtccctctgggacatgaaccccccgacgacggacgcggcgctcgaccgcttcctgcaggccgcggtggtgcgcatcaccgtgtgcgaggggctggacctgatcgaggcggccaacgccgtcctggacgagagcaccccggggcggaagggaaaagtgtataa
PRV-delete UL10  ccgccgccggctcgccttcagcgacaggccgtgcgaggccaacgtgccctggcgcggcgcgacgcacgccttcaacaagcgcatcttctgcgcggccgtggggcgcgtggccgaggcccacgcccgcgccgccgccgagtccctctgggacatgaaccccccgacgacggacgcggcgctcgaccgcttcctgcaggccgcggtggtgcgcatcaccgtgtgcgaggggctggacctgatcgaggcggccaacgccgtcctggacgagagcaccccggggcggaagggaaaagtgtataa
                 ############################################################################################################################################################################################################################################################################################################


                       9610      9620      9630      9640      9650      9660      9670      9680      9690      9700      9710      9720      9730      9740      9750      9760      9770      9780      9790      9800      9810      9820      9830      9840      9850      9860      9870      9880      9890      9900
                 =========+=========+=========+=========+=========+=========+=========+=========+=========+=========+=========+=========+=========+=========+=========+=========+=========+=========+=========+=========+=========+=========+=========+=========+=========+=========+=========+=========+=========+=========+
PRV-WT complete  ataaaggcgttgcaaatggcaccccgctcagtcgtccgctgtcttgcttgtgggggggtcggtgaggatgcgcgacgaggagtgcgtggtcgcgttcgacgaggccctgctggggggcgcggccccgggccggccgcccgtggtggccgtcccgcgggcggccagcccggacgcgctgtaccagcgcctcatcagcgacctggccttcgacgagggcccggcgctgctgagcgccatggagcgctggaacgaggacctgttctcgtgcctgcccggcaacgaggacctgtacgcgcgcat
PRV-delete UL10  ataaaggcgttgcaaatggcaccccgctcagtcgtccgctgtcttgcttgtgggggggtcggtgaggatgcgcgacgaggagtgcgtggtcgcgttcgacgaggccctgctggggggcgcggccccgggccggccgcccgtggtggccgtcccgcgggcggccagcccggacgcgctgtaccagcgcctcatcagcgacctggccttcgacgagggcccggcgctgctgagcgccatggagcgctggaacgaggacctgttctcgtgcctgcccggcaacgaggacctgtacgcgcgcat
                 ############################################################################################################################################################################################################################################################################################################


                       9910      9920      9930      9940      9950      9960      9970      9980      9990     10000     10010     10020     10030     10040     10050     10060     10070     10080     10090     10100     10110     10120     10130     10140     10150     10160     10170     10180     10190     10200
                 =========+=========+=========+=========+=========+=========+=========+=========+=========+=========+=========+=========+=========+=========+=========+=========+=========+=========+=========+=========+=========+=========+=========+=========+=========+=========+=========+=========+=========+=========+
PRV-WT complete  ggtcatgctctcggcctccgcggacgaggtggcggcccaggtccaggcgccgacggcggacgcgagcgtcgacctcggggtgcccggggcggagcccatgccgcgggtcccggccgtggaggaggagctgcccgcgtacgtggcggccgtggagcgcttcttcgtgtccacgctgcgggcgcgcgaggagcggtacgcgcgcctgctggacgcctactgccgcgcggtgctgcgctacctgcacgcgcaggcgcgccgcgacgagggccggctccggcagctggtgcgcgggcgctacta
PRV-delete UL10  ggtcatgctctcggcctccgcggacgaggtggcggcccaggtccaggcgccgacggcggacgcgagcgtcgacctcggggtgcccggggcggagcccatgccgcgggtcccggccgtggaggaggagctgcccgcgtacgtggcggccgtggagcgcttcttcgtgtccacgctgcgggcgcgcgaggagcggtacgcgcgcctgctggacgcctactgccgcgcggtgctgcgctacctgcacgcgcaggcgcgccgcgacgagggccggctccggcagctggtgcgcgggcgctacta
                 ############################################################################################################################################################################################################################################################################################################


                      10210     10220     10230     10240     10250     10260     10270     10280     10290     10300     10310     10320     10330     10340     10350     10360     10370     10380     10390     10400     10410     10420     10430     10440     10450     10460     10470     10480     10490     10500
                 =========+=========+=========+=========+=========+=========+=========+=========+=========+=========+=========+=========+=========+=========+=========+=========+=========+=========+=========+=========+=========+=========+=========+=========+=========+=========+=========+=========+=========+=========+
PRV-WT complete  ccgcgacgtcgcgcgcctggcgcgcctgctctttctgcacctgtacgtggccacggcgcgcgagctctcgtggcgcctgcacgcggaccaggtggtggcccaggacgtgttcatgtccctgcgctacgagtgggaccagacgcggcagctgacgtgcctcttccacccggtgctgttcaaccacggcgtggtgctgctggcgggcgcgccggtgccggccgcgcagctgcgcgccgtcaaccgccgccggcgcgagctgggcctgccgctcgtgcgcgccggcctcatcgaggaggacgg
PRV-delete UL10  ccgcgacgtcgcgcgcctggcgcgcctgctctttctgcacctgtacgtggccacggcgcgcgagctctcgtggcgcctgcacgcggaccaggtggtggcccaggacgtgttcatgtccctgcgctacgagtgggaccagacgcggcagctgacgtgcctcttccacccggtgctgttcaaccacggcgtggtgctgctggcgggcgcgccggtgccggccgcgcagctgcgcgccgtcaaccgccgccggcgcgagctgggcctgccgctcgtgcgcgccggcctcatcgaggaggacgg
                 ############################################################################################################################################################################################################################################################################################################


                      10510     10520     10530     10540     10550     10560     10570     10580     10590     10600     10610     10620     10630     10640     10650     10660     10670     10680     10690     10700     10710     10720     10730     10740     10750     10760     10770     10780     10790     10800
                 =========+=========+=========+=========+=========+=========+=========+=========+=========+=========+=========+=========+=========+=========+=========+=========+=========+=========+=========+=========+=========+=========+=========+=========+=========+=========+=========+=========+=========+=========+
PRV-WT complete  cgccgacctggtcgaggagccgccgttctccgccgcgctgccgcgcgccgccggctacctctcgcaccaggtgcggatcaagatggaggcctactcgcgcgagtaccgcgaccacacgtactgccgcccgccctcgcccgtggccagctacggcagcaccgcggaggccctgctgccgcccccgtcgccctcggccgtgctgccctgcgacccgacgccgccgccgcgcgtctcggccgccccgctcatcaccacggtgacgctggccgaggccgaggaggacggcgcgctgacgacggc
PRV-delete UL10  cgccgacctggtcgaggagccgccgttctccgccgcgctgccgcgcgccgccggctacctctcgcaccaggtgcggatcaagatggaggcctactcgcgcgagtaccgcgaccacacgtactgccgcccgccctcgcccgtggccagctacggcagcaccgcggaggccctgctgccgcccccgtcgccctcggccgtgctgccctgcgacccgacgccgccgccgcgcgtctcggccgccccgctcatcaccacggtgacgctggccgaggccgaggaggacggcgcgctgacgacggc
                 ############################################################################################################################################################################################################################################################################################################


                      10810     10820     10830     10840     10850     10860     10870     10880     10890     10900     10910     10920     10930     10940     10950     10960     10970     10980     10990     11000     11010     11020     11030     11040     11050     11060     11070     11080     11090     11100
                 =========+=========+=========+=========+=========+=========+=========+=========+=========+=========+=========+=========+=========+=========+=========+=========+=========+=========+=========+=========+=========+=========+=========+=========+=========+=========+=========+=========+=========+=========+
PRV-WT complete  ggcgcccgcggccgcggtcgcgaccgcgacggtcgcctcgcccgggcccgccacccccgcctaccacctcatcccccgcgacgcgctcaaccgcatgtttgagatgtgacgccgcgccccgcgcgcgcggtcggatctgcggcgcccgaccacctgtgggcggggcgtataaagccgcgggccggcccggagggggggcatttgccgcagcgaccaccatgagcgacgcggggcccgtgcggcgcccgaggcgctcgcaagagcagcgcttccagccgttcccccgcgcggggctcctgg
PRV-delete UL10  ggcgcccgcggccgcggtcgcgaccgcgacggtcgcctcgcccgggcccgccacccccgcctaccacctcatcccccgcgacgcgctcaaccgcatgtttgagatgtgacgccgcgccccgcgcgcgcggtcggatctgcggcgcccgaccacctgtgggcggggcgtataaagccgcgggccggcccggagggggggcatttgccgcagcgaccaccatgagcgacgcggggcccgtgcggcgcccgaggcgctcgcaagagcagcgcttccagccgttcccccgcgcggggctcctgg
                 ############################################################################################################################################################################################################################################################################################################


                      11110     11120     11130     11140     11150     11160     11170     11180     11190     11200     11210     11220     11230     11240     11250     11260     11270     11280     11290     11300     11310     11320     11330     11340     11350     11360     11370     11380     11390     11400
                 =========+=========+=========+=========+=========+=========+=========+=========+=========+=========+=========+=========+=========+=========+=========+=========+=========+=========+=========+=========+=========+=========+=========+=========+=========+=========+=========+=========+=========+=========+
PRV-WT complete  tgcacctgcaggacgtgctcgtgggcgaggtgcggcgcccggacttccgccctccgcccgacgaggagagcagcgaggaggaggagcccgtctggaccgacgacgaggaggaggacgagggggaggaggggggagcccggcgcggggacggggagggggacgacgacgtggacgtggacgacgaggaggaggagagcgaggggggcgcgtggtccgacggggagctgtacgtggcggccgacggcgacgcgtgggacgcggaagaggacgaggacggggacgaggatgaggatgatgact
PRV-delete UL10  tgcacctgcaggacgtgctcgtgggcgaggtgcggcgcccggacttccgccctccgcccgacgaggagagcagcgaggaggaggagcccgtctggaccgacgacgaggaggaggacgagggggaggaggggggagcccggcgcggggacggggagggggacgacgacgtggacgtggacgacgaggaggaggagagcgaggggggcgcgtggtccgacggggagctgtacgtggcggccgacggcgacgcgtgggacgcggaagaggacgaggacggggacgaggatgaggatgatgact
                 ############################################################################################################################################################################################################################################################################################################


                      11410     11420     11430     11440     11450     11460     11470     11480     11490     11500     11510     11520     11530     11540     11550     11560     11570     11580     11590     11600     11610     11620     11630     11640     11650     11660     11670     11680     11690     11700
                 =========+=========+=========+=========+=========+=========+=========+=========+=========+=========+=========+=========+=========+=========+=========+=========+=========+=========+=========+=========+=========+=========+=========+=========+=========+=========+=========+=========+=========+=========+
PRV-WT complete  atgacgatgacgacgacggctatgacggccgcggcgccgcggcctcccgcgccgcggtcgggccccccgccgacgtggactacctgtcgcgcgccctccgcggcatggagacggcctccggccccgacgagcgggaggccttcacccgctacgccggcacgctctaccgccgccagttccagcccgggcggccggggtaccggcccccgcggccgcgcgacccgccgcctcccccgcccccgcgggcgcggccgccgctgacgacggcgacgacggccgcgcccgccgtcggcaccgccg
PRV-delete UL10  atgacgatgacgacgacggctatgacggccgcggcgccgcggcctcccgcgccgcggtcgggccccccgccgacgtggactacctgtcgcgcgccctccgcggcatggagacggcctccggccccgacgagcgggaggccttcacccgctacgccggcacgctctaccgccgccagttccagcccgggcggccggggtaccggcccccgcggccgcgcgacccgccgcctcccccgcccccgcgggcgcggccgccgctgacgacggcgacgacggccgcgcccgccgtcggcaccgccg
                 ############################################################################################################################################################################################################################################################################################################


                      11710     11720     11730     11740     11750     11760     11770     11780     11790     11800     11810     11820     11830     11840     11850     11860     11870     11880     11890     11900     11910     11920     11930     11940     11950     11960     11970     11980     11990     12000
                 =========+=========+=========+=========+=========+=========+=========+=========+=========+=========+=========+=========+=========+=========+=========+=========+=========+=========+=========+=========+=========+=========+=========+=========+=========+=========+=========+=========+=========+=========+
PRV-WT complete  ccccctgcggcggcgacgactccgcgccgcgcctcgagcgggacccggacgccgcgtacgccagctggacgcgcgacatgcccgacgggacctttctggccatgccgtcgtgggtggcgctgcgccgcgagccccgcgggcccccgggccgccccgtcgggcccccggacatcctgcgccgcgccccgcgggccctgaccttcacgccgcaggcggcctcggcggcgctcgtgagccgcgaccaggacgcctgggaggcgctggtgccgctgcaccacctgcagatgcactcgtggggcg
PRV-delete UL10  ccccctgcggcggcgacgactccgcgccgcgcctcgagcgggacccggacgccgcgtacgccagctggacgcgcgacatgcccgacgggacctttctggccatgccgtcgtgggtggcgctgcgccgcgagccccgcgggcccccgggccgccccgtcgggcccccggacatcctgcgccgcgccccgcgggccctgaccttcacgccgcaggcggcctcggcggcgctcgtgagccgcgaccaggacgcctgggaggcgctggtgccgctgcaccacctgcagatgcactcgtggggcg
                 ############################################################################################################################################################################################################################################################################################################


                      12010     12020     12030     12040     12050     12060     12070     12080     12090     12100     12110     12120     12130     12140     12150     12160     12170     12180     12190     12200     12210     12220     12230     12240     12250     12260     12270     12280     12290     12300
                 =========+=========+=========+=========+=========+=========+=========+=========+=========+=========+=========+=========+=========+=========+=========+=========+=========+=========+=========+=========+=========+=========+=========+=========+=========+=========+=========+=========+=========+=========+
PRV-WT complete  gcagcgcgccgccctcgcgcgggggcgtgtacgcgacgcccgcgcccgagacgcgcggcgtgtggcgccgcgcgctgcggcaggccatcgccctgcaccacgccatgttcctggcgccgctgcagtcctcgacggtcggcgcgcccgcgctcgcgggcgaggcgctggccttcgcgctggacgccgcggcgcgcgcggccgtcaaccacgacgccgcggcgcgcgagctccccgagcgcgcggcgcgcgcgctgcgcgccgtcggccggcgcgcgcccgcggcctcggggccgcggcccg
PRV-delete UL10  gcagcgcgccgccctcgcgcgggggcgtgtacgcgacgcccgcgcccgagacgcgcggcgtgtggcgccgcgcgctgcggcaggccatcgccctgcaccacgccatgttcctggcgccgctgcagtcctcgacggtcggcgcgcccgcgctcgcgggcgaggcgctggccttcgcgctggacgccgcggcgcgcgcggccgtcaaccacgacgccgcggcgcgcgagctccccgagcgcgcggcgcgcgcgctgcgcgccgtcggccggcgcgcgcccgcggcctcggggccgcggcccg
                 ############################################################################################################################################################################################################################################################################################################


                      12310     12320     12330     12340     12350     12360     12370     12380     12390     12400     12410     12420     12430     12440     12450     12460     12470     12480     12490     12500     12510     12520     12530     12540     12550     12560     12570     12580     12590     12600
                 =========+=========+=========+=========+=========+=========+=========+=========+=========+=========+=========+=========+=========+=========+=========+=========+=========+=========+=========+=========+=========+=========+=========+=========+=========+=========+=========+=========+=========+=========+
PRV-WT complete  gcctcttccgcgccttctgcgggtccctggcctactggccggagctgcgcgtcctcagcgggcaccccgaggacgtccgcttcgcggccttccacctggcggtggccgaggtgtacctgctggcgcgcgcccacggccagaacccgggcttcacggccgaggagcgcgagctgctcgcgcccatgttcacgctgaccgtgctcgcggtgcaccacgcgctgcgctggctcacgacggccgtggcgcgcgccgtctcggacatccccgacgacgaggccttccgcgccgtgcgctcgcgcg
PRV-delete UL10  gcctcttccgcgccttctgcgggtccctggcctactggccggagctgcgcgtcctcagcgggcaccccgaggacgtccgcttcgcggccttccacctggcggtggccgaggtgtacctgctggcgcgcgcccacggccagaacccgggcttcacggccgaggagcgcgagctgctcgcgcccatgttcacgctgaccgtgctcgcggtgcaccacgcgctgcgctggctcacgacggccgtggcgcgcgccgtctcggacatccccgacgacgaggccttccgcgccgtgcgctcgcgcg
                 ############################################################################################################################################################################################################################################################################################################


                      12610     12620     12630     12640     12650     12660     12670     12680     12690     12700     12710     12720     12730     12740     12750     12760     12770     12780     12790     12800     12810     12820     12830     12840     12850     12860     12870     12880     12890     12900
                 =========+=========+=========+=========+=========+=========+=========+=========+=========+=========+=========+=========+=========+=========+=========+=========+=========+=========+=========+=========+=========+=========+=========+=========+=========+=========+=========+=========+=========+=========+
PRV-WT complete  tgccggcgtcgctggtgccgctgggcagcatcgcgctctcggacgccgagtacgccgtgctgaacgcgaccgaggtggcggcggcgcgcgacgccacgggcctgggccaggccgtgtcgctgggctacgccgccgcgcgctccgcgctcacgggcctcatgcgggcccacgccggcgggagcgacgccctcgtcgccttcgcgctggtgctgcagcgcctggcgggccacgcgaacctgctgctcaactgcctgctcggcgccgccgtccacggcggccggccggtgcgcgtgtacgagg
PRV-delete UL10  tgccggcgtcgctggtgccgctgggcagcatcgcgctctcggacgccgagtacgccgtgctgaacgcgaccgaggtggcggcggcgcgcgacgccacgggcctgggccaggccgtgtcgctgggctacgccgccgcgcgctccgcgctcacgggcctcatgcgggcccacgccggcgggagcgacgccctcgtcgccttcgcgctggtgctgcagcgcctggcgggccacgcgaacctgctgctcaactgcctgctcggcgccgccgtccacggcggccggccggtgcgcgtgtacgagg
                 ############################################################################################################################################################################################################################################################################################################


                      12910     12920     12930     12940     12950     12960     12970     12980     12990     13000     13010     13020     13030     13040     13050     13060     13070     13080     13090     13100     13110     13120     13130     13140     13150     13160     13170     13180     13190     13200
                 =========+=========+=========+=========+=========+=========+=========+=========+=========+=========+=========+=========+=========+=========+=========+=========+=========+=========+=========+=========+=========+=========+=========+=========+=========+=========+=========+=========+=========+=========+
PRV-WT complete  cggcgctggacgactacgcggagctcatggacgcgctcgacccgctggtgcgcgcgtgcccgctcgcggagttctgggagcagcgcgacgccgtcatgcgccagctgcggctgacgccggccccgggcccccccacgggcggcaagcggctcgtcatcgcgcccgcgctgccctcggaggacctggacgggctcgtcccgcccacggccgtgcgccccgcccacttggggccggacgtcgacctcgccgaatacgccgcgcgccacccggagctggtgggcgtgccggaaccccgagccc
PRV-delete UL10  cggcgctggacgactacgcggagctcatggacgcgctcgacccgctggtgcgcgcgtgcccgctcgcggagttctgggagcagcgcgacgccgtcatgcgccagctgcggctgacgccggccccgggcccccccacgggcggcaagcggctcgtcatcgcgcccgcgctgccctcggaggacctggacgggctcgtcccgcccacggccgtgcgccccgcccacttggggccggacgtcgacctcgccgaatacgccgcgcgccacccggagctggtgggcgtgccggaaccccgagccc
                 ############################################################################################################################################################################################################################################################################################################


                      13210     13220     13230     13240     13250     13260     13270     13280     13290     13300     13310     13320     13330     13340     13350     13360     13370     13380     13390     13400     13410     13420     13430     13440     13450     13460     13470     13480     13490     13500
                 =========+=========+=========+=========+=========+=========+=========+=========+=========+=========+=========+=========+=========+=========+=========+=========+=========+=========+=========+=========+=========+=========+=========+=========+=========+=========+=========+=========+=========+=========+
PRV-WT complete  atttatcggcgcgccggccgccccggggccgctaacccgtccctgcctccgccatgatccgccgcgcccgaggaacgcgccgcgcttcgtggaaggatgcctcgcgccgcgtcaccgaggggcgcacccgcgccagctgcctgctggcggcccccggggaggtgctgacggcggccgtggcggccctgcgcgaggcggccgagcgccagtgcgcgcccgcgctcttcggcgcccagcgcgcctcggccctggcgctcgtgcgcagcaactacgcccccgagtccgtgatcacggcgtgct
PRV-delete UL10  atttatcggcgcgccggccgccccggggccgctaacccgtccctgcctccgccatgatccgccgcgcccgaggaacgcgccgcgcttcgtggaaggatgcctcgcgccgcgtcaccgaggggcgcacccgcgccagctgcctgctggcggcccccggggaggtgctgacggcggccgtggcggccctgcgcgaggcggccgagcgccagtgcgcgcccgcgctcttcggcgcccagcgcgcctcggccctggcgctcgtgcgcagcaactacgcccccgagtccgtgatcacggcgtgct
                 ############################################################################################################################################################################################################################################################################################################


                      13510     13520     13530     13540     13550     13560     13570     13580     13590     13600     13610     13620     13630     13640     13650     13660     13670     13680     13690     13700     13710     13720     13730     13740     13750     13760     13770     13780     13790     13800
                 =========+=========+=========+=========+=========+=========+=========+=========+=========+=========+=========+=========+=========+=========+=========+=========+=========+=========+=========+=========+=========+=========+=========+=========+=========+=========+=========+=========+=========+=========+
PRV-WT complete  cggcggagccgcaccgcgaggcgtacgcgcgcgcggccgaggccgcgctgaagggcatctcggcggacgtggcctggcgcgcggtgctggccacctactggcgctacctgaaggccagctcgggcgtggaggtgcgctcggacgacgagcgcgacgcccccaccgtcatgctgctgtggtcgacgttcggcaagacgctgtcgaagcacccgttcaagcacaaggcccagagcgccgcgtacggcgccacgcgcgccgcgctcgccgaggcgaccgaggccgtggagcggtacgcctact
PRV-delete UL10  cggcggagccgcaccgcgaggcgtacgcgcgcgcggccgaggccgcgctgaagggcatctcggcggacgtggcctggcgcgcggtgctggccacctactggcgctacctgaaggccagctcgggcgtggaggtgcgctcggacgacgagcgcgacgcccccaccgtcatgctgctgtggtcgacgttcggcaagacgctgtcgaagcacccgttcaagcacaaggcccagagcgccgcgtacggcgccacgcgcgccgcgctcgccgaggcgaccgaggccgtggagcggtacgcctact
                 ############################################################################################################################################################################################################################################################################################################


                      13810     13820     13830     13840     13850     13860     13870     13880     13890     13900     13910     13920     13930     13940     13950     13960     13970     13980     13990     14000     14010     14020     14030     14040     14050     14060     14070     14080     14090     14100
                 =========+=========+=========+=========+=========+=========+=========+=========+=========+=========+=========+=========+=========+=========+=========+=========+=========+=========+=========+=========+=========+=========+=========+=========+=========+=========+=========+=========+=========+=========+
PRV-WT complete  tcatgcgcccgctcgaccccatggtctccagcccccagacgagcgtgcgcctgcacgagatgctggcgtacgcggccacgctgtaccgctggctgctgtggatgatggacacggtcgacgcgcgcgtgctccgccacctggggcgcacgccgcgggtggtgcgcggcccgcgcgagacgatgtcgcccgaggcgctctttgggcggcaccgcgccggcgggcccgccgtggcctcgggctcgggcgaggtgctcgtgctgacgagccacacggcgacggtgtttgacgccctggagatcc
PRV-delete UL10  tcatgcgcccgctcgaccccatggtctccagcccccagacgagcgtgcgcctgcacgagatgctggcgtacgcggccacgctgtaccgctggctgctgtggatgatggacacggtcgacgcgcgcgtgctccgccacctggggcgcacgccgcgggtggtgcgcggcccgcgcgagacgatgtcgcccgaggcgctctttgggcggcaccgcgccggcgggcccgccgtggcctcgggctcgggcgaggtgctcgtgctgacgagccacacggcgacggtgtttgacgccctggagatcc
                 ############################################################################################################################################################################################################################################################################################################


                      14110     14120     14130     14140     14150     14160     14170     14180     14190     14200     14210     14220     14230     14240     14250     14260     14270     14280     14290     14300     14310     14320     14330     14340     14350     14360     14370     14380     14390     14400
                 =========+=========+=========+=========+=========+=========+=========+=========+=========+=========+=========+=========+=========+=========+=========+=========+=========+=========+=========+=========+=========+=========+=========+=========+=========+=========+=========+=========+=========+=========+
PRV-WT complete  tcggcgccacgtgggccgagacgccctggaagagcggcgtgtgcggcctcacggccgccgtcgtggccgccgtggacctcgtgaccttcgtgcaccaccacgcgcagaccctcatcaacctgacgctcgccggctacgtgtgctggctcgacgacgggatggaggacccgtacctgcgcgcggcgctgcgcgcgcagtgccgcttccaccacctgatcggcgacctggcccccacgagctcgtcgcacgcctggggcgccatggagcgcggcacgctcgcctggttcaactacgccatcg
PRV-delete UL10  tcggcgccacgtgggccgagacgccctggaagagcggcgtgtgcggcctcacggccgccgtcgtggccgccgtggacctcgtgaccttcgtgcaccaccacgcgcagaccctcatcaacctgacgctcgccggctacgtgtgctggctcgacgacgggatggaggacccgtacctgcgcgcggcgctgcgcgcgcagtgccgcttccaccacctgatcggcgacctggcccccacgagctcgtcgcacgcctggggcgccatggagcgcggcacgctcgcctggttcaactacgccatcg
                 ############################################################################################################################################################################################################################################################################################################


                      14410     14420     14430     14440     14450     14460     14470     14480     14490     14500     14510     14520     14530     14540     14550     14560     14570     14580     14590     14600     14610     14620     14630     14640     14650     14660     14670     14680     14690     14700
                 =========+=========+=========+=========+=========+=========+=========+=========+=========+=========+=========+=========+=========+=========+=========+=========+=========+=========+=========+=========+=========+=========+=========+=========+=========+=========+=========+=========+=========+=========+
PRV-WT complete  cgcgcagcctggcctcgtacggcgggcccacggagcgcttcgcgcgcgtgctggcgacgacgggccagcgggacatgcgcttcctggccgtcgcccccgcgcagccgccgctgccgcccctgccgcgggcgtcgcggacgccctcgccgccgccccgcgaccgcgacgcggagccgccccacgactacgtggaccccctcgccgagtacctgaactggcccgtgcccgcctcgccgcccccgctccccgaggggcccccgagctcggacgacgagctggaggtggacggcggcgggcggc
PRV-delete UL10  cgcgcagcctggcctcgtacggcgggcccacggagcgcttcgcgcgcgtgctggcgacgacgggccagcgggacatgcgcttcctggccgtcgcccccgcgcagccgccgctgccgcccctgccgcgggcgtcgcggacgccctcgccgccgccccgcgaccgcgacgcggagccgccccacgactacgtggaccccctcgccgagtacctgaactggcccgtgcccgcctcgccgcccccgctccccgaggggcccccgagctcggacgacgagctggaggtggacggcggcgggcggc
                 ############################################################################################################################################################################################################################################################################################################


                      14710     14720     14730     14740     14750     14760     14770     14780     14790     14800     14810     14820     14830     14840     14850     14860     14870     14880     14890     14900     14910     14920     14930     14940     14950     14960     14970     14980     14990     15000
                 =========+=========+=========+=========+=========+=========+=========+=========+=========+=========+=========+=========+=========+=========+=========+=========+=========+=========+=========+=========+=========+=========+=========+=========+=========+=========+=========+=========+=========+=========+
PRV-WT complete  gcccgctccggcgcagccgcgacgccgccacgtacgtgaaccgcaaggacattgcccgcgcgatggcgggggcggaggacgaggacgaggatgaggaggaggacggccggtacccgcgccgcgacggggacgccacggcctcgacgagctcgtcgcaggagagcgccgccgacgaggagttcccgcccgggatcggcgtgcgcgacggcgaggaggacgacgaggacgagggggtggacgaggaggaggaggaggaggacgtgtacgtcggccccgacggtaccggcgaacggggcgccg
PRV-delete UL10  gcccgctccggcgcagccgcgacgccgccacgtacgtgaaccgcaaggacattgcccgcgcgatggcgggggcggaggacgaggacgaggatgaggaggaggacggccggtacccgcgccgcgacggggacgccacggcctcgacgagctcgtcgcaggagagcgccgccgacgaggagttcccgcccgggatcggcgtgcgcgacggcgaggaggacgacgaggacgagggggtggacgaggaggaggaggaggaggacgtgtacgtcggccccgacggtaccggcgaacggggcgccg
                 ############################################################################################################################################################################################################################################################################################################


                      15010     15020     15030     15040     15050     15060     15070     15080     15090     15100     15110     15120     15130     15140     15150     15160     15170     15180     15190     15200     15210     15220     15230     15240     15250     15260     15270     15280     15290     15300
                 =========+=========+=========+=========+=========+=========+=========+=========+=========+=========+=========+=========+=========+=========+=========+=========+=========+=========+=========+=========+=========+=========+=========+=========+=========+=========+=========+=========+=========+=========+
PRV-WT complete  ccggcggcggcaacgccgcggacgacgacgacggcgccccctgggccccgctgacgcgccacgggagcatgcgcaccagcttccggcgcggggtccgcgcggcccagcgcttcgtgcgccgccggctctcgcgcacgagcgccgaggcggccccgcgggcctccggcgactcagcctccgcggcggcccccgccgccgcctccgcccgcggcgagaccgaccacgtgtaccagcacccccgcccgcggacccgcgcggacgacggcctgtaccagcaaccccgacccgtcatcgacctca
PRV-delete UL10  ccggcggcggcaacgccgcggacgacgacgacggcgccccctgggccccgctgacgcgccacgggagcatgcgcaccagcttccggcgcggggtccgcgcggcccagcgcttcgtgcgccgccggctctcgcgcacgagcgccgaggcggccccgcgggcctccggcgactcagcctccgcggcggcccccgccgccgcctccgcccgcggcgagaccgaccacgtgtaccagcacccccgcccgcggacccgcgcggacgacggcctgtaccagcaaccccgacccgtcatcgacctca
                 ############################################################################################################################################################################################################################################################################################################


                      15310     15320     15330     15340     15350     15360     15370     15380     15390     15400     15410     15420     15430     15440     15450     15460     15470     15480     15490     15500     15510     15520     15530     15540     15550     15560     15570     15580     15590     15600
                 =========+=========+=========+=========+=========+=========+=========+=========+=========+=========+=========+=========+=========+=========+=========+=========+=========+=========+=========+=========+=========+=========+=========+=========+=========+=========+=========+=========+=========+=========+
PRV-WT complete  ccggccaccgcgcgtcgcgccgcaagagctggcgcgtgtgacggctcccacggactcaccgttgtattgttttcccccgttgcccccctgtgtggaaataaagtttttttctaattctgtacacaaggcgtgtgtgtcgtggcggtcgtggtcgcgggacgggagggcggcggggagaggggagacgagagggggagaggggagacgagaggggagaggggagacgagaggggagaggggagacgagaggggagaggggagacgagaggggagaggggagacgagaggggagaggggaga
PRV-delete UL10  ccggccaccgcgcgtcgcgccgcaagagctggcgcgtgtgacggctcccacggactcaccgttgtattgttttcccccgttgcccccctgtgtggaaataaagtttttttctaattctgtacacaaggcgtgtgtgtcgtggcggtcgtggtcgcgggacgggagggcggcggggagaggggagacgagagggggagaggggagacgagaggggagaggggagacgagaggggagaggggagacgagaggggagaggggagacgagaggggagaggggagacgagaggggagaggggaga
                 ############################################################################################################################################################################################################################################################################################################


                      15610     15620     15630     15640     15650     15660     15670     15680     15690     15700     15710     15720     15730     15740     15750     15760     15770     15780     15790     15800     15810     15820     15830     15840     15850     15860     15870     15880     15890     15900
                 =========+=========+=========+=========+=========+=========+=========+=========+=========+=========+=========+=========+=========+=========+=========+=========+=========+=========+=========+=========+=========+=========+=========+=========+=========+=========+=========+=========+=========+=========+
PRV-WT complete  cgagaggggagaggggagacgagaggggagagggacgaggcggacgcgagacgtgcgatcaacggcatctttatttttttccatctgcggggagggggctagggggcgtcggggtcctcgttctcgaggcgctggtagtgccggcggcgcgtggccatcgccccgacgcggctggccagcagcgcgggcccgctgttcttcttgcgcgccttgtgctcctgctgctcgagggccgacacgatggacatgtaccggatcatgtcccgggcctggtccagcttggcctcgtccacgtcgtcc
PRV-delete UL10  cgagaggggagaggggagacgagaggggagagggacgaggcggacgcgagacgtgcgatcaacggcatctttatttttttccatctgcggggagggggctagggggcgtcggggtcctcgttctcgaggcgctggtagtgccggcggcgcgtggccatcgccccgacgcggctggccagcagcgcgggcccgctgttcttcttgcgcgccttgtgctcctgctgctcgagggccgacacgatggacatgtaccggatcatgtcccgggcctggtccagcttggcctcgtccacgtcgtcc
                 ############################################################################################################################################################################################################################################################################################################


                      15910     15920     15930     15940     15950     15960     15970     15980     15990     16000     16010     16020     16030     16040     16050     16060     16070     16080     16090     16100     16110     16120     16130     16140     16150     16160     16170     16180     16190     16200
                 =========+=========+=========+=========+=========+=========+=========+=========+=========+=========+=========+=========+=========+=========+=========+=========+=========+=========+=========+=========+=========+=========+=========+=========+=========+=========+=========+=========+=========+=========+
PRV-WT complete  tcttcgacgccgtcctccttgagcgccttcgtcgtgacggggtacagggccttcatggggttgcggcgcaggcgcgagatgtgccggtaggccaggaaggccgcgaccaggccggccagcaccagcagcccgatggcgagcgccccgaaggggttggacaggaaggacaccatgccgccgacggccgagatcacggcccccgtggcgcccaggaccaccttgccgacggcggcgcccacgtcgccgaggccctggaagaagttggcgatgccgcgcagcagcaccacgttgtggtccacc
PRV-delete UL10  tcttcgacgccgtcctccttgagcgccttcgtcgtgacggggtacagggccttcatggggttgcggcgcaggcgcgagatgtgccggtaggccaggaaggccgcgaccaggccggccagcaccagcagcccgatggcgagcgccccgaaggggttggacaggaaggacaccatgccgccgacggccgagatcacggcccccgtggcgcccaggaccaccttgccgacggcggcgcccacgtcgccgaggccctggaagaagttggcgatgccgcgcagcagcaccacgttgtggtccacc
                 ############################################################################################################################################################################################################################################################################################################


                      16210     16220     16230     16240     16250     16260     16270     16280     16290     16300     16310     16320     16330     16340     16350     16360     16370     16380     16390     16400     16410     16420     16430     16440     16450     16460     16470     16480     16490     16500
                 =========+=========+=========+=========+=========+=========+=========+=========+=========+=========+=========+=========+=========+=========+=========+=========+=========+=========+=========+=========+=========+=========+=========+=========+=========+=========+=========+=========+=========+=========+
PRV-WT complete  ttgaccacgcggtcaatgtcgtagaacttgagcgcgtgcagctggttgcggcgctggatctcgctgtagtccaggaggcccgtgtcggcgagctcctcgcgcgtgtacacctcgaggggcaggaactcgcggtcctcgagcagcgtcaggttcagggtcacccgcgtgctgatcgtctcgggcacctccaccatgcgcacgtagctgtagtcctcgtagtacacgtacccgccgcccagcttaaagtagcgccggtggttgccggtgcagggctcgatgaggtcgcgcgagatgaggagc
PRV-delete UL10  ttgaccacgcggtcaatgtcgtagaacttgagcgcgtgcagctggttgcggcgctggatctcgctgtagtccaggaggcccgtgtcggcgagctcctcgcgcgtgtacacctcgaggggcaggaactcgcggtcctcgagcagcgtcaggttcagggtcacccgcgtgctgatcgtctcgggcacctccaccatgcgcacgtagctgtagtcctcgtagtacacgtacccgccgcccagcttaaagtagcgccggtggttgccggtgcagggctcgatgaggtcgcgcgagatgaggagc
                 ############################################################################################################################################################################################################################################################################################################


                      16510     16520     16530     16540     16550     16560     16570     16580     16590     16600     16610     16620     16630     16640     16650     16660     16670     16680     16690     16700     16710     16720     16730     16740     16750     16760     16770     16780     16790     16800
                 =========+=========+=========+=========+=========+=========+=========+=========+=========+=========+=========+=========+=========+=========+=========+=========+=========+=========+=========+=========+=========+=========+=========+=========+=========+=========+=========+=========+=========+=========+
PRV-WT complete  tcgttgtcgtcgccgagctggccctcgatcacgcccgtgccgttgtgctcgaaggtcaccagcgggcggctgtagcacgtgccgcgctcgccgggcacgcgcatggagttctgcacgtacacgccgccgcgcacctccacgcaccgcgagatggccatcacgtcgccgagcatgcgcgccgagacgcgctggcccagcgcggccgtggccacggcgctggggttcaggcgcgacatctcgccccacagggtgcggtccttgttctgcagctcgcaccaggcggccgcgatgcggctcagc
PRV-delete UL10  tcgttgtcgtcgccgagctggccctcgatcacgcccgtgccgttgtgctcgaaggtcaccagcgggcggctgtagcacgtgccgcgctcgccgggcacgcgcatggagttctgcacgtacacgccgccgcgcacctccacgcaccgcgagatggccatcacgtcgccgagcatgcgcgccgagacgcgctggcccagcgcggccgtggccacggcgctggggttcaggcgcgacatctcgccccacagggtgcggtccttgttctgcagctcgcaccaggcggccgcgatgcggctcagc
                 ############################################################################################################################################################################################################################################################################################################


                      16810     16820     16830     16840     16850     16860     16870     16880     16890     16900     16910     16920     16930     16940     16950     16960     16970     16980     16990     17000     17010     17020     17030     17040     17050     17060     17070     17080     17090     17100
                 =========+=========+=========+=========+=========+=========+=========+=========+=========+=========+=========+=========+=========+=========+=========+=========+=========+=========+=========+=========+=========+=========+=========+=========+=========+=========+=========+=========+=========+=========+
PRV-WT complete  atgtcgttcacgtgcgcctggatgtggtcgtaggtgaactgcaggcgcgcaaactcggccgagcccgtggtgatgcgcaggtgccccgtgccgttgacggccggcggctcgggcgtccccgccgggccgggggagcgccgggcccgacgggcggccgcgggggacgcggggcccacgacgccggcgaggccgaggcgctcgagctcgcgcgcgtacagctgcgccagctcgttcgagatcagcgggcggaaggccaccacgaagcccccgcgggcgaggtacacctcgggcttgtcgccg
PRV-delete UL10  atgtcgttcacgtgcgcctggatgtggtcgtaggtgaactgcaggcgcgcaaactcggccgagcccgtggtgatgcgcaggtgccccgtgccgttgacggccggcggctcgggcgtccccgccgggccgggggagcgccgggcccgacgggcggccgcgggggacgcggggcccacgacgccggcgaggccgaggcgctcgagctcgcgcgcgtacagctgcgccagctcgttcgagatcagcgggcggaaggccaccacgaagcccccgcgggcgaggtacacctcgggcttgtcgccg
                 ############################################################################################################################################################################################################################################################################################################


                      17110     17120     17130     17140     17150     17160     17170     17180     17190     17200     17210     17220     17230     17240     17250     17260     17270     17280     17290     17300     17310     17320     17330     17340     17350     17360     17370     17380     17390     17400
                 =========+=========+=========+=========+=========+=========+=========+=========+=========+=========+=========+=========+=========+=========+=========+=========+=========+=========+=========+=========+=========+=========+=========+=========+=========+=========+=========+=========+=========+=========+
PRV-WT complete  gccagcacgtgcgtgttgttgtagcgccgccggtagatggcgtcgatggcctccgaggcctcgcggaggacgcagtcgcccaggtgcacgcgctgcaggtcgagctgcgtgacgtcgctgacgaaggaggcgcccagggcccgcgacgtgaagcggaaggacccgtcgcgcgtctcgtcgcggatcatctcctcggcctcgcgccacttggccaggctgcacacgcgccgcgtcttgggggcccagtcccaggccaccgtgaagtgcggcgtgcgcagaaagttgcgcgtcacgctctcg
PRV-delete UL10  gccagcacgtgcgtgttgttgtagcgccgccggtagatggcgtcgatggcctccgaggcctcgcggaggacgcagtcgcccaggtgcacgcgctgcaggtcgagctgcgtgacgtcgctgacgaaggaggcgcccagggcccgcgacgtgaagcggaaggacccgtcgcgcgtctcgtcgcggatcatctcctcggcctcgcgccacttggccaggctgcacacgcgccgcgtcttgggggcccagtcccaggccaccgtgaagtgcggcgtgcgcagaaagttgcgcgtcacgctctcg
                 ############################################################################################################################################################################################################################################################################################################


                      17410     17420     17430     17440     17450     17460     17470     17480     17490     17500     17510     17520     17530     17540     17550     17560     17570     17580     17590     17600     17610     17620     17630     17640     17650     17660     17670     17680     17690     17700
                 =========+=========+=========+=========+=========+=========+=========+=========+=========+=========+=========+=========+=========+=========+=========+=========+=========+=========+=========+=========+=========+=========+=========+=========+=========+=========+=========+=========+=========+=========+
PRV-WT complete  gaggcgcggaggcgcgagtccaggtcgatggggtagtagtgctccacctgctggaagcgcccgggcgcgtagccgatgtgctccccgtgggccccctcgcgcaggccgtagaagggggacatgtacacgatgtcccccgtggacagggcgaaggagtcgtaggggtacacggagcgcgcctccacctcctcgacgatgcagttgacggaggtgcccgtgtggtagaagcccgcggcgccgatcttggtgtaggtgtcgttggtggtgtgccagccgcgggtgccgagcgcgttcaggcgc
PRV-delete UL10  gaggcgcggaggcgcgagtccaggtcgatggggtagtagtgctccacctgctggaagcgcccgggcgcgtagccgatgtgctccccgtgggccccctcgcgcaggccgtagaagggggacatgtacacgatgtcccccgtggacagggcgaaggagtcgtaggggtacacggagcgcgcctccacctcctcgacgatgcagttgacggaggtgcccgtgtggtagaagcccgcggcgccgatcttggtgtaggtgtcgttggtggtgtgccagccgcgggtgccgagcgcgttcaggcgc
                 ############################################################################################################################################################################################################################################################################################################


                      17710     17720     17730     17740     17750     17760     17770     17780     17790     17800     17810     17820     17830     17840     17850     17860     17870     17880     17890     17900     17910     17920     17930     17940     17950     17960     17970     17980     17990     18000
                 =========+=========+=========+=========+=========+=========+=========+=========+=========+=========+=========+=========+=========+=========+=========+=========+=========+=========+=========+=========+=========+=========+=========+=========+=========+=========+=========+=========+=========+=========+
PRV-WT complete  gaggggcgcaggtccacctcgacggggttctcgtcgcggtcgaaggcggtcaccttgtggttgttgcgcacgtactcggccttggagacgcacttgccgcggcggtcgatcacgtccgtgatctcctgcacggggacgggcacgcggtccgtgaagcggttcgtgatggccgcgtacgtgctcccggaccacacggtcgtgacgatgacgttcttgtagtagatgtgggccttgaacttgtgcggggcgatgttctccttgaagagcacggcgatcccctccgtgaagttgcgcccctgc
PRV-delete UL10  gaggggcgcaggtccacctcgacggggttctcgtcgcggtcgaaggcggtcaccttgtggttgttgcgcacgtactcggccttggagacgcacttgccgcggcggtcgatcacgtccgtgatctcctgcacggggacgggcacgcggtccgtgaagcggttcgtgatggccgcgtacgtgctcccggaccacacggtcgtgacgatgacgttcttgtagtagatgtgggccttgaacttgtgcggggcgatgttctccttgaagagcacggcgatcccctccgtgaagttgcgcccctgc
                 ############################################################################################################################################################################################################################################################################################################


                      18010     18020     18030     18040     18050     18060     18070     18080     18090     18100     18110     18120     18130     18140     18150     18160     18170     18180     18190     18200     18210     18220     18230     18240     18250     18260     18270     18280     18290     18300
                 =========+=========+=========+=========+=========+=========+=========+=========+=========+=========+=========+=========+=========+=========+=========+=========+=========+=========+=========+=========+=========+=========+=========+=========+=========+=========+=========+=========+=========+=========+
PRV-WT complete  gagtactcggggcaggcctgctcgggctccaggcgcaccaccgtggagccggacggcggcgggcagacgtagaagcggtcccgctcggtcgcggccgcgcgcacggccgtgcgcgcgtccaggtcgccgtactcgccgtcgggggcgtccgaggggccgggggagacggccccgtcgatctcctcgagggactcctccgcggagaagccgtctggggtggcgcccgtcccgggcgcgggcgaggccgaggcggcccgcgtcacggccgccgcgccgcacgtcggggtcgcggcgagcgcc
PRV-delete UL10  gagtactcggggcaggcctgctcgggctccaggcgcaccaccgtggagccggacggcggcgggcagacgtagaagcggtcccgctcggtcgcggccgcgcgcacggccgtgcgcgcgtccaggtcgccgtactcgccgtcgggggcgtccgaggggccgggggagacggccccgtcgatctcctcgagggactcctccgcggagaagccgtctggggtggcgcccgtcccgggcgcgggcgaggccgaggcggcccgcgtcacggccgccgcgccgcacgtcggggtcgcggcgagcgcc
                 ############################################################################################################################################################################################################################################################################################################


                      18310     18320     18330     18340     18350     18360     18370     18380     18390     18400     18410     18420     18430     18440     18450     18460     18470     18480     18490     18500     18510     18520     18530     18540     18550     18560     18570     18580     18590     18600
                 =========+=========+=========+=========+=========+=========+=========+=========+=========+=========+=========+=========+=========+=========+=========+=========+=========+=========+=========+=========+=========+=========+=========+=========+=========+=========+=========+=========+=========+=========+
PRV-WT complete  agcagcagcagcgctagcgcgacggcgccccgcgcagctgcagcgtggtgtggagcaggccaaagacgtccgaggccagcaccgccgtggtgcccgggccgatgcccgcggggcccgcgccaaagaccgccaccagcgggcatgacgcctcgtaggtcaggtacagcccgtcgcgtccggcgaagcgatccgcgcggaggacctcgacgtccccgcagtggaagacggacctgaagactgcaaccgccagcaccagctcgcggatatatcgccaggcctgccgctgggtggcggttacgc
PRV-delete UL10  agcagcagcagcgctagcgcgacggcgccccgcgcagctgcagcgtggtgtggagcaggccaaagacgtccgaggccagcaccgccgtggtgcccgggccgatgcccgcggggcccgcgccaaagaccgccaccagcgggcatgacgcctcgtaggtcaggtacagcccgtcgcgtccggcgaagcgatccgcgcggaggacctcgacgtccccgcagtggaagacggacctgaagactgcaaccgccagcaccagctcgcggatatatcgccaggcctgccgctgggtggcggttacgc
                 ############################################################################################################################################################################################################################################################################################################


                      18610     18620     18630     18640     18650     18660     18670     18680     18690     18700     18710     18720     18730     18740     18750     18760     18770     18780     18790     18800     18810     18820     18830     18840     18850     18860     18870     18880     18890     18900
                 =========+=========+=========+=========+=========+=========+=========+=========+=========+=========+=========+=========+=========+=========+=========+=========+=========+=========+=========+=========+=========+=========+=========+=========+=========+=========+=========+=========+=========+=========+
PRV-WT complete  cggacgtctggaagcggtagaagccgcggaactcgctgacgcaccaatcgcgggccaccatgaagcgcgccagctcctccttcaggtgcggcaggaggcccacgttctccaccgcaaagtacagcgccgtgttggggggctgggcgaaggcgtgcgtgtcgtgcgcgaagagcggcccgttgacgagctcgaagaacttgtgcgtgagcgccgggaggagcgcggcgtccacggggtggcgctgcaccgtggccttcatgaagctgtgcgcgtcgaagcagcccgtctcgcgctcgtcca
PRV-delete UL10  cggacgtctggaagcggtagaagccgcggaactcgctgacgcaccaatcgcgggccaccatgaagcgcgccagctcctccttcaggtgcggcaggaggcccacgttctccaccgcaaagtacagcgccgtgttggggggctgggcgaaggcgtgcgtgtcgtgcgcgaagagcggcccgttgacgagctcgaagaacttgtgcgtgagcgccgggaggagcgcggcgtccacggggtggcgctgcaccgtggccttcatgaagctgtgcgcgtcgaagcagcccgtctcgcgctcgtcca
                 ############################################################################################################################################################################################################################################################################################################


                      18910     18920     18930     18940     18950     18960     18970     18980     18990     19000     19010     19020     19030     19040     19050     19060     19070     19080     19090     19100     19110     19120     19130     19140     19150     19160     19170     19180     19190     19200
                 =========+=========+=========+=========+=========+=========+=========+=========+=========+=========+=========+=========+=========+=========+=========+=========+=========+=========+=========+=========+=========+=========+=========+=========+=========+=========+=========+=========+=========+=========+
PRV-WT complete  cgacggtgcccgcgcgcgccacggcctcgcagaacgcgcgccgcgcgcggaacccggcggccaccgccacgtacgtgtgcagcagcgcgtcgccgtacacgttcacgcgcagggtcttctccagctcgcgccgctgctcgcgcacgcactgcgccaggctcgccgccgagcgccgcgagaggcggtccgcgtacaggcgccgccggcgctcggcgtcgcgcagggccgcggccggcaggtccgcccacgccacggcgtccgccggcgggggcgcgccgggcccgtcctcgccgccaccgc
PRV-delete UL10  cgacggtgcccgcgcgcgccacggcctcgcagaacgcgcgccgcgcgcggaacccggcggccaccgccacgtacgtgtgcagcagcgcgtcgccgtacacgttcacgcgcagggtcttctccagctcgcgccgctgctcgcgcacgcactgcgccaggctcgccgccgagcgccgcgagaggcggtccgcgtacaggcgccgccggcgctcggcgtcgcgcagggccgcggccggcaggtccgcccacgccacggcgtccgccggcgggggcgcgccgggcccgtcctcgccgccaccgc
                 ############################################################################################################################################################################################################################################################################################################


                      19210     19220     19230     19240     19250     19260     19270     19280     19290     19300     19310     19320     19330     19340     19350     19360     19370     19380     19390     19400     19410     19420     19430     19440     19450     19460     19470     19480     19490     19500
                 =========+=========+=========+=========+=========+=========+=========+=========+=========+=========+=========+=========+=========+=========+=========+=========+=========+=========+=========+=========+=========+=========+=========+=========+=========+=========+=========+=========+=========+=========+
PRV-WT complete  cccccgcgccgccgccggcgtccgcgccccccgcggcgccgccctcggggtccgcgtcgtcgccgccctcgaggaggcgctcgagggcgtcgcggttggcccggtccgggtccagcatgcgcagcatgggcgtggacatgtggtggtcgtagcacgcgcggatgagcgcctcgaggcgctcgtcgggcgaggccgcacagccgcccaccagcagcccgtcgatgatgtcgagctcgcgggccgcgcgggcgcggtcaaagtgctcggggcgccgcccgaagagcgcgagctccacggccg
PRV-delete UL10  cccccgcgccgccgccggcgtccgcgccccccgcggcgccgccctcggggtccgcgtcgtcgccgccctcgaggaggcgctcgagggcgtcgcggttggcccggtccgggtccagcatgcgcagcatgggcgtggacatgtggtggtcgtagcacgcgcggatgagcgcctcgaggcgctcgtcgggcgaggccgcacagccgcccaccagcagcccgtcgatgatgtcgagctcgcgggccgcgcgggcgcggtcaaagtgctcggggcgccgcccgaagagcgcgagctccacggccg
                 ############################################################################################################################################################################################################################################################################################################


                      19510     19520     19530     19540     19550     19560     19570     19580     19590     19600     19610     19620     19630     19640     19650     19660     19670     19680     19690     19700     19710     19720     19730     19740     19750     19760     19770     19780     19790     19800
                 =========+=========+=========+=========+=========+=========+=========+=========+=========+=========+=========+=========+=========+=========+=========+=========+=========+=========+=========+=========+=========+=========+=========+=========+=========+=========+=========+=========+=========+=========+
PRV-WT complete  ccgcgcgcaccgcggcgcggcgctcgcgccgctccaggtcgtccaggttgtgcgtgaacaggtcgagggcccgcaccgcctggttagtggacgccagccagaactgcagctcgctcacggcgtacaggcgccgcgaggcgggccggaacacgtcgtgcgcgtccaggagcgcgtccgcgcgccggcgcgccgtctcgcccgcgtccccggcctcggcgcccgccgccgccgcctccgcctcccgggcctcggccgcgtccacggcggcgagcgccgccagcgcgcgcccgcggcgcgcct
PRV-delete UL10  ccgcgcgcaccgcggcgcggcgctcgcgccgctccaggtcgtccaggttgtgcgtgaacaggtcgagggcccgcaccgcctggttagtggacgccagccagaactgcagctcgctcacggcgtacaggcgccgcgaggcgggccggaacacgtcgtgcgcgtccaggagcgcgtccgcgcgccggcgcgccgtctcgcccgcgtccccggcctcggcgcccgccgccgccgcctccgcctcccgggcctcggccgcgtccacggcggcgagcgccgccagcgcgcgcccgcggcgcgcct
                 ############################################################################################################################################################################################################################################################################################################


                      19810     19820     19830     19840     19850     19860     19870     19880     19890     19900     19910     19920     19930     19940     19950     19960     19970     19980     19990     20000     20010     20020     20030     20040     20050     20060     20070     20080     20090     20100
                 =========+=========+=========+=========+=========+=========+=========+=========+=========+=========+=========+=========+=========+=========+=========+=========+=========+=========+=========+=========+=========+=========+=========+=========+=========+=========+=========+=========+=========+=========+
PRV-WT complete  cgtcgagcccgcgcacgtgcggcaggttcttggccacgtcctcggggtccacgcgcaccgccagctgccgcgtgaggtggtcgcacacgcagccgagcaggcgccggtgcgtggcctcgccctggttggccgtcacgcacagctcctcgaagcacaccgcgcacggctgcgccgggtcgtacagctccggcggcaccacgagcccgccgccgatcgtgcgcgcgaggaactcgtccacggccgacaggtccaccgcgtggggcgtgatcaggtggcagtagttgagctgcttgagcaggt
PRV-delete UL10  cgtcgagcccgcgcacgtgcggcaggttcttggccacgtcctcggggtccacgcgcaccgccagctgccgcgtgaggtggtcgcacacgcagccgagcaggcgccggtgcgtggcctcgccctggttggccgtcacgcacagctcctcgaagcacaccgcgcacggctgcgccgggtcgtacagctccggcggcaccacgagcccgccgccgatcgtgcgcgcgaggaactcgtccacggccgacaggtccaccgcgtggggcgtgatcaggtggcagtagttgagctgcttgagcaggt
                 ############################################################################################################################################################################################################################################################################################################


                      20110     20120     20130     20140     20150     20160     20170     20180     20190     20200     20210     20220     20230     20240     20250     20260     20270     20280     20290     20300     20310     20320     20330     20340     20350     20360     20370     20380     20390     20400
                 =========+=========+=========+=========+=========+=========+=========+=========+=========+=========+=========+=========+=========+=========+=========+=========+=========+=========+=========+=========+=========+=========+=========+=========+=========+=========+=========+=========+=========+=========+
PRV-WT complete  tctcggcgtcgtggaggaactgcagctccgtctccacgcgcccgccgtacgtgtcgagcgcgacgcgcgcgtggaagcggcacgcgccgcccatggtgcgctcaaagtaggcctcggcgtcgtcgccggcggcggccagcgcccccagcacgcggtccccctcggcccgcgcgtacgcgagcgccaggcgcagcgcgcacgtgagcggcgtcatctgcgcctccagcggcagccgccgcgcgaggtagcggcacatcagcgcgttcagcttgacgcgcggggccagctcgcgcaccaccg
PRV-delete UL10  tctcggcgtcgtggaggaactgcagctccgtctccacgcgcccgccgtacgtgtcgagcgcgacgcgcgcgtggaagcggcacgcgccgcccatggtgcgctcaaagtaggcctcggcgtcgtcgccggcggcggccagcgcccccagcacgcggtccccctcggcccgcgcgtacgcgagcgccaggcgcagcgcgcacgtgagcggcgtcatctgcgcctccagcggcagccgccgcgcgaggtagcggcacatcagcgcgttcagcttgacgcgcggggccagctcgcgcaccaccg
                 ############################################################################################################################################################################################################################################################################################################


                      20410     20420     20430     20440     20450     20460     20470     20480     20490     20500     20510     20520     20530     20540     20550     20560     20570     20580     20590     20600     20610     20620     20630     20640     20650     20660     20670     20680     20690     20700
                 =========+=========+=========+=========+=========+=========+=========+=========+=========+=========+=========+=========+=========+=========+=========+=========+=========+=========+=========+=========+=========+=========+=========+=========+=========+=========+=========+=========+=========+=========+
PRV-WT complete  cggggtcgcagcgcttcagcatctccagctgaaacacgtacgtctgcacctgccccaggaccgccacgagccgccgctccgccatggccgagtccgcccgctcgctccctccctccgcccgcccgtccgccgcgagagaggccacgggacgcgcgtgtgtgtctgtcaaacgcggtcatctttattgatacagccgggcacccgcgcccgtcccctcctcctcctccccccatcacagcatgtcgaaggtcagccgcttctcgggcggggcgcccatgtcaaacaggtcgtcctccgggg
PRV-delete UL10  cggggtcgcagcgcttcagcatctccagctgaaacacgtacgtctgcacctgccccaggaccgccacgagccgccgctccgccatggccgagtccgcccgctcgctccctccctccgcccgcccgtccgccgcgagagaggccacgggacgcgcgtgtgtgtctgtcaaacgcggtcatctttattgatacagccgggcacccgcgcccgtcccctcctcctcctccccccatcacagcatgtcgaaggtcagccgcttctcgggcggggcgcccatgtcaaacaggtcgtcctccgggg
                 ############################################################################################################################################################################################################################################################################################################


                      20710     20720     20730     20740     20750     20760     20770     20780     20790     20800     20810     20820     20830     20840     20850     20860     20870     20880     20890     20900     20910     20920     20930     20940     20950     20960     20970     20980     20990     21000
                 =========+=========+=========+=========+=========+=========+=========+=========+=========+=========+=========+=========+=========+=========+=========+=========+=========+=========+=========+=========+=========+=========+=========+=========+=========+=========+=========+=========+=========+=========+
PRV-WT complete  gcgggcgcttgccgcccagctccgcgggcgccagcgaggccggcgcgagccccgcgcccgcgtccccgcccgcgccgcaggccccaaagtcgaaggcggtctcctcgccgccgccgtccgcgcccgtctggccctcgaggtcgcgcaccagctcggcggccgcctccacgctccagcgcccgtcgcggtcggccacgcgcccgttgatctccgccagcgcggccgccaggaactcgtcgtccacgatcgcccgccagtcgtcgggctccaggtgctgcgtgcgcgccccgagcgcgtgca
PRV-delete UL10  gcgggcgcttgccgcccagctccgcgggcgccagcgaggccggcgcgagccccgcgcccgcgtccccgcccgcgccgcaggccccaaagtcgaaggcggtctcctcgccgccgccgtccgcgcccgtctggccctcgaggtcgcgcaccagctcggcggccgcctccacgctccagcgcccgtcgcggtcggccacgcgcccgttgatctccgccagcgcggccgccaggaactcgtcgtccacgatcgcccgccagtcgtcgggctccaggtgctgcgtgcgcgccccgagcgcgtgca
                 ############################################################################################################################################################################################################################################################################################################


                      21010     21020     21030     21040     21050     21060     21070     21080     21090     21100     21110     21120     21130     21140     21150     21160     21170     21180     21190     21200     21210     21220     21230     21240     21250     21260     21270     21280     21290     21300
                 =========+=========+=========+=========+=========+=========+=========+=========+=========+=========+=========+=========+=========+=========+=========+=========+=========+=========+=========+=========+=========+=========+=========+=========+=========+=========+=========+=========+=========+=========+
PRV-WT complete  gcacggccgcgtacacggccgtctgcaccagcggcccgccgtcggccaggatggtcttgacgtgctccccgagcgagtggtcgtgcagcccggcgccgccgcccgtctgcgagcacgtgaagcccacgcgcgggcaggccagcacaaagtgccgcgtgcggtcaaacaccatcagggggcacacgtgcttgccgccgttgagcccgctccagttccccgcctggaagacgcggttgttgccggccatgccgtggtacttgctgacgcccaggcccagcaccaccagcgggcgcgaggcca
PRV-delete UL10  gcacggccgcgtacacggccgtctgcaccagcggcccgccgtcggccaggatggtcttgacgtgctccccgagcgagtggtcgtgcagcccggcgccgccgcccgtctgcgagcacgtgaagcccacgcgcgggcaggccagcacaaagtgccgcgtgcggtcaaacaccatcagggggcacacgtgcttgccgccgttgagcccgctccagttccccgcctggaagacgcggttgttgccggccatgccgtggtacttgctgacgcccaggcccagcaccaccagcgggcgcgaggcca
                 ############################################################################################################################################################################################################################################################################################################


                      21310     21320     21330     21340     21350     21360     21370     21380     21390     21400     21410     21420     21430     21440     21450     21460     21470     21480     21490     21500     21510     21520     21530     21540     21550     21560     21570     21580     21590     21600
                 =========+=========+=========+=========+=========+=========+=========+=========+=========+=========+=========+=========+=========+=========+=========+=========+=========+=========+=========+=========+=========+=========+=========+=========+=========+=========+=========+=========+=========+=========+
PRV-WT complete  tgacggagcggagcaggtggctggacgtgtacgtggccacccacgtgtcgtcgcgcggcgcgtccagcacctcgcgcgcggcgcgctccgcgtcggccgcgtccgccagcgggcgccgcacccaggccatgacggcggccgggtcccgcgggcgcttcgactgcatcgtgatccccgtgaggctgttgatgaagaactgcttgtggtcgcagtagcgcaggatcaggttggccaggtagaactgggccagctccgccacggtgcccggggtcaggttcacgtagttgatggcgccgtagt
PRV-delete UL10  tgacggagcggagcaggtggctggacgtgtacgtggccacccacgtgtcgtcgcgcggcgcgtccagcacctcgcgcgcggcgcgctccgcgtcggccgcgtccgccagcgggcgccgcacccaggccatgacggcggccgggtcccgcgggcgcttcgactgcatcgtgatccccgtgaggctgttgatgaagaactgcttgtggtcgcagtagcgcaggatcaggttggccaggtagaactgggccagctccgccacggtgcccggggtcaggttcacgtagttgatggcgccgtagt
                 ############################################################################################################################################################################################################################################################################################################


                      21610     21620     21630     21640     21650     21660     21670     21680     21690     21700     21710     21720     21730     21740     21750     21760     21770     21780     21790     21800     21810     21820     21830     21840     21850     21860     21870     21880     21890     21900
                 =========+=========+=========+=========+=========+=========+=========+=========+=========+=========+=========+=========+=========+=========+=========+=========+=========+=========+=========+=========+=========+=========+=========+=========+=========+=========+=========+=========+=========+=========+
PRV-WT complete  ccaccgagaagcgcttcacggccgcgatggtctcgatgtcgtccttggtcagcagccgcgccggcagctggttgcgctgcagcagcgtccagaaccactgcgggttggggctctgcccgccgggcggcttgccgcgcgggaacagcgtcgcgtggtgctgcttcagcaggaagcccagcgggccgttgagcacgtccacggccgtgtccgggcgctggtaggcgcccgcgagcccggccacgcgggcgcgcgcggcgtccgagaggctgcccgagcccgtcgagaacatgacccggctct
PRV-delete UL10  ccaccgagaagcgcttcacggccgcgatggtctcgatgtcgtccttggtcagcagccgcgccggcagctggttgcgctgcagcagcgtccagaaccactgcgggttggggctctgcccgccgggcggcttgccgcgcgggaacagcgtcgcgtggtgctgcttcagcaggaagcccagcgggccgttgagcacgtccacggccgtgtccgggcgctggtaggcgcccgcgagcccggccacgcgggcgcgcgcggcgtccgagaggctgcccgagcccgtcgagaacatgacccggctct
                 ############################################################################################################################################################################################################################################################################################################


                      21910     21920     21930     21940     21950     21960     21970     21980     21990     22000     22010     22020     22030     22040     22050     22060     22070     22080     22090     22100     22110     22120     22130     22140     22150     22160     22170     22180     22190     22200
                 =========+=========+=========+=========+=========+=========+=========+=========+=========+=========+=========+=========+=========+=========+=========+=========+=========+=========+=========+=========+=========+=========+=========+=========+=========+=========+=========+=========+=========+=========+
PRV-WT complete  tgacgcgcagctcccgcagcacctcgaagctcacgcgcgccaggtccccgtcgtggtcgcgcgccgggggctccgcgctgcccttggtggggtccggggccacgatgccgtcggcgagcgtcaccgtcaccgtcttggccgtgacgaagcccccgttgagcatgtccaccacgcggcggcgcagcaccggctggaactggttgcggaagttgcgcccctccacgggctgcccgtggagcacgccgtggcactggctcagggccaggtcctgcagcaccgccagcagcgtgcgccgcgcca
PRV-delete UL10  tgacgcgcagctcccgcagcacctcgaagctcacgcgcgccaggtccccgtcgtggtcgcgcgccgggggctccgcgctgcccttggtggggtccggggccacgatgccgtcggcgagcgtcaccgtcaccgtcttggccgtgacgaagcccccgttgagcatgtccaccacgcggcggcgcagcaccggctggaactggttgcggaagttgcgcccctccacgggctgcccgtggagcacgccgtggcactggctcagggccaggtcctgcagcaccgccagcagcgtgcgccgcgcca
                 ############################################################################################################################################################################################################################################################################################################


                      22210     22220     22230     22240     22250     22260     22270     22280     22290     22300     22310     22320     22330     22340     22350     22360     22370     22380     22390     22400     22410     22420     22430     22440     22450     22460     22470     22480     22490     22500
                 =========+=========+=========+=========+=========+=========+=========+=========+=========+=========+=========+=========+=========+=========+=========+=========+=========+=========+=========+=========+=========+=========+=========+=========+=========+=========+=========+=========+=========+=========+
PRV-WT complete  ggaagctcgtcgccgggcacagcgcgcccgagtacgggtccagcgagagggacatggtgtggttggcgtcgtagagcgcgtcgcggagcttgaagtcgcgcgtctccacgagcgtgcgcacgaagcggtccgccgcctgctcgaccgtgtcgcgcagggcgcgcagcgcgtgccggaagccggcgtggtccgtcaccgtctcgccgatgcgcgcgccggccacgtccgccagcagccggtccacggcggcgcggtacgtgtcctgcatgatcgccttcgggggctcgctgtcgttggggc
PRV-delete UL10  ggaagctcgtcgccgggcacagcgcgcccgagtacgggtccagcgagagggacatggtgtggttggcgtcgtagagcgcgtcgcggagcttgaagtcgcgcgtctccacgagcgtgcgcacgaagcggtccgccgcctgctcgaccgtgtcgcgcagggcgcgcagcgcgtgccggaagccggcgtggtccgtcaccgtctcgccgatgcgcgcgccggccacgtccgccagcagccggtccacggcggcgcggtacgtgtcctgcatgatcgccttcgggggctcgctgtcgttggggc
                 ############################################################################################################################################################################################################################################################################################################


                      22510     22520     22530     22540     22550     22560     22570     22580     22590     22600     22610     22620     22630     22640     22650     22660     22670     22680     22690     22700     22710     22720     22730     22740     22750     22760     22770     22780     22790     22800
                 =========+=========+=========+=========+=========+=========+=========+=========+=========+=========+=========+=========+=========+=========+=========+=========+=========+=========+=========+=========+=========+=========+=========+=========+=========+=========+=========+=========+=========+=========+
PRV-WT complete  gcttgagggccccgtacgaggcgtagttgccgagcacgtcgcagtcgctgtaggcgctgttcatggtcccgaagacgcccatggggctgcgcgtcggcgcgccgaagcgcgggaggcggtggcgcaggcggtgcagcgtcgtgtgcgcgcaggcggggcgcgaggcgcggtcgcagagcgagcaggggacctcggcgtcgaggctgccggccacgtagcgcacggcgtccacgtcgccgcggcccacgaacgtgcccgcgtcgcagcgctccaggtagaacagcacgcgcgccagcagct
PRV-delete UL10  gcttgagggccccgtacgaggcgtagttgccgagcacgtcgcagtcgctgtaggcgctgttcatggtcccgaagacgcccatggggctgcgcgtcggcgcgccgaagcgcgggaggcggtggcgcaggcggtgcagcgtcgtgtgcgcgcaggcggggcgcgaggcgcggtcgcagagcgagcaggggacctcggcgtcgaggctgccggccacgtagcgcacggcgtccacgtcgccgcggcccacgaacgtgcccgcgtcgcagcgctccaggtagaacagcacgcgcgccagcagct
                 ############################################################################################################################################################################################################################################################################################################


                      22810     22820     22830     22840     22850     22860     22870     22880     22890     22900     22910     22920     22930     22940     22950     22960     22970     22980     22990     23000     23010     23020     23030     23040     23050     23060     23070     23080     23090     23100
                 =========+=========+=========+=========+=========+=========+=========+=========+=========+=========+=========+=========+=========+=========+=========+=========+=========+=========+=========+=========+=========+=========+=========+=========+=========+=========+=========+=========+=========+=========+
PRV-WT complete  gcgggcagaagccgcacgccatcaccaggtggtccatggcgaagtcgtggttgcccggggccgtcgcgggctgcgaggccgtgtgcggcagcacgcgcccgtccttgtccgtctgcgggttcccggccaggtagggcgccgccacctggtagaagcggttaaacgaggggccggcgccctccttgccgccgccgtcggcggcgcccgcgtcgtccacctccgtcaggtacagcaccgagttggagctgaacaccagcgcccccacgaggccggccgcgcggctcacgtacgcgccgagcg
PRV-delete UL10  gcgggcagaagccgcacgccatcaccaggtggtccatggcgaagtcgtggttgcccggggccgtcgcgggctgcgaggccgtgtgcggcagcacgcgcccgtccttgtccgtctgcgggttcccggccaggtagggcgccgccacctggtagaagcggttaaacgaggggccggcgccctccttgccgccgccgtcggcggcgcccgcgtcgtccacctccgtcaggtacagcaccgagttggagctgaacaccagcgcccccacgaggccggccgcgcggctcacgtacgcgccgagcg
                 ############################################################################################################################################################################################################################################################################################################


                      23110     23120     23130     23140     23150     23160     23170     23180     23190     23200     23210     23220     23230     23240     23250     23260     23270     23280     23290     23300     23310     23320     23330     23340     23350     23360     23370     23380     23390     23400
                 =========+=========+=========+=========+=========+=========+=========+=========+=========+=========+=========+=========+=========+=========+=========+=========+=========+=========+=========+=========+=========+=========+=========+=========+=========+=========+=========+=========+=========+=========+
PRV-WT complete  ccgcggcctgcgagacgggcgcgcccgcggaggcggccgcgccgccgccgccgtccttggcgcccgcctcggagagcacgggccagtcgtccagcgcggggggctcctcgtcgtacacgccggcggcgacgagcgcctccagcgacagcgccgcgtcggcgctcatgacggacgccatgcggcgctcgaggccgccggcggccgcgtcgccgccgcgccgccgctgctcaaagagcgtgaaggtcacgtcggcgggcagcacggcgctctcgtggttctcgtcgaaggccaggtgcgccg
PRV-delete UL10  ccgcggcctgcgagacgggcgcgcccgcggaggcggccgcgccgccgccgccgtccttggcgcccgcctcggagagcacgggccagtcgtccagcgcggggggctcctcgtcgtacacgccggcggcgacgagcgcctccagcgacagcgccgcgtcggcgctcatgacggacgccatgcggcgctcgaggccgccggcggccgcgtcgccgccgcgccgccgctgctcaaagagcgtgaaggtcacgtcggcgggcagcacggcgctctcgtggttctcgtcgaaggccaggtgcgccg
                 ############################################################################################################################################################################################################################################################################################################


                      23410     23420     23430     23440     23450     23460     23470     23480     23490     23500     23510     23520     23530     23540     23550     23560     23570     23580     23590     23600     23610     23620     23630     23640     23650     23660     23670     23680     23690     23700
                 =========+=========+=========+=========+=========+=========+=========+=========+=========+=========+=========+=========+=========+=========+=========+=========+=========+=========+=========+=========+=========+=========+=========+=========+=========+=========+=========+=========+=========+=========+
PRV-WT complete  cgccgcgcgccaccgcgtccaggttccgcacgcgcgtggccacggccgccgggcccagcacgtagccgtgcagcaggcggcagagcgggccgttgaagaaggggcgcgggtacacaaactcctcgctgatggagcggttcttggcgttgaaggggtcggccacgagccggttgacgtccggcatgaagaggtgcacggggtagagcggcacgcgccacgcctcgtgcccgttgatggacaccgtgcccgcgcccccgtagtgcaggaaggcgttgcacaggtacaccgcctccttgaagc
PRV-delete UL10  cgccgcgcgccaccgcgtccaggttccgcacgcgcgtggccacggccgccgggcccagcacgtagccgtgcagcaggcggcagagcgggccgttgaagaaggggcgcgggtacacaaactcctcgctgatggagcggttcttggcgttgaaggggtcggccacgagccggttgacgtccggcatgaagaggtgcacggggtagagcggcacgcgccacgcctcgtgcccgttgatggacaccgtgcccgcgcccccgtagtgcaggaaggcgttgcacaggtacaccgcctccttgaagc
                 ############################################################################################################################################################################################################################################################################################################


                      23710     23720     23730     23740     23750     23760     23770     23780     23790     23800     23810     23820     23830     23840     23850     23860     23870     23880     23890     23900     23910     23920     23930     23940     23950     23960     23970     23980     23990     24000
                 =========+=========+=========+=========+=========+=========+=========+=========+=========+=========+=========+=========+=========+=========+=========+=========+=========+=========+=========+=========+=========+=========+=========+=========+=========+=========+=========+=========+=========+=========+
PRV-WT complete  cgtcggccaccacgaggtaggccgcgtgcgcgtccgcggccaggccgagccgcgcgcacacctccgcgcccgtcgtctcctcggcgttgtccaccggcgtccggtaggccgagaacccgaagcgggcgcgcgccgcctcgcaggcgcgcgagagggcgggcgcggcgctgctcgggggcacgcagtccccgttgtggaagaagaacacgttggggtggaagtggttgggcgtcagcttcagcgtgagcccgccgccgagcccggtggtccgggcgcccgccaccacggccacgtgcccgg
PRV-delete UL10  cgtcggccaccacgaggtaggccgcgtgcgcgtccgcggccaggccgagccgcgcgcacacctccgcgcccgtcgtctcctcggcgttgtccaccggcgtccggtaggccgagaacccgaagcgggcgcgcgccgcctcgcaggcgcgcgagagggcgggcgcggcgctgctcgggggcacgcagtccccgttgtggaagaagaacacgttggggtggaagtggttgggcgtcagcttcagcgtgagcccgccgccgagcccggtggtccgggcgcccgccaccacggccacgtgcccgg
                 ############################################################################################################################################################################################################################################################################################################


                      24010     24020     24030     24040     24050     24060     24070     24080     24090     24100     24110     24120     24130     24140     24150     24160     24170     24180     24190     24200     24210     24220     24230     24240     24250     24260     24270     24280     24290     24300
                 =========+=========+=========+=========+=========+=========+=========+=========+=========+=========+=========+=========+=========+=========+=========+=========+=========+=========+=========+=========+=========+=========+=========+=========+=========+=========+=========+=========+=========+=========+
PRV-WT complete  cgaagccggcctcgacggtcaggccgcgcaccagcggcgccaccgccgcggcgtcgtcggcgctgcgcgccacgagcagcgccagcaggtcgcggcgcagcgcctcgatcggcgtcacgtacacgtagcccagcggcgcggcgcgcacggtcacggtcttggccgcggcctccatcgtcgcgggtgagacgagacgagagcgggagcgggagcgggagcgggagagagagaaggcgcggcggacggggtcgagtctcggtcccctctccgggcggacgggtccggcggcggggtgaagag
PRV-delete UL10  cgaagccggcctcgacggtcaggccgcgcaccagcggcgccaccgccgcggcgtcgtcggcgctgcgcgccacgagcagcgccagcaggtcgcggcgcagcgcctcgatcggcgtcacgtacacgtagcccagcggcgcggcgcgcacggtcacggtcttggccgcggcctccatcgtcgcgggtgagacgagacgagagcgggagcgggagcgggagcgggagagagagaaggcgcggcggacggggtcgagtctcggtcccctctccgggcggacgggtccggcggcggggtgaagag
                 ############################################################################################################################################################################################################################################################################################################


                      24310     24320     24330     24340     24350     24360     24370     24380     24390     24400     24410     24420     24430     24440     24450     24460     24470     24480     24490     24500     24510     24520     24530     24540     24550     24560     24570     24580     24590     24600
                 =========+=========+=========+=========+=========+=========+=========+=========+=========+=========+=========+=========+=========+=========+=========+=========+=========+=========+=========+=========+=========+=========+=========+=========+=========+=========+=========+=========+=========+=========+
PRV-WT complete  agagacggaggtgtcggcgccgaggggcgaacgcgacgtcggcggcggtggtagagcccggcccgccccggccgggtcttaaagcgtggggccccgccccgccggggcgtggcgccccgccccggagcctatacctcggccggcggcgttcgcacctccgcgtgggacgagcgatggcggcgcggcagggcagctacgtgacgcgcctgagcgagttcaagttcatcgcgccgcggtgcctggacgccccggaccagcgcggcgtgcacgtgggcacgctcgcgcgcgagcccacggtct
PRV-delete UL10  agagacggaggtgtcggcgccgaggggcgaacgcgacgtcggcggcggtggtagagcccggcccgccccggccgggtcttaaagcgtggggccccgccccgccggggcgtggcgccccgccccggagcctatacctcggccggcggcgttcgcacctccgcgtgggacgagcgatggcggcgcggcagggcagctacgtgacgcgcctgagcgagttcaagttcatcgcgccgcggtgcctggacgccccggaccagcgcggcgtgcacgtgggcacgctcgcgcgcgagcccacggtct
                 ############################################################################################################################################################################################################################################################################################################


                      24610     24620     24630     24640     24650     24660     24670     24680     24690     24700     24710     24720     24730     24740     24750     24760     24770     24780     24790     24800     24810     24820     24830     24840     24850     24860     24870     24880     24890     24900
                 =========+=========+=========+=========+=========+=========+=========+=========+=========+=========+=========+=========+=========+=========+=========+=========+=========+=========+=========+=========+=========+=========+=========+=========+=========+=========+=========+=========+=========+=========+
PRV-WT complete  actgcggcggcgccacgcggcccatcctccgcggggagcccttctggccgcggcgcgtcgccgcgtgggacggggcccccgagcggcccgtgagcccgcgcttcgagcgcttccacgtgtacgacatcgtcgagagcaccgagtacgccagcgcggacccgcgcttccccaacggcacggtggtcacgctgctggggctcagcgcctgcggcaagcgcgtggccgtgcacgtgtacggcgtgcgccactacttcttcctgggcaaggccgaggccgacgccgccctcggcgtgacgagcg
PRV-delete UL10  actgcggcggcgccacgcggcccatcctccgcggggagcccttctggccgcggcgcgtcgccgcgtgggacggggcccccgagcggcccgtgagcccgcgcttcgagcgcttccacgtgtacgacatcgtcgagagcaccgagtacgccagcgcggacccgcgcttccccaacggcacggtggtcacgctgctggggctcagcgcctgcggcaagcgcgtggccgtgcacgtgtacggcgtgcgccactacttcttcctgggcaaggccgaggccgacgccgccctcggcgtgacgagcg
                 ############################################################################################################################################################################################################################################################################################################


                      24910     24920     24930     24940     24950     24960     24970     24980     24990     25000     25010     25020     25030     25040     25050     25060     25070     25080     25090     25100     25110     25120     25130     25140     25150     25160     25170     25180     25190     25200
                 =========+=========+=========+=========+=========+=========+=========+=========+=========+=========+=========+=========+=========+=========+=========+=========+=========+=========+=========+=========+=========+=========+=========+=========+=========+=========+=========+=========+=========+=========+
PRV-WT complete  ccgagcagctcgcgcgcgcgctctcggcggcggcggcgcgcggcccgcgcctcgggcccgccgacgtggacgcgcgcgtcgtcgacgccgcgcccgtctactactacgacgccccccggcggcccttctaccgcgtctcgagcaacagcgggcgcctggtcgcgcacctccgcgagacggtctgcgccggcctggtgacgcacgaggccggcgtggacgcgaccacgcgcctgctgctcgaccacgacctgcccagcttcggctggtaccgcctgcggcccggccccgcgggggagcgcg
PRV-delete UL10  ccgagcagctcgcgcgcgcgctctcggcggcggcggcgcgcggcccgcgcctcgggcccgccgacgtggacgcgcgcgtcgtcgacgccgcgcccgtctactactacgacgccccccggcggcccttctaccgcgtctcgagcaacagcgggcgcctggtcgcgcacctccgcgagacggtctgcgccggcctggtgacgcacgaggccggcgtggacgcgaccacgcgcctgctgctcgaccacgacctgcccagcttcggctggtaccgcctgcggcccggccccgcgggggagcgcg
                 ############################################################################################################################################################################################################################################################################################################


                      25210     25220     25230     25240     25250     25260     25270     25280     25290     25300     25310     25320     25330     25340     25350     25360     25370     25380     25390     25400     25410     25420     25430     25440     25450     25460     25470     25480     25490     25500
                 =========+=========+=========+=========+=========+=========+=========+=========+=========+=========+=========+=========+=========+=========+=========+=========+=========+=========+=========+=========+=========+=========+=========+=========+=========+=========+=========+=========+=========+=========+
PRV-WT complete  tggtgctgcggcaccagcgcctcacgtccagcgacgtggaggtcaactgcacgcccctcaacctggcccgcgacgaggacggcccgtggcccgactacaagctcctgtgcttcgacatcgagtgcaaggccggcgacgacgcggccttccccgccgccgagaaccccgaggacctggtgatccagatctcgtgcctgctgtactcgctcgccacccagcggctggagcacacgctgctcttctcgctgggctcgtgcgactcggacgaccccgcggtgacggtcctcgagttcgacagcg
PRV-delete UL10  tggtgctgcggcaccagcgcctcacgtccagcgacgtggaggtcaactgcacgcccctcaacctggcccgcgacgaggacggcccgtggcccgactacaagctcctgtgcttcgacatcgagtgcaaggccggcgacgacgcggccttccccgccgccgagaaccccgaggacctggtgatccagatctcgtgcctgctgtactcgctcgccacccagcggctggagcacacgctgctcttctcgctgggctcgtgcgactcggacgaccccgcggtgacggtcctcgagttcgacagcg
                 ############################################################################################################################################################################################################################################################################################################


                      25510     25520     25530     25540     25550     25560     25570     25580     25590     25600     25610     25620     25630     25640     25650     25660     25670     25680     25690     25700     25710     25720     25730     25740     25750     25760     25770     25780     25790     25800
                 =========+=========+=========+=========+=========+=========+=========+=========+=========+=========+=========+=========+=========+=========+=========+=========+=========+=========+=========+=========+=========+=========+=========+=========+=========+=========+=========+=========+=========+=========+
PRV-WT complete  agttcgagctgctgctcgccttcgtgaccttcctgaagcagtacgcgcccgagttcgccacggggtacaacatcatcaactttgactgggccttcgtgcacaccaagctcacggccgtctacgggctggccctcgacggctacgggcgcttcaaccgcggcggccagttccgcgtctacgacgcgggccagaacagcttccagaagcgcagcaaggtcaagatcaacggcctcgtctccctggacatgtacgccgtcgccgccgacaagctgaagctgccgagctacaagctcaacgccg
PRV-delete UL10  agttcgagctgctgctcgccttcgtgaccttcctgaagcagtacgcgcccgagttcgccacggggtacaacatcatcaactttgactgggccttcgtgcacaccaagctcacggccgtctacgggctggccctcgacggctacgggcgcttcaaccgcggcggccagttccgcgtctacgacgcgggccagaacagcttccagaagcgcagcaaggtcaagatcaacggcctcgtctccctggacatgtacgccgtcgccgccgacaagctgaagctgccgagctacaagctcaacgccg
                 ############################################################################################################################################################################################################################################################################################################


                      25810     25820     25830     25840     25850     25860     25870     25880     25890     25900     25910     25920     25930     25940     25950     25960     25970     25980     25990     26000     26010     26020     26030     26040     26050     26060     26070     26080     26090     26100
                 =========+=========+=========+=========+=========+=========+=========+=========+=========+=========+=========+=========+=========+=========+=========+=========+=========+=========+=========+=========+=========+=========+=========+=========+=========+=========+=========+=========+=========+=========+
PRV-WT complete  tggccgaggaggcgctcggcgagcgcaagctggacctggactacaaggacatcccgcggtactacgccgcgggcccgcgcgagcgcggcgtcatcgggcgctactgcgtgcaggactcggcgctcgtgggcaagctcttcttcaagttcctgccgcacctcgagctctcggccgtggcgcggctcgccaacatcacgctcgcgcgcgccatctacgacggccagcagatccgcgtgttcacgtgcctgctgaagctcgcgggctcgcgcggcttcgtgctgcccgacaagcgccgcgaca
PRV-delete UL10  tggccgaggaggcgctcggcgagcgcaagctggacctggactacaaggacatcccgcggtactacgccgcgggcccgcgcgagcgcggcgtcatcgggcgctactgcgtgcaggactcggcgctcgtgggcaagctcttcttcaagttcctgccgcacctcgagctctcggccgtggcgcggctcgccaacatcacgctcgcgcgcgccatctacgacggccagcagatccgcgtgttcacgtgcctgctgaagctcgcgggctcgcgcggcttcgtgctgcccgacaagcgccgcgaca
                 ############################################################################################################################################################################################################################################################################################################


                      26110     26120     26130     26140     26150     26160     26170     26180     26190     26200     26210     26220     26230     26240     26250     26260     26270     26280     26290     26300     26310     26320     26330     26340     26350     26360     26370     26380     26390     26400
                 =========+=========+=========+=========+=========+=========+=========+=========+=========+=========+=========+=========+=========+=========+=========+=========+=========+=========+=========+=========+=========+=========+=========+=========+=========+=========+=========+=========+=========+=========+
PRV-WT complete  tcgcggacgaggacgacggcggcggctaccagggcgccaaggtgctcgagcccgactcgggcttccacgtggacccggtgctcgtgctggactttgccagcctgtacccgagcatcatccaggcgcacaacctgtgcttcacgacgctcgcgctcgcgcgcccggcgggcctgcgcgaggacgagttcagcgccttcgaggtcaacggggagcggctctactttgtgcacgcgggggtgcgcgagagcctgctctcgatcctgctgcgcgactggttggccatgcgcaaggccatccgcg
PRV-delete UL10  tcgcggacgaggacgacggcggcggctaccagggcgccaaggtgctcgagcccgactcgggcttccacgtggacccggtgctcgtgctggactttgccagcctgtacccgagcatcatccaggcgcacaacctgtgcttcacgacgctcgcgctcgcgcgcccggcgggcctgcgcgaggacgagttcagcgccttcgaggtcaacggggagcggctctactttgtgcacgcgggggtgcgcgagagcctgctctcgatcctgctgcgcgactggttggccatgcgcaaggccatccgcg
                 ############################################################################################################################################################################################################################################################################################################


                      26410     26420     26430     26440     26450     26460     26470     26480     26490     26500     26510     26520     26530     26540     26550     26560     26570     26580     26590     26600     26610     26620     26630     26640     26650     26660     26670     26680     26690     26700
                 =========+=========+=========+=========+=========+=========+=========+=========+=========+=========+=========+=========+=========+=========+=========+=========+=========+=========+=========+=========+=========+=========+=========+=========+=========+=========+=========+=========+=========+=========+
PRV-WT complete  cgcgcatcccgacgagcgcgcccgaggaggccgtgctgctggacaagcagcaggcggccatcaaggtggtgtgcaactcggtgtacgggttcacgggcgtggccaacgggctgctcccctgcctccccgtggcggccaccgtgacgaccatcggccgcgacatgctggtggccacgcgcgactacgtgcagacgcgctgggccacgcgcgagctgctcgagcgcgacctgcccgcgcgcccgcccgcgggcgagtacgccgtgcgcgtggtctacggggacacggactcggtcttcatcc
PRV-delete UL10  cgcgcatcccgacgagcgcgcccgaggaggccgtgctgctggacaagcagcaggcggccatcaaggtggtgtgcaactcggtgtacgggttcacgggcgtggccaacgggctgctcccctgcctccccgtggcggccaccgtgacgaccatcggccgcgacatgctggtggccacgcgcgactacgtgcagacgcgctgggccacgcgcgagctgctcgagcgcgacctgcccgcgcgcccgcccgcgggcgagtacgccgtgcgcgtggtctacggggacacggactcggtcttcatcc
                 ############################################################################################################################################################################################################################################################################################################


                      26710     26720     26730     26740     26750     26760     26770     26780     26790     26800     26810     26820     26830     26840     26850     26860     26870     26880     26890     26900     26910     26920     26930     26940     26950     26960     26970     26980     26990     27000
                 =========+=========+=========+=========+=========+=========+=========+=========+=========+=========+=========+=========+=========+=========+=========+=========+=========+=========+=========+=========+=========+=========+=========+=========+=========+=========+=========+=========+=========+=========+
PRV-WT complete  gcttctcgggcatcgcgtacgacgacgtgtgcgagctcggggagctcatggccgcgcgcatcacgcgcgacctcttccgcccccccatcaagctcgagtgcgagaagaccttccggcgcctgctgctcatcacgaagaagaagtacatcggcgtcatcaacggcggcaagatgctcatgaagggcgtcgacctggtgcgcaagaacaactgcgccttcatcaacgcgtacgcgcgccgcctggtggacctgctctttggcgacgaggccgtctcggcggccgcggccgcgatcgcgcgcg
PRV-delete UL10  gcttctcgggcatcgcgtacgacgacgtgtgcgagctcggggagctcatggccgcgcgcatcacgcgcgacctcttccgcccccccatcaagctcgagtgcgagaagaccttccggcgcctgctgctcatcacgaagaagaagtacatcggcgtcatcaacggcggcaagatgctcatgaagggcgtcgacctggtgcgcaagaacaactgcgccttcatcaacgcgtacgcgcgccgcctggtggacctgctctttggcgacgaggccgtctcggcggccgcggccgcgatcgcgcgcg
                 ############################################################################################################################################################################################################################################################################################################


                      27010     27020     27030     27040     27050     27060     27070     27080     27090     27100     27110     27120     27130     27140     27150     27160     27170     27180     27190     27200     27210     27220     27230     27240     27250     27260     27270     27280     27290     27300
                 =========+=========+=========+=========+=========+=========+=========+=========+=========+=========+=========+=========+=========+=========+=========+=========+=========+=========+=========+=========+=========+=========+=========+=========+=========+=========+=========+=========+=========+=========+
PRV-WT complete  cgcccgcggcgcgctggctggagcgccccctgccgcgcggcttcgccgccttcgggcgcgtgctggccgaggcgcacgcgcgcgtcgccggcggcgccggcctggacgtggccgacttcgtcatgacggccgagctcagccgccccccggacgcgtacagcaacacgcgcctgccgcacctcaccgtctaccacaagctggccatgcgccacggggaggtgccgagcgtcaaggagcgcgtgtcctacgtcatcatcgcgcccacccccgaggccgagcgcgacgcgcgggccgtgcgcc
PRV-delete UL10  cgcccgcggcgcgctggctggagcgccccctgccgcgcggcttcgccgccttcgggcgcgtgctggccgaggcgcacgcgcgcgtcgccggcggcgccggcctggacgtggccgacttcgtcatgacggccgagctcagccgccccccggacgcgtacagcaacacgcgcctgccgcacctcaccgtctaccacaagctggccatgcgccacggggaggtgccgagcgtcaaggagcgcgtgtcctacgtcatcatcgcgcccacccccgaggccgagcgcgacgcgcgggccgtgcgcc
                 ############################################################################################################################################################################################################################################################################################################


                      27310     27320     27330     27340     27350     27360     27370     27380     27390     27400     27410     27420     27430     27440     27450     27460     27470     27480     27490     27500     27510     27520     27530     27540     27550     27560     27570     27580     27590     27600
                 =========+=========+=========+=========+=========+=========+=========+=========+=========+=========+=========+=========+=========+=========+=========+=========+=========+=========+=========+=========+=========+=========+=========+=========+=========+=========+=========+=========+=========+=========+
PRV-WT complete  cgggcctcgccccggggaagctgctcgtcagcgacctggccgaggaccccgcctacgtggtcgcgcacggcgtgcccctcaacaccgagtactacttctcccacctgctgagcacgctcagcgtcaccttcaaggccctctttggaaatgacaccaagatcacggagaggttgctcaagcgctttattccggaaaccgccccgggggacgcgcccttcgagcacgaggccttcgcgccgctcacgggcgaggggggcgaaagtcttcaaacgctgcgtacaatcttttgtactccaacag
PRV-delete UL10  cgggcctcgccccggggaagctgctcgtcagcgacctggccgaggaccccgcctacgtggtcgcgcacggcgtgcccctcaacaccgagtactacttctcccacctgctgagcacgctcagcgtcaccttcaaggccctctttggaaatgacaccaagatcacggagaggttgctcaagcgctttattccggaaaccgccccgggggacgcgcccttcgagcacgaggccttcgcgccgctcacgggcgaggggggcgaaagtcttcaaacgctgcgtacaatcttttgtactccaacag
                 ############################################################################################################################################################################################################################################################################################################


                      27610     27620     27630     27640     27650     27660     27670     27680     27690     27700     27710     27720     27730     27740     27750     27760     27770     27780     27790     27800     27810     27820     27830     27840     27850     27860     27870     27880     27890     27900
                 =========+=========+=========+=========+=========+=========+=========+=========+=========+=========+=========+=========+=========+=========+=========+=========+=========+=========+=========+=========+=========+=========+=========+=========+=========+=========+=========+=========+=========+=========+
PRV-WT complete  cagctccccgtcgaagctgatgtcccgcatcttgcaataaatgtcgctcgcgctgacggcgggcacgtcgtccgcgggtctcccgtctcccttctttcgcaccaccaacacgaacatggtctgccacacgtgcgcgacgatgcggtggccggcgcaggccccgagcaggcggtccagcacgcggtggtgcacgtgcaccgacttctcggggaacaccacgtacatcatgagcgcgccctgcgcgtccgggtcctcgaagaagagcacgcgcacgtcgcgcaggcccccgtcgcgcagcac
PRV-delete UL10  cagctccccgtcgaagctgatgtcccgcatcttgcaataaatgtcgctcgcgctgacggcgggcacgtcgtccgcgggtctcccgtctcccttctttcgcaccaccaacacgaacatggtctgccacacgtgcgcgacgatgcggtggccggcgcaggccccgagcaggcggtccagcacgcggtggtgcacgtgcaccgacttctcggggaacaccacgtacatcatgagcgcgccctgcgcgtccgggtcctcgaagaagagcacgcgcacgtcgcgcaggcccccgtcgcgcagcac
                 ############################################################################################################################################################################################################################################################################################################


                      27910     27920     27930     27940     27950     27960     27970     27980     27990     28000     28010     28020     28030     28040     28050     28060     28070     28080     28090     28100     28110     28120     28130     28140     28150     28160     28170     28180     28190     28200
                 =========+=========+=========+=========+=========+=========+=========+=========+=========+=========+=========+=========+=========+=========+=========+=========+=========+=========+=========+=========+=========+=========+=========+=========+=========+=========+=========+=========+=========+=========+
PRV-WT complete  gtagtaggcgaaaaagagctccgggtgcgcgagcacctcctcgagcgcgcgctccgaggggtcccgcgcggccaccgaggccaggaacacgcggtactcgtagatgctgttgagctgctgcacgtaggccagcacgagcgccgcgcggtcgctgcgcccgagccgcggctcggcggcggcgcacgtcgggcagcagccgccgaggcccaggtagtagcccatgcccgagagcgacaggcagttgtcggccaccgcctggccgaggtcgaagggcagcgtcacgggggccgtcttgatgag
PRV-delete UL10  gtagtaggcgaaaaagagctccgggtgcgcgagcacctcctcgagcgcgcgctccgaggggtcccgcgcggccaccgaggccaggaacacgcggtactcgtagatgctgttgagctgctgcacgtaggccagcacgagcgccgcgcggtcgctgcgcccgagccgcggctcggcggcggcgcacgtcgggcagcagccgccgaggcccaggtagtagcccatgcccgagagcgacaggcagttgtcggccaccgcctggccgaggtcgaagggcagcgtcacgggggccgtcttgatgag
                 ############################################################################################################################################################################################################################################################################################################


                      28210     28220     28230     28240     28250     28260     28270     28280     28290     28300     28310     28320     28330     28340     28350     28360     28370     28380     28390     28400     28410     28420     28430     28440     28450     28460     28470     28480     28490     28500
                 =========+=========+=========+=========+=========+=========+=========+=========+=========+=========+=========+=========+=========+=========+=========+=========+=========+=========+=========+=========+=========+=========+=========+=========+=========+=========+=========+=========+=========+=========+
PRV-WT complete  cgggttcgagaggccgcgcacggtggccacctcgtccgagggctgcgcggccgcgtaggcaaagtagggcgagtagcgatcgcggaccgcgcgcgccagcgtcttgcgccgcgcggccgccgacttgcgccgcaggagtcgccgccgctcaaacatagctgccgagcgtgtgctcgagggagacgagcgcgaggccgtggcgccgcagcagggcgtgtgcgtcgcgcaggaaggcggcgcgggcagtgggcttcggcgggaacagctggtacgctttaaatacgtacgccagggtccaca
PRV-delete UL10  cgggttcgagaggccgcgcacggtggccacctcgtccgagggctgcgcggccgcgtaggcaaagtagggcgagtagcgatcgcggaccgcgcgcgccagcgtcttgcgccgcgcggccgccgacttgcgccgcaggagtcgccgccgctcaaacatagctgccgagcgtgtgctcgagggagacgagcgcgaggccgtggcgccgcagcagggcgtgtgcgtcgcgcaggaaggcggcgcgggcagtgggcttcggcgggaacagctggtacgctttaaatacgtacgccagggtccaca
                 ############################################################################################################################################################################################################################################################################################################


                      28510     28520     28530     28540     28550     28560     28570     28580     28590     28600     28610     28620     28630     28640     28650     28660     28670     28680     28690     28700     28710     28720     28730     28740     28750     28760     28770     28780     28790     28800
                 =========+=========+=========+=========+=========+=========+=========+=========+=========+=========+=========+=========+=========+=========+=========+=========+=========+=========+=========+=========+=========+=========+=========+=========+=========+=========+=========+=========+=========+=========+
PRV-WT complete  gctccccgcacacgcgcccctcgaagcggttctcggcggcgatctgcgccttgcgcaacgcgtcgccgcgctcaaagagcacgcgcgggtccgcctggcgctcggccacggcgcacagggggtcgcagaagaggtgcttgtagacggcgcgcgggcccgcggcgcgcatcagcgccaggaagcgcgcgtccgccgcgaagccctcgagcacgggctcgatgcagtcaaagaggcccgcgttgttctccgagtagctgagcacgcggcgccggaagcggcgcagcgccagccagtgcgcgc
PRV-delete UL10  gctccccgcacacgcgcccctcgaagcggttctcggcggcgatctgcgccttgcgcaacgcgtcgccgcgctcaaagagcacgcgcgggtccgcctggcgctcggccacggcgcacagggggtcgcagaagaggtgcttgtagacggcgcgcgggcccgcggcgcgcatcagcgccaggaagcgcgcgtccgccgcgaagccctcgagcacgggctcgatgcagtcaaagaggcccgcgttgttctccgagtagctgagcacgcggcgccggaagcggcgcagcgccagccagtgcgcgc
                 ############################################################################################################################################################################################################################################################################################################


                      28810     28820     28830     28840     28850     28860     28870     28880     28890     28900     28910     28920     28930     28940     28950     28960     28970     28980     28990     29000     29010     29020     29030     29040     29050     29060     29070     29080     29090     29100
                 =========+=========+=========+=========+=========+=========+=========+=========+=========+=========+=========+=========+=========+=========+=========+=========+=========+=========+=========+=========+=========+=========+=========+=========+=========+=========+=========+=========+=========+=========+
PRV-WT complete  gcgtgagcagcatgttgcagagcaggcactcgccgtgcgtgcggttgcggccgcgcacgtgcttcagcgccagcgccagcacgggccccagcgtggcgtccaccgcgcggtagcgcgcgtacgccgcggcggtgttgcgggccaggaagccctcgtgctccgcccagtactcgtccacggcgcggtgccggcgctcctcggcggcgtccgtctgcgcgttgggctcccccgtggccacgttgcccagcagcagcatggcgaggtcggcgtagccgggcccgccggcgtccgcgtcctcgg
PRV-delete UL10  gcgtgagcagcatgttgcagagcaggcactcgccgtgcgtgcggttgcggccgcgcacgtgcttcagcgccagcgccagcacgggccccagcgtggcgtccaccgcgcggtagcgcgcgtacgccgcggcggtgttgcgggccaggaagccctcgtgctccgcccagtactcgtccacggcgcggtgccggcgctcctcggcggcgtccgtctgcgcgttgggctcccccgtggccacgttgcccagcagcagcatggcgaggtcggcgtagccgggcccgccggcgtccgcgtcctcgg
                 ############################################################################################################################################################################################################################################################################################################


                      29110     29120     29130     29140     29150     29160     29170     29180     29190     29200     29210     29220     29230     29240     29250     29260     29270     29280     29290     29300     29310     29320     29330     29340     29350     29360     29370     29380     29390     29400
                 =========+=========+=========+=========+=========+=========+=========+=========+=========+=========+=========+=========+=========+=========+=========+=========+=========+=========+=========+=========+=========+=========+=========+=========+=========+=========+=========+=========+=========+=========+
PRV-WT complete  ccccgcccccgcccccggcggcggcgacggcggcgcgctcgtacgcgtcgtcctcgcgctccgccgccgcgcagcacccggcgcacgcctcgtcgcccgtgtggtccttccagccccccaggctcggcggcgcgcgcgcgtccgccacggtgaaaaacatcgcgcgcgtcgcggcctggaccagaaaggactggttcgagaagcggatcttgtcgccgacggtgcggaagcagccgtgcaggaaaaagtgccgctgcacgtccacgagcgtgagcacgcgcgtggcgaaggcgcgctgca
PRV-delete UL10  ccccgcccccgcccccggcggcggcgacggcggcgcgctcgtacgcgtcgtcctcgcgctccgccgccgcgcagcacccggcgcacgcctcgtcgcccgtgtggtccttccagccccccaggctcggcggcgcgcgcgcgtccgccacggtgaaaaacatcgcgcgcgtcgcggcctggaccagaaaggactggttcgagaagcggatcttgtcgccgacggtgcggaagcagccgtgcaggaaaaagtgccgctgcacgtccacgagcgtgagcacgcgcgtggcgaaggcgcgctgca
                 ############################################################################################################################################################################################################################################################################################################


                      29410     29420     29430     29440     29450     29460     29470     29480     29490     29500     29510     29520     29530     29540     29550     29560     29570     29580     29590     29600     29610     29620     29630     29640     29650     29660     29670     29680     29690     29700
                 =========+=========+=========+=========+=========+=========+=========+=========+=========+=========+=========+=========+=========+=========+=========+=========+=========+=========+=========+=========+=========+=========+=========+=========+=========+=========+=========+=========+=========+=========+
PRV-WT complete  gcgacgcgcgcccgcgaaagtgcttgtccatcacgtacacgaactcgaaggcggccacgaagacggccgtggcgcacggggggcaggccagggccttggcgcacagcgccgcgtagtccgccagccagggcggggccagcgagcgctcgcggcggtacacgtcgatcgtgcgacacacgcggcagggctcgtccagcgccagcggcgccgcggcggcctccacgaagcccgcgggcgagtcctcctcgtccagcatcacgtgggcggcgaagatcagctcgcggaagagggcgtcgttct
PRV-delete UL10  gcgacgcgcgcccgcgaaagtgcttgtccatcacgtacacgaactcgaaggcggccacgaagacggccgtggcgcacggggggcaggccagggccttggcgcacagcgccgcgtagtccgccagccagggcggggccagcgagcgctcgcggcggtacacgtcgatcgtgcgacacacgcggcagggctcgtccagcgccagcggcgccgcggcggcctccacgaagcccgcgggcgagtcctcctcgtccagcatcacgtgggcggcgaagatcagctcgcggaagagggcgtcgttct
                 ############################################################################################################################################################################################################################################################################################################


                      29710     29720     29730     29740     29750     29760     29770     29780     29790     29800     29810     29820     29830     29840     29850     29860     29870     29880     29890     29900     29910     29920     29930     29940     29950     29960     29970     29980     29990     30000
                 =========+=========+=========+=========+=========+=========+=========+=========+=========+=========+=========+=========+=========+=========+=========+=========+=========+=========+=========+=========+=========+=========+=========+=========+=========+=========+=========+=========+=========+=========+
PRV-WT complete  tggtcagcagcgcggggtcgaaggccgtgtacggcgcctcgaaggcgtcctcgctccagcccatggcgtgcgacggcggcggcagcggcggcggcgcggtcgatctgcgcgcggcgatcccggaggccgcgctgcgcgacttcgacgtggactttctcgaggccaactacctgcccgcgcgggtgcgcgtctggttcgaggacgtgatgccgcgcgagctggaagtgatcctccccacgacggacgccaagctaaattacctggcccacacgcagcgcctggcggcggcgacgagcgagc
PRV-delete UL10  tggtcagcagcgcggggtcgaaggccgtgtacggcgcctcgaaggcgtcctcgctccagcccatggcgtgcgacggcggcggcagcggcggcggcgcggtcgatctgcgcgcggcgatcccggaggccgcgctgcgcgacttcgacgtggactttctcgaggccaactacctgcccgcgcgggtgcgcgtctggttcgaggacgtgatgccgcgcgagctggaagtgatcctccccacgacggacgccaagctaaattacctggcccacacgcagcgcctggcggcggcgacgagcgagc
                 ############################################################################################################################################################################################################################################################################################################


                      30010     30020     30030     30040     30050     30060     30070     30080     30090     30100     30110     30120     30130     30140     30150     30160     30170     30180     30190     30200     30210     30220     30230     30240     30250     30260     30270     30280     30290     30300
                 =========+=========+=========+=========+=========+=========+=========+=========+=========+=========+=========+=========+=========+=========+=========+=========+=========+=========+=========+=========+=========+=========+=========+=========+=========+=========+=========+=========+=========+=========+
PRV-WT complete  gcgactgtgcccacggggaggcgctgcgggcgcggcgcgatcgcttcgccgcggccgtcaacaagtttctggacctgcaccaggtcctgcgcgacggggtggagctgggcgccctataaagtccgccgcccggccccgggagctccaccagccccaccaccaccgccactgccaccgcagcccgcccgcccgccgggaagaccgccgccgtcgccgcgtcgacgtcgacgtcttagccgcgccgctctccctcgccctcccggcccacggcgacgacaacgacgacgaccatgagcggca
PRV-delete UL10  gcgactgtgcccacggggaggcgctgcgggcgcggcgcgatcgcttcgccgcggccgtcaacaagtttctggacctgcaccaggtcctgcgcgacggggtggagctgggcgccctataaagtccgccgcccggccccgggagctccaccagccccaccaccaccgccactgccaccgcagcccgcccgcccgccgggaagaccgccgccgtcgccgcgtcgacgtcgacgtcttagccgcgccgctctccctcgccctcccggcccacggcgacgacaacgacgacgaccatgagcggca
                 ############################################################################################################################################################################################################################################################################################################


                      30310     30320     30330     30340     30350     30360     30370     30380     30390     30400     30410     30420     30430     30440     30450     30460     30470     30480     30490     30500     30510     30520     30530     30540     30550     30560     30570     30580     30590     30600
                 =========+=========+=========+=========+=========+=========+=========+=========+=========+=========+=========+=========+=========+=========+=========+=========+=========+=========+=========+=========+=========+=========+=========+=========+=========+=========+=========+=========+=========+=========+
PRV-WT complete  ccctgatccagcgcctgaagctcatcctctccggggggaacctgcgctgcagcgacggcgagacggcctgcgaccccgagcggcccccgacgcggtgcgtcttccaggtccacgggcaggacggctccaacgacaccttcccgctggagtacgtgctgcgcctcatgcgcagctgggcccacgtcccctgcgacccctacgtgcgcgtgcagaacacgggcgtgtcggtgctcttccagggcttcttcttccggcccgccgacgcccccctggccgcgatcacggccgagcacaacaacg
PRV-delete UL10  ccctgatccagcgcctgaagctcatcctctccggggggaacctgcgctgcagcgacggcgagacggcctgcgaccccgagcggcccccgacgcggtgcgtcttccaggtccacgggcaggacggctccaacgacaccttcccgctggagtacgtgctgcgcctcatgcgcagctgggcccacgtcccctgcgacccctacgtgcgcgtgcagaacacgggcgtgtcggtgctcttccagggcttcttcttccggcccgccgacgcccccctggccgcgatcacggccgagcacaacaacg
                 ############################################################################################################################################################################################################################################################################################################


                      30610     30620     30630     30640     30650     30660     30670     30680     30690     30700     30710     30720     30730     30740     30750     30760     30770     30780     30790     30800     30810     30820     30830     30840     30850     30860     30870     30880     30890     30900
                 =========+=========+=========+=========+=========+=========+=========+=========+=========+=========+=========+=========+=========+=========+=========+=========+=========+=========+=========+=========+=========+=========+=========+=========+=========+=========+=========+=========+=========+=========+
PRV-WT complete  tgatcttggcctcgacgcacagcaccggcatgagcctctcggcgctcgacgacatcaagcgcgccgggggcgtggacatgcggccgctgcgcgccatgatgtcggtgagctgcttcgtgcgcatgccgcgcgtgcagctctcgttccgcttcatgggccccgacgacgcctcgcagatgcagcgcctcctggaccgcgccgagctgcggcagcgctcggtctcccgccccggcggtggcgacgacggctgctcgggcgagggcccctcgccccgcgcgcccatccgcccacccgtcctct
PRV-delete UL10  tgatcttggcctcgacgcacagcaccggcatgagcctctcggcgctcgacgacatcaagcgcgccgggggcgtggacatgcggccgctgcgcgccatgatgtcggtgagctgcttcgtgcgcatgccgcgcgtgcagctctcgttccgcttcatgggccccgacgacgcctcgcagatgcagcgcctcctggaccgcgccgagctgcggcagcgctcggtctcccgccccggcggtggcgacgacggctgctcgggcgagggcccctcgccccgcgcgcccatccgcccacccgtcctct
                 ############################################################################################################################################################################################################################################################################################################


                      30910     30920     30930     30940     30950     30960     30970     30980     30990     31000     31010     31020     31030     31040     31050     31060     31070     31080     31090     31100     31110     31120     31130     31140     31150     31160     31170     31180     31190     31200
                 =========+=========+=========+=========+=========+=========+=========+=========+=========+=========+=========+=========+=========+=========+=========+=========+=========+=========+=========+=========+=========+=========+=========+=========+=========+=========+=========+=========+=========+=========+
PRV-WT complete  cgcccgtgcccggacacgcggccgccgccctcgtgggccaggccgcgtaccctccgcccacgcggttcccggcctcgctgctgcacgccctcctggggctgcggcgcctcgccggctacgccgtggcgtgcgtcaccggcgctctcgcgatagtcatcatcctgaacatgcgttaaaggcggccgcgcgcgcgggggcgcgcacagacgcgcgctccccgccgagccatcatgtccttcgacccgaacaatccccggacgatcaccgcgcagacgctcgagggcgccctgcccgtggaca
PRV-delete UL10  cgcccgtgcccggacacgcggccgccgccctcgtgggccaggccgcgtaccctccgcccacgcggttcccggcctcgctgctgcacgccctcctggggctgcggcgcctcgccggctacgccgtggcgtgcgtcaccggcgctctcgcgatagtcatcatcctgaacatgcgttaaaggcggccgcgcgcgcgggggcgcgcacagacgcgcgctccccgccgagccatcatgtccttcgacccgaacaatccccggacgatcaccgcgcagacgctcgagggcgccctgcccgtggaca
                 ############################################################################################################################################################################################################################################################################################################


                      31210     31220     31230     31240     31250     31260     31270     31280     31290     31300     31310     31320     31330     31340     31350     31360     31370     31380     31390     31400     31410     31420     31430     31440     31450     31460     31470     31480     31490     31500
                 =========+=========+=========+=========+=========+=========+=========+=========+=========+=========+=========+=========+=========+=========+=========+=========+=========+=========+=========+=========+=========+=========+=========+=========+=========+=========+=========+=========+=========+=========+
PRV-WT complete  ttctgctccggctcaaccgcgcgacggggctgcatatggatgcggccgaggcgcacgcgatcgtggaggacgcccgccggaccctgtttatcggcacctccctggccctcgtgaacctgcggcacgcgcacgacaagcacctcgtggagcgccagcccatgttcgccaccagcgactacagcagctgggccaggcccacggtcggcctgaagcggaccttctgccctcgcccctcgccctagccccgcgcgatcaataaagcgagccgcgttgcaccgtcctctcgtctctcgtgtggtg
PRV-delete UL10  ttctgctccggctcaaccgcgcgacggggctgcatatggatgcggccgaggcgcacgcgatcgtggaggacgcccgccggaccctgtttatcggcacctccctggccctcgtgaacctgcggcacgcgcacgacaagcacctcgtggagcgccagcccatgttcgccaccagcgactacagcagctgggccaggcccacggtcggcctgaagcggaccttctgccctcgcccctcgccctagccccgcgcgatcaataaagcgagccgcgttgcaccgtcctctcgtctctcgtgtggtg
                 ############################################################################################################################################################################################################################################################################################################


                      31510     31520     31530     31540     31550     31560     31570     31580     31590     31600     31610     31620     31630     31640     31650     31660     31670     31680     31690     31700     31710     31720     31730     31740     31750     31760     31770     31780     31790     31800
                 =========+=========+=========+=========+=========+=========+=========+=========+=========+=========+=========+=========+=========+=========+=========+=========+=========+=========+=========+=========+=========+=========+=========+=========+=========+=========+=========+=========+=========+=========+
PRV-WT complete  tctgggtggggtttcccccgagatcccccgatctccccgccgggtctttgggtcccccaccgagttccccaccgagttccccaccgagttccccaccgagttccccaaatgcccaccaccaccaagtctttgggtcccacaacccccaagttttggggtctcgggtccgagtcccccgagtcccccgagtcccccgagtcccccgagtcccccgagtcccccgagtcccccgagtcccccgagtcccccgagtccccgagtcccccgagtcccccgagtcccccgagtccccgagtcccc
PRV-delete UL10  tctgggtggggtttcccccgagatcccccgatctccccgccgggtctttgggtcccccaccgagttccccaccgagttccccaccgagttccccaccgagttccccaaatgcccaccaccaccaagtctttgggtcccacaacccccaagttttggggtctcgggtccgagtcccccgagtcccccgagtcccccgagtcccccgagtcccccgagtcccccgagtcccccgagtcccccgagtcccccgagtccccgagtcccccgagtcccccgagtcccccgagtccccgagtcccc
                 ############################################################################################################################################################################################################################################################################################################


                      31810     31820     31830     31840     31850     31860     31870     31880     31890     31900     31910     31920     31930     31940     31950     31960     31970     31980     31990     32000     32010     32020     32030     32040     32050     32060     32070     32080     32090     32100
                 =========+=========+=========+=========+=========+=========+=========+=========+=========+=========+=========+=========+=========+=========+=========+=========+=========+=========+=========+=========+=========+=========+=========+=========+=========+=========+=========+=========+=========+=========+
PRV-WT complete  gagtcccccgagtcccccgagtcccccgagtcccccgagtcccccgagtccccgagtcccccgagtcccccgagtcccccgagtccccgagtcccccgagtcccccgagtcccccgagtcccccgagtcccccgagtcccccgagtcccccgagtcccccgagtccccgagtcccccgaacacaccaccgcagagacaaacaggttgggtaataaacaatttattaaccaagaatcaggcggacctgaaataggtccacgttgaaccggcgggcgctgcccttgaggttcgcccgcgtct
PRV-delete UL10  gagtcccccgagtcccccgagtcccccgagtcccccgagtcccccgagtccccgagtcccccgagtcccccgagtcccccgagtccccgagtcccccgagtcccccgagtcccccgagtcccccgagtcccccgagtcccccgagtcccccgagtcccccgagtccccgagtcccccgaacacaccaccgcagagacaaacaggttgggtaataaacaatttattaaccaagaatcaggcggacctgaaataggtccacgttgaaccggcgggcgctgcccttgaggttcgcccgcgtct
                 ############################################################################################################################################################################################################################################################################################################


                      32110     32120     32130     32140     32150     32160     32170     32180     32190     32200     32210     32220     32230     32240     32250     32260     32270     32280     32290     32300     32310     32320     32330     32340     32350     32360     32370     32380     32390     32400
                 =========+=========+=========+=========+=========+=========+=========+=========+=========+=========+=========+=========+=========+=========+=========+=========+=========+=========+=========+=========+=========+=========+=========+=========+=========+=========+=========+=========+=========+=========+
PRV-WT complete  cccgcaggcggcgcgcgatcaggcggcacgcggcgatgagcagggccagggccccaagtccggtggcgcgcatgtaccgcctgttgatgagagtgtccgactccaccacgcgcggggggagcagctcctgggccagagagacgtcctcgtcctcgtcgtcctcatcgtcctcgaactccgcgccccaggggcccggctcgggcgcctcctcggcgggctcgcgggtcaggtccgcccgggtgggccagacgcggccgaggccgcccaggggagagtccggcggtgggggtctctgctgct
PRV-delete UL10  cccgcaggcggcgcgcgatcaggcggcacgcggcgatgagcagggccagggccccaagtccggtggcgcgcatgtaccgcctgttgatgagagtgtccgactccaccacgcgcggggggagcagctcctgggccagagagacgtcctcgtcctcgtcgtcctcatcgtcctcgaactccgcgccccaggggcccggctcgggcgcctcctcggcgggctcgcgggtcaggtccgcccgggtgggccagacgcggccgaggccgcccaggggagagtccggcggtgggggtctctgctgct
                 ############################################################################################################################################################################################################################################################################################################


                      32410     32420     32430     32440     32450     32460     32470     32480     32490     32500     32510     32520     32530     32540     32550     32560     32570     32580     32590     32600     32610     32620     32630     32640     32650     32660     32670     32680     32690     32700
                 =========+=========+=========+=========+=========+=========+=========+=========+=========+=========+=========+=========+=========+=========+=========+=========+=========+=========+=========+=========+=========+=========+=========+=========+=========+=========+=========+=========+=========+=========+
PRV-WT complete  gccgcggcggcggaggagggtgggcgggagggacgagcgcggccaggtcgtcgaaccacggcgggggctggtggcggtccgccagcggggagcgcggggccggcggcgcaacctccggcgggagctcggggacggtggtgggcgcctcagacagcggcggccagggcagtcgctccagcggcgagcgcggcggggtcgccggggcctcggcctcttctccctcgctcgcccatagcggctgagggggcggcggcagcggtgatggcggcagcggcggcgcctctcctccggggaacggga
PRV-delete UL10  gccgcggcggcggaggagggtgggcgggagggacgagcgcggccaggtcgtcgaaccacggcgggggctggtggcggtccgccagcggggagcgcggggccggcggcgcaacctccggcgggagctcggggacggtggtgggcgcctcagacagcggcggccagggcagtcgctccagcggcgagcgcggcggggtcgccggggcctcggcctcttctccctcgctcgcccatagcggctgagggggcggcggcagcggtgatggcggcagcggcggcgcctctcctccggggaacggga
                 ############################################################################################################################################################################################################################################################################################################


                      32710     32720     32730     32740     32750     32760     32770     32780     32790     32800     32810     32820     32830     32840     32850     32860     32870     32880     32890     32900     32910     32920     32930     32940     32950     32960     32970     32980     32990     33000
                 =========+=========+=========+=========+=========+=========+=========+=========+=========+=========+=========+=========+=========+=========+=========+=========+=========+=========+=========+=========+=========+=========+=========+=========+=========+=========+=========+=========+=========+=========+
PRV-WT complete  acgggaacggctggtccgagggcggcggggtgaagagcgcgggtgggccggcggggtccctcggctcgggccacgaccacggcgcctccggggggcgctgctgagacggctgcggcggcagcggcggaggtgtgaacaggagcctagggggctccgcgggcttctcgggctccgccggcgccggtctcgtctgcgccggggcccgggcgggctcggccctcggcggcgccggggccggccgcgaagatggtggtggtggtggcggcggcggcggctggggtggtggtggagcggcggcgg
PRV-delete UL10  acgggaacggctggtccgagggcggcggggtgaagagcgcgggtgggccggcggggtccctcggctcgggccacgaccacggcgcctccggggggcgctgctgagacggctgcggcggcagcggcggaggtgtgaacaggagcctagggggctccgcgggcttctcgggctccgccggcgccggtctcgtctgcgccggggcccgggcgggctcggccctcggcggcgccggggccggccgcgaagatggtggtggtggtggcggcggcggcggctggggtggtggtggagcggcggcgg
                 ############################################################################################################################################################################################################################################################################################################


                      33010     33020     33030     33040     33050     33060     33070     33080     33090     33100     33110     33120     33130     33140     33150     33160     33170     33180     33190     33200     33210     33220     33230     33240     33250     33260     33270     33280     33290     33300
                 =========+=========+=========+=========+=========+=========+=========+=========+=========+=========+=========+=========+=========+=========+=========+=========+=========+=========+=========+=========+=========+=========+=========+=========+=========+=========+=========+=========+=========+=========+
PRV-WT complete  aggaggccgaggaggagatggtggtggcggcggtggaggaggaggtggtggtggcggtggcggtggtggtagaggccggtgctgttgttgctgttgttgttggagctgagacggcggcggggggccgcgcgctctcgggggcggcggccagcgacgtcggggccgccagcggagaccgcggcggctgggtctgcgccggggccggagtctccggcgaggccggcagcggcaccgcgacgccctccagcggcacagactgcggcggcgggatgtggagacggaggtgcccggccggcgggc
PRV-delete UL10  aggaggccgaggaggagatggtggtggcggcggtggaggaggaggtggtggtggcggtggcggtggtggtagaggccggtgctgttgttgctgttgttgttggagctgagacggcggcggggggccgcgcgctctcgggggcggcggccagcgacgtcggggccgccagcggagaccgcggcggctgggtctgcgccggggccggagtctccggcgaggccggcagcggcaccgcgacgccctccagcggcacagactgcggcggcgggatgtggagacggaggtgcccggccggcgggc
                 ############################################################################################################################################################################################################################################################################################################


                      33310     33320     33330     33340     33350     33360     33370     33380     33390     33400     33410     33420     33430     33440     33450     33460     33470     33480     33490     33500     33510     33520     33530     33540     33550     33560     33570     33580     33590     33600
                 =========+=========+=========+=========+=========+=========+=========+=========+=========+=========+=========+=========+=========+=========+=========+=========+=========+=========+=========+=========+=========+=========+=========+=========+=========+=========+=========+=========+=========+=========+
PRV-WT complete  gcgcgacggcggcggctcgcctcactggtcctccgggggctgggaccacatctccgagcccgactccgagggcggggacggctgctgctgctgttgctgctgccgccgccggtgggcccgctggagcagtcgccggcgcttgggcttgggcttgaggcgggcgcggttcagctcccccgggaggacggccctcgggttgagccgcgggcggaagactcccggtctggcgcgaggctgatacgggccttcgcggaacaagagccatggattttcggcgactgtggtctctaatacccaccg
PRV-delete UL10  gcgcgacggcggcggctcgcctcactggtcctccgggggctgggaccacatctccgagcccgactccgagggcggggacggctgctgctgctgttgctgctgccgccgccggtgggcccgctggagcagtcgccggcgcttgggcttgggcttgaggcgggcgcggttcagctcccccgggaggacggccctcgggttgagccgcgggcggaagactcccggtctggcgcgaggctgatacgggccttcgcggaacaagagccatggattttcggcgactgtggtctctaatacccaccg
                 ############################################################################################################################################################################################################################################################################################################


                      33610     33620     33630     33640     33650     33660     33670     33680     33690     33700     33710     33720     33730     33740     33750     33760     33770     33780     33790     33800     33810     33820     33830     33840     33850     33860     33870     33880     33890     33900
                 =========+=========+=========+=========+=========+=========+=========+=========+=========+=========+=========+=========+=========+=========+=========+=========+=========+=========+=========+=========+=========+=========+=========+=========+=========+=========+=========+=========+=========+=========+
PRV-WT complete  acgttcgcgccttggtgcgtagaacagcggagggaaggggctgcgccactggggccgcggggagatgtcccgcaggttgatctccacggctcgcatccgaagccgggccaccgtctcctcggcgctggccaccactcccgggggctccaggaggtgctcgggccgctgctccggctgctgggggggaacctccgcgggagccatcgctcggtgttccggccggggaagaagaggaggacgaggcggcttctcgggacggagtcgaggcggtggaggaggacgcggacgagagctccgagt
PRV-delete UL10  acgttcgcgccttggtgcgtagaacagcggagggaaggggctgcgccactggggccgcggggagatgtcccgcaggttgatctccacggctcgcatccgaagccgggccaccgtctcctcggcgctggccaccactcccgggggctccaggaggtgctcgggccgctgctccggctgctgggggggaacctccgcgggagccatcgctcggtgttccggccggggaagaagaggaggacgaggcggcttctcgggacggagtcgaggcggtggaggaggacgcggacgagagctccgagt
                 ############################################################################################################################################################################################################################################################################################################


                      33910     33920     33930     33940     33950     33960     33970     33980     33990     34000     34010     34020     34030     34040     34050     34060     34070     34080     34090     34100     34110     34120     34130     34140     34150     34160     34170     34180     34190     34200
                 =========+=========+=========+=========+=========+=========+=========+=========+=========+=========+=========+=========+=========+=========+=========+=========+=========+=========+=========+=========+=========+=========+=========+=========+=========+=========+=========+=========+=========+=========+
PRV-WT complete  ccgatgtcccgggaggagagcgggtccgacgctggtctggtccgaccgtgtgttcgcacttgtctgcgcaccccgctttatagcgttcgtcatcgagggggcggcccggggaggcggtccccgccaccctctcaccaaatttggtgtctcggacgtccggcggggcatcggccctcggcgctacgatgcggtcaccgtcgggtttctgggccgatggggcgggcccgggcgcggggggccggaaggtcgccgtcggggcagatgggggtggcggggcgggcggggcggcgggcccggggg
PRV-delete UL10  ccgatgtcccgggaggagagcgggtccgacgctggtctggtccgaccgtgtgttcgcacttgtctgcgcaccccgctttatagcgttcgtcatcgagggggcggcccggggaggcggtccccgccaccctctcaccaaatttggtgtctcggacgtccggcggggcatcggccctcggcgctacgatgcggtcaccgtcgggtttctgggccgatggggcgggcccgggcgcggggggccggaaggtcgccgtcggggcagatgggggtggcggggcgggcggggcggcgggcccggggg
                 ############################################################################################################################################################################################################################################################################################################


                      34210     34220     34230     34240     34250     34260     34270     34280     34290     34300     34310     34320     34330     34340     34350     34360     34370     34380     34390     34400     34410     34420     34430     34440     34450     34460     34470     34480     34490     34500
                 =========+=========+=========+=========+=========+=========+=========+=========+=========+=========+=========+=========+=========+=========+=========+=========+=========+=========+=========+=========+=========+=========+=========+=========+=========+=========+=========+=========+=========+=========+
PRV-WT complete  tcgtcgtcggggcagatgggggcggcggctgtgggggagcgggcggggcggggggtgccgggggcgcgggcggggcgccccggggcttggcggccggctgggacgatgggggctgagcggcggcggcggcgacggtggtggtcgcggcgggctgtggcgggggaccctgtgacggaggttggggccggggctggggctgggtctgggggatgggggccttggccgcggcgggctgcgacggtggggcctgggtcgggggagccggggccgacgggggagcctgagtctggggctggggtt
PRV-delete UL10  tcgtcgtcggggcagatgggggcggcggctgtgggggagcgggcggggcggggggtgccgggggcgcgggcggggcgccccggggcttggcggccggctgggacgatgggggctgagcggcggcggcggcgacggtggtggtcgcggcgggctgtggcgggggaccctgtgacggaggttggggccggggctggggctgggtctgggggatgggggccttggccgcggcgggctgcgacggtggggcctgggtcgggggagccggggccgacgggggagcctgagtctggggctggggtt
                 ############################################################################################################################################################################################################################################################################################################


                      34510     34520     34530     34540     34550     34560     34570     34580     34590     34600     34610     34620     34630     34640     34650     34660     34670     34680     34690     34700     34710     34720     34730     34740     34750     34760     34770     34780     34790     34800
                 =========+=========+=========+=========+=========+=========+=========+=========+=========+=========+=========+=========+=========+=========+=========+=========+=========+=========+=========+=========+=========+=========+=========+=========+=========+=========+=========+=========+=========+=========+
PRV-WT complete  ggggggtcggcttcgccgtcgcggcgggctgcggcgggggcgcctgggccgggggagccggggcctggggggtctgctccgccggcgcggcgggctgggtctggggggccttctcgggcttcgggggcgccgcggggtaaggcgggttgatgatcggcggcgggggctcgacgcgcgccttcagcgagggctggatctttcgcggcgacgagtgcggcttgggggtgggctcctggggccgctgcttgggggccgcggcggccttggcggccttcttggaggcggtggcggcggggggct
PRV-delete UL10  ggggggtcggcttcgccgtcgcggcgggctgcggcgggggcgcctgggccgggggagccggggcctggggggtctgctccgccggcgcggcgggctgggtctggggggccttctcgggcttcgggggcgccgcggggtaaggcgggttgatgatcggcggcgggggctcgacgcgcgccttcagcgagggctggatctttcgcggcgacgagtgcggcttgggggtgggctcctggggccgctgcttgggggccgcggcggccttggcggccttcttggaggcggtggcggcggggggct
                 ############################################################################################################################################################################################################################################################################################################


                      34810     34820     34830     34840     34850     34860     34870     34880     34890     34900     34910     34920     34930     34940     34950     34960     34970     34980     34990     35000     35010     35020     35030     35040     35050     35060     35070     35080     35090     35100
                 =========+=========+=========+=========+=========+=========+=========+=========+=========+=========+=========+=========+=========+=========+=========+=========+=========+=========+=========+=========+=========+=========+=========+=========+=========+=========+=========+=========+=========+=========+
PRV-WT complete  cctgccgctgctgctgctgctgttgttgctgctgcggcggcggcggttgcgatggcgccttgggggcggcggggggctcctgctgccgctgcggcggcggctgctgcggctgcggcggcggcggcgccctggtgagcggggtcgcctccacgggcgcggcgggcggcggcgccggcggggccagcttgacgagcggggtcgtctccgcgggcgcggcgggcggcggcgccggcttggcggcgggggccgcctccgcggggggagccggtggcggcggcgccgggggggccagcggcggga
PRV-delete UL10  cctgccgctgctgctgctgctgttgttgctgctgcggcggcggcggttgcgatggcgccttgggggcggcggggggctcctgctgccgctgcggcggcggctgctgcggctgcggcggcggcggcgccctggtgagcggggtcgcctccacgggcgcggcgggcggcggcgccggcggggccagcttgacgagcggggtcgtctccgcgggcgcggcgggcggcggcgccggcttggcggcgggggccgcctccgcggggggagccggtggcggcggcgccgggggggccagcggcggga
                 ############################################################################################################################################################################################################################################################################################################


                      35110     35120     35130     35140     35150     35160     35170     35180     35190     35200     35210     35220     35230     35240     35250     35260     35270     35280     35290     35300     35310     35320     35330     35340     35350     35360     35370     35380     35390     35400
                 =========+=========+=========+=========+=========+=========+=========+=========+=========+=========+=========+=========+=========+=========+=========+=========+=========+=========+=========+=========+=========+=========+=========+=========+=========+=========+=========+=========+=========+=========+
PRV-WT complete  gcgcgggcgggtcgaacggcggcgacgggggcagcggcagcgcgctggccaccaggtcgtcgccgagcaggtccagccacgggttggcgtcaaagtcgtcgtccgggacgaacccgtcgtcctcctcggccagctcgggcggcagcccggggatcggggtcgcgggccgggtgggcgtcgccgacacggcgtacgggaacgggtcgcccaggttgcggaagctcggccggccgtacagctcctccgcggtggccgggtccacgcgggcgtcgcccatgatgatggaggtgaacagcgggt
PRV-delete UL10  gcgcgggcgggtcgaacggcggcgacgggggcagcggcagcgcgctggccaccaggtcgtcgccgagcaggtccagccacgggttggcgtcaaagtcgtcgtccgggacgaacccgtcgtcctcctcggccagctcgggcggcagcccggggatcggggtcgcgggccgggtgggcgtcgccgacacggcgtacgggaacgggtcgcccaggttgcggaagctcggccggccgtacagctcctccgcggtggccgggtccacgcgggcgtcgcccatgatgatggaggtgaacagcgggt
                 ############################################################################################################################################################################################################################################################################################################


                      35410     35420     35430     35440     35450     35460     35470     35480     35490     35500     35510     35520     35530     35540     35550     35560     35570     35580     35590     35600     35610     35620     35630     35640     35650     35660     35670     35680     35690     35700
                 =========+=========+=========+=========+=========+=========+=========+=========+=========+=========+=========+=========+=========+=========+=========+=========+=========+=========+=========+=========+=========+=========+=========+=========+=========+=========+=========+=========+=========+=========+
PRV-WT complete  cccgggggtccatccccggcagctggtcctccgtcgtctccaggaaggggatctcccgcagcagcgggtcgtcctcgagcgtgaagcccacgcgccgcggcggctcgggcggcgcccagagcacgcgcgacaccgccagctgcgagtccaccagcacgaggcaggccggcgtgccggccaggggccgcgccgcgatcagcgccgagagccgctccagctgcggcgtcaggcacgcgttctcgatggggttgtcgtcgagcgccagcaggcgctcgccccaggagctgaggtccgtggaca
PRV-delete UL10  cccgggggtccatccccggcagctggtcctccgtcgtctccaggaaggggatctcccgcagcagcgggtcgtcctcgagcgtgaagcccacgcgccgcggcggctcgggcggcgcccagagcacgcgcgacaccgccagctgcgagtccaccagcacgaggcaggccggcgtgccggccaggggccgcgccgcgatcagcgccgagagccgctccagctgcggcgtcaggcacgcgttctcgatggggttgtcgtcgagcgccagcaggcgctcgccccaggagctgaggtccgtggaca
                 ############################################################################################################################################################################################################################################################################################################


                      35710     35720     35730     35740     35750     35760     35770     35780     35790     35800     35810     35820     35830     35840     35850     35860     35870     35880     35890     35900     35910     35920     35930     35940     35950     35960     35970     35980     35990     36000
                 =========+=========+=========+=========+=========+=========+=========+=========+=========+=========+=========+=========+=========+=========+=========+=========+=========+=========+=========+=========+=========+=========+=========+=========+=========+=========+=========+=========+=========+=========+
PRV-WT complete  cgccgcggtgcagcgcggggtcgcgcaggtcgcacagcgtcacggccagggtcaggcccgagccgggcgagtacacgtccaggttctccatggcgatcaccagcggggccccgacgagcaccgccgccgcggccaggtccatggcgctcacgcgcgtcgcgctgctcggcggcgagcccgtgacggtgaacgtcatgcccgtgcccacgggcgcgtacaggtcgccgggcgccggcgcgccgagcgccgcggcctcgcgcacgtgcgccgcggcgtccaggcgctccgtgaccgcggcgt
PRV-delete UL10  cgccgcggtgcagcgcggggtcgcgcaggtcgcacagcgtcacggccagggtcaggcccgagccgggcgagtacacgtccaggttctccatggcgatcaccagcggggccccgacgagcaccgccgccgcggccaggtccatggcgctcacgcgcgtcgcgctgctcggcggcgagcccgtgacggtgaacgtcatgcccgtgcccacgggcgcgtacaggtcgccgggcgccggcgcgccgagcgccgcggcctcgcgcacgtgcgccgcggcgtccaggcgctccgtgaccgcggcgt
                 ############################################################################################################################################################################################################################################################################################################


                      36010     36020     36030     36040     36050     36060     36070     36080     36090     36100     36110     36120     36130     36140     36150     36160     36170     36180     36190     36200     36210     36220     36230     36240     36250     36260     36270     36280     36290     36300
                 =========+=========+=========+=========+=========+=========+=========+=========+=========+=========+=========+=========+=========+=========+=========+=========+=========+=========+=========+=========+=========+=========+=========+=========+=========+=========+=========+=========+=========+=========+
PRV-WT complete  cgtacgcggcgttcgcctccggctgcagggcggaccacagcgccgagagcagccgcggggggatgcacatgcgcgccagcacggtgaaggtggccagcgcgcccggcacggcgcgcgccagccgctggcccgtctccgtgtgcgcccagggcgcccacaggtccgtctccgagaagcggccctcggtccagtcggcgacgcgcagcgtgaacagcgcctgcccgtcgcgcagcgggcgccgcggcgggcgcgcgcccgcgtccagcgtgtccaccgcgtccagcgcgcggtccagcaccg
PRV-delete UL10  cgtacgcggcgttcgcctccggctgcagggcggaccacagcgccgagagcagccgcggggggatgcacatgcgcgccagcacggtgaaggtggccagcgcgcccggcacggcgcgcgccagccgctggcccgtctccgtgtgcgcccagggcgcccacaggtccgtctccgagaagcggccctcggtccagtcggcgacgcgcagcgtgaacagcgcctgcccgtcgcgcagcgggcgccgcggcgggcgcgcgcccgcgtccagcgtgtccaccgcgtccagcgcgcggtccagcaccg
                 ############################################################################################################################################################################################################################################################################################################


                      36310     36320     36330     36340     36350     36360     36370     36380     36390     36400     36410     36420     36430     36440     36450     36460     36470     36480     36490     36500     36510     36520     36530     36540     36550     36560     36570     36580     36590     36600
                 =========+=========+=========+=========+=========+=========+=========+=========+=========+=========+=========+=========+=========+=========+=========+=========+=========+=========+=========+=========+=========+=========+=========+=========+=========+=========+=========+=========+=========+=========+
PRV-WT complete  cgtccagcgtcttggccatgtactcgtgctgcccggccaggtccacgcgcgtaaagttgaacaggtgcgccggctcgccggccaccgcggccaccatcaggtcggggagggcgagccgcaccgccggctccagcggcggctgcacgacgcggtccagcgccccgaaggtgcgcgagagcgtcgtggccagcacggccgcgtacgtctccgcggcggccttggccgccgagtcccagtcggcgcggcggtggcgcacgaagcgcgcgaaggtggcgaagcccgtgtcgcgggccttctcgt
PRV-delete UL10  cgtccagcgtcttggccatgtactcgtgctgcccggccaggtccacgcgcgtaaagttgaacaggtgcgccggctcgccggccaccgcggccaccatcaggtcggggagggcgagccgcaccgccggctccagcggcggctgcacgacgcggtccagcgccccgaaggtgcgcgagagcgtcgtggccagcacggccgcgtacgtctccgcggcggccttggccgccgagtcccagtcggcgcggcggtggcgcacgaagcgcgcgaaggtggcgaagcccgtgtcgcgggccttctcgt
                 ############################################################################################################################################################################################################################################################################################################


                      36610     36620     36630     36640     36650     36660     36670     36680     36690     36700     36710     36720     36730     36740     36750     36760     36770     36780     36790     36800     36810     36820     36830     36840     36850     36860     36870     36880     36890     36900
                 =========+=========+=========+=========+=========+=========+=========+=========+=========+=========+=========+=========+=========+=========+=========+=========+=========+=========+=========+=========+=========+=========+=========+=========+=========+=========+=========+=========+=========+=========+
PRV-WT complete  agttgagccgcaggtggatgatctcgttcatgaggcgcatgcccgccacggtcgccgtctccatgagcccctcggtgagcggcgggcgcagctcggcgccctcgtcggtgcacagcatggccgccagcttgtcgccgaccgcgcggtagcacacctggtactccacgggcacgttgttgtcgtccaggaagaccgtggggaacaccgcgtccgcggcgctgcgcgcctcgcgcaccgtgcagatgacgtcgcggtcgcccaggcgcgccgccacgcgctgcttcaggcgcgcgcacggcg
PRV-delete UL10  agttgagccgcaggtggatgatctcgttcatgaggcgcatgcccgccacggtcgccgtctccatgagcccctcggtgagcggcgggcgcagctcggcgccctcgtcggtgcacagcatggccgccagcttgtcgccgaccgcgcggtagcacacctggtactccacgggcacgttgttgtcgtccaggaagaccgtggggaacaccgcgtccgcggcgctgcgcgcctcgcgcaccgtgcagatgacgtcgcggtcgcccaggcgcgccgccacgcgctgcttcaggcgcgcgcacggcg
                 ############################################################################################################################################################################################################################################################################################################


                      36910     36920     36930     36940     36950     36960     36970     36980     36990     37000     37010     37020     37030     37040     37050     37060     37070     37080     37090     37100     37110     37120     37130     37140     37150     37160     37170     37180     37190     37200
                 =========+=========+=========+=========+=========+=========+=========+=========+=========+=========+=========+=========+=========+=========+=========+=========+=========+=========+=========+=========+=========+=========+=========+=========+=========+=========+=========+=========+=========+=========+
PRV-WT complete  ccgtcgaggcgcgcggccgcgccggcgtcggccccgggccgccgtaggccgcgtagaactgcagccgcagcgtgagcagctcgcggtgcgcggcgtacttgggcgggacccagcgcggcagccgctccgcgcgcgcgtcgaaggcggccgccagcgcgcgcagggcgtcgtgctgcagctcgggcccgatgcgcgccgccatgcggtcgagctcgcgcatgtcctcctcgacggcgccggcgtcgcggaagaagtcgcccagggccccggcgcgcgccgagtacttgctctcgatgcgcc
PRV-delete UL10  ccgtcgaggcgcgcggccgcgccggcgtcggccccgggccgccgtaggccgcgtagaactgcagccgcagcgtgagcagctcgcggtgcgcggcgtacttgggcgggacccagcgcggcagccgctccgcgcgcgcgtcgaaggcggccgccagcgcgcgcagggcgtcgtgctgcagctcgggcccgatgcgcgccgccatgcggtcgagctcgcgcatgtcctcctcgacggcgccggcgtcgcggaagaagtcgcccagggccccggcgcgcgccgagtacttgctctcgatgcgcc
                 ############################################################################################################################################################################################################################################################################################################


                      37210     37220     37230     37240     37250     37260     37270     37280     37290     37300     37310     37320     37330     37340     37350     37360     37370     37380     37390     37400     37410     37420     37430     37440     37450     37460     37470     37480     37490     37500
                 =========+=========+=========+=========+=========+=========+=========+=========+=========+=========+=========+=========+=========+=========+=========+=========+=========+=========+=========+=========+=========+=========+=========+=========+=========+=========+=========+=========+=========+=========+
PRV-WT complete  ccacgaacttggcgggcaggcgcgggtagacggcgtcgctgcgcagcgccagcacctccatggtcgccgtctgcagcgcgcgcgcgcgggcctcggccaggcgcgcctcgtcctcggaggcgccggcgcggccgcgcgcgcgcccaaagtcctcccaggcctcgtcccaggcgacctcggcggccgtcagccgctgccgcagcgtgtccgcctcctggcgcagcgtctgcagcgcgtcgatgcgctcggcgaactcgtccatgggcccgcggccgtcgacgcgcgtcgtcagcgggtgcg
PRV-delete UL10  ccacgaacttggcgggcaggcgcgggtagacggcgtcgctgcgcagcgccagcacctccatggtcgccgtctgcagcgcgcgcgcgcgggcctcggccaggcgcgcctcgtcctcggaggcgccggcgcggccgcgcgcgcgcccaaagtcctcccaggcctcgtcccaggcgacctcggcggccgtcagccgctgccgcagcgtgtccgcctcctggcgcagcgtctgcagcgcgtcgatgcgctcggcgaactcgtccatgggcccgcggccgtcgacgcgcgtcgtcagcgggtgcg
                 ############################################################################################################################################################################################################################################################################################################


                      37510     37520     37530     37540     37550     37560     37570     37580     37590     37600     37610     37620     37630     37640     37650     37660     37670     37680     37690     37700     37710     37720     37730     37740     37750     37760     37770     37780     37790     37800
                 =========+=========+=========+=========+=========+=========+=========+=========+=========+=========+=========+=========+=========+=========+=========+=========+=========+=========+=========+=========+=========+=========+=========+=========+=========+=========+=========+=========+=========+=========+
PRV-WT complete  tgtcgatgatgagccgcgcctggtgcagccactcgacggcctgcacgtccagctcgggcgcctcctcgacggcgccgagcagcagcgccgcctgcgtcaccagctcctccgcggaggcggcccggtcgatgttgagcgaggccggcggcggcgtcacccgcagcaggttcttgaggttggccagctcgagggcgccgtcgcggtcgcgcgccgccgccgcgtccaccacctcgcgcagcaggcgcgtggcgcgctccgtggccgcggcctggtccgcctccgccgcggcgaggccgtcgc
PRV-delete UL10  tgtcgatgatgagccgcgcctggtgcagccactcgacggcctgcacgtccagctcgggcgcctcctcgacggcgccgagcagcagcgccgcctgcgtcaccagctcctccgcggaggcggcccggtcgatgttgagcgaggccggcggcggcgtcacccgcagcaggttcttgaggttggccagctcgagggcgccgtcgcggtcgcgcgccgccgccgcgtccaccacctcgcgcagcaggcgcgtggcgcgctccgtggccgcggcctggtccgcctccgccgcggcgaggccgtcgc
                 ############################################################################################################################################################################################################################################################################################################


                      37810     37820     37830     37840     37850     37860     37870     37880     37890     37900     37910     37920     37930     37940     37950     37960     37970     37980     37990     38000     38010     38020     38030     38040     38050     38060     38070     38080     38090     38100
                 =========+=========+=========+=========+=========+=========+=========+=========+=========+=========+=========+=========+=========+=========+=========+=========+=========+=========+=========+=========+=========+=========+=========+=========+=========+=========+=========+=========+=========+=========+
PRV-WT complete  gcgccgccgccaggtcgcggcgcacgagcgcgcgcatggcctccgcgtacgccgtgcgctcgaagcgcgcaaagtcgaagcgctccaggtaggtcacggcgccgcgcagcagctcggtgctcggcacgtgcaggtgcacgccctcgtcgagcgcgcgcgccgcgtcggcggtcgcgcgcacgtacgtcacctcctcgagcgcgcggcagatctcggcgccgcggctgccgccggcctgcagcacgtccgcgcggcgcgcgcccagggcggccagctcggcctcgtactcggggaagccgc
PRV-delete UL10  gcgccgccgccaggtcgcggcgcacgagcgcgcgcatggcctccgcgtacgccgtgcgctcgaagcgcgcaaagtcgaagcgctccaggtaggtcacggcgccgcgcagcagctcggtgctcggcacgtgcaggtgcacgccctcgtcgagcgcgcgcgccgcgtcggcggtcgcgcgcacgtacgtcacctcctcgagcgcgcggcagatctcggcgccgcggctgccgccggcctgcagcacgtccgcgcggcgcgcgcccagggcggccagctcggcctcgtactcggggaagccgc
                 ############################################################################################################################################################################################################################################################################################################


                      38110     38120     38130     38140     38150     38160     38170     38180     38190     38200     38210     38220     38230     38240     38250     38260     38270     38280     38290     38300     38310     38320     38330     38340     38350     38360     38370     38380     38390     38400
                 =========+=========+=========+=========+=========+=========+=========+=========+=========+=========+=========+=========+=========+=========+=========+=========+=========+=========+=========+=========+=========+=========+=========+=========+=========+=========+=========+=========+=========+=========+
PRV-WT complete  ggcggtaaaagtccacgtacttggccaggcgcggcacgtgcgccatgtccgccccgacgaagcccacggcgtcgtagtagctgaagcgccccatgccggcggcggcgcgggccagcacgccgccggcgatgcgcgccagccgcgagagcggctccgtgtcggcgccgaagagcgagccgatgacgggcgccgcgacggcgtagtcgtcgagccaggtcaggcgctgcagcgcctgcagcggcgggcccgccgccgcgccggcgttctcgggcaggtgcgggttgaaggcgaacacggcgt
PRV-delete UL10  ggcggtaaaagtccacgtacttggccaggcgcggcacgtgcgccatgtccgccccgacgaagcccacggcgtcgtagtagctgaagcgccccatgccggcggcggcgcgggccagcacgccgccggcgatgcgcgccagccgcgagagcggctccgtgtcggcgccgaagagcgagccgatgacgggcgccgcgacggcgtagtcgtcgagccaggtcaggcgctgcagcgcctgcagcggcgggcccgccgccgcgccggcgttctcgggcaggtgcgggttgaaggcgaacacggcgt
                 ############################################################################################################################################################################################################################################################################################################


                      38410     38420     38430     38440     38450     38460     38470     38480     38490     38500     38510     38520     38530     38540     38550     38560     38570     38580     38590     38600     38610     38620     38630     38640     38650     38660     38670     38680     38690     38700
                 =========+=========+=========+=========+=========+=========+=========+=========+=========+=========+=========+=========+=========+=========+=========+=========+=========+=========+=========+=========+=========+=========+=========+=========+=========+=========+=========+=========+=========+=========+
PRV-WT complete  ccagcgccgtggtcacggtctcggcgttcgcggcgagcgcgcgctcggcggcggggcgcagctccgcggcgtcgtagccgcggtccgccgccacgtcgcgcaggcgcacgaactcggccgcgtcgaaggcgtgctgctcggcgcggcgcagcgccgcgtcggcggcgcgcgcccagagctggcgctgcgcgcgctccacctccgccgcgcggcgcgcctccacggccgccacggccgcctccagctcgccgacgcgctggcgcgccgcgagcgccatcttctgcagctgcggctcctcca
PRV-delete UL10  ccagcgccgtggtcacggtctcggcgttcgcggcgagcgcgcgctcggcggcggggcgcagctccgcggcgtcgtagccgcggtccgccgccacgtcgcgcaggcgcacgaactcggccgcgtcgaaggcgtgctgctcggcgcggcgcagcgccgcgtcggcggcgcgcgcccagagctggcgctgcgcgcgctccacctccgccgcgcggcgcgcctccacggccgccacggccgcctccagctcgccgacgcgctggcgcgccgcgagcgccatcttctgcagctgcggctcctcca
                 ############################################################################################################################################################################################################################################################################################################


                      38710     38720     38730     38740     38750     38760     38770     38780     38790     38800     38810     38820     38830     38840     38850     38860     38870     38880     38890     38900     38910     38920     38930     38940     38950     38960     38970     38980     38990     39000
                 =========+=========+=========+=========+=========+=========+=========+=========+=========+=========+=========+=========+=========+=========+=========+=========+=========+=========+=========+=========+=========+=========+=========+=========+=========+=========+=========+=========+=========+=========+
PRV-WT complete  cggcggccgcgcgcgccacctccagcgccaggtgccgcgcctcgaccagcgtcccctcgtcgggctgcgcgcgcagccgcccgagcatctccacggccgccgccacggccgccgacagcgcggcctgcagcgccgccacgcgctcagactcgcgcagcgccgcggcgcgctcctcgcactgccgcgccagcggctccaggaagcccagctccgggtgctccacggcggcgcggagcacccccgcggcgcgcgccgccttctccgccgtgtccgcgtccgccagcgccgccgccaccgcgt
PRV-delete UL10  cggcggccgcgcgcgccacctccagcgccaggtgccgcgcctcgaccagcgtcccctcgtcgggctgcgcgcgcagccgcccgagcatctccacggccgccgccacggccgccgacagcgcggcctgcagcgccgccacgcgctcagactcgcgcagcgccgcggcgcgctcctcgcactgccgcgccagcggctccaggaagcccagctccgggtgctccacggcggcgcggagcacccccgcggcgcgcgccgccttctccgccgtgtccgcgtccgccagcgccgccgccaccgcgt
                 ############################################################################################################################################################################################################################################################################################################


                      39010     39020     39030     39040     39050     39060     39070     39080     39090     39100     39110     39120     39130     39140     39150     39160     39170     39180     39190     39200     39210     39220     39230     39240     39250     39260     39270     39280     39290     39300
                 =========+=========+=========+=========+=========+=========+=========+=========+=========+=========+=========+=========+=========+=========+=========+=========+=========+=========+=========+=========+=========+=========+=========+=========+=========+=========+=========+=========+=========+=========+
PRV-WT complete  cggcgtgcgcgaggaagaagcgcgtgatcggcgggttgtccgcgagcgcgttgaccagcgcgccgagcggctgcccggcgcccaccagcgcgccggggtcggaggtcagcagctcgcggtagcgcgccaccagccgccacagcgccgcgaccaccgtgccgggcacgggcgtggccgtgatgtgcgcgagcaggtccaccgccgggcccagcgtggcaaagtcgccgagcagcgcgcgcagccgcatgtagaggtcgttctgctcggcggcgcgctgctgcgccgcctgcaccagcgtgc
PRV-delete UL10  cggcgtgcgcgaggaagaagcgcgtgatcggcgggttgtccgcgagcgcgttgaccagcgcgccgagcggctgcccggcgcccaccagcgcgccggggtcggaggtcagcagctcgcggtagcgcgccaccagccgccacagcgccgcgaccaccgtgccgggcacgggcgtggccgtgatgtgcgcgagcaggtccaccgccgggcccagcgtggcaaagtcgccgagcagcgcgcgcagccgcatgtagaggtcgttctgctcggcggcgcgctgctgcgccgcctgcaccagcgtgc
                 ############################################################################################################################################################################################################################################################################################################


                      39310     39320     39330     39340     39350     39360     39370     39380     39390     39400     39410     39420     39430     39440     39450     39460     39470     39480     39490     39500     39510     39520     39530     39540     39550     39560     39570     39580     39590     39600
                 =========+=========+=========+=========+=========+=========+=========+=========+=========+=========+=========+=========+=========+=========+=========+=========+=========+=========+=========+=========+=========+=========+=========+=========+=========+=========+=========+=========+=========+=========+
PRV-WT complete  gcagctcggcgtcgcgccgcgcgagccgcgcgcgcgtctcctggctcagcacgtggccgctcatgccgcggatgtgccgcagcaggtcctccatcgcgcgcacgcgcgtctcctcgtccggggcggccgccagggcggcgtccatggcgtcgaagcgcgccaggtcggcgtcggccgaggcgcgcttggccgcgcgctcgttgatctggttcacgtcgcgcgtgagcgtgtccagctcgcgccgctccagatgcccgtgcgcctgcgcctcggtcagcagcgcgaaccaggccccgaggc
PRV-delete UL10  gcagctcggcgtcgcgccgcgcgagccgcgcgcgcgtctcctggctcagcacgtggccgctcatgccgcggatgtgccgcagcaggtcctccatcgcgcgcacgcgcgtctcctcgtccggggcggccgccagggcggcgtccatggcgtcgaagcgcgccaggtcggcgtcggccgaggcgcgcttggccgcgcgctcgttgatctggttcacgtcgcgcgtgagcgtgtccagctcgcgccgctccagatgcccgtgcgcctgcgcctcggtcagcagcgcgaaccaggccccgaggc
                 ############################################################################################################################################################################################################################################################################################################


                      39610     39620     39630     39640     39650     39660     39670     39680     39690     39700     39710     39720     39730     39740     39750     39760     39770     39780     39790     39800     39810     39820     39830     39840     39850     39860     39870     39880     39890     39900
                 =========+=========+=========+=========+=========+=========+=========+=========+=========+=========+=========+=========+=========+=========+=========+=========+=========+=========+=========+=========+=========+=========+=========+=========+=========+=========+=========+=========+=========+=========+
PRV-WT complete  gctcgtccgtgctcacgtcctcgcccgcctccagcagctgcgccagcagcgcgccgtcgtgggcggcgggcgccgcgtccagcgcgcgcagccgctcctcggtggccgccagcgagtcgacgaggcggcgcaggcccgccaccgcggcgttggccacgcggaagcgctgcgcgccgttgcggtccatcagcatggccttggcgctgtactgcgcgccgcgcagaaagtactcgcggatggcgtcgccgaccatcagcgccagcttgcgcggcagcttggccgcgcgctccttgacgtcgc
PRV-delete UL10  gctcgtccgtgctcacgtcctcgcccgcctccagcagctgcgccagcagcgcgccgtcgtgggcggcgggcgccgcgtccagcgcgcgcagccgctcctcggtggccgccagcgagtcgacgaggcggcgcaggcccgccaccgcggcgttggccacgcggaagcgctgcgcgccgttgcggtccatcagcatggccttggcgctgtactgcgcgccgcgcagaaagtactcgcggatggcgtcgccgaccatcagcgccagcttgcgcggcagcttggccgcgcgctccttgacgtcgc
                 ############################################################################################################################################################################################################################################################################################################


                      39910     39920     39930     39940     39950     39960     39970     39980     39990     40000     40010     40020     40030     40040     40050     40060     40070     40080     40090     40100     40110     40120     40130     40140     40150     40160     40170     40180     40190     40200
                 =========+=========+=========+=========+=========+=========+=========+=========+=========+=========+=========+=========+=========+=========+=========+=========+=========+=========+=========+=========+=========+=========+=========+=========+=========+=========+=========+=========+=========+=========+
PRV-WT complete  gcgtgttcggctcggccgcgccgggcggcgcccccgcgtccacggcgtcaaagttggcgtgcgcggcctcgagcgccgcctcgagcgcgctcacgtcgcgcgcgcgctcgagcgcgcgctgcgactcgcgggactcgcgcgcccgcgcgcgcgcaaacaggccccgcgcgcgcgacaccaccgtctggtcgtagacgccgccggggtcccgcgccgcggcctccatgaagcacgccgagagctgcgcgtaggagcccccggactcgagcgcgtccagcgcccggtgcatggcctcgttcg
PRV-delete UL10  gcgtgttcggctcggccgcgccgggcggcgcccccgcgtccacggcgtcaaagttggcgtgcgcggcctcgagcgccgcctcgagcgcgctcacgtcgcgcgcgcgctcgagcgcgcgctgcgactcgcgggactcgcgcgcccgcgcgcgcgcaaacaggccccgcgcgcgcgacaccaccgtctggtcgtagacgccgccggggtcccgcgccgcggcctccatgaagcacgccgagagctgcgcgtaggagcccccggactcgagcgcgtccagcgcccggtgcatggcctcgttcg
                 ############################################################################################################################################################################################################################################################################################################


                      40210     40220     40230     40240     40250     40260     40270     40280     40290     40300     40310     40320     40330     40340     40350     40360     40370     40380     40390     40400     40410     40420     40430     40440     40450     40460     40470     40480     40490     40500
                 =========+=========+=========+=========+=========+=========+=========+=========+=========+=========+=========+=========+=========+=========+=========+=========+=========+=========+=========+=========+=========+=========+=========+=========+=========+=========+=========+=========+=========+=========+
PRV-WT complete  cgggccgcgcgacgcgcgccgccaccagcgccagcttggccacgggcgcggacgcctcgccgccggccgcgtccagcagcgcgcgcgcctccgtcagcaccatgtgcgtggcggccacaaagtccccggccgggccgcgctccggcagcgcggccaggacgggctcgaagagcgccgcgacggcgcacggcgcctccgcgtgggtgcgggcgccgttctccaccagaaagtcctgcaggtgcccgaagaggagctcggcgaagcgctccagcagcccggcgcgcccggggaacaggctcc
PRV-delete UL10  cgggccgcgcgacgcgcgccgccaccagcgccagcttggccacgggcgcggacgcctcgccgccggccgcgtccagcagcgcgcgcgcctccgtcagcaccatgtgcgtggcggccacaaagtccccggccgggccgcgctccggcagcgcggccaggacgggctcgaagagcgccgcgacggcgcacggcgcctccgcgtgggtgcgggcgccgttctccaccagaaagtcctgcaggtgcccgaagaggagctcggcgaagcgctccagcagcccggcgcgcccggggaacaggctcc
                 ############################################################################################################################################################################################################################################################################################################


                      40510     40520     40530     40540     40550     40560     40570     40580     40590     40600     40610     40620     40630     40640     40650     40660     40670     40680     40690     40700     40710     40720     40730     40740     40750     40760     40770     40780     40790     40800
                 =========+=========+=========+=========+=========+=========+=========+=========+=========+=========+=========+=========+=========+=========+=========+=========+=========+=========+=========+=========+=========+=========+=========+=========+=========+=========+=========+=========+=========+=========+
PRV-WT complete  ggcccgcgcacgcgaccacgtgctcctcgagctcgtcggcgtgggcctgcagcgggcccaggtcgagcgggaccagctccagctcccgcacggggaagcccatgggctcgcgggtgcgcgggcggtacttgcgcgacggccgcccgccgaccgtgtgcgtgctgctcaggtcctcctggctcgccggccacgggggccggcgccgcttgggcatgctcggccgcttctggacccgcggcggcgcggcgagggacgagggctggggctgggactggggcaccgccgtcgagaggggggcgg
PRV-delete UL10  ggcccgcgcacgcgaccacgtgctcctcgagctcgtcggcgtgggcctgcagcgggcccaggtcgagcgggaccagctccagctcccgcacggggaagcccatgggctcgcgggtgcgcgggcggtacttgcgcgacggccgcccgccgaccgtgtgcgtgctgctcaggtcctcctggctcgccggccacgggggccggcgccgcttgggcatgctcggccgcttctggacccgcggcggcgcggcgagggacgagggctggggctgggactggggcaccgccgtcgagaggggggcgg
                 ############################################################################################################################################################################################################################################################################################################


                      40810     40820     40830     40840     40850     40860     40870     40880     40890     40900     40910     40920     40930     40940     40950     40960     40970     40980     40990     41000     41010     41020     41030     41040     41050     41060     41070     41080     41090     41100
                 =========+=========+=========+=========+=========+=========+=========+=========+=========+=========+=========+=========+=========+=========+=========+=========+=========+=========+=========+=========+=========+=========+=========+=========+=========+=========+=========+=========+=========+=========+
PRV-WT complete  cggggacgggggcgacggcgaggacggggatggtcccgacggcggcgacggcgggggcgcccccggggccgcgcggcggcggcgcggcggccgccgggtccgggggcagggccatcgggtgcacggagggcaccgcccgctccacgtacggctcgtccacgaggtacgtctcgcccacgccgtagagcgcgggcacggcggcgaactggtcgtggggcaggaagaacacgaaggccccggcccagcggagctcgctggcggcgtagcccgcgacgtgcgcgtacacgtccgccgggcgca
PRV-delete UL10  cggggacgggggcgacggcgaggacggggatggtcccgacggcggcgacggcgggggcgcccccggggccgcgcggcggcggcgcggcggccgccgggtccgggggcagggccatcgggtgcacggagggcaccgcccgctccacgtacggctcgtccacgaggtacgtctcgcccacgccgtagagcgcgggcacggcggcgaactggtcgtggggcaggaagaacacgaaggccccggcccagcggagctcgctggcggcgtagcccgcgacgtgcgcgtacacgtccgccgggcgca
                 ############################################################################################################################################################################################################################################################################################################


                      41110     41120     41130     41140     41150     41160     41170     41180     41190     41200     41210     41220     41230     41240     41250     41260     41270     41280     41290     41300     41310     41320     41330     41340     41350     41360     41370     41380     41390     41400
                 =========+=========+=========+=========+=========+=========+=========+=========+=========+=========+=========+=========+=========+=========+=========+=========+=========+=========+=========+=========+=========+=========+=========+=========+=========+=========+=========+=========+=========+=========+
PRV-WT complete  ggcgcgccacgaaggcctgcgtgatctgcccgtggccgtgcgggtcgaagacgtagacgacgtcgccgcggcggtagaggcccaggcccacggcgccgaccacgaccagcgtgaaggactcggcgcgctgcgcccagaggccgtcgaagaaggcgcgcgccgtgatctgcgtgctctggaagccctcgccgggcggcgtgaagaagttgcactcgccgtgcacccgcgagaagacgcagtgcagcgcggcgccgccggcgccctcgtagacgatcttgttcgggagctcgctgatggcgc
PRV-delete UL10  ggcgcgccacgaaggcctgcgtgatctgcccgtggccgtgcgggtcgaagacgtagacgacgtcgccgcggcggtagaggcccaggcccacggcgccgaccacgaccagcgtgaaggactcggcgcgctgcgcccagaggccgtcgaagaaggcgcgcgccgtgatctgcgtgctctggaagccctcgccgggcggcgtgaagaagttgcactcgccgtgcacccgcgagaagacgcagtgcagcgcggcgccgccggcgccctcgtagacgatcttgttcgggagctcgctgatggcgc
                 ############################################################################################################################################################################################################################################################################################################


                      41410     41420     41430     41440     41450     41460     41470     41480     41490     41500     41510     41520     41530     41540     41550     41560     41570     41580     41590     41600     41610     41620     41630     41640     41650     41660     41670     41680     41690     41700
                 =========+=========+=========+=========+=========+=========+=========+=========+=========+=========+=========+=========+=========+=========+=========+=========+=========+=========+=========+=========+=========+=========+=========+=========+=========+=========+=========+=========+=========+=========+
PRV-WT complete  acatgcccccgtcgggccccgtccacgcctggccctcccgcaggcacgcgtccacggcgtccgccgtcagggcggcctccacgccgttcgtgaagaccaggcgcaggaaggagagggaggagcgcaggcacgagacgcccgacccgggccccaggtcggggtcatactgattacgatagccgacgaccaccgcgtcggccgtcatggctgaaataacacacgcgcgtgggggaaaaatctttttatttggacacggcgtcgacctcggccaggagccgatccacgggcacccacgtttgc
PRV-delete UL10  acatgcccccgtcgggccccgtccacgcctggccctcccgcaggcacgcgtccacggcgtccgccgtcagggcggcctccacgccgttcgtgaagaccaggcgcaggaaggagagggaggagcgcaggcacgagacgcccgacccgggccccaggtcggggtcatactgattacgatagccgacgaccaccgcgtcggccgtcatggctgaaataacacacgcgcgtgggggaaaaatctttttatttggacacggcgtcgacctcggccaggagccgatccacgggcacccacgtttgc
                 ############################################################################################################################################################################################################################################################################################################


                      41710     41720     41730     41740     41750     41760     41770     41780     41790     41800     41810     41820     41830     41840     41850     41860     41870     41880     41890     41900     41910     41920     41930     41940     41950     41960     41970     41980     41990     42000
                 =========+=========+=========+=========+=========+=========+=========+=========+=========+=========+=========+=========+=========+=========+=========+=========+=========+=========+=========+=========+=========+=========+=========+=========+=========+=========+=========+=========+=========+=========+
PRV-WT complete  gcgagcaggggcacgcccgcgcccgcgtcgtcgggcgcgcgcgcgccgcggcgcagcacctccaggttcacggcggcccagtcgcgcacgttgacgctcgccgagagcggcacggcgtcggggcccgcgtcgccgtcggtcgccacctcgtggtagtcgcccaggacgcggtccgcggcctcgcgcagctccccgggcgtgaaggcggccgcgggcggcgcctgctcctcgcgcgcctcggtccaggtctcgcggagcgtcgcggcgagcgccagcacgccgcgctcgtcgacggcgcgg
PRV-delete UL10  gcgagcaggggcacgcccgcgcccgcgtcgtcgggcgcgcgcgcgccgcggcgcagcacctccaggttcacggcggcccagtcgcgcacgttgacgctcgccgagagcggcacggcgtcggggcccgcgtcgccgtcggtcgccacctcgtggtagtcgcccaggacgcggtccgcggcctcgcgcagctccccgggcgtgaaggcggccgcgggcggcgcctgctcctcgcgcgcctcggtccaggtctcgcggagcgtcgcggcgagcgccagcacgccgcgctcgtcgacggcgcgg
                 ############################################################################################################################################################################################################################################################################################################


                      42010     42020     42030     42040     42050     42060     42070     42080     42090     42100     42110     42120     42130     42140     42150     42160     42170     42180     42190     42200     42210     42220     42230     42240     42250     42260     42270     42280     42290     42300
                 =========+=========+=========+=========+=========+=========+=========+=========+=========+=========+=========+=========+=========+=========+=========+=========+=========+=========+=========+=========+=========+=========+=========+=========+=========+=========+=========+=========+=========+=========+
PRV-WT complete  cgcagcgcggcgctgaccaggtcccagcgcgccaggaagcgctccacgggccgcagcacggtggcggcctgcaggcggccggccgtgagggcggcgctcgtctgcagcttgctcaggcgcgcgtggtcggcgcacagcgtccggaacacgggcgcctgcaggcccagcgcctcgcactcgcgcagcgcctccgtcgtctgcgccatcacgccgcgcagcgcccccgcgtcggcgcgcatgtccgagcggtgcgtgttaacctcggcgtggaaggcgcccacggcgtccacgacgtccgcc
PRV-delete UL10  cgcagcgcggcgctgaccaggtcccagcgcgccaggaagcgctccacgggccgcagcacggtggcggcctgcaggcggccggccgtgagggcggcgctcgtctgcagcttgctcaggcgcgcgtggtcggcgcacagcgtccggaacacgggcgcctgcaggcccagcgcctcgcactcgcgcagcgcctccgtcgtctgcgccatcacgccgcgcagcgcccccgcgtcggcgcgcatgtccgagcggtgcgtgttaacctcggcgtggaaggcgcccacggcgtccacgacgtccgcc
                 ############################################################################################################################################################################################################################################################################################################


                      42310     42320     42330     42340     42350     42360     42370     42380     42390     42400     42410     42420     42430     42440     42450     42460     42470     42480     42490     42500     42510     42520     42530     42540     42550     42560     42570     42580     42590     42600
                 =========+=========+=========+=========+=========+=========+=========+=========+=========+=========+=========+=========+=========+=========+=========+=========+=========+=========+=========+=========+=========+=========+=========+=========+=========+=========+=========+=========+=========+=========+
PRV-WT complete  aggcgccacgtgcccaggcagggcccgccctcgccgggcgcgaagacggaggccgtgcgcccgcggaagacgaagcggcaggtgaagaggcgctcgagcgccaccacgcgctcggccacggcgcccgcggccgtggccatcgagtccagcaggtcgcccgtgtaggtggccaggtcctccaggcggcgccggagggcgcgcgccatcaggtccccgaggaaggcgctggtggccagcgcgggcggcgcgggcaccggcgcctcgagcacctcggcggccgcgcgcacgaccccgtgcagc
PRV-delete UL10  aggcgccacgtgcccaggcagggcccgccctcgccgggcgcgaagacggaggccgtgcgcccgcggaagacgaagcggcaggtgaagaggcgctcgagcgccaccacgcgctcggccacggcgcccgcggccgtggccatcgagtccagcaggtcgcccgtgtaggtggccaggtcctccaggcggcgccggagggcgcgcgccatcaggtccccgaggaaggcgctggtggccagcgcgggcggcgcgggcaccggcgcctcgagcacctcggcggccgcgcgcacgaccccgtgcagc
                 ############################################################################################################################################################################################################################################################################################################


                      42610     42620     42630     42640     42650     42660     42670     42680     42690     42700     42710     42720     42730     42740     42750     42760     42770     42780     42790     42800     42810     42820     42830     42840     42850     42860     42870     42880     42890     42900
                 =========+=========+=========+=========+=========+=========+=========+=========+=========+=========+=========+=========+=========+=========+=========+=========+=========+=========+=========+=========+=========+=========+=========+=========+=========+=========+=========+=========+=========+=========+
PRV-WT complete  tcgtgcgcgagcgcctcgacgttgcgcgcgtgcgccaccgtcaggtcctcgcgcgccagcgccgccacgagcgcggcgagcttggtgcggtagccgctgacgttggggatgaggccggcgccgtagtcgcgcgcccacgtcagcacctgcgccagctccgcgcggcggtcgtaggaggccgtcaggaggcagttgtaggcgtcggccacggccgtcacgacgaagaagggcgcgaaggacgggtcggccatggcctcgaggtgcaccatggacgggtagttcacgaggtccgcggcggcg
PRV-delete UL10  tcgtgcgcgagcgcctcgacgttgcgcgcgtgcgccaccgtcaggtcctcgcgcgccagcgccgccacgagcgcggcgagcttggtgcggtagccgctgacgttggggatgaggccggcgccgtagtcgcgcgcccacgtcagcacctgcgccagctccgcgcggcggtcgtaggaggccgtcaggaggcagttgtaggcgtcggccacggccgtcacgacgaagaagggcgcgaaggacgggtcggccatggcctcgaggtgcaccatggacgggtagttcacgaggtccgcggcggcg
                 ############################################################################################################################################################################################################################################################################################################


                      42910     42920     42930     42940     42950     42960     42970     42980     42990     43000     43010     43020     43030     43040     43050     43060     43070     43080     43090     43100     43110     43120     43130     43140     43150     43160     43170     43180     43190     43200
                 =========+=========+=========+=========+=========+=========+=========+=========+=========+=========+=========+=========+=========+=========+=========+=========+=========+=========+=========+=========+=========+=========+=========+=========+=========+=========+=========+=========+=========+=========+
PRV-WT complete  gcgaggtcgacgagcgccgcgctcgtcggcgcctccatgggcgtctcgccggcgccgtcggcgcgcaggccccagcggctctgcgcgcggaagccgagccacgtgccgagggcctcgcgcgccgcgctgtgcgccagcccggcgcggcgctcggccaccgccacctcgcgcatcagcgcggcgcgcgcggcgcccacgtgctccgtgggcgggcgcggcgccatgcagtggcgcaccacgtgctccacgcccttctcggggagccccagcgccgccgtcacgtccccgtagaggggctcc
PRV-delete UL10  gcgaggtcgacgagcgccgcgctcgtcggcgcctccatgggcgtctcgccggcgccgtcggcgcgcaggccccagcggctctgcgcgcggaagccgagccacgtgccgagggcctcgcgcgccgcgctgtgcgccagcccggcgcggcgctcggccaccgccacctcgcgcatcagcgcggcgcgcgcggcgcccacgtgctccgtgggcgggcgcggcgccatgcagtggcgcaccacgtgctccacgcccttctcggggagccccagcgccgccgtcacgtccccgtagaggggctcc
                 ############################################################################################################################################################################################################################################################################################################


                      43210     43220     43230     43240     43250     43260     43270     43280     43290     43300     43310     43320     43330     43340     43350     43360     43370     43380     43390     43400     43410     43420     43430     43440     43450     43460     43470     43480     43490     43500
                 =========+=========+=========+=========+=========+=========+=========+=========+=========+=========+=========+=========+=========+=========+=========+=========+=========+=========+=========+=========+=========+=========+=========+=========+=========+=========+=========+=========+=========+=========+
PRV-WT complete  agcgtctccgcgacgtccgtgtaggcgcgcacgtagtccagcccgggcccgtagtcgtcgaggaggcggtgcacgacgccgccgacgaccgccgtgcagccgacggcgcgctgcgcgtccgtcaggcggggcgcgcggtccagcacggcgcgctcgaggaggtcgcagtggcggcgcgtgaagcccgcggtcacgagcgcctccacgacctgcagcggcgaggcgctggcctgcaccgcctcccacacgcccgtggcgtccacctgcgcgtggtcgaggtccgcggcggcggccacgtgc
PRV-delete UL10  agcgtctccgcgacgtccgtgtaggcgcgcacgtagtccagcccgggcccgtagtcgtcgaggaggcggtgcacgacgccgccgacgaccgccgtgcagccgacggcgcgctgcgcgtccgtcaggcggggcgcgcggtccagcacggcgcgctcgaggaggtcgcagtggcggcgcgtgaagcccgcggtcacgagcgcctccacgacctgcagcggcgaggcgctggcctgcaccgcctcccacacgcccgtggcgtccacctgcgcgtggtcgaggtccgcggcggcggccacgtgc
                 ############################################################################################################################################################################################################################################################################################################


                      43510     43520     43530     43540     43550     43560     43570     43580     43590     43600     43610     43620     43630     43640     43650     43660     43670     43680     43690     43700     43710     43720     43730     43740     43750     43760     43770     43780     43790     43800
                 =========+=========+=========+=========+=========+=========+=========+=========+=========+=========+=========+=========+=========+=========+=========+=========+=========+=========+=========+=========+=========+=========+=========+=========+=========+=========+=========+=========+=========+=========+
PRV-WT complete  acggcctgcgccccggggtccagcagcgccagcacgccctggtccgtgcgcaggcgccgcgccacggtgcccgggcgcagctcgcgcacgtacgcgtccccgtgcgcgagggccgcggacacgcgcacctcgacggccgtgcgcggcgtggcgttgacctcgtccacgggcaccagcgccgcgtcgtccacggcccgcgccgcctcggcgaactgctcgcgcagcgtcgggttctggcggtcgtggaagaactcgtcggcgatcgtgagggcaaagagcgcgcgcgtcagcggcttcagg
PRV-delete UL10  acggcctgcgccccggggtccagcagcgccagcacgccctggtccgtgcgcaggcgccgcgccacggtgcccgggcgcagctcgcgcacgtacgcgtccccgtgcgcgagggccgcggacacgcgcacctcgacggccgtgcgcggcgtggcgttgacctcgtccacgggcaccagcgccgcgtcgtccacggcccgcgccgcctcggcgaactgctcgcgcagcgtcgggttctggcggtcgtggaagaactcgtcggcgatcgtgagggcaaagagcgcgcgcgtcagcggcttcagg
                 ############################################################################################################################################################################################################################################################################################################


                      43810     43820     43830     43840     43850     43860     43870     43880     43890     43900     43910     43920     43930     43940     43950     43960     43970     43980     43990     44000     44010     44020     44030     44040     44050     44060     44070     44080     44090     44100
                 =========+=========+=========+=========+=========+=========+=========+=========+=========+=========+=========+=========+=========+=========+=========+=========+=========+=========+=========+=========+=========+=========+=========+=========+=========+=========+=========+=========+=========+=========+
PRV-WT complete  ccctcgttctgcatctggcgcaccgccagcgccagcggcagcgcccagttggtgcgccgcgccacgtggcgcaggccgtcctgcacaaagtccatgtcgtagagcgtggcgaagcggggctgcacgagcgcgcgcgccgtctgctccgggggcacgacgcggggcgcggccaggacgtcgtcggcggagcgcgcgcccgccagggcctcgtcgaaggcggcgcaccactcgctcacgaggcggaacatggcgtcggggccgaactcggccgaggtgcgcagcccggcctcggtgagcgcg
PRV-delete UL10  ccctcgttctgcatctggcgcaccgccagcgccagcggcagcgcccagttggtgcgccgcgccacgtggcgcaggccgtcctgcacaaagtccatgtcgtagagcgtggcgaagcggggctgcacgagcgcgcgcgccgtctgctccgggggcacgacgcggggcgcggccaggacgtcgtcggcggagcgcgcgcccgccagggcctcgtcgaaggcggcgcaccactcgctcacgaggcggaacatggcgtcggggccgaactcggccgaggtgcgcagcccggcctcggtgagcgcg
                 ############################################################################################################################################################################################################################################################################################################


                      44110     44120     44130     44140     44150     44160     44170     44180     44190     44200     44210     44220     44230     44240     44250     44260     44270     44280     44290     44300     44310     44320     44330     44340     44350     44360     44370     44380     44390     44400
                 =========+=========+=========+=========+=========+=========+=========+=========+=========+=========+=========+=========+=========+=========+=========+=========+=========+=========+=========+=========+=========+=========+=========+=========+=========+=========+=========+=========+=========+=========+
PRV-WT complete  gccgtggtggcgtcggcgttgacgacccccaggagctccagcagcggggccgtgccctcgtcccagtcgtggcgcaggcgccacagcgccaggccggccaggttctcggcgagcacggccgcctcgcaggtgccggtcgcggcgtaggcgcggcaggccaggcgcagcgcgcgcgcccagagcccgcgcacccccgcgggcgtctcgcggcccggggccatgaagaactccaggatgtgcgactgcaccaccgtggcgacggcatggtcggcctcctcgagcgcgcgcacgagcgcctcc
PRV-delete UL10  gccgtggtggcgtcggcgttgacgacccccaggagctccagcagcggggccgtgccctcgtcccagtcgtggcgcaggcgccacagcgccaggccggccaggttctcggcgagcacggccgcctcgcaggtgccggtcgcggcgtaggcgcggcaggccaggcgcagcgcgcgcgcccagagcccgcgcacccccgcgggcgtctcgcggcccggggccatgaagaactccaggatgtgcgactgcaccaccgtggcgacggcatggtcggcctcctcgagcgcgcgcacgagcgcctcc
                 ############################################################################################################################################################################################################################################################################################################


                      44410     44420     44430     44440     44450     44460     44470     44480     44490     44500     44510     44520     44530     44540     44550     44560     44570     44580     44590     44600     44610     44620     44630     44640     44650     44660     44670     44680     44690     44700
                 =========+=========+=========+=========+=========+=========+=========+=========+=========+=========+=========+=========+=========+=========+=========+=========+=========+=========+=========+=========+=========+=========+=========+=========+=========+=========+=========+=========+=========+=========+
PRV-WT complete  attataagagggtccgcgcgcgggcgccgccgcgcactcgaccccgcgcggcgcgacgatgagcgtgcagatcggcaacgggctgctgatggtggtcgcgccgggcacactaaccgtgggctcggcgcgcgcgcgccttatacgccaggtgacgctggcggacttttgcgagccccaggccgagcgccccgggctggtggtgctggcgctgcggcaccccgcggacctggccggcgccgcctacgcggccacgccgcccggcaagaaccaccgcgacctggaggaggcgtggctggccct
PRV-delete UL10  attataagagggtccgcgcgcgggcgccgccgcgcactcgaccccgcgcggcgcgacgatgagcgtgcagatcggcaacgggctgctgatggtggtcgcgccgggcacactaaccgtgggctcggcgcgcgcgcgccttatacgccaggtgacgctggcggacttttgcgagccccaggccgagcgccccgggctggtggtgctggcgctgcggcaccccgcggacctggccggcgccgcctacgcggccacgccgcccggcaagaaccaccgcgacctggaggaggcgtggctggccct
                 ############################################################################################################################################################################################################################################################################################################


                      44710     44720     44730     44740     44750     44760     44770     44780     44790     44800     44810     44820     44830     44840     44850     44860     44870     44880     44890     44900     44910     44920     44930     44940     44950     44960     44970     44980     44990     45000
                 =========+=========+=========+=========+=========+=========+=========+=========+=========+=========+=========+=========+=========+=========+=========+=========+=========+=========+=========+=========+=========+=========+=========+=========+=========+=========+=========+=========+=========+=========+
PRV-WT complete  cgacgagggcgggcgcggcctcggcggcgacggcatccgcgcctccgtcgtctcgctcaactttttggtggcggccgccgagaacggggacgacgcgctgcgcgcccacgtgacgaccaactaccgcgaccggcgcacggccgcgcgcctcgagcgcttcgcggccgtgctgcgcgccatgatccggagccacgtgttcccgcaccgcgcgctgcacgtcctcggagggctcctgggccacgtgacgcaggaccggctggccagcgtcacgtgcgtggcgcgcggcgaccaggaggcggc
PRV-delete UL10  cgacgagggcgggcgcggcctcggcggcgacggcatccgcgcctccgtcgtctcgctcaactttttggtggcggccgccgagaacggggacgacgcgctgcgcgcccacgtgacgaccaactaccgcgaccggcgcacggccgcgcgcctcgagcgcttcgcggccgtgctgcgcgccatgatccggagccacgtgttcccgcaccgcgcgctgcacgtcctcggagggctcctgggccacgtgacgcaggaccggctggccagcgtcacgtgcgtggcgcgcggcgaccaggaggcggc
                 ############################################################################################################################################################################################################################################################################################################


                      45010     45020     45030     45040     45050     45060     45070     45080     45090     45100     45110     45120     45130     45140     45150     45160     45170     45180     45190     45200     45210     45220     45230     45240     45250     45260     45270     45280     45290     45300
                 =========+=========+=========+=========+=========+=========+=========+=========+=========+=========+=========+=========+=========+=========+=========+=========+=========+=========+=========+=========+=========+=========+=========+=========+=========+=========+=========+=========+=========+=========+
PRV-WT complete  gcgcaccaacgacatggccgcgcgccgctcgcaggtgcacgtgcccgcgtgcgcgctgatggacgtggaccgcgagctgcgcctcggcggcgacgacgggctccgcttcgtgtacctggtcttcgtctacacgcagcgccaccgccgcgaggcgctgcgcgtccacgtggcggtgagccgcctgcccgagctcggcgacgccctcagcttcctcctggccggcacgcgcgtcgacaacgcgatccacggcacggacgaggccgacgcccccgccgcgcccgccgccgccgcggccttccc
PRV-delete UL10  gcgcaccaacgacatggccgcgcgccgctcgcaggtgcacgtgcccgcgtgcgcgctgatggacgtggaccgcgagctgcgcctcggcggcgacgacgggctccgcttcgtgtacctggtcttcgtctacacgcagcgccaccgccgcgaggcgctgcgcgtccacgtggcggtgagccgcctgcccgagctcggcgacgccctcagcttcctcctggccggcacgcgcgtcgacaacgcgatccacggcacggacgaggccgacgcccccgccgcgcccgccgccgccgcggccttccc
                 ############################################################################################################################################################################################################################################################################################################


                      45310     45320     45330     45340     45350     45360     45370     45380     45390     45400     45410     45420     45430     45440     45450     45460     45470     45480     45490     45500     45510     45520     45530     45540     45550     45560     45570     45580     45590     45600
                 =========+=========+=========+=========+=========+=========+=========+=========+=========+=========+=========+=========+=========+=========+=========+=========+=========+=========+=========+=========+=========+=========+=========+=========+=========+=========+=========+=========+=========+=========+
PRV-WT complete  cgcgtacctgttcaacgacccgcgcagcgcgcgctgcccgacgggccggctcaacacgcccgccgccgaggcgctgcccgtgtgggcccccgacatgcgcggccgcgccacccggaactcgtgcatgtacgccgcctacgtgcgcctcggcaccgtcgagcgcgtcgtgcgccgggccgagcgctgcggctcggtggacctgccgctggcccacatggagcgcttcacctgggacgtgggggcgtgggaggagtgtttcttctgaaaaaaaacggggggaggtgtgcgtgagacgggtct
PRV-delete UL10  cgcgtacctgttcaacgacccgcgcagcgcgcgctgcccgacgggccggctcaacacgcccgccgccgaggcgctgcccgtgtgggcccccgacatgcgcggccgcgccacccggaactcgtgcatgtacgccgcctacgtgcgcctcggcaccgtcgagcgcgtcgtgcgccgggccgagcgctgcggctcggtggacctgccgctggcccacatggagcgcttcacctgggacgtgggggcgtgggaggagtgtttcttctgaaaaaaaacggggggaggtgtgcgtgagacgggtct
                 ############################################################################################################################################################################################################################################################################################################


                      45610     45620     45630     45640     45650     45660     45670     45680     45690     45700     45710     45720     45730     45740     45750     45760     45770     45780     45790     45800     45810     45820     45830     45840     45850     45860     45870     45880     45890     45900
                 =========+=========+=========+=========+=========+=========+=========+=========+=========+=========+=========+=========+=========+=========+=========+=========+=========+=========+=========+=========+=========+=========+=========+=========+=========+=========+=========+=========+=========+=========+
PRV-WT complete  gatgttgctgacgagactaataaaaagcgggggatacgcgcgcgcgtgtgtccccccgttgacccttcatgccgcgtccgcgtcgtgtgtgactcacccccatcgtctctcccgcccgcgatcccggcccgtcccgactcgtcccgcccgcccagacgcgtcccgtcacgcccccgcgcacgccccgcccgccgggcgcggttttcccccctttccccccattggcggcctccgccgctaaaagcccgcgcgccccgagctcgggactctccgcgcactccccgtttcgagcacacaccg
PRV-delete UL10  gatgttgctgacgagactaataaaaagcgggggatacgcgcgcgcgtgtgtccccccgttgacccttcatgccgcgtccgcgtcgtgtgtgactcacccccatcgtctctcccgcccgcgatcccggcccgtcccgactcgtcccgcccgcccagacgcgtcccgtcacgcccccgcgcacgccccgcccgccgggcgcggttttcccccctttccccccattggcggcctccgccgctaaaagcccgcgcgccccgagctcgggactctccgcgcactccccgtttcgagcacacaccg
                 ############################################################################################################################################################################################################################################################################################################


                      45910     45920     45930     45940     45950     45960     45970     45980     45990     46000     46010     46020     46030     46040     46050     46060     46070     46080     46090     46100     46110     46120     46130     46140     46150     46160     46170     46180     46190     46200
                 =========+=========+=========+=========+=========+=========+=========+=========+=========+=========+=========+=========+=========+=========+=========+=========+=========+=========+=========+=========+=========+=========+=========+=========+=========+=========+=========+=========+=========+=========+
PRV-WT complete  tcatggcctcccccgtcgtgcccgccgccgccgccgccgctcccggcgcggactccttcctcgacgccgtctgccccgaggacgtcgcccgggccctggccgccgagctcgaggcgctccgcgccctcgggcacgacgtcggcgcgcccgcgcccggggcctcgcgtcgcgaggtggcgctgtttgtcacccgcgccgtggacgggctcaaggccttctcccgggtggacgagcgcgtgtacgtggcctgcggcaagctggtgcacctgcgcgtgcgcagccgcgaggcggacctggacg
PRV-delete UL10  tcatggcctcccccgtcgtgcccgccgccgccgccgccgctcccggcgcggactccttcctcgacgccgtctgccccgaggacgtcgcccgggccctggccgccgagctcgaggcgctccgcgccctcgggcacgacgtcggcgcgcccgcgcccggggcctcgcgtcgcgaggtggcgctgtttgtcacccgcgccgtggacgggctcaaggccttctcccgggtggacgagcgcgtgtacgtggcctgcggcaagctggtgcacctgcgcgtgcgcagccgcgaggcggacctggacg
                 ############################################################################################################################################################################################################################################################################################################


                      46210     46220     46230     46240     46250     46260     46270     46280     46290     46300     46310     46320     46330     46340     46350     46360     46370     46380     46390     46400     46410     46420     46430     46440     46450     46460     46470     46480     46490     46500
                 =========+=========+=========+=========+=========+=========+=========+=========+=========+=========+=========+=========+=========+=========+=========+=========+=========+=========+=========+=========+=========+=========+=========+=========+=========+=========+=========+=========+=========+=========+
PRV-WT complete  cctggctcgcctcgccggagctcgcgctgatccccgcggtggcgggcgccgtgcgccggcaccgcgcgcgcgtggaggcggccctccgctggttctggcgggaggcctacccggcgctctacgcgcgcggcctgcagtcggcgctcaagtacgaggagatgtacctggcccgcctcgagcacgggcgctgcgaggccatggaccagttctttgtgcgcctcgccgccgccgccgccacggcgacgcggcgcgccatggcgctcgtgctctgcggctcggacgcgtggccggaggtctttg
PRV-delete UL10  cctggctcgcctcgccggagctcgcgctgatccccgcggtggcgggcgccgtgcgccggcaccgcgcgcgcgtggaggcggccctccgctggttctggcgggaggcctacccggcgctctacgcgcgcggcctgcagtcggcgctcaagtacgaggagatgtacctggcccgcctcgagcacgggcgctgcgaggccatggaccagttctttgtgcgcctcgccgccgccgccgccacggcgacgcggcgcgccatggcgctcgtgctctgcggctcggacgcgtggccggaggtctttg
                 ############################################################################################################################################################################################################################################################################################################


                      46510     46520     46530     46540     46550     46560     46570     46580     46590     46600     46610     46620     46630     46640     46650     46660     46670     46680     46690     46700     46710     46720     46730     46740     46750     46760     46770     46780     46790     46800
                 =========+=========+=========+=========+=========+=========+=========+=========+=========+=========+=========+=========+=========+=========+=========+=========+=========+=========+=========+=========+=========+=========+=========+=========+=========+=========+=========+=========+=========+=========+
PRV-WT complete  acgcgtactttcgcgcgctggccacgcaggcgatcgtgcccgccaccccgctcatgctcttcgccggccgcgcgcgcggctcgctcgccagctgctacctgctgaaccccctgccgcgcaccaccgaggaggcggtgcgcgccatcacggacgaggtggcccccatcctgctgcgccgcggcggcgtcgggctctcgctgcagagcttcaaccgcacgccctcgggcgactgcacgcgcggcatcatggccgtgctcaaggcgctggactcgatgacggcggcgatcaacagcgacagcg
PRV-delete UL10  acgcgtactttcgcgcgctggccacgcaggcgatcgtgcccgccaccccgctcatgctcttcgccggccgcgcgcgcggctcgctcgccagctgctacctgctgaaccccctgccgcgcaccaccgaggaggcggtgcgcgccatcacggacgaggtggcccccatcctgctgcgccgcggcggcgtcgggctctcgctgcagagcttcaaccgcacgccctcgggcgactgcacgcgcggcatcatggccgtgctcaaggcgctggactcgatgacggcggcgatcaacagcgacagcg
                 ############################################################################################################################################################################################################################################################################################################


                      46810     46820     46830     46840     46850     46860     46870     46880     46890     46900     46910     46920     46930     46940     46950     46960     46970     46980     46990     47000     47010     47020     47030     47040     47050     47060     47070     47080     47090     47100
                 =========+=========+=========+=========+=========+=========+=========+=========+=========+=========+=========+=========+=========+=========+=========+=========+=========+=========+=========+=========+=========+=========+=========+=========+=========+=========+=========+=========+=========+=========+
PRV-WT complete  agcgccccacgggcgtgtgcgtgtacgtcgagccgtggcacgcggacgtgcgcgccgtgctgaacatgcgcggcatgctcgccgccgacgagagcctccgctgcgacaacatcttcagctgcctctggacgccggacctgttcttccagcgctaccagcggcacctggacggcgagcgcgccgtcaagtggacgctctttgacgaccgcgcctcgcacctgacctcgctgcacgggcccgactttgcgcgcgagtacgagcgcctggagcgcgccggcctcggcgtcgagtccctgccca
PRV-delete UL10  agcgccccacgggcgtgtgcgtgtacgtcgagccgtggcacgcggacgtgcgcgccgtgctgaacatgcgcggcatgctcgccgccgacgagagcctccgctgcgacaacatcttcagctgcctctggacgccggacctgttcttccagcgctaccagcggcacctggacggcgagcgcgccgtcaagtggacgctctttgacgaccgcgcctcgcacctgacctcgctgcacgggcccgactttgcgcgcgagtacgagcgcctggagcgcgccggcctcggcgtcgagtccctgccca
                 ############################################################################################################################################################################################################################################################################################################


                      47110     47120     47130     47140     47150     47160     47170     47180     47190     47200     47210     47220     47230     47240     47250     47260     47270     47280     47290     47300     47310     47320     47330     47340     47350     47360     47370     47380     47390     47400
                 =========+=========+=========+=========+=========+=========+=========+=========+=========+=========+=========+=========+=========+=========+=========+=========+=========+=========+=========+=========+=========+=========+=========+=========+=========+=========+=========+=========+=========+=========+
PRV-WT complete  tccaggacatggccttcctcatcgtccgcagcgccgtcatgacgggcagccccttcctcatgatgaaggacgcgtgcaaccggcacttccacacggacacgcgcggcgccgccctcgccacgtccaacctctgcacggagatcgtgcagcgggccacgcccggcgagaacggcgtctgcaacctggccagcgtgaacctgcccgcgtgcctggcgggcggcgccttcgactttgccgcgctgcgccgggccgcgcgcgtcgccgccgtcttcgtcaacgccatgatgcgcatcgggaact
PRV-delete UL10  tccaggacatggccttcctcatcgtccgcagcgccgtcatgacgggcagccccttcctcatgatgaaggacgcgtgcaaccggcacttccacacggacacgcgcggcgccgccctcgccacgtccaacctctgcacggagatcgtgcagcgggccacgcccggcgagaacggcgtctgcaacctggccagcgtgaacctgcccgcgtgcctggcgggcggcgccttcgactttgccgcgctgcgccgggccgcgcgcgtcgccgccgtcttcgtcaacgccatgatgcgcatcgggaact
                 ############################################################################################################################################################################################################################################################################################################


                      47410     47420     47430     47440     47450     47460     47470     47480     47490     47500     47510     47520     47530     47540     47550     47560     47570     47580     47590     47600     47610     47620     47630     47640     47650     47660     47670     47680     47690     47700
                 =========+=========+=========+=========+=========+=========+=========+=========+=========+=========+=========+=========+=========+=========+=========+=========+=========+=========+=========+=========+=========+=========+=========+=========+=========+=========+=========+=========+=========+=========+
PRV-WT complete  acccgacggaggcctcggtcgagggcgtgcggcgcagccgctcgctgggcatcggcctgcagggcctgcacacgaccgtgctggcgctggacatggacatggccgacccggcggcgcggcgcctcaacgccgccatcgccgaggagctgctctacggcgtcatggacgccagcgtggagctctgcgagcgcggcctgcgccccttcgacggcttcgagcacagccgctacgcgcgcggggtcatgcccttcgacgcctacgagcgcgtctcgctccgcgagccgatgcgctgggacgcgc
PRV-delete UL10  acccgacggaggcctcggtcgagggcgtgcggcgcagccgctcgctgggcatcggcctgcagggcctgcacacgaccgtgctggcgctggacatggacatggccgacccggcggcgcggcgcctcaacgccgccatcgccgaggagctgctctacggcgtcatggacgccagcgtggagctctgcgagcgcggcctgcgccccttcgacggcttcgagcacagccgctacgcgcgcggggtcatgcccttcgacgcctacgagcgcgtctcgctccgcgagccgatgcgctgggacgcgc
                 ############################################################################################################################################################################################################################################################################################################


                      47710     47720     47730     47740     47750     47760     47770     47780     47790     47800     47810     47820     47830     47840     47850     47860     47870     47880     47890     47900     47910     47920     47930     47940     47950     47960     47970     47980     47990     48000
                 =========+=========+=========+=========+=========+=========+=========+=========+=========+=========+=========+=========+=========+=========+=========+=========+=========+=========+=========+=========+=========+=========+=========+=========+=========+=========+=========+=========+=========+=========+
PRV-WT complete  tgcgcgtccgcatcgcggagcacggcgtgtacaacgcgcagtttgtggcgctgatgcccaccgtgtcctcgtcgcaggtcaccgagagcagcgagggcttctcgcccaccttcaccaacatgttcagcaaggtcaccatctcgggggagctcctgcggcccaacctgccgctcatggagacgctgcggcgcctgtttccgcgcgagtgcgcgcgccgggacgccgtggcgcggctggagcgcgcgcagtggtccgtggccgcggccttcggggagctgcccgccgggcacccgctggcca
PRV-delete UL10  tgcgcgtccgcatcgcggagcacggcgtgtacaacgcgcagtttgtggcgctgatgcccaccgtgtcctcgtcgcaggtcaccgagagcagcgagggcttctcgcccaccttcaccaacatgttcagcaaggtcaccatctcgggggagctcctgcggcccaacctgccgctcatggagacgctgcggcgcctgtttccgcgcgagtgcgcgcgccgggacgccgtggcgcggctggagcgcgcgcagtggtccgtggccgcggccttcggggagctgcccgccgggcacccgctggcca
                 ############################################################################################################################################################################################################################################################################################################


                      48010     48020     48030     48040     48050     48060     48070     48080     48090     48100     48110     48120     48130     48140     48150     48160     48170     48180     48190     48200     48210     48220     48230     48240     48250     48260     48270     48280     48290     48300
                 =========+=========+=========+=========+=========+=========+=========+=========+=========+=========+=========+=========+=========+=========+=========+=========+=========+=========+=========+=========+=========+=========+=========+=========+=========+=========+=========+=========+=========+=========+
PRV-WT complete  agttcaagacggccttcgagtacgaccaggagctgctcatcgacatgtgcgcggaccgcgccccctttgtggaccacagccagtccatgtccctcttcctgaccgagcccgccgacgggaagttacacgcctcccgcgtcatgggcctcctcatgcgggcatataacctgggcctcaagacgggcatgtactactgcaagatcaggaaggccaccaacaacggggtgttcaccggcggggacctggtctgcacgagctgccacctgtgagcgcgcgccatggagtacttttacacgtccc
PRV-delete UL10  agttcaagacggccttcgagtacgaccaggagctgctcatcgacatgtgcgcggaccgcgccccctttgtggaccacagccagtccatgtccctcttcctgaccgagcccgccgacgggaagttacacgcctcccgcgtcatgggcctcctcatgcgggcatataacctgggcctcaagacgggcatgtactactgcaagatcaggaaggccaccaacaacggggtgttcaccggcggggacctggtctgcacgagctgccacctgtgagcgcgcgccatggagtacttttacacgtccc
                 ############################################################################################################################################################################################################################################################################################################


                      48310     48320     48330     48340     48350     48360     48370     48380     48390     48400     48410     48420     48430     48440     48450     48460     48470     48480     48490     48500     48510     48520     48530     48540     48550     48560     48570     48580     48590     48600
                 =========+=========+=========+=========+=========+=========+=========+=========+=========+=========+=========+=========+=========+=========+=========+=========+=========+=========+=========+=========+=========+=========+=========+=========+=========+=========+=========+=========+=========+=========+
PRV-WT complete  agtgccccgacatggaccacctccgctcgctgagcgtggccaaccgctggctcgagacggacctgccgctgggcgacgacgccaaggacgtggccgcgctcagcgaggccgagctcgagttctaccgcttcctcttcgccttcctctccgccgcggacgacctcgtcaacgtcaacctgggcagcctctcggagctcttcacgcagaaggacatcctgcactactacatcgagcaggagtgcatcgaggtggtgcactcgcgcgtgtacagcgccatccagctgatgctgttccgcggcg
PRV-delete UL10  agtgccccgacatggaccacctccgctcgctgagcgtggccaaccgctggctcgagacggacctgccgctgggcgacgacgccaaggacgtggccgcgctcagcgaggccgagctcgagttctaccgcttcctcttcgccttcctctccgccgcggacgacctcgtcaacgtcaacctgggcagcctctcggagctcttcacgcagaaggacatcctgcactactacatcgagcaggagtgcatcgaggtggtgcactcgcgcgtgtacagcgccatccagctgatgctgttccgcggcg
                 ############################################################################################################################################################################################################################################################################################################


                      48610     48620     48630     48640     48650     48660     48670     48680     48690     48700     48710     48720     48730     48740     48750     48760     48770     48780     48790     48800     48810     48820     48830     48840     48850     48860     48870     48880     48890     48900
                 =========+=========+=========+=========+=========+=========+=========+=========+=========+=========+=========+=========+=========+=========+=========+=========+=========+=========+=========+=========+=========+=========+=========+=========+=========+=========+=========+=========+=========+=========+
PRV-WT complete  acgccgcggcgcgcgagcgctacgtgcgcgcggcgctgcgggacgaggccatccgccgcaaggtggagtggctcgactcgcgcgtcgccgagtgcgcctccgtcgcggaaaagtacctgctcatgatcctcatcgagggcatcttcttcgcctcctcgttcgcctccatctcgtacctccgcacgcacaacctgtttgtggtgacgtgccagtccaacgacttcatcagccgcgacgaggccatccacacctcggcctcgtgctgcatctacaacaactacctgggggacgccccgcgcc
PRV-delete UL10  acgccgcggcgcgcgagcgctacgtgcgcgcggcgctgcgggacgaggccatccgccgcaaggtggagtggctcgactcgcgcgtcgccgagtgcgcctccgtcgcggaaaagtacctgctcatgatcctcatcgagggcatcttcttcgcctcctcgttcgcctccatctcgtacctccgcacgcacaacctgtttgtggtgacgtgccagtccaacgacttcatcagccgcgacgaggccatccacacctcggcctcgtgctgcatctacaacaactacctgggggacgccccgcgcc
                 ############################################################################################################################################################################################################################################################################################################


                      48910     48920     48930     48940     48950     48960     48970     48980     48990     49000     49010     49020     49030     49040     49050     49060     49070     49080     49090     49100     49110     49120     49130     49140     49150     49160     49170     49180     49190     49200
                 =========+=========+=========+=========+=========+=========+=========+=========+=========+=========+=========+=========+=========+=========+=========+=========+=========+=========+=========+=========+=========+=========+=========+=========+=========+=========+=========+=========+=========+=========+
PRV-WT complete  ccgacgaggcccgcatccaccagctcttcgccgaggcggtggagatcgagtgcgagtttctgcgggcgcgcgccccgcgcgacagcctcctgctggacctgccggccatcatctcgtacgtgcgctacagcgcggaccggctgctgcaggccatcggcgcgagcccgctctttggcgcgcccgcccccgcggcggactttcccatggcgctgatggtcgccgagaagcacaccaactttttcgagcggcgcagcaccaactacacggggaccgtcgtgaacgacctgtagcgcccagcgg
PRV-delete UL10  ccgacgaggcccgcatccaccagctcttcgccgaggcggtggagatcgagtgcgagtttctgcgggcgcgcgccccgcgcgacagcctcctgctggacctgccggccatcatctcgtacgtgcgctacagcgcggaccggctgctgcaggccatcggcgcgagcccgctctttggcgcgcccgcccccgcggcggactttcccatggcgctgatggtcgccgagaagcacaccaactttttcgagcggcgcagcaccaactacacggggaccgtcgtgaacgacctgtagcgcccagcgg
                 ############################################################################################################################################################################################################################################################################################################


                      49210     49220     49230     49240     49250     49260     49270     49280     49290     49300     49310     49320     49330     49340     49350     49360     49370     49380     49390     49400     49410     49420     49430     49440     49450     49460     49470     49480     49490     49500
                 =========+=========+=========+=========+=========+=========+=========+=========+=========+=========+=========+=========+=========+=========+=========+=========+=========+=========+=========+=========+=========+=========+=========+=========+=========+=========+=========+=========+=========+=========+
PRV-WT complete  cggcgacccccgctctcccccctcctcctctctctgcgacaaaacactaataaagcgttgagacactagcgcgcgcctccggccgtcatccttggggcgagggtgggatggggaagtggaggcgggatgggagagtgtggggagagtgtggggagagtggggtggggagagtgcggggatgatgacgggggaagggagatggggctgacgggggtggactgggactggggaaggatgacggcggccggcgcagagtgggaggcggtgaggtggacgggggacggaggggaggaccgatgt
PRV-delete UL10  cggcgacccccgctctcccccctcctcctctctctgcgacaaaacactaataaagcgttgagacactagcgcgcgcctccggccgtcatccttggggcgagggtgggatggggaagtggaggcgggatgggagagtgtggggagagtgtggggagagtggggtggggagagtgcggggatgatgacgggggaagggagatggggctgacgggggtggactgggactggggaaggatgacggcggccggcgcagagtgggaggcggtgaggtggacgggggacggaggggaggaccgatgt
                 ############################################################################################################################################################################################################################################################################################################


                      49510     49520     49530     49540     49550     49560     49570     49580     49590     49600     49610     49620     49630     49640     49650     49660     49670     49680     49690     49700     49710     49720     49730     49740     49750     49760     49770     49780     49790     49800
                 =========+=========+=========+=========+=========+=========+=========+=========+=========+=========+=========+=========+=========+=========+=========+=========+=========+=========+=========+=========+=========+=========+=========+=========+=========+=========+=========+=========+=========+=========+
PRV-WT complete  gggacggcagaggggcggcagaggggcggcagaggggcggcagaggggcggcagaggggcggcagaggggcggcagaggggcggcagaggggcggcagaggggcggcagaggggcggcagagggacggcagaggggcggcagagggacggcagaggggcggcagaggggcggcagaggggcggcagaggggcggcggcgaggtgagacggagcccgcggacggagtggccggcggggggaggcgacgacggagagagaaggtgggggagagggcgagcatcacacgggccgcacaaccga
PRV-delete UL10  gggacggcagaggggcggcagaggggcggcagaggggcggcagaggggcggcagaggggcggcagaggggcggcagaggggcggcagaggggcggcagaggggcggcagaggggcggcagagggacggcagaggggcggcagagggacggcagaggggcggcagaggggcggcagaggggcggcagaggggcggcggcgaggtgagacggagcccgcggacggagtggccggcggggggaggcgacgacggagagagaaggtgggggagagggcgagcatcacacgggccgcacaaccga
                 ############################################################################################################################################################################################################################################################################################################


                      49810     49820     49830     49840     49850     49860     49870     49880     49890     49900     49910     49920     49930     49940     49950     49960     49970     49980     49990     50000     50010     50020     50030     50040     50050     50060     50070     50080     50090     50100
                 =========+=========+=========+=========+=========+=========+=========+=========+=========+=========+=========+=========+=========+=========+=========+=========+=========+=========+=========+=========+=========+=========+=========+=========+=========+=========+=========+=========+=========+=========+
PRV-WT complete  ggatttttttcaagttgttttttattttctcctatgggcgttacagtcgtcccagtacgtcatgaggacggcctggtagttcggaggcggaggtatgtgcttccaaaacaggcccgccagctccttcgcccgcgtctcgttcttcacgtgccgcagcagcgagccaaagaccttgttcacgtccgagggctcctgcacgatggggacgcgccgcagcacgctcaggtgcccgtgccgcgccggcgtgagcatggccaccacgtgctggatgaactcgcgctcggcgcggcgcgagcgcgc
PRV-delete UL10  ggatttttttcaagttgttttttattttctcctatgggcgttacagtcgtcccagtacgtcatgaggacggcctggtagttcggaggcggaggtatgtgcttccaaaacaggcccgccagctccttcgcccgcgtctcgttcttcacgtgccgcagcagcgagccaaagaccttgttcacgtccgagggctcctgcacgatggggacgcgccgcagcacgctcaggtgcccgtgccgcgccggcgtgagcatggccaccacgtgctggatgaactcgcgctcggcgcggcgcgagcgcgc
                 ############################################################################################################################################################################################################################################################################################################


                      50110     50120     50130     50140     50150     50160     50170     50180     50190     50200     50210     50220     50230     50240     50250     50260     50270     50280     50290     50300     50310     50320     50330     50340     50350     50360     50370     50380     50390     50400
                 =========+=========+=========+=========+=========+=========+=========+=========+=========+=========+=========+=========+=========+=========+=========+=========+=========+=========+=========+=========+=========+=========+=========+=========+=========+=========+=========+=========+=========+=========+
PRV-WT complete  gtccccggccgccggcagcagcgcgagcgccttgccggcgtcgcgcatgatgtcggggcagcgggacgcgtacttgagcttgacgtcctcgggcgccacctcgcggccctccagcgactcggccacctgctgcaccgagtccacgtccggcgcccggtgcaggtccgtgtggcagcgcacgaacgcggccaggaactccgagtagtccacccccaggctcgcgaggacgtcgcggcagcgcagcaccagcgggaacatgggcgcgatgtcgaggatcatgtcgcacccggtgaggatcat
PRV-delete UL10  gtccccggccgccggcagcagcgcgagcgccttgccggcgtcgcgcatgatgtcggggcagcgggacgcgtacttgagcttgacgtcctcgggcgccacctcgcggccctccagcgactcggccacctgctgcaccgagtccacgtccggcgcccggtgcaggtccgtgtggcagcgcacgaacgcggccaggaactccgagtagtccacccccaggctcgcgaggacgtcgcggcagcgcagcaccagcgggaacatgggcgcgatgtcgaggatcatgtcgcacccggtgaggatcat
                 ############################################################################################################################################################################################################################################################################################################


                      50410     50420     50430     50440     50450     50460     50470     50480     50490     50500     50510     50520     50530     50540     50550     50560     50570     50580     50590     50600     50610     50620     50630     50640     50650     50660     50670     50680     50690     50700
                 =========+=========+=========+=========+=========+=========+=========+=========+=========+=========+=========+=========+=========+=========+=========+=========+=========+=========+=========+=========+=========+=========+=========+=========+=========+=========+=========+=========+=========+=========+
PRV-WT complete  gtccgtgtcggtcgtgtgcacctgcgcgaccgtgttggtgtggtagaggttggcgcagacgtcgtccgcctccatgtccgacacgtccacgtaggcgtagcccatgtggcggatgaggttgacgcagaggcggtggacgatgcgcggcgcgtgcagcatcgtgctccagcgcggccgcggcggcgcgtcggcggccccgtcgccgtcggcgcgcgccgccgcggccatgatggccttggcgccgtgcgcgacgcggccgttcccgaagatgccgcggtccgagacgaagatggggaagta
PRV-delete UL10  gtccgtgtcggtcgtgtgcacctgcgcgaccgtgttggtgtggtagaggttggcgcagacgtcgtccgcctccatgtccgacacgtccacgtaggcgtagcccatgtggcggatgaggttgacgcagaggcggtggacgatgcgcggcgcgtgcagcatcgtgctccagcgcggccgcggcggcgcgtcggcggccccgtcgccgtcggcgcgcgccgccgcggccatgatggccttggcgccgtgcgcgacgcggccgttcccgaagatgccgcggtccgagacgaagatggggaagta
                 ############################################################################################################################################################################################################################################################################################################


                      50710     50720     50730     50740     50750     50760     50770     50780     50790     50800     50810     50820     50830     50840     50850     50860     50870     50880     50890     50900     50910     50920     50930     50940     50950     50960     50970     50980     50990     51000
                 =========+=========+=========+=========+=========+=========+=========+=========+=========+=========+=========+=========+=========+=========+=========+=========+=========+=========+=========+=========+=========+=========+=========+=========+=========+=========+=========+=========+=========+=========+
PRV-WT complete  ggtgcgcttgtgcagcatgcgcagcaggcgcagcaggcagcgggcggtcgtggtcgcgttgtcctcggtcgtctcctggtagtgcttctccatgagggtgtacatgacgttccacaggtcgatggcgatgggcgtgaggaccccgggcggcgtggagatggcctccgagcggaccagacgatggcgatgtgcgtactttaaaaggccaaagagccccatgtctccgctcgcacaccgaggcgccggtctaaaatacgcaggtggcggggcggggccacccgcgcatataagccgggcggt
PRV-delete UL10  ggtgcgcttgtgcagcatgcgcagcaggcgcagcaggcagcgggcggtcgtggtcgcgttgtcctcggtcgtctcctggtagtgcttctccatgagggtgtacatgacgttccacaggtcgatggcgatgggcgtgaggaccccgggcggcgtggagatggcctccgagcggaccagacgatggcgatgtgcgtactttaaaaggccaaagagccccatgtctccgctcgcacaccgaggcgccggtctaaaatacgcaggtggcggggcggggccacccgcgcatataagccgggcggt
                 ############################################################################################################################################################################################################################################################################################################


                      51010     51020     51030     51040     51050     51060     51070     51080     51090     51100     51110     51120     51130     51140     51150     51160     51170     51180     51190     51200     51210     51220     51230     51240     51250     51260     51270     51280     51290     51300
                 =========+=========+=========+=========+=========+=========+=========+=========+=========+=========+=========+=========+=========+=========+=========+=========+=========+=========+=========+=========+=========+=========+=========+=========+=========+=========+=========+=========+=========+=========+
PRV-WT complete  gattggtctggcgcacaccgcccgcccgagcccgctcgcccgccgcgatgtcgctgttcgacgacggcctcgaggacctggaccgccaccccacccacgcgcaccacccggcgcaggtgatccacgacgggcccttcgtgctggaggacggcgagcccctgcagcgcaccggcatgctggtgctcagcgacgagcacctggagcacgcgcgcgccgccatcgccccgctcgccgcgcacctcgcgcacgccttcctcgtcttcagcgaggccgggctgctggtgcacgccagcgtgcgcg
PRV-delete UL10  gattggtctggcgcacaccgcccgcccgagcccgctcgcccgccgcgatgtcgctgttcgacgacggcctcgaggacctggaccgccaccccacccacgcgcaccacccggcgcaggtgatccacgacgggcccttcgtgctggaggacggcgagcccctgcagcgcaccggcatgctggtgctcagcgacgagcacctggagcacgcgcgcgccgccatcgccccgctcgccgcgcacctcgcgcacgccttcctcgtcttcagcgaggccgggctgctggtgcacgccagcgtgcgcg
                 ############################################################################################################################################################################################################################################################################################################


                      51310     51320     51330     51340     51350     51360     51370     51380     51390     51400     51410     51420     51430     51440     51450     51460     51470     51480     51490     51500     51510     51520     51530     51540     51550     51560     51570     51580     51590     51600
                 =========+=========+=========+=========+=========+=========+=========+=========+=========+=========+=========+=========+=========+=========+=========+=========+=========+=========+=========+=========+=========+=========+=========+=========+=========+=========+=========+=========+=========+=========+
PRV-WT complete  gcgagcaggtctacgtgaccctggccccggaccagttcagcacgttcgtgtggagcgggccccaggccgtgttcctgggcaacgtcgacggcagcggcggcgtgctcgacgcgctcaaggtcgaccggcggcggaccgtcttcaacgtcaccttcgaggtgtacggcgccttcccggcgcggctgctgacgcggcgcgcgtactttgcggacgcgggcctcctcgcggcggggcccggctccccgagcgtcgcctgcgtctacaagcacgagttcaacgactactgcatcatgctcccct
PRV-delete UL10  gcgagcaggtctacgtgaccctggccccggaccagttcagcacgttcgtgtggagcgggccccaggccgtgttcctgggcaacgtcgacggcagcggcggcgtgctcgacgcgctcaaggtcgaccggcggcggaccgtcttcaacgtcaccttcgaggtgtacggcgccttcccggcgcggctgctgacgcggcgcgcgtactttgcggacgcgggcctcctcgcggcggggcccggctccccgagcgtcgcctgcgtctacaagcacgagttcaacgactactgcatcatgctcccct
                 ############################################################################################################################################################################################################################################################################################################


                      51610     51620     51630     51640     51650     51660     51670     51680     51690     51700     51710     51720     51730     51740     51750     51760     51770     51780     51790     51800     51810     51820     51830     51840     51850     51860     51870     51880     51890     51900
                 =========+=========+=========+=========+=========+=========+=========+=========+=========+=========+=========+=========+=========+=========+=========+=========+=========+=========+=========+=========+=========+=========+=========+=========+=========+=========+=========+=========+=========+=========+
PRV-WT complete  cgcgggcgcccgacgtgagcctgacgctctcgcgcccccaggtggccaagctcgccgccgtggcgaagggcgccgcggccgggacgaccttcgcgctcgcccgcggcctcgacttctccgtctcctccagcgccggggtcgtgaccttcccggcgcgcgaccacgacgggaccgccgtgctggagcgcgccagccggcggcgccagggcgtcgacgcggtcggcgcgacggagcccttcgccatgacgctcgaggcggcgcacgggctgctgacgctgctgcagcggctgcgggccggga
PRV-delete UL10  cgcgggcgcccgacgtgagcctgacgctctcgcgcccccaggtggccaagctcgccgccgtggcgaagggcgccgcggccgggacgaccttcgcgctcgcccgcggcctcgacttctccgtctcctccagcgccggggtcgtgaccttcccggcgcgcgaccacgacgggaccgccgtgctggagcgcgccagccggcggcgccagggcgtcgacgcggtcggcgcgacggagcccttcgccatgacgctcgaggcggcgcacgggctgctgacgctgctgcagcggctgcgggccggga
                 ############################################################################################################################################################################################################################################################################################################


                      51910     51920     51930     51940     51950     51960     51970     51980     51990     52000     52010     52020     52030     52040     52050     52060     52070     52080     52090     52100     52110     52120     52130     52140     52150     52160     52170     52180     52190     52200
                 =========+=========+=========+=========+=========+=========+=========+=========+=========+=========+=========+=========+=========+=========+=========+=========+=========+=========+=========+=========+=========+=========+=========+=========+=========+=========+=========+=========+=========+=========+
PRV-WT complete  acgccgagctcacgttcaactttttcacgacgccgcggcaggcgcccctgttcagcgtgaccacctgcggcccggtgcgggcgaccaccttcttcttctgcgcgcccgccgaccccgccaccgtgcccgccgcccccgagggcgccgccgccaccgtcgccgccgcctgcggggcgggcgcgtccgccgcctcccccgccgcgggggacaagcggcccgccgccccgcgcatgtacacgcccatcgccaagcgcccgcggaccgcctcgggggaagggggccacgcctacggagatttat
PRV-delete UL10  acgccgagctcacgttcaactttttcacgacgccgcggcaggcgcccctgttcagcgtgaccacctgcggcccggtgcgggcgaccaccttcttcttctgcgcgcccgccgaccccgccaccgtgcccgccgcccccgagggcgccgccgccaccgtcgccgccgcctgcggggcgggcgcgtccgccgcctcccccgccgcgggggacaagcggcccgccgccccgcgcatgtacacgcccatcgccaagcgcccgcggaccgcctcgggggaagggggccacgcctacggagatttat
                 ############################################################################################################################################################################################################################################################################################################


                      52210     52220     52230     52240     52250     52260     52270     52280     52290     52300     52310     52320     52330     52340     52350     52360     52370     52380     52390     52400     52410     52420     52430     52440     52450     52460     52470     52480     52490     52500
                 =========+=========+=========+=========+=========+=========+=========+=========+=========+=========+=========+=========+=========+=========+=========+=========+=========+=========+=========+=========+=========+=========+=========+=========+=========+=========+=========+=========+=========+=========+
PRV-WT complete  tctaataaagtgagcaggtgtataaaagagaggtcgcacctccgcgtctttactcgccgtcgcgatgatctcccgggggaacggagggggttgccgccccggcgagccatgctggcgctgcgcgctcgagtccacccgctgcatcacgctgatgggcgtgctcgtcgcgctcctcgccgcctgcatgctgtccgtcccgcccgcggcgtcgacgatgctgctcggcgtcgccagcctcatggccatgctgcgcctgcccatgcccctcgtggaccggttcatccccgcgtgcatgggcct
PRV-delete UL10  tctaataaagtgagcaggtgtataaaagagaggtcgcacctccgcgtctttactcgccgtcgcgatgatctcccgggggaacggagggggttgccgccccggcgagccatgctggcgctgcgcgctcgagtccacccgctgcatcacgctgatgggcgtgctcgtcgcgctcctcgccgcctgcatgctgtccgtcccgcccgcggcgtcgacgatgctgctcggcgtcgccagcctcatggccatgctgcgcctgcccatgcccctcgtggaccggttcatccccgcgtgcatgggcct
                 ############################################################################################################################################################################################################################################################################################################


                      52510     52520     52530     52540     52550     52560     52570     52580     52590     52600     52610     52620     52630     52640     52650     52660     52670     52680     52690     52700     52710     52720     52730     52740     52750     52760     52770     52780     52790     52800
                 =========+=========+=========+=========+=========+=========+=========+=========+=========+=========+=========+=========+=========+=========+=========+=========+=========+=========+=========+=========+=========+=========+=========+=========+=========+=========+=========+=========+=========+=========+
PRV-WT complete  ccagctcgtcggcgccgccgtcttcgccgcgggctgggcgctggccagccgcgacgcgatctcggccggcgtcctgctgtgggccgtctgcgcgctcatctcgcacatgtacaacgtcgtctgcgtcgccagcgggcccgacgcgcactaccgccccgcctgcctcgtcatgggcgtcgccgcggcctgcggcgcggcgggggccctcgtgaacgtgcgcaccgaggcgcgcctcggcatcgccctggggctggccgtcacgtgcgccacgaacaacgtcgcccgcagcctgcgcggcac
PRV-delete UL10  ccagctcgtcggcgccgccgtcttcgccgcgggctgggcgctggccagccgcgacgcgatctcggccggcgtcctgctgtgggccgtctgcgcgctcatctcgcacatgtacaacgtcgtctgcgtcgccagcgggcccgacgcgcactaccgccccgcctgcctcgtcatgggcgtcgccgcggcctgcggcgcggcgggggccctcgtgaacgtgcgcaccgaggcgcgcctcggcatcgccctggggctggccgtcacgtgcgccacgaacaacgtcgcccgcagcctgcgcggcac
                 ############################################################################################################################################################################################################################################################################################################


                      52810     52820     52830     52840     52850     52860     52870     52880     52890     52900     52910     52920     52930     52940     52950     52960     52970     52980     52990     53000     53010     53020     53030     53040     53050     53060     53070     53080     53090     53100
                 =========+=========+=========+=========+=========+=========+=========+=========+=========+=========+=========+=========+=========+=========+=========+=========+=========+=========+=========+=========+=========+=========+=========+=========+=========+=========+=========+=========+=========+=========+
PRV-WT complete  gtgcacctacgtcgccagccgcgcccggttcctggccgcgcccgcggacctggggcgcggctacagcgtggagaacgcggacgccgaccccaccgccgagcccgagcggcgcgtctacgaggcgaccgtcccgcccacccacgtctacgccggcagcatcgcgctcttcgccctcgtcttctcggccgcctcctccctccagtggatggtctcccagatggtgggccgcggcaaccagctcgtctcgcccaccaccgcggccgcggccggcgccgccgggttcctggacgcggcggcgct
PRV-delete UL10  gtgcacctacgtcgccagccgcgcccggttcctggccgcgcccgcggacctggggcgcggctacagcgtggagaacgcggacgccgaccccaccgccgagcccgagcggcgcgtctacgaggcgaccgtcccgcccacccacgtctacgccggcagcatcgcgctcttcgccctcgtcttctcggccgcctcctccctccagtggatggtctcccagatggtgggccgcggcaaccagctcgtctcgcccaccaccgcggccgcggccggcgccgccgggttcctggacgcggcggcgct
                 ############################################################################################################################################################################################################################################################################################################


                      53110     53120     53130     53140     53150     53160     53170     53180     53190     53200     53210     53220     53230     53240     53250     53260     53270     53280     53290     53300     53310     53320     53330     53340     53350     53360     53370     53380     53390     53400
                 =========+=========+=========+=========+=========+=========+=========+=========+=========+=========+=========+=========+=========+=========+=========+=========+=========+=========+=========+=========+=========+=========+=========+=========+=========+=========+=========+=========+=========+=========+
PRV-WT complete  ctcgctctttgtgcgcccgagcacgcgccacctctcggtggccgtcaagggcgcgcacgcgctcctcatcctcgcggccatcgtcctcacggccgccggcgagcccatgggcgtgcccatcagcctcgcggcctccaccggcctcgcgctgctcgaggcggaccacgtccgcctgcgccacacccgcgcgtaccggctcgccgccgcgcacgtgacgcgggccctgctcgtgcaggcgtacgtgaccgtcgccatgtgtgccactagcattaaatccgtttcctgattcacgcccacgcc
PRV-delete UL10  ctcgctctttgtgcgcccgagcacgcgccacctctcggtggccgtcaagggcgcgcacgcgctcctcatcctcgcggccatcgtcctcacggccgccggcgagcccatgggcgtgcccatcagcctcgcggcctccaccggcctcgcgctgctcgaggcggaccacgtccgcctgcgccacacccgcgcgtaccggctcgccgccgcgcacgtgacgcgggccctgctcgtgcaggcgtacgtgaccgtcgccatgtgtgccactagcattaaatccgtttcctgattcacgcccacgcc
                 ############################################################################################################################################################################################################################################################################################################


                      53410     53420     53430     53440     53450     53460     53470     53480     53490     53500     53510     53520     53530     53540     53550     53560     53570     53580     53590     53600     53610     53620     53630     53640     53650     53660     53670     53680     53690     53700
                 =========+=========+=========+=========+=========+=========+=========+=========+=========+=========+=========+=========+=========+=========+=========+=========+=========+=========+=========+=========+=========+=========+=========+=========+=========+=========+=========+=========+=========+=========+
PRV-WT complete  cgtgtcgtttttaaaaccgcgatggggggacggggggccattcgcacgcgccatggcctcgctcgcgcgtgcgatgctcgcgctgctggcgctctacacggcggccatcgccgcggcgccgtcgtccacgacggcgctcggcacgacgcccaacgggggcgggggcggcaacagcagcgcgggcgagctctcgccctcgccgccctcgacgcccgagcccgtctcggggacgacgggggccgcggcctccacgcccgccgccgtctcgacgccccgggtcccgccgccctcggtctcgcg
PRV-delete UL10  cgtgtcgtttttaaaaccgcgatggggggacggggggccattcgcacgcgccatggcctcgctcgcgcgtgcgatgctcgcgctgctggcgctctacacggcggccatcgccgcggcgccgtcgtccacgacggcgctcggcacgacgcccaacgggggcgggggcggcaacagcagcgcgggcgagctctcgccctcgccgccctcgacgcccgagcccgtctcggggacgacgggggccgcggcctccacgcccgccgccgtctcgacgccccgggtcccgccgccctcggtctcgcg
                 ############################################################################################################################################################################################################################################################################################################


                      53710     53720     53730     53740     53750     53760     53770     53780     53790     53800     53810     53820     53830     53840     53850     53860     53870     53880     53890     53900     53910     53920     53930     53940     53950     53960     53970     53980     53990     54000
                 =========+=========+=========+=========+=========+=========+=========+=========+=========+=========+=========+=========+=========+=========+=========+=========+=========+=========+=========+=========+=========+=========+=========+=========+=========+=========+=========+=========+=========+=========+
PRV-WT complete  ccggaagccccagcggaacggcaacaggacgcgcgtccacggcgacaaggccacctcgcacgggcgcaagcgcatcgtgtgccgcgagcggctgttctcggcgagggtgggggacgcggtcagcttcgggtgcgccgtcgtcccgcgcgccggggagaccttcgaggtccgcttctgccgccgcgggcgcttccgctcgcccgacgccgaccccgagtactttgacgagcccccgcgcccggagctcccgcgggagcggctcctcttcagctccgccaacgcctccctcgcccacgcgga
PRV-delete UL10  ccggaagccccagcggaacggcaacaggacgcgcgtccacggcgacaaggccacctcgcacgggcgcaagcgcatcgtgtgccgcgagcggctgttctcggcgagggtgggggacgcggtcagcttcgggtgcgccgtcgtcccgcgcgccggggagaccttcgaggtccgcttctgccgccgcgggcgcttccgctcgcccgacgccgaccccgagtactttgacgagcccccgcgcccggagctcccgcgggagcggctcctcttcagctccgccaacgcctccctcgcccacgcgga
                 ############################################################################################################################################################################################################################################################################################################


                      54010     54020     54030     54040     54050     54060     54070     54080     54090     54100     54110     54120     54130     54140     54150     54160     54170     54180     54190     54200     54210     54220     54230     54240     54250     54260     54270     54280     54290     54300
                 =========+=========+=========+=========+=========+=========+=========+=========+=========+=========+=========+=========+=========+=========+=========+=========+=========+=========+=========+=========+=========+=========+=========+=========+=========+=========+=========+=========+=========+=========+
PRV-WT complete  cgcgctcgcctccgccgtcgtcgtcgagggcgagcgcgcgaccgtcgccaacgtctcgggcgaggtgtccgtgcgcgtggccgcggcggacgccgagaccgagggcgtctacacgtggcgcgtgctgtccgccaacggcaccgaggtccgcagcgccaacgtctcgctcgtcctgtaccaccagcccgagttcggcctgagcgcgccgcccgtcctcttcggcgagcccttccgggcggtgtgcgtcgtccgcgactactacccgcggcgcagcgtgcgcctgcgctggttcgcggacga
PRV-delete UL10  cgcgctcgcctccgccgtcgtcgtcgagggcgagcgcgcgaccgtcgccaacgtctcgggcgaggtgtccgtgcgcgtggccgcggcggacgccgagaccgagggcgtctacacgtggcgcgtgctgtccgccaacggcaccgaggtccgcagcgccaacgtctcgctcgtcctgtaccaccagcccgagttcggcctgagcgcgccgcccgtcctcttcggcgagcccttccgggcggtgtgcgtcgtccgcgactactacccgcggcgcagcgtgcgcctgcgctggttcgcggacga
                 ############################################################################################################################################################################################################################################################################################################


                      54310     54320     54330     54340     54350     54360     54370     54380     54390     54400     54410     54420     54430     54440     54450     54460     54470     54480     54490     54500     54510     54520     54530     54540     54550     54560     54570     54580     54590     54600
                 =========+=========+=========+=========+=========+=========+=========+=========+=========+=========+=========+=========+=========+=========+=========+=========+=========+=========+=========+=========+=========+=========+=========+=========+=========+=========+=========+=========+=========+=========+
PRV-WT complete  gcacccggtggacgccgccttcgtgaccaacagcaccgtggccgacgagctcgggcgccgcacgcgcgtctccgtggtgaacgtgacgcgcgcggacgtcccgggcctcgcggccgcggacgacgcggacgcgctcgcgccgagcctgcgctgcgaggccgtgtggtaccgcgacagcgtggcctcgcagcgcttctccgaggccctgcgcccccacgtctaccacccggcggcggtctcggtgcgcttcgtcgagggcttcgccgtctgcgacggcctctgcgtgcccccggaggcgcg
PRV-delete UL10  gcacccggtggacgccgccttcgtgaccaacagcaccgtggccgacgagctcgggcgccgcacgcgcgtctccgtggtgaacgtgacgcgcgcggacgtcccgggcctcgcggccgcggacgacgcggacgcgctcgcgccgagcctgcgctgcgaggccgtgtggtaccgcgacagcgtggcctcgcagcgcttctccgaggccctgcgcccccacgtctaccacccggcggcggtctcggtgcgcttcgtcgagggcttcgccgtctgcgacggcctctgcgtgcccccggaggcgcg
                 ############################################################################################################################################################################################################################################################################################################


                      54610     54620     54630     54640     54650     54660     54670     54680     54690     54700     54710     54720     54730     54740     54750     54760     54770     54780     54790     54800     54810     54820     54830     54840     54850     54860     54870     54880     54890     54900
                 =========+=========+=========+=========+=========+=========+=========+=========+=========+=========+=========+=========+=========+=========+=========+=========+=========+=========+=========+=========+=========+=========+=========+=========+=========+=========+=========+=========+=========+=========+
PRV-WT complete  cctcgcctggtccgaccacgccgccgacaccgtctaccacctcggcgcctgcgccgagcaccccggcctgctcaacgtgcggagcgcccgcccgctgtcggacctcgacgggcccgtcgactacacctgccgcctcgagggcatgccctcgcagctgcccatcttcgaggacacgcagcgctacgacgcctcccccacgtccgtgagctggcccgtcgtgaccagcatgatcaccgtcatcgccggcatcgccatcctagccatcgtgctggtcatcatggcgacgtgcgtctactaccg
PRV-delete UL10  cctcgcctggtccgaccacgccgccgacaccgtctaccacctcggcgcctgcgccgagcaccccggcctgctcaacgtgcggagcgcccgcccgctgtcggacctcgacgggcccgtcgactacacctgccgcctcgagggcatgccctcgcagctgcccatcttcgaggacacgcagcgctacgacgcctcccccacgtccgtgagctggcccgtcgtgaccagcatgatcaccgtcatcgccggcatcgccatcctagccatcgtgctggtcatcatggcgacgtgcgtctactaccg
                 ############################################################################################################################################################################################################################################################################################################


                      54910     54920     54930     54940     54950     54960     54970     54980     54990     55000     55010     55020     55030     55040     55050     55060     55070     55080     55090     55100     55110     55120     55130     55140     55150     55160     55170     55180     55190     55200
                 =========+=========+=========+=========+=========+=========+=========+=========+=========+=========+=========+=========+=========+=========+=========+=========+=========+=========+=========+=========+=========+=========+=========+=========+=========+=========+=========+=========+=========+=========+
PRV-WT complete  ccggtccgcgctgtgacgcccccgcccgccccgaatcaataaacgaccgcgcgttgaaccgagccatcgcgtccggttgtttgtttgtgcgtgtgtgtgtgagctctcgcgcgtccccgggccccgctctctcccgtcccactccactctcctctcccgcaccctccccacacacacgcccggcccgcaataaaccacacgagacgacggcgtgttgtaccggcgcgaccgggccatctctttattggtcgtcggggaacagggacctggccggggtggggggtgtgggcggcgggcgag
PRV-delete UL10  ccggtccgcgctgtgacgcccccgcccgccccgaatcaataaacgaccgcgcgttgaaccgagccatcgcgtccggttgtttgtttgtgcgtgtgtgtgtgagctctcgcgcgtccccgggccccgctctctcccgtcccactccactctcctctcccgcaccctccccacacacacgcccggcccgcaataaaccacacgagacgacggcgtgttgtaccggcgcgaccgggccatctctttattggtcgtcggggaacagggacctggccggggtggggggtgtgggcggcgggcgag
                 ############################################################################################################################################################################################################################################################################################################


                      55210     55220     55230     55240     55250     55260     55270     55280     55290     55300     55310     55320     55330     55340     55350     55360     55370     55380     55390     55400     55410     55420     55430     55440     55450     55460     55470     55480     55490     55500
                 =========+=========+=========+=========+=========+=========+=========+=========+=========+=========+=========+=========+=========+=========+=========+=========+=========+=========+=========+=========+=========+=========+=========+=========+=========+=========+=========+=========+=========+=========+
PRV-WT complete  cgacgccgtcctcatcggtgggacatcatctgggagacgaagatgtcggcgccgcgctggcaggccacggcggccgagctggcgtcgatggtgccggcggagacgtctgccggggccgagaccgaggcctggacgggcggctggggggccgaggcgggcgcggccggggcggcggcctgaacgggggcggccgcggcggccgccgctgctggagccggagcgggggcggccgcggcggcggcggcggcgggcacggccaccggctgggcctgcaccaccaccggctgctgcgggaccacc
PRV-delete UL10  cgacgccgtcctcatcggtgggacatcatctgggagacgaagatgtcggcgccgcgctggcaggccacggcggccgagctggcgtcgatggtgccggcggagacgtctgccggggccgagaccgaggcctggacgggcggctggggggccgaggcgggcgcggccggggcggcggcctgaacgggggcggccgcggcggccgccgctgctggagccggagcgggggcggccgcggcggcggcggcggcgggcacggccaccggctgggcctgcaccaccaccggctgctgcgggaccacc
                 ############################################################################################################################################################################################################################################################################################################


                      55510     55520     55530     55540     55550     55560     55570     55580     55590     55600     55610     55620     55630     55640     55650     55660     55670     55680     55690     55700     55710     55720     55730     55740     55750     55760     55770     55780     55790     55800
                 =========+=========+=========+=========+=========+=========+=========+=========+=========+=========+=========+=========+=========+=========+=========+=========+=========+=========+=========+=========+=========+=========+=========+=========+=========+=========+=========+=========+=========+=========+
PRV-WT complete  tgggcggggacgtgcgcgacgggggccgccagaccctggaggcccggcaggcccgggagcgccggcacggcgtgcgccacgggcgaggggggcggcgcgtgcagccccggctggtgggcgtacatcggcgggtacagcgccccggcggcgtggtgcgggtgctggtgcgcgtgtcccacgacggccccggggggcaggccgggcggcagcagctgcggcggcgtcgcgggcgcgtagtacggcgtcgggtagcggagctgctgggcgcgcaggtggctgatctcgcgctggagcgacgag
PRV-delete UL10  tgggcggggacgtgcgcgacgggggccgccagaccctggaggcccggcaggcccgggagcgccggcacggcgtgcgccacgggcgaggggggcggcgcgtgcagccccggctggtgggcgtacatcggcgggtacagcgccccggcggcgtggtgcgggtgctggtgcgcgtgtcccacgacggccccggggggcaggccgggcggcagcagctgcggcggcgtcgcgggcgcgtagtacggcgtcgggtagcggagctgctgggcgcgcaggtggctgatctcgcgctggagcgacgag
                 ############################################################################################################################################################################################################################################################################################################


                      55810     55820     55830     55840     55850     55860     55870     55880     55890     55900     55910     55920     55930     55940     55950     55960     55970     55980     55990     56000     56010     56020     56030     56040     56050     56060     56070     56080     56090     56100
                 =========+=========+=========+=========+=========+=========+=========+=========+=========+=========+=========+=========+=========+=========+=========+=========+=========+=========+=========+=========+=========+=========+=========+=========+=========+=========+=========+=========+=========+=========+
PRV-WT complete  accacggcggcgaggtcgctcgcggggggcggcgcctccccggggtagtacgcgttgtcctgcgcgtagtcgtcgtagcggcgcttccgggcggggggccgcggctcgggcgagggcgagcggcggtggcggcggtccatggcggccgagacgatggcgccgagctgggactccaggctgggcgccgggcgctggttcaccaccagctggttgtactgcgccgcgggcacgaagatgtagtcgccgggcgctgcgctcggcggggctgccatggccgcactgggcccgggcgccggggag
PRV-delete UL10  accacggcggcgaggtcgctcgcggggggcggcgcctccccggggtagtacgcgttgtcctgcgcgtagtcgtcgtagcggcgcttccgggcggggggccgcggctcgggcgagggcgagcggcggtggcggcggtccatggcggccgagacgatggcgccgagctgggactccaggctgggcgccgggcgctggttcaccaccagctggttgtactgcgccgcgggcacgaagatgtagtcgccgggcgctgcgctcggcggggctgccatggccgcactgggcccgggcgccggggag
                 ############################################################################################################################################################################################################################################################################################################


                      56110     56120     56130     56140     56150     56160     56170     56180     56190     56200     56210     56220     56230     56240     56250     56260     56270     56280     56290     56300     56310     56320     56330     56340     56350     56360     56370     56380     56390     56400
                 =========+=========+=========+=========+=========+=========+=========+=========+=========+=========+=========+=========+=========+=========+=========+=========+=========+=========+=========+=========+=========+=========+=========+=========+=========+=========+=========+=========+=========+=========+
PRV-WT complete  gccccgcttttaggcagaagccccgcccacatcgtcgcctgcaggtaggtgtgtccgcgcacgccggcctcgcggcggcgcgccgcgaccagctcccagcggtcgcgcagcagcatgttgttcacggccgtcgacagcagcgcgcgcgtgagcgcctcctcgctcatgtgccacacgcgctcgcggtccggcgactcggcggcgcgcgccagcagctcggagcgcgcccgcgccgagagctgccggaacggcgccacggcggcctcgggcgaggcgtcgtacaccacgatggtgcccacg
PRV-delete UL10  gccccgcttttaggcagaagccccgcccacatcgtcgcctgcaggtaggtgtgtccgcgcacgccggcctcgcggcggcgcgccgcgaccagctcccagcggtcgcgcagcagcatgttgttcacggccgtcgacagcagcgcgcgcgtgagcgcctcctcgctcatgtgccacacgcgctcgcggtccggcgactcggcggcgcgcgccagcagctcggagcgcgcccgcgccgagagctgccggaacggcgccacggcggcctcgggcgaggcgtcgtacaccacgatggtgcccacg
                 ############################################################################################################################################################################################################################################################################################################


                      56410     56420     56430     56440     56450     56460     56470     56480     56490     56500     56510     56520     56530     56540     56550     56560     56570     56580     56590     56600     56610     56620     56630     56640     56650     56660     56670     56680     56690     56700
                 =========+=========+=========+=========+=========+=========+=========+=========+=========+=========+=========+=========+=========+=========+=========+=========+=========+=========+=========+=========+=========+=========+=========+=========+=========+=========+=========+=========+=========+=========+
PRV-WT complete  cgccgcccgatcacgcagagcgccacgtgcgcaaagagcgtctcgtcgggggcctcgccgggcgccaggcgccgcgaggacagggacgccgagggcaggtagttgctcagcaggtacagcagccgctcctcgtccgagagccgcatgtccccgaagaagtccgggcccacggcgcgggcgagcacggcccccagctgcgggcagttgacgacgccgaggaagaaggggccgcggtcgtcgtcgaccacggccagcacggcgccgatgtcgcagcgcgggcggtggtcgatgttgatgggc
PRV-delete UL10  cgccgcccgatcacgcagagcgccacgtgcgcaaagagcgtctcgtcgggggcctcgccgggcgccaggcgccgcgaggacagggacgccgagggcaggtagttgctcagcaggtacagcagccgctcctcgtccgagagccgcatgtccccgaagaagtccgggcccacggcgcgggcgagcacggcccccagctgcgggcagttgacgacgccgaggaagaaggggccgcggtcgtcgtcgaccacggccagcacggcgccgatgtcgcagcgcgggcggtggtcgatgttgatgggc
                 ############################################################################################################################################################################################################################################################################################################


                      56710     56720     56730     56740     56750     56760     56770     56780     56790     56800     56810     56820     56830     56840     56850     56860     56870     56880     56890     56900     56910     56920     56930     56940     56950     56960     56970     56980     56990     57000
                 =========+=========+=========+=========+=========+=========+=========+=========+=========+=========+=========+=========+=========+=========+=========+=========+=========+=========+=========+=========+=========+=========+=========+=========+=========+=========+=========+=========+=========+=========+
PRV-WT complete  agcgggcccgccgggggcagcgcggcggccacgacctcgcgcgtcagcgcgagctccccgccgtccctatcgtacagggccaggtagccggacacgtacacgggccccatggcggcggcggtggcgacggcggcggcgcgcgcgcggaggagactgcgggctcaggcggcggcgaactgcgggatatagcccaggcagagaaagtacaggaggtcgtagtcgctggagacgccaaactgcccgaggcgcttgtccgccgcgttgcgcaggtgcggctgctgcagcagcaggcccagcccc
PRV-delete UL10  agcgggcccgccgggggcagcgcggcggccacgacctcgcgcgtcagcgcgagctccccgccgtccctatcgtacagggccaggtagccggacacgtacacgggccccatggcggcggcggtggcgacggcggcggcgcgcgcgcggaggagactgcgggctcaggcggcggcgaactgcgggatatagcccaggcagagaaagtacaggaggtcgtagtcgctggagacgccaaactgcccgaggcgcttgtccgccgcgttgcgcaggtgcggctgctgcagcagcaggcccagcccc
                 ############################################################################################################################################################################################################################################################################################################


                      57010     57020     57030     57040     57050     57060     57070     57080     57090     57100     57110     57120     57130     57140     57150     57160     57170     57180     57190     57200     57210     57220     57230     57240     57250     57260     57270     57280     57290     57300
                 =========+=========+=========+=========+=========+=========+=========+=========+=========+=========+=========+=========+=========+=========+=========+=========+=========+=========+=========+=========+=========+=========+=========+=========+=========+=========+=========+=========+=========+=========+
PRV-WT complete  tcctcgtactgcacggccacggcgtcgtgcgccgcgatgacctcgttgacgggggccgtcacgcgctggcggttctgcagctccagcgccaccaggcgcaccaggctgagctggtggcgctgcacgcgcgcgcggatcgagtgcgcgtccagcgccagcgcggccagcccggggaagagctgcgtgagctccgtgcgctcgtccgcgcggtaggccacgacgacgtagtgggccagcaggaagcgcaggttgccgtcgccgctgcgcgaggcgggcgcgtccccgctggcgccgcgcggc
PRV-delete UL10  tcctcgtactgcacggccacggcgtcgtgcgccgcgatgacctcgttgacgggggccgtcacgcgctggcggttctgcagctccagcgccaccaggcgcaccaggctgagctggtggcgctgcacgcgcgcgcggatcgagtgcgcgtccagcgccagcgcggccagcccggggaagagctgcgtgagctccgtgcgctcgtccgcgcggtaggccacgacgacgtagtgggccagcaggaagcgcaggttgccgtcgccgctgcgcgaggcgggcgcgtccccgctggcgccgcgcggc
                 ############################################################################################################################################################################################################################################################################################################


                      57310     57320     57330     57340     57350     57360     57370     57380     57390     57400     57410     57420     57430     57440     57450     57460     57470     57480     57490     57500     57510     57520     57530     57540     57550     57560     57570     57580     57590     57600
                 =========+=========+=========+=========+=========+=========+=========+=========+=========+=========+=========+=========+=========+=========+=========+=========+=========+=========+=========+=========+=========+=========+=========+=========+=========+=========+=========+=========+=========+=========+
PRV-WT complete  ggcggcgcgttgggcaccagcgcgccgagctggaagttgttgcgcaggcggtccccgtacacgttgccgttgtgcagcaggcgccgcagcagcagcaggcccgtgatggtgttcaccgtggcgcgcagcagcggcgcgtcctcgccgaggaagaggttctggccgcggcccaggaaggcggccgcggcgcggttgacctcgtccacgaagcccgcgccgtcgtcgacctcgttggcgccgacgttcgcgccggggaccacgcgcaggcgcgccagcgcggcgagcacgctgtggccctcc
PRV-delete UL10  ggcggcgcgttgggcaccagcgcgccgagctggaagttgttgcgcaggcggtccccgtacacgttgccgttgtgcagcaggcgccgcagcagcagcaggcccgtgatggtgttcaccgtggcgcgcagcagcggcgcgtcctcgccgaggaagaggttctggccgcggcccaggaaggcggccgcggcgcggttgacctcgtccacgaagcccgcgccgtcgtcgacctcgttggcgccgacgttcgcgccggggaccacgcgcaggcgcgccagcgcggcgagcacgctgtggccctcc
                 ############################################################################################################################################################################################################################################################################################################


                      57610     57620     57630     57640     57650     57660     57670     57680     57690     57700     57710     57720     57730     57740     57750     57760     57770     57780     57790     57800     57810     57820     57830     57840     57850     57860     57870     57880     57890     57900
                 =========+=========+=========+=========+=========+=========+=========+=========+=========+=========+=========+=========+=========+=========+=========+=========+=========+=========+=========+=========+=========+=========+=========+=========+=========+=========+=========+=========+=========+=========+
PRV-WT complete  agcgcgtagcgcccgtggagctgctcgcgcggcacgcgcgcgtagttgtagcggtacgagatgcgcccgccctcggccagcgcctggctcagcgcgtccaggtactcggggatccgctccagcaggtccacgatgcccagcggcgcgggcgccgccgccgcggcggcggggccctgccgcgcgcggtgcaccaggtgcaggcacagcgcggcgctctcgagcgccgagtagtgccggttgcccacgtacaggcgcccgcaggcctgcagcgccgtggtcgtcacgcccatgaaggtgcgc
PRV-delete UL10  agcgcgtagcgcccgtggagctgctcgcgcggcacgcgcgcgtagttgtagcggtacgagatgcgcccgccctcggccagcgcctggctcagcgcgtccaggtactcggggatccgctccagcaggtccacgatgcccagcggcgcgggcgccgccgccgcggcggcggggccctgccgcgcgcggtgcaccaggtgcaggcacagcgcggcgctctcgagcgccgagtagtgccggttgcccacgtacaggcgcccgcaggcctgcagcgccgtggtcgtcacgcccatgaaggtgcgc
                 ############################################################################################################################################################################################################################################################################################################


                      57910     57920     57930     57940     57950     57960     57970     57980     57990     58000     58010     58020     58030     58040     58050     58060     58070     58080     58090     58100     58110     58120     58130     58140     58150     58160     58170     58180     58190     58200
                 =========+=========+=========+=========+=========+=========+=========+=========+=========+=========+=========+=========+=========+=========+=========+=========+=========+=========+=========+=========+=========+=========+=========+=========+=========+=========+=========+=========+=========+=========+
PRV-WT complete  gacatgcggccgtcgcgcgagtcgatggcgcgcgagacctgcgggcgctccgcgacgagcgtggcctggagcgtggcgtaccacgtgccgaagagcacgccggaggcggcgccgcgcgcggcgtacaccatggccaggaagtccagcgagaggttggcgtcgtaggcgagcgccacgtcgttcttggcgatctgcacctcgcggccctcgtccgcggccgcgaccgcctcgggcgcctcctcggcggcgcgcgccgcgtccgcctcctcggcggcgcgcgccgtctcctcgagcaccacg
PRV-delete UL10  gacatgcggccgtcgcgcgagtcgatggcgcgcgagacctgcgggcgctccgcgacgagcgtggcctggagcgtggcgtaccacgtgccgaagagcacgccggaggcggcgccgcgcgcggcgtacaccatggccaggaagtccagcgagaggttggcgtcgtaggcgagcgccacgtcgttcttggcgatctgcacctcgcggccctcgtccgcggccgcgaccgcctcgggcgcctcctcggcggcgcgcgccgcgtccgcctcctcggcggcgcgcgccgtctcctcgagcaccacg
                 ############################################################################################################################################################################################################################################################################################################


                      58210     58220     58230     58240     58250     58260     58270     58280     58290     58300     58310     58320     58330     58340     58350     58360     58370     58380     58390     58400     58410     58420     58430     58440     58450     58460     58470     58480     58490     58500
                 =========+=========+=========+=========+=========+=========+=========+=========+=========+=========+=========+=========+=========+=========+=========+=========+=========+=========+=========+=========+=========+=========+=========+=========+=========+=========+=========+=========+=========+=========+
PRV-WT complete  agcgcctcggccacgcgctccacctgccgctccagcggccgcagctgctcgtccacctcggcctcgaggcgcgcgcccgcggccatggcgttgtccagcgccgcggcggccgcgcggcggcgcgcgttcgcgtgcgccagcgcgaggcgcgcgtcgaggccctcgccgaagcccgggcgggcccagaagcccacggggaacgggggcgcgatgaagtggcgcgcgctgcccgggatcgcagcggcctcgaaggcgaaccacgcgcggtccatggcgcggggggacatgggccgcgcggcg
PRV-delete UL10  agcgcctcggccacgcgctccacctgccgctccagcggccgcagctgctcgtccacctcggcctcgaggcgcgcgcccgcggccatggcgttgtccagcgccgcggcggccgcgcggcggcgcgcgttcgcgtgcgccagcgcgaggcgcgcgtcgaggccctcgccgaagcccgggcgggcccagaagcccacggggaacgggggcgcgatgaagtggcgcgcgctgcccgggatcgcagcggcctcgaaggcgaaccacgcgcggtccatggcgcggggggacatgggccgcgcggcg
                 ############################################################################################################################################################################################################################################################################################################


                      58510     58520     58530     58540     58550     58560     58570     58580     58590     58600     58610     58620     58630     58640     58650     58660     58670     58680     58690     58700     58710     58720     58730     58740     58750     58760     58770     58780     58790     58800
                 =========+=========+=========+=========+=========+=========+=========+=========+=========+=========+=========+=========+=========+=========+=========+=========+=========+=========+=========+=========+=========+=========+=========+=========+=========+=========+=========+=========+=========+=========+
PRV-WT complete  gcgcgcgccgccttatcatccccgctccccgccgccgcccggcccggccccgcgcgcgccgcgatcgcgatcaccgccgcggcccggcgacgtactcggcgaggccgcgcacggtcgcggccatcgcgctcgcgttgccgcgcgtctgggtgcagggcaggcgcgtcacgtcgagcacgcgcatgctccgctgggccacaaacaccagcaggggcacgagcgtgatctcctcgccgcccgggggcacggcggcggcgaggaggcgcgccgagtcgcgcagctggcacagcccctcgtgcc
PRV-delete UL10  gcgcgcgccgccttatcatccccgctccccgccgccgcccggcccggccccgcgcgcgccgcgatcgcgatcaccgccgcggcccggcgacgtactcggcgaggccgcgcacggtcgcggccatcgcgctcgcgttgccgcgcgtctgggtgcagggcaggcgcgtcacgtcgagcacgcgcatgctccgctgggccacaaacaccagcaggggcacgagcgtgatctcctcgccgcccgggggcacggcggcggcgaggaggcgcgccgagtcgcgcagctggcacagcccctcgtgcc
                 ############################################################################################################################################################################################################################################################################################################


                      58810     58820     58830     58840     58850     58860     58870     58880     58890     58900     58910     58920     58930     58940     58950     58960     58970     58980     58990     59000     59010     59020     59030     59040     59050     59060     59070     59080     59090     59100
                 =========+=========+=========+=========+=========+=========+=========+=========+=========+=========+=========+=========+=========+=========+=========+=========+=========+=========+=========+=========+=========+=========+=========+=========+=========+=========+=========+=========+=========+=========+
PRV-WT complete  gctgcccgcgcttgctgggcgtgttgaggttccgggggaagcggcacgtcttgagctcgatgaggaagcacaggtgcgggcccgcccccagccgcaccacgcacacgcagtcggggcggcgcaccccgaggttgacttcaaaggccagggtcaaggacgccttcttaagcgtctctcggggaagcccgaagagactctcgccgtacgcggacgggtcgcgtcgcaggcgttcgtagaagcggttgtggcagcggatccccgcccggaagcgcgccgggatgcgcatcctccggatctacc
PRV-delete UL10  gctgcccgcgcttgctgggcgtgttgaggttccgggggaagcggcacgtcttgagctcgatgaggaagcacaggtgcgggcccgcccccagccgcaccacgcacacgcagtcggggcggcgcaccccgaggttgacttcaaaggccagggtcaaggacgccttcttaagcgtctctcggggaagcccgaagagactctcgccgtacgcggacgggtcgcgtcgcaggcgttcgtagaagcggttgtggcagcggatccccgcccggaagcgcgccgggatgcgcatcctccggatctacc
                 ############################################################################################################################################################################################################################################################################################################


                      59110     59120     59130     59140     59150     59160     59170     59180     59190     59200     59210     59220     59230     59240     59250     59260     59270     59280     59290     59300     59310     59320     59330     59340     59350     59360     59370     59380     59390     59400
                 =========+=========+=========+=========+=========+=========+=========+=========+=========+=========+=========+=========+=========+=========+=========+=========+=========+=========+=========+=========+=========+=========+=========+=========+=========+=========+=========+=========+=========+=========+
PRV-WT complete  tcgacggcgcctacggcaccggcaagagcaccacggcccgggtgatggcgctcggcggggcgctgtacgtgcccgagccgatggcgtactggcgcactctgttcgacacggacacggtggccggtatttacgatgcgcagacccggaagcagaacggcagcctgagcgaggaggacgcggccctcgtcacggcgcagcaccaggccgccttcgcgacgccgtacctgctgctgcacacgcgcctggtcccgctcttcgggcccgcggtcgagggcccgcccgagatgacggtcgtctttg
PRV-delete UL10  tcgacggcgcctacggcaccggcaagagcaccacggcccgggtgatggcgctcggcggggcgctgtacgtgcccgagccgatggcgtactggcgcactctgttcgacacggacacggtggccggtatttacgatgcgcagacccggaagcagaacggcagcctgagcgaggaggacgcggccctcgtcacggcgcagcaccaggccgccttcgcgacgccgtacctgctgctgcacacgcgcctggtcccgctcttcgggcccgcggtcgagggcccgcccgagatgacggtcgtctttg
                 ############################################################################################################################################################################################################################################################################################################


                      59410     59420     59430     59440     59450     59460     59470     59480     59490     59500     59510     59520     59530     59540     59550     59560     59570     59580     59590     59600     59610     59620     59630     59640     59650     59660     59670     59680     59690     59700
                 =========+=========+=========+=========+=========+=========+=========+=========+=========+=========+=========+=========+=========+=========+=========+=========+=========+=========+=========+=========+=========+=========+=========+=========+=========+=========+=========+=========+=========+=========+
PRV-WT complete  accgccacccggtggccgcgacggtgtgcttcccgctggcgcgcttcatcgtcggggacatcagcgcggcggccttcgtgggcctggcggccacgctgcccggggagccccccggcggcaacctggtggtggcctcgctggacccggacgagcacctgcggcgcctgcgcgcccgcgcgcgcgccggggagcacgtggacgcgcgcctgctcacggccctgcgcaacgtctacgccatgctggtcaacacgtcgcgctacctgagctcggggcgccgctggcgcgacgactgggggcgcg
PRV-delete UL10  accgccacccggtggccgcgacggtgtgcttcccgctggcgcgcttcatcgtcggggacatcagcgcggcggccttcgtgggcctggcggccacgctgcccggggagccccccggcggcaacctggtggtggcctcgctggacccggacgagcacctgcggcgcctgcgcgcccgcgcgcgcgccggggagcacgtggacgcgcgcctgctcacggccctgcgcaacgtctacgccatgctggtcaacacgtcgcgctacctgagctcggggcgccgctggcgcgacgactgggggcgcg
                 ############################################################################################################################################################################################################################################################################################################


                      59710     59720     59730     59740     59750     59760     59770     59780     59790     59800     59810     59820     59830     59840     59850     59860     59870     59880     59890     59900     59910     59920     59930     59940     59950     59960     59970     59980     59990     60000
                 =========+=========+=========+=========+=========+=========+=========+=========+=========+=========+=========+=========+=========+=========+=========+=========+=========+=========+=========+=========+=========+=========+=========+=========+=========+=========+=========+=========+=========+=========+
PRV-WT complete  cgccgcgcttcgaccagaccgtgcgcgactgcctcgcgctcaacgagctctgccgcccgcgcgacgaccccgagctccaggacaccctcttcggcgcgtacaaggcgcccgagctctgcgaccggcgcgggcgcccgctcgaggtgcacgcgtgggcgatggacgcgctcgtggccaagctgctgccgctgcgcgtctccaccgtcgacctggggccctcgccgcgcgtctgcgccgcggccgtggcggcgcaggcgcgcggcatggaggtgacggagtccgcgtacggcgaccacatcc
PRV-delete UL10  cgccgcgcttcgaccagaccgtgcgcgactgcctcgcgctcaacgagctctgccgcccgcgcgacgaccccgagctccaggacaccctcttcggcgcgtacaaggcgcccgagctctgcgaccggcgcgggcgcccgctcgaggtgcacgcgtgggcgatggacgcgctcgtggccaagctgctgccgctgcgcgtctccaccgtcgacctggggccctcgccgcgcgtctgcgccgcggccgtggcggcgcaggcgcgcggcatggaggtgacggagtccgcgtacggcgaccacatcc
                 ############################################################################################################################################################################################################################################################################################################


                      60010     60020     60030     60040     60050     60060     60070     60080     60090     60100     60110     60120     60130     60140     60150     60160     60170     60180     60190     60200     60210     60220     60230     60240     60250     60260     60270     60280     60290     60300
                 =========+=========+=========+=========+=========+=========+=========+=========+=========+=========+=========+=========+=========+=========+=========+=========+=========+=========+=========+=========+=========+=========+=========+=========+=========+=========+=========+=========+=========+=========+
PRV-WT complete  ggcagtgcgtgtgcgccttcacgtcggagatgggggtgtgaccctcgcccctcccacccgcgccgcggccggatggagaccgcgacggaggcaacgacgacggcgtgggagggggctcggggcgcgtataaagccatgtgtatgtcatcccaataaagtttgccgtgcccgtcaccatgcccgcgtcgtccgtgcgcctcccgctgcgcctcctgaccctcgcgggcctcctggccctcgcgggggccgccgccctcgcccgcggcgcgccgcagggtgggccgccctcgccgcaggggg
PRV-delete UL10  ggcagtgcgtgtgcgccttcacgtcggagatgggggtgtgaccctcgcccctcccacccgcgccgcggccggatggagaccgcgacggaggcaacgacgacggcgtgggagggggctcggggcgcgtataaagccatgtgtatgtcatcccaataaagtttgccgtgcccgtcaccatgcccgcgtcgtccgtgcgcctcccgctgcgcctcctgaccctcgcgggcctcctggccctcgcgggggccgccgccctcgcccgcggcgcgccgcagggtgggccgccctcgccgcaggggg
                 ############################################################################################################################################################################################################################################################################################################


                      60310     60320     60330     60340     60350     60360     60370     60380     60390     60400     60410     60420     60430     60440     60450     60460     60470     60480     60490     60500     60510     60520     60530     60540     60550     60560     60570     60580     60590     60600
                 =========+=========+=========+=========+=========+=========+=========+=========+=========+=========+=========+=========+=========+=========+=========+=========+=========+=========+=========+=========+=========+=========+=========+=========+=========+=========+=========+=========+=========+=========+
PRV-WT complete  gtcccgcgcccaccgcggcgcccgcgcgcgggcccaccctgttcgtcctggacggcgacggctccgcgtggttcgtcttccagctcggcgggctgggggcgctcaacgacacgcgcatccgcgggcacctgctcggccggtacctcgtctcgtaccaggtggtgcccccgcccgtctccgcgtggtactttgtgcagcgcccgcgcgagcgcccgcgcctctcggggccgccctcgggcgcggagctcgtggccttcgacgcgcccggcgtctggcgcacgtacaccacggcggcggtgt
PRV-delete UL10  gtcccgcgcccaccgcggcgcccgcgcgcgggcccaccctgttcgtcctggacggcgacggctccgcgtggttcgtcttccagctcggcgggctgggggcgctcaacgacacgcgcatccgcgggcacctgctcggccggtacctcgtctcgtaccaggtggtgcccccgcccgtctccgcgtggtactttgtgcagcgcccgcgcgagcgcccgcgcctctcggggccgccctcgggcgcggagctcgtggccttcgacgcgcccggcgtctggcgcacgtacaccacggcggcggtgt
                 ############################################################################################################################################################################################################################################################################################################


                      60610     60620     60630     60640     60650     60660     60670     60680     60690     60700     60710     60720     60730     60740     60750     60760     60770     60780     60790     60800     60810     60820     60830     60840     60850     60860     60870     60880     60890     60900
                 =========+=========+=========+=========+=========+=========+=========+=========+=========+=========+=========+=========+=========+=========+=========+=========+=========+=========+=========+=========+=========+=========+=========+=========+=========+=========+=========+=========+=========+=========+
PRV-WT complete  ggcccgcggaggtggccgtcctcgcggacgcggaggcgcgctgccccgcggccgtcttcaacgtgacgctgggcgaggccttcctcggcctgcgcgtcgcgctgcgctccttcctgccgctggaggtcatcatctccgccgagcggatgcgcatgatcgcgcccccggcgctcggctcggacctggagccgccgggcccgcccgcgggccgcttccacgtgtacacgctcggcttcctctccgacggggccatgcaccagacgatgcgcgacgtggccgcctacgtgcacgagagcgacg
PRV-delete UL10  ggcccgcggaggtggccgtcctcgcggacgcggaggcgcgctgccccgcggccgtcttcaacgtgacgctgggcgaggccttcctcggcctgcgcgtcgcgctgcgctccttcctgccgctggaggtcatcatctccgccgagcggatgcgcatgatcgcgcccccggcgctcggctcggacctggagccgccgggcccgcccgcgggccgcttccacgtgtacacgctcggcttcctctccgacggggccatgcaccagacgatgcgcgacgtggccgcctacgtgcacgagagcgacg
                 ############################################################################################################################################################################################################################################################################################################


                      60910     60920     60930     60940     60950     60960     60970     60980     60990     61000     61010     61020     61030     61040     61050     61060     61070     61080     61090     61100     61110     61120     61130     61140     61150     61160     61170     61180     61190     61200
                 =========+=========+=========+=========+=========+=========+=========+=========+=========+=========+=========+=========+=========+=========+=========+=========+=========+=========+=========+=========+=========+=========+=========+=========+=========+=========+=========+=========+=========+=========+
PRV-WT complete  actacctcgcccagctgtcggcggcgcacgcggccgccctggccgccgtggtgcagcccgggccgtactacttttaccgcgcggcggtgcgcctcggcgtggccgccttcgtcttctccgaggcggcgcgccgcgaccggcgcgcctcggcgccggcgctcctgcgcgtcgagagcgacgcgcgcctgctctcgcgcctgctcatgcgcgcggccggctgccccgcgggcttcgccgggctcttcgacgggcgcgccgagcgcgtcccggtggcgcccgcggaccagctccgcgccgcct
PRV-delete UL10  actacctcgcccagctgtcggcggcgcacgcggccgccctggccgccgtggtgcagcccgggccgtactacttttaccgcgcggcggtgcgcctcggcgtggccgccttcgtcttctccgaggcggcgcgccgcgaccggcgcgcctcggcgccggcgctcctgcgcgtcgagagcgacgcgcgcctgctctcgcgcctgctcatgcgcgcggccggctgccccgcgggcttcgccgggctcttcgacgggcgcgccgagcgcgtcccggtggcgcccgcggaccagctccgcgccgcct
                 ############################################################################################################################################################################################################################################################################################################


                      61210     61220     61230     61240     61250     61260     61270     61280     61290     61300     61310     61320     61330     61340     61350     61360     61370     61380     61390     61400     61410     61420     61430     61440     61450     61460     61470     61480     61490     61500
                 =========+=========+=========+=========+=========+=========+=========+=========+=========+=========+=========+=========+=========+=========+=========+=========+=========+=========+=========+=========+=========+=========+=========+=========+=========+=========+=========+=========+=========+=========+
PRV-WT complete  ggaccttcggcgaggacccggcgccccggctggacctcgcgcgggcgaccgtcgccgaggcgtaccgccgctccgtgcgggggaagcccttcgaccagcaggcgctcttcttcgccgtcgccctgctgctgcgcgccggcggccccggcgacgcgcgcgagaccctgctccgcaccacggccatgtgcaccgcggagcgcgccgccgcggccgccgagctcacgcgggccgcgctctcgccgacggccgcgtggaacgagcccttcagcctgctcgacgtcctctcgccgtgcgccgtct
PRV-delete UL10  ggaccttcggcgaggacccggcgccccggctggacctcgcgcgggcgaccgtcgccgaggcgtaccgccgctccgtgcgggggaagcccttcgaccagcaggcgctcttcttcgccgtcgccctgctgctgcgcgccggcggccccggcgacgcgcgcgagaccctgctccgcaccacggccatgtgcaccgcggagcgcgccgccgcggccgccgagctcacgcgggccgcgctctcgccgacggccgcgtggaacgagcccttcagcctgctcgacgtcctctcgccgtgcgccgtct
                 ############################################################################################################################################################################################################################################################################################################


                      61510     61520     61530     61540     61550     61560     61570     61580     61590     61600     61610     61620     61630     61640     61650     61660     61670     61680     61690     61700     61710     61720     61730     61740     61750     61760     61770     61780     61790     61800
                 =========+=========+=========+=========+=========+=========+=========+=========+=========+=========+=========+=========+=========+=========+=========+=========+=========+=========+=========+=========+=========+=========+=========+=========+=========+=========+=========+=========+=========+=========+
PRV-WT complete  cgctgcgccgcgacctcggcggggacgccaccctggccaacctgggcgccgcggcgcggctcgcgctggcgcccgccggggccccgggcgcggcggcggcgacggacgagggggcggaggaggaggaggaccccgtcgcgcgcgccgcgcccgagatccccgccgaggcgctgctcgccctgcccctgcgcgggggcgccagcttcgtgttcacgcgccggcgcccggactgcggcccggcgtacacgctcggcggcgtggacatcgccaacccgctcgtgctcgccctcgtcagcaacg
PRV-delete UL10  cgctgcgccgcgacctcggcggggacgccaccctggccaacctgggcgccgcggcgcggctcgcgctggcgcccgccggggccccgggcgcggcggcggcgacggacgagggggcggaggaggaggaggaccccgtcgcgcgcgccgcgcccgagatccccgccgaggcgctgctcgccctgcccctgcgcgggggcgccagcttcgtgttcacgcgccggcgcccggactgcggcccggcgtacacgctcggcggcgtggacatcgccaacccgctcgtgctcgccctcgtcagcaacg
                 ############################################################################################################################################################################################################################################################################################################


                      61810     61820     61830     61840     61850     61860     61870     61880     61890     61900     61910     61920     61930     61940     61950     61960     61970     61980     61990     62000     62010     62020     62030     62040     62050     62060     62070     62080     62090     62100
                 =========+=========+=========+=========+=========+=========+=========+=========+=========+=========+=========+=========+=========+=========+=========+=========+=========+=========+=========+=========+=========+=========+=========+=========+=========+=========+=========+=========+=========+=========+
PRV-WT complete  acagcgccgcgtgcgactacacggaccgcatgcccgagtcccagcacctgccggcgacggacaacccgtccgtgtgcgtgtactgcgactgcgtgttcgtgcgctactcctccgcgggcacgatcctggagaccgtcctcatcgagtccaaggacatggaggagcagctcatggccggcgccaactccaccatccccagcttcaacccgacgctgcacggcggcgacgtcaaggccctgatgctcttccccaacggcaccgtggtcgacctgctgtcgttcacgtcgacgcggctcgcgc
PRV-delete UL10  acagcgccgcgtgcgactacacggaccgcatgcccgagtcccagcacctgccggcgacggacaacccgtccgtgtgcgtgtactgcgactgcgtgttcgtgcgctactcctccgcgggcacgatcctggagaccgtcctcatcgagtccaaggacatggaggagcagctcatggccggcgccaactccaccatccccagcttcaacccgacgctgcacggcggcgacgtcaaggccctgatgctcttccccaacggcaccgtggtcgacctgctgtcgttcacgtcgacgcggctcgcgc
                 ############################################################################################################################################################################################################################################################################################################


                      62110     62120     62130     62140     62150     62160     62170     62180     62190     62200     62210     62220     62230     62240     62250     62260     62270     62280     62290     62300     62310     62320     62330     62340     62350     62360     62370     62380     62390     62400
                 =========+=========+=========+=========+=========+=========+=========+=========+=========+=========+=========+=========+=========+=========+=========+=========+=========+=========+=========+=========+=========+=========+=========+=========+=========+=========+=========+=========+=========+=========+
PRV-WT complete  ccgtgtccccggcctacgtggtggcctccgtcgtgggggcggccatcaccgtggggatcctgtacgccctgttcaagatgctctgcagcttctcctccgagggctattctcggttaataaacgccaggtcgtgaggcccgcgccgggccgcgaacccagactctctgcgtgcgcgtgttttttccttgtcgggcgcaggggagccgggaggggggagagagacggcacgggcgccgtccgtcggggagacggcgggatgacattacgagagtcgggtggaggggatgcggggagcgccgt
PRV-delete UL10  ccgtgtccccggcctacgtggtggcctccgtcgtgggggcggccatcaccgtggggatcctgtacgccctgttcaagatgctctgcagcttctcctccgagggctattctcggttaataaacgccaggtcgtgaggcccgcgccgggccgcgaacccagactctctgcgtgcgcgtgttttttccttgtcgggcgcaggggagccgggaggggggagagagacggcacgggcgccgtccgtcggggagacggcgggatgacattacgagagtcgggtggaggggatgcggggagcgccgt
                 ############################################################################################################################################################################################################################################################################################################


                      62410     62420     62430     62440     62450     62460     62470     62480     62490     62500     62510     62520     62530     62540     62550     62560     62570     62580     62590     62600     62610     62620     62630     62640     62650     62660     62670     62680     62690     62700
                 =========+=========+=========+=========+=========+=========+=========+=========+=========+=========+=========+=========+=========+=========+=========+=========+=========+=========+=========+=========+=========+=========+=========+=========+=========+=========+=========+=========+=========+=========+
PRV-WT complete  ccacgggggaaggctgctggatgacataattagagccaggggtgaggatcccctttcccccgttccccgagtatacaccactctctcccccgcacacatttcccaccgggcgccgaacgtggattgcaccccggtccgcgctacccggttgcgtgtgagacgggcggagagccgccctcgccccggtcccccaaaaactcctgcggatgcgtggtccgacgaagcctccgcggatatgtccgccgtaccctcatccccctctctcccacagccctctctcccacagccctctctcccaca
PRV-delete UL10  ccacgggggaaggctgctggatgacataattagagccaggggtgaggatcccctttcccccgttccccgagtatacaccactctctcccccgcacacatttcccaccgggcgccgaacgtggattgcaccccggtccgcgctacccggttgcgtgtgagacgggcggagagccgccctcgccccggtcccccaaaaactcctgcggatgcgtggtccgacgaagcctccgcggatatgtccgccgtaccctcatccccctctctcccacagccctctctcccacagccctctctcccaca
                 ############################################################################################################################################################################################################################################################################################################


                      62710     62720     62730     62740     62750     62760     62770     62780     62790     62800     62810     62820     62830     62840     62850     62860     62870     62880     62890     62900     62910     62920     62930     62940     62950     62960     62970     62980     62990     63000
                 =========+=========+=========+=========+=========+=========+=========+=========+=========+=========+=========+=========+=========+=========+=========+=========+=========+=========+=========+=========+=========+=========+=========+=========+=========+=========+=========+=========+=========+=========+
PRV-WT complete  gccctctctcccacagccctctctcccacagccctctctcccacagccctctctcccacagccctctctcccacagccctctctcccacagccctctctcccacagccctctctcccacagccctctctcccacagccctctctcccacagccctctctcccacagccctctctcccacagccagactcccgcctcccccgtccccgatcgccgaacgcgttcaagaattttttacccgctccgaggaaggggtctctaaggggtctctaaggggtctctaaggggtctctaaggggtct
PRV-delete UL10  gccctctctcccacagccctctctcccacagccctctctcccacagccctctctcccacagccctctctcccacagccctctctcccacagccctctctcccacagccctctctcccacagccctctctcccacagccctctctcccacagccctctctcccacagccctctctcccacagccagactcccgcctcccccgtccccgatcgccgaacgcgttcaagaattttttacccgctccgaggaaggggtctctaaggggtctctaaggggtctctaaggggtctctaaggggtct
                 ############################################################################################################################################################################################################################################################################################################


                      63010     63020     63030     63040     63050     63060     63070     63080     63090     63100     63110     63120     63130     63140     63150     63160     63170     63180     63190     63200     63210     63220     63230     63240     63250     63260     63270     63280     63290     63300
                 =========+=========+=========+=========+=========+=========+=========+=========+=========+=========+=========+=========+=========+=========+=========+=========+=========+=========+=========+=========+=========+=========+=========+=========+=========+=========+=========+=========+=========+=========+
PRV-WT complete  ctaaggggtctctaaggggtctctaaggggtctctaaggggtctctaaggggtctctaaggggtctctaaggggtctctaagggggaaccagagagtaaagaccggagagtaaagaccagagacacattttacaatttcgttgtacccgggtttattgaaaactttgttcaagagttaaagtgtctgccgcttgccggtgcgccgcgccgaggccgcgatccgtccgccgtggaccgggggatccgtggggcccgcgggcctggattcgacgggatggcgggtccgagcgacggcggtcc
PRV-delete UL10  ctaaggggtctctaaggggtctctaaggggtctctaaggggtctctaaggggtctctaaggggtctctaaggggtctctaagggggaaccagagagtaaagaccggagagtaaagaccagagacacattttacaatttcgttgtacccgggtttattgaaaactttgttcaagagttaaagtgtctgccgcttgccggtgcgccgcgccgaggccgcgatccgtccgccgtggaccgggggatccgtggggcccgcgggcctggattcgacgggatggcgggtccgagcgacggcggtcc
                 ############################################################################################################################################################################################################################################################################################################


                      63310     63320     63330     63340     63350     63360     63370     63380     63390     63400     63410     63420     63430     63440     63450     63460     63470     63480     63490     63500     63510     63520     63530     63540     63550     63560     63570     63580     63590     63600
                 =========+=========+=========+=========+=========+=========+=========+=========+=========+=========+=========+=========+=========+=========+=========+=========+=========+=========+=========+=========+=========+=========+=========+=========+=========+=========+=========+=========+=========+=========+
PRV-WT complete  ggcggcgtttcggctcgcggtcctcgcggcggcgacgatcctggtccgggggatacagcggcgacggcggcgctccgggttgtggcgccgggctccggctccccggtcttttatacccacacctcatgaatattcatgaatggttggtatgtgcgaatttttcggcgttcgcacttgttgtatataaaatattatatcctatatatattagcaattggtgcgaacgtgacgtggcccaatcattttcctcggatttgcccatgtgaccgagtttccaatagctccgcccggatcgatcac
PRV-delete UL10  ggcggcgtttcggctcgcggtcctcgcggcggcgacgatcctggtccgggggatacagcggcgacggcggcgctccgggttgtggcgccgggctccggctccccggtcttttatacccacacctcatgaatattcatgaatggttggtatgtgcgaatttttcggcgttcgcacttgttgtatataaaatattatatcctatatatattagcaattggtgcgaacgtgacgtggcccaatcattttcctcggatttgcccatgtgaccgagtttccaatagctccgcccggatcgatcac
                 ############################################################################################################################################################################################################################################################################################################


                      63610     63620     63630     63640     63650     63660     63670     63680     63690     63700     63710     63720     63730     63740     63750     63760     63770     63780     63790     63800     63810     63820     63830     63840     63850     63860     63870     63880     63890     63900
                 =========+=========+=========+=========+=========+=========+=========+=========+=========+=========+=========+=========+=========+=========+=========+=========+=========+=========+=========+=========+=========+=========+=========+=========+=========+=========+=========+=========+=========+=========+
PRV-WT complete  cgggccgatattttgccgataatccatcaatattttcttcggccgacgcgggttccacccgggccgtcccgccggggagcccggcccgggcgcgggactctgctctccgggcgccgcgcggcgtcgagaccgcgccgggacgatggcgtctgcgcatgcgcgggtggaggcgagtggccgggaatcccctctcgtgccgagccccgcccatcgtcgcgtgtcctctccctcacccccttctcccccgaccgggcgacgatgggccgcgtcctcgccacgatggaggagaggcgctcacgc
PRV-delete UL10  cgggccgatattttgccgataatccatcaatattttcttcggccgacgcgggttccacccgggccgtcccgccggggagcccggcccgggcgcgggactctgctctccgggcgccgcgcggcgtcgagaccgcgccgggacgatggcgtctgcgcatgcgcgggtggaggcgagtggccgggaatcccctctcgtgccgagccccgcccatcgtcgcgtgtcctctccctcacccccttctcccccgaccgggcgacgatgggccgcgtcctcgccacgatggaggagaggcgctcacgc
                 ############################################################################################################################################################################################################################################################################################################


                      63910     63920     63930     63940     63950     63960     63970     63980     63990     64000     64010     64020     64030     64040     64050     64060     64070     64080     64090     64100     64110     64120     64130     64140     64150     64160     64170     64180     64190     64200
                 =========+=========+=========+=========+=========+=========+=========+=========+=========+=========+=========+=========+=========+=========+=========+=========+=========+=========+=========+=========+=========+=========+=========+=========+=========+=========+=========+=========+=========+=========+
PRV-WT complete  acgcgcgggtacgcgggagttgcggggatcggggaccgggacgatcggggtgtgcggaccgtggcgcgtcggggggctgggtgcgagcgggggttgggcgcgcgcgtgtgtggggggggaacgggaaacgggggacgggggaacccgccgaggcgcgcgtgcgcgcgcacgcgtgtgtgtgccgcggacagagacggacgggagaacggacgtgtgatttgtttaacggtttttattgaggacgatggagatgttggcgcgggccaggcgcgcccgaaacagctgaaacaggtactgggc
PRV-delete UL10  acgcgcgggtacgcgggagttgcggggatcggggaccgggacgatcggggtgtgcggaccgtggcgcgtcggggggctgggtgcgagcgggggttgggcgcgcgcgtgtgtggggggggaacgggaaacgggggacgggggaacccgccgaggcgcgcgtgcgcgcgcacgcgtgtgtgtgccgcggacagagacggacgggagaacggacgtgtgatttgtttaacggtttttattgaggacgatggagatgttggcgcgggccaggcgcgcccgaaacagctgaaacaggtactgggc
                 ############################################################################################################################################################################################################################################################################################################


                      64210     64220     64230     64240     64250     64260     64270     64280     64290     64300     64310     64320     64330     64340     64350     64360     64370     64380     64390     64400     64410     64420     64430     64440     64450     64460     64470     64480     64490     64500
                 =========+=========+=========+=========+=========+=========+=========+=========+=========+=========+=========+=========+=========+=========+=========+=========+=========+=========+=========+=========+=========+=========+=========+=========+=========+=========+=========+=========+=========+=========+
PRV-WT complete  cccacggagggccgcgggcgagtcgccgaaggcggtcaggagcgccgcctcggcgcagacggcggacgtgagccgcagcgcgcggcccacgggcgcggtcgcggcgacgaggccccggccggcgtagagcccgtccagcacccgcgccccggccgcctccaccagctcgcacacgccgggcgccggcgcgttcgcctccgcgtcgctcatcatgaccgccagctccaggcccgtcgccgcgtcctcccgggcgtgcttcgtcgtcggcggtgaggcccccgccggggccgcggagacgat
PRV-delete UL10  cccacggagggccgcgggcgagtcgccgaaggcggtcaggagcgccgcctcggcgcagacggcggacgtgagccgcagcgcgcggcccacgggcgcggtcgcggcgacgaggccccggccggcgtagagcccgtccagcacccgcgccccggccgcctccaccagctcgcacacgccgggcgccggcgcgttcgcctccgcgtcgctcatcatgaccgccagctccaggcccgtcgccgcgtcctcccgggcgtgcttcgtcgtcggcggtgaggcccccgccggggccgcggagacgat
                 ############################################################################################################################################################################################################################################################################################################


                      64510     64520     64530     64540     64550     64560     64570     64580     64590     64600     64610     64620     64630     64640     64650     64660     64670     64680     64690     64700     64710     64720     64730     64740     64750     64760     64770     64780     64790     64800
                 =========+=========+=========+=========+=========+=========+=========+=========+=========+=========+=========+=========+=========+=========+=========+=========+=========+=========+=========+=========+=========+=========+=========+=========+=========+=========+=========+=========+=========+=========+
PRV-WT complete  gggcgcgagcgcgggggcgtgcaccacggcggcgttcacgagcatccgcatcacctcgccgaagacggtcgcctcgcgcagcacgcggttgacggcgtgggccacggcgctcgcggcccgagaggcgccgccgtcgccgttgccgttgcctccgccgcctcccccctcgccgccgtcctcgtcgtcgcccgcgcccgccccggggcgctcgtccagcgtgtcgagcatgccctccagcgcggcgtagcagctcggcaggtgccagcgggccaggatgaactggtacagcgcgaaccgctg
PRV-delete UL10  gggcgcgagcgcgggggcgtgcaccacggcggcgttcacgagcatccgcatcacctcgccgaagacggtcgcctcgcgcagcacgcggttgacggcgtgggccacggcgctcgcggcccgagaggcgccgccgtcgccgttgccgttgcctccgccgcctcccccctcgccgccgtcctcgtcgtcgcccgcgcccgccccggggcgctcgtccagcgtgtcgagcatgccctccagcgcggcgtagcagctcggcaggtgccagcgggccaggatgaactggtacagcgcgaaccgctg
                 ############################################################################################################################################################################################################################################################################################################


                      64810     64820     64830     64840     64850     64860     64870     64880     64890     64900     64910     64920     64930     64940     64950     64960     64970     64980     64990     65000     65010     65020     65030     65040     65050     65060     65070     65080     65090     65100
                 =========+=========+=========+=========+=========+=========+=========+=========+=========+=========+=========+=========+=========+=========+=========+=========+=========+=========+=========+=========+=========+=========+=========+=========+=========+=========+=========+=========+=========+=========+
PRV-WT complete  cgcgcccgtgagcgacgtggagcccgtcccgaagaaggggtccgccgcgacggcggacacggcctcgaccacggcggccgaggcgtcgtccgaggcgcacgcggcccgccgcaggatggcgtccgccccggccacgaggtcgcagagcatctgcagggacaggctcccgccgcgcgtcgcccagatgcgccgcgtcgccgggatgtagcgcacctgcacaaagtcgctgaccaccgtcgcgcgcagcgcgcgctcggcgtaccggcgcgggcgcacgctcagcgtgggggccgcgcgccc
PRV-delete UL10  cgcgcccgtgagcgacgtggagcccgtcccgaagaaggggtccgccgcgacggcggacacggcctcgaccacggcggccgaggcgtcgtccgaggcgcacgcggcccgccgcaggatggcgtccgccccggccacgaggtcgcagagcatctgcagggacaggctcccgccgcgcgtcgcccagatgcgccgcgtcgccgggatgtagcgcacctgcacaaagtcgctgaccaccgtcgcgcgcagcgcgcgctcggcgtaccggcgcgggcgcacgctcagcgtgggggccgcgcgccc
                 ############################################################################################################################################################################################################################################################################################################


                      65110     65120     65130     65140     65150     65160     65170     65180     65190     65200     65210     65220     65230     65240     65250     65260     65270     65280     65290     65300     65310     65320     65330     65340     65350     65360     65370     65380     65390     65400
                 =========+=========+=========+=========+=========+=========+=========+=========+=========+=========+=========+=========+=========+=========+=========+=========+=========+=========+=========+=========+=========+=========+=========+=========+=========+=========+=========+=========+=========+=========+
PRV-WT complete  gcccttcaccaccacggccgtgggccgcgtctcgccgctgagcggggggcgcacgccggggatgccgtcaaagagcccctccgtcagcacctgcagcgagctgggcagctcggagaggtacgcctgcaccagggcgaagcaggcctcgttcacggcgtacacgaagcccgaggaggggtgcacgatggcgggctcggccaggagctccaggacacggtcctgctggcggtcgtgcacgagcgtcagcgagagcgccgacgccggcagcgagaccatgcactcgacgaggtaggggtcgta
PRV-delete UL10  gcccttcaccaccacggccgtgggccgcgtctcgccgctgagcggggggcgcacgccggggatgccgtcaaagagcccctccgtcagcacctgcagcgagctgggcagctcggagaggtacgcctgcaccagggcgaagcaggcctcgttcacggcgtacacgaagcccgaggaggggtgcacgatggcgggctcggccaggagctccaggacacggtcctgctggcggtcgtgcacgagcgtcagcgagagcgccgacgccggcagcgagaccatgcactcgacgaggtaggggtcgta
                 ############################################################################################################################################################################################################################################################################################################


                      65410     65420     65430     65440     65450     65460     65470     65480     65490     65500     65510     65520     65530     65540     65550     65560     65570     65580     65590     65600     65610     65620     65630     65640     65650     65660     65670     65680     65690     65700
                 =========+=========+=========+=========+=========+=========+=========+=========+=========+=========+=========+=========+=========+=========+=========+=========+=========+=========+=========+=========+=========+=========+=========+=========+=========+=========+=========+=========+=========+=========+
PRV-WT complete  cacgtccagctccgcgcggccgcagaggtccagctccgtcagcggcagcagcagccccagggcgtccacgaagacgtcgtcgctcccctgcggggcggcccacgcgcggccgaggcgcgccagctccgtgcgcacgtagttggcgacgacgcggttccccggcgcgttcccgcgcagcgtgagcccgaacttgccgatctcccgcgagcgccggtgcacggacacgatgcagcccccgtgcacaaagtacgcgcggtccccgccgtcggcgatgtagaacagcaccccctggtgcacgat
PRV-delete UL10  cacgtccagctccgcgcggccgcagaggtccagctccgtcagcggcagcagcagccccagggcgtccacgaagacgtcgtcgctcccctgcggggcggcccacgcgcggccgaggcgcgccagctccgtgcgcacgtagttggcgacgacgcggttccccggcgcgttcccgcgcagcgtgagcccgaacttgccgatctcccgcgagcgccggtgcacggacacgatgcagcccccgtgcacaaagtacgcgcggtccccgccgtcggcgatgtagaacagcaccccctggtgcacgat
                 ############################################################################################################################################################################################################################################################################################################


                      65710     65720     65730     65740     65750     65760     65770     65780     65790     65800     65810     65820     65830     65840     65850     65860     65870     65880     65890     65900     65910     65920     65930     65940     65950     65960     65970     65980     65990     66000
                 =========+=========+=========+=========+=========+=========+=========+=========+=========+=========+=========+=========+=========+=========+=========+=========+=========+=========+=========+=========+=========+=========+=========+=========+=========+=========+=========+=========+=========+=========+
PRV-WT complete  cgtgctctggtactcaaactccatggcggcggacacacgcggcgcggcaaatgggtgagcgaggcggcgcgaccactcctttaaacccgccggggggcgcctcccgccgcacacacgcggcggcgcggggatggaggacgcggcggcggtggacgtggacgcggcgtcggacgcgacgacgacggcggcggtgggggagaaggacgcgctgctgagctcggcctttgtgggcgcgcgcccgccgcgcccgcgcttcagctcgcacgtggtgagcctcctggtcctggcgctcgcgctccg
PRV-delete UL10  cgtgctctggtactcaaactccatggcggcggacacacgcggcgcggcaaatgggtgagcgaggcggcgcgaccactcctttaaacccgccggggggcgcctcccgccgcacacacgcggcggcgcggggatggaggacgcggcggcggtggacgtggacgcggcgtcggacgcgacgacgacggcggcggtgggggagaaggacgcgctgctgagctcggcctttgtgggcgcgcgcccgccgcgcccgcgcttcagctcgcacgtggtgagcctcctggtcctggcgctcgcgctccg
                 ############################################################################################################################################################################################################################################################################################################


                      66010     66020     66030     66040     66050     66060     66070     66080     66090     66100     66110     66120     66130     66140     66150     66160     66170     66180     66190     66200     66210     66220     66230     66240     66250     66260     66270     66280     66290     66300
                 =========+=========+=========+=========+=========+=========+=========+=========+=========+=========+=========+=========+=========+=========+=========+=========+=========+=========+=========+=========+=========+=========+=========+=========+=========+=========+=========+=========+=========+=========+
PRV-WT complete  gcccgcgtgctgcctcgtcctggcgctccacggatcgcgggccaccctcgcggcgctcctcaccgcgctggccttctacgcgcgcgcggccgtctgcgccgccctcgtggcgcggaacgtggcccgggaccggatgccgctctcgccggcgcagcaggtggcgctggggctgctggccgcggcgcggctggccttcttgtacgtcgccctggacgcgggccgccattacgcgcccgccctcgccggggcgctgtacggcgccgactgcgtctgtgacgcgctcgccttcctgctgccgcg
PRV-delete UL10  gcccgcgtgctgcctcgtcctggcgctccacggatcgcgggccaccctcgcggcgctcctcaccgcgctggccttctacgcgcgcgcggccgtctgcgccgccctcgtggcgcggaacgtggcccgggaccggatgccgctctcgccggcgcagcaggtggcgctggggctgctggccgcggcgcggctggccttcttgtacgtcgccctggacgcgggccgccattacgcgcccgccctcgccggggcgctgtacggcgccgactgcgtctgtgacgcgctcgccttcctgctgccgcg
                 ############################################################################################################################################################################################################################################################################################################


                      66310     66320     66330     66340     66350     66360     66370     66380     66390     66400     66410     66420     66430     66440     66450     66460     66470     66480     66490     66500     66510     66520     66530     66540     66550     66560     66570     66580     66590     66600
                 =========+=========+=========+=========+=========+=========+=========+=========+=========+=========+=========+=========+=========+=========+=========+=========+=========+=========+=========+=========+=========+=========+=========+=========+=========+=========+=========+=========+=========+=========+
PRV-WT complete  cgcgtacgcgcgctccatcatgcattaaaaggcggccgcgacgcgcccgcgccattcttcccccgccgcgccccagcgaccgcgcacacgcgcccgtccgcggtcgtcgctacacgatggagcgcccggccatcctgccgtccgggcagatcctgtccaacatcgaggtgcactcgcaccgcgcgctcttcgacctcttcaagcgcttccgctcggacgacaacaacctctacggcgccgagttcgacgcgctgctcggcacctactgcagcacgctctcgctcgtgcgcttcctggagc
PRV-delete UL10  cgcgtacgcgcgctccatcatgcattaaaaggcggccgcgacgcgcccgcgccattcttcccccgccgcgccccagcgaccgcgcacacgcgcccgtccgcggtcgtcgctacacgatggagcgcccggccatcctgccgtccgggcagatcctgtccaacatcgaggtgcactcgcaccgcgcgctcttcgacctcttcaagcgcttccgctcggacgacaacaacctctacggcgccgagttcgacgcgctgctcggcacctactgcagcacgctctcgctcgtgcgcttcctggagc
                 ############################################################################################################################################################################################################################################################################################################


                      66610     66620     66630     66640     66650     66660     66670     66680     66690     66700     66710     66720     66730     66740     66750     66760     66770     66780     66790     66800     66810     66820     66830     66840     66850     66860     66870     66880     66890     66900
                 =========+=========+=========+=========+=========+=========+=========+=========+=========+=========+=========+=========+=========+=========+=========+=========+=========+=========+=========+=========+=========+=========+=========+=========+=========+=========+=========+=========+=========+=========+
PRV-WT complete  tcgggctctcggtggcgtgcgtgtgcaccaagttcccggagctcagctacgtggccgagggcaccatccagtttgaggtgcagcagccgatgatcgcgcgcgacgggccgcacccggccgaccagcccgtgcacaactacatgatcaagcgcctcgaccgccgctccctcaacgccgccttctcgatcgccgtcgaggccctcgggctcatctcgggcgagaacctcgacggcacgcacatctcgtcggccatgcgcctgcgcgccatccagcagctcgcgcgcaacgtgcaggcggtgc
PRV-delete UL10  tcgggctctcggtggcgtgcgtgtgcaccaagttcccggagctcagctacgtggccgagggcaccatccagtttgaggtgcagcagccgatgatcgcgcgcgacgggccgcacccggccgaccagcccgtgcacaactacatgatcaagcgcctcgaccgccgctccctcaacgccgccttctcgatcgccgtcgaggccctcgggctcatctcgggcgagaacctcgacggcacgcacatctcgtcggccatgcgcctgcgcgccatccagcagctcgcgcgcaacgtgcaggcggtgc
                 ############################################################################################################################################################################################################################################################################################################


                      66910     66920     66930     66940     66950     66960     66970     66980     66990     67000     67010     67020     67030     67040     67050     67060     67070     67080     67090     67100     67110     67120     67130     67140     67150     67160     67170     67180     67190     67200
                 =========+=========+=========+=========+=========+=========+=========+=========+=========+=========+=========+=========+=========+=========+=========+=========+=========+=========+=========+=========+=========+=========+=========+=========+=========+=========+=========+=========+=========+=========+
PRV-WT complete  tggactcgttcgagcgcggcacggccgaccagatgctgcgcgtcctcatggagaaggcgccgcccctgtcgctgctggcgcccttcacgctctacgagggccggctcgcggaccgcgtggcctgcgccgcgctcgtgtcggagctgaagcgccgcgtgcgcgacgacaccttcttcctcacgaagcacgagcgcaacaaggacgcggtcctggaccggctctcggacctggtgaactgcaccgcgccctcggtggccgtcgcgcgcatgacccacgcggacacgcaggggcgccccgtcg
PRV-delete UL10  tggactcgttcgagcgcggcacggccgaccagatgctgcgcgtcctcatggagaaggcgccgcccctgtcgctgctggcgcccttcacgctctacgagggccggctcgcggaccgcgtggcctgcgccgcgctcgtgtcggagctgaagcgccgcgtgcgcgacgacaccttcttcctcacgaagcacgagcgcaacaaggacgcggtcctggaccggctctcggacctggtgaactgcaccgcgccctcggtggccgtcgcgcgcatgacccacgcggacacgcaggggcgccccgtcg
                 ############################################################################################################################################################################################################################################################################################################


                      67210     67220     67230     67240     67250     67260     67270     67280     67290     67300     67310     67320     67330     67340     67350     67360     67370     67380     67390     67400     67410     67420     67430     67440     67450     67460     67470     67480     67490     67500
                 =========+=========+=========+=========+=========+=========+=========+=========+=========+=========+=========+=========+=========+=========+=========+=========+=========+=========+=========+=========+=========+=========+=========+=========+=========+=========+=========+=========+=========+=========+
PRV-WT complete  acggggtgctggtgaccacggccggcgtgcgccagcggctcctgcaccacgtgctgaccctggcggacacgcacgcggacgtgcccgtgacctacggcgagatggtgatcgccaacaccaacctcgtgacggcgctggtgatgggcaaggccgtcagcaacatggacgacgtggcgcgctacctgctcggcggcgagccggcccccgacgacggcaagcccgtgggctcggcgcgcgtgcgcgcggacctcgtcgtcgtcggcgaccgcctggtgttcctcgaggccctcgagaagcgcg
PRV-delete UL10  acggggtgctggtgaccacggccggcgtgcgccagcggctcctgcaccacgtgctgaccctggcggacacgcacgcggacgtgcccgtgacctacggcgagatggtgatcgccaacaccaacctcgtgacggcgctggtgatgggcaaggccgtcagcaacatggacgacgtggcgcgctacctgctcggcggcgagccggcccccgacgacggcaagcccgtgggctcggcgcgcgtgcgcgcggacctcgtcgtcgtcggcgaccgcctggtgttcctcgaggccctcgagaagcgcg
                 ############################################################################################################################################################################################################################################################################################################


                      67510     67520     67530     67540     67550     67560     67570     67580     67590     67600     67610     67620     67630     67640     67650     67660     67670     67680     67690     67700     67710     67720     67730     67740     67750     67760     67770     67780     67790     67800
                 =========+=========+=========+=========+=========+=========+=========+=========+=========+=========+=========+=========+=========+=========+=========+=========+=========+=========+=========+=========+=========+=========+=========+=========+=========+=========+=========+=========+=========+=========+
PRV-WT complete  tgtaccaggccacgcaggtgccgtacccgctggtgggcaacctggacgtgaccttcgtcatgcccctgggggtcttcaagccggccgccgaccgctacgcgcgccacgccggcagcttcgcgcccacgccgggcctcccggacccgcgcacgcacccgccccgcgccgtgcacttcttcaacaaggacggcgtgccctgccacgtgaccttcgagcacgccatgggcaccctctgccacccctccttcctggacgtggacgccacgctggcggcgctgcgccaggagcccgcggaggtgc
PRV-delete UL10  tgtaccaggccacgcaggtgccgtacccgctggtgggcaacctggacgtgaccttcgtcatgcccctgggggtcttcaagccggccgccgaccgctacgcgcgccacgccggcagcttcgcgcccacgccgggcctcccggacccgcgcacgcacccgccccgcgccgtgcacttcttcaacaaggacggcgtgccctgccacgtgaccttcgagcacgccatgggcaccctctgccacccctccttcctggacgtggacgccacgctggcggcgctgcgccaggagcccgcggaggtgc
                 ############################################################################################################################################################################################################################################################################################################


                      67810     67820     67830     67840     67850     67860     67870     67880     67890     67900     67910     67920     67930     67940     67950     67960     67970     67980     67990     68000     68010     68020     68030     68040     68050     68060     68070     68080     68090     68100
                 =========+=========+=========+=========+=========+=========+=========+=========+=========+=========+=========+=========+=========+=========+=========+=========+=========+=========+=========+=========+=========+=========+=========+=========+=========+=========+=========+=========+=========+=========+
PRV-WT complete  agtgcgccttcggcgcctacgtggccgacgcgcgccccgacgccctcgtcgggctcatgcagcgcttcctcgaggagtggcccggcatgatgcccgtgcgcccgcgctgggccgcgccgccggccgccgaccagctgctggcgcccggcaacgccgacctgcgcctggagctgcacccggcctttgacttcttcgtcgcgcccgaggtcgacgtccccgggcccttcgccgtgccccaggtgatggggcaggtgcgcgcgatgccgcgcatcatcaacggcaacatcccgctggccctgt
PRV-delete UL10  agtgcgccttcggcgcctacgtggccgacgcgcgccccgacgccctcgtcgggctcatgcagcgcttcctcgaggagtggcccggcatgatgcccgtgcgcccgcgctgggccgcgccgccggccgccgaccagctgctggcgcccggcaacgccgacctgcgcctggagctgcacccggcctttgacttcttcgtcgcgcccgaggtcgacgtccccgggcccttcgccgtgccccaggtgatggggcaggtgcgcgcgatgccgcgcatcatcaacggcaacatcccgctggccctgt
                 ############################################################################################################################################################################################################################################################################################################


                      68110     68120     68130     68140     68150     68160     68170     68180     68190     68200     68210     68220     68230     68240     68250     68260     68270     68280     68290     68300     68310     68320     68330     68340     68350     68360     68370     68380     68390     68400
                 =========+=========+=========+=========+=========+=========+=========+=========+=========+=========+=========+=========+=========+=========+=========+=========+=========+=========+=========+=========+=========+=========+=========+=========+=========+=========+=========+=========+=========+=========+
PRV-WT complete  gccccgtggactttagggacgcgcgcggcttcgagctcagcgtggaccggcaccggctggccccggccacggtggccgcggtgcgcggcgccttccgcgacgccaactaccccatggtgttctacatcatcgaggccgtgatccacgggagcgagcgcaccttctgcgcgctcgcgcggctcgtggcgcagtgcatccagagctactggcgcaacacacacaacgccgccttcgtgaacaacttctacatggtcatgtacatcaacacctacctgggcaacggggagctgcccgaggact
PRV-delete UL10  gccccgtggactttagggacgcgcgcggcttcgagctcagcgtggaccggcaccggctggccccggccacggtggccgcggtgcgcggcgccttccgcgacgccaactaccccatggtgttctacatcatcgaggccgtgatccacgggagcgagcgcaccttctgcgcgctcgcgcggctcgtggcgcagtgcatccagagctactggcgcaacacacacaacgccgccttcgtgaacaacttctacatggtcatgtacatcaacacctacctgggcaacggggagctgcccgaggact
                 ############################################################################################################################################################################################################################################################################################################


                      68410     68420     68430     68440     68450     68460     68470     68480     68490     68500     68510     68520     68530     68540     68550     68560     68570     68580     68590     68600     68610     68620     68630     68640     68650     68660     68670     68680     68690     68700
                 =========+=========+=========+=========+=========+=========+=========+=========+=========+=========+=========+=========+=========+=========+=========+=========+=========+=========+=========+=========+=========+=========+=========+=========+=========+=========+=========+=========+=========+=========+
PRV-WT complete  gcgccgccgtgtacaaggacctgctggagcacgtgcacgccctgcggcgcctcatcggcgagttcacgctgcccggggacccgctgggcaaccagccccaggaggagctcaaccacgcgctggcggacgccgcgctgctgccgccgctcatctgggactgcgaccccatcctgtaccgcgacgggctcgccgagcgcctgccggagctgcgcgtcaacggcgcgcactttcagcacatcctgtgggtcgagatggcccaggtgaactttcgcaacgtcggcggcggcctggtccacaacc
PRV-delete UL10  gcgccgccgtgtacaaggacctgctggagcacgtgcacgccctgcggcgcctcatcggcgagttcacgctgcccggggacccgctgggcaaccagccccaggaggagctcaaccacgcgctggcggacgccgcgctgctgccgccgctcatctgggactgcgaccccatcctgtaccgcgacgggctcgccgagcgcctgccggagctgcgcgtcaacggcgcgcactttcagcacatcctgtgggtcgagatggcccaggtgaactttcgcaacgtcggcggcggcctggtccacaacc
                 ############################################################################################################################################################################################################################################################################################################


                      68710     68720     68730     68740     68750     68760     68770     68780     68790     68800     68810     68820     68830     68840     68850     68860     68870     68880     68890     68900     68910     68920     68930     68940     68950     68960     68970     68980     68990     69000
                 =========+=========+=========+=========+=========+=========+=========+=========+=========+=========+=========+=========+=========+=========+=========+=========+=========+=========+=========+=========+=========+=========+=========+=========+=========+=========+=========+=========+=========+=========+
PRV-WT complete  ggcccgtgcggaacgagaaccagcccctgcacccgcaccacgacgccgagtggtcggtgctgtccaagatctactactacgccgtggtgcccgccttctcgcgcggcaactgctgcaccatgggcgtgcgctacgaccgcgtgtaccagctggtgcagacgatggtggtgcccgaggcggacgaggaggtgggcgcggacgacccccggcacccgctgcacccgcgcaacctcgtgcccaactcgctgaacgtgctcttccacaacgcctgcgtcgccgtggacgcggacgccatgctga
PRV-delete UL10  ggcccgtgcggaacgagaaccagcccctgcacccgcaccacgacgccgagtggtcggtgctgtccaagatctactactacgccgtggtgcccgccttctcgcgcggcaactgctgcaccatgggcgtgcgctacgaccgcgtgtaccagctggtgcagacgatggtggtgcccgaggcggacgaggaggtgggcgcggacgacccccggcacccgctgcacccgcgcaacctcgtgcccaactcgctgaacgtgctcttccacaacgcctgcgtcgccgtggacgcggacgccatgctga
                 ############################################################################################################################################################################################################################################################################################################


                      69010     69020     69030     69040     69050     69060     69070     69080     69090     69100     69110     69120     69130     69140     69150     69160     69170     69180     69190     69200     69210     69220     69230     69240     69250     69260     69270     69280     69290     69300
                 =========+=========+=========+=========+=========+=========+=========+=========+=========+=========+=========+=========+=========+=========+=========+=========+=========+=========+=========+=========+=========+=========+=========+=========+=========+=========+=========+=========+=========+=========+
PRV-WT complete  tcctgcaggagacggtgacgaacatggccgagcgcacgacgccgctcctggcctcggcggcgcccgacgcgggcatggccacggtggccacgcgggacatgcgcacgcacgacggctcgctgcaccacggcctgctcatgatggcctaccagcccaacgacgccacgctgctcgagggggccttcttctacccggcgcccgtgaacgcgctctttgcctgcgccgaccacctcggcgccatgcgcgacgtgggcgccgaggtgcgcgccgccgcccagcacgtgccctgcgtgccccact
PRV-delete UL10  tcctgcaggagacggtgacgaacatggccgagcgcacgacgccgctcctggcctcggcggcgcccgacgcgggcatggccacggtggccacgcgggacatgcgcacgcacgacggctcgctgcaccacggcctgctcatgatggcctaccagcccaacgacgccacgctgctcgagggggccttcttctacccggcgcccgtgaacgcgctctttgcctgcgccgaccacctcggcgccatgcgcgacgtgggcgccgaggtgcgcgccgccgcccagcacgtgccctgcgtgccccact
                 ############################################################################################################################################################################################################################################################################################################


                      69310     69320     69330     69340     69350     69360     69370     69380     69390     69400     69410     69420     69430     69440     69450     69460     69470     69480     69490     69500     69510     69520     69530     69540     69550     69560     69570     69580     69590     69600
                 =========+=========+=========+=========+=========+=========+=========+=========+=========+=========+=========+=========+=========+=========+=========+=========+=========+=========+=========+=========+=========+=========+=========+=========+=========+=========+=========+=========+=========+=========+
PRV-WT complete  ttctcggggccaactactacgccacggtgcgccagcccgtggcccagcacgcggcgcagagccgcgccgacgagaacacgctctcgtacgcgctcatggcgggctacttcaagatgagccccgtggccttcacccaccagctgcggcgccagctgcaccccggcttcgccctgacggtggtgcgccaggaccgcttcgccacggagaacgtgctctttgcggagaaggccagcgagtcgtactttatgggccagatgcaggtggcgcgcaccgagagcggcggcgggctgcacatgcagc
PRV-delete UL10  ttctcggggccaactactacgccacggtgcgccagcccgtggcccagcacgcggcgcagagccgcgccgacgagaacacgctctcgtacgcgctcatggcgggctacttcaagatgagccccgtggccttcacccaccagctgcggcgccagctgcaccccggcttcgccctgacggtggtgcgccaggaccgcttcgccacggagaacgtgctctttgcggagaaggccagcgagtcgtactttatgggccagatgcaggtggcgcgcaccgagagcggcggcgggctgcacatgcagc
                 ############################################################################################################################################################################################################################################################################################################


                      69610     69620     69630     69640     69650     69660     69670     69680     69690     69700     69710     69720     69730     69740     69750     69760     69770     69780     69790     69800     69810     69820     69830     69840     69850     69860     69870     69880     69890     69900
                 =========+=========+=========+=========+=========+=========+=========+=========+=========+=========+=========+=========+=========+=========+=========+=========+=========+=========+=========+=========+=========+=========+=========+=========+=========+=========+=========+=========+=========+=========+
PRV-WT complete  tcacgcagccccgcgccaacgtggacctgggcgtgggcttcacggccgcgtacgcggcggccgcgctgcgtgcccccgtgacggacatgggcaacctgccgcagaacctcttcgccacgcgcggcgccccgcccatgctggacgcggacgcggacgactacctgcgccgcacggtcaacgccggcaaccgcctggcgcccgtgcccgtcttcggccagatgctgccccaggtgcccgtgggcctggcccgcgggcagcagtcggtgtgcgagttcatcgccacgcccgtctcggtcgacc
PRV-delete UL10  tcacgcagccccgcgccaacgtggacctgggcgtgggcttcacggccgcgtacgcggcggccgcgctgcgtgcccccgtgacggacatgggcaacctgccgcagaacctcttcgccacgcgcggcgccccgcccatgctggacgcggacgcggacgactacctgcgccgcacggtcaacgccggcaaccgcctggcgcccgtgcccgtcttcggccagatgctgccccaggtgcccgtgggcctggcccgcgggcagcagtcggtgtgcgagttcatcgccacgcccgtctcggtcgacc
                 ############################################################################################################################################################################################################################################################################################################


                      69910     69920     69930     69940     69950     69960     69970     69980     69990     70000     70010     70020     70030     70040     70050     70060     70070     70080     70090     70100     70110     70120     70130     70140     70150     70160     70170     70180     70190     70200
                 =========+=========+=========+=========+=========+=========+=========+=========+=========+=========+=========+=========+=========+=========+=========+=========+=========+=========+=========+=========+=========+=========+=========+=========+=========+=========+=========+=========+=========+=========+
PRV-WT complete  tggcctactttcggcgcgcgtgcaacccgcgcgggcgcgccgccggcgaggtgcacggcgaggaggggctgatgttcgaccacagccacgcggaccccgcgcacccccaccgcgccaccgccaacccctgggcctcgcagcggcactcgtacgcggaccggctctacaacggccagtacaacatgagcggcccggcctacagcccctgcttcaagttcttcacgcccgccgaggcggtcgccaagagccgggggctggcgcggctcatcgccgacacgggcgccgccgcctcgccgacga
PRV-delete UL10  tggcctactttcggcgcgcgtgcaacccgcgcgggcgcgccgccggcgaggtgcacggcgaggaggggctgatgttcgaccacagccacgcggaccccgcgcacccccaccgcgccaccgccaacccctgggcctcgcagcggcactcgtacgcggaccggctctacaacggccagtacaacatgagcggcccggcctacagcccctgcttcaagttcttcacgcccgccgaggcggtcgccaagagccgggggctggcgcggctcatcgccgacacgggcgccgccgcctcgccgacga
                 ############################################################################################################################################################################################################################################################################################################


                      70210     70220     70230     70240     70250     70260     70270     70280     70290     70300     70310     70320     70330     70340     70350     70360     70370     70380     70390     70400     70410     70420     70430     70440     70450     70460     70470     70480     70490     70500
                 =========+=========+=========+=========+=========+=========+=========+=========+=========+=========+=========+=========+=========+=========+=========+=========+=========+=========+=========+=========+=========+=========+=========+=========+=========+=========+=========+=========+=========+=========+
PRV-WT complete  gcaacggcgagtaccagttcaagcggcccgtgggcgccggcgagctcgtcgaggacccgtgcgcgctcttccaggaggcgtacccgccgctgtgcgccagcgactcggcgctgctgcggacgcccctcggcgccgaggagcactttgcgcagtacctcatccgcgacgagtccccgctgaagggctgcttccagcacgcgagcgcctgagccgccggaccgataccggaccgccgccgaaccgtcgtctccgccctgccccctccccccccgactgctctgtcacccgcccccaccctcc
PRV-delete UL10  gcaacggcgagtaccagttcaagcggcccgtgggcgccggcgagctcgtcgaggacccgtgcgcgctcttccaggaggcgtacccgccgctgtgcgccagcgactcggcgctgctgcggacgcccctcggcgccgaggagcactttgcgcagtacctcatccgcgacgagtccccgctgaagggctgcttccagcacgcgagcgcctgagccgccggaccgataccggaccgccgccgaaccgtcgtctccgccctgccccctccccccccgactgctctgtcacccgcccccaccctcc
                 ############################################################################################################################################################################################################################################################################################################


                      70510     70520     70530     70540     70550     70560     70570     70580     70590     70600     70610     70620     70630     70640     70650     70660     70670     70680     70690     70700     70710     70720     70730     70740     70750     70760     70770     70780     70790     70800
                 =========+=========+=========+=========+=========+=========+=========+=========+=========+=========+=========+=========+=========+=========+=========+=========+=========+=========+=========+=========+=========+=========+=========+=========+=========+=========+=========+=========+=========+=========+
PRV-WT complete  cgcgccggccgcgcgccgcgcgtgtatataaactgtgtacacggcacccgcccgcgtcagtgtcttctttcgctcgcgtcgccatggaggtggacattgcgctgccgacgctgtccccgggggatctgtcggccctgcagaggtgcgagggccgcgtggtgtttctggagacgctgcggcgtcacgcgacgctgcgcgaggtcgcgctgccctgcggcggcgatgtgctcgcggccatggccgcgtaccggcggcgcttcgcggccgtcatcacgcgcgtgacgccgcaccgcatgctgg
PRV-delete UL10  cgcgccggccgcgcgccgcgcgtgtatataaactgtgtacacggcacccgcccgcgtcagtgtcttctttcgctcgcgtcgccatggaggtggacattgcgctgccgacgctgtccccgggggatctgtcggccctgcagaggtgcgagggccgcgtggtgtttctggagacgctgcggcgtcacgcgacgctgcgcgaggtcgcgctgccctgcggcggcgatgtgctcgcggccatggccgcgtaccggcggcgcttcgcggccgtcatcacgcgcgtgacgccgcaccgcatgctgg
                 ############################################################################################################################################################################################################################################################################################################


                      70810     70820     70830     70840     70850     70860     70870     70880     70890     70900     70910     70920     70930     70940     70950     70960     70970     70980     70990     71000     71010     71020     71030     71040     71050     71060     71070     71080     71090     71100
                 =========+=========+=========+=========+=========+=========+=========+=========+=========+=========+=========+=========+=========+=========+=========+=========+=========+=========+=========+=========+=========+=========+=========+=========+=========+=========+=========+=========+=========+=========+
PRV-WT complete  ccacgccgctcggcgtcggcggccgcgggcagtcgctggtgctgcagaacacggggcccttcgacctgaccaacggggaccacgtgtgcctcgtcccgccgctgctcggcgacgagtgcctgcggctcacgtccgccaacctggagctgcgcttccccatgacgctgccgctcgcccaggcccgcgagctgacggcgcgggtggtggcgcgcgccgtcgagacgctgcgcggcggcgcgcccgcgcggggcgccgacgtggtcttcaacaacggccggcgctaccagctgcccccgccgc
PRV-delete UL10  ccacgccgctcggcgtcggcggccgcgggcagtcgctggtgctgcagaacacggggcccttcgacctgaccaacggggaccacgtgtgcctcgtcccgccgctgctcggcgacgagtgcctgcggctcacgtccgccaacctggagctgcgcttccccatgacgctgccgctcgcccaggcccgcgagctgacggcgcgggtggtggcgcgcgccgtcgagacgctgcgcggcggcgcgcccgcgcggggcgccgacgtggtcttcaacaacggccggcgctaccagctgcccccgccgc
                 ############################################################################################################################################################################################################################################################################################################


                      71110     71120     71130     71140     71150     71160     71170     71180     71190     71200     71210     71220     71230     71240     71250     71260     71270     71280     71290     71300     71310     71320     71330     71340     71350     71360     71370     71380     71390     71400
                 =========+=========+=========+=========+=========+=========+=========+=========+=========+=========+=========+=========+=========+=========+=========+=========+=========+=========+=========+=========+=========+=========+=========+=========+=========+=========+=========+=========+=========+=========+
PRV-WT complete  accgcgacaacgccgaggcggccacgcgctcgctggtgctgaacatgatcttcctgctcaacgagggcgccgtgatcctgctgtcgctcatcccgaacctgctgacgctgggggcgcaggacggctacgccaacgccgtgatccagctgggcagcgccacgcgcgagctcggccagctggtgcggcagcccccgccgccgctgccgcaggaccacgcgcggcgcttctgcgtgttcgaggcgctggaggcctggatcgcctcggcctcgcggctgggcgacacgctcggcacgcggcccg
PRV-delete UL10  accgcgacaacgccgaggcggccacgcgctcgctggtgctgaacatgatcttcctgctcaacgagggcgccgtgatcctgctgtcgctcatcccgaacctgctgacgctgggggcgcaggacggctacgccaacgccgtgatccagctgggcagcgccacgcgcgagctcggccagctggtgcggcagcccccgccgccgctgccgcaggaccacgcgcggcgcttctgcgtgttcgaggcgctggaggcctggatcgcctcggcctcgcggctgggcgacacgctcggcacgcggcccg
                 ############################################################################################################################################################################################################################################################################################################


                      71410     71420     71430     71440     71450     71460     71470     71480     71490     71500     71510     71520     71530     71540     71550     71560     71570     71580     71590     71600     71610     71620     71630     71640     71650     71660     71670     71680     71690     71700
                 =========+=========+=========+=========+=========+=========+=========+=========+=========+=========+=========+=========+=========+=========+=========+=========+=========+=========+=========+=========+=========+=========+=========+=========+=========+=========+=========+=========+=========+=========+
PRV-WT complete  tggcgcgcgtgtgcatcttcgacggccccccgaccgtcccccccggcgagaaggccgcggtggtggaggtgtgacggcgcggcgccgagggggcccgtgtttcccccgccccaccccacccccctgcaataaaaaaatccgcgaaggaaaacaaagaaacgagtcgtgcgcgtctggcgtttattttccgggtcggggaaaatgggggcgtcggggggtggagggcggacggtggacggtggatgggggaagccccctcaggcaaacagcccgtcgggcagcgtgctcaggtgcacggcc
PRV-delete UL10  tggcgcgcgtgtgcatcttcgacggccccccgaccgtcccccccggcgagaaggccgcggtggtggaggtgtgacggcgcggcgccgagggggcccgtgtttcccccgccccaccccacccccctgcaataaaaaaatccgcgaaggaaaacaaagaaacgagtcgtgcgcgtctggcgtttattttccgggtcggggaaaatgggggcgtcggggggtggagggcggacggtggacggtggatgggggaagccccctcaggcaaacagcccgtcgggcagcgtgctcaggtgcacggcc
                 ############################################################################################################################################################################################################################################################################################################


                      71710     71720     71730     71740     71750     71760     71770     71780     71790     71800     71810     71820     71830     71840     71850     71860     71870     71880     71890     71900     71910     71920     71930     71940     71950     71960     71970     71980     71990     72000
                 =========+=========+=========+=========+=========+=========+=========+=========+=========+=========+=========+=========+=========+=========+=========+=========+=========+=========+=========+=========+=========+=========+=========+=========+=========+=========+=========+=========+=========+=========+
PRV-WT complete  atgacgagggccacggcgaggtcgtcggcgcagccgccgcgcttgccggtgaagacgcggtgctcgccggcccccgaggccgtctccacgaggttcccgagctgggcgttcaggtactcgacggggtcggcgcgcgcgcccacggtcacggacacgagctcctgcgaggccatgacggcgcccgagttgaactgcttcacgaaaaagtcgaaggcgggggtcttttgcttctgcagcaggaagaaggggtagcgcacggcgcccgcgcccgcgcccggggcgccgtggtggtagaagacc
PRV-delete UL10  atgacgagggccacggcgaggtcgtcggcgcagccgccgcgcttgccggtgaagacgcggtgctcgccggcccccgaggccgtctccacgaggttcccgagctgggcgttcaggtactcgacggggtcggcgcgcgcgcccacggtcacggacacgagctcctgcgaggccatgacggcgcccgagttgaactgcttcacgaaaaagtcgaaggcgggggtcttttgcttctgcagcaggaagaaggggtagcgcacggcgcccgcgcccgcgcccggggcgccgtggtggtagaagacc
                 ############################################################################################################################################################################################################################################################################################################


                      72010     72020     72030     72040     72050     72060     72070     72080     72090     72100     72110     72120     72130     72140     72150     72160     72170     72180     72190     72200     72210     72220     72230     72240     72250     72260     72270     72280     72290     72300
                 =========+=========+=========+=========+=========+=========+=========+=========+=========+=========+=========+=========+=========+=========+=========+=========+=========+=========+=========+=========+=========+=========+=========+=========+=========+=========+=========+=========+=========+=========+
PRV-WT complete  acggccgggtcggcgacgacgcgcgcggcgcgcagctccgcgagctcgcggcgcatggcgcccacgatggccacggccgagtcctggctgctgttgccctcgacggccacgcgcaccgccgagaaggcgccgtcgtgcagcgccagcacgcgcgccaggcagcgcacgacgcagcgcgcgatctcgtcggcggaggcgccggtgagcgcgcgcaggaaaaagtgctccaggccgagcacgatccagctgcgccggtagctgcccacgacggccacgccggtgccggaggcgcgcgcgttg
PRV-delete UL10  acggccgggtcggcgacgacgcgcgcggcgcgcagctccgcgagctcgcggcgcatggcgcccacgatggccacggccgagtcctggctgctgttgccctcgacggccacgcgcaccgccgagaaggcgccgtcgtgcagcgccagcacgcgcgccaggcagcgcacgacgcagcgcgcgatctcgtcggcggaggcgccggtgagcgcgcgcaggaaaaagtgctccaggccgagcacgatccagctgcgccggtagctgcccacgacggccacgccggtgccggaggcgcgcgcgttg
                 ############################################################################################################################################################################################################################################################################################################


                      72310     72320     72330     72340     72350     72360     72370     72380     72390     72400     72410     72420     72430     72440     72450     72460     72470     72480     72490     72500     72510     72520     72530     72540     72550     72560     72570     72580     72590     72600
                 =========+=========+=========+=========+=========+=========+=========+=========+=========+=========+=========+=========+=========+=========+=========+=========+=========+=========+=========+=========+=========+=========+=========+=========+=========+=========+=========+=========+=========+=========+
PRV-WT complete  gccgtgaaggccgggtccacgtacacgtgcagcgtgccggccagcagccggcggttggccaccgaggaggggcggtagagcaggaagcgctcgccggcggcgcgcgtgaggacgggcgcctcgcgcgcggccacggcgccgccgaggatctcctgcatgaaggagtccgggagcagccgctcggccgtggtgcgcgtggcggcgtccatggtgatgaagacgggcttgttgagcacgtagcacgagcaggcggtggcgtcggtgtgcgccaccacgcggcgcatgtgctcgtcgcagacg
PRV-delete UL10  gccgtgaaggccgggtccacgtacacgtgcagcgtgccggccagcagccggcggttggccaccgaggaggggcggtagagcaggaagcgctcgccggcggcgcgcgtgaggacgggcgcctcgcgcgcggccacggcgccgccgaggatctcctgcatgaaggagtccgggagcagccgctcggccgtggtgcgcgtggcggcgtccatggtgatgaagacgggcttgttgagcacgtagcacgagcaggcggtggcgtcggtgtgcgccaccacgcggcgcatgtgctcgtcgcagacg
                 ############################################################################################################################################################################################################################################################################################################


                      72610     72620     72630     72640     72650     72660     72670     72680     72690     72700     72710     72720     72730     72740     72750     72760     72770     72780     72790     72800     72810     72820     72830     72840     72850     72860     72870     72880     72890     72900
                 =========+=========+=========+=========+=========+=========+=========+=========+=========+=========+=========+=========+=========+=========+=========+=========+=========+=========+=========+=========+=========+=========+=========+=========+=========+=========+=========+=========+=========+=========+
PRV-WT complete  tacgtgaccacgttgagcatgtcggtgccgcggaggttggtgaggaagctggtgctggccttgccggtgttggtggaggagacgaagatgatcttgcagctggcctggttcatgaagccgaggatggtctgcacggcatcggggcgtataaagttggcctcgtcgacaaagagcaggttaaagtcctgtccgcggatcccctgtaacgacacgcggggagagagagggggggacggtgagcggcgccgcgatggacgcccacatcgccaacgagaccaagcagcagatgacgcgcttcgc
PRV-delete UL10  tacgtgaccacgttgagcatgtcggtgccgcggaggttggtgaggaagctggtgctggccttgccggtgttggtggaggagacgaagatgatcttgcagctggcctggttcatgaagccgaggatggtctgcacggcatcggggcgtataaagttggcctcgtcgacaaagagcaggttaaagtcctgtccgcggatcccctgtaacgacacgcggggagagagagggggggacggtgagcggcgccgcgatggacgcccacatcgccaacgagaccaagcagcagatgacgcgcttcgc
                 ############################################################################################################################################################################################################################################################################################################


                      72910     72920     72930     72940     72950     72960     72970     72980     72990     73000     73010     73020     73030     73040     73050     73060     73070     73080     73090     73100     73110     73120     73130     73140     73150     73160     73170     73180     73190     73200
                 =========+=========+=========+=========+=========+=========+=========+=========+=========+=========+=========+=========+=========+=========+=========+=========+=========+=========+=========+=========+=========+=========+=========+=========+=========+=========+=========+=========+=========+=========+
PRV-WT complete  gcccgcgctcgtgcacgtcatcgtgcccgaccccctcctgcggcgcgcgggcgtggacccgctcgcgcccttcgccgcccacgcgcagacgcgctaccacggctcgggcgtctgcgagccgtgggtctccgtgttcgcgggccacgtgcagacgggcgccgtcgagagcgtgctgacgctgccgccgctgcagcgcgcgcgcggccccggggggctgttcgtctcgctgcccctggcgctcggcgcccactttgacggcttcacggcggtggcgctgcgcgtgggcgcgcgcgagctgat
PRV-delete UL10  gcccgcgctcgtgcacgtcatcgtgcccgaccccctcctgcggcgcgcgggcgtggacccgctcgcgcccttcgccgcccacgcgcagacgcgctaccacggctcgggcgtctgcgagccgtgggtctccgtgttcgcgggccacgtgcagacgggcgccgtcgagagcgtgctgacgctgccgccgctgcagcgcgcgcgcggccccggggggctgttcgtctcgctgcccctggcgctcggcgcccactttgacggcttcacggcggtggcgctgcgcgtgggcgcgcgcgagctgat
                 ############################################################################################################################################################################################################################################################################################################


                      73210     73220     73230     73240     73250     73260     73270     73280     73290     73300     73310     73320     73330     73340     73350     73360     73370     73380     73390     73400     73410     73420     73430     73440     73450     73460     73470     73480     73490     73500
                 =========+=========+=========+=========+=========+=========+=========+=========+=========+=========+=========+=========+=========+=========+=========+=========+=========+=========+=========+=========+=========+=========+=========+=========+=========+=========+=========+=========+=========+=========+
PRV-WT complete  cttcacctacgacgagctcctgccggcccgcacgcgctacaacgtggacggcgagcgcctggagcgcctgtgccgccagttcgccaactacgcgcgcgcgcggcgcgtggccccggaggtcgcggccgcggggggccacatcgacgcgctgctgcccccggcggcggccaccatcgacggcgaggcgcagctgacgcgcgggggcttcgacgacccggcggcgccgcacgcgcgcgacgccgaccgcgagatcctgtcgctggtgcgccgcgcggccgagctcgtcgcggcgcggcaccc
PRV-delete UL10  cttcacctacgacgagctcctgccggcccgcacgcgctacaacgtggacggcgagcgcctggagcgcctgtgccgccagttcgccaactacgcgcgcgcgcggcgcgtggccccggaggtcgcggccgcggggggccacatcgacgcgctgctgcccccggcggcggccaccatcgacggcgaggcgcagctgacgcgcgggggcttcgacgacccggcggcgccgcacgcgcgcgacgccgaccgcgagatcctgtcgctggtgcgccgcgcggccgagctcgtcgcggcgcggcaccc
                 ############################################################################################################################################################################################################################################################################################################


                      73510     73520     73530     73540     73550     73560     73570     73580     73590     73600     73610     73620     73630     73640     73650     73660     73670     73680     73690     73700     73710     73720     73730     73740     73750     73760     73770     73780     73790     73800
                 =========+=========+=========+=========+=========+=========+=========+=========+=========+=========+=========+=========+=========+=========+=========+=========+=========+=========+=========+=========+=========+=========+=========+=========+=========+=========+=========+=========+=========+=========+
PRV-WT complete  ggtgcgcagccacgtggcgagcgggctgatgcagggcgccctcgcgcggcgcggcggggacggcggcggctccggggcgctcgaggcggccgcggcggtgcccgccgcgccgcgcgaggccgcgggcggcggcgacggcggcggcgccgcctggcgcgacgagctcctgctcacgccccaggacccccggccgctgacggcgctcgactggctggacgcgggctacgccgcgctcgcgggcggcgacgcgccggcgcacgtgtggcgccggcggcccgtgtccctggtggcgcgccgcca
PRV-delete UL10  ggtgcgcagccacgtggcgagcgggctgatgcagggcgccctcgcgcggcgcggcggggacggcggcggctccggggcgctcgaggcggccgcggcggtgcccgccgcgccgcgcgaggccgcgggcggcggcgacggcggcggcgccgcctggcgcgacgagctcctgctcacgccccaggacccccggccgctgacggcgctcgactggctggacgcgggctacgccgcgctcgcgggcggcgacgcgccggcgcacgtgtggcgccggcggcccgtgtccctggtggcgcgccgcca
                 ############################################################################################################################################################################################################################################################################################################


                      73810     73820     73830     73840     73850     73860     73870     73880     73890     73900     73910     73920     73930     73940     73950     73960     73970     73980     73990     74000     74010     74020     74030     74040     74050     74060     74070     74080     74090     74100
                 =========+=========+=========+=========+=========+=========+=========+=========+=========+=========+=========+=========+=========+=========+=========+=========+=========+=========+=========+=========+=========+=========+=========+=========+=========+=========+=========+=========+=========+=========+
PRV-WT complete  ctaccagaccggcgagacgttcgtcgtcgtcgcctacgagcactccaccgcctggggcggccggcgcgggcccgcgagcgagcccctggcgcgcgtgctcgccgaggagtgcgagcgccacggcgtcgagcacccgcgcgccctgcccgcggaggcgcggctggagctggtgcggcggcacgccgagctggccgtgccgctgggggacgaggagccgccgctgccggtctttgacgccaccgcggagctggtgctgctggagcgcttccgcaacgcctgcgtgcgcgcgctgctggccgg
PRV-delete UL10  ctaccagaccggcgagacgttcgtcgtcgtcgcctacgagcactccaccgcctggggcggccggcgcgggcccgcgagcgagcccctggcgcgcgtgctcgccgaggagtgcgagcgccacggcgtcgagcacccgcgcgccctgcccgcggaggcgcggctggagctggtgcggcggcacgccgagctggccgtgccgctgggggacgaggagccgccgctgccggtctttgacgccaccgcggagctggtgctgctggagcgcttccgcaacgcctgcgtgcgcgcgctgctggccgg
                 ############################################################################################################################################################################################################################################################################################################


                      74110     74120     74130     74140     74150     74160     74170     74180     74190     74200     74210     74220     74230     74240     74250     74260     74270     74280     74290     74300     74310     74320     74330     74340     74350     74360     74370     74380     74390     74400
                 =========+=========+=========+=========+=========+=========+=========+=========+=========+=========+=========+=========+=========+=========+=========+=========+=========+=========+=========+=========+=========+=========+=========+=========+=========+=========+=========+=========+=========+=========+
PRV-WT complete  cgtccgcgagtccgtgcgccgcgagccgcgcatgcgccagatcatcgagttcgcgatccgcccgcgcgaccgcgaggccgtcctcgacgtggccgggcgcgccccggcgctgctggacgcgttcgcgcggcgcctcgagcagacgccggcgcgggagatggtcgactcgggcctgatgacggccgccgcggcgcacctcgccgcgcgcgccaccgccggctacgtgaccttcgagagcggcccgctgctgggcggcgttttcctcttcgactactacagcgccggcggggaggttataag
PRV-delete UL10  cgtccgcgagtccgtgcgccgcgagccgcgcatgcgccagatcatcgagttcgcgatccgcccgcgcgaccgcgaggccgtcctcgacgtggccgggcgcgccccggcgctgctggacgcgttcgcgcggcgcctcgagcagacgccggcgcgggagatggtcgactcgggcctgatgacggccgccgcggcgcacctcgccgcgcgcgccaccgccggctacgtgaccttcgagagcggcccgctgctgggcggcgttttcctcttcgactactacagcgccggcggggaggttataag
                 ############################################################################################################################################################################################################################################################################################################


                      74410     74420     74430     74440     74450     74460     74470     74480     74490     74500     74510     74520     74530     74540     74550     74560     74570     74580     74590     74600     74610     74620     74630     74640     74650     74660     74670     74680     74690     74700
                 =========+=========+=========+=========+=========+=========+=========+=========+=========+=========+=========+=========+=========+=========+=========+=========+=========+=========+=========+=========+=========+=========+=========+=========+=========+=========+=========+=========+=========+=========+
PRV-WT complete  ggtgacgcgggccccgccggccgtggcagtggagccggcgacgcgcgggcagttcgcgtgccgcttccgcggcgcgtcgcatcgctgtctcccgggcgagagctacgcgtacctctgcgtcggcgtgtcgcgggacctgcgcgcgctggtggtgctgcccggcggcttcggcttcttcgcggccctgcggctggagtggcccgccgcgctggtggaccccgtgctcgagcgcctgtgccgccgcgtgtagcgagggtgggcgcgacgagcgccgccatggaggtggccgcggccctgacg
PRV-delete UL10  ggtgacgcgggccccgccggccgtggcagtggagccggcgacgcgcgggcagttcgcgtgccgcttccgcggcgcgtcgcatcgctgtctcccgggcgagagctacgcgtacctctgcgtcggcgtgtcgcgggacctgcgcgcgctggtggtgctgcccggcggcttcggcttcttcgcggccctgcggctggagtggcccgccgcgctggtggaccccgtgctcgagcgcctgtgccgccgcgtgtagcgagggtgggcgcgacgagcgccgccatggaggtggccgcggccctgacg
                 ############################################################################################################################################################################################################################################################################################################


                      74710     74720     74730     74740     74750     74760     74770     74780     74790     74800     74810     74820     74830     74840     74850     74860     74870     74880     74890     74900     74910     74920     74930     74940     74950     74960     74970     74980     74990     75000
                 =========+=========+=========+=========+=========+=========+=========+=========+=========+=========+=========+=========+=========+=========+=========+=========+=========+=========+=========+=========+=========+=========+=========+=========+=========+=========+=========+=========+=========+=========+
PRV-WT complete  gaggactttgccgcctggcggctgctgcgctccgactcgcgcgtcaaggtctacgccgccctcggcgtggtgggcgcgcgcctggccgccgtggcgccgggcgccgcgaccgtcgacgcgcgcgtctacctgacgcgcccgcgggccctgcgcctggcccagggccgcttccacgtcgtggtcctgctcaacgacgccgcctacgcgctggtggcggccgtgaccacgaccacgctccgcgggagcggcggcgagctcgtgcgcctgacgctgggcgacgcgagcctcgaggccctgccc
PRV-delete UL10  gaggactttgccgcctggcggctgctgcgctccgactcgcgcgtcaaggtctacgccgccctcggcgtggtgggcgcgcgcctggccgccgtggcgccgggcgccgcgaccgtcgacgcgcgcgtctacctgacgcgcccgcgggccctgcgcctggcccagggccgcttccacgtcgtggtcctgctcaacgacgccgcctacgcgctggtggcggccgtgaccacgaccacgctccgcgggagcggcggcgagctcgtgcgcctgacgctgggcgacgcgagcctcgaggccctgccc
                 ############################################################################################################################################################################################################################################################################################################


                      75010     75020     75030     75040     75050     75060     75070     75080     75090     75100     75110     75120     75130     75140     75150     75160     75170     75180     75190     75200     75210     75220     75230     75240     75250     75260     75270     75280     75290     75300
                 =========+=========+=========+=========+=========+=========+=========+=========+=========+=========+=========+=========+=========+=========+=========+=========+=========+=========+=========+=========+=========+=========+=========+=========+=========+=========+=========+=========+=========+=========+
PRV-WT complete  gccgacctgcccgtcgccgagcccgtgcccgccgcgccggtgagccgcctggacctggacgccgccgagcccgtgtccgcggcgcccgcgcgccgccgcgagtgcgtggtcctggcccccggcgcgtggtggtcgcgcgaccgcatctactttctgcagatggacccggcgctgctggcgctgtgccccgccggctggcgcgcgcgccacctcggcgccgtgctcgccgggctgctgagcccccgcgacgacgacggcggcagctgctgccgcgagtgccgcgtggaccacgtggacgcg
PRV-delete UL10  gccgacctgcccgtcgccgagcccgtgcccgccgcgccggtgagccgcctggacctggacgccgccgagcccgtgtccgcggcgcccgcgcgccgccgcgagtgcgtggtcctggcccccggcgcgtggtggtcgcgcgaccgcatctactttctgcagatggacccggcgctgctggcgctgtgccccgccggctggcgcgcgcgccacctcggcgccgtgctcgccgggctgctgagcccccgcgacgacgacggcggcagctgctgccgcgagtgccgcgtggaccacgtggacgcg
                 ############################################################################################################################################################################################################################################################################################################
[truncated: 363,101 more chars]
